# Genome-Scale Metabolic Model Reconstruction and in Silico Investigations of Methane Metabolism in *Methylosinus trichosporium* OB3b

**DOI:** 10.3390/microorganisms8030437

**Published:** 2020-03-20

**Authors:** Sanzhar Naizabekov, Eun Yeol Lee

**Affiliations:** Department of Chemical Engineering, Kyung Hee University, Gyeonggi-do 17104, Korea; snaizabekov@nu.edu.kz

**Keywords:** *Methylosinus trichosporium*, genome-scale metabolic model, C1 metabolism, methane, methanotroph

## Abstract

*Methylosinus trichosporium* OB3b is an obligate aerobic methane-utilizing alpha-proteobacterium. Since its isolation, *M. trichosporium* OB3b has been established as a model organism to study methane metabolism in type II methanotrophs. *M. trichosporium* OB3b utilizes soluble and particulate methane monooxygenase (sMMO and pMMO respectively) for methane oxidation. While the source of electrons is known for sMMO, there is less consensus regarding electron donor to pMMO. To investigate this and other questions regarding methane metabolism, the genome-scale metabolic model for *M. trichosporium* OB3b (model ID: iMsOB3b) was reconstructed. The model accurately predicted oxygen: methane molar uptake ratios and specific growth rates on nitrate-supplemented medium with methane as carbon and energy source. The redox-arm mechanism which links methane oxidation with complex I of electron transport chain has been found to be the most optimal mode of electron transfer. The model was also qualitatively validated on ammonium-supplemented medium indicating its potential to accurately predict methane metabolism in different environmental conditions. Finally, in silico investigations regarding flux distribution in central carbon metabolism of *M. trichosporium* OB3b were performed. Overall, iMsOB3b can be used as an organism-specific knowledgebase and a platform for hypothesis-driven theoretical investigations of methane metabolism.

## 1. Introduction

The Gram-negative *Methylosinus trichosporium* OB3b belongs to an obligate aerobic methane oxidizing alpha-proteobacterium (type II methanotrophs) [1]. After it was isolated by Roger Whittenbury in 1970, *M. trichosporium* OB3b has been established as a model organism used to study methanotrophic metabolism [2]. In particular, it has been used to study the structure and function of key enzymes of methane oxidation such as an extracellular copper chelator methanobactin, and both soluble and particulate methane monooxygenases because it possesses two different types of methane monooxygenase: a membrane-bound, particulate methane monooxygenase (pMMO), and, its soluble form, soluble methane monooxygenase (sMMO) [2,3,4,5,6,7,8,9,10,11,12,13]. The structure and function of methane monooxygenase, an enzyme responsible for the oxidation of methane to methanol, however, remains to be one of the most intriguing questions in methanotrophy.

The activation of pMMO and sMMO depends on the extracellular copper concentration. sMMO is expressed in medium with either low or no copper concentrations, whilst pMMO is expressed in mediums with relatively high copper concentrations [14,15]. Both these enzymes need an electron donor to convert methane to methanol. While there is a consensus regarding electron donor for sMMO which is NADH [11,16], there are still debates regarding electron donor to pMMO.

**Figure 1:**
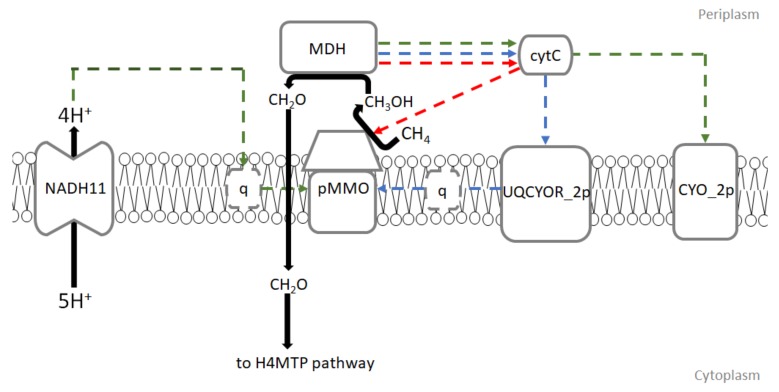
Modes of electron transfer to pMMO in iMsOB3b model developed in this study. The dotted lines represent possible electron flow in 3 different modes of electron transfer. The green dotted lines refer to electron flow in redox-arm mode. The blue dotted lines refer to electron flow in uphill electron transfer mode. The red dotted lines represent electron flow during direct coupling mode. pMMO refers to particulate methane monooxygenase, q refers to ubiquinone, cytC refers to cytochrome C, NADH11 refers to ubiquinone oxidoreductase, UQCYOR_2p refers to ubiquinol-cytochrome-c reductase, CYO_2p refers to cytochrome C oxidase, H4MTP refers to tetrahydromethanopterin pathway.

Three potential modes of electron transfer have been proposed, none of which cannot be ruled out completely (Figure 1):

1. Redox-arm mode: In this mode, ubiquinol is the electron donor for methane oxidation while methanol dehydrogenase (MDH) gives electrons via cytochrome c directly to complex IV. This leads up to proton motive force and ATP production. Recently, this mode of electron transfer has been suggested to be the main mode in *Methylocystis*, another type II methanotroph genus [17,18].
2. Direct coupling mode: In this mode, methanol dehydrogenase passes electrons directly to pMMO with cytochrome c as an electron donor for methane oxidation.
3. In uphill electron transfer, a reverse electron flow from cytochrome c to ubiquinone via ubiquinol-cytochrome-c reductase occurs. This mode is expected to significantly reduce efficiency of electron transport chain in methanotrophs.

In addition to the physiological studies described above, several attempts of industrial applications of *M. trichosporium* OB3b have been investigated as well. A few examples are biodegradation of recalcitrant hydrocarbons like trichloroethylene and attempts to produce polyhydroxybutyrate (PHB), an important biodegradable polymer [19,20,21,22,23,24,25,26]. The production of PHB is of particular interest because *M. trichosporium* OB3b has been shown to produce PHB up to 67% of its dry cell weight, the highest PHB content reported for a methane-utilizing organism [27]. 

Recent concerns regarding global warming and a drop in natural gas price due to hydraulic fracturing has led to a spike of interest in methane utilizing organisms [28]. This, along with the new development of genetic manipulation tools for *M. trichosporium* OB3b, makes it a promising platform for industrial applications [29].

A genome scale metabolic model (GEM) is used to study and optimize biological systems. It allows to both compile a knowledgebase of the organism of interest and test hypothesis of interest in silico [30]. Here, we provide first manually curated GEM for *M. trichosporium* OB3b (model ID: iMsOB3b), which can be used for hypothesis-driven metabolic investigations and metabolic engineering purposes. Here, we try to investigate type of mode of methane oxidation which is active in *M. trichosporium* OB3b by comparing in silico predictions with the available experimental data. We have found that redox-arm seems to be the most optimal mode of methane oxidation which agrees with previous findings. We further validate the model, both quantitatively and qualitatively, by comparing in silico growth rate predictions with the available experimental data. Finally, we describe in silico flux distribution predictions in iMsOB3b to check several hypotheses about its central carbon metabolism. 

## 2. Materials and Methods 

### 2.1. Model Reconstruction and Curation

The updated version of GEM of the related organism, *Methylorubrum extorquens* AM1, and the genome sequence of *M. trichosporium* OB3b (Genbank assembly: GCA_002752655.1, BioProject: PRJNA413061) were used to generate the draft model [31,32]. In particular, InParanoid analysis between genomes of *M. extorquens* AM1 (Genbank assembly: GCA_000022685.1, BioProject: PRJNA20) and *M. trichosporium* OB3b was run and reactions with gene reaction rules identified as orthologs were kept in the draft model based on accelerated genome-scale reconstruction protocol described previously [33,34]. The gap-filling reactions that were necessary for flux towards a biomass equation were kept in the draft model even if they did not have orthologs as gene reaction rules. 

The resultant draft model was manually checked for gene-reaction associations by paying special attention to central carbon metabolism, nitrogen metabolism and electron transport chain. Literature-specific information and the KEGG database were used to construct and refine selected biochemical pathways in the draft model [35,36,37,38,39]. In addition, 44 OB3b-specific reactions which were initially missing in the draft model were added based on manual exploration of *M. trichosporium* OB3b’s Genbank genome annotations.

Several modifications of the draft model were also performed:

1. To keep reactions balanced and to avoid metabolite production out of nowhere, reactions in the draft model were checked for mass and charge balance;
2. To avoid unfeasible ATP production in the model, only reactions related to electron transport chain and lower glycolysis (phosphoglycerate kinase and pyruvate kinase) were allowed to produce ATP;
3. To avoid proton flux outside the cell, only reactions of electron transport chain or reactions linked to some energy cost were allowed to have flux towards exterior of the cell;
4. To make the draft model user-friendly and comparable with other models, all reaction and metabolite IDs were changed to BiGG Models IDs [40].

### 2.2. Applied Constraints

To find the relevant mode of methane oxidation in *M. trichosporium* OB3b, flux balance analysis solutions were quantitatively and/or qualitatively compared against experimental values from the literature [17,18,36,41,42,43,44,45,46,47,48]. The literature information provided several values of specific methane uptake rates, oxygen:methane molar uptake ratios, specific growth rates under different nitrogen sources (ammonium, nitrate or molecular nitrogen) and/or copper concentrations which directly affect type of methane monooxygenase activated (either sMMO or pMMO).

Since there was no specific methane uptake rate available for the highest specific growth rate measurement available for *M. trichosporium* OB3b, the methane uptake rate was limited to allow mostly 14.9 mmol gCDW^−1^ h^−1^, a value adapted from the closely related organism, *Methylocystis parvus* OBBP [17]. The uptake rates of other medium metabolites were left unconstrained.

The adaption of template biomass equations from semi-automatic reconstruction workflows (e.g., ModelSEED) may lack non-universal cofactors and other organism-specific biomass precursors which, in turn, may affect in silico growth rate predictions [49]. Since there is no measured biomass composition for *M. trichosporium* OB3b yet, the biomass equation was adapted from *M. extorquens* AM1 instead. 

Flux maximization via a biomass equation was used as a model objective for parsimonious flux balance analysis (pFBA) with which in silico simulations were performed. pFBA was used for in silico calculations because it assumes that the cell tries to minimize its overall flux whilst still maximizing the growth rate. This decrease in the overall flux leads to the smaller number of active reactions in the model which, in turn, should provide better approximation of flux distribution than the regular FBA [50]. 

The non-growth associated maintenance (NGAM) value has been shown to affect in silico growth predictions in some cases [30]. Since the non-growth associated maintenance pseudo-reaction is unconstrained in *M. extorquens* AM1 and there is no measured NGAM value for *M. trichosporium* OB3b, the value (3.5 mmol gCDW^−1^ h^−1^) for non-growth associated maintenance has been adapted from *M. parvus* OBBP [17].

Since each mode of methane oxidation could be represented by specific reactions in the model (model reaction IDs: redox-arm = PMMOipp, direct coupling = PMMODCipp, and uphill electron transfer = UQCYOR_2p and PMMOipp), the switch between redox-arm and direct coupling modes of methane oxidation was done by allowing flux through each corresponding reaction. To allow an uphill electron transfer mode, ubiquinol-cytochrome-c reductase (model reaction ID: UQCYOR_2p) was allowed to proceed in the reverse direction whilst keeping PMMOipp active at the same time.

### 2.3. Used Software

Cobrapy, a python version of the popular Cobratoolbox software, was used for the described model manipulations. The model was developed and tested in the Cobrapy-compatible json format and systems biology community standard sbml format [51]. The Escher web-tool was used for the visualization of selected biochemical pathways and their relevant fluxes [52]. The model files are available online at https://github.com/ensakz/gem_methylosinus_trichosporium.

## 3. Results and Discussion

### 3.1. Nitrogen Metabolism

**Figure 2:**
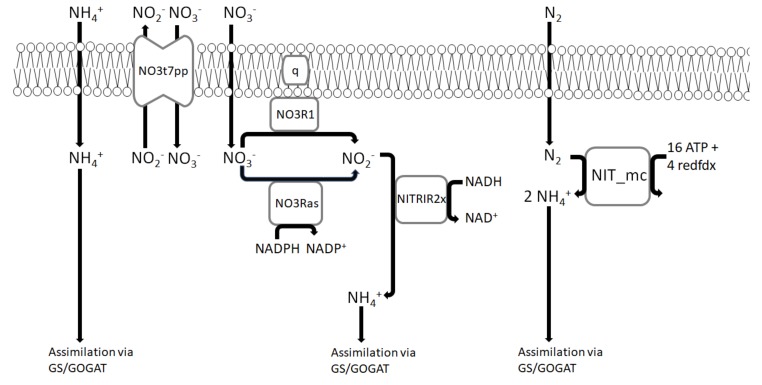
Nitrogen assimilation and fixation pathways implemented in iMsOB3b. NO3t7pp refers to nitrate transporter via nitrite antiport, q refers to ubiquinone. NO3R1 refers to NADP-dependent nitrate reductase, NO3Ras refers to ubiquinol-dependent nitrate reductase, NITRIR2x refers to NAD-dependent nitrite reductase, NIT_mc refers to nitrogenase, and redfdx refers to reduced ferredoxin.

Besides copper, nitrogen metabolism has been also shown to affect pMMO activity [35,53]. To account for this effect on the model’s in silico predictability, nitrogen metabolism pathways have been added to the model (Figure 2). 

The *M. trichosporium* OB3b model can utilize nitrogen sources such as nitrate (NO_3_^−^) and ammonium (NH_4_^+^) in different ways. The model possesses two potential ammonium assimilation reactions: glutamine synthetase (model reaction ID: GLNS) and NAD/NADP-dependent glutamate dehydrogenase (model reaction IDs: GLUDxi and GLUDy). Since ammonium assimilation has been shown to occur exclusively via glutamine synthetase/glutamate synthase (GS/GOGAT) pathway, the directionality of NAD/NADP-dependent glutamate dehydrogenases has been reversed to allow ammonium production only [53]. In addition to ammonium assimilation, the model can convert ammonium into hydroxylamine and nitrite by the mechanisms which are described above. 

Nitrate assimilation can be done by its conversion into nitrite via ubiquinol or NADP-dependent nitrate reductase (model reaction IDs: NO3Ras or NO3R1), followed by the subsequent reduction via NAD-dependent nitrite reductase (model reaction ID: NTRIR2x). 

*M. trichosporium* OB3b has been also shown to utilize molecular nitrogen [36,54]. The corresponding nitrogenase reaction was added to the model (model reaction ID: NIT_mc). 

### 3.2. Determination of Mode of Electron Transfer to pMMO

The literature values were used to constrain the genome-scale model to determine the type of electron transfer which is active in *M. trichosporium* OB3b. Since each mode of methane oxidation is related to the way pMMO receives electrons, the available data of *M. trichosporium* OB3b growth in high-copper medium with nitrate added were used. The oxygen:methane consumption ratio was chosen to be the key parameter for the determination of electron transfer mode in *M. trichosporium* OB3b since it is a function of the electron transfer mode to pMMO. The oxygen:methane molar consumption ratios for the nitrate high-copper medium was 1.48 ± 0.02 [42].

**Figure 3:**
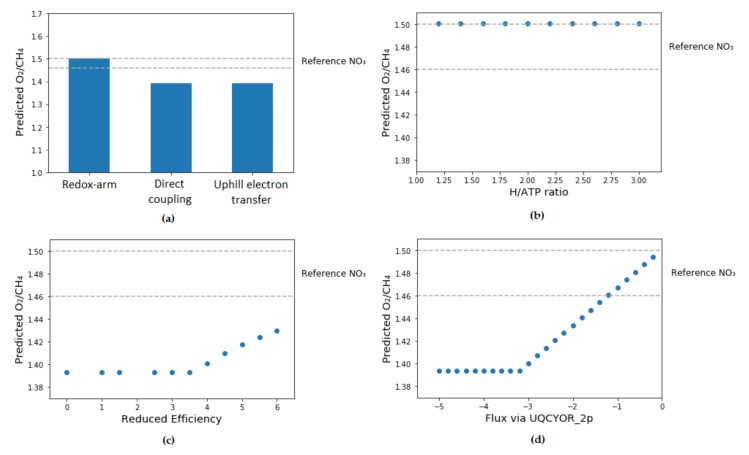
Parameter fitting. The grey dotted lines refer to literature values for oxygen:methane molar consumption ratio ranges on high-copper nitrate medium. (**a**) Comparison of predicted O_2_/CH_4_ molar ratios between 3 modes of electron transfer to pMMO, with no additional parameter fitting. (**b**) For redox-arm mode, the number of protons necessary for the synthesis of 1 mol ATP was iterated. There was no change in the predicted O_2_/CH_4_ molar ratio with all values fitting into experimental range. (**c**) For direct-coupling mode, a part of the flux was constrained to go via PMMOipp. Despite a slight improvement in the predicted O_2_/CH_4_ molar ratio, this value is still below experimentally determined range. (**d**) For uphill electron transfer mode, the reverse flux via ubiquinol-cytochrome c reductase was fixed at different values. The predicted O_2_/CH_4_ molar ratios felt into experimental values when reverse reaction flux was in 0.20–1.20 mmol gDCW^−1^ h^−1^ range.

The available oxygen:methane molar consumption ratios were compared with each variant of the model which was constrained to one of the electron transfer modes exclusively. Among the three modes of methane oxidation, only redox-arm has shown oxygen:methane values within the range of experimental values (1.5 in nitrate-supplemented high-copper medium). To further investigate the type of electron transfer which is active in *M. trichosporium*, the efficiency of each mode of electron transfer was altered (Figure 3).

For redox-arm, the number of moles of ATP produced per 1 mole of hydrogen transferred via electron transport system was altered. This did not change the predicted values for the oxygen:methane specific uptake rate suggesting that the system is reducing power limited rather than energy limited. Interestingly, these results are consistent with the similar simulations performed with type I methanotroph models [35,55].

For direct coupling, the reaction’s ratio constraints were used to force a portion of the flux via regular pMMO while keeping direct coupling mode active. Even when the efficiency of direct coupling mode was significantly reduced, the maximum determined oxygen:methane ratio was still 1.43 which is below the experimental values. Overall, it seems that direct coupling mode is not supported by the data.

For uphill electron transfer, the maximum reverse electron flow via ubiquinol-cytochrome-c reductase reaction was controlled by iteratively constraining the reaction’s lower boundary. To achieve values which meet the minimum range for experimental data (1.46), a significant drop in the flux of uphill electron flow was required (from the unbounded flux of 3.21 mmol gDCW^−1^ h^−1^ to at least 1.20 mmol gDCW^−1^ h^−1^) so that the model behavior started to resemble redox-arm mode. 

Overall, redox-arm mode seems to be more likely mode of electron transfer to pMMO in type II methanotrophs as its predictions felt within the range of experimental values without additional parameter fitting. Nevertheless, the possibility of highly inefficient uphill electron transfer mode cannot be ruled out completely as it also fits experimental oxygen: methane molar ratios. It is worth mentioning that genome-scale models for the related *Methylocystis* family have shown redox-arm as the main mode of electron transfer [17,18]. Furthermore, the possibility of redox-arm mode is further supported by the reports that quinols are effective to provide reducing equivalents to pMMO in membrane fractions isolated from *M. trichosporium* OB3b [56,57]. 

### 3.3. Model Validation and Predictions under Different Environmental Conditions

To further validate genome-scale model, the maximum reported specific growth rate of *M. trichosporium* OB3b on copper-supplemented nitrate medium was compared against model prediction. The literature value was 0.126 h^−1^ [41]. The model’s in silico growth rate prediction with the assumption of redox-arm mode of electron transfer was 0.123 h^−1^ which is close to the literature value (Table 1).

The model prediction for the maximum specific growth rate on nitrate medium without copper were above the maximum reported literature value (0.109 h^−1^ and 0.097 h^−1^ respectively, Table 1). Nevertheless, the published data regarding the comparison between *M. trichosporium* OB3b growth rates in copper-added and copper-free nitrate medium is inconsistent with some studies showing higher specific growth rates in copper-added and other studies showing higher specific growth rates in copper-free medium [42,43,44,47]. It is worth mentioning that the maximum recorded specific growth rate values for both copper-added and copper-free nitrate mediums come from the same study [41]. This implies that the same controlled batch culture conditions were used to measure specific growth rates, thus making these values statistically comparable. One of the possible reasons for the model’s higher than predicted growth rate might be relatively poor functional annotation of the *M. trichosporium* OB3b genome and proteome. For example, there are only nine manually reviewed proteins in the *M. trichosporium* OB3b UniProt database, which is far below the 459 manually reviewed proteins for *Methylococcus capsulatus* Bath for which the genome-scale model has been reconstructed previously [58,59]. As the quality of genome-scale models significantly depends on annotations, these poor annotations may leave many crucial biochemical pathways unincluded in the genome-scale model, which, in turn, may affect in silico specific growth rate predictions. 

*M. trichosporium* OB3b has been shown to grow at a faster rate under a ammonium medium than under a nitrate medium, both in copper-added and copper-free mediums [42,48]. Nevertheless, the maximum reported value for *M. trichosporium* OB3b’s specific growth rate under copper-supplemented ammonium medium is lower than those under copper-supplemented nitrate medium (0.123 h^−1 vs.^ 0.126 h^−1^ respectively) [48]. One potential explanation for the observed phenomenon is that different batch culture conditions (for example, different mixing rates) were used in different studies, making them quantitatively incomparable. Bacterial cultivation under methane-limited continuous culture conditions is expected to provide more reliable representation of the steady-state bacterial growth [60,61]. In the case of copper-free ammonium-supplemented medium, there is no reported values of specific growth rates for *M. trichosporium* OB3b. Nevertheless, despite these inconsistencies in the literature data, the model, qualitatively, should predict a higher growth rate under ammonium than the nitrogen source both in copper-added and copper-free mediums. 

Since the nitrogen oxidation state in nitrate is higher than those in amino acids, nitrate should be reduced to ammonium before it can be incorporated into amino acids. This, theoretically, should lead to higher and faster growth under ammonium-supplied medium than the nitrate-supplied medium. Nevertheless, ammonium has been shown to competitively inhibit pMMO via ammonium oxidation to hydroxylamine, which is toxic for bacterial growth [62,63]. Therefore, in order to have higher grow rates under ammonium-supplemented medium, *M. trichosporium* OB3b should possess either one of the following biochemical pathways:

1. In the first pathway, hydroxylamine is oxidized to nitrite via hydroxylamine oxidoreductase which is then reduced to nitric oxide and nitrous oxide. Despite the fact that no gene for hydroxylamine oxidoreductase was found in *M. trichosporium* OB3b genome, this reaction (model reaction ID: HAORipp) was added due to multiple reports of nitric oxide and nitrous oxide production by *M. trichosporium* OB3b [42,64].
2. In the second pathway, the formed hydroxylamine is reduced back to ammonium. The genome of *M. trichosporium* OB3b possesses a gene for hydroxylamine reductase (CQW49_14985) and the corresponding reaction (model reaction ID: HAMR) was added to the model.

In silico simulations with ammonium as the nitrogen source have shown specific growth rate values higher than those with nitrate as the nitrogen source for a copper-added medium. The values for the predicted specific growth rates were 0.178 h^−1^ and 0.131 h^−1^ respectively (Table 1). 

The maximum reported specific growth rate of *M. trichosporium* OB3b with molecular nitrogen supplied was 0.023 h^−1^ [41]. Nevertheless, the model cannot predict any in silico growth with the constraints of molecular nitrogen as nitrogen source (Table 1). Since the nitrogenase reaction has not been characterized in *M. trichosporium* OB3b yet, it was adapted from *Geobacter metallireducens*. One possible explanation for the observed discrepancy can be that ATP requirements of the adapted nitrogenase reaction are too high (16 ATPs are required for reduction of 1 mol of N_2_) and do not consider *M. trichosporium* OB3b’s energetic requirements. 

### 3.4. Flux Distribution in Central Carbon Metabolism

Since the copper-supplemented nitrate medium was the only environmental condition in which model was validated against the available experimental data, this condition was used for the subsequent simulations of *M. trichosporium* OB3b’s central carbon metabolism. This is consistent with some other published genome-scale models of methanotrophs which have been verified under one particular environmental condition [55,65].

**Figure 4:**
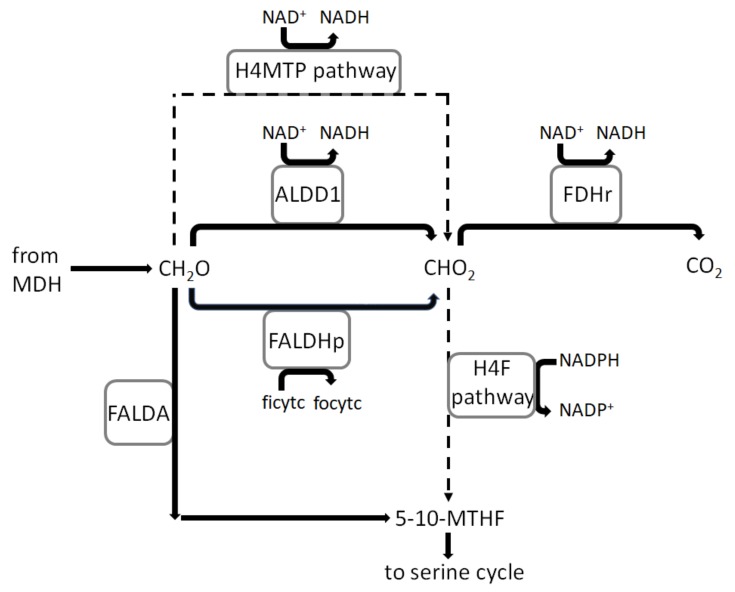
Formaldehyde and formate oxidation and assimilation pathways as implemented in iMsOB3b. The continuous lines represent a single reaction, while the dashed lines represent a series of reaction. ALDD1 refers to non-specific aldehyde dehydrogenase, FALDHp refers to formaldehyde dehydrogenase, 5-10-MTHF refers to 5-10-methylenetetrahydrofolate.

Following the path of methane monooxygenase and methanol dehydrogenase, the central carbon metabolism includes three different pathways for formaldehyde oxidation into formate with the tetrahydromethanopterin (H4MTP)-linked pathway being the main pathway (Figure 4). Formate is expected to be the key molecule which is responsible for carbon assimilation and dissimilation in type II methanotrophs [37]. The assimilation occurs via the tetrahydrofolate (H4F) pathway via a series of reactions that convert formate into methylene tetrahydrofolate. The dissimilation should occur via NAD-dependent formate dehydrogenase (model reaction ID: FDHr), which converts formate oxidation into carbon dioxide [38]. The model also contains a spontaneous reaction for formaldehyde assimilation (model reaction ID: FALDA) which has been made inactive since the condensation of formaldehyde with tetrahydrofolate, which results in methylene tetrahydrofolate, has been shown to be insignificant in closely-related methylotrophs [66]. 

Methylene tetrahydrofolate serves as an entry point for the serine cycle. In the serine cycle, methylene tetrahydrofolate combines with the glycine generated from glyoxylate to form C3 compounds such as 2-phosphoglycerate which are later carboxylated to form C4 compounds such as oxaloacetate. The model contains ethylmalonyl-CoA (EMC) pathway variant of the serine cycle as the number of multi-omics studies have shown it as the primary pathway for glyoxylate regeneration [36,37]. 

**Figure 5:**
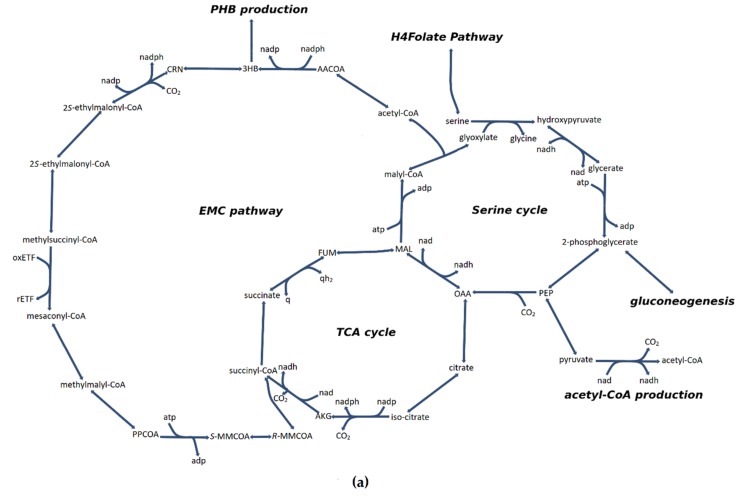

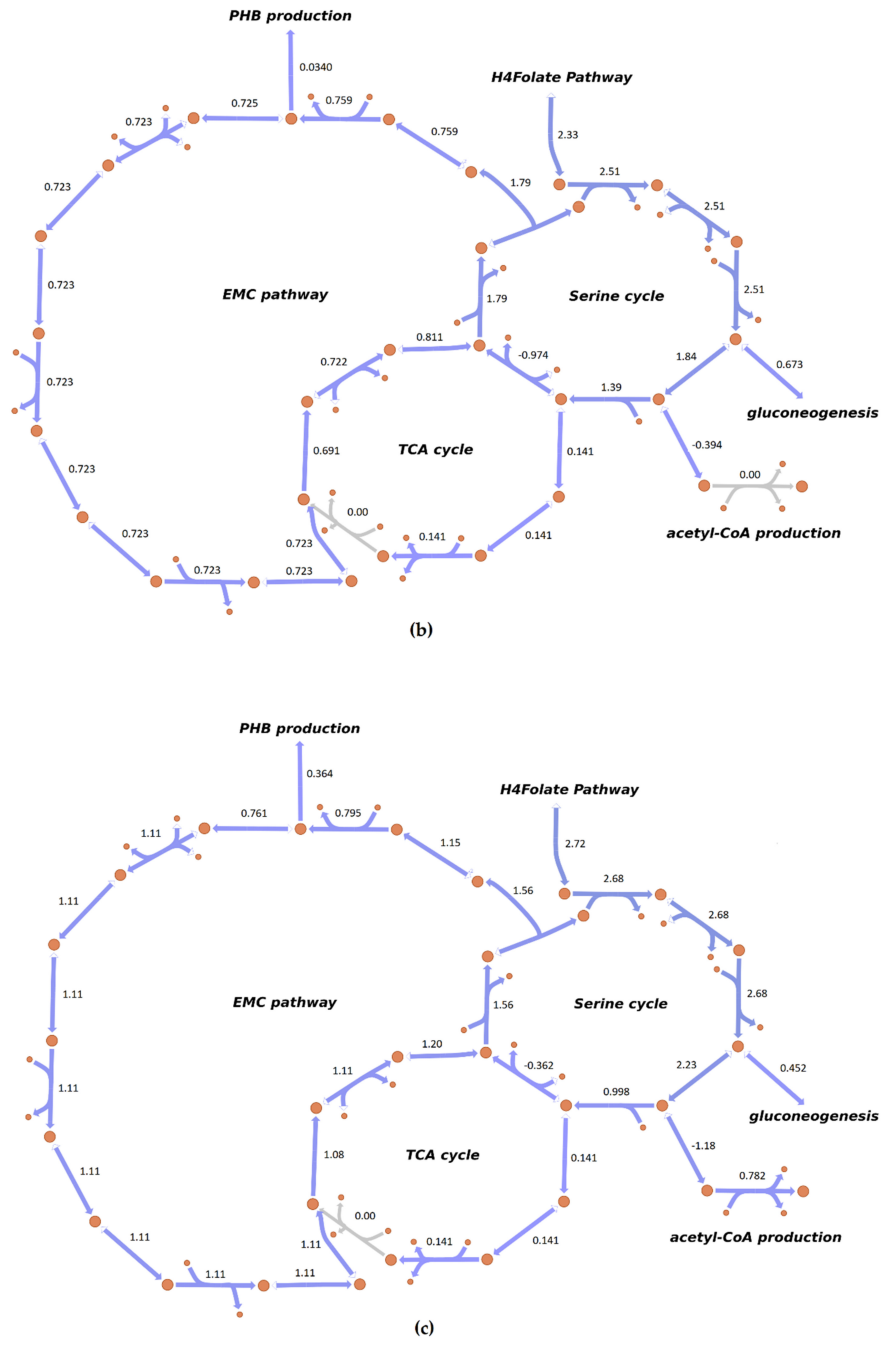

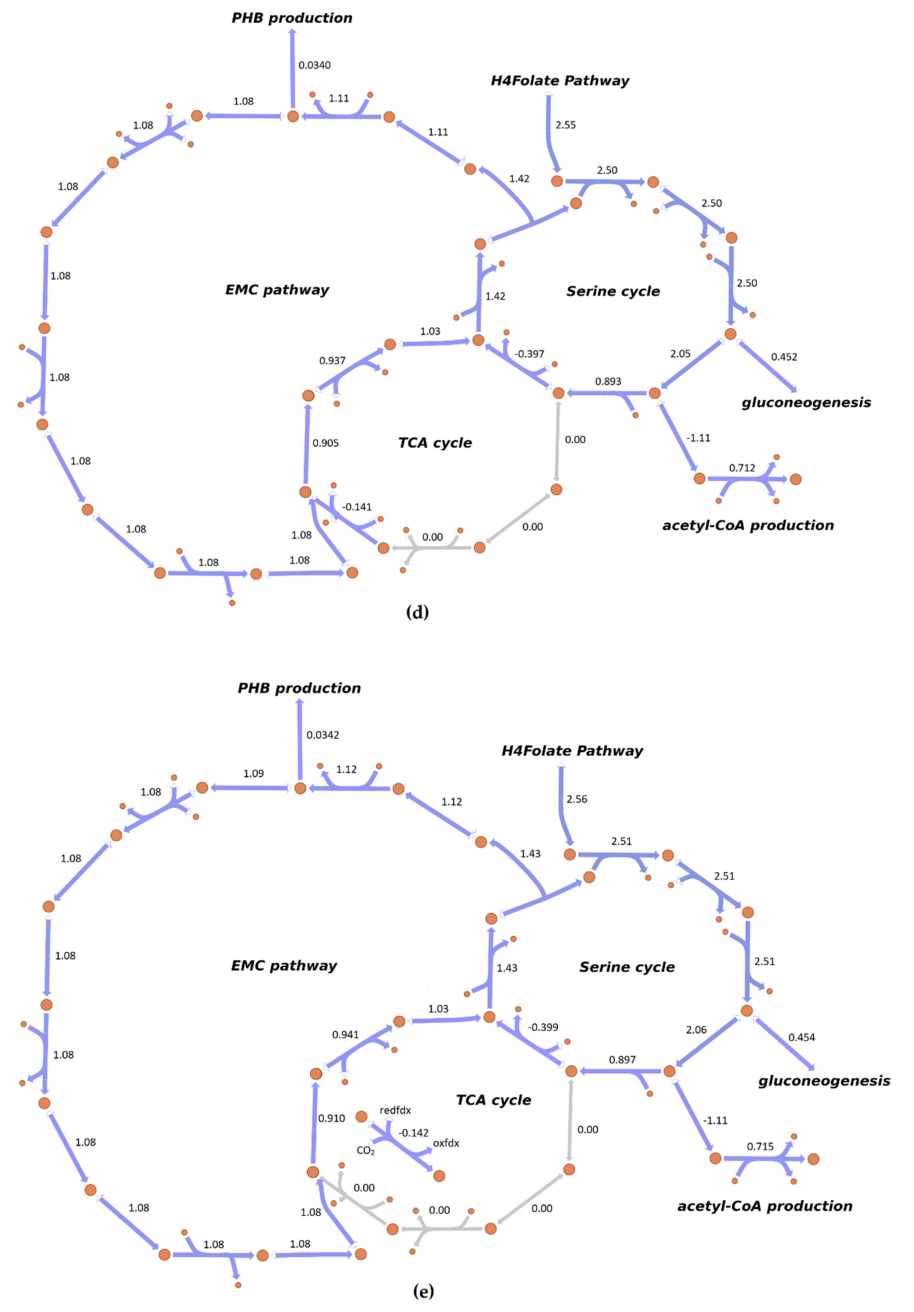

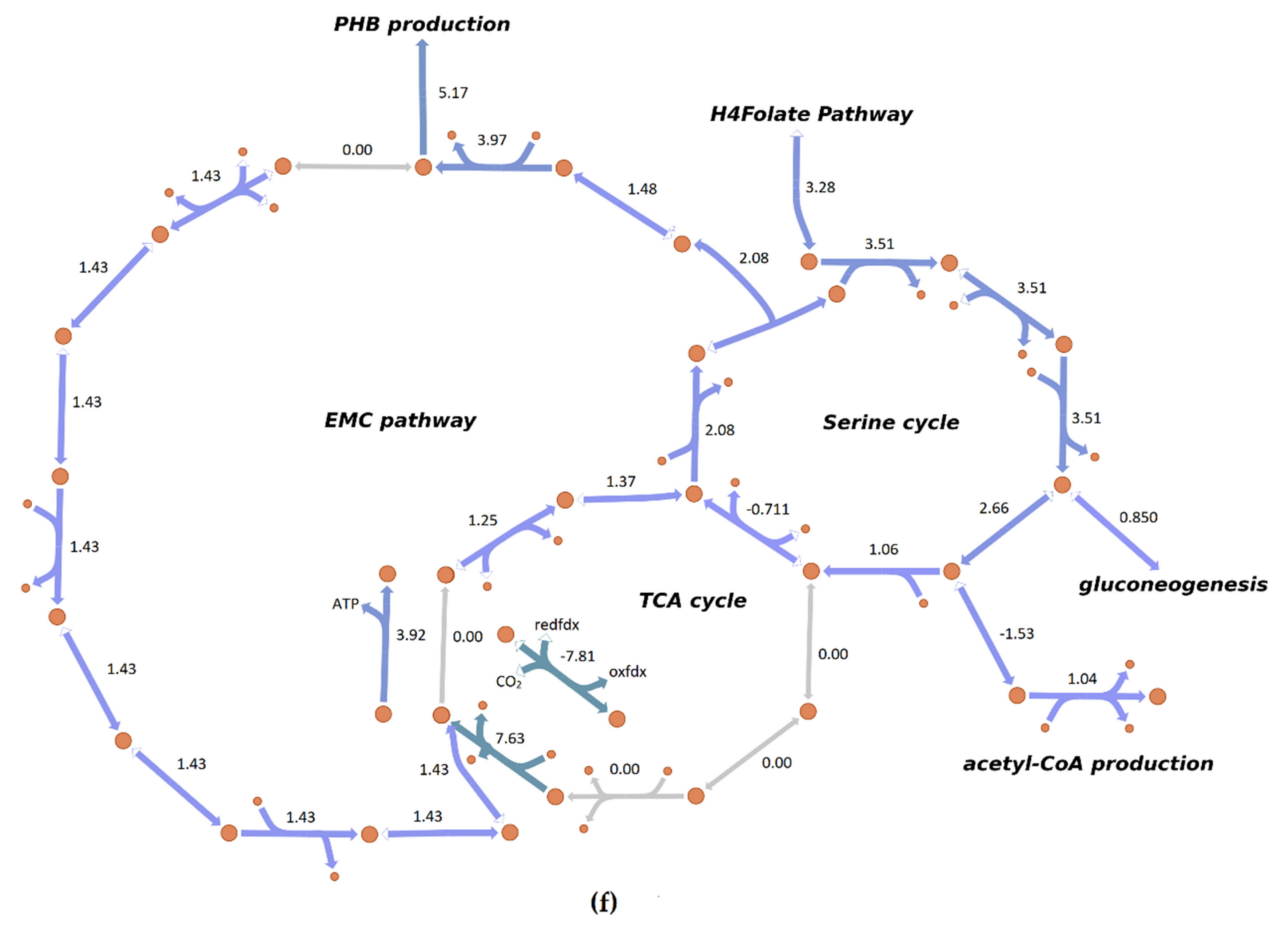
The flux distribution maps between the serine cycle, EMC pathway and TCA cycle. The maps were drawn with the help of Escher software [52]. The circles refer to metabolites, the arrows refer to reactions, and numbers refer to flux values in mmol gDCW^−1^ h^−1^. (**a**) Reference map for metabolite names, carbon and energy balance. CRN refers to crotonyl-CoA, 3HB refers to 3-hydroxybutyrate, AACOA refers to acetoacetyl-CoA, PEP refers to phosphoenolpyruvate, OAA refers to oxaloacetate, AKG refers to alpha-ketoglutarate, FUM refers to fumarate, MAL refers to malate; *S*- and *R*-MMCOA refer to *S*- and *R*-methylmalonyl-CoA respectively; and PPCOA refers to propionyl-CoA. (**b**) Unconstrained flux distribution map. No acetyl-CoA production flux via pyruvate is recorded. (**c**) Flux distribution when 1/3 of acetyl-CoA is forced to be produced from pyruvate. Figure 5D,E,F refers to reversible TCA flux distribution maps: (**d**) 2-oxoglutarate is allowed to be produced from succinyl-CoA via reversible 2-oxoglutarate dehydrogenase reaction; (**e**) 2-oxoglutarate is allowed to be produced from succinyl-CoA via forward 2-oxoacid ferredoxin synthase reaction only; (**f**) both irreversible 2-oxoglutarate reaction and 2-oxoacid ferredoxin synthase reactions are allowed to carry flux.

Both serine cycle and the EMC pathway share reactions with tricarboxylic acid cycle (TCA) cycle, thus, forming a series of tightly connected metabolic cycles (Figure 5a). The serine cycle shares malate dehydrogenase with the TCA cycle, and malyl-CoA synthetase and malyl-CoA lyase share reactions with the EMC pathway. The EMC pathway, on the other hand, shares succinate dehydrogenase and fumarase reactions with TCA cycle. In addition to the reactions describe above, the EMC pathway also shares reactions with polyhydroxybutyrate synthesis (acetyl-CoA C-acetyltransferase, acetoacetyl-CoA reductase), a compound with a significant commercial interest. 

The C2 (pyruvate, phosphoenolpyruvate and 2-phosphoglycerate) and C4 intermediates (oxaloacetate, malate) seem to play a critical role in the network organization as they are crucial in maintaining the carbon flux between the three major pathways described above. To further study the interplay between these intermediates and flux distributions in central carbon metabolisms in general, several additional constraints have been applied on the model based on the available transcriptomics and metabolomics data [36,37]. 

^13^C labelling studies have shown that at least 1/3 of acetyl-CoA comes from pyruvate, most likely via pyruvate dehydrogenase (model reaction ID: PDH). The unconstrained network selected malyl-CoA produced via malyl-CoA lyase as the main source of acetyl-CoA, indicating that the serine cycle can replenish the acetyl-CoA pool completely (Figure 5b). After the model was constrained to produce 1/3 of acetyl-CoA via pyruvate dehydrogenase, the flux towards the acetyl-CoA and EMC cycles increased (Figure 5c). This is expected as the EMC cycle strongly depends on the acetyl-CoA pool, which was increased via forced constraint. In case of the serine cycle, the flux towards phosphoenolpyruvate (PEP) slightly increased, while the flux from PEP significantly decreased. This happened because PEP acts a precursor for pyruvate. The imposed constraint drained flux from the serine cycle for acetyl-CoA production, thus, leaving phosphoenolpyruvate carboxylase and subsequent serine cycle reactions with decreased flux. The flux toward a lower part of TCA (citrate synthase, aconitate hydratase and isocitrate dehydrogenase) was not changed, indicating no effect of the constraint on it. The change in the source of acetyl-CoA did not have any effect on the predicted in silico growth rate too. One interesting aspect of the given flux distribution is that the model seems to possess an incomplete TCA cycle with no flux that goes via 2-oxoglutarate dehydrogenase. This suggests that the pool of succinyl-CoA and downstream metabolites (succinate, fumarate) is replenished primarily via the EMC pathway. This seems to be at odds with previous enzymatic studies which suggested there was a complete TCA cycle in type II methanotrophs [67]. Another interesting aspect is the fact that the source of malate comes primarily via the EMC pathway too, as the flux via malate dehydrogenase is much lower than those of fumarase (0.362 mmol gDCW^−1^ h^−1^ vs. 1.2 mmol gDCW^−1^ h^−1^). This seems to agree with ^13^C labelling data that has shown that fumarate is the most likely precursor of malate [37]. 

It has been suggested that *M. trichosporium* OB3b may possess partially reversible TCA. ^13^C labelling data have shown that 2-oxoglutarate may be produced from succinyl-CoA via putative 2-oxoacid ferredoxin reductase based on the most recent genome annotation available at the time of study [37]. Since then, the genome of *M. trichosporium* OB3b has been updated and 2-oxoacid ferredoxin reductase has been removed from genome annotations [32]. The updated genome of *M. trichosporium* OB3b contains 2-oxoglutarate dehydrogenase that may produce 2-oxoglutarate from succinyl-CoA. To check the feasibility of reversible TCA, three different enzyme combinations have been tried: (1) 2-oxoglutarate dehydrogenase (model ID reaction: AKGDH) has been allowed to proceed in the direction of 2-oxoglutarate production (Figure 5d); (2) 2-oxoacid ferredoxin synthase (model ID reaction: OOR3r) has been added to the model while blocking 2-oxoglutarate reaction (Figure 5e); (3) both 2-oxoglutarate reaction and adapted 2-oxoacid ferredoxin reaction were allowed to carry flux (Figure 5f). 

For the first and the second condition, the model confirmed predictions that succinyl-CoA can be the precursor for oxoglutarate as there was flux through the corresponding reactions. Nevertheless, the lower TCA (citrate synthase, aconitate hydratase and isocitrate dehydrogenase) stops carrying flux at all, leading to an incomplete TCA cycle. This unexpected flux distribution seems to be at odds with the ^13^C labelling data, which strongly suggested flux via lower TCA [37]. The third enzyme combination has led to the significant amount of flux in opposite directions via 2-oxoglutarate dehydrogenase and 2-oxoglutarate synthase. The model’s specific growth rate (0.155 h^−1^ vs. 0.123 h^−1^ model’s initial specific growth rate) and the flux via all 3 major pathways increased significantly. Despite this increase in flux distribution, there was still no flux via lower TCA. It seems like the third enzyme combination is unlikely to occur as the predicted specific growth rate does not match experimental values. Overall, these simulation results suggest that lower TCA plays a crucial role in replenishing oxoglutarate pool in the model while activating oxoglutarate production via another route removes the need of lower TCA to keep the flux via a biomass equation. 

Batch culture supplementation of carbon dioxide has been shown to have positive effect on the growth of *M. trichosporium* OB3b [37,43,68]. In particular, the shortening of lag-phase and an increase in the specific growth rate has been reported. To check whether an increased uptake of carbon dioxide would increase in silico specific growth rate predicted by the model, a demand reaction which supplies additional cytoplasmic carbon dioxide (model reaction ID: DM_co2_c) has been added to the model. CO_2_ supplementation was implemented by forcing a flux via carbon dioxide demand reaction. Nevertheless, this additional CO_2_ has not been assimilated in the biomass leaving the system via a carbon dioxide exchange reaction (model reaction ID: EX_co2_e) with the extracellular environment (Supplementary File: Apply omics information to the model.ipynb). The reduced costs analysis has also shown that none of the carboxylases (2-oxoglutarate dehydrogenase, crotonyl-CoA reductase-carboxylase, phosphoenolpyruvate carboxylase) involved in the central carbon metabolism reactions can increase specific growth rate (Supplementary File: Reduced_costs_redox_arm_falda_knockout.csv). The discrepancy of results with experimental data may indicate gaps in the biochemical network such as the lack of necessary carboxylase systems or pathways which can increase CO_2_ uptake by the model. 

### 3.5. Final Model Statistics

The final draft of iMsOB3b model contains 1043 reactions, 1020 metabolites and 683 genes. Of these, up to 735 reactions are used to produce 775 intracellular metabolites that are spanned over three compartments (extracellular, periplasmic and cytoplasmic). The number of reactions that are catalyzed by enzyme complexes is 125. The number of reactions without assigned genes is 227. The extended model statistics is provided in the memote report, a state-of-the-art-software used to assess the quality of genome-scale models (Supplementary File: MemoteReportApp.html) [69]. 

### 3.6. Future Research Directions

iMsOB3b represents the first, manually curated GEM for *M. trichosporium* OB3b. Nevertheless, both the model validation and its predictive behavior can be improved in many ways. 

To improve model validation, continuous culture cultivation under different combinations of nitrogen and copper-supplied mediums will provide more reliable experimental data such as flux balance analysis and other in silico approaches used to validate models assume steady-state bacterial growth. 

The improvement in the predictive behavior of the model also requires additional experimental data. The availability of the specific biomass composition would lead to the inclusion of new metabolites in the biomass equation, for which novel biosynthetic pathways will be included in the model. The improvement in the genome and proteome annotations would decrease both the number of reactions without assigned genes and reactions which were assigned based on erroneous gene annotations. The availability of global non-targeted metabolomics data may be used to improve flux distribution in the model by comparing differences in experimental metabolite concentrations in different environmental conditions with the predicted flux distributions [65]. Finally, since the model is hosted on the publicly available Github platform, any comments in the form of issues, comments, and pull requests are welcomed. 

Future applications of the model include use for metabolic engineering purposes for the increased production of metabolites of interest, as has been previously demonstrated using genome-scale models for type I methanotrophs [70,71]. In addition, since the methanotrophs, and *M. trichosporium OB3b* in particular, play a significant role in carbon cycle, the model can be used along with other models for ecosystem modelling [72]. 

## Figures and Tables

**Table 1:** In silico uptake rates, growth predictions and molar ratios for iMsOB3b in different environmental conditions. URF refers uptake rate flux. pMMO is active in copper-added medium while sMMO is active in copper-free medium. Growth rates are in h^−1^ units, flux values are in mmol gDCW^−1^ h^−1^ units.

	NO_3_^−^, pMMO	NO_3_^−^, sMMO	NH_4_^+^, pMMO	NH_4_^+^, sMMO	N_2_, pMMO	N_2_, sMMO
Methane URF	−14.9	−14.9	−14.9	−14.9	−0.88	−1.17
Oxygen URF	−22.36	−23.16	−22.49	−24.43	−1.75	−2.33
CO_2_ URF	9.97	10.50	7.75	9.65	0.875	1.17
NH_4_ URF	0	0	−1.74	−1.28	0	0
N_2_ URF	0	0	0	0	0	0
NO_3_ URF	−1.20	−1.07	0	0	0	0
Uphill Electron Transfer Flux	0	0	0	0	0	0
Growth Rate	0.123	0.109	0.178	0.131	0	0
Growth Yield ^1^	0.51	0.46	0.74	0.55	0	0
O_2_/CH_4_ Ratio	1.50	1.55	1.51	1.64	2	2
NH_4_/CH_4_ Ratio	0	0	0.12	0.086	0	0
N_2_/CH_4_ Ratio	0	0	0	0	0	0
NO_3_/CH_4_ Ratio	0.08	0.07	0	0	0	0
ATP/CH_4_ Ratio	1.43	1.24	1.87	1.44	4	3

^1^ The growth yield was calculated using this formula: $growth\;yield=\frac{\mathrm{growth}\;\mathrm{rate}}{\left|\left(\frac{\mathrm{Methane}\;\mathrm{URF}}{1000}\times 16.04\;g/\mathrm{mol}\right)\right|}$.

## Supplementary Materials

The following are available online at https://www.mdpi.com/2076-2607/8/3/437/s1. The program code used for model validation and reconstruction as well as other supplementary materials are available online at https://github.com/ensakz/gem_methylosinus_trichosporium.
