# Supplementary material for: Genome-Scale Metabolic Model Reconstruction and in Silico Investigations of Methane Metabolism in Methylosinus trichosporium OB3b
Source: Microorganisms. 2020 Mar 20;8(3):437. doi: 10.3390/microorganisms8030437 (PMC7144005; doi:10.3390/microorganisms8030437)
Supplement: Supplementary file 1 [file microorganisms-08-00437-s001.zip › supplementary_materials/gem_reconstruction_supplementary_materials/Inparanoid_outputs/AM1_GEM_locus_tags_vs_OB3b_locus_tags/orthologs.AM1_locus_tags.txt-OB3b_locus_tags.txt.html]

```
###################################
1937 groups of orthologs
2118 in-paralogs from AM1_locus_tags.txt
2068 in-paralogs from OB3b_locus_tags.txt
Grey zone 0 bits
Score cutoff 40 bits
In-paralogs with confidence less than 0.05 not shown
Sequence overlap cutoff 0.5
Group merging cutoff 0.5
Scoring matrix BLOSUM62
###################################
```

---

### Group of orthologs #1. Best score 2288 bits Score difference with first non-orthologous sequence - AM1\_locus\_tags.txt:2288 OB3b\_locus\_tags.txt:2288

```
META1_3477          	100.00%		CQW49_RS02665       	100.00%
Bootstrap support for META1_3477 as seed ortholog is 100%.
Bootstrap support for CQW49_RS02665 as seed ortholog is 100%.
```

---

### Group of orthologs #2. Best score 2273 bits Score difference with first non-orthologous sequence - AM1\_locus\_tags.txt:2273 OB3b\_locus\_tags.txt:2273

```
META1_4405          	100.00%		CQW49_RS17320       	100.00%
Bootstrap support for META1_4405 as seed ortholog is 100%.
Bootstrap support for CQW49_RS17320 as seed ortholog is 100%.
```

---

### Group of orthologs #3. Best score 2226 bits Score difference with first non-orthologous sequence - AM1\_locus\_tags.txt:2226 OB3b\_locus\_tags.txt:2226

```
META1_4406          	100.00%		CQW49_RS17330       	100.00%
Bootstrap support for META1_4406 as seed ortholog is 100%.
Bootstrap support for CQW49_RS17330 as seed ortholog is 100%.
```

---

### Group of orthologs #4. Best score 1851 bits Score difference with first non-orthologous sequence - AM1\_locus\_tags.txt:481 OB3b\_locus\_tags.txt:1851

```
META1_0872          	100.00%		CQW49_RS05145       	100.00%
Bootstrap support for META1_0872 as seed ortholog is 100%.
Bootstrap support for CQW49_RS05145 as seed ortholog is 100%.
```

---

### Group of orthologs #5. Best score 1819 bits Score difference with first non-orthologous sequence - AM1\_locus\_tags.txt:1819 OB3b\_locus\_tags.txt:1819

```
META1_1494          	100.00%		CQW49_RS19115       	100.00%
Bootstrap support for META1_1494 as seed ortholog is 100%.
Bootstrap support for CQW49_RS19115 as seed ortholog is 100%.
```

---

### Group of orthologs #6. Best score 1802 bits Score difference with first non-orthologous sequence - AM1\_locus\_tags.txt:1802 OB3b\_locus\_tags.txt:1802

```
META1_0858          	100.00%		CQW49_RS10620       	100.00%
Bootstrap support for META1_0858 as seed ortholog is 100%.
Bootstrap support for CQW49_RS10620 as seed ortholog is 100%.
```

---

### Group of orthologs #7. Best score 1579 bits Score difference with first non-orthologous sequence - AM1\_locus\_tags.txt:1025 OB3b\_locus\_tags.txt:950

```
META2_0113          	100.00%		CQW49_RS14875       	100.00%
META2_0217          	53.64%		
Bootstrap support for META2_0113 as seed ortholog is 100%.
Bootstrap support for CQW49_RS14875 as seed ortholog is 100%.
```

---

### Group of orthologs #8. Best score 1574 bits Score difference with first non-orthologous sequence - AM1\_locus\_tags.txt:1574 OB3b\_locus\_tags.txt:1574

```
META1_4357          	100.00%		CQW49_RS11690       	100.00%
Bootstrap support for META1_4357 as seed ortholog is 100%.
Bootstrap support for CQW49_RS11690 as seed ortholog is 100%.
```

---

### Group of orthologs #9. Best score 1572 bits Score difference with first non-orthologous sequence - AM1\_locus\_tags.txt:897 OB3b\_locus\_tags.txt:1572

```
META1_4552          	100.00%		CQW49_RS16705       	100.00%
Bootstrap support for META1_4552 as seed ortholog is 100%.
Bootstrap support for CQW49_RS16705 as seed ortholog is 100%.
```

---

### Group of orthologs #10. Best score 1517 bits Score difference with first non-orthologous sequence - AM1\_locus\_tags.txt:1517 OB3b\_locus\_tags.txt:1517

```
META1_3644          	100.00%		CQW49_RS12670       	100.00%
Bootstrap support for META1_3644 as seed ortholog is 100%.
Bootstrap support for CQW49_RS12670 as seed ortholog is 100%.
```

---

### Group of orthologs #11. Best score 1488 bits Score difference with first non-orthologous sequence - AM1\_locus\_tags.txt:866 OB3b\_locus\_tags.txt:1488

```
META1_4267          	100.00%		CQW49_RS20705       	100.00%
Bootstrap support for META1_4267 as seed ortholog is 100%.
Bootstrap support for CQW49_RS20705 as seed ortholog is 100%.
```

---

### Group of orthologs #12. Best score 1466 bits Score difference with first non-orthologous sequence - AM1\_locus\_tags.txt:909 OB3b\_locus\_tags.txt:1466

```
META1_4848          	100.00%		CQW49_RS16665       	100.00%
                    	       		CQW49_RS16160       	36.25%
Bootstrap support for META1_4848 as seed ortholog is 100%.
Bootstrap support for CQW49_RS16665 as seed ortholog is 100%.
```

---

### Group of orthologs #13. Best score 1426 bits Score difference with first non-orthologous sequence - AM1\_locus\_tags.txt:661 OB3b\_locus\_tags.txt:688

```
META2_0174          	100.00%		CQW49_RS19930       	100.00%
META1_2655          	65.66%		
Bootstrap support for META2_0174 as seed ortholog is 100%.
Bootstrap support for CQW49_RS19930 as seed ortholog is 100%.
```

---

### Group of orthologs #14. Best score 1394 bits Score difference with first non-orthologous sequence - AM1\_locus\_tags.txt:1394 OB3b\_locus\_tags.txt:1394

```
META1_1540          	100.00%		CQW49_RS16425       	100.00%
Bootstrap support for META1_1540 as seed ortholog is 100%.
Bootstrap support for CQW49_RS16425 as seed ortholog is 100%.
```

---

### Group of orthologs #15. Best score 1386 bits Score difference with first non-orthologous sequence - AM1\_locus\_tags.txt:1386 OB3b\_locus\_tags.txt:1386

```
META1_2327          	100.00%		CQW49_RS20490       	100.00%
Bootstrap support for META1_2327 as seed ortholog is 100%.
Bootstrap support for CQW49_RS20490 as seed ortholog is 100%.
```

---

### Group of orthologs #16. Best score 1368 bits Score difference with first non-orthologous sequence - AM1\_locus\_tags.txt:1368 OB3b\_locus\_tags.txt:1368

```
META1_1393          	100.00%		CQW49_RS12450       	100.00%
Bootstrap support for META1_1393 as seed ortholog is 100%.
Bootstrap support for CQW49_RS12450 as seed ortholog is 100%.
```

---

### Group of orthologs #17. Best score 1329 bits Score difference with first non-orthologous sequence - AM1\_locus\_tags.txt:652 OB3b\_locus\_tags.txt:1051

```
META1_0816          	100.00%		CQW49_RS01715       	100.00%
Bootstrap support for META1_0816 as seed ortholog is 100%.
Bootstrap support for CQW49_RS01715 as seed ortholog is 100%.
```

---

### Group of orthologs #18. Best score 1321 bits Score difference with first non-orthologous sequence - AM1\_locus\_tags.txt:36 OB3b\_locus\_tags.txt:1321

```
META1_2349          	100.00%		CQW49_RS18940       	100.00%
Bootstrap support for META1_2349 as seed ortholog is 80%.
Bootstrap support for CQW49_RS18940 as seed ortholog is 100%.
```

---

### Group of orthologs #19. Best score 1320 bits Score difference with first non-orthologous sequence - AM1\_locus\_tags.txt:1320 OB3b\_locus\_tags.txt:1320

```
META1_2828          	100.00%		CQW49_RS13170       	100.00%
Bootstrap support for META1_2828 as seed ortholog is 100%.
Bootstrap support for CQW49_RS13170 as seed ortholog is 100%.
```

---

### Group of orthologs #20. Best score 1289 bits Score difference with first non-orthologous sequence - AM1\_locus\_tags.txt:831 OB3b\_locus\_tags.txt:128

```
META1_2487          	100.00%		CQW49_RS08380       	100.00%
Bootstrap support for META1_2487 as seed ortholog is 100%.
Bootstrap support for CQW49_RS08380 as seed ortholog is 99%.
```

---

### Group of orthologs #21. Best score 1287 bits Score difference with first non-orthologous sequence - AM1\_locus\_tags.txt:1287 OB3b\_locus\_tags.txt:1287

```
META1_5143          	100.00%		CQW49_RS08640       	100.00%
Bootstrap support for META1_5143 as seed ortholog is 100%.
Bootstrap support for CQW49_RS08640 as seed ortholog is 100%.
```

---

### Group of orthologs #22. Best score 1276 bits Score difference with first non-orthologous sequence - AM1\_locus\_tags.txt:643 OB3b\_locus\_tags.txt:1276

```
META1_1371          	100.00%		CQW49_RS16155       	100.00%
Bootstrap support for META1_1371 as seed ortholog is 100%.
Bootstrap support for CQW49_RS16155 as seed ortholog is 100%.
```

---

### Group of orthologs #23. Best score 1272 bits Score difference with first non-orthologous sequence - AM1\_locus\_tags.txt:1272 OB3b\_locus\_tags.txt:1272

```
META1_2421          	100.00%		CQW49_RS16140       	100.00%
Bootstrap support for META1_2421 as seed ortholog is 100%.
Bootstrap support for CQW49_RS16140 as seed ortholog is 100%.
```

---

### Group of orthologs #24. Best score 1269 bits Score difference with first non-orthologous sequence - AM1\_locus\_tags.txt:957 OB3b\_locus\_tags.txt:261

```
META1_5025          	100.00%		CQW49_RS22535       	100.00%
Bootstrap support for META1_5025 as seed ortholog is 100%.
Bootstrap support for CQW49_RS22535 as seed ortholog is 99%.
```

---

### Group of orthologs #25. Best score 1263 bits Score difference with first non-orthologous sequence - AM1\_locus\_tags.txt:1263 OB3b\_locus\_tags.txt:1152

```
META1_3097          	100.00%		CQW49_RS04525       	100.00%
Bootstrap support for META1_3097 as seed ortholog is 100%.
Bootstrap support for CQW49_RS04525 as seed ortholog is 100%.
```

---

### Group of orthologs #26. Best score 1262 bits Score difference with first non-orthologous sequence - AM1\_locus\_tags.txt:968 OB3b\_locus\_tags.txt:430

```
META1_4229          	100.00%		CQW49_RS11395       	100.00%
Bootstrap support for META1_4229 as seed ortholog is 100%.
Bootstrap support for CQW49_RS11395 as seed ortholog is 100%.
```

---

### Group of orthologs #27. Best score 1257 bits Score difference with first non-orthologous sequence - AM1\_locus\_tags.txt:487 OB3b\_locus\_tags.txt:1257

```
META1_5016          	100.00%		CQW49_RS02060       	100.00%
Bootstrap support for META1_5016 as seed ortholog is 100%.
Bootstrap support for CQW49_RS02060 as seed ortholog is 100%.
```

---

### Group of orthologs #28. Best score 1252 bits Score difference with first non-orthologous sequence - AM1\_locus\_tags.txt:773 OB3b\_locus\_tags.txt:1252

```
META1_5259          	100.00%		CQW49_RS20690       	100.00%
Bootstrap support for META1_5259 as seed ortholog is 100%.
Bootstrap support for CQW49_RS20690 as seed ortholog is 100%.
```

---

### Group of orthologs #29. Best score 1235 bits Score difference with first non-orthologous sequence - AM1\_locus\_tags.txt:1156 OB3b\_locus\_tags.txt:1235

```
META1_3523          	100.00%		CQW49_RS02295       	100.00%
Bootstrap support for META1_3523 as seed ortholog is 100%.
Bootstrap support for CQW49_RS02295 as seed ortholog is 100%.
```

---

### Group of orthologs #30. Best score 1231 bits Score difference with first non-orthologous sequence - AM1\_locus\_tags.txt:836 OB3b\_locus\_tags.txt:33

```
META1_2488          	100.00%		CQW49_RS08385       	100.00%
Bootstrap support for META1_2488 as seed ortholog is 100%.
Bootstrap support for CQW49_RS08385 as seed ortholog is 32%.
Alternative seed ortholog is CQW49_RS13675 (33 bits away from this cluster)
```

---

### Group of orthologs #31. Best score 1225 bits Score difference with first non-orthologous sequence - AM1\_locus\_tags.txt:957 OB3b\_locus\_tags.txt:1039

```
META1_2014          	100.00%		CQW49_RS01040       	100.00%
Bootstrap support for META1_2014 as seed ortholog is 100%.
Bootstrap support for CQW49_RS01040 as seed ortholog is 100%.
```

---

### Group of orthologs #32. Best score 1185 bits Score difference with first non-orthologous sequence - AM1\_locus\_tags.txt:1185 OB3b\_locus\_tags.txt:903

```
META1_2150          	100.00%		CQW49_RS10535       	100.00%
Bootstrap support for META1_2150 as seed ortholog is 100%.
Bootstrap support for CQW49_RS10535 as seed ortholog is 100%.
```

---

### Group of orthologs #33. Best score 1182 bits Score difference with first non-orthologous sequence - AM1\_locus\_tags.txt:1182 OB3b\_locus\_tags.txt:1182

```
META1_2718          	100.00%		CQW49_RS11135       	100.00%
Bootstrap support for META1_2718 as seed ortholog is 100%.
Bootstrap support for CQW49_RS11135 as seed ortholog is 100%.
```

---

### Group of orthologs #34. Best score 1180 bits Score difference with first non-orthologous sequence - AM1\_locus\_tags.txt:1180 OB3b\_locus\_tags.txt:1180

```
META1_1305          	100.00%		CQW49_RS04005       	100.00%
Bootstrap support for META1_1305 as seed ortholog is 100%.
Bootstrap support for CQW49_RS04005 as seed ortholog is 100%.
```

---

### Group of orthologs #35. Best score 1180 bits Score difference with first non-orthologous sequence - AM1\_locus\_tags.txt:1180 OB3b\_locus\_tags.txt:1180

```
META1_4834          	100.00%		CQW49_RS21180       	100.00%
Bootstrap support for META1_4834 as seed ortholog is 100%.
Bootstrap support for CQW49_RS21180 as seed ortholog is 100%.
```

---

### Group of orthologs #36. Best score 1163 bits Score difference with first non-orthologous sequence - AM1\_locus\_tags.txt:1163 OB3b\_locus\_tags.txt:1163

```
META1_4445          	100.00%		CQW49_RS16815       	100.00%
Bootstrap support for META1_4445 as seed ortholog is 100%.
Bootstrap support for CQW49_RS16815 as seed ortholog is 100%.
```

---

### Group of orthologs #37. Best score 1158 bits Score difference with first non-orthologous sequence - AM1\_locus\_tags.txt:381 OB3b\_locus\_tags.txt:1158

```
META1_2546          	100.00%		CQW49_RS03785       	100.00%
Bootstrap support for META1_2546 as seed ortholog is 100%.
Bootstrap support for CQW49_RS03785 as seed ortholog is 100%.
```

---

### Group of orthologs #38. Best score 1158 bits Score difference with first non-orthologous sequence - AM1\_locus\_tags.txt:1059 OB3b\_locus\_tags.txt:1158

```
META1_1470          	100.00%		CQW49_RS10965       	100.00%
Bootstrap support for META1_1470 as seed ortholog is 100%.
Bootstrap support for CQW49_RS10965 as seed ortholog is 100%.
```

---

### Group of orthologs #39. Best score 1153 bits Score difference with first non-orthologous sequence - AM1\_locus\_tags.txt:218 OB3b\_locus\_tags.txt:1153

```
META1_2436          	100.00%		CQW49_RS03165       	100.00%
Bootstrap support for META1_2436 as seed ortholog is 99%.
Bootstrap support for CQW49_RS03165 as seed ortholog is 100%.
```

---

### Group of orthologs #40. Best score 1129 bits Score difference with first non-orthologous sequence - AM1\_locus\_tags.txt:1077 OB3b\_locus\_tags.txt:676

```
META1_1732          	100.00%		CQW49_RS12265       	100.00%
Bootstrap support for META1_1732 as seed ortholog is 100%.
Bootstrap support for CQW49_RS12265 as seed ortholog is 100%.
```

---

### Group of orthologs #41. Best score 1128 bits Score difference with first non-orthologous sequence - AM1\_locus\_tags.txt:1128 OB3b\_locus\_tags.txt:1128

```
META1_4416          	100.00%		CQW49_RS21120       	100.00%
Bootstrap support for META1_4416 as seed ortholog is 100%.
Bootstrap support for CQW49_RS21120 as seed ortholog is 100%.
```

---

### Group of orthologs #42. Best score 1116 bits Score difference with first non-orthologous sequence - AM1\_locus\_tags.txt:1116 OB3b\_locus\_tags.txt:1116

```
META1_2041          	100.00%		CQW49_RS15650       	100.00%
Bootstrap support for META1_2041 as seed ortholog is 100%.
Bootstrap support for CQW49_RS15650 as seed ortholog is 100%.
```

---

### Group of orthologs #43. Best score 1096 bits Score difference with first non-orthologous sequence - AM1\_locus\_tags.txt:716 OB3b\_locus\_tags.txt:679

```
META1_4300          	100.00%		CQW49_RS11710       	100.00%
META1_0312          	45.50%		
Bootstrap support for META1_4300 as seed ortholog is 100%.
Bootstrap support for CQW49_RS11710 as seed ortholog is 100%.
```

---

### Group of orthologs #44. Best score 1088 bits Score difference with first non-orthologous sequence - AM1\_locus\_tags.txt:1088 OB3b\_locus\_tags.txt:221

```
META2_0008          	100.00%		CQW49_RS21550       	100.00%
                    	       		CQW49_RS21490       	6.75%
                    	       		CQW49_RS21485       	5.31%
Bootstrap support for META2_0008 as seed ortholog is 100%.
Bootstrap support for CQW49_RS21550 as seed ortholog is 96%.
```

---

### Group of orthologs #45. Best score 1082 bits Score difference with first non-orthologous sequence - AM1\_locus\_tags.txt:769 OB3b\_locus\_tags.txt:689

```
META1_4538          	100.00%		CQW49_RS14460       	100.00%
Bootstrap support for META1_4538 as seed ortholog is 100%.
Bootstrap support for CQW49_RS14460 as seed ortholog is 100%.
```

---

### Group of orthologs #46. Best score 1074 bits Score difference with first non-orthologous sequence - AM1\_locus\_tags.txt:172 OB3b\_locus\_tags.txt:1074

```
META1_3164          	100.00%		CQW49_RS20640       	100.00%
Bootstrap support for META1_3164 as seed ortholog is 100%.
Bootstrap support for CQW49_RS20640 as seed ortholog is 100%.
```

---

### Group of orthologs #47. Best score 1061 bits Score difference with first non-orthologous sequence - AM1\_locus\_tags.txt:1061 OB3b\_locus\_tags.txt:1061

```
META1_0010          	100.00%		CQW49_RS11955       	100.00%
Bootstrap support for META1_0010 as seed ortholog is 100%.
Bootstrap support for CQW49_RS11955 as seed ortholog is 100%.
```

---

### Group of orthologs #48. Best score 1054 bits Score difference with first non-orthologous sequence - AM1\_locus\_tags.txt:1054 OB3b\_locus\_tags.txt:1054

```
META1_1524          	100.00%		CQW49_RS15785       	100.00%
Bootstrap support for META1_1524 as seed ortholog is 100%.
Bootstrap support for CQW49_RS15785 as seed ortholog is 100%.
```

---

### Group of orthologs #49. Best score 1054 bits Score difference with first non-orthologous sequence - AM1\_locus\_tags.txt:1054 OB3b\_locus\_tags.txt:1054

```
META1_5251          	100.00%		CQW49_RS12585       	100.00%
Bootstrap support for META1_5251 as seed ortholog is 100%.
Bootstrap support for CQW49_RS12585 as seed ortholog is 100%.
```

---

### Group of orthologs #50. Best score 1042 bits Score difference with first non-orthologous sequence - AM1\_locus\_tags.txt:1042 OB3b\_locus\_tags.txt:1042

```
META1_0229          	100.00%		CQW49_RS19455       	100.00%
Bootstrap support for META1_0229 as seed ortholog is 100%.
Bootstrap support for CQW49_RS19455 as seed ortholog is 100%.
```

---

### Group of orthologs #51. Best score 1040 bits Score difference with first non-orthologous sequence - AM1\_locus\_tags.txt:700 OB3b\_locus\_tags.txt:637

```
META1_0214          	100.00%		CQW49_RS17045       	100.00%
Bootstrap support for META1_0214 as seed ortholog is 100%.
Bootstrap support for CQW49_RS17045 as seed ortholog is 100%.
```

---

### Group of orthologs #52. Best score 1037 bits Score difference with first non-orthologous sequence - AM1\_locus\_tags.txt:1037 OB3b\_locus\_tags.txt:1037

```
META1_2049          	100.00%		CQW49_RS02860       	100.00%
Bootstrap support for META1_2049 as seed ortholog is 100%.
Bootstrap support for CQW49_RS02860 as seed ortholog is 100%.
```

---

### Group of orthologs #53. Best score 1033 bits Score difference with first non-orthologous sequence - AM1\_locus\_tags.txt:1033 OB3b\_locus\_tags.txt:1033

```
META1_2054          	100.00%		CQW49_RS18690       	100.00%
Bootstrap support for META1_2054 as seed ortholog is 100%.
Bootstrap support for CQW49_RS18690 as seed ortholog is 100%.
```

---

### Group of orthologs #54. Best score 1020 bits Score difference with first non-orthologous sequence - AM1\_locus\_tags.txt:538 OB3b\_locus\_tags.txt:1020

```
META1_3919          	100.00%		CQW49_RS11815       	100.00%
Bootstrap support for META1_3919 as seed ortholog is 100%.
Bootstrap support for CQW49_RS11815 as seed ortholog is 100%.
```

---

### Group of orthologs #55. Best score 1010 bits Score difference with first non-orthologous sequence - AM1\_locus\_tags.txt:590 OB3b\_locus\_tags.txt:860

```
META2_0206          	100.00%		CQW49_RS14870       	100.00%
META1_2605          	78.13%		
META1_1889          	53.26%		
META2_0089          	43.74%		
Bootstrap support for META2_0206 as seed ortholog is 100%.
Bootstrap support for CQW49_RS14870 as seed ortholog is 100%.
```

---

### Group of orthologs #56. Best score 1010 bits Score difference with first non-orthologous sequence - AM1\_locus\_tags.txt:1010 OB3b\_locus\_tags.txt:1010

```
META1_3452          	100.00%		CQW49_RS15535       	100.00%
Bootstrap support for META1_3452 as seed ortholog is 100%.
Bootstrap support for CQW49_RS15535 as seed ortholog is 100%.
```

---

### Group of orthologs #57. Best score 1007 bits Score difference with first non-orthologous sequence - AM1\_locus\_tags.txt:1007 OB3b\_locus\_tags.txt:1007

```
META1_3408          	100.00%		CQW49_RS13460       	100.00%
Bootstrap support for META1_3408 as seed ortholog is 100%.
Bootstrap support for CQW49_RS13460 as seed ortholog is 100%.
```

---

### Group of orthologs #58. Best score 1006 bits Score difference with first non-orthologous sequence - AM1\_locus\_tags.txt:1006 OB3b\_locus\_tags.txt:1006

```
META1_5061          	100.00%		CQW49_RS03945       	100.00%
Bootstrap support for META1_5061 as seed ortholog is 100%.
Bootstrap support for CQW49_RS03945 as seed ortholog is 100%.
```

---

### Group of orthologs #59. Best score 1005 bits Score difference with first non-orthologous sequence - AM1\_locus\_tags.txt:1005 OB3b\_locus\_tags.txt:1005

```
META1_0361          	100.00%		CQW49_RS19515       	100.00%
Bootstrap support for META1_0361 as seed ortholog is 100%.
Bootstrap support for CQW49_RS19515 as seed ortholog is 100%.
```

---

### Group of orthologs #60. Best score 1003 bits Score difference with first non-orthologous sequence - AM1\_locus\_tags.txt:1003 OB3b\_locus\_tags.txt:1003

```
META1_2890          	100.00%		CQW49_RS11185       	100.00%
Bootstrap support for META1_2890 as seed ortholog is 100%.
Bootstrap support for CQW49_RS11185 as seed ortholog is 100%.
```

---

### Group of orthologs #61. Best score 997 bits Score difference with first non-orthologous sequence - AM1\_locus\_tags.txt:997 OB3b\_locus\_tags.txt:997

```
META1_1025          	100.00%		CQW49_RS04230       	100.00%
Bootstrap support for META1_1025 as seed ortholog is 100%.
Bootstrap support for CQW49_RS04230 as seed ortholog is 100%.
```

---

### Group of orthologs #62. Best score 988 bits Score difference with first non-orthologous sequence - AM1\_locus\_tags.txt:612 OB3b\_locus\_tags.txt:988

```
META1_2868          	100.00%		CQW49_RS03915       	100.00%
Bootstrap support for META1_2868 as seed ortholog is 100%.
Bootstrap support for CQW49_RS03915 as seed ortholog is 100%.
```

---

### Group of orthologs #63. Best score 987 bits Score difference with first non-orthologous sequence - AM1\_locus\_tags.txt:987 OB3b\_locus\_tags.txt:987

```
META1_0211          	100.00%		CQW49_RS13000       	100.00%
Bootstrap support for META1_0211 as seed ortholog is 100%.
Bootstrap support for CQW49_RS13000 as seed ortholog is 100%.
```

---

### Group of orthologs #64. Best score 986 bits Score difference with first non-orthologous sequence - AM1\_locus\_tags.txt:986 OB3b\_locus\_tags.txt:986

```
META1_1050          	100.00%		CQW49_RS13050       	100.00%
Bootstrap support for META1_1050 as seed ortholog is 100%.
Bootstrap support for CQW49_RS13050 as seed ortholog is 100%.
```

---

### Group of orthologs #65. Best score 985 bits Score difference with first non-orthologous sequence - AM1\_locus\_tags.txt:985 OB3b\_locus\_tags.txt:468

```
META1_1821          	100.00%		CQW49_RS23875       	100.00%
Bootstrap support for META1_1821 as seed ortholog is 100%.
Bootstrap support for CQW49_RS23875 as seed ortholog is 100%.
```

---

### Group of orthologs #66. Best score 980 bits Score difference with first non-orthologous sequence - AM1\_locus\_tags.txt:894 OB3b\_locus\_tags.txt:980

```
META1_3861          	100.00%		CQW49_RS10840       	100.00%
Bootstrap support for META1_3861 as seed ortholog is 100%.
Bootstrap support for CQW49_RS10840 as seed ortholog is 100%.
```

---

### Group of orthologs #67. Best score 977 bits Score difference with first non-orthologous sequence - AM1\_locus\_tags.txt:977 OB3b\_locus\_tags.txt:977

```
META1_3608          	100.00%		CQW49_RS21455       	100.00%
Bootstrap support for META1_3608 as seed ortholog is 100%.
Bootstrap support for CQW49_RS21455 as seed ortholog is 100%.
```

---

### Group of orthologs #68. Best score 971 bits Score difference with first non-orthologous sequence - AM1\_locus\_tags.txt:971 OB3b\_locus\_tags.txt:971

```
META1_0578          	100.00%		CQW49_RS21200       	100.00%
Bootstrap support for META1_0578 as seed ortholog is 100%.
Bootstrap support for CQW49_RS21200 as seed ortholog is 100%.
```

---

### Group of orthologs #69. Best score 968 bits Score difference with first non-orthologous sequence - AM1\_locus\_tags.txt:968 OB3b\_locus\_tags.txt:968

```
META1_4680          	100.00%		CQW49_RS03200       	100.00%
Bootstrap support for META1_4680 as seed ortholog is 100%.
Bootstrap support for CQW49_RS03200 as seed ortholog is 100%.
```

---

### Group of orthologs #70. Best score 967 bits Score difference with first non-orthologous sequence - AM1\_locus\_tags.txt:601 OB3b\_locus\_tags.txt:967

```
META1_0822          	100.00%		CQW49_RS09520       	100.00%
Bootstrap support for META1_0822 as seed ortholog is 100%.
Bootstrap support for CQW49_RS09520 as seed ortholog is 100%.
```

---

### Group of orthologs #71. Best score 966 bits Score difference with first non-orthologous sequence - AM1\_locus\_tags.txt:966 OB3b\_locus\_tags.txt:884

```
META1_0731          	100.00%		CQW49_RS01035       	100.00%
Bootstrap support for META1_0731 as seed ortholog is 100%.
Bootstrap support for CQW49_RS01035 as seed ortholog is 100%.
```

---

### Group of orthologs #72. Best score 962 bits Score difference with first non-orthologous sequence - AM1\_locus\_tags.txt:539 OB3b\_locus\_tags.txt:798

```
META1_2531          	100.00%		CQW49_RS13870       	100.00%
Bootstrap support for META1_2531 as seed ortholog is 100%.
Bootstrap support for CQW49_RS13870 as seed ortholog is 100%.
```

---

### Group of orthologs #73. Best score 962 bits Score difference with first non-orthologous sequence - AM1\_locus\_tags.txt:962 OB3b\_locus\_tags.txt:962

```
META1_2963          	100.00%		CQW49_RS21015       	100.00%
Bootstrap support for META1_2963 as seed ortholog is 100%.
Bootstrap support for CQW49_RS21015 as seed ortholog is 100%.
```

---

### Group of orthologs #74. Best score 962 bits Score difference with first non-orthologous sequence - AM1\_locus\_tags.txt:962 OB3b\_locus\_tags.txt:962

```
META1_4393          	100.00%		CQW49_RS19850       	100.00%
Bootstrap support for META1_4393 as seed ortholog is 100%.
Bootstrap support for CQW49_RS19850 as seed ortholog is 100%.
```

---

### Group of orthologs #75. Best score 959 bits Score difference with first non-orthologous sequence - AM1\_locus\_tags.txt:959 OB3b\_locus\_tags.txt:959

```
META1_4678          	100.00%		CQW49_RS16775       	100.00%
Bootstrap support for META1_4678 as seed ortholog is 100%.
Bootstrap support for CQW49_RS16775 as seed ortholog is 100%.
```

---

### Group of orthologs #76. Best score 958 bits Score difference with first non-orthologous sequence - AM1\_locus\_tags.txt:471 OB3b\_locus\_tags.txt:958

```
META1_3331          	100.00%		CQW49_RS09485       	100.00%
Bootstrap support for META1_3331 as seed ortholog is 100%.
Bootstrap support for CQW49_RS09485 as seed ortholog is 100%.
```

---

### Group of orthologs #77. Best score 956 bits Score difference with first non-orthologous sequence - AM1\_locus\_tags.txt:956 OB3b\_locus\_tags.txt:956

```
META1_3299          	100.00%		CQW49_RS19550       	100.00%
Bootstrap support for META1_3299 as seed ortholog is 100%.
Bootstrap support for CQW49_RS19550 as seed ortholog is 100%.
```

---

### Group of orthologs #78. Best score 953 bits Score difference with first non-orthologous sequence - AM1\_locus\_tags.txt:495 OB3b\_locus\_tags.txt:503

```
META1_3203          	100.00%		CQW49_RS20880       	100.00%
Bootstrap support for META1_3203 as seed ortholog is 100%.
Bootstrap support for CQW49_RS20880 as seed ortholog is 100%.
```

---

### Group of orthologs #79. Best score 946 bits Score difference with first non-orthologous sequence - AM1\_locus\_tags.txt:946 OB3b\_locus\_tags.txt:946

```
META1_1503          	100.00%		CQW49_RS04945       	100.00%
Bootstrap support for META1_1503 as seed ortholog is 100%.
Bootstrap support for CQW49_RS04945 as seed ortholog is 100%.
```

---

### Group of orthologs #80. Best score 945 bits Score difference with first non-orthologous sequence - AM1\_locus\_tags.txt:945 OB3b\_locus\_tags.txt:945

```
META1_2323          	100.00%		CQW49_RS16010       	100.00%
Bootstrap support for META1_2323 as seed ortholog is 100%.
Bootstrap support for CQW49_RS16010 as seed ortholog is 100%.
```

---

### Group of orthologs #81. Best score 940 bits Score difference with first non-orthologous sequence - AM1\_locus\_tags.txt:940 OB3b\_locus\_tags.txt:940

```
META1_5299          	100.00%		CQW49_RS18575       	100.00%
                    	       		CQW49_RS18565       	75.62%
Bootstrap support for META1_5299 as seed ortholog is 100%.
Bootstrap support for CQW49_RS18575 as seed ortholog is 100%.
```

---

### Group of orthologs #82. Best score 940 bits Score difference with first non-orthologous sequence - AM1\_locus\_tags.txt:635 OB3b\_locus\_tags.txt:940

```
META1_0435          	100.00%		CQW49_RS04780       	100.00%
Bootstrap support for META1_0435 as seed ortholog is 100%.
Bootstrap support for CQW49_RS04780 as seed ortholog is 100%.
```

---

### Group of orthologs #83. Best score 936 bits Score difference with first non-orthologous sequence - AM1\_locus\_tags.txt:177 OB3b\_locus\_tags.txt:936

```
META1_2090          	100.00%		CQW49_RS04520       	100.00%
Bootstrap support for META1_2090 as seed ortholog is 99%.
Bootstrap support for CQW49_RS04520 as seed ortholog is 100%.
```

---

### Group of orthologs #84. Best score 929 bits Score difference with first non-orthologous sequence - AM1\_locus\_tags.txt:163 OB3b\_locus\_tags.txt:479

```
META1_3542          	100.00%		CQW49_RS21995       	100.00%
META1_2762          	30.85%		
Bootstrap support for META1_3542 as seed ortholog is 99%.
Bootstrap support for CQW49_RS21995 as seed ortholog is 100%.
```

---

### Group of orthologs #85. Best score 929 bits Score difference with first non-orthologous sequence - AM1\_locus\_tags.txt:363 OB3b\_locus\_tags.txt:929

```
META1_0515          	100.00%		CQW49_RS19770       	100.00%
Bootstrap support for META1_0515 as seed ortholog is 100%.
Bootstrap support for CQW49_RS19770 as seed ortholog is 100%.
```

---

### Group of orthologs #86. Best score 925 bits Score difference with first non-orthologous sequence - AM1\_locus\_tags.txt:547 OB3b\_locus\_tags.txt:477

```
META1_1740          	100.00%		CQW49_RS14430       	100.00%
META1_2757          	56.67%		
Bootstrap support for META1_1740 as seed ortholog is 100%.
Bootstrap support for CQW49_RS14430 as seed ortholog is 100%.
```

---

### Group of orthologs #87. Best score 925 bits Score difference with first non-orthologous sequence - AM1\_locus\_tags.txt:925 OB3b\_locus\_tags.txt:925

```
META1_0844          	100.00%		CQW49_RS05300       	100.00%
Bootstrap support for META1_0844 as seed ortholog is 100%.
Bootstrap support for CQW49_RS05300 as seed ortholog is 100%.
```

---

### Group of orthologs #88. Best score 925 bits Score difference with first non-orthologous sequence - AM1\_locus\_tags.txt:871 OB3b\_locus\_tags.txt:925

```
META1_1809          	100.00%		CQW49_RS06480       	100.00%
Bootstrap support for META1_1809 as seed ortholog is 100%.
Bootstrap support for CQW49_RS06480 as seed ortholog is 100%.
```

---

### Group of orthologs #89. Best score 923 bits Score difference with first non-orthologous sequence - AM1\_locus\_tags.txt:738 OB3b\_locus\_tags.txt:923

```
META1_5321          	100.00%		CQW49_RS06830       	100.00%
Bootstrap support for META1_5321 as seed ortholog is 100%.
Bootstrap support for CQW49_RS06830 as seed ortholog is 100%.
```

---

### Group of orthologs #90. Best score 922 bits Score difference with first non-orthologous sequence - AM1\_locus\_tags.txt:922 OB3b\_locus\_tags.txt:922

```
META1_3916          	100.00%		CQW49_RS13135       	100.00%
Bootstrap support for META1_3916 as seed ortholog is 100%.
Bootstrap support for CQW49_RS13135 as seed ortholog is 100%.
```

---

### Group of orthologs #91. Best score 921 bits Score difference with first non-orthologous sequence - AM1\_locus\_tags.txt:311 OB3b\_locus\_tags.txt:809

```
p2METAp0038         	100.00%		CQW49_RS08930       	100.00%
Bootstrap support for p2METAp0038 as seed ortholog is 100%.
Bootstrap support for CQW49_RS08930 as seed ortholog is 100%.
```

---

### Group of orthologs #92. Best score 914 bits Score difference with first non-orthologous sequence - AM1\_locus\_tags.txt:782 OB3b\_locus\_tags.txt:914

```
META1_0849          	100.00%		CQW49_RS05325       	100.00%
Bootstrap support for META1_0849 as seed ortholog is 100%.
Bootstrap support for CQW49_RS05325 as seed ortholog is 100%.
```

---

### Group of orthologs #93. Best score 912 bits Score difference with first non-orthologous sequence - AM1\_locus\_tags.txt:737 OB3b\_locus\_tags.txt:749

```
META1_0130          	100.00%		CQW49_RS10655       	100.00%
Bootstrap support for META1_0130 as seed ortholog is 100%.
Bootstrap support for CQW49_RS10655 as seed ortholog is 100%.
```

---

### Group of orthologs #94. Best score 910 bits Score difference with first non-orthologous sequence - AM1\_locus\_tags.txt:910 OB3b\_locus\_tags.txt:910

```
META1_0055          	100.00%		CQW49_RS16360       	100.00%
Bootstrap support for META1_0055 as seed ortholog is 100%.
Bootstrap support for CQW49_RS16360 as seed ortholog is 100%.
```

---

### Group of orthologs #95. Best score 908 bits Score difference with first non-orthologous sequence - AM1\_locus\_tags.txt:908 OB3b\_locus\_tags.txt:908

```
META1_1996          	100.00%		CQW49_RS19130       	100.00%
Bootstrap support for META1_1996 as seed ortholog is 100%.
Bootstrap support for CQW49_RS19130 as seed ortholog is 100%.
```

---

### Group of orthologs #96. Best score 907 bits Score difference with first non-orthologous sequence - AM1\_locus\_tags.txt:231 OB3b\_locus\_tags.txt:907

```
META1_3224          	100.00%		CQW49_RS21400       	100.00%
Bootstrap support for META1_3224 as seed ortholog is 100%.
Bootstrap support for CQW49_RS21400 as seed ortholog is 100%.
```

---

### Group of orthologs #97. Best score 892 bits Score difference with first non-orthologous sequence - AM1\_locus\_tags.txt:892 OB3b\_locus\_tags.txt:892

```
META1_1813          	100.00%		CQW49_RS09240       	100.00%
META1_4706          	90.80%		
Bootstrap support for META1_1813 as seed ortholog is 100%.
Bootstrap support for CQW49_RS09240 as seed ortholog is 100%.
```

---

### Group of orthologs #98. Best score 882 bits Score difference with first non-orthologous sequence - AM1\_locus\_tags.txt:882 OB3b\_locus\_tags.txt:882

```
META1_1178          	100.00%		CQW49_RS05135       	100.00%
Bootstrap support for META1_1178 as seed ortholog is 100%.
Bootstrap support for CQW49_RS05135 as seed ortholog is 100%.
```

---

### Group of orthologs #99. Best score 877 bits Score difference with first non-orthologous sequence - AM1\_locus\_tags.txt:877 OB3b\_locus\_tags.txt:877

```
META1_2066          	100.00%		CQW49_RS00465       	100.00%
Bootstrap support for META1_2066 as seed ortholog is 100%.
Bootstrap support for CQW49_RS00465 as seed ortholog is 100%.
```

---

### Group of orthologs #100. Best score 876 bits Score difference with first non-orthologous sequence - AM1\_locus\_tags.txt:876 OB3b\_locus\_tags.txt:876

```
META1_0180          	100.00%		CQW49_RS20220       	100.00%
Bootstrap support for META1_0180 as seed ortholog is 100%.
Bootstrap support for CQW49_RS20220 as seed ortholog is 100%.
```

---

### Group of orthologs #101. Best score 873 bits Score difference with first non-orthologous sequence - AM1\_locus\_tags.txt:873 OB3b\_locus\_tags.txt:873

```
META1_0083          	100.00%		CQW49_RS13415       	100.00%
Bootstrap support for META1_0083 as seed ortholog is 100%.
Bootstrap support for CQW49_RS13415 as seed ortholog is 100%.
```

---

### Group of orthologs #102. Best score 871 bits Score difference with first non-orthologous sequence - AM1\_locus\_tags.txt:595 OB3b\_locus\_tags.txt:650

```
META1_2722          	100.00%		CQW49_RS01470       	100.00%
Bootstrap support for META1_2722 as seed ortholog is 100%.
Bootstrap support for CQW49_RS01470 as seed ortholog is 100%.
```

---

### Group of orthologs #103. Best score 867 bits Score difference with first non-orthologous sequence - AM1\_locus\_tags.txt:867 OB3b\_locus\_tags.txt:867

```
META1_2951          	100.00%		CQW49_RS06035       	100.00%
Bootstrap support for META1_2951 as seed ortholog is 100%.
Bootstrap support for CQW49_RS06035 as seed ortholog is 100%.
```

---

### Group of orthologs #104. Best score 865 bits Score difference with first non-orthologous sequence - AM1\_locus\_tags.txt:865 OB3b\_locus\_tags.txt:865

```
META1_1818          	100.00%		CQW49_RS17155       	100.00%
Bootstrap support for META1_1818 as seed ortholog is 100%.
Bootstrap support for CQW49_RS17155 as seed ortholog is 100%.
```

---

### Group of orthologs #105. Best score 863 bits Score difference with first non-orthologous sequence - AM1\_locus\_tags.txt:863 OB3b\_locus\_tags.txt:863

```
META1_4550          	100.00%		CQW49_RS01535       	100.00%
Bootstrap support for META1_4550 as seed ortholog is 100%.
Bootstrap support for CQW49_RS01535 as seed ortholog is 100%.
```

---

### Group of orthologs #106. Best score 859 bits Score difference with first non-orthologous sequence - AM1\_locus\_tags.txt:375 OB3b\_locus\_tags.txt:699

```
META1_2883          	100.00%		CQW49_RS04965       	100.00%
Bootstrap support for META1_2883 as seed ortholog is 100%.
Bootstrap support for CQW49_RS04965 as seed ortholog is 100%.
```

---

### Group of orthologs #107. Best score 857 bits Score difference with first non-orthologous sequence - AM1\_locus\_tags.txt:857 OB3b\_locus\_tags.txt:596

```
META1_0172          	100.00%		CQW49_RS16120       	100.00%
Bootstrap support for META1_0172 as seed ortholog is 100%.
Bootstrap support for CQW49_RS16120 as seed ortholog is 100%.
```

---

### Group of orthologs #108. Best score 856 bits Score difference with first non-orthologous sequence - AM1\_locus\_tags.txt:856 OB3b\_locus\_tags.txt:856

```
META1_3083          	100.00%		CQW49_RS03700       	100.00%
Bootstrap support for META1_3083 as seed ortholog is 100%.
Bootstrap support for CQW49_RS03700 as seed ortholog is 100%.
```

---

### Group of orthologs #109. Best score 852 bits Score difference with first non-orthologous sequence - AM1\_locus\_tags.txt:729 OB3b\_locus\_tags.txt:436

```
META1_1361          	100.00%		CQW49_RS00295       	100.00%
Bootstrap support for META1_1361 as seed ortholog is 100%.
Bootstrap support for CQW49_RS00295 as seed ortholog is 100%.
```

---

### Group of orthologs #110. Best score 851 bits Score difference with first non-orthologous sequence - AM1\_locus\_tags.txt:504 OB3b\_locus\_tags.txt:535

```
META1_3933          	100.00%		CQW49_RS09135       	100.00%
Bootstrap support for META1_3933 as seed ortholog is 100%.
Bootstrap support for CQW49_RS09135 as seed ortholog is 100%.
```

---

### Group of orthologs #111. Best score 851 bits Score difference with first non-orthologous sequence - AM1\_locus\_tags.txt:851 OB3b\_locus\_tags.txt:851

```
META1_5110          	100.00%		CQW49_RS19000       	100.00%
Bootstrap support for META1_5110 as seed ortholog is 100%.
Bootstrap support for CQW49_RS19000 as seed ortholog is 100%.
```

---

### Group of orthologs #112. Best score 848 bits Score difference with first non-orthologous sequence - AM1\_locus\_tags.txt:848 OB3b\_locus\_tags.txt:848

```
META1_4361          	100.00%		CQW49_RS01415       	100.00%
Bootstrap support for META1_4361 as seed ortholog is 100%.
Bootstrap support for CQW49_RS01415 as seed ortholog is 100%.
```

---

### Group of orthologs #113. Best score 844 bits Score difference with first non-orthologous sequence - AM1\_locus\_tags.txt:754 OB3b\_locus\_tags.txt:605

```
META1_2211          	100.00%		CQW49_RS17010       	100.00%
Bootstrap support for META1_2211 as seed ortholog is 100%.
Bootstrap support for CQW49_RS17010 as seed ortholog is 100%.
```

---

### Group of orthologs #114. Best score 842 bits Score difference with first non-orthologous sequence - AM1\_locus\_tags.txt:842 OB3b\_locus\_tags.txt:842

```
META1_2382          	100.00%		CQW49_RS02305       	100.00%
Bootstrap support for META1_2382 as seed ortholog is 100%.
Bootstrap support for CQW49_RS02305 as seed ortholog is 100%.
```

---

### Group of orthologs #115. Best score 842 bits Score difference with first non-orthologous sequence - AM1\_locus\_tags.txt:842 OB3b\_locus\_tags.txt:842

```
META1_3689          	100.00%		CQW49_RS01770       	100.00%
Bootstrap support for META1_3689 as seed ortholog is 100%.
Bootstrap support for CQW49_RS01770 as seed ortholog is 100%.
```

---

### Group of orthologs #116. Best score 842 bits Score difference with first non-orthologous sequence - AM1\_locus\_tags.txt:409 OB3b\_locus\_tags.txt:842

```
META1_1328          	100.00%		CQW49_RS17905       	100.00%
Bootstrap support for META1_1328 as seed ortholog is 100%.
Bootstrap support for CQW49_RS17905 as seed ortholog is 100%.
```

---

### Group of orthologs #117. Best score 842 bits Score difference with first non-orthologous sequence - AM1\_locus\_tags.txt:842 OB3b\_locus\_tags.txt:842

```
META1_1349          	100.00%		CQW49_RS20965       	100.00%
Bootstrap support for META1_1349 as seed ortholog is 100%.
Bootstrap support for CQW49_RS20965 as seed ortholog is 100%.
```

---

### Group of orthologs #118. Best score 828 bits Score difference with first non-orthologous sequence - AM1\_locus\_tags.txt:583 OB3b\_locus\_tags.txt:555

```
META1_4736          	100.00%		CQW49_RS04935       	100.00%
Bootstrap support for META1_4736 as seed ortholog is 100%.
Bootstrap support for CQW49_RS04935 as seed ortholog is 100%.
```

---

### Group of orthologs #119. Best score 828 bits Score difference with first non-orthologous sequence - AM1\_locus\_tags.txt:828 OB3b\_locus\_tags.txt:828

```
META1_3135          	100.00%		CQW49_RS20850       	100.00%
Bootstrap support for META1_3135 as seed ortholog is 100%.
Bootstrap support for CQW49_RS20850 as seed ortholog is 100%.
```

---

### Group of orthologs #120. Best score 824 bits Score difference with first non-orthologous sequence - AM1\_locus\_tags.txt:729 OB3b\_locus\_tags.txt:824

```
META1_1474          	100.00%		CQW49_RS12800       	100.00%
Bootstrap support for META1_1474 as seed ortholog is 100%.
Bootstrap support for CQW49_RS12800 as seed ortholog is 100%.
```

---

### Group of orthologs #121. Best score 823 bits Score difference with first non-orthologous sequence - AM1\_locus\_tags.txt:710 OB3b\_locus\_tags.txt:729

```
META1_3456          	100.00%		CQW49_RS15525       	100.00%
Bootstrap support for META1_3456 as seed ortholog is 100%.
Bootstrap support for CQW49_RS15525 as seed ortholog is 100%.
```

---

### Group of orthologs #122. Best score 817 bits Score difference with first non-orthologous sequence - AM1\_locus\_tags.txt:817 OB3b\_locus\_tags.txt:743

```
META1_4825          	100.00%		CQW49_RS06840       	100.00%
Bootstrap support for META1_4825 as seed ortholog is 100%.
Bootstrap support for CQW49_RS06840 as seed ortholog is 100%.
```

---

### Group of orthologs #123. Best score 816 bits Score difference with first non-orthologous sequence - AM1\_locus\_tags.txt:816 OB3b\_locus\_tags.txt:816

```
META1_4425          	100.00%		CQW49_RS00090       	100.00%
Bootstrap support for META1_4425 as seed ortholog is 100%.
Bootstrap support for CQW49_RS00090 as seed ortholog is 100%.
```

---

### Group of orthologs #124. Best score 814 bits Score difference with first non-orthologous sequence - AM1\_locus\_tags.txt:814 OB3b\_locus\_tags.txt:814

```
META1_0219          	100.00%		CQW49_RS12765       	100.00%
Bootstrap support for META1_0219 as seed ortholog is 100%.
Bootstrap support for CQW49_RS12765 as seed ortholog is 100%.
```

---

### Group of orthologs #125. Best score 813 bits Score difference with first non-orthologous sequence - AM1\_locus\_tags.txt:813 OB3b\_locus\_tags.txt:650

```
META1_3652          	100.00%		CQW49_RS11455       	100.00%
Bootstrap support for META1_3652 as seed ortholog is 100%.
Bootstrap support for CQW49_RS11455 as seed ortholog is 100%.
```

---

### Group of orthologs #126. Best score 812 bits Score difference with first non-orthologous sequence - AM1\_locus\_tags.txt:812 OB3b\_locus\_tags.txt:812

```
META1_3440          	100.00%		CQW49_RS10235       	100.00%
Bootstrap support for META1_3440 as seed ortholog is 100%.
Bootstrap support for CQW49_RS10235 as seed ortholog is 100%.
```

---

### Group of orthologs #127. Best score 809 bits Score difference with first non-orthologous sequence - AM1\_locus\_tags.txt:333 OB3b\_locus\_tags.txt:809

```
META1_5233          	100.00%		CQW49_RS19585       	100.00%
Bootstrap support for META1_5233 as seed ortholog is 100%.
Bootstrap support for CQW49_RS19585 as seed ortholog is 100%.
```

---

### Group of orthologs #128. Best score 808 bits Score difference with first non-orthologous sequence - AM1\_locus\_tags.txt:808 OB3b\_locus\_tags.txt:808

```
META1_4546          	100.00%		CQW49_RS06615       	100.00%
Bootstrap support for META1_4546 as seed ortholog is 100%.
Bootstrap support for CQW49_RS06615 as seed ortholog is 100%.
```

---

### Group of orthologs #129. Best score 804 bits Score difference with first non-orthologous sequence - AM1\_locus\_tags.txt:804 OB3b\_locus\_tags.txt:804

```
META1_2892          	100.00%		CQW49_RS11175       	100.00%
Bootstrap support for META1_2892 as seed ortholog is 100%.
Bootstrap support for CQW49_RS11175 as seed ortholog is 100%.
```

---

### Group of orthologs #130. Best score 804 bits Score difference with first non-orthologous sequence - AM1\_locus\_tags.txt:453 OB3b\_locus\_tags.txt:418

```
META1_4733          	100.00%		CQW49_RS05700       	100.00%
Bootstrap support for META1_4733 as seed ortholog is 100%.
Bootstrap support for CQW49_RS05700 as seed ortholog is 100%.
```

---

### Group of orthologs #131. Best score 803 bits Score difference with first non-orthologous sequence - AM1\_locus\_tags.txt:803 OB3b\_locus\_tags.txt:803

```
META1_4513          	100.00%		CQW49_RS16630       	100.00%
Bootstrap support for META1_4513 as seed ortholog is 100%.
Bootstrap support for CQW49_RS16630 as seed ortholog is 100%.
```

---

### Group of orthologs #132. Best score 800 bits Score difference with first non-orthologous sequence - AM1\_locus\_tags.txt:800 OB3b\_locus\_tags.txt:406

```
META1_5239          	100.00%		CQW49_RS19065       	100.00%
META1_3552          	24.14%		
Bootstrap support for META1_5239 as seed ortholog is 100%.
Bootstrap support for CQW49_RS19065 as seed ortholog is 100%.
```

---

### Group of orthologs #133. Best score 800 bits Score difference with first non-orthologous sequence - AM1\_locus\_tags.txt:120 OB3b\_locus\_tags.txt:800

```
META2_0992          	100.00%		CQW49_RS01055       	100.00%
Bootstrap support for META2_0992 as seed ortholog is 57%.
Alternative seed ortholog is META1_5091 (120 bits away from this cluster)
Bootstrap support for CQW49_RS01055 as seed ortholog is 100%.
```

---

### Group of orthologs #134. Best score 798 bits Score difference with first non-orthologous sequence - AM1\_locus\_tags.txt:798 OB3b\_locus\_tags.txt:798

```
META1_3277          	100.00%		CQW49_RS01110       	100.00%
Bootstrap support for META1_3277 as seed ortholog is 100%.
Bootstrap support for CQW49_RS01110 as seed ortholog is 100%.
```

---

### Group of orthologs #135. Best score 797 bits Score difference with first non-orthologous sequence - AM1\_locus\_tags.txt:797 OB3b\_locus\_tags.txt:797

```
META1_1293          	100.00%		CQW49_RS11320       	100.00%
Bootstrap support for META1_1293 as seed ortholog is 100%.
Bootstrap support for CQW49_RS11320 as seed ortholog is 100%.
```

---

### Group of orthologs #136. Best score 792 bits Score difference with first non-orthologous sequence - AM1\_locus\_tags.txt:359 OB3b\_locus\_tags.txt:408

```
META1_3473          	100.00%		CQW49_RS10065       	100.00%
Bootstrap support for META1_3473 as seed ortholog is 100%.
Bootstrap support for CQW49_RS10065 as seed ortholog is 100%.
```

---

### Group of orthologs #137. Best score 790 bits Score difference with first non-orthologous sequence - AM1\_locus\_tags.txt:647 OB3b\_locus\_tags.txt:353

```
META1_1359          	100.00%		CQW49_RS00305       	100.00%
Bootstrap support for META1_1359 as seed ortholog is 100%.
Bootstrap support for CQW49_RS00305 as seed ortholog is 100%.
```

---

### Group of orthologs #138. Best score 790 bits Score difference with first non-orthologous sequence - AM1\_locus\_tags.txt:145 OB3b\_locus\_tags.txt:790

```
META1_3624          	100.00%		CQW49_RS06220       	100.00%
Bootstrap support for META1_3624 as seed ortholog is 99%.
Bootstrap support for CQW49_RS06220 as seed ortholog is 100%.
```

---

### Group of orthologs #139. Best score 784 bits Score difference with first non-orthologous sequence - AM1\_locus\_tags.txt:784 OB3b\_locus\_tags.txt:784

```
META1_0347          	100.00%		CQW49_RS03830       	100.00%
Bootstrap support for META1_0347 as seed ortholog is 100%.
Bootstrap support for CQW49_RS03830 as seed ortholog is 100%.
```

---

### Group of orthologs #140. Best score 781 bits Score difference with first non-orthologous sequence - AM1\_locus\_tags.txt:634 OB3b\_locus\_tags.txt:781

```
META1_2379          	100.00%		CQW49_RS20740       	100.00%
Bootstrap support for META1_2379 as seed ortholog is 100%.
Bootstrap support for CQW49_RS20740 as seed ortholog is 100%.
```

---

### Group of orthologs #141. Best score 780 bits Score difference with first non-orthologous sequence - AM1\_locus\_tags.txt:780 OB3b\_locus\_tags.txt:780

```
META1_3307          	100.00%		CQW49_RS10595       	100.00%
Bootstrap support for META1_3307 as seed ortholog is 100%.
Bootstrap support for CQW49_RS10595 as seed ortholog is 100%.
```

---

### Group of orthologs #142. Best score 774 bits Score difference with first non-orthologous sequence - AM1\_locus\_tags.txt:484 OB3b\_locus\_tags.txt:688

```
META1_5196          	100.00%		CQW49_RS16495       	100.00%
Bootstrap support for META1_5196 as seed ortholog is 100%.
Bootstrap support for CQW49_RS16495 as seed ortholog is 100%.
```

---

### Group of orthologs #143. Best score 773 bits Score difference with first non-orthologous sequence - AM1\_locus\_tags.txt:497 OB3b\_locus\_tags.txt:773

```
META1_3433          	100.00%		CQW49_RS19355       	100.00%
Bootstrap support for META1_3433 as seed ortholog is 100%.
Bootstrap support for CQW49_RS19355 as seed ortholog is 100%.
```

---

### Group of orthologs #144. Best score 772 bits Score difference with first non-orthologous sequence - AM1\_locus\_tags.txt:410 OB3b\_locus\_tags.txt:772

```
META1_0699          	100.00%		CQW49_RS04095       	100.00%
Bootstrap support for META1_0699 as seed ortholog is 100%.
Bootstrap support for CQW49_RS04095 as seed ortholog is 100%.
```

---

### Group of orthologs #145. Best score 769 bits Score difference with first non-orthologous sequence - AM1\_locus\_tags.txt:722 OB3b\_locus\_tags.txt:769

```
META1_1797          	100.00%		CQW49_RS04450       	100.00%
Bootstrap support for META1_1797 as seed ortholog is 100%.
Bootstrap support for CQW49_RS04450 as seed ortholog is 100%.
```

---

### Group of orthologs #146. Best score 769 bits Score difference with first non-orthologous sequence - AM1\_locus\_tags.txt:529 OB3b\_locus\_tags.txt:561

```
META1_2223          	100.00%		CQW49_RS16385       	100.00%
Bootstrap support for META1_2223 as seed ortholog is 100%.
Bootstrap support for CQW49_RS16385 as seed ortholog is 100%.
```

---

### Group of orthologs #147. Best score 767 bits Score difference with first non-orthologous sequence - AM1\_locus\_tags.txt:767 OB3b\_locus\_tags.txt:667

```
META1_4642          	100.00%		CQW49_RS05985       	100.00%
Bootstrap support for META1_4642 as seed ortholog is 100%.
Bootstrap support for CQW49_RS05985 as seed ortholog is 100%.
```

---

### Group of orthologs #148. Best score 767 bits Score difference with first non-orthologous sequence - AM1\_locus\_tags.txt:767 OB3b\_locus\_tags.txt:767

```
META1_3645          	100.00%		CQW49_RS12665       	100.00%
Bootstrap support for META1_3645 as seed ortholog is 100%.
Bootstrap support for CQW49_RS12665 as seed ortholog is 100%.
```

---

### Group of orthologs #149. Best score 767 bits Score difference with first non-orthologous sequence - AM1\_locus\_tags.txt:767 OB3b\_locus\_tags.txt:767

```
META1_4876          	100.00%		CQW49_RS14325       	100.00%
Bootstrap support for META1_4876 as seed ortholog is 100%.
Bootstrap support for CQW49_RS14325 as seed ortholog is 100%.
```

---

### Group of orthologs #150. Best score 765 bits Score difference with first non-orthologous sequence - AM1\_locus\_tags.txt:577 OB3b\_locus\_tags.txt:628

```
META1_4883          	100.00%		CQW49_RS09375       	100.00%
Bootstrap support for META1_4883 as seed ortholog is 100%.
Bootstrap support for CQW49_RS09375 as seed ortholog is 100%.
```

---

### Group of orthologs #151. Best score 764 bits Score difference with first non-orthologous sequence - AM1\_locus\_tags.txt:668 OB3b\_locus\_tags.txt:764

```
META1_3397          	100.00%		CQW49_RS02910       	100.00%
Bootstrap support for META1_3397 as seed ortholog is 100%.
Bootstrap support for CQW49_RS02910 as seed ortholog is 100%.
```

---

### Group of orthologs #152. Best score 764 bits Score difference with first non-orthologous sequence - AM1\_locus\_tags.txt:646 OB3b\_locus\_tags.txt:764

```
META1_2566          	100.00%		CQW49_RS21670       	100.00%
Bootstrap support for META1_2566 as seed ortholog is 100%.
Bootstrap support for CQW49_RS21670 as seed ortholog is 100%.
```

---

### Group of orthologs #153. Best score 754 bits Score difference with first non-orthologous sequence - AM1\_locus\_tags.txt:754 OB3b\_locus\_tags.txt:754

```
META2_1021          	100.00%		CQW49_RS09855       	100.00%
Bootstrap support for META2_1021 as seed ortholog is 100%.
Bootstrap support for CQW49_RS09855 as seed ortholog is 100%.
```

---

### Group of orthologs #154. Best score 753 bits Score difference with first non-orthologous sequence - AM1\_locus\_tags.txt:634 OB3b\_locus\_tags.txt:753

```
META1_4875          	100.00%		CQW49_RS14340       	100.00%
Bootstrap support for META1_4875 as seed ortholog is 100%.
Bootstrap support for CQW49_RS14340 as seed ortholog is 100%.
```

---

### Group of orthologs #155. Best score 752 bits Score difference with first non-orthologous sequence - AM1\_locus\_tags.txt:752 OB3b\_locus\_tags.txt:752

```
META1_0328          	100.00%		CQW49_RS00020       	100.00%
Bootstrap support for META1_0328 as seed ortholog is 100%.
Bootstrap support for CQW49_RS00020 as seed ortholog is 100%.
```

---

### Group of orthologs #156. Best score 750 bits Score difference with first non-orthologous sequence - AM1\_locus\_tags.txt:750 OB3b\_locus\_tags.txt:362

```
META1_0866          	100.00%		CQW49_RS05020       	100.00%
Bootstrap support for META1_0866 as seed ortholog is 100%.
Bootstrap support for CQW49_RS05020 as seed ortholog is 100%.
```

---

### Group of orthologs #157. Best score 750 bits Score difference with first non-orthologous sequence - AM1\_locus\_tags.txt:750 OB3b\_locus\_tags.txt:750

```
META1_2535          	100.00%		CQW49_RS01750       	100.00%
Bootstrap support for META1_2535 as seed ortholog is 100%.
Bootstrap support for CQW49_RS01750 as seed ortholog is 100%.
```

---

### Group of orthologs #158. Best score 748 bits Score difference with first non-orthologous sequence - AM1\_locus\_tags.txt:748 OB3b\_locus\_tags.txt:748

```
META1_0132          	100.00%		CQW49_RS10150       	100.00%
Bootstrap support for META1_0132 as seed ortholog is 100%.
Bootstrap support for CQW49_RS10150 as seed ortholog is 100%.
```

---

### Group of orthologs #159. Best score 748 bits Score difference with first non-orthologous sequence - AM1\_locus\_tags.txt:748 OB3b\_locus\_tags.txt:748

```
META1_2024          	100.00%		CQW49_RS06010       	100.00%
Bootstrap support for META1_2024 as seed ortholog is 100%.
Bootstrap support for CQW49_RS06010 as seed ortholog is 100%.
```

---

### Group of orthologs #160. Best score 745 bits Score difference with first non-orthologous sequence - AM1\_locus\_tags.txt:745 OB3b\_locus\_tags.txt:593

```
META1_2448          	100.00%		CQW49_RS17420       	100.00%
Bootstrap support for META1_2448 as seed ortholog is 100%.
Bootstrap support for CQW49_RS17420 as seed ortholog is 100%.
```

---

### Group of orthologs #161. Best score 739 bits Score difference with first non-orthologous sequence - AM1\_locus\_tags.txt:739 OB3b\_locus\_tags.txt:739

```
META1_0329          	100.00%		CQW49_RS12230       	100.00%
Bootstrap support for META1_0329 as seed ortholog is 100%.
Bootstrap support for CQW49_RS12230 as seed ortholog is 100%.
```

---

### Group of orthologs #162. Best score 733 bits Score difference with first non-orthologous sequence - AM1\_locus\_tags.txt:733 OB3b\_locus\_tags.txt:733

```
META1_4320          	100.00%		CQW49_RS02025       	100.00%
Bootstrap support for META1_4320 as seed ortholog is 100%.
Bootstrap support for CQW49_RS02025 as seed ortholog is 100%.
```

---

### Group of orthologs #163. Best score 733 bits Score difference with first non-orthologous sequence - AM1\_locus\_tags.txt:733 OB3b\_locus\_tags.txt:733

```
META1_2420          	100.00%		CQW49_RS16145       	100.00%
Bootstrap support for META1_2420 as seed ortholog is 100%.
Bootstrap support for CQW49_RS16145 as seed ortholog is 100%.
```

---

### Group of orthologs #164. Best score 732 bits Score difference with first non-orthologous sequence - AM1\_locus\_tags.txt:624 OB3b\_locus\_tags.txt:622

```
META1_0178          	100.00%		CQW49_RS03800       	100.00%
Bootstrap support for META1_0178 as seed ortholog is 100%.
Bootstrap support for CQW49_RS03800 as seed ortholog is 100%.
```

---

### Group of orthologs #165. Best score 731 bits Score difference with first non-orthologous sequence - AM1\_locus\_tags.txt:616 OB3b\_locus\_tags.txt:590

```
META1_0843          	100.00%		CQW49_RS05295       	100.00%
Bootstrap support for META1_0843 as seed ortholog is 100%.
Bootstrap support for CQW49_RS05295 as seed ortholog is 100%.
```

---

### Group of orthologs #166. Best score 731 bits Score difference with first non-orthologous sequence - AM1\_locus\_tags.txt:731 OB3b\_locus\_tags.txt:731

```
META1_4800          	100.00%		CQW49_RS16020       	100.00%
Bootstrap support for META1_4800 as seed ortholog is 100%.
Bootstrap support for CQW49_RS16020 as seed ortholog is 100%.
```

---

### Group of orthologs #167. Best score 730 bits Score difference with first non-orthologous sequence - AM1\_locus\_tags.txt:587 OB3b\_locus\_tags.txt:730

```
META1_3538          	100.00%		CQW49_RS01100       	100.00%
Bootstrap support for META1_3538 as seed ortholog is 100%.
Bootstrap support for CQW49_RS01100 as seed ortholog is 100%.
```

---

### Group of orthologs #168. Best score 727 bits Score difference with first non-orthologous sequence - AM1\_locus\_tags.txt:463 OB3b\_locus\_tags.txt:727

```
META1_0850          	100.00%		CQW49_RS05330       	100.00%
Bootstrap support for META1_0850 as seed ortholog is 100%.
Bootstrap support for CQW49_RS05330 as seed ortholog is 100%.
```

---

### Group of orthologs #169. Best score 727 bits Score difference with first non-orthologous sequence - AM1\_locus\_tags.txt:727 OB3b\_locus\_tags.txt:727

```
META1_3145          	100.00%		CQW49_RS01170       	100.00%
Bootstrap support for META1_3145 as seed ortholog is 100%.
Bootstrap support for CQW49_RS01170 as seed ortholog is 100%.
```

---

### Group of orthologs #170. Best score 725 bits Score difference with first non-orthologous sequence - AM1\_locus\_tags.txt:725 OB3b\_locus\_tags.txt:725

```
META1_4314          	100.00%		CQW49_RS03770       	100.00%
Bootstrap support for META1_4314 as seed ortholog is 100%.
Bootstrap support for CQW49_RS03770 as seed ortholog is 100%.
```

---

### Group of orthologs #171. Best score 723 bits Score difference with first non-orthologous sequence - AM1\_locus\_tags.txt:627 OB3b\_locus\_tags.txt:723

```
META1_4662          	100.00%		CQW49_RS00850       	100.00%
Bootstrap support for META1_4662 as seed ortholog is 100%.
Bootstrap support for CQW49_RS00850 as seed ortholog is 100%.
```

---

### Group of orthologs #172. Best score 722 bits Score difference with first non-orthologous sequence - AM1\_locus\_tags.txt:722 OB3b\_locus\_tags.txt:722

```
META1_4832          	100.00%		CQW49_RS10530       	100.00%
META1_2151          	98.48%		CQW49_RS02140       	100.00%
Bootstrap support for META1_4832 as seed ortholog is 100%.
Bootstrap support for CQW49_RS10530 as seed ortholog is 100%.
Bootstrap support for CQW49_RS02140 as seed ortholog is 100%.
```

---

### Group of orthologs #173. Best score 722 bits Score difference with first non-orthologous sequence - AM1\_locus\_tags.txt:541 OB3b\_locus\_tags.txt:515

```
META1_1984          	100.00%		CQW49_RS14935       	100.00%
META1_4415          	27.47%		
Bootstrap support for META1_1984 as seed ortholog is 100%.
Bootstrap support for CQW49_RS14935 as seed ortholog is 100%.
```

---

### Group of orthologs #174. Best score 719 bits Score difference with first non-orthologous sequence - AM1\_locus\_tags.txt:719 OB3b\_locus\_tags.txt:719

```
META1_0773          	100.00%		CQW49_RS16840       	100.00%
Bootstrap support for META1_0773 as seed ortholog is 100%.
Bootstrap support for CQW49_RS16840 as seed ortholog is 100%.
```

---

### Group of orthologs #175. Best score 718 bits Score difference with first non-orthologous sequence - AM1\_locus\_tags.txt:416 OB3b\_locus\_tags.txt:652

```
META1_1807          	100.00%		CQW49_RS06075       	100.00%
Bootstrap support for META1_1807 as seed ortholog is 100%.
Bootstrap support for CQW49_RS06075 as seed ortholog is 100%.
```

---

### Group of orthologs #176. Best score 715 bits Score difference with first non-orthologous sequence - AM1\_locus\_tags.txt:446 OB3b\_locus\_tags.txt:502

```
META1_3021          	100.00%		CQW49_RS01665       	100.00%
Bootstrap support for META1_3021 as seed ortholog is 100%.
Bootstrap support for CQW49_RS01665 as seed ortholog is 100%.
```

---

### Group of orthologs #177. Best score 714 bits Score difference with first non-orthologous sequence - AM1\_locus\_tags.txt:714 OB3b\_locus\_tags.txt:714

```
META1_5129          	100.00%		CQW49_RS04760       	100.00%
Bootstrap support for META1_5129 as seed ortholog is 100%.
Bootstrap support for CQW49_RS04760 as seed ortholog is 100%.
```

---

### Group of orthologs #178. Best score 713 bits Score difference with first non-orthologous sequence - AM1\_locus\_tags.txt:713 OB3b\_locus\_tags.txt:713

```
META1_2269          	100.00%		CQW49_RS02005       	100.00%
Bootstrap support for META1_2269 as seed ortholog is 100%.
Bootstrap support for CQW49_RS02005 as seed ortholog is 100%.
```

---

### Group of orthologs #179. Best score 713 bits Score difference with first non-orthologous sequence - AM1\_locus\_tags.txt:713 OB3b\_locus\_tags.txt:713

```
META1_2043          	100.00%		CQW49_RS15640       	100.00%
Bootstrap support for META1_2043 as seed ortholog is 100%.
Bootstrap support for CQW49_RS15640 as seed ortholog is 100%.
```

---

### Group of orthologs #180. Best score 707 bits Score difference with first non-orthologous sequence - AM1\_locus\_tags.txt:707 OB3b\_locus\_tags.txt:511

```
META1_2867          	100.00%		CQW49_RS13165       	100.00%
Bootstrap support for META1_2867 as seed ortholog is 100%.
Bootstrap support for CQW49_RS13165 as seed ortholog is 100%.
```

---

### Group of orthologs #181. Best score 703 bits Score difference with first non-orthologous sequence - AM1\_locus\_tags.txt:54 OB3b\_locus\_tags.txt:201

```
META1_4969          	100.00%		CQW49_RS14895       	100.00%
Bootstrap support for META1_4969 as seed ortholog is 83%.
Bootstrap support for CQW49_RS14895 as seed ortholog is 99%.
```

---

### Group of orthologs #182. Best score 702 bits Score difference with first non-orthologous sequence - AM1\_locus\_tags.txt:608 OB3b\_locus\_tags.txt:601

```
META1_1268          	100.00%		CQW49_RS11030       	100.00%
Bootstrap support for META1_1268 as seed ortholog is 100%.
Bootstrap support for CQW49_RS11030 as seed ortholog is 100%.
```

---

### Group of orthologs #183. Best score 702 bits Score difference with first non-orthologous sequence - AM1\_locus\_tags.txt:634 OB3b\_locus\_tags.txt:702

```
META1_1454          	100.00%		CQW49_RS12810       	100.00%
Bootstrap support for META1_1454 as seed ortholog is 100%.
Bootstrap support for CQW49_RS12810 as seed ortholog is 100%.
```

---

### Group of orthologs #184. Best score 702 bits Score difference with first non-orthologous sequence - AM1\_locus\_tags.txt:702 OB3b\_locus\_tags.txt:702

```
META1_5088          	100.00%		CQW49_RS11230       	100.00%
Bootstrap support for META1_5088 as seed ortholog is 100%.
Bootstrap support for CQW49_RS11230 as seed ortholog is 100%.
```

---

### Group of orthologs #185. Best score 701 bits Score difference with first non-orthologous sequence - AM1\_locus\_tags.txt:556 OB3b\_locus\_tags.txt:701

```
META1_3302          	100.00%		CQW49_RS10985       	100.00%
Bootstrap support for META1_3302 as seed ortholog is 100%.
Bootstrap support for CQW49_RS10985 as seed ortholog is 100%.
```

---

### Group of orthologs #186. Best score 699 bits Score difference with first non-orthologous sequence - AM1\_locus\_tags.txt:699 OB3b\_locus\_tags.txt:699

```
META2_0910          	100.00%		CQW49_RS18980       	100.00%
Bootstrap support for META2_0910 as seed ortholog is 100%.
Bootstrap support for CQW49_RS18980 as seed ortholog is 100%.
```

---

### Group of orthologs #187. Best score 696 bits Score difference with first non-orthologous sequence - AM1\_locus\_tags.txt:432 OB3b\_locus\_tags.txt:539

```
META1_5286          	100.00%		CQW49_RS16600       	100.00%
Bootstrap support for META1_5286 as seed ortholog is 100%.
Bootstrap support for CQW49_RS16600 as seed ortholog is 100%.
```

---

### Group of orthologs #188. Best score 695 bits Score difference with first non-orthologous sequence - AM1\_locus\_tags.txt:695 OB3b\_locus\_tags.txt:695

```
META1_2958          	100.00%		CQW49_RS13105       	100.00%
Bootstrap support for META1_2958 as seed ortholog is 100%.
Bootstrap support for CQW49_RS13105 as seed ortholog is 100%.
```

---

### Group of orthologs #189. Best score 693 bits Score difference with first non-orthologous sequence - AM1\_locus\_tags.txt:693 OB3b\_locus\_tags.txt:693

```
META1_0128          	100.00%		CQW49_RS10650       	100.00%
Bootstrap support for META1_0128 as seed ortholog is 100%.
Bootstrap support for CQW49_RS10650 as seed ortholog is 100%.
```

---

### Group of orthologs #190. Best score 693 bits Score difference with first non-orthologous sequence - AM1\_locus\_tags.txt:693 OB3b\_locus\_tags.txt:693

```
META1_3384          	100.00%		CQW49_RS06375       	100.00%
Bootstrap support for META1_3384 as seed ortholog is 100%.
Bootstrap support for CQW49_RS06375 as seed ortholog is 100%.
```

---

### Group of orthologs #191. Best score 691 bits Score difference with first non-orthologous sequence - AM1\_locus\_tags.txt:691 OB3b\_locus\_tags.txt:691

```
META1_3354          	100.00%		CQW49_RS01455       	100.00%
Bootstrap support for META1_3354 as seed ortholog is 100%.
Bootstrap support for CQW49_RS01455 as seed ortholog is 100%.
```

---

### Group of orthologs #192. Best score 691 bits Score difference with first non-orthologous sequence - AM1\_locus\_tags.txt:691 OB3b\_locus\_tags.txt:691

```
META1_4882          	100.00%		CQW49_RS09355       	100.00%
Bootstrap support for META1_4882 as seed ortholog is 100%.
Bootstrap support for CQW49_RS09355 as seed ortholog is 100%.
```

---

### Group of orthologs #193. Best score 690 bits Score difference with first non-orthologous sequence - AM1\_locus\_tags.txt:189 OB3b\_locus\_tags.txt:690

```
META1_1489          	100.00%		CQW49_RS11790       	100.00%
Bootstrap support for META1_1489 as seed ortholog is 100%.
Bootstrap support for CQW49_RS11790 as seed ortholog is 100%.
```

---

### Group of orthologs #194. Best score 689 bits Score difference with first non-orthologous sequence - AM1\_locus\_tags.txt:285 OB3b\_locus\_tags.txt:448

```
META1_3547          	100.00%		CQW49_RS03815       	100.00%
Bootstrap support for META1_3547 as seed ortholog is 100%.
Bootstrap support for CQW49_RS03815 as seed ortholog is 100%.
```

---

### Group of orthologs #195. Best score 687 bits Score difference with first non-orthologous sequence - AM1\_locus\_tags.txt:687 OB3b\_locus\_tags.txt:687

```
META1_2294          	100.00%		CQW49_RS15660       	100.00%
Bootstrap support for META1_2294 as seed ortholog is 100%.
Bootstrap support for CQW49_RS15660 as seed ortholog is 100%.
```

---

### Group of orthologs #196. Best score 687 bits Score difference with first non-orthologous sequence - AM1\_locus\_tags.txt:687 OB3b\_locus\_tags.txt:307

```
META1_3304          	100.00%		CQW49_RS10975       	100.00%
Bootstrap support for META1_3304 as seed ortholog is 100%.
Bootstrap support for CQW49_RS10975 as seed ortholog is 100%.
```

---

### Group of orthologs #197. Best score 685 bits Score difference with first non-orthologous sequence - AM1\_locus\_tags.txt:413 OB3b\_locus\_tags.txt:308

```
META1_0005          	100.00%		CQW49_RS21260       	100.00%
Bootstrap support for META1_0005 as seed ortholog is 100%.
Bootstrap support for CQW49_RS21260 as seed ortholog is 100%.
```

---

### Group of orthologs #198. Best score 682 bits Score difference with first non-orthologous sequence - AM1\_locus\_tags.txt:682 OB3b\_locus\_tags.txt:682

```
META1_1519          	100.00%		CQW49_RS19865       	100.00%
Bootstrap support for META1_1519 as seed ortholog is 100%.
Bootstrap support for CQW49_RS19865 as seed ortholog is 100%.
```

---

### Group of orthologs #199. Best score 681 bits Score difference with first non-orthologous sequence - AM1\_locus\_tags.txt:681 OB3b\_locus\_tags.txt:681

```
META1_4926          	100.00%		CQW49_RS05005       	100.00%
Bootstrap support for META1_4926 as seed ortholog is 100%.
Bootstrap support for CQW49_RS05005 as seed ortholog is 100%.
```

---

### Group of orthologs #200. Best score 680 bits Score difference with first non-orthologous sequence - AM1\_locus\_tags.txt:273 OB3b\_locus\_tags.txt:279

```
META1_0236          	100.00%		CQW49_RS06110       	100.00%
Bootstrap support for META1_0236 as seed ortholog is 100%.
Bootstrap support for CQW49_RS06110 as seed ortholog is 100%.
```

---

### Group of orthologs #201. Best score 678 bits Score difference with first non-orthologous sequence - AM1\_locus\_tags.txt:373 OB3b\_locus\_tags.txt:503

```
META1_2359          	100.00%		CQW49_RS01065       	100.00%
Bootstrap support for META1_2359 as seed ortholog is 100%.
Bootstrap support for CQW49_RS01065 as seed ortholog is 100%.
```

---

### Group of orthologs #202. Best score 678 bits Score difference with first non-orthologous sequence - AM1\_locus\_tags.txt:678 OB3b\_locus\_tags.txt:678

```
META1_2199          	100.00%		CQW49_RS16345       	100.00%
Bootstrap support for META1_2199 as seed ortholog is 100%.
Bootstrap support for CQW49_RS16345 as seed ortholog is 100%.
```

---

### Group of orthologs #203. Best score 677 bits Score difference with first non-orthologous sequence - AM1\_locus\_tags.txt:677 OB3b\_locus\_tags.txt:677

```
META1_5130          	100.00%		CQW49_RS04755       	100.00%
Bootstrap support for META1_5130 as seed ortholog is 100%.
Bootstrap support for CQW49_RS04755 as seed ortholog is 100%.
```

---

### Group of orthologs #204. Best score 675 bits Score difference with first non-orthologous sequence - AM1\_locus\_tags.txt:414 OB3b\_locus\_tags.txt:675

```
META1_0840          	100.00%		CQW49_RS05280       	100.00%
Bootstrap support for META1_0840 as seed ortholog is 100%.
Bootstrap support for CQW49_RS05280 as seed ortholog is 100%.
```

---

### Group of orthologs #205. Best score 673 bits Score difference with first non-orthologous sequence - AM1\_locus\_tags.txt:673 OB3b\_locus\_tags.txt:673

```
META1_0477          	100.00%		CQW49_RS20425       	100.00%
Bootstrap support for META1_0477 as seed ortholog is 100%.
Bootstrap support for CQW49_RS20425 as seed ortholog is 100%.
```

---

### Group of orthologs #206. Best score 672 bits Score difference with first non-orthologous sequence - AM1\_locus\_tags.txt:672 OB3b\_locus\_tags.txt:672

```
META1_0049          	100.00%		CQW49_RS17185       	100.00%
Bootstrap support for META1_0049 as seed ortholog is 100%.
Bootstrap support for CQW49_RS17185 as seed ortholog is 100%.
```

---

### Group of orthologs #207. Best score 671 bits Score difference with first non-orthologous sequence - AM1\_locus\_tags.txt:379 OB3b\_locus\_tags.txt:413

```
META1_2950          	100.00%		CQW49_RS06030       	100.00%
Bootstrap support for META1_2950 as seed ortholog is 100%.
Bootstrap support for CQW49_RS06030 as seed ortholog is 100%.
```

---

### Group of orthologs #208. Best score 671 bits Score difference with first non-orthologous sequence - AM1\_locus\_tags.txt:671 OB3b\_locus\_tags.txt:671

```
META1_4700          	100.00%		CQW49_RS20570       	100.00%
Bootstrap support for META1_4700 as seed ortholog is 100%.
Bootstrap support for CQW49_RS20570 as seed ortholog is 100%.
```

---

### Group of orthologs #209. Best score 665 bits Score difference with first non-orthologous sequence - AM1\_locus\_tags.txt:665 OB3b\_locus\_tags.txt:665

```
META1_2354          	100.00%		CQW49_RS08615       	100.00%
Bootstrap support for META1_2354 as seed ortholog is 100%.
Bootstrap support for CQW49_RS08615 as seed ortholog is 100%.
```

---

### Group of orthologs #210. Best score 664 bits Score difference with first non-orthologous sequence - AM1\_locus\_tags.txt:587 OB3b\_locus\_tags.txt:664

```
META1_0776          	100.00%		CQW49_RS06980       	100.00%
Bootstrap support for META1_0776 as seed ortholog is 100%.
Bootstrap support for CQW49_RS06980 as seed ortholog is 100%.
```

---

### Group of orthologs #211. Best score 664 bits Score difference with first non-orthologous sequence - AM1\_locus\_tags.txt:411 OB3b\_locus\_tags.txt:470

```
META1_3024          	100.00%		CQW49_RS01645       	100.00%
Bootstrap support for META1_3024 as seed ortholog is 100%.
Bootstrap support for CQW49_RS01645 as seed ortholog is 100%.
```

---

### Group of orthologs #212. Best score 664 bits Score difference with first non-orthologous sequence - AM1\_locus\_tags.txt:664 OB3b\_locus\_tags.txt:414

```
META1_2987          	100.00%		CQW49_RS01950       	100.00%
Bootstrap support for META1_2987 as seed ortholog is 100%.
Bootstrap support for CQW49_RS01950 as seed ortholog is 100%.
```

---

### Group of orthologs #213. Best score 664 bits Score difference with first non-orthologous sequence - AM1\_locus\_tags.txt:664 OB3b\_locus\_tags.txt:664

```
META1_0165          	100.00%		CQW49_RS18905       	100.00%
Bootstrap support for META1_0165 as seed ortholog is 100%.
Bootstrap support for CQW49_RS18905 as seed ortholog is 100%.
```

---

### Group of orthologs #214. Best score 663 bits Score difference with first non-orthologous sequence - AM1\_locus\_tags.txt:663 OB3b\_locus\_tags.txt:80

```
META1_0852          	100.00%		CQW49_RS05340       	100.00%
Bootstrap support for META1_0852 as seed ortholog is 100%.
Bootstrap support for CQW49_RS05340 as seed ortholog is 99%.
```

---

### Group of orthologs #215. Best score 663 bits Score difference with first non-orthologous sequence - AM1\_locus\_tags.txt:203 OB3b\_locus\_tags.txt:284

```
META1_2222          	100.00%		CQW49_RS10240       	100.00%
Bootstrap support for META1_2222 as seed ortholog is 100%.
Bootstrap support for CQW49_RS10240 as seed ortholog is 100%.
```

---

### Group of orthologs #216. Best score 662 bits Score difference with first non-orthologous sequence - AM1\_locus\_tags.txt:662 OB3b\_locus\_tags.txt:662

```
META1_3905          	100.00%		CQW49_RS05040       	100.00%
Bootstrap support for META1_3905 as seed ortholog is 100%.
Bootstrap support for CQW49_RS05040 as seed ortholog is 100%.
```

---

### Group of orthologs #217. Best score 662 bits Score difference with first non-orthologous sequence - AM1\_locus\_tags.txt:662 OB3b\_locus\_tags.txt:662

```
META1_4287          	100.00%		CQW49_RS15745       	100.00%
Bootstrap support for META1_4287 as seed ortholog is 100%.
Bootstrap support for CQW49_RS15745 as seed ortholog is 100%.
```

---

### Group of orthologs #218. Best score 661 bits Score difference with first non-orthologous sequence - AM1\_locus\_tags.txt:661 OB3b\_locus\_tags.txt:661

```
META1_1974          	100.00%		CQW49_RS09110       	100.00%
Bootstrap support for META1_1974 as seed ortholog is 100%.
Bootstrap support for CQW49_RS09110 as seed ortholog is 100%.
```

---

### Group of orthologs #219. Best score 661 bits Score difference with first non-orthologous sequence - AM1\_locus\_tags.txt:410 OB3b\_locus\_tags.txt:487

```
META1_5117          	100.00%		CQW49_RS20340       	100.00%
Bootstrap support for META1_5117 as seed ortholog is 100%.
Bootstrap support for CQW49_RS20340 as seed ortholog is 100%.
```

---

### Group of orthologs #220. Best score 660 bits Score difference with first non-orthologous sequence - AM1\_locus\_tags.txt:660 OB3b\_locus\_tags.txt:660

```
META1_3148          	100.00%		CQW49_RS11265       	100.00%
Bootstrap support for META1_3148 as seed ortholog is 100%.
Bootstrap support for CQW49_RS11265 as seed ortholog is 100%.
```

---

### Group of orthologs #221. Best score 660 bits Score difference with first non-orthologous sequence - AM1\_locus\_tags.txt:546 OB3b\_locus\_tags.txt:552

```
META1_4494          	100.00%		CQW49_RS11885       	100.00%
Bootstrap support for META1_4494 as seed ortholog is 100%.
Bootstrap support for CQW49_RS11885 as seed ortholog is 100%.
```

---

### Group of orthologs #222. Best score 657 bits Score difference with first non-orthologous sequence - AM1\_locus\_tags.txt:657 OB3b\_locus\_tags.txt:657

```
META1_3503          	100.00%		CQW49_RS11985       	100.00%
Bootstrap support for META1_3503 as seed ortholog is 100%.
Bootstrap support for CQW49_RS11985 as seed ortholog is 100%.
```

---

### Group of orthologs #223. Best score 655 bits Score difference with first non-orthologous sequence - AM1\_locus\_tags.txt:655 OB3b\_locus\_tags.txt:655

```
META1_1446          	100.00%		CQW49_RS13080       	100.00%
Bootstrap support for META1_1446 as seed ortholog is 100%.
Bootstrap support for CQW49_RS13080 as seed ortholog is 100%.
```

---

### Group of orthologs #224. Best score 655 bits Score difference with first non-orthologous sequence - AM1\_locus\_tags.txt:400 OB3b\_locus\_tags.txt:655

```
META1_4847          	100.00%		CQW49_RS16670       	100.00%
Bootstrap support for META1_4847 as seed ortholog is 100%.
Bootstrap support for CQW49_RS16670 as seed ortholog is 100%.
```

---

### Group of orthologs #225. Best score 654 bits Score difference with first non-orthologous sequence - AM1\_locus\_tags.txt:476 OB3b\_locus\_tags.txt:448

```
META1_0340          	100.00%		CQW49_RS09335       	100.00%
Bootstrap support for META1_0340 as seed ortholog is 100%.
Bootstrap support for CQW49_RS09335 as seed ortholog is 100%.
```

---

### Group of orthologs #226. Best score 653 bits Score difference with first non-orthologous sequence - AM1\_locus\_tags.txt:653 OB3b\_locus\_tags.txt:653

```
META1_2172          	100.00%		CQW49_RS10420       	100.00%
Bootstrap support for META1_2172 as seed ortholog is 100%.
Bootstrap support for CQW49_RS10420 as seed ortholog is 100%.
```

---

### Group of orthologs #227. Best score 650 bits Score difference with first non-orthologous sequence - AM1\_locus\_tags.txt:430 OB3b\_locus\_tags.txt:650

```
META1_2990          	100.00%		CQW49_RS06870       	100.00%
Bootstrap support for META1_2990 as seed ortholog is 100%.
Bootstrap support for CQW49_RS06870 as seed ortholog is 100%.
```

---

### Group of orthologs #228. Best score 649 bits Score difference with first non-orthologous sequence - AM1\_locus\_tags.txt:602 OB3b\_locus\_tags.txt:649

```
META1_5297          	100.00%		CQW49_RS20465       	100.00%
Bootstrap support for META1_5297 as seed ortholog is 100%.
Bootstrap support for CQW49_RS20465 as seed ortholog is 100%.
```

---

### Group of orthologs #229. Best score 648 bits Score difference with first non-orthologous sequence - AM1\_locus\_tags.txt:432 OB3b\_locus\_tags.txt:648

```
META1_0486          	100.00%		CQW49_RS10875       	100.00%
Bootstrap support for META1_0486 as seed ortholog is 100%.
Bootstrap support for CQW49_RS10875 as seed ortholog is 100%.
```

---

### Group of orthologs #230. Best score 648 bits Score difference with first non-orthologous sequence - AM1\_locus\_tags.txt:648 OB3b\_locus\_tags.txt:648

```
META1_2984          	100.00%		CQW49_RS08635       	100.00%
Bootstrap support for META1_2984 as seed ortholog is 100%.
Bootstrap support for CQW49_RS08635 as seed ortholog is 100%.
```

---

### Group of orthologs #231. Best score 648 bits Score difference with first non-orthologous sequence - AM1\_locus\_tags.txt:648 OB3b\_locus\_tags.txt:648

```
META1_4857          	100.00%		CQW49_RS15830       	100.00%
Bootstrap support for META1_4857 as seed ortholog is 100%.
Bootstrap support for CQW49_RS15830 as seed ortholog is 100%.
```

---

### Group of orthologs #232. Best score 645 bits Score difference with first non-orthologous sequence - AM1\_locus\_tags.txt:645 OB3b\_locus\_tags.txt:645

```
META1_2088          	100.00%		CQW49_RS04890       	100.00%
Bootstrap support for META1_2088 as seed ortholog is 100%.
Bootstrap support for CQW49_RS04890 as seed ortholog is 100%.
```

---

### Group of orthologs #233. Best score 643 bits Score difference with first non-orthologous sequence - AM1\_locus\_tags.txt:643 OB3b\_locus\_tags.txt:643

```
META1_0471          	100.00%		CQW49_RS04415       	100.00%
Bootstrap support for META1_0471 as seed ortholog is 100%.
Bootstrap support for CQW49_RS04415 as seed ortholog is 100%.
```

---

### Group of orthologs #234. Best score 641 bits Score difference with first non-orthologous sequence - AM1\_locus\_tags.txt:283 OB3b\_locus\_tags.txt:641

```
META1_2959          	100.00%		CQW49_RS01310       	100.00%
Bootstrap support for META1_2959 as seed ortholog is 100%.
Bootstrap support for CQW49_RS01310 as seed ortholog is 100%.
```

---

### Group of orthologs #235. Best score 638 bits Score difference with first non-orthologous sequence - AM1\_locus\_tags.txt:523 OB3b\_locus\_tags.txt:504

```
META1_1822          	100.00%		CQW49_RS11755       	100.00%
META1_3235          	82.08%		
Bootstrap support for META1_1822 as seed ortholog is 100%.
Bootstrap support for CQW49_RS11755 as seed ortholog is 100%.
```

---

### Group of orthologs #236. Best score 638 bits Score difference with first non-orthologous sequence - AM1\_locus\_tags.txt:638 OB3b\_locus\_tags.txt:638

```
META1_0594          	100.00%		CQW49_RS10940       	100.00%
Bootstrap support for META1_0594 as seed ortholog is 100%.
Bootstrap support for CQW49_RS10940 as seed ortholog is 100%.
```

---

### Group of orthologs #237. Best score 637 bits Score difference with first non-orthologous sequence - AM1\_locus\_tags.txt:637 OB3b\_locus\_tags.txt:439

```
META1_3455          	100.00%		CQW49_RS15530       	100.00%
Bootstrap support for META1_3455 as seed ortholog is 100%.
Bootstrap support for CQW49_RS15530 as seed ortholog is 100%.
```

---

### Group of orthologs #238. Best score 636 bits Score difference with first non-orthologous sequence - AM1\_locus\_tags.txt:442 OB3b\_locus\_tags.txt:310

```
META1_1421          	100.00%		CQW49_RS08575       	100.00%
Bootstrap support for META1_1421 as seed ortholog is 100%.
Bootstrap support for CQW49_RS08575 as seed ortholog is 100%.
```

---

### Group of orthologs #239. Best score 635 bits Score difference with first non-orthologous sequence - AM1\_locus\_tags.txt:635 OB3b\_locus\_tags.txt:635

```
META1_1490          	100.00%		CQW49_RS12340       	100.00%
Bootstrap support for META1_1490 as seed ortholog is 100%.
Bootstrap support for CQW49_RS12340 as seed ortholog is 100%.
```

---

### Group of orthologs #240. Best score 634 bits Score difference with first non-orthologous sequence - AM1\_locus\_tags.txt:634 OB3b\_locus\_tags.txt:634

```
META1_2901          	100.00%		CQW49_RS02830       	100.00%
Bootstrap support for META1_2901 as seed ortholog is 100%.
Bootstrap support for CQW49_RS02830 as seed ortholog is 100%.
```

---

### Group of orthologs #241. Best score 634 bits Score difference with first non-orthologous sequence - AM1\_locus\_tags.txt:634 OB3b\_locus\_tags.txt:589

```
META1_3478          	100.00%		CQW49_RS02785       	100.00%
Bootstrap support for META1_3478 as seed ortholog is 100%.
Bootstrap support for CQW49_RS02785 as seed ortholog is 100%.
```

---

### Group of orthologs #242. Best score 633 bits Score difference with first non-orthologous sequence - AM1\_locus\_tags.txt:215 OB3b\_locus\_tags.txt:633

```
META1_0436          	100.00%		CQW49_RS04775       	100.00%
Bootstrap support for META1_0436 as seed ortholog is 100%.
Bootstrap support for CQW49_RS04775 as seed ortholog is 100%.
```

---

### Group of orthologs #243. Best score 633 bits Score difference with first non-orthologous sequence - AM1\_locus\_tags.txt:340 OB3b\_locus\_tags.txt:633

```
META1_1160          	100.00%		CQW49_RS11665       	100.00%
Bootstrap support for META1_1160 as seed ortholog is 100%.
Bootstrap support for CQW49_RS11665 as seed ortholog is 100%.
```

---

### Group of orthologs #244. Best score 632 bits Score difference with first non-orthologous sequence - AM1\_locus\_tags.txt:632 OB3b\_locus\_tags.txt:632

```
META1_3158          	100.00%		CQW49_RS17855       	100.00%
Bootstrap support for META1_3158 as seed ortholog is 100%.
Bootstrap support for CQW49_RS17855 as seed ortholog is 100%.
```

---

### Group of orthologs #245. Best score 628 bits Score difference with first non-orthologous sequence - AM1\_locus\_tags.txt:628 OB3b\_locus\_tags.txt:628

```
META1_3154          	100.00%		CQW49_RS11300       	100.00%
Bootstrap support for META1_3154 as seed ortholog is 100%.
Bootstrap support for CQW49_RS11300 as seed ortholog is 100%.
```

---

### Group of orthologs #246. Best score 628 bits Score difference with first non-orthologous sequence - AM1\_locus\_tags.txt:628 OB3b\_locus\_tags.txt:628

```
META1_4455          	100.00%		CQW49_RS12360       	100.00%
Bootstrap support for META1_4455 as seed ortholog is 100%.
Bootstrap support for CQW49_RS12360 as seed ortholog is 100%.
```

---

### Group of orthologs #247. Best score 626 bits Score difference with first non-orthologous sequence - AM1\_locus\_tags.txt:626 OB3b\_locus\_tags.txt:626

```
META1_1730          	100.00%		CQW49_RS12255       	100.00%
Bootstrap support for META1_1730 as seed ortholog is 100%.
Bootstrap support for CQW49_RS12255 as seed ortholog is 100%.
```

---

### Group of orthologs #248. Best score 626 bits Score difference with first non-orthologous sequence - AM1\_locus\_tags.txt:626 OB3b\_locus\_tags.txt:626

```
META1_4660          	100.00%		CQW49_RS18250       	100.00%
Bootstrap support for META1_4660 as seed ortholog is 100%.
Bootstrap support for CQW49_RS18250 as seed ortholog is 100%.
```

---

### Group of orthologs #249. Best score 624 bits Score difference with first non-orthologous sequence - AM1\_locus\_tags.txt:624 OB3b\_locus\_tags.txt:624

```
META2_1331          	100.00%		CQW49_RS18155       	100.00%
Bootstrap support for META2_1331 as seed ortholog is 100%.
Bootstrap support for CQW49_RS18155 as seed ortholog is 100%.
```

---

### Group of orthologs #250. Best score 619 bits Score difference with first non-orthologous sequence - AM1\_locus\_tags.txt:619 OB3b\_locus\_tags.txt:619

```
META1_1794          	100.00%		CQW49_RS00670       	100.00%
Bootstrap support for META1_1794 as seed ortholog is 100%.
Bootstrap support for CQW49_RS00670 as seed ortholog is 100%.
```

---

### Group of orthologs #251. Best score 619 bits Score difference with first non-orthologous sequence - AM1\_locus\_tags.txt:364 OB3b\_locus\_tags.txt:619

```
META1_2053          	100.00%		CQW49_RS01265       	100.00%
Bootstrap support for META1_2053 as seed ortholog is 100%.
Bootstrap support for CQW49_RS01265 as seed ortholog is 100%.
```

---

### Group of orthologs #252. Best score 619 bits Score difference with first non-orthologous sequence - AM1\_locus\_tags.txt:414 OB3b\_locus\_tags.txt:619

```
META1_3910          	100.00%		CQW49_RS01840       	100.00%
Bootstrap support for META1_3910 as seed ortholog is 100%.
Bootstrap support for CQW49_RS01840 as seed ortholog is 100%.
```

---

### Group of orthologs #253. Best score 617 bits Score difference with first non-orthologous sequence - AM1\_locus\_tags.txt:617 OB3b\_locus\_tags.txt:617

```
META1_4318          	100.00%		CQW49_RS03755       	100.00%
Bootstrap support for META1_4318 as seed ortholog is 100%.
Bootstrap support for CQW49_RS03755 as seed ortholog is 100%.
```

---

### Group of orthologs #254. Best score 616 bits Score difference with first non-orthologous sequence - AM1\_locus\_tags.txt:616 OB3b\_locus\_tags.txt:616

```
META1_1538          	100.00%		CQW49_RS19010       	100.00%
Bootstrap support for META1_1538 as seed ortholog is 100%.
Bootstrap support for CQW49_RS19010 as seed ortholog is 100%.
```

---

### Group of orthologs #255. Best score 614 bits Score difference with first non-orthologous sequence - AM1\_locus\_tags.txt:545 OB3b\_locus\_tags.txt:543

```
META1_1022          	100.00%		CQW49_RS12375       	100.00%
Bootstrap support for META1_1022 as seed ortholog is 100%.
Bootstrap support for CQW49_RS12375 as seed ortholog is 100%.
```

---

### Group of orthologs #256. Best score 613 bits Score difference with first non-orthologous sequence - AM1\_locus\_tags.txt:391 OB3b\_locus\_tags.txt:398

```
META1_0379          	100.00%		CQW49_RS17745       	100.00%
Bootstrap support for META1_0379 as seed ortholog is 100%.
Bootstrap support for CQW49_RS17745 as seed ortholog is 100%.
```

---

### Group of orthologs #257. Best score 608 bits Score difference with first non-orthologous sequence - AM1\_locus\_tags.txt:608 OB3b\_locus\_tags.txt:608

```
META1_1535          	100.00%		CQW49_RS06785       	100.00%
Bootstrap support for META1_1535 as seed ortholog is 100%.
Bootstrap support for CQW49_RS06785 as seed ortholog is 100%.
```

---

### Group of orthologs #258. Best score 605 bits Score difference with first non-orthologous sequence - AM1\_locus\_tags.txt:542 OB3b\_locus\_tags.txt:605

```
META1_3023          	100.00%		CQW49_RS01650       	100.00%
Bootstrap support for META1_3023 as seed ortholog is 100%.
Bootstrap support for CQW49_RS01650 as seed ortholog is 100%.
```

---

### Group of orthologs #259. Best score 604 bits Score difference with first non-orthologous sequence - AM1\_locus\_tags.txt:604 OB3b\_locus\_tags.txt:604

```
META1_4621          	100.00%		CQW49_RS03020       	100.00%
Bootstrap support for META1_4621 as seed ortholog is 100%.
Bootstrap support for CQW49_RS03020 as seed ortholog is 100%.
```

---

### Group of orthologs #260. Best score 601 bits Score difference with first non-orthologous sequence - AM1\_locus\_tags.txt:399 OB3b\_locus\_tags.txt:601

```
META1_5083          	100.00%		CQW49_RS12090       	100.00%
                    	       		CQW49_RS14840       	93.74%
Bootstrap support for META1_5083 as seed ortholog is 100%.
Bootstrap support for CQW49_RS12090 as seed ortholog is 100%.
```

---

### Group of orthologs #261. Best score 600 bits Score difference with first non-orthologous sequence - AM1\_locus\_tags.txt:396 OB3b\_locus\_tags.txt:531

```
META1_1542          	100.00%		CQW49_RS12150       	100.00%
Bootstrap support for META1_1542 as seed ortholog is 100%.
Bootstrap support for CQW49_RS12150 as seed ortholog is 100%.
```

---

### Group of orthologs #262. Best score 598 bits Score difference with first non-orthologous sequence - AM1\_locus\_tags.txt:598 OB3b\_locus\_tags.txt:598

```
META1_0732          	100.00%		CQW49_RS02245       	100.00%
Bootstrap support for META1_0732 as seed ortholog is 100%.
Bootstrap support for CQW49_RS02245 as seed ortholog is 100%.
```

---

### Group of orthologs #263. Best score 598 bits Score difference with first non-orthologous sequence - AM1\_locus\_tags.txt:598 OB3b\_locus\_tags.txt:598

```
META1_3192          	100.00%		CQW49_RS02260       	100.00%
Bootstrap support for META1_3192 as seed ortholog is 100%.
Bootstrap support for CQW49_RS02260 as seed ortholog is 100%.
```

---

### Group of orthologs #264. Best score 597 bits Score difference with first non-orthologous sequence - AM1\_locus\_tags.txt:140 OB3b\_locus\_tags.txt:466

```
META1_2941          	100.00%		CQW49_RS01150       	100.00%
Bootstrap support for META1_2941 as seed ortholog is 99%.
Bootstrap support for CQW49_RS01150 as seed ortholog is 100%.
```

---

### Group of orthologs #265. Best score 597 bits Score difference with first non-orthologous sequence - AM1\_locus\_tags.txt:379 OB3b\_locus\_tags.txt:597

```
META1_4570          	100.00%		CQW49_RS07080       	100.00%
Bootstrap support for META1_4570 as seed ortholog is 100%.
Bootstrap support for CQW49_RS07080 as seed ortholog is 100%.
```

---

### Group of orthologs #266. Best score 597 bits Score difference with first non-orthologous sequence - AM1\_locus\_tags.txt:597 OB3b\_locus\_tags.txt:597

```
META1_2288          	100.00%		CQW49_RS19635       	100.00%
Bootstrap support for META1_2288 as seed ortholog is 100%.
Bootstrap support for CQW49_RS19635 as seed ortholog is 100%.
```

---

### Group of orthologs #267. Best score 593 bits Score difference with first non-orthologous sequence - AM1\_locus\_tags.txt:433 OB3b\_locus\_tags.txt:593

```
META1_2849          	100.00%		CQW49_RS02730       	100.00%
Bootstrap support for META1_2849 as seed ortholog is 100%.
Bootstrap support for CQW49_RS02730 as seed ortholog is 100%.
```

---

### Group of orthologs #268. Best score 589 bits Score difference with first non-orthologous sequence - AM1\_locus\_tags.txt:589 OB3b\_locus\_tags.txt:589

```
META1_4433          	100.00%		CQW49_RS06595       	100.00%
Bootstrap support for META1_4433 as seed ortholog is 100%.
Bootstrap support for CQW49_RS06595 as seed ortholog is 100%.
```

---

### Group of orthologs #269. Best score 588 bits Score difference with first non-orthologous sequence - AM1\_locus\_tags.txt:588 OB3b\_locus\_tags.txt:588

```
META1_2378          	100.00%		CQW49_RS20750       	100.00%
Bootstrap support for META1_2378 as seed ortholog is 100%.
Bootstrap support for CQW49_RS20750 as seed ortholog is 100%.
```

---

### Group of orthologs #270. Best score 587 bits Score difference with first non-orthologous sequence - AM1\_locus\_tags.txt:587 OB3b\_locus\_tags.txt:249

```
META1_3700          	100.00%		CQW49_RS17805       	100.00%
Bootstrap support for META1_3700 as seed ortholog is 100%.
Bootstrap support for CQW49_RS17805 as seed ortholog is 100%.
```

---

### Group of orthologs #271. Best score 586 bits Score difference with first non-orthologous sequence - AM1\_locus\_tags.txt:354 OB3b\_locus\_tags.txt:505

```
META1_0239          	100.00%		CQW49_RS06285       	100.00%
Bootstrap support for META1_0239 as seed ortholog is 100%.
Bootstrap support for CQW49_RS06285 as seed ortholog is 100%.
```

---

### Group of orthologs #272. Best score 586 bits Score difference with first non-orthologous sequence - AM1\_locus\_tags.txt:586 OB3b\_locus\_tags.txt:586

```
META1_2962          	100.00%		CQW49_RS21010       	100.00%
Bootstrap support for META1_2962 as seed ortholog is 100%.
Bootstrap support for CQW49_RS21010 as seed ortholog is 100%.
```

---

### Group of orthologs #273. Best score 583 bits Score difference with first non-orthologous sequence - AM1\_locus\_tags.txt:583 OB3b\_locus\_tags.txt:583

```
META1_2438          	100.00%		CQW49_RS09395       	100.00%
Bootstrap support for META1_2438 as seed ortholog is 100%.
Bootstrap support for CQW49_RS09395 as seed ortholog is 100%.
```

---

### Group of orthologs #274. Best score 583 bits Score difference with first non-orthologous sequence - AM1\_locus\_tags.txt:583 OB3b\_locus\_tags.txt:481

```
META1_4365          	100.00%		CQW49_RS01400       	100.00%
Bootstrap support for META1_4365 as seed ortholog is 100%.
Bootstrap support for CQW49_RS01400 as seed ortholog is 100%.
```

---

### Group of orthologs #275. Best score 583 bits Score difference with first non-orthologous sequence - AM1\_locus\_tags.txt:79 OB3b\_locus\_tags.txt:583

```
META1_2857          	100.00%		CQW49_RS13280       	100.00%
Bootstrap support for META1_2857 as seed ortholog is 98%.
Bootstrap support for CQW49_RS13280 as seed ortholog is 100%.
```

---

### Group of orthologs #276. Best score 582 bits Score difference with first non-orthologous sequence - AM1\_locus\_tags.txt:319 OB3b\_locus\_tags.txt:302

```
META1_5076          	100.00%		CQW49_RS23550       	100.00%
                    	       		CQW49_RS23560       	42.38%
                    	       		CQW49_RS23570       	23.48%
Bootstrap support for META1_5076 as seed ortholog is 100%.
Bootstrap support for CQW49_RS23550 as seed ortholog is 100%.
```

---

### Group of orthologs #277. Best score 582 bits Score difference with first non-orthologous sequence - AM1\_locus\_tags.txt:582 OB3b\_locus\_tags.txt:582

```
META1_4913          	100.00%		CQW49_RS12625       	100.00%
Bootstrap support for META1_4913 as seed ortholog is 100%.
Bootstrap support for CQW49_RS12625 as seed ortholog is 100%.
```

---

### Group of orthologs #278. Best score 580 bits Score difference with first non-orthologous sequence - AM1\_locus\_tags.txt:441 OB3b\_locus\_tags.txt:514

```
META1_0362          	100.00%		CQW49_RS20480       	100.00%
Bootstrap support for META1_0362 as seed ortholog is 100%.
Bootstrap support for CQW49_RS20480 as seed ortholog is 100%.
```

---

### Group of orthologs #279. Best score 579 bits Score difference with first non-orthologous sequence - AM1\_locus\_tags.txt:579 OB3b\_locus\_tags.txt:484

```
META1_4211          	100.00%		CQW49_RS10785       	100.00%
Bootstrap support for META1_4211 as seed ortholog is 100%.
Bootstrap support for CQW49_RS10785 as seed ortholog is 100%.
```

---

### Group of orthologs #280. Best score 579 bits Score difference with first non-orthologous sequence - AM1\_locus\_tags.txt:579 OB3b\_locus\_tags.txt:579

```
META1_3197          	100.00%		CQW49_RS19520       	100.00%
Bootstrap support for META1_3197 as seed ortholog is 100%.
Bootstrap support for CQW49_RS19520 as seed ortholog is 100%.
```

---

### Group of orthologs #281. Best score 579 bits Score difference with first non-orthologous sequence - AM1\_locus\_tags.txt:579 OB3b\_locus\_tags.txt:579

```
META1_3322          	100.00%		CQW49_RS19005       	100.00%
Bootstrap support for META1_3322 as seed ortholog is 100%.
Bootstrap support for CQW49_RS19005 as seed ortholog is 100%.
```

---

### Group of orthologs #282. Best score 578 bits Score difference with first non-orthologous sequence - AM1\_locus\_tags.txt:578 OB3b\_locus\_tags.txt:578

```
META1_0175          	100.00%		CQW49_RS06810       	100.00%
Bootstrap support for META1_0175 as seed ortholog is 100%.
Bootstrap support for CQW49_RS06810 as seed ortholog is 100%.
```

---

### Group of orthologs #283. Best score 578 bits Score difference with first non-orthologous sequence - AM1\_locus\_tags.txt:350 OB3b\_locus\_tags.txt:491

```
META1_4370          	100.00%		CQW49_RS20670       	100.00%
Bootstrap support for META1_4370 as seed ortholog is 100%.
Bootstrap support for CQW49_RS20670 as seed ortholog is 100%.
```

---

### Group of orthologs #284. Best score 576 bits Score difference with first non-orthologous sequence - AM1\_locus\_tags.txt:487 OB3b\_locus\_tags.txt:576

```
META1_2027          	100.00%		CQW49_RS06705       	100.00%
Bootstrap support for META1_2027 as seed ortholog is 100%.
Bootstrap support for CQW49_RS06705 as seed ortholog is 100%.
```

---

### Group of orthologs #285. Best score 576 bits Score difference with first non-orthologous sequence - AM1\_locus\_tags.txt:576 OB3b\_locus\_tags.txt:576

```
META1_0671          	100.00%		CQW49_RS17600       	100.00%
Bootstrap support for META1_0671 as seed ortholog is 100%.
Bootstrap support for CQW49_RS17600 as seed ortholog is 100%.
```

---

### Group of orthologs #286. Best score 576 bits Score difference with first non-orthologous sequence - AM1\_locus\_tags.txt:576 OB3b\_locus\_tags.txt:576

```
META1_2721          	100.00%		CQW49_RS11140       	100.00%
Bootstrap support for META1_2721 as seed ortholog is 100%.
Bootstrap support for CQW49_RS11140 as seed ortholog is 100%.
```

---

### Group of orthologs #287. Best score 575 bits Score difference with first non-orthologous sequence - AM1\_locus\_tags.txt:575 OB3b\_locus\_tags.txt:575

```
META1_3521          	100.00%		CQW49_RS04855       	100.00%
Bootstrap support for META1_3521 as seed ortholog is 100%.
Bootstrap support for CQW49_RS04855 as seed ortholog is 100%.
```

---

### Group of orthologs #288. Best score 575 bits Score difference with first non-orthologous sequence - AM1\_locus\_tags.txt:575 OB3b\_locus\_tags.txt:575

```
META1_2167          	100.00%		CQW49_RS12570       	100.00%
Bootstrap support for META1_2167 as seed ortholog is 100%.
Bootstrap support for CQW49_RS12570 as seed ortholog is 100%.
```

---

### Group of orthologs #289. Best score 575 bits Score difference with first non-orthologous sequence - AM1\_locus\_tags.txt:575 OB3b\_locus\_tags.txt:575

```
META1_3690          	100.00%		CQW49_RS17165       	100.00%
Bootstrap support for META1_3690 as seed ortholog is 100%.
Bootstrap support for CQW49_RS17165 as seed ortholog is 100%.
```

---

### Group of orthologs #290. Best score 575 bits Score difference with first non-orthologous sequence - AM1\_locus\_tags.txt:575 OB3b\_locus\_tags.txt:575

```
META1_5245          	100.00%		CQW49_RS10920       	100.00%
Bootstrap support for META1_5245 as seed ortholog is 100%.
Bootstrap support for CQW49_RS10920 as seed ortholog is 100%.
```

---

### Group of orthologs #291. Best score 574 bits Score difference with first non-orthologous sequence - AM1\_locus\_tags.txt:574 OB3b\_locus\_tags.txt:574

```
META1_3072          	100.00%		CQW49_RS15710       	100.00%
Bootstrap support for META1_3072 as seed ortholog is 100%.
Bootstrap support for CQW49_RS15710 as seed ortholog is 100%.
```

---

### Group of orthologs #292. Best score 573 bits Score difference with first non-orthologous sequence - AM1\_locus\_tags.txt:573 OB3b\_locus\_tags.txt:573

```
META1_4295          	100.00%		CQW49_RS16250       	100.00%
Bootstrap support for META1_4295 as seed ortholog is 100%.
Bootstrap support for CQW49_RS16250 as seed ortholog is 100%.
```

---

### Group of orthologs #293. Best score 572 bits Score difference with first non-orthologous sequence - AM1\_locus\_tags.txt:572 OB3b\_locus\_tags.txt:572

```
META1_4334          	100.00%		CQW49_RS06330       	100.00%
META1_2091          	32.30%		
Bootstrap support for META1_4334 as seed ortholog is 100%.
Bootstrap support for CQW49_RS06330 as seed ortholog is 100%.
```

---

### Group of orthologs #294. Best score 572 bits Score difference with first non-orthologous sequence - AM1\_locus\_tags.txt:572 OB3b\_locus\_tags.txt:572

```
META1_4627          	100.00%		CQW49_RS02200       	100.00%
Bootstrap support for META1_4627 as seed ortholog is 100%.
Bootstrap support for CQW49_RS02200 as seed ortholog is 100%.
```

---

### Group of orthologs #295. Best score 571 bits Score difference with first non-orthologous sequence - AM1\_locus\_tags.txt:571 OB3b\_locus\_tags.txt:571

```
META1_3465          	100.00%		CQW49_RS10025       	100.00%
Bootstrap support for META1_3465 as seed ortholog is 100%.
Bootstrap support for CQW49_RS10025 as seed ortholog is 100%.
```

---

### Group of orthologs #296. Best score 571 bits Score difference with first non-orthologous sequence - AM1\_locus\_tags.txt:571 OB3b\_locus\_tags.txt:571

```
META1_5200          	100.00%		CQW49_RS16455       	100.00%
Bootstrap support for META1_5200 as seed ortholog is 100%.
Bootstrap support for CQW49_RS16455 as seed ortholog is 100%.
```

---

### Group of orthologs #297. Best score 570 bits Score difference with first non-orthologous sequence - AM1\_locus\_tags.txt:570 OB3b\_locus\_tags.txt:570

```
META1_1961          	100.00%		CQW49_RS00600       	100.00%
Bootstrap support for META1_1961 as seed ortholog is 100%.
Bootstrap support for CQW49_RS00600 as seed ortholog is 100%.
```

---

### Group of orthologs #298. Best score 570 bits Score difference with first non-orthologous sequence - AM1\_locus\_tags.txt:570 OB3b\_locus\_tags.txt:570

```
META1_5249          	100.00%		CQW49_RS10960       	100.00%
Bootstrap support for META1_5249 as seed ortholog is 100%.
Bootstrap support for CQW49_RS10960 as seed ortholog is 100%.
```

---

### Group of orthologs #299. Best score 569 bits Score difference with first non-orthologous sequence - AM1\_locus\_tags.txt:569 OB3b\_locus\_tags.txt:569

```
META1_2118          	100.00%		CQW49_RS09945       	100.00%
Bootstrap support for META1_2118 as seed ortholog is 100%.
Bootstrap support for CQW49_RS09945 as seed ortholog is 100%.
```

---

### Group of orthologs #300. Best score 569 bits Score difference with first non-orthologous sequence - AM1\_locus\_tags.txt:188 OB3b\_locus\_tags.txt:569

```
META1_1726          	100.00%		CQW49_RS12235       	100.00%
Bootstrap support for META1_1726 as seed ortholog is 100%.
Bootstrap support for CQW49_RS12235 as seed ortholog is 100%.
```

---

### Group of orthologs #301. Best score 565 bits Score difference with first non-orthologous sequence - AM1\_locus\_tags.txt:565 OB3b\_locus\_tags.txt:565

```
META1_1534          	100.00%		CQW49_RS02905       	100.00%
Bootstrap support for META1_1534 as seed ortholog is 100%.
Bootstrap support for CQW49_RS02905 as seed ortholog is 100%.
```

---

### Group of orthologs #302. Best score 564 bits Score difference with first non-orthologous sequence - AM1\_locus\_tags.txt:430 OB3b\_locus\_tags.txt:564

```
META1_4677          	100.00%		CQW49_RS04920       	100.00%
Bootstrap support for META1_4677 as seed ortholog is 100%.
Bootstrap support for CQW49_RS04920 as seed ortholog is 100%.
```

---

### Group of orthologs #303. Best score 561 bits Score difference with first non-orthologous sequence - AM1\_locus\_tags.txt:561 OB3b\_locus\_tags.txt:561

```
META1_0001          	100.00%		CQW49_RS00005       	100.00%
Bootstrap support for META1_0001 as seed ortholog is 100%.
Bootstrap support for CQW49_RS00005 as seed ortholog is 100%.
```

---

### Group of orthologs #304. Best score 561 bits Score difference with first non-orthologous sequence - AM1\_locus\_tags.txt:561 OB3b\_locus\_tags.txt:561

```
META1_2510          	100.00%		CQW49_RS00055       	100.00%
Bootstrap support for META1_2510 as seed ortholog is 100%.
Bootstrap support for CQW49_RS00055 as seed ortholog is 100%.
```

---

### Group of orthologs #305. Best score 560 bits Score difference with first non-orthologous sequence - AM1\_locus\_tags.txt:487 OB3b\_locus\_tags.txt:560

```
META1_1689          	100.00%		CQW49_RS17060       	100.00%
Bootstrap support for META1_1689 as seed ortholog is 100%.
Bootstrap support for CQW49_RS17060 as seed ortholog is 100%.
```

---

### Group of orthologs #306. Best score 560 bits Score difference with first non-orthologous sequence - AM1\_locus\_tags.txt:560 OB3b\_locus\_tags.txt:560

```
META1_5208          	100.00%		CQW49_RS09030       	100.00%
Bootstrap support for META1_5208 as seed ortholog is 100%.
Bootstrap support for CQW49_RS09030 as seed ortholog is 100%.
```

---

### Group of orthologs #307. Best score 559 bits Score difference with first non-orthologous sequence - AM1\_locus\_tags.txt:559 OB3b\_locus\_tags.txt:559

```
META1_3441          	100.00%		CQW49_RS09055       	100.00%
Bootstrap support for META1_3441 as seed ortholog is 100%.
Bootstrap support for CQW49_RS09055 as seed ortholog is 100%.
```

---

### Group of orthologs #308. Best score 559 bits Score difference with first non-orthologous sequence - AM1\_locus\_tags.txt:559 OB3b\_locus\_tags.txt:559

```
META1_5174          	100.00%		CQW49_RS02450       	100.00%
Bootstrap support for META1_5174 as seed ortholog is 100%.
Bootstrap support for CQW49_RS02450 as seed ortholog is 100%.
```

---

### Group of orthologs #309. Best score 557 bits Score difference with first non-orthologous sequence - AM1\_locus\_tags.txt:557 OB3b\_locus\_tags.txt:557

```
META1_2050          	100.00%		CQW49_RS02855       	100.00%
Bootstrap support for META1_2050 as seed ortholog is 100%.
Bootstrap support for CQW49_RS02855 as seed ortholog is 100%.
```

---

### Group of orthologs #310. Best score 557 bits Score difference with first non-orthologous sequence - AM1\_locus\_tags.txt:557 OB3b\_locus\_tags.txt:557

```
META1_2466          	100.00%		CQW49_RS13315       	100.00%
Bootstrap support for META1_2466 as seed ortholog is 100%.
Bootstrap support for CQW49_RS13315 as seed ortholog is 100%.
```

---

### Group of orthologs #311. Best score 554 bits Score difference with first non-orthologous sequence - AM1\_locus\_tags.txt:481 OB3b\_locus\_tags.txt:554

```
META1_1785          	100.00%		CQW49_RS02145       	100.00%
Bootstrap support for META1_1785 as seed ortholog is 100%.
Bootstrap support for CQW49_RS02145 as seed ortholog is 100%.
```

---

### Group of orthologs #312. Best score 554 bits Score difference with first non-orthologous sequence - AM1\_locus\_tags.txt:491 OB3b\_locus\_tags.txt:554

```
META1_4672          	100.00%		CQW49_RS02965       	100.00%
Bootstrap support for META1_4672 as seed ortholog is 100%.
Bootstrap support for CQW49_RS02965 as seed ortholog is 100%.
```

---

### Group of orthologs #313. Best score 554 bits Score difference with first non-orthologous sequence - AM1\_locus\_tags.txt:247 OB3b\_locus\_tags.txt:473

```
META1_3012          	100.00%		CQW49_RS15935       	100.00%
Bootstrap support for META1_3012 as seed ortholog is 100%.
Bootstrap support for CQW49_RS15935 as seed ortholog is 100%.
```

---

### Group of orthologs #314. Best score 554 bits Score difference with first non-orthologous sequence - AM1\_locus\_tags.txt:554 OB3b\_locus\_tags.txt:554

```
META1_2896          	100.00%		CQW49_RS17825       	100.00%
Bootstrap support for META1_2896 as seed ortholog is 100%.
Bootstrap support for CQW49_RS17825 as seed ortholog is 100%.
```

---

### Group of orthologs #315. Best score 553 bits Score difference with first non-orthologous sequence - AM1\_locus\_tags.txt:362 OB3b\_locus\_tags.txt:553

```
META1_1718          	100.00%		CQW49_RS10745       	100.00%
Bootstrap support for META1_1718 as seed ortholog is 100%.
Bootstrap support for CQW49_RS10745 as seed ortholog is 100%.
```

---

### Group of orthologs #316. Best score 552 bits Score difference with first non-orthologous sequence - AM1\_locus\_tags.txt:423 OB3b\_locus\_tags.txt:508

```
META2_0097          	100.00%		CQW49_RS10895       	100.00%
Bootstrap support for META2_0097 as seed ortholog is 100%.
Bootstrap support for CQW49_RS10895 as seed ortholog is 100%.
```

---

### Group of orthologs #317. Best score 552 bits Score difference with first non-orthologous sequence - AM1\_locus\_tags.txt:91 OB3b\_locus\_tags.txt:552

```
META1_5225          	100.00%		CQW49_RS20060       	100.00%
Bootstrap support for META1_5225 as seed ortholog is 99%.
Bootstrap support for CQW49_RS20060 as seed ortholog is 100%.
```

---

### Group of orthologs #318. Best score 552 bits Score difference with first non-orthologous sequence - AM1\_locus\_tags.txt:439 OB3b\_locus\_tags.txt:552

```
META2_1131          	100.00%		CQW49_RS17190       	100.00%
Bootstrap support for META2_1131 as seed ortholog is 100%.
Bootstrap support for CQW49_RS17190 as seed ortholog is 100%.
```

---

### Group of orthologs #319. Best score 551 bits Score difference with first non-orthologous sequence - AM1\_locus\_tags.txt:551 OB3b\_locus\_tags.txt:551

```
META1_3285          	100.00%		CQW49_RS01510       	100.00%
Bootstrap support for META1_3285 as seed ortholog is 100%.
Bootstrap support for CQW49_RS01510 as seed ortholog is 100%.
```

---

### Group of orthologs #320. Best score 551 bits Score difference with first non-orthologous sequence - AM1\_locus\_tags.txt:434 OB3b\_locus\_tags.txt:551

```
META1_3165          	100.00%		CQW49_RS20630       	100.00%
Bootstrap support for META1_3165 as seed ortholog is 100%.
Bootstrap support for CQW49_RS20630 as seed ortholog is 100%.
```

---

### Group of orthologs #321. Best score 549 bits Score difference with first non-orthologous sequence - AM1\_locus\_tags.txt:549 OB3b\_locus\_tags.txt:549

```
META1_2370          	100.00%		CQW49_RS18455       	100.00%
Bootstrap support for META1_2370 as seed ortholog is 100%.
Bootstrap support for CQW49_RS18455 as seed ortholog is 100%.
```

---

### Group of orthologs #322. Best score 547 bits Score difference with first non-orthologous sequence - AM1\_locus\_tags.txt:450 OB3b\_locus\_tags.txt:444

```
META1_0842          	100.00%		CQW49_RS05290       	100.00%
Bootstrap support for META1_0842 as seed ortholog is 100%.
Bootstrap support for CQW49_RS05290 as seed ortholog is 100%.
```

---

### Group of orthologs #323. Best score 547 bits Score difference with first non-orthologous sequence - AM1\_locus\_tags.txt:422 OB3b\_locus\_tags.txt:547

```
META1_2481          	100.00%		CQW49_RS06100       	100.00%
Bootstrap support for META1_2481 as seed ortholog is 100%.
Bootstrap support for CQW49_RS06100 as seed ortholog is 100%.
```

---

### Group of orthologs #324. Best score 545 bits Score difference with first non-orthologous sequence - AM1\_locus\_tags.txt:423 OB3b\_locus\_tags.txt:354

```
META1_1788          	100.00%		CQW49_RS04700       	100.00%
Bootstrap support for META1_1788 as seed ortholog is 100%.
Bootstrap support for CQW49_RS04700 as seed ortholog is 100%.
```

---

### Group of orthologs #325. Best score 544 bits Score difference with first non-orthologous sequence - AM1\_locus\_tags.txt:487 OB3b\_locus\_tags.txt:473

```
META1_3364          	100.00%		CQW49_RS04355       	100.00%
Bootstrap support for META1_3364 as seed ortholog is 100%.
Bootstrap support for CQW49_RS04355 as seed ortholog is 100%.
```

---

### Group of orthologs #326. Best score 544 bits Score difference with first non-orthologous sequence - AM1\_locus\_tags.txt:452 OB3b\_locus\_tags.txt:544

```
META1_4933          	100.00%		CQW49_RS20170       	100.00%
Bootstrap support for META1_4933 as seed ortholog is 100%.
Bootstrap support for CQW49_RS20170 as seed ortholog is 100%.
```

---

### Group of orthologs #327. Best score 543 bits Score difference with first non-orthologous sequence - AM1\_locus\_tags.txt:421 OB3b\_locus\_tags.txt:543

```
META1_0237          	100.00%		CQW49_RS06105       	100.00%
Bootstrap support for META1_0237 as seed ortholog is 100%.
Bootstrap support for CQW49_RS06105 as seed ortholog is 100%.
```

---

### Group of orthologs #328. Best score 543 bits Score difference with first non-orthologous sequence - AM1\_locus\_tags.txt:543 OB3b\_locus\_tags.txt:543

```
META1_5084          	100.00%		CQW49_RS11250       	100.00%
Bootstrap support for META1_5084 as seed ortholog is 100%.
Bootstrap support for CQW49_RS11250 as seed ortholog is 100%.
```

---

### Group of orthologs #329. Best score 540 bits Score difference with first non-orthologous sequence - AM1\_locus\_tags.txt:540 OB3b\_locus\_tags.txt:540

```
META1_0440          	100.00%		CQW49_RS11990       	100.00%
Bootstrap support for META1_0440 as seed ortholog is 100%.
Bootstrap support for CQW49_RS11990 as seed ortholog is 100%.
```

---

### Group of orthologs #330. Best score 540 bits Score difference with first non-orthologous sequence - AM1\_locus\_tags.txt:540 OB3b\_locus\_tags.txt:540

```
META1_3415          	100.00%		CQW49_RS09980       	100.00%
Bootstrap support for META1_3415 as seed ortholog is 100%.
Bootstrap support for CQW49_RS09980 as seed ortholog is 100%.
```

---

### Group of orthologs #331. Best score 539 bits Score difference with first non-orthologous sequence - AM1\_locus\_tags.txt:357 OB3b\_locus\_tags.txt:539

```
META1_3402          	100.00%		CQW49_RS13430       	100.00%
Bootstrap support for META1_3402 as seed ortholog is 100%.
Bootstrap support for CQW49_RS13430 as seed ortholog is 100%.
```

---

### Group of orthologs #332. Best score 536 bits Score difference with first non-orthologous sequence - AM1\_locus\_tags.txt:536 OB3b\_locus\_tags.txt:421

```
META1_0955          	100.00%		CQW49_RS22070       	100.00%
Bootstrap support for META1_0955 as seed ortholog is 100%.
Bootstrap support for CQW49_RS22070 as seed ortholog is 100%.
```

---

### Group of orthologs #333. Best score 535 bits Score difference with first non-orthologous sequence - AM1\_locus\_tags.txt:196 OB3b\_locus\_tags.txt:391

```
META1_0507          	100.00%		CQW49_RS18770       	100.00%
Bootstrap support for META1_0507 as seed ortholog is 100%.
Bootstrap support for CQW49_RS18770 as seed ortholog is 100%.
```

---

### Group of orthologs #334. Best score 535 bits Score difference with first non-orthologous sequence - AM1\_locus\_tags.txt:535 OB3b\_locus\_tags.txt:535

```
META1_3908          	100.00%		CQW49_RS05065       	100.00%
Bootstrap support for META1_3908 as seed ortholog is 100%.
Bootstrap support for CQW49_RS05065 as seed ortholog is 100%.
```

---

### Group of orthologs #335. Best score 535 bits Score difference with first non-orthologous sequence - AM1\_locus\_tags.txt:387 OB3b\_locus\_tags.txt:300

```
META1_4464          	100.00%		CQW49_RS13095       	100.00%
Bootstrap support for META1_4464 as seed ortholog is 100%.
Bootstrap support for CQW49_RS13095 as seed ortholog is 100%.
```

---

### Group of orthologs #336. Best score 534 bits Score difference with first non-orthologous sequence - AM1\_locus\_tags.txt:307 OB3b\_locus\_tags.txt:534

```
META1_0454          	100.00%		CQW49_RS18700       	100.00%
Bootstrap support for META1_0454 as seed ortholog is 100%.
Bootstrap support for CQW49_RS18700 as seed ortholog is 100%.
```

---

### Group of orthologs #337. Best score 534 bits Score difference with first non-orthologous sequence - AM1\_locus\_tags.txt:534 OB3b\_locus\_tags.txt:534

```
META1_2367          	100.00%		CQW49_RS11920       	100.00%
Bootstrap support for META1_2367 as seed ortholog is 100%.
Bootstrap support for CQW49_RS11920 as seed ortholog is 100%.
```

---

### Group of orthologs #338. Best score 534 bits Score difference with first non-orthologous sequence - AM1\_locus\_tags.txt:534 OB3b\_locus\_tags.txt:534

```
META1_1774          	100.00%		CQW49_RS15615       	100.00%
Bootstrap support for META1_1774 as seed ortholog is 100%.
Bootstrap support for CQW49_RS15615 as seed ortholog is 100%.
```

---

### Group of orthologs #339. Best score 534 bits Score difference with first non-orthologous sequence - AM1\_locus\_tags.txt:68 OB3b\_locus\_tags.txt:534

```
META1_1968          	100.00%		CQW49_RS19240       	100.00%
Bootstrap support for META1_1968 as seed ortholog is 98%.
Bootstrap support for CQW49_RS19240 as seed ortholog is 100%.
```

---

### Group of orthologs #340. Best score 533 bits Score difference with first non-orthologous sequence - AM1\_locus\_tags.txt:533 OB3b\_locus\_tags.txt:533

```
META1_3419          	100.00%		CQW49_RS04845       	100.00%
Bootstrap support for META1_3419 as seed ortholog is 100%.
Bootstrap support for CQW49_RS04845 as seed ortholog is 100%.
```

---

### Group of orthologs #341. Best score 533 bits Score difference with first non-orthologous sequence - AM1\_locus\_tags.txt:533 OB3b\_locus\_tags.txt:533

```
META1_1429          	100.00%		CQW49_RS19200       	100.00%
Bootstrap support for META1_1429 as seed ortholog is 100%.
Bootstrap support for CQW49_RS19200 as seed ortholog is 100%.
```

---

### Group of orthologs #342. Best score 533 bits Score difference with first non-orthologous sequence - AM1\_locus\_tags.txt:533 OB3b\_locus\_tags.txt:533

```
META1_4874          	100.00%		CQW49_RS14345       	100.00%
Bootstrap support for META1_4874 as seed ortholog is 100%.
Bootstrap support for CQW49_RS14345 as seed ortholog is 100%.
```

---

### Group of orthologs #343. Best score 532 bits Score difference with first non-orthologous sequence - AM1\_locus\_tags.txt:532 OB3b\_locus\_tags.txt:240

```
META1_1318          	100.00%		CQW49_RS04060       	100.00%
Bootstrap support for META1_1318 as seed ortholog is 100%.
Bootstrap support for CQW49_RS04060 as seed ortholog is 100%.
```

---

### Group of orthologs #344. Best score 532 bits Score difference with first non-orthologous sequence - AM1\_locus\_tags.txt:532 OB3b\_locus\_tags.txt:532

```
META1_3026          	100.00%		CQW49_RS01630       	100.00%
Bootstrap support for META1_3026 as seed ortholog is 100%.
Bootstrap support for CQW49_RS01630 as seed ortholog is 100%.
```

---

### Group of orthologs #345. Best score 532 bits Score difference with first non-orthologous sequence - AM1\_locus\_tags.txt:532 OB3b\_locus\_tags.txt:532

```
META1_1523          	100.00%		CQW49_RS15795       	100.00%
Bootstrap support for META1_1523 as seed ortholog is 100%.
Bootstrap support for CQW49_RS15795 as seed ortholog is 100%.
```

---

### Group of orthologs #346. Best score 531 bits Score difference with first non-orthologous sequence - AM1\_locus\_tags.txt:531 OB3b\_locus\_tags.txt:531

```
META1_1941          	100.00%		CQW49_RS20830       	100.00%
Bootstrap support for META1_1941 as seed ortholog is 100%.
Bootstrap support for CQW49_RS20830 as seed ortholog is 100%.
```

---

### Group of orthologs #347. Best score 530 bits Score difference with first non-orthologous sequence - AM1\_locus\_tags.txt:434 OB3b\_locus\_tags.txt:443

```
META1_3129          	100.00%		CQW49_RS02135       	100.00%
Bootstrap support for META1_3129 as seed ortholog is 100%.
Bootstrap support for CQW49_RS02135 as seed ortholog is 100%.
```

---

### Group of orthologs #348. Best score 530 bits Score difference with first non-orthologous sequence - AM1\_locus\_tags.txt:530 OB3b\_locus\_tags.txt:530

```
META1_2042          	100.00%		CQW49_RS15645       	100.00%
Bootstrap support for META1_2042 as seed ortholog is 100%.
Bootstrap support for CQW49_RS15645 as seed ortholog is 100%.
```

---

### Group of orthologs #349. Best score 529 bits Score difference with first non-orthologous sequence - AM1\_locus\_tags.txt:359 OB3b\_locus\_tags.txt:529

```
META1_0336          	100.00%		CQW49_RS06760       	100.00%
Bootstrap support for META1_0336 as seed ortholog is 100%.
Bootstrap support for CQW49_RS06760 as seed ortholog is 100%.
```

---

### Group of orthologs #350. Best score 529 bits Score difference with first non-orthologous sequence - AM1\_locus\_tags.txt:529 OB3b\_locus\_tags.txt:529

```
META1_0788          	100.00%		CQW49_RS09915       	100.00%
Bootstrap support for META1_0788 as seed ortholog is 100%.
Bootstrap support for CQW49_RS09915 as seed ortholog is 100%.
```

---

### Group of orthologs #351. Best score 529 bits Score difference with first non-orthologous sequence - AM1\_locus\_tags.txt:360 OB3b\_locus\_tags.txt:529

```
META1_2491          	100.00%		CQW49_RS18985       	100.00%
Bootstrap support for META1_2491 as seed ortholog is 100%.
Bootstrap support for CQW49_RS18985 as seed ortholog is 100%.
```

---

### Group of orthologs #352. Best score 528 bits Score difference with first non-orthologous sequence - AM1\_locus\_tags.txt:528 OB3b\_locus\_tags.txt:528

```
META1_1861          	100.00%		CQW49_RS18460       	100.00%
META1_4949          	54.10%		
Bootstrap support for META1_1861 as seed ortholog is 100%.
Bootstrap support for CQW49_RS18460 as seed ortholog is 100%.
```

---

### Group of orthologs #353. Best score 528 bits Score difference with first non-orthologous sequence - AM1\_locus\_tags.txt:528 OB3b\_locus\_tags.txt:528

```
META1_3528          	100.00%		CQW49_RS02150       	100.00%
Bootstrap support for META1_3528 as seed ortholog is 100%.
Bootstrap support for CQW49_RS02150 as seed ortholog is 100%.
```

---

### Group of orthologs #354. Best score 528 bits Score difference with first non-orthologous sequence - AM1\_locus\_tags.txt:528 OB3b\_locus\_tags.txt:404

```
META1_1541          	100.00%		CQW49_RS16430       	100.00%
Bootstrap support for META1_1541 as seed ortholog is 100%.
Bootstrap support for CQW49_RS16430 as seed ortholog is 100%.
```

---

### Group of orthologs #355. Best score 527 bits Score difference with first non-orthologous sequence - AM1\_locus\_tags.txt:309 OB3b\_locus\_tags.txt:527

```
META1_1623          	100.00%		CQW49_RS05735       	100.00%
Bootstrap support for META1_1623 as seed ortholog is 100%.
Bootstrap support for CQW49_RS05735 as seed ortholog is 100%.
```

---

### Group of orthologs #356. Best score 527 bits Score difference with first non-orthologous sequence - AM1\_locus\_tags.txt:527 OB3b\_locus\_tags.txt:527

```
META1_3511          	100.00%		CQW49_RS10310       	100.00%
Bootstrap support for META1_3511 as seed ortholog is 100%.
Bootstrap support for CQW49_RS10310 as seed ortholog is 100%.
```

---

### Group of orthologs #357. Best score 526 bits Score difference with first non-orthologous sequence - AM1\_locus\_tags.txt:526 OB3b\_locus\_tags.txt:526

```
META1_1817          	100.00%		CQW49_RS11765       	100.00%
Bootstrap support for META1_1817 as seed ortholog is 100%.
Bootstrap support for CQW49_RS11765 as seed ortholog is 100%.
```

---

### Group of orthologs #358. Best score 526 bits Score difference with first non-orthologous sequence - AM1\_locus\_tags.txt:526 OB3b\_locus\_tags.txt:526

```
META1_1733          	100.00%		CQW49_RS12270       	100.00%
Bootstrap support for META1_1733 as seed ortholog is 100%.
Bootstrap support for CQW49_RS12270 as seed ortholog is 100%.
```

---

### Group of orthologs #359. Best score 526 bits Score difference with first non-orthologous sequence - AM1\_locus\_tags.txt:526 OB3b\_locus\_tags.txt:526

```
META1_4247          	100.00%		CQW49_RS00610       	100.00%
Bootstrap support for META1_4247 as seed ortholog is 100%.
Bootstrap support for CQW49_RS00610 as seed ortholog is 100%.
```

---

### Group of orthologs #360. Best score 526 bits Score difference with first non-orthologous sequence - AM1\_locus\_tags.txt:526 OB3b\_locus\_tags.txt:526

```
META1_3311          	100.00%		CQW49_RS21020       	100.00%
Bootstrap support for META1_3311 as seed ortholog is 100%.
Bootstrap support for CQW49_RS21020 as seed ortholog is 100%.
```

---

### Group of orthologs #361. Best score 525 bits Score difference with first non-orthologous sequence - AM1\_locus\_tags.txt:426 OB3b\_locus\_tags.txt:433

```
META1_0683          	100.00%		CQW49_RS16565       	100.00%
Bootstrap support for META1_0683 as seed ortholog is 100%.
Bootstrap support for CQW49_RS16565 as seed ortholog is 100%.
```

---

### Group of orthologs #362. Best score 525 bits Score difference with first non-orthologous sequence - AM1\_locus\_tags.txt:383 OB3b\_locus\_tags.txt:385

```
META1_4647          	100.00%		CQW49_RS00185       	100.00%
Bootstrap support for META1_4647 as seed ortholog is 100%.
Bootstrap support for CQW49_RS00185 as seed ortholog is 100%.
```

---

### Group of orthologs #363. Best score 525 bits Score difference with first non-orthologous sequence - AM1\_locus\_tags.txt:525 OB3b\_locus\_tags.txt:525

```
META1_0793          	100.00%		CQW49_RS18920       	100.00%
Bootstrap support for META1_0793 as seed ortholog is 100%.
Bootstrap support for CQW49_RS18920 as seed ortholog is 100%.
```

---

### Group of orthologs #364. Best score 522 bits Score difference with first non-orthologous sequence - AM1\_locus\_tags.txt:522 OB3b\_locus\_tags.txt:397

```
META1_2956          	100.00%		CQW49_RS13115       	100.00%
Bootstrap support for META1_2956 as seed ortholog is 100%.
Bootstrap support for CQW49_RS13115 as seed ortholog is 100%.
```

---

### Group of orthologs #365. Best score 522 bits Score difference with first non-orthologous sequence - AM1\_locus\_tags.txt:522 OB3b\_locus\_tags.txt:522

```
META1_3288          	100.00%		CQW49_RS20530       	100.00%
Bootstrap support for META1_3288 as seed ortholog is 100%.
Bootstrap support for CQW49_RS20530 as seed ortholog is 100%.
```

---

### Group of orthologs #366. Best score 520 bits Score difference with first non-orthologous sequence - AM1\_locus\_tags.txt:520 OB3b\_locus\_tags.txt:520

```
META1_5131          	100.00%		CQW49_RS04730       	100.00%
Bootstrap support for META1_5131 as seed ortholog is 100%.
Bootstrap support for CQW49_RS04730 as seed ortholog is 100%.
```

---

### Group of orthologs #367. Best score 519 bits Score difference with first non-orthologous sequence - AM1\_locus\_tags.txt:519 OB3b\_locus\_tags.txt:519

```
META1_2418          	100.00%		CQW49_RS20890       	100.00%
Bootstrap support for META1_2418 as seed ortholog is 100%.
Bootstrap support for CQW49_RS20890 as seed ortholog is 100%.
```

---

### Group of orthologs #368. Best score 519 bits Score difference with first non-orthologous sequence - AM1\_locus\_tags.txt:519 OB3b\_locus\_tags.txt:471

```
META1_3198          	100.00%		CQW49_RS18150       	100.00%
Bootstrap support for META1_3198 as seed ortholog is 100%.
Bootstrap support for CQW49_RS18150 as seed ortholog is 100%.
```

---

### Group of orthologs #369. Best score 519 bits Score difference with first non-orthologous sequence - AM1\_locus\_tags.txt:427 OB3b\_locus\_tags.txt:79

```
META1_4129          	100.00%		CQW49_RS23515       	100.00%
Bootstrap support for META1_4129 as seed ortholog is 100%.
Bootstrap support for CQW49_RS23515 as seed ortholog is 90%.
```

---

### Group of orthologs #370. Best score 518 bits Score difference with first non-orthologous sequence - AM1\_locus\_tags.txt:518 OB3b\_locus\_tags.txt:518

```
META1_2390          	100.00%		CQW49_RS17505       	100.00%
Bootstrap support for META1_2390 as seed ortholog is 100%.
Bootstrap support for CQW49_RS17505 as seed ortholog is 100%.
```

---

### Group of orthologs #371. Best score 517 bits Score difference with first non-orthologous sequence - AM1\_locus\_tags.txt:427 OB3b\_locus\_tags.txt:517

```
META1_1262          	100.00%		CQW49_RS17495       	100.00%
Bootstrap support for META1_1262 as seed ortholog is 100%.
Bootstrap support for CQW49_RS17495 as seed ortholog is 100%.
```

---

### Group of orthologs #372. Best score 517 bits Score difference with first non-orthologous sequence - AM1\_locus\_tags.txt:517 OB3b\_locus\_tags.txt:517

```
META1_2057          	100.00%		CQW49_RS15700       	100.00%
Bootstrap support for META1_2057 as seed ortholog is 100%.
Bootstrap support for CQW49_RS15700 as seed ortholog is 100%.
```

---

### Group of orthologs #373. Best score 516 bits Score difference with first non-orthologous sequence - AM1\_locus\_tags.txt:335 OB3b\_locus\_tags.txt:437

```
META1_2704          	100.00%		CQW49_RS02805       	100.00%
Bootstrap support for META1_2704 as seed ortholog is 100%.
Bootstrap support for CQW49_RS02805 as seed ortholog is 100%.
```

---

### Group of orthologs #374. Best score 516 bits Score difference with first non-orthologous sequence - AM1\_locus\_tags.txt:516 OB3b\_locus\_tags.txt:516

```
META1_3393          	100.00%		CQW49_RS09150       	100.00%
Bootstrap support for META1_3393 as seed ortholog is 100%.
Bootstrap support for CQW49_RS09150 as seed ortholog is 100%.
```

---

### Group of orthologs #375. Best score 516 bits Score difference with first non-orthologous sequence - AM1\_locus\_tags.txt:72 OB3b\_locus\_tags.txt:516

```
META1_5250          	100.00%		CQW49_RS21175       	100.00%
Bootstrap support for META1_5250 as seed ortholog is 96%.
Bootstrap support for CQW49_RS21175 as seed ortholog is 100%.
```

---

### Group of orthologs #376. Best score 515 bits Score difference with first non-orthologous sequence - AM1\_locus\_tags.txt:515 OB3b\_locus\_tags.txt:515

```
META1_0848          	100.00%		CQW49_RS05320       	100.00%
Bootstrap support for META1_0848 as seed ortholog is 100%.
Bootstrap support for CQW49_RS05320 as seed ortholog is 100%.
```

---

### Group of orthologs #377. Best score 515 bits Score difference with first non-orthologous sequence - AM1\_locus\_tags.txt:396 OB3b\_locus\_tags.txt:515

```
META1_3658          	100.00%		CQW49_RS20200       	100.00%
Bootstrap support for META1_3658 as seed ortholog is 100%.
Bootstrap support for CQW49_RS20200 as seed ortholog is 100%.
```

---

### Group of orthologs #378. Best score 514 bits Score difference with first non-orthologous sequence - AM1\_locus\_tags.txt:514 OB3b\_locus\_tags.txt:514

```
META1_1321          	100.00%		CQW49_RS04080       	100.00%
Bootstrap support for META1_1321 as seed ortholog is 100%.
Bootstrap support for CQW49_RS04080 as seed ortholog is 100%.
```

---

### Group of orthologs #379. Best score 514 bits Score difference with first non-orthologous sequence - AM1\_locus\_tags.txt:514 OB3b\_locus\_tags.txt:514

```
META1_0204          	100.00%		CQW49_RS12750       	100.00%
Bootstrap support for META1_0204 as seed ortholog is 100%.
Bootstrap support for CQW49_RS12750 as seed ortholog is 100%.
```

---

### Group of orthologs #380. Best score 514 bits Score difference with first non-orthologous sequence - AM1\_locus\_tags.txt:514 OB3b\_locus\_tags.txt:514

```
META1_1731          	100.00%		CQW49_RS12260       	100.00%
Bootstrap support for META1_1731 as seed ortholog is 100%.
Bootstrap support for CQW49_RS12260 as seed ortholog is 100%.
```

---

### Group of orthologs #381. Best score 514 bits Score difference with first non-orthologous sequence - AM1\_locus\_tags.txt:342 OB3b\_locus\_tags.txt:514

```
META1_4310          	100.00%		CQW49_RS18485       	100.00%
Bootstrap support for META1_4310 as seed ortholog is 100%.
Bootstrap support for CQW49_RS18485 as seed ortholog is 100%.
```

---

### Group of orthologs #382. Best score 513 bits Score difference with first non-orthologous sequence - AM1\_locus\_tags.txt:513 OB3b\_locus\_tags.txt:513

```
META1_2369          	100.00%		CQW49_RS11910       	100.00%
Bootstrap support for META1_2369 as seed ortholog is 100%.
Bootstrap support for CQW49_RS11910 as seed ortholog is 100%.
```

---

### Group of orthologs #383. Best score 513 bits Score difference with first non-orthologous sequence - AM1\_locus\_tags.txt:139 OB3b\_locus\_tags.txt:513

```
META1_4239          	100.00%		CQW49_RS11525       	100.00%
Bootstrap support for META1_4239 as seed ortholog is 100%.
Bootstrap support for CQW49_RS11525 as seed ortholog is 100%.
```

---

### Group of orthologs #384. Best score 510 bits Score difference with first non-orthologous sequence - AM1\_locus\_tags.txt:510 OB3b\_locus\_tags.txt:121

```
META1_0002          	100.00%		CQW49_RS19600       	100.00%
Bootstrap support for META1_0002 as seed ortholog is 100%.
Bootstrap support for CQW49_RS19600 as seed ortholog is 99%.
```

---

### Group of orthologs #385. Best score 509 bits Score difference with first non-orthologous sequence - AM1\_locus\_tags.txt:509 OB3b\_locus\_tags.txt:509

```
META1_4544          	100.00%		CQW49_RS09145       	100.00%
Bootstrap support for META1_4544 as seed ortholog is 100%.
Bootstrap support for CQW49_RS09145 as seed ortholog is 100%.
```

---

### Group of orthologs #386. Best score 507 bits Score difference with first non-orthologous sequence - AM1\_locus\_tags.txt:507 OB3b\_locus\_tags.txt:507

```
META1_1757          	100.00%		CQW49_RS03840       	100.00%
Bootstrap support for META1_1757 as seed ortholog is 100%.
Bootstrap support for CQW49_RS03840 as seed ortholog is 100%.
```

---

### Group of orthologs #387. Best score 507 bits Score difference with first non-orthologous sequence - AM1\_locus\_tags.txt:507 OB3b\_locus\_tags.txt:507

```
META1_0485          	100.00%		CQW49_RS10870       	100.00%
Bootstrap support for META1_0485 as seed ortholog is 100%.
Bootstrap support for CQW49_RS10870 as seed ortholog is 100%.
```

---

### Group of orthologs #388. Best score 507 bits Score difference with first non-orthologous sequence - AM1\_locus\_tags.txt:507 OB3b\_locus\_tags.txt:507

```
META1_3190          	100.00%		CQW49_RS02170       	100.00%
Bootstrap support for META1_3190 as seed ortholog is 100%.
Bootstrap support for CQW49_RS02170 as seed ortholog is 100%.
```

---

### Group of orthologs #389. Best score 506 bits Score difference with first non-orthologous sequence - AM1\_locus\_tags.txt:506 OB3b\_locus\_tags.txt:506

```
META1_1539          	100.00%		CQW49_RS16420       	100.00%
Bootstrap support for META1_1539 as seed ortholog is 100%.
Bootstrap support for CQW49_RS16420 as seed ortholog is 100%.
```

---

### Group of orthologs #390. Best score 505 bits Score difference with first non-orthologous sequence - AM1\_locus\_tags.txt:385 OB3b\_locus\_tags.txt:505

```
META1_2330          	100.00%		CQW49_RS09070       	100.00%
Bootstrap support for META1_2330 as seed ortholog is 100%.
Bootstrap support for CQW49_RS09070 as seed ortholog is 100%.
```

---

### Group of orthologs #391. Best score 505 bits Score difference with first non-orthologous sequence - AM1\_locus\_tags.txt:52 OB3b\_locus\_tags.txt:141

```
META1_1488          	100.00%		CQW49_RS21955       	100.00%
Bootstrap support for META1_1488 as seed ortholog is 79%.
Bootstrap support for CQW49_RS21955 as seed ortholog is 99%.
```

---

### Group of orthologs #392. Best score 502 bits Score difference with first non-orthologous sequence - AM1\_locus\_tags.txt:409 OB3b\_locus\_tags.txt:502

```
META1_0184          	100.00%		CQW49_RS04345       	100.00%
Bootstrap support for META1_0184 as seed ortholog is 100%.
Bootstrap support for CQW49_RS04345 as seed ortholog is 100%.
```

---

### Group of orthologs #393. Best score 502 bits Score difference with first non-orthologous sequence - AM1\_locus\_tags.txt:502 OB3b\_locus\_tags.txt:502

```
META1_5085          	100.00%		CQW49_RS11245       	100.00%
Bootstrap support for META1_5085 as seed ortholog is 100%.
Bootstrap support for CQW49_RS11245 as seed ortholog is 100%.
```

---

### Group of orthologs #394. Best score 501 bits Score difference with first non-orthologous sequence - AM1\_locus\_tags.txt:331 OB3b\_locus\_tags.txt:501

```
META1_2215          	100.00%		CQW49_RS16445       	100.00%
Bootstrap support for META1_2215 as seed ortholog is 100%.
Bootstrap support for CQW49_RS16445 as seed ortholog is 100%.
```

---

### Group of orthologs #395. Best score 500 bits Score difference with first non-orthologous sequence - AM1\_locus\_tags.txt:500 OB3b\_locus\_tags.txt:500

```
META1_2045          	100.00%		CQW49_RS01380       	100.00%
Bootstrap support for META1_2045 as seed ortholog is 100%.
Bootstrap support for CQW49_RS01380 as seed ortholog is 100%.
```

---

### Group of orthologs #396. Best score 496 bits Score difference with first non-orthologous sequence - AM1\_locus\_tags.txt:322 OB3b\_locus\_tags.txt:496

```
META1_0694          	100.00%		CQW49_RS04365       	100.00%
Bootstrap support for META1_0694 as seed ortholog is 100%.
Bootstrap support for CQW49_RS04365 as seed ortholog is 100%.
```

---

### Group of orthologs #397. Best score 496 bits Score difference with first non-orthologous sequence - AM1\_locus\_tags.txt:496 OB3b\_locus\_tags.txt:496

```
META1_4852          	100.00%		CQW49_RS12745       	100.00%
Bootstrap support for META1_4852 as seed ortholog is 100%.
Bootstrap support for CQW49_RS12745 as seed ortholog is 100%.
```

---

### Group of orthologs #398. Best score 495 bits Score difference with first non-orthologous sequence - AM1\_locus\_tags.txt:495 OB3b\_locus\_tags.txt:495

```
META1_0419          	100.00%		CQW49_RS16715       	100.00%
META1_4597          	6.94%		
Bootstrap support for META1_0419 as seed ortholog is 100%.
Bootstrap support for CQW49_RS16715 as seed ortholog is 100%.
```

---

### Group of orthologs #399. Best score 495 bits Score difference with first non-orthologous sequence - AM1\_locus\_tags.txt:27 OB3b\_locus\_tags.txt:106

```
META1_4418          	100.00%		CQW49_RS12220       	100.00%
Bootstrap support for META1_4418 as seed ortholog is 46%.
Alternative seed ortholog is META1_4899 (27 bits away from this cluster)
Bootstrap support for CQW49_RS12220 as seed ortholog is 99%.
```

---

### Group of orthologs #400. Best score 494 bits Score difference with first non-orthologous sequence - AM1\_locus\_tags.txt:383 OB3b\_locus\_tags.txt:494

```
META1_2244          	100.00%		CQW49_RS02420       	100.00%
Bootstrap support for META1_2244 as seed ortholog is 100%.
Bootstrap support for CQW49_RS02420 as seed ortholog is 100%.
```

---

### Group of orthologs #401. Best score 494 bits Score difference with first non-orthologous sequence - AM1\_locus\_tags.txt:345 OB3b\_locus\_tags.txt:29

```
META1_3818          	100.00%		CQW49_RS20810       	100.00%
Bootstrap support for META1_3818 as seed ortholog is 100%.
Bootstrap support for CQW49_RS20810 as seed ortholog is 37%.
Alternative seed ortholog is CQW49_RS15900 (29 bits away from this cluster)
```

---

### Group of orthologs #402. Best score 493 bits Score difference with first non-orthologous sequence - AM1\_locus\_tags.txt:439 OB3b\_locus\_tags.txt:493

```
META1_2843          	100.00%		CQW49_RS12195       	100.00%
Bootstrap support for META1_2843 as seed ortholog is 100%.
Bootstrap support for CQW49_RS12195 as seed ortholog is 100%.
```

---

### Group of orthologs #403. Best score 493 bits Score difference with first non-orthologous sequence - AM1\_locus\_tags.txt:493 OB3b\_locus\_tags.txt:493

```
META1_3153          	100.00%		CQW49_RS11295       	100.00%
Bootstrap support for META1_3153 as seed ortholog is 100%.
Bootstrap support for CQW49_RS11295 as seed ortholog is 100%.
```

---

### Group of orthologs #404. Best score 493 bits Score difference with first non-orthologous sequence - AM1\_locus\_tags.txt:493 OB3b\_locus\_tags.txt:493

```
META1_1537          	100.00%		CQW49_RS19015       	100.00%
Bootstrap support for META1_1537 as seed ortholog is 100%.
Bootstrap support for CQW49_RS19015 as seed ortholog is 100%.
```

---

### Group of orthologs #405. Best score 493 bits Score difference with first non-orthologous sequence - AM1\_locus\_tags.txt:493 OB3b\_locus\_tags.txt:421

```
META1_3187          	100.00%		CQW49_RS12110       	100.00%
Bootstrap support for META1_3187 as seed ortholog is 100%.
Bootstrap support for CQW49_RS12110 as seed ortholog is 100%.
```

---

### Group of orthologs #406. Best score 491 bits Score difference with first non-orthologous sequence - AM1\_locus\_tags.txt:491 OB3b\_locus\_tags.txt:378

```
META1_0319          	100.00%		CQW49_RS18860       	100.00%
Bootstrap support for META1_0319 as seed ortholog is 100%.
Bootstrap support for CQW49_RS18860 as seed ortholog is 100%.
```

---

### Group of orthologs #407. Best score 491 bits Score difference with first non-orthologous sequence - AM1\_locus\_tags.txt:292 OB3b\_locus\_tags.txt:370

```
META1_0222          	100.00%		CQW49_RS19485       	100.00%
Bootstrap support for META1_0222 as seed ortholog is 100%.
Bootstrap support for CQW49_RS19485 as seed ortholog is 100%.
```

---

### Group of orthologs #408. Best score 489 bits Score difference with first non-orthologous sequence - AM1\_locus\_tags.txt:489 OB3b\_locus\_tags.txt:489

```
META1_4244          	100.00%		CQW49_RS04985       	100.00%
Bootstrap support for META1_4244 as seed ortholog is 100%.
Bootstrap support for CQW49_RS04985 as seed ortholog is 100%.
```

---

### Group of orthologs #409. Best score 488 bits Score difference with first non-orthologous sequence - AM1\_locus\_tags.txt:488 OB3b\_locus\_tags.txt:488

```
META1_3527          	100.00%		CQW49_RS10355       	100.00%
Bootstrap support for META1_3527 as seed ortholog is 100%.
Bootstrap support for CQW49_RS10355 as seed ortholog is 100%.
```

---

### Group of orthologs #410. Best score 485 bits Score difference with first non-orthologous sequence - AM1\_locus\_tags.txt:62 OB3b\_locus\_tags.txt:485

```
META1_0042          	100.00%		CQW49_RS00390       	100.00%
Bootstrap support for META1_0042 as seed ortholog is 91%.
Bootstrap support for CQW49_RS00390 as seed ortholog is 100%.
```

---

### Group of orthologs #411. Best score 485 bits Score difference with first non-orthologous sequence - AM1\_locus\_tags.txt:485 OB3b\_locus\_tags.txt:485

```
META1_3180          	100.00%		CQW49_RS06070       	100.00%
Bootstrap support for META1_3180 as seed ortholog is 100%.
Bootstrap support for CQW49_RS06070 as seed ortholog is 100%.
```

---

### Group of orthologs #412. Best score 485 bits Score difference with first non-orthologous sequence - AM1\_locus\_tags.txt:485 OB3b\_locus\_tags.txt:485

```
META1_4307          	100.00%		CQW49_RS21310       	100.00%
Bootstrap support for META1_4307 as seed ortholog is 100%.
Bootstrap support for CQW49_RS21310 as seed ortholog is 100%.
```

---

### Group of orthologs #413. Best score 482 bits Score difference with first non-orthologous sequence - AM1\_locus\_tags.txt:482 OB3b\_locus\_tags.txt:482

```
META1_3000          	100.00%		CQW49_RS01710       	100.00%
Bootstrap support for META1_3000 as seed ortholog is 100%.
Bootstrap support for CQW49_RS01710 as seed ortholog is 100%.
```

---

### Group of orthologs #414. Best score 482 bits Score difference with first non-orthologous sequence - AM1\_locus\_tags.txt:482 OB3b\_locus\_tags.txt:482

```
META1_5216          	100.00%		CQW49_RS00705       	100.00%
Bootstrap support for META1_5216 as seed ortholog is 100%.
Bootstrap support for CQW49_RS00705 as seed ortholog is 100%.
```

---

### Group of orthologs #415. Best score 482 bits Score difference with first non-orthologous sequence - AM1\_locus\_tags.txt:380 OB3b\_locus\_tags.txt:82

```
META1_3330          	100.00%		CQW49_RS23510       	100.00%
Bootstrap support for META1_3330 as seed ortholog is 100%.
Bootstrap support for CQW49_RS23510 as seed ortholog is 97%.
```

---

### Group of orthologs #416. Best score 481 bits Score difference with first non-orthologous sequence - AM1\_locus\_tags.txt:481 OB3b\_locus\_tags.txt:481

```
META1_0488          	100.00%		CQW49_RS05590       	100.00%
Bootstrap support for META1_0488 as seed ortholog is 100%.
Bootstrap support for CQW49_RS05590 as seed ortholog is 100%.
```

---

### Group of orthologs #417. Best score 478 bits Score difference with first non-orthologous sequence - AM1\_locus\_tags.txt:7 OB3b\_locus\_tags.txt:341

```
META1_2326          	100.00%		CQW49_RS20265       	100.00%
Bootstrap support for META1_2326 as seed ortholog is 63%.
Alternative seed ortholog is META1_3271 (7 bits away from this cluster)
Bootstrap support for CQW49_RS20265 as seed ortholog is 100%.
```

---

### Group of orthologs #418. Best score 477 bits Score difference with first non-orthologous sequence - AM1\_locus\_tags.txt:284 OB3b\_locus\_tags.txt:477

```
META1_1682          	100.00%		CQW49_RS02410       	100.00%
Bootstrap support for META1_1682 as seed ortholog is 100%.
Bootstrap support for CQW49_RS02410 as seed ortholog is 100%.
```

---

### Group of orthologs #419. Best score 477 bits Score difference with first non-orthologous sequence - AM1\_locus\_tags.txt:248 OB3b\_locus\_tags.txt:477

```
META1_2302          	100.00%		CQW49_RS14285       	100.00%
Bootstrap support for META1_2302 as seed ortholog is 100%.
Bootstrap support for CQW49_RS14285 as seed ortholog is 100%.
```

---

### Group of orthologs #420. Best score 477 bits Score difference with first non-orthologous sequence - AM1\_locus\_tags.txt:477 OB3b\_locus\_tags.txt:477

```
META1_2447          	100.00%		CQW49_RS17415       	100.00%
Bootstrap support for META1_2447 as seed ortholog is 100%.
Bootstrap support for CQW49_RS17415 as seed ortholog is 100%.
```

---

### Group of orthologs #421. Best score 476 bits Score difference with first non-orthologous sequence - AM1\_locus\_tags.txt:476 OB3b\_locus\_tags.txt:476

```
META1_3856          	100.00%		CQW49_RS10890       	100.00%
Bootstrap support for META1_3856 as seed ortholog is 100%.
Bootstrap support for CQW49_RS10890 as seed ortholog is 100%.
```

---

### Group of orthologs #422. Best score 476 bits Score difference with first non-orthologous sequence - AM1\_locus\_tags.txt:476 OB3b\_locus\_tags.txt:476

```
META1_4888          	100.00%		CQW49_RS15465       	100.00%
Bootstrap support for META1_4888 as seed ortholog is 100%.
Bootstrap support for CQW49_RS15465 as seed ortholog is 100%.
```

---

### Group of orthologs #423. Best score 475 bits Score difference with first non-orthologous sequence - AM1\_locus\_tags.txt:475 OB3b\_locus\_tags.txt:475

```
META1_2156          	100.00%		CQW49_RS10505       	100.00%
Bootstrap support for META1_2156 as seed ortholog is 100%.
Bootstrap support for CQW49_RS10505 as seed ortholog is 100%.
```

---

### Group of orthologs #424. Best score 475 bits Score difference with first non-orthologous sequence - AM1\_locus\_tags.txt:475 OB3b\_locus\_tags.txt:475

```
META1_5087          	100.00%		CQW49_RS11235       	100.00%
Bootstrap support for META1_5087 as seed ortholog is 100%.
Bootstrap support for CQW49_RS11235 as seed ortholog is 100%.
```

---

### Group of orthologs #425. Best score 475 bits Score difference with first non-orthologous sequence - AM1\_locus\_tags.txt:475 OB3b\_locus\_tags.txt:121

```
META1_5024          	100.00%		CQW49_RS22530       	100.00%
Bootstrap support for META1_5024 as seed ortholog is 100%.
Bootstrap support for CQW49_RS22530 as seed ortholog is 99%.
```

---

### Group of orthologs #426. Best score 474 bits Score difference with first non-orthologous sequence - AM1\_locus\_tags.txt:474 OB3b\_locus\_tags.txt:474

```
META1_0729          	100.00%		CQW49_RS00990       	100.00%
Bootstrap support for META1_0729 as seed ortholog is 100%.
Bootstrap support for CQW49_RS00990 as seed ortholog is 100%.
```

---

### Group of orthologs #427. Best score 474 bits Score difference with first non-orthologous sequence - AM1\_locus\_tags.txt:474 OB3b\_locus\_tags.txt:474

```
META1_1709          	100.00%		CQW49_RS12320       	100.00%
Bootstrap support for META1_1709 as seed ortholog is 100%.
Bootstrap support for CQW49_RS12320 as seed ortholog is 100%.
```

---

### Group of orthologs #428. Best score 474 bits Score difference with first non-orthologous sequence - AM1\_locus\_tags.txt:474 OB3b\_locus\_tags.txt:474

```
META1_4153          	100.00%		CQW49_RS03920       	100.00%
Bootstrap support for META1_4153 as seed ortholog is 100%.
Bootstrap support for CQW49_RS03920 as seed ortholog is 100%.
```

---

### Group of orthologs #429. Best score 474 bits Score difference with first non-orthologous sequence - AM1\_locus\_tags.txt:474 OB3b\_locus\_tags.txt:474

```
META1_3146          	100.00%		CQW49_RS11255       	100.00%
Bootstrap support for META1_3146 as seed ortholog is 100%.
Bootstrap support for CQW49_RS11255 as seed ortholog is 100%.
```

---

### Group of orthologs #430. Best score 473 bits Score difference with first non-orthologous sequence - AM1\_locus\_tags.txt:473 OB3b\_locus\_tags.txt:473

```
META1_0412          	100.00%		CQW49_RS19235       	100.00%
Bootstrap support for META1_0412 as seed ortholog is 100%.
Bootstrap support for CQW49_RS19235 as seed ortholog is 100%.
```

---

### Group of orthologs #431. Best score 473 bits Score difference with first non-orthologous sequence - AM1\_locus\_tags.txt:473 OB3b\_locus\_tags.txt:473

```
META1_1404          	100.00%		CQW49_RS23725       	100.00%
Bootstrap support for META1_1404 as seed ortholog is 100%.
Bootstrap support for CQW49_RS23725 as seed ortholog is 100%.
```

---

### Group of orthologs #432. Best score 472 bits Score difference with first non-orthologous sequence - AM1\_locus\_tags.txt:472 OB3b\_locus\_tags.txt:346

```
META1_2986          	100.00%		CQW49_RS01955       	100.00%
Bootstrap support for META1_2986 as seed ortholog is 100%.
Bootstrap support for CQW49_RS01955 as seed ortholog is 100%.
```

---

### Group of orthologs #433. Best score 472 bits Score difference with first non-orthologous sequence - AM1\_locus\_tags.txt:472 OB3b\_locus\_tags.txt:472

```
META1_3120          	100.00%		CQW49_RS02845       	100.00%
Bootstrap support for META1_3120 as seed ortholog is 100%.
Bootstrap support for CQW49_RS02845 as seed ortholog is 100%.
```

---

### Group of orthologs #434. Best score 471 bits Score difference with first non-orthologous sequence - AM1\_locus\_tags.txt:471 OB3b\_locus\_tags.txt:471

```
META1_3071          	100.00%		CQW49_RS02225       	100.00%
Bootstrap support for META1_3071 as seed ortholog is 100%.
Bootstrap support for CQW49_RS02225 as seed ortholog is 100%.
```

---

### Group of orthologs #435. Best score 470 bits Score difference with first non-orthologous sequence - AM1\_locus\_tags.txt:64 OB3b\_locus\_tags.txt:470

```
META1_3705          	100.00%		CQW49_RS10085       	100.00%
Bootstrap support for META1_3705 as seed ortholog is 96%.
Bootstrap support for CQW49_RS10085 as seed ortholog is 100%.
```

---

### Group of orthologs #436. Best score 469 bits Score difference with first non-orthologous sequence - AM1\_locus\_tags.txt:469 OB3b\_locus\_tags.txt:469

```
META1_1714          	100.00%		CQW49_RS06355       	100.00%
Bootstrap support for META1_1714 as seed ortholog is 100%.
Bootstrap support for CQW49_RS06355 as seed ortholog is 100%.
```

---

### Group of orthologs #437. Best score 468 bits Score difference with first non-orthologous sequence - AM1\_locus\_tags.txt:468 OB3b\_locus\_tags.txt:468

```
META1_0499          	100.00%		CQW49_RS06970       	100.00%
Bootstrap support for META1_0499 as seed ortholog is 100%.
Bootstrap support for CQW49_RS06970 as seed ortholog is 100%.
```

---

### Group of orthologs #438. Best score 467 bits Score difference with first non-orthologous sequence - AM1\_locus\_tags.txt:348 OB3b\_locus\_tags.txt:467

```
META1_2707          	100.00%		CQW49_RS02790       	100.00%
Bootstrap support for META1_2707 as seed ortholog is 100%.
Bootstrap support for CQW49_RS02790 as seed ortholog is 100%.
```

---

### Group of orthologs #439. Best score 467 bits Score difference with first non-orthologous sequence - AM1\_locus\_tags.txt:467 OB3b\_locus\_tags.txt:467

```
META1_4486          	100.00%		CQW49_RS06920       	100.00%
Bootstrap support for META1_4486 as seed ortholog is 100%.
Bootstrap support for CQW49_RS06920 as seed ortholog is 100%.
```

---

### Group of orthologs #440. Best score 466 bits Score difference with first non-orthologous sequence - AM1\_locus\_tags.txt:364 OB3b\_locus\_tags.txt:466

```
META1_3621          	100.00%		CQW49_RS09435       	100.00%
Bootstrap support for META1_3621 as seed ortholog is 100%.
Bootstrap support for CQW49_RS09435 as seed ortholog is 100%.
```

---

### Group of orthologs #441. Best score 466 bits Score difference with first non-orthologous sequence - AM1\_locus\_tags.txt:244 OB3b\_locus\_tags.txt:466

```
META2_0850          	100.00%		CQW49_RS06715       	100.00%
Bootstrap support for META2_0850 as seed ortholog is 100%.
Bootstrap support for CQW49_RS06715 as seed ortholog is 100%.
```

---

### Group of orthologs #442. Best score 465 bits Score difference with first non-orthologous sequence - AM1\_locus\_tags.txt:50 OB3b\_locus\_tags.txt:465

```
META1_0685          	100.00%		CQW49_RS22125       	100.00%
Bootstrap support for META1_0685 as seed ortholog is 74%.
Alternative seed ortholog is META1_0103 (50 bits away from this cluster)
Bootstrap support for CQW49_RS22125 as seed ortholog is 100%.
```

---

### Group of orthologs #443. Best score 464 bits Score difference with first non-orthologous sequence - AM1\_locus\_tags.txt:108 OB3b\_locus\_tags.txt:464

```
META1_0030          	100.00%		CQW49_RS00415       	100.00%
Bootstrap support for META1_0030 as seed ortholog is 98%.
Bootstrap support for CQW49_RS00415 as seed ortholog is 100%.
```

---

### Group of orthologs #444. Best score 464 bits Score difference with first non-orthologous sequence - AM1\_locus\_tags.txt:464 OB3b\_locus\_tags.txt:65

```
META1_2228          	100.00%		CQW49_RS00355       	100.00%
Bootstrap support for META1_2228 as seed ortholog is 100%.
Bootstrap support for CQW49_RS00355 as seed ortholog is 95%.
```

---

### Group of orthologs #445. Best score 463 bits Score difference with first non-orthologous sequence - AM1\_locus\_tags.txt:184 OB3b\_locus\_tags.txt:164

```
META1_2028          	100.00%		CQW49_RS06710       	100.00%
Bootstrap support for META1_2028 as seed ortholog is 99%.
Bootstrap support for CQW49_RS06710 as seed ortholog is 99%.
```

---

### Group of orthologs #446. Best score 463 bits Score difference with first non-orthologous sequence - AM1\_locus\_tags.txt:463 OB3b\_locus\_tags.txt:308

```
META1_4249          	100.00%		CQW49_RS06935       	100.00%
Bootstrap support for META1_4249 as seed ortholog is 100%.
Bootstrap support for CQW49_RS06935 as seed ortholog is 100%.
```

---

### Group of orthologs #447. Best score 463 bits Score difference with first non-orthologous sequence - AM1\_locus\_tags.txt:463 OB3b\_locus\_tags.txt:463

```
META1_5183          	100.00%		CQW49_RS04285       	100.00%
Bootstrap support for META1_5183 as seed ortholog is 100%.
Bootstrap support for CQW49_RS04285 as seed ortholog is 100%.
```

---

### Group of orthologs #448. Best score 462 bits Score difference with first non-orthologous sequence - AM1\_locus\_tags.txt:301 OB3b\_locus\_tags.txt:176

```
META1_2036          	100.00%		CQW49_RS03740       	100.00%
Bootstrap support for META1_2036 as seed ortholog is 100%.
Bootstrap support for CQW49_RS03740 as seed ortholog is 100%.
```

---

### Group of orthologs #449. Best score 462 bits Score difference with first non-orthologous sequence - AM1\_locus\_tags.txt:249 OB3b\_locus\_tags.txt:400

```
META1_2540          	100.00%		CQW49_RS04715       	100.00%
Bootstrap support for META1_2540 as seed ortholog is 100%.
Bootstrap support for CQW49_RS04715 as seed ortholog is 100%.
```

---

### Group of orthologs #450. Best score 462 bits Score difference with first non-orthologous sequence - AM1\_locus\_tags.txt:462 OB3b\_locus\_tags.txt:462

```
META1_3682          	100.00%		CQW49_RS05085       	100.00%
Bootstrap support for META1_3682 as seed ortholog is 100%.
Bootstrap support for CQW49_RS05085 as seed ortholog is 100%.
```

---

### Group of orthologs #451. Best score 462 bits Score difference with first non-orthologous sequence - AM1\_locus\_tags.txt:217 OB3b\_locus\_tags.txt:462

```
META1_1351          	100.00%		CQW49_RS20955       	100.00%
Bootstrap support for META1_1351 as seed ortholog is 100%.
Bootstrap support for CQW49_RS20955 as seed ortholog is 100%.
```

---

### Group of orthologs #452. Best score 462 bits Score difference with first non-orthologous sequence - AM1\_locus\_tags.txt:315 OB3b\_locus\_tags.txt:462

```
META1_4534          	100.00%		CQW49_RS14485       	100.00%
Bootstrap support for META1_4534 as seed ortholog is 100%.
Bootstrap support for CQW49_RS14485 as seed ortholog is 100%.
```

---

### Group of orthologs #453. Best score 460 bits Score difference with first non-orthologous sequence - AM1\_locus\_tags.txt:460 OB3b\_locus\_tags.txt:460

```
META1_0805          	100.00%		CQW49_RS09645       	100.00%
Bootstrap support for META1_0805 as seed ortholog is 100%.
Bootstrap support for CQW49_RS09645 as seed ortholog is 100%.
```

---

### Group of orthologs #454. Best score 459 bits Score difference with first non-orthologous sequence - AM1\_locus\_tags.txt:459 OB3b\_locus\_tags.txt:459

```
META1_5290          	100.00%		CQW49_RS15505       	100.00%
Bootstrap support for META1_5290 as seed ortholog is 100%.
Bootstrap support for CQW49_RS15505 as seed ortholog is 100%.
```

---

### Group of orthologs #455. Best score 458 bits Score difference with first non-orthologous sequence - AM1\_locus\_tags.txt:162 OB3b\_locus\_tags.txt:458

```
META1_5189          	100.00%		CQW49_RS09230       	100.00%
Bootstrap support for META1_5189 as seed ortholog is 99%.
Bootstrap support for CQW49_RS09230 as seed ortholog is 100%.
```

---

### Group of orthologs #456. Best score 458 bits Score difference with first non-orthologous sequence - AM1\_locus\_tags.txt:377 OB3b\_locus\_tags.txt:458

```
META1_3319          	100.00%		CQW49_RS18880       	100.00%
Bootstrap support for META1_3319 as seed ortholog is 100%.
Bootstrap support for CQW49_RS18880 as seed ortholog is 100%.
```

---

### Group of orthologs #457. Best score 456 bits Score difference with first non-orthologous sequence - AM1\_locus\_tags.txt:290 OB3b\_locus\_tags.txt:456

```
META1_5141          	100.00%		CQW49_RS19495       	100.00%
META1_4199          	77.61%		
Bootstrap support for META1_5141 as seed ortholog is 100%.
Bootstrap support for CQW49_RS19495 as seed ortholog is 100%.
```

---

### Group of orthologs #458. Best score 456 bits Score difference with first non-orthologous sequence - AM1\_locus\_tags.txt:456 OB3b\_locus\_tags.txt:456

```
META1_4633          	100.00%		CQW49_RS01135       	100.00%
Bootstrap support for META1_4633 as seed ortholog is 100%.
Bootstrap support for CQW49_RS01135 as seed ortholog is 100%.
```

---

### Group of orthologs #459. Best score 455 bits Score difference with first non-orthologous sequence - AM1\_locus\_tags.txt:455 OB3b\_locus\_tags.txt:455

```
META1_1527          	100.00%		CQW49_RS09500       	100.00%
Bootstrap support for META1_1527 as seed ortholog is 100%.
Bootstrap support for CQW49_RS09500 as seed ortholog is 100%.
```

---

### Group of orthologs #460. Best score 453 bits Score difference with first non-orthologous sequence - AM1\_locus\_tags.txt:453 OB3b\_locus\_tags.txt:453

```
META1_3151          	100.00%		CQW49_RS11285       	100.00%
Bootstrap support for META1_3151 as seed ortholog is 100%.
Bootstrap support for CQW49_RS11285 as seed ortholog is 100%.
```

---

### Group of orthologs #461. Best score 453 bits Score difference with first non-orthologous sequence - AM1\_locus\_tags.txt:453 OB3b\_locus\_tags.txt:453

```
META1_4713          	100.00%		CQW49_RS16625       	100.00%
Bootstrap support for META1_4713 as seed ortholog is 100%.
Bootstrap support for CQW49_RS16625 as seed ortholog is 100%.
```

---

### Group of orthologs #462. Best score 452 bits Score difference with first non-orthologous sequence - AM1\_locus\_tags.txt:374 OB3b\_locus\_tags.txt:265

```
META1_2582          	100.00%		CQW49_RS22345       	100.00%
META1_1198          	59.34%		
META2_1016          	57.07%		
Bootstrap support for META1_2582 as seed ortholog is 100%.
Bootstrap support for CQW49_RS22345 as seed ortholog is 100%.
```

---

### Group of orthologs #463. Best score 452 bits Score difference with first non-orthologous sequence - AM1\_locus\_tags.txt:452 OB3b\_locus\_tags.txt:452

```
META1_2920          	100.00%		CQW49_RS21225       	100.00%
Bootstrap support for META1_2920 as seed ortholog is 100%.
Bootstrap support for CQW49_RS21225 as seed ortholog is 100%.
```

---

### Group of orthologs #464. Best score 451 bits Score difference with first non-orthologous sequence - AM1\_locus\_tags.txt:301 OB3b\_locus\_tags.txt:451

```
META1_3416          	100.00%		CQW49_RS09985       	100.00%
Bootstrap support for META1_3416 as seed ortholog is 100%.
Bootstrap support for CQW49_RS09985 as seed ortholog is 100%.
```

---

### Group of orthologs #465. Best score 451 bits Score difference with first non-orthologous sequence - AM1\_locus\_tags.txt:451 OB3b\_locus\_tags.txt:451

```
META1_4234          	100.00%		CQW49_RS09385       	100.00%
Bootstrap support for META1_4234 as seed ortholog is 100%.
Bootstrap support for CQW49_RS09385 as seed ortholog is 100%.
```

---

### Group of orthologs #466. Best score 450 bits Score difference with first non-orthologous sequence - AM1\_locus\_tags.txt:86 OB3b\_locus\_tags.txt:393

```
META1_4521          	100.00%		CQW49_RS06910       	100.00%
Bootstrap support for META1_4521 as seed ortholog is 99%.
Bootstrap support for CQW49_RS06910 as seed ortholog is 100%.
```

---

### Group of orthologs #467. Best score 450 bits Score difference with first non-orthologous sequence - AM1\_locus\_tags.txt:450 OB3b\_locus\_tags.txt:450

```
META1_5086          	100.00%		CQW49_RS11240       	100.00%
Bootstrap support for META1_5086 as seed ortholog is 100%.
Bootstrap support for CQW49_RS11240 as seed ortholog is 100%.
```

---

### Group of orthologs #468. Best score 450 bits Score difference with first non-orthologous sequence - AM1\_locus\_tags.txt:450 OB3b\_locus\_tags.txt:450

```
META1_3139          	100.00%		CQW49_RS20845       	100.00%
Bootstrap support for META1_3139 as seed ortholog is 100%.
Bootstrap support for CQW49_RS20845 as seed ortholog is 100%.
```

---

### Group of orthologs #469. Best score 449 bits Score difference with first non-orthologous sequence - AM1\_locus\_tags.txt:362 OB3b\_locus\_tags.txt:365

```
META1_1486          	100.00%		CQW49_RS08860       	100.00%
Bootstrap support for META1_1486 as seed ortholog is 100%.
Bootstrap support for CQW49_RS08860 as seed ortholog is 100%.
```

---

### Group of orthologs #470. Best score 449 bits Score difference with first non-orthologous sequence - AM1\_locus\_tags.txt:449 OB3b\_locus\_tags.txt:449

```
META1_4394          	100.00%		CQW49_RS05070       	100.00%
Bootstrap support for META1_4394 as seed ortholog is 100%.
Bootstrap support for CQW49_RS05070 as seed ortholog is 100%.
```

---

### Group of orthologs #471. Best score 447 bits Score difference with first non-orthologous sequence - AM1\_locus\_tags.txt:447 OB3b\_locus\_tags.txt:26

```
META1_2229          	100.00%		CQW49_RS00350       	100.00%
Bootstrap support for META1_2229 as seed ortholog is 100%.
Bootstrap support for CQW49_RS00350 as seed ortholog is 83%.
```

---

### Group of orthologs #472. Best score 447 bits Score difference with first non-orthologous sequence - AM1\_locus\_tags.txt:447 OB3b\_locus\_tags.txt:447

```
META1_0314          	100.00%		CQW49_RS17125       	100.00%
Bootstrap support for META1_0314 as seed ortholog is 100%.
Bootstrap support for CQW49_RS17125 as seed ortholog is 100%.
```

---

### Group of orthologs #473. Best score 446 bits Score difference with first non-orthologous sequence - AM1\_locus\_tags.txt:446 OB3b\_locus\_tags.txt:446

```
META1_1278          	100.00%		CQW49_RS00920       	100.00%
Bootstrap support for META1_1278 as seed ortholog is 100%.
Bootstrap support for CQW49_RS00920 as seed ortholog is 100%.
```

---

### Group of orthologs #474. Best score 446 bits Score difference with first non-orthologous sequence - AM1\_locus\_tags.txt:315 OB3b\_locus\_tags.txt:446

```
META1_1727          	100.00%		CQW49_RS12240       	100.00%
Bootstrap support for META1_1727 as seed ortholog is 100%.
Bootstrap support for CQW49_RS12240 as seed ortholog is 100%.
```

---

### Group of orthologs #475. Best score 444 bits Score difference with first non-orthologous sequence - AM1\_locus\_tags.txt:444 OB3b\_locus\_tags.txt:444

```
META1_2918          	100.00%		CQW49_RS00235       	100.00%
Bootstrap support for META1_2918 as seed ortholog is 100%.
Bootstrap support for CQW49_RS00235 as seed ortholog is 100%.
```

---

### Group of orthologs #476. Best score 443 bits Score difference with first non-orthologous sequence - AM1\_locus\_tags.txt:339 OB3b\_locus\_tags.txt:400

```
META1_2395          	100.00%		CQW49_RS06255       	100.00%
META1_4041          	92.20%		
Bootstrap support for META1_2395 as seed ortholog is 100%.
Bootstrap support for CQW49_RS06255 as seed ortholog is 100%.
```

---

### Group of orthologs #477. Best score 443 bits Score difference with first non-orthologous sequence - AM1\_locus\_tags.txt:443 OB3b\_locus\_tags.txt:443

```
META1_0558          	100.00%		CQW49_RS11625       	100.00%
Bootstrap support for META1_0558 as seed ortholog is 100%.
Bootstrap support for CQW49_RS11625 as seed ortholog is 100%.
```

---

### Group of orthologs #478. Best score 443 bits Score difference with first non-orthologous sequence - AM1\_locus\_tags.txt:443 OB3b\_locus\_tags.txt:443

```
META1_3283          	100.00%		CQW49_RS01500       	100.00%
Bootstrap support for META1_3283 as seed ortholog is 100%.
Bootstrap support for CQW49_RS01500 as seed ortholog is 100%.
```

---

### Group of orthologs #479. Best score 442 bits Score difference with first non-orthologous sequence - AM1\_locus\_tags.txt:355 OB3b\_locus\_tags.txt:442

```
META1_0808          	100.00%		CQW49_RS16740       	100.00%
Bootstrap support for META1_0808 as seed ortholog is 100%.
Bootstrap support for CQW49_RS16740 as seed ortholog is 100%.
```

---

### Group of orthologs #480. Best score 442 bits Score difference with first non-orthologous sequence - AM1\_locus\_tags.txt:259 OB3b\_locus\_tags.txt:385

```
META1_3915          	100.00%		CQW49_RS14445       	100.00%
Bootstrap support for META1_3915 as seed ortholog is 100%.
Bootstrap support for CQW49_RS14445 as seed ortholog is 100%.
```

---

### Group of orthologs #481. Best score 441 bits Score difference with first non-orthologous sequence - AM1\_locus\_tags.txt:441 OB3b\_locus\_tags.txt:441

```
META1_1002          	100.00%		CQW49_RS13640       	100.00%
Bootstrap support for META1_1002 as seed ortholog is 100%.
Bootstrap support for CQW49_RS13640 as seed ortholog is 100%.
```

---

### Group of orthologs #482. Best score 441 bits Score difference with first non-orthologous sequence - AM1\_locus\_tags.txt:441 OB3b\_locus\_tags.txt:441

```
META1_0380          	100.00%		CQW49_RS17750       	100.00%
Bootstrap support for META1_0380 as seed ortholog is 100%.
Bootstrap support for CQW49_RS17750 as seed ortholog is 100%.
```

---

### Group of orthologs #483. Best score 441 bits Score difference with first non-orthologous sequence - AM1\_locus\_tags.txt:387 OB3b\_locus\_tags.txt:441

```
META1_3486          	100.00%		CQW49_RS16640       	100.00%
Bootstrap support for META1_3486 as seed ortholog is 100%.
Bootstrap support for CQW49_RS16640 as seed ortholog is 100%.
```

---

### Group of orthologs #484. Best score 441 bits Score difference with first non-orthologous sequence - AM1\_locus\_tags.txt:56 OB3b\_locus\_tags.txt:441

```
META1_3558          	100.00%		CQW49_RS22220       	100.00%
Bootstrap support for META1_3558 as seed ortholog is 87%.
Bootstrap support for CQW49_RS22220 as seed ortholog is 100%.
```

---

### Group of orthologs #485. Best score 440 bits Score difference with first non-orthologous sequence - AM1\_locus\_tags.txt:440 OB3b\_locus\_tags.txt:440

```
META1_0751          	100.00%		CQW49_RS10365       	100.00%
Bootstrap support for META1_0751 as seed ortholog is 100%.
Bootstrap support for CQW49_RS10365 as seed ortholog is 100%.
```

---

### Group of orthologs #486. Best score 440 bits Score difference with first non-orthologous sequence - AM1\_locus\_tags.txt:343 OB3b\_locus\_tags.txt:440

```
META1_0778          	100.00%		CQW49_RS15595       	100.00%
Bootstrap support for META1_0778 as seed ortholog is 100%.
Bootstrap support for CQW49_RS15595 as seed ortholog is 100%.
```

---

### Group of orthologs #487. Best score 440 bits Score difference with first non-orthologous sequence - AM1\_locus\_tags.txt:97 OB3b\_locus\_tags.txt:440

```
META1_0448          	100.00%		CQW49_RS18830       	100.00%
Bootstrap support for META1_0448 as seed ortholog is 99%.
Bootstrap support for CQW49_RS18830 as seed ortholog is 100%.
```

---

### Group of orthologs #488. Best score 440 bits Score difference with first non-orthologous sequence - AM1\_locus\_tags.txt:440 OB3b\_locus\_tags.txt:440

```
META1_0836          	100.00%		CQW49_RS20930       	100.00%
Bootstrap support for META1_0836 as seed ortholog is 100%.
Bootstrap support for CQW49_RS20930 as seed ortholog is 100%.
```

---

### Group of orthologs #489. Best score 440 bits Score difference with first non-orthologous sequence - AM1\_locus\_tags.txt:440 OB3b\_locus\_tags.txt:440

```
META1_4465          	100.00%		CQW49_RS13090       	100.00%
Bootstrap support for META1_4465 as seed ortholog is 100%.
Bootstrap support for CQW49_RS13090 as seed ortholog is 100%.
```

---

### Group of orthologs #490. Best score 440 bits Score difference with first non-orthologous sequence - AM1\_locus\_tags.txt:440 OB3b\_locus\_tags.txt:440

```
META1_3434          	100.00%		CQW49_RS21270       	100.00%
Bootstrap support for META1_3434 as seed ortholog is 100%.
Bootstrap support for CQW49_RS21270 as seed ortholog is 100%.
```

---

### Group of orthologs #491. Best score 439 bits Score difference with first non-orthologous sequence - AM1\_locus\_tags.txt:439 OB3b\_locus\_tags.txt:439

```
META1_2836          	100.00%		CQW49_RS10335       	100.00%
Bootstrap support for META1_2836 as seed ortholog is 100%.
Bootstrap support for CQW49_RS10335 as seed ortholog is 100%.
```

---

### Group of orthologs #492. Best score 438 bits Score difference with first non-orthologous sequence - AM1\_locus\_tags.txt:438 OB3b\_locus\_tags.txt:438

```
META1_1236          	100.00%		CQW49_RS01445       	100.00%
META2_1135          	40.97%		
META1_3501          	8.56%		
Bootstrap support for META1_1236 as seed ortholog is 100%.
Bootstrap support for CQW49_RS01445 as seed ortholog is 100%.
```

---

### Group of orthologs #493. Best score 437 bits Score difference with first non-orthologous sequence - AM1\_locus\_tags.txt:437 OB3b\_locus\_tags.txt:437

```
META1_0283          	100.00%		CQW49_RS03005       	100.00%
Bootstrap support for META1_0283 as seed ortholog is 100%.
Bootstrap support for CQW49_RS03005 as seed ortholog is 100%.
```

---

### Group of orthologs #494. Best score 437 bits Score difference with first non-orthologous sequence - AM1\_locus\_tags.txt:437 OB3b\_locus\_tags.txt:437

```
META1_2713          	100.00%		CQW49_RS17580       	100.00%
Bootstrap support for META1_2713 as seed ortholog is 100%.
Bootstrap support for CQW49_RS17580 as seed ortholog is 100%.
```

---

### Group of orthologs #495. Best score 437 bits Score difference with first non-orthologous sequence - AM1\_locus\_tags.txt:437 OB3b\_locus\_tags.txt:437

```
META1_1405          	100.00%		CQW49_RS23720       	100.00%
Bootstrap support for META1_1405 as seed ortholog is 100%.
Bootstrap support for CQW49_RS23720 as seed ortholog is 100%.
```

---

### Group of orthologs #496. Best score 436 bits Score difference with first non-orthologous sequence - AM1\_locus\_tags.txt:436 OB3b\_locus\_tags.txt:436

```
META1_4148          	100.00%		CQW49_RS05575       	100.00%
Bootstrap support for META1_4148 as seed ortholog is 100%.
Bootstrap support for CQW49_RS05575 as seed ortholog is 100%.
```

---

### Group of orthologs #497. Best score 435 bits Score difference with first non-orthologous sequence - AM1\_locus\_tags.txt:286 OB3b\_locus\_tags.txt:435

```
META1_1140          	100.00%		CQW49_RS16725       	100.00%
Bootstrap support for META1_1140 as seed ortholog is 100%.
Bootstrap support for CQW49_RS16725 as seed ortholog is 100%.
```

---

### Group of orthologs #498. Best score 435 bits Score difference with first non-orthologous sequence - AM1\_locus\_tags.txt:341 OB3b\_locus\_tags.txt:435

```
META2_0464          	100.00%		CQW49_RS19505       	100.00%
Bootstrap support for META2_0464 as seed ortholog is 100%.
Bootstrap support for CQW49_RS19505 as seed ortholog is 100%.
```

---

### Group of orthologs #499. Best score 434 bits Score difference with first non-orthologous sequence - AM1\_locus\_tags.txt:434 OB3b\_locus\_tags.txt:434

```
META1_2068          	100.00%		CQW49_RS00455       	100.00%
Bootstrap support for META1_2068 as seed ortholog is 100%.
Bootstrap support for CQW49_RS00455 as seed ortholog is 100%.
```

---

### Group of orthologs #500. Best score 434 bits Score difference with first non-orthologous sequence - AM1\_locus\_tags.txt:61 OB3b\_locus\_tags.txt:296

```
META1_0590          	100.00%		CQW49_RS08595       	100.00%
Bootstrap support for META1_0590 as seed ortholog is 99%.
Bootstrap support for CQW49_RS08595 as seed ortholog is 100%.
```

---

### Group of orthologs #501. Best score 434 bits Score difference with first non-orthologous sequence - AM1\_locus\_tags.txt:305 OB3b\_locus\_tags.txt:284

```
META1_0862          	100.00%		CQW49_RS10605       	100.00%
Bootstrap support for META1_0862 as seed ortholog is 100%.
Bootstrap support for CQW49_RS10605 as seed ortholog is 100%.
```

---

### Group of orthologs #502. Best score 434 bits Score difference with first non-orthologous sequence - AM1\_locus\_tags.txt:434 OB3b\_locus\_tags.txt:434

```
META1_2521          	100.00%		CQW49_RS07650       	100.00%
Bootstrap support for META1_2521 as seed ortholog is 100%.
Bootstrap support for CQW49_RS07650 as seed ortholog is 100%.
```

---

### Group of orthologs #503. Best score 431 bits Score difference with first non-orthologous sequence - AM1\_locus\_tags.txt:431 OB3b\_locus\_tags.txt:431

```
META1_3122          	100.00%		CQW49_RS01320       	100.00%
Bootstrap support for META1_3122 as seed ortholog is 100%.
Bootstrap support for CQW49_RS01320 as seed ortholog is 100%.
```

---

### Group of orthologs #504. Best score 431 bits Score difference with first non-orthologous sequence - AM1\_locus\_tags.txt:182 OB3b\_locus\_tags.txt:379

```
META1_2220          	100.00%		CQW49_RS09130       	100.00%
Bootstrap support for META1_2220 as seed ortholog is 99%.
Bootstrap support for CQW49_RS09130 as seed ortholog is 100%.
```

---

### Group of orthologs #505. Best score 431 bits Score difference with first non-orthologous sequence - AM1\_locus\_tags.txt:431 OB3b\_locus\_tags.txt:431

```
META1_3212          	100.00%		CQW49_RS20545       	100.00%
Bootstrap support for META1_3212 as seed ortholog is 100%.
Bootstrap support for CQW49_RS20545 as seed ortholog is 100%.
```

---

### Group of orthologs #506. Best score 430 bits Score difference with first non-orthologous sequence - AM1\_locus\_tags.txt:430 OB3b\_locus\_tags.txt:430

```
META1_1937          	100.00%		CQW49_RS21005       	100.00%
Bootstrap support for META1_1937 as seed ortholog is 100%.
Bootstrap support for CQW49_RS21005 as seed ortholog is 100%.
```

---

### Group of orthologs #507. Best score 430 bits Score difference with first non-orthologous sequence - AM1\_locus\_tags.txt:95 OB3b\_locus\_tags.txt:294

```
META1_4653          	100.00%		CQW49_RS17935       	100.00%
Bootstrap support for META1_4653 as seed ortholog is 99%.
Bootstrap support for CQW49_RS17935 as seed ortholog is 100%.
```

---

### Group of orthologs #508. Best score 429 bits Score difference with first non-orthologous sequence - AM1\_locus\_tags.txt:335 OB3b\_locus\_tags.txt:429

```
META1_3485          	100.00%		CQW49_RS16635       	100.00%
Bootstrap support for META1_3485 as seed ortholog is 100%.
Bootstrap support for CQW49_RS16635 as seed ortholog is 100%.
```

---

### Group of orthologs #509. Best score 428 bits Score difference with first non-orthologous sequence - AM1\_locus\_tags.txt:428 OB3b\_locus\_tags.txt:428

```
META1_0720          	100.00%		CQW49_RS08900       	100.00%
Bootstrap support for META1_0720 as seed ortholog is 100%.
Bootstrap support for CQW49_RS08900 as seed ortholog is 100%.
```

---

### Group of orthologs #510. Best score 428 bits Score difference with first non-orthologous sequence - AM1\_locus\_tags.txt:287 OB3b\_locus\_tags.txt:428

```
META1_2375          	100.00%		CQW49_RS04165       	100.00%
Bootstrap support for META1_2375 as seed ortholog is 100%.
Bootstrap support for CQW49_RS04165 as seed ortholog is 100%.
```

---

### Group of orthologs #511. Best score 428 bits Score difference with first non-orthologous sequence - AM1\_locus\_tags.txt:361 OB3b\_locus\_tags.txt:352

```
META1_3020          	100.00%		CQW49_RS01670       	100.00%
Bootstrap support for META1_3020 as seed ortholog is 100%.
Bootstrap support for CQW49_RS01670 as seed ortholog is 100%.
```

---

### Group of orthologs #512. Best score 427 bits Score difference with first non-orthologous sequence - AM1\_locus\_tags.txt:427 OB3b\_locus\_tags.txt:427

```
META1_2512          	100.00%		CQW49_RS00280       	100.00%
Bootstrap support for META1_2512 as seed ortholog is 100%.
Bootstrap support for CQW49_RS00280 as seed ortholog is 100%.
```

---

### Group of orthologs #513. Best score 427 bits Score difference with first non-orthologous sequence - AM1\_locus\_tags.txt:427 OB3b\_locus\_tags.txt:427

```
META1_5116          	100.00%		CQW49_RS20345       	100.00%
Bootstrap support for META1_5116 as seed ortholog is 100%.
Bootstrap support for CQW49_RS20345 as seed ortholog is 100%.
```

---

### Group of orthologs #514. Best score 426 bits Score difference with first non-orthologous sequence - AM1\_locus\_tags.txt:426 OB3b\_locus\_tags.txt:426

```
META1_0712          	100.00%		CQW49_RS19450       	100.00%
Bootstrap support for META1_0712 as seed ortholog is 100%.
Bootstrap support for CQW49_RS19450 as seed ortholog is 100%.
```

---

### Group of orthologs #515. Best score 426 bits Score difference with first non-orthologous sequence - AM1\_locus\_tags.txt:324 OB3b\_locus\_tags.txt:344

```
META2_0114          	100.00%		CQW49_RS14880       	100.00%
Bootstrap support for META2_0114 as seed ortholog is 100%.
Bootstrap support for CQW49_RS14880 as seed ortholog is 100%.
```

---

### Group of orthologs #516. Best score 425 bits Score difference with first non-orthologous sequence - AM1\_locus\_tags.txt:363 OB3b\_locus\_tags.txt:425

```
META1_0403          	100.00%		CQW49_RS18530       	100.00%
                    	       		CQW49_RS09460       	27.61%
Bootstrap support for META1_0403 as seed ortholog is 100%.
Bootstrap support for CQW49_RS18530 as seed ortholog is 100%.
```

---

### Group of orthologs #517. Best score 425 bits Score difference with first non-orthologous sequence - AM1\_locus\_tags.txt:425 OB3b\_locus\_tags.txt:425

```
META1_1436          	100.00%		CQW49_RS20310       	100.00%
Bootstrap support for META1_1436 as seed ortholog is 100%.
Bootstrap support for CQW49_RS20310 as seed ortholog is 100%.
```

---

### Group of orthologs #518. Best score 424 bits Score difference with first non-orthologous sequence - AM1\_locus\_tags.txt:424 OB3b\_locus\_tags.txt:348

```
META1_1320          	100.00%		CQW49_RS04075       	100.00%
Bootstrap support for META1_1320 as seed ortholog is 100%.
Bootstrap support for CQW49_RS04075 as seed ortholog is 100%.
```

---

### Group of orthologs #519. Best score 424 bits Score difference with first non-orthologous sequence - AM1\_locus\_tags.txt:424 OB3b\_locus\_tags.txt:424

```
META1_1517          	100.00%		CQW49_RS16285       	100.00%
Bootstrap support for META1_1517 as seed ortholog is 100%.
Bootstrap support for CQW49_RS16285 as seed ortholog is 100%.
```

---

### Group of orthologs #520. Best score 421 bits Score difference with first non-orthologous sequence - AM1\_locus\_tags.txt:205 OB3b\_locus\_tags.txt:229

```
META1_1296          	100.00%		CQW49_RS03970       	100.00%
Bootstrap support for META1_1296 as seed ortholog is 100%.
Bootstrap support for CQW49_RS03970 as seed ortholog is 100%.
```

---

### Group of orthologs #521. Best score 420 bits Score difference with first non-orthologous sequence - AM1\_locus\_tags.txt:420 OB3b\_locus\_tags.txt:420

```
META1_2275          	100.00%		CQW49_RS13485       	100.00%
Bootstrap support for META1_2275 as seed ortholog is 100%.
Bootstrap support for CQW49_RS13485 as seed ortholog is 100%.
```

---

### Group of orthologs #522. Best score 420 bits Score difference with first non-orthologous sequence - AM1\_locus\_tags.txt:420 OB3b\_locus\_tags.txt:420

```
META1_3150          	100.00%		CQW49_RS11270       	100.00%
Bootstrap support for META1_3150 as seed ortholog is 100%.
Bootstrap support for CQW49_RS11270 as seed ortholog is 100%.
```

---

### Group of orthologs #523. Best score 420 bits Score difference with first non-orthologous sequence - AM1\_locus\_tags.txt:182 OB3b\_locus\_tags.txt:198

```
META1_3932          	100.00%		CQW49_RS09140       	100.00%
Bootstrap support for META1_3932 as seed ortholog is 99%.
Bootstrap support for CQW49_RS09140 as seed ortholog is 99%.
```

---

### Group of orthologs #524. Best score 420 bits Score difference with first non-orthologous sequence - AM1\_locus\_tags.txt:420 OB3b\_locus\_tags.txt:420

```
META1_2714          	100.00%		CQW49_RS17575       	100.00%
Bootstrap support for META1_2714 as seed ortholog is 100%.
Bootstrap support for CQW49_RS17575 as seed ortholog is 100%.
```

---

### Group of orthologs #525. Best score 420 bits Score difference with first non-orthologous sequence - AM1\_locus\_tags.txt:188 OB3b\_locus\_tags.txt:420

```
META1_5123          	100.00%		CQW49_RS20235       	100.00%
Bootstrap support for META1_5123 as seed ortholog is 100%.
Bootstrap support for CQW49_RS20235 as seed ortholog is 100%.
```

---

### Group of orthologs #526. Best score 418 bits Score difference with first non-orthologous sequence - AM1\_locus\_tags.txt:418 OB3b\_locus\_tags.txt:418

```
META1_2387          	100.00%		CQW49_RS16585       	100.00%
Bootstrap support for META1_2387 as seed ortholog is 100%.
Bootstrap support for CQW49_RS16585 as seed ortholog is 100%.
```

---

### Group of orthologs #527. Best score 418 bits Score difference with first non-orthologous sequence - AM1\_locus\_tags.txt:418 OB3b\_locus\_tags.txt:418

```
META1_2445          	100.00%		CQW49_RS17405       	100.00%
Bootstrap support for META1_2445 as seed ortholog is 100%.
Bootstrap support for CQW49_RS17405 as seed ortholog is 100%.
```

---

### Group of orthologs #528. Best score 416 bits Score difference with first non-orthologous sequence - AM1\_locus\_tags.txt:416 OB3b\_locus\_tags.txt:416

```
META1_0371          	100.00%		CQW49_RS01760       	100.00%
Bootstrap support for META1_0371 as seed ortholog is 100%.
Bootstrap support for CQW49_RS01760 as seed ortholog is 100%.
```

---

### Group of orthologs #529. Best score 416 bits Score difference with first non-orthologous sequence - AM1\_locus\_tags.txt:416 OB3b\_locus\_tags.txt:416

```
META1_0003          	100.00%		CQW49_RS04605       	100.00%
Bootstrap support for META1_0003 as seed ortholog is 100%.
Bootstrap support for CQW49_RS04605 as seed ortholog is 100%.
```

---

### Group of orthologs #530. Best score 416 bits Score difference with first non-orthologous sequence - AM1\_locus\_tags.txt:342 OB3b\_locus\_tags.txt:416

```
META1_1485          	100.00%		CQW49_RS08855       	100.00%
Bootstrap support for META1_1485 as seed ortholog is 100%.
Bootstrap support for CQW49_RS08855 as seed ortholog is 100%.
```

---

### Group of orthologs #531. Best score 416 bits Score difference with first non-orthologous sequence - AM1\_locus\_tags.txt:416 OB3b\_locus\_tags.txt:416

```
META1_2870          	100.00%		CQW49_RS03155       	100.00%
Bootstrap support for META1_2870 as seed ortholog is 100%.
Bootstrap support for CQW49_RS03155 as seed ortholog is 100%.
```

---

### Group of orthologs #532. Best score 416 bits Score difference with first non-orthologous sequence - AM1\_locus\_tags.txt:96 OB3b\_locus\_tags.txt:416

```
META1_5107          	100.00%		CQW49_RS06625       	100.00%
Bootstrap support for META1_5107 as seed ortholog is 99%.
Bootstrap support for CQW49_RS06625 as seed ortholog is 100%.
```

---

### Group of orthologs #533. Best score 415 bits Score difference with first non-orthologous sequence - AM1\_locus\_tags.txt:415 OB3b\_locus\_tags.txt:415

```
META1_1697          	100.00%		CQW49_RS09165       	100.00%
Bootstrap support for META1_1697 as seed ortholog is 100%.
Bootstrap support for CQW49_RS09165 as seed ortholog is 100%.
```

---

### Group of orthologs #534. Best score 413 bits Score difference with first non-orthologous sequence - AM1\_locus\_tags.txt:322 OB3b\_locus\_tags.txt:250

```
META1_0541          	100.00%		CQW49_RS09040       	100.00%
Bootstrap support for META1_0541 as seed ortholog is 100%.
Bootstrap support for CQW49_RS09040 as seed ortholog is 100%.
```

---

### Group of orthologs #535. Best score 413 bits Score difference with first non-orthologous sequence - AM1\_locus\_tags.txt:413 OB3b\_locus\_tags.txt:413

```
META1_1940          	100.00%		CQW49_RS20835       	100.00%
Bootstrap support for META1_1940 as seed ortholog is 100%.
Bootstrap support for CQW49_RS20835 as seed ortholog is 100%.
```

---

### Group of orthologs #536. Best score 412 bits Score difference with first non-orthologous sequence - AM1\_locus\_tags.txt:412 OB3b\_locus\_tags.txt:412

```
META1_5204          	100.00%		CQW49_RS04460       	100.00%
Bootstrap support for META1_5204 as seed ortholog is 100%.
Bootstrap support for CQW49_RS04460 as seed ortholog is 100%.
```

---

### Group of orthologs #537. Best score 411 bits Score difference with first non-orthologous sequence - AM1\_locus\_tags.txt:345 OB3b\_locus\_tags.txt:411

```
META1_3469          	100.00%		CQW49_RS10045       	100.00%
Bootstrap support for META1_3469 as seed ortholog is 100%.
Bootstrap support for CQW49_RS10045 as seed ortholog is 100%.
```

---

### Group of orthologs #538. Best score 410 bits Score difference with first non-orthologous sequence - AM1\_locus\_tags.txt:410 OB3b\_locus\_tags.txt:410

```
META1_3429          	100.00%		CQW49_RS00075       	100.00%
Bootstrap support for META1_3429 as seed ortholog is 100%.
Bootstrap support for CQW49_RS00075 as seed ortholog is 100%.
```

---

### Group of orthologs #539. Best score 410 bits Score difference with first non-orthologous sequence - AM1\_locus\_tags.txt:410 OB3b\_locus\_tags.txt:368

```
META1_1053          	100.00%		CQW49_RS11465       	100.00%
Bootstrap support for META1_1053 as seed ortholog is 100%.
Bootstrap support for CQW49_RS11465 as seed ortholog is 100%.
```

---

### Group of orthologs #540. Best score 410 bits Score difference with first non-orthologous sequence - AM1\_locus\_tags.txt:410 OB3b\_locus\_tags.txt:321

```
META1_3626          	100.00%		CQW49_RS06210       	100.00%
Bootstrap support for META1_3626 as seed ortholog is 100%.
Bootstrap support for CQW49_RS06210 as seed ortholog is 100%.
```

---

### Group of orthologs #541. Best score 410 bits Score difference with first non-orthologous sequence - AM1\_locus\_tags.txt:410 OB3b\_locus\_tags.txt:324

```
META1_5145          	100.00%		CQW49_RS10385       	100.00%
Bootstrap support for META1_5145 as seed ortholog is 100%.
Bootstrap support for CQW49_RS10385 as seed ortholog is 100%.
```

---

### Group of orthologs #542. Best score 409 bits Score difference with first non-orthologous sequence - AM1\_locus\_tags.txt:409 OB3b\_locus\_tags.txt:409

```
META1_5257          	100.00%		CQW49_RS01725       	100.00%
Bootstrap support for META1_5257 as seed ortholog is 100%.
Bootstrap support for CQW49_RS01725 as seed ortholog is 100%.
```

---

### Group of orthologs #543. Best score 409 bits Score difference with first non-orthologous sequence - AM1\_locus\_tags.txt:409 OB3b\_locus\_tags.txt:409

```
META1_5094          	100.00%		CQW49_RS18475       	100.00%
Bootstrap support for META1_5094 as seed ortholog is 100%.
Bootstrap support for CQW49_RS18475 as seed ortholog is 100%.
```

---

### Group of orthologs #544. Best score 408 bits Score difference with first non-orthologous sequence - AM1\_locus\_tags.txt:408 OB3b\_locus\_tags.txt:408

```
META1_2035          	100.00%		CQW49_RS03735       	100.00%
Bootstrap support for META1_2035 as seed ortholog is 100%.
Bootstrap support for CQW49_RS03735 as seed ortholog is 100%.
```

---

### Group of orthologs #545. Best score 408 bits Score difference with first non-orthologous sequence - AM1\_locus\_tags.txt:252 OB3b\_locus\_tags.txt:408

```
META1_1161          	100.00%		CQW49_RS11660       	100.00%
Bootstrap support for META1_1161 as seed ortholog is 100%.
Bootstrap support for CQW49_RS11660 as seed ortholog is 100%.
```

---

### Group of orthologs #546. Best score 408 bits Score difference with first non-orthologous sequence - AM1\_locus\_tags.txt:408 OB3b\_locus\_tags.txt:408

```
META1_1465          	100.00%		CQW49_RS16605       	100.00%
Bootstrap support for META1_1465 as seed ortholog is 100%.
Bootstrap support for CQW49_RS16605 as seed ortholog is 100%.
```

---

### Group of orthologs #547. Best score 407 bits Score difference with first non-orthologous sequence - AM1\_locus\_tags.txt:407 OB3b\_locus\_tags.txt:407

```
META1_3894          	100.00%		CQW49_RS11340       	100.00%
Bootstrap support for META1_3894 as seed ortholog is 100%.
Bootstrap support for CQW49_RS11340 as seed ortholog is 100%.
```

---

### Group of orthologs #548. Best score 407 bits Score difference with first non-orthologous sequence - AM1\_locus\_tags.txt:289 OB3b\_locus\_tags.txt:407

```
META1_5226          	100.00%		CQW49_RS06565       	100.00%
Bootstrap support for META1_5226 as seed ortholog is 100%.
Bootstrap support for CQW49_RS06565 as seed ortholog is 100%.
```

---

### Group of orthologs #549. Best score 406 bits Score difference with first non-orthologous sequence - AM1\_locus\_tags.txt:406 OB3b\_locus\_tags.txt:406

```
META1_0167          	100.00%		CQW49_RS12000       	100.00%
Bootstrap support for META1_0167 as seed ortholog is 100%.
Bootstrap support for CQW49_RS12000 as seed ortholog is 100%.
```

---

### Group of orthologs #550. Best score 406 bits Score difference with first non-orthologous sequence - AM1\_locus\_tags.txt:279 OB3b\_locus\_tags.txt:274

```
META1_1023          	100.00%		CQW49_RS12370       	100.00%
Bootstrap support for META1_1023 as seed ortholog is 100%.
Bootstrap support for CQW49_RS12370 as seed ortholog is 100%.
```

---

### Group of orthologs #551. Best score 406 bits Score difference with first non-orthologous sequence - AM1\_locus\_tags.txt:331 OB3b\_locus\_tags.txt:328

```
META1_0405          	100.00%		CQW49_RS18540       	100.00%
Bootstrap support for META1_0405 as seed ortholog is 100%.
Bootstrap support for CQW49_RS18540 as seed ortholog is 100%.
```

---

### Group of orthologs #552. Best score 406 bits Score difference with first non-orthologous sequence - AM1\_locus\_tags.txt:126 OB3b\_locus\_tags.txt:406

```
META1_2756          	100.00%		CQW49_RS15555       	100.00%
Bootstrap support for META1_2756 as seed ortholog is 99%.
Bootstrap support for CQW49_RS15555 as seed ortholog is 100%.
```

---

### Group of orthologs #553. Best score 406 bits Score difference with first non-orthologous sequence - AM1\_locus\_tags.txt:189 OB3b\_locus\_tags.txt:406

```
META1_5134          	100.00%		CQW49_RS11075       	100.00%
Bootstrap support for META1_5134 as seed ortholog is 100%.
Bootstrap support for CQW49_RS11075 as seed ortholog is 100%.
```

---

### Group of orthologs #554. Best score 404 bits Score difference with first non-orthologous sequence - AM1\_locus\_tags.txt:263 OB3b\_locus\_tags.txt:404

```
META1_2197          	100.00%		CQW49_RS05390       	100.00%
Bootstrap support for META1_2197 as seed ortholog is 100%.
Bootstrap support for CQW49_RS05390 as seed ortholog is 100%.
```

---

### Group of orthologs #555. Best score 404 bits Score difference with first non-orthologous sequence - AM1\_locus\_tags.txt:120 OB3b\_locus\_tags.txt:404

```
META1_1823          	100.00%		CQW49_RS07665       	100.00%
Bootstrap support for META1_1823 as seed ortholog is 99%.
Bootstrap support for CQW49_RS07665 as seed ortholog is 100%.
```

---

### Group of orthologs #556. Best score 404 bits Score difference with first non-orthologous sequence - AM1\_locus\_tags.txt:404 OB3b\_locus\_tags.txt:81

```
META1_4715          	100.00%		CQW49_RS00030       	100.00%
Bootstrap support for META1_4715 as seed ortholog is 100%.
Bootstrap support for CQW49_RS00030 as seed ortholog is 99%.
```

---

### Group of orthologs #557. Best score 404 bits Score difference with first non-orthologous sequence - AM1\_locus\_tags.txt:404 OB3b\_locus\_tags.txt:404

```
META1_2835          	100.00%		CQW49_RS10345       	100.00%
Bootstrap support for META1_2835 as seed ortholog is 100%.
Bootstrap support for CQW49_RS10345 as seed ortholog is 100%.
```

---

### Group of orthologs #558. Best score 404 bits Score difference with first non-orthologous sequence - AM1\_locus\_tags.txt:286 OB3b\_locus\_tags.txt:404

```
META1_2846          	100.00%		CQW49_RS20450       	100.00%
Bootstrap support for META1_2846 as seed ortholog is 100%.
Bootstrap support for CQW49_RS20450 as seed ortholog is 100%.
```

---

### Group of orthologs #559. Best score 404 bits Score difference with first non-orthologous sequence - AM1\_locus\_tags.txt:305 OB3b\_locus\_tags.txt:326

```
META2_0869          	100.00%		CQW49_RS05640       	100.00%
Bootstrap support for META2_0869 as seed ortholog is 100%.
Bootstrap support for CQW49_RS05640 as seed ortholog is 100%.
```

---

### Group of orthologs #560. Best score 403 bits Score difference with first non-orthologous sequence - AM1\_locus\_tags.txt:403 OB3b\_locus\_tags.txt:403

```
META1_0647          	100.00%		CQW49_RS15570       	100.00%
Bootstrap support for META1_0647 as seed ortholog is 100%.
Bootstrap support for CQW49_RS15570 as seed ortholog is 100%.
```

---

### Group of orthologs #561. Best score 403 bits Score difference with first non-orthologous sequence - AM1\_locus\_tags.txt:141 OB3b\_locus\_tags.txt:252

```
META1_4871          	100.00%		CQW49_RS04455       	100.00%
Bootstrap support for META1_4871 as seed ortholog is 99%.
Bootstrap support for CQW49_RS04455 as seed ortholog is 100%.
```

---

### Group of orthologs #562. Best score 403 bits Score difference with first non-orthologous sequence - AM1\_locus\_tags.txt:350 OB3b\_locus\_tags.txt:337

```
META1_4251          	100.00%		CQW49_RS18890       	100.00%
Bootstrap support for META1_4251 as seed ortholog is 100%.
Bootstrap support for CQW49_RS18890 as seed ortholog is 100%.
```

---

### Group of orthologs #563. Best score 402 bits Score difference with first non-orthologous sequence - AM1\_locus\_tags.txt:331 OB3b\_locus\_tags.txt:352

```
META1_4689          	100.00%		CQW49_RS16880       	100.00%
Bootstrap support for META1_4689 as seed ortholog is 100%.
Bootstrap support for CQW49_RS16880 as seed ortholog is 100%.
```

---

### Group of orthologs #564. Best score 401 bits Score difference with first non-orthologous sequence - AM1\_locus\_tags.txt:177 OB3b\_locus\_tags.txt:401

```
META1_2067          	100.00%		CQW49_RS00460       	100.00%
Bootstrap support for META1_2067 as seed ortholog is 100%.
Bootstrap support for CQW49_RS00460 as seed ortholog is 100%.
```

---

### Group of orthologs #565. Best score 401 bits Score difference with first non-orthologous sequence - AM1\_locus\_tags.txt:151 OB3b\_locus\_tags.txt:401

```
META1_2452          	100.00%		CQW49_RS09370       	100.00%
Bootstrap support for META1_2452 as seed ortholog is 99%.
Bootstrap support for CQW49_RS09370 as seed ortholog is 100%.
```

---

### Group of orthologs #566. Best score 401 bits Score difference with first non-orthologous sequence - AM1\_locus\_tags.txt:401 OB3b\_locus\_tags.txt:401

```
META1_1493          	100.00%		CQW49_RS19120       	100.00%
Bootstrap support for META1_1493 as seed ortholog is 100%.
Bootstrap support for CQW49_RS19120 as seed ortholog is 100%.
```

---

### Group of orthologs #567. Best score 400 bits Score difference with first non-orthologous sequence - AM1\_locus\_tags.txt:400 OB3b\_locus\_tags.txt:400

```
META1_2363          	100.00%		CQW49_RS04130       	100.00%
Bootstrap support for META1_2363 as seed ortholog is 100%.
Bootstrap support for CQW49_RS04130 as seed ortholog is 100%.
```

---

### Group of orthologs #568. Best score 400 bits Score difference with first non-orthologous sequence - AM1\_locus\_tags.txt:400 OB3b\_locus\_tags.txt:400

```
META1_0758          	100.00%		CQW49_RS18465       	100.00%
Bootstrap support for META1_0758 as seed ortholog is 100%.
Bootstrap support for CQW49_RS18465 as seed ortholog is 100%.
```

---

### Group of orthologs #569. Best score 399 bits Score difference with first non-orthologous sequence - AM1\_locus\_tags.txt:399 OB3b\_locus\_tags.txt:399

```
META1_1748          	100.00%		CQW49_RS03365       	100.00%
Bootstrap support for META1_1748 as seed ortholog is 100%.
Bootstrap support for CQW49_RS03365 as seed ortholog is 100%.
```

---

### Group of orthologs #570. Best score 399 bits Score difference with first non-orthologous sequence - AM1\_locus\_tags.txt:399 OB3b\_locus\_tags.txt:322

```
META1_2536          	100.00%		CQW49_RS01755       	100.00%
Bootstrap support for META1_2536 as seed ortholog is 100%.
Bootstrap support for CQW49_RS01755 as seed ortholog is 100%.
```

---

### Group of orthologs #571. Best score 399 bits Score difference with first non-orthologous sequence - AM1\_locus\_tags.txt:399 OB3b\_locus\_tags.txt:399

```
META1_2159          	100.00%		CQW49_RS10490       	100.00%
Bootstrap support for META1_2159 as seed ortholog is 100%.
Bootstrap support for CQW49_RS10490 as seed ortholog is 100%.
```

---

### Group of orthologs #572. Best score 399 bits Score difference with first non-orthologous sequence - AM1\_locus\_tags.txt:399 OB3b\_locus\_tags.txt:399

```
META1_3376          	100.00%		CQW49_RS08265       	100.00%
Bootstrap support for META1_3376 as seed ortholog is 100%.
Bootstrap support for CQW49_RS08265 as seed ortholog is 100%.
```

---

### Group of orthologs #573. Best score 399 bits Score difference with first non-orthologous sequence - AM1\_locus\_tags.txt:399 OB3b\_locus\_tags.txt:30

```
META1_3863          	100.00%		CQW49_RS10855       	100.00%
Bootstrap support for META1_3863 as seed ortholog is 100%.
Bootstrap support for CQW49_RS10855 as seed ortholog is 82%.
```

---

### Group of orthologs #574. Best score 399 bits Score difference with first non-orthologous sequence - AM1\_locus\_tags.txt:348 OB3b\_locus\_tags.txt:399

```
META1_4889          	100.00%		CQW49_RS15460       	100.00%
Bootstrap support for META1_4889 as seed ortholog is 100%.
Bootstrap support for CQW49_RS15460 as seed ortholog is 100%.
```

---

### Group of orthologs #575. Best score 398 bits Score difference with first non-orthologous sequence - AM1\_locus\_tags.txt:398 OB3b\_locus\_tags.txt:398

```
META1_1330          	100.00%		CQW49_RS17900       	100.00%
Bootstrap support for META1_1330 as seed ortholog is 100%.
Bootstrap support for CQW49_RS17900 as seed ortholog is 100%.
```

---

### Group of orthologs #576. Best score 398 bits Score difference with first non-orthologous sequence - AM1\_locus\_tags.txt:58 OB3b\_locus\_tags.txt:398

```
META1_1779          	100.00%		CQW49_RS19330       	100.00%
Bootstrap support for META1_1779 as seed ortholog is 98%.
Bootstrap support for CQW49_RS19330 as seed ortholog is 100%.
```

---

### Group of orthologs #577. Best score 397 bits Score difference with first non-orthologous sequence - AM1\_locus\_tags.txt:397 OB3b\_locus\_tags.txt:397

```
META1_2345          	100.00%		CQW49_RS08625       	100.00%
Bootstrap support for META1_2345 as seed ortholog is 100%.
Bootstrap support for CQW49_RS08625 as seed ortholog is 100%.
```

---

### Group of orthologs #578. Best score 397 bits Score difference with first non-orthologous sequence - AM1\_locus\_tags.txt:397 OB3b\_locus\_tags.txt:397

```
META1_3174          	100.00%		CQW49_RS17660       	100.00%
Bootstrap support for META1_3174 as seed ortholog is 100%.
Bootstrap support for CQW49_RS17660 as seed ortholog is 100%.
```

---

### Group of orthologs #579. Best score 396 bits Score difference with first non-orthologous sequence - AM1\_locus\_tags.txt:396 OB3b\_locus\_tags.txt:396

```
META1_3530          	100.00%		CQW49_RS01075       	100.00%
Bootstrap support for META1_3530 as seed ortholog is 100%.
Bootstrap support for CQW49_RS01075 as seed ortholog is 100%.
```

---

### Group of orthologs #580. Best score 396 bits Score difference with first non-orthologous sequence - AM1\_locus\_tags.txt:396 OB3b\_locus\_tags.txt:10

```
META2_0527          	100.00%		CQW49_RS23115       	100.00%
Bootstrap support for META2_0527 as seed ortholog is 100%.
Bootstrap support for CQW49_RS23115 as seed ortholog is 60%.
Alternative seed ortholog is CQW49_RS21375 (10 bits away from this cluster)
```

---

### Group of orthologs #581. Best score 395 bits Score difference with first non-orthologous sequence - AM1\_locus\_tags.txt:395 OB3b\_locus\_tags.txt:395

```
META1_1526          	100.00%		CQW49_RS16100       	100.00%
Bootstrap support for META1_1526 as seed ortholog is 100%.
Bootstrap support for CQW49_RS16100 as seed ortholog is 100%.
```

---

### Group of orthologs #582. Best score 395 bits Score difference with first non-orthologous sequence - AM1\_locus\_tags.txt:395 OB3b\_locus\_tags.txt:395

```
META1_1724          	100.00%		CQW49_RS16085       	100.00%
Bootstrap support for META1_1724 as seed ortholog is 100%.
Bootstrap support for CQW49_RS16085 as seed ortholog is 100%.
```

---

### Group of orthologs #583. Best score 395 bits Score difference with first non-orthologous sequence - AM1\_locus\_tags.txt:395 OB3b\_locus\_tags.txt:395

```
META1_5127          	100.00%		CQW49_RS00485       	100.00%
Bootstrap support for META1_5127 as seed ortholog is 100%.
Bootstrap support for CQW49_RS00485 as seed ortholog is 100%.
```

---

### Group of orthologs #584. Best score 394 bits Score difference with first non-orthologous sequence - AM1\_locus\_tags.txt:394 OB3b\_locus\_tags.txt:394

```
META1_0469          	100.00%		CQW49_RS18780       	100.00%
Bootstrap support for META1_0469 as seed ortholog is 100%.
Bootstrap support for CQW49_RS18780 as seed ortholog is 100%.
```

---

### Group of orthologs #585. Best score 393 bits Score difference with first non-orthologous sequence - AM1\_locus\_tags.txt:393 OB3b\_locus\_tags.txt:156

```
META1_1441          	100.00%		CQW49_RS13015       	100.00%
Bootstrap support for META1_1441 as seed ortholog is 100%.
Bootstrap support for CQW49_RS13015 as seed ortholog is 99%.
```

---

### Group of orthologs #586. Best score 393 bits Score difference with first non-orthologous sequence - AM1\_locus\_tags.txt:393 OB3b\_locus\_tags.txt:314

```
META1_2661          	100.00%		CQW49_RS19925       	100.00%
Bootstrap support for META1_2661 as seed ortholog is 100%.
Bootstrap support for CQW49_RS19925 as seed ortholog is 100%.
```

---

### Group of orthologs #587. Best score 391 bits Score difference with first non-orthologous sequence - AM1\_locus\_tags.txt:391 OB3b\_locus\_tags.txt:391

```
META1_0286          	100.00%		CQW49_RS20375       	100.00%
META2_0327          	9.06%		
Bootstrap support for META1_0286 as seed ortholog is 100%.
Bootstrap support for CQW49_RS20375 as seed ortholog is 100%.
```

---

### Group of orthologs #588. Best score 391 bits Score difference with first non-orthologous sequence - AM1\_locus\_tags.txt:391 OB3b\_locus\_tags.txt:391

```
META1_0562          	100.00%		CQW49_RS12865       	100.00%
Bootstrap support for META1_0562 as seed ortholog is 100%.
Bootstrap support for CQW49_RS12865 as seed ortholog is 100%.
```

---

### Group of orthologs #589. Best score 391 bits Score difference with first non-orthologous sequence - AM1\_locus\_tags.txt:288 OB3b\_locus\_tags.txt:252

```
META1_0376          	100.00%		CQW49_RS17725       	100.00%
Bootstrap support for META1_0376 as seed ortholog is 100%.
Bootstrap support for CQW49_RS17725 as seed ortholog is 100%.
```

---

### Group of orthologs #590. Best score 391 bits Score difference with first non-orthologous sequence - AM1\_locus\_tags.txt:296 OB3b\_locus\_tags.txt:273

```
META1_1435          	100.00%		CQW49_RS20305       	100.00%
Bootstrap support for META1_1435 as seed ortholog is 100%.
Bootstrap support for CQW49_RS20305 as seed ortholog is 100%.
```

---

### Group of orthologs #591. Best score 390 bits Score difference with first non-orthologous sequence - AM1\_locus\_tags.txt:390 OB3b\_locus\_tags.txt:390

```
META1_0232          	100.00%		CQW49_RS04595       	100.00%
Bootstrap support for META1_0232 as seed ortholog is 100%.
Bootstrap support for CQW49_RS04595 as seed ortholog is 100%.
```

---

### Group of orthologs #592. Best score 390 bits Score difference with first non-orthologous sequence - AM1\_locus\_tags.txt:281 OB3b\_locus\_tags.txt:156

```
META1_0809          	100.00%		CQW49_RS15845       	100.00%
Bootstrap support for META1_0809 as seed ortholog is 100%.
Bootstrap support for CQW49_RS15845 as seed ortholog is 99%.
```

---

### Group of orthologs #593. Best score 390 bits Score difference with first non-orthologous sequence - AM1\_locus\_tags.txt:390 OB3b\_locus\_tags.txt:390

```
META1_1363          	100.00%		CQW49_RS13565       	100.00%
Bootstrap support for META1_1363 as seed ortholog is 100%.
Bootstrap support for CQW49_RS13565 as seed ortholog is 100%.
```

---

### Group of orthologs #594. Best score 390 bits Score difference with first non-orthologous sequence - AM1\_locus\_tags.txt:390 OB3b\_locus\_tags.txt:390

```
META2_0254          	100.00%		CQW49_RS14575       	100.00%
Bootstrap support for META2_0254 as seed ortholog is 100%.
Bootstrap support for CQW49_RS14575 as seed ortholog is 100%.
```

---

### Group of orthologs #595. Best score 389 bits Score difference with first non-orthologous sequence - AM1\_locus\_tags.txt:389 OB3b\_locus\_tags.txt:389

```
META1_2095          	100.00%		CQW49_RS09305       	100.00%
Bootstrap support for META1_2095 as seed ortholog is 100%.
Bootstrap support for CQW49_RS09305 as seed ortholog is 100%.
```

---

### Group of orthologs #596. Best score 389 bits Score difference with first non-orthologous sequence - AM1\_locus\_tags.txt:389 OB3b\_locus\_tags.txt:389

```
META1_3497          	100.00%		CQW49_RS13185       	100.00%
Bootstrap support for META1_3497 as seed ortholog is 100%.
Bootstrap support for CQW49_RS13185 as seed ortholog is 100%.
```

---

### Group of orthologs #597. Best score 388 bits Score difference with first non-orthologous sequence - AM1\_locus\_tags.txt:153 OB3b\_locus\_tags.txt:388

```
META1_0077          	100.00%		CQW49_RS09405       	100.00%
META1_2712          	13.32%		
Bootstrap support for META1_0077 as seed ortholog is 99%.
Bootstrap support for CQW49_RS09405 as seed ortholog is 100%.
```

---

### Group of orthologs #598. Best score 388 bits Score difference with first non-orthologous sequence - AM1\_locus\_tags.txt:388 OB3b\_locus\_tags.txt:388

```
META1_3018          	100.00%		CQW49_RS01680       	100.00%
Bootstrap support for META1_3018 as seed ortholog is 100%.
Bootstrap support for CQW49_RS01680 as seed ortholog is 100%.
```

---

### Group of orthologs #599. Best score 386 bits Score difference with first non-orthologous sequence - AM1\_locus\_tags.txt:325 OB3b\_locus\_tags.txt:386

```
META1_3392          	100.00%		CQW49_RS04810       	100.00%
Bootstrap support for META1_3392 as seed ortholog is 100%.
Bootstrap support for CQW49_RS04810 as seed ortholog is 100%.
```

---

### Group of orthologs #600. Best score 384 bits Score difference with first non-orthologous sequence - AM1\_locus\_tags.txt:384 OB3b\_locus\_tags.txt:384

```
META1_5164          	100.00%		CQW49_RS15965       	100.00%
                    	       		CQW49_RS06775       	34.08%
Bootstrap support for META1_5164 as seed ortholog is 100%.
Bootstrap support for CQW49_RS15965 as seed ortholog is 100%.
```

---

### Group of orthologs #601. Best score 384 bits Score difference with first non-orthologous sequence - AM1\_locus\_tags.txt:384 OB3b\_locus\_tags.txt:384

```
META1_2200          	100.00%		CQW49_RS16350       	100.00%
Bootstrap support for META1_2200 as seed ortholog is 100%.
Bootstrap support for CQW49_RS16350 as seed ortholog is 100%.
```

---

### Group of orthologs #602. Best score 384 bits Score difference with first non-orthologous sequence - AM1\_locus\_tags.txt:323 OB3b\_locus\_tags.txt:384

```
META1_3517          	100.00%		CQW49_RS18595       	100.00%
Bootstrap support for META1_3517 as seed ortholog is 100%.
Bootstrap support for CQW49_RS18595 as seed ortholog is 100%.
```

---

### Group of orthologs #603. Best score 384 bits Score difference with first non-orthologous sequence - AM1\_locus\_tags.txt:264 OB3b\_locus\_tags.txt:325

```
META1_5071          	100.00%		CQW49_RS14250       	100.00%
Bootstrap support for META1_5071 as seed ortholog is 100%.
Bootstrap support for CQW49_RS14250 as seed ortholog is 100%.
```

---

### Group of orthologs #604. Best score 383 bits Score difference with first non-orthologous sequence - AM1\_locus\_tags.txt:383 OB3b\_locus\_tags.txt:383

```
META1_0587          	100.00%		CQW49_RS04235       	100.00%
Bootstrap support for META1_0587 as seed ortholog is 100%.
Bootstrap support for CQW49_RS04235 as seed ortholog is 100%.
```

---

### Group of orthologs #605. Best score 382 bits Score difference with first non-orthologous sequence - AM1\_locus\_tags.txt:382 OB3b\_locus\_tags.txt:257

```
META1_1791          	100.00%		CQW49_RS04685       	100.00%
Bootstrap support for META1_1791 as seed ortholog is 100%.
Bootstrap support for CQW49_RS04685 as seed ortholog is 100%.
```

---

### Group of orthologs #606. Best score 382 bits Score difference with first non-orthologous sequence - AM1\_locus\_tags.txt:309 OB3b\_locus\_tags.txt:282

```
META1_1476          	100.00%		CQW49_RS12790       	100.00%
Bootstrap support for META1_1476 as seed ortholog is 100%.
Bootstrap support for CQW49_RS12790 as seed ortholog is 100%.
```

---

### Group of orthologs #607. Best score 381 bits Score difference with first non-orthologous sequence - AM1\_locus\_tags.txt:381 OB3b\_locus\_tags.txt:381

```
META1_2065          	100.00%		CQW49_RS00470       	100.00%
Bootstrap support for META1_2065 as seed ortholog is 100%.
Bootstrap support for CQW49_RS00470 as seed ortholog is 100%.
```

---

### Group of orthologs #608. Best score 381 bits Score difference with first non-orthologous sequence - AM1\_locus\_tags.txt:330 OB3b\_locus\_tags.txt:328

```
META1_5014          	100.00%		CQW49_RS01915       	100.00%
Bootstrap support for META1_5014 as seed ortholog is 100%.
Bootstrap support for CQW49_RS01915 as seed ortholog is 100%.
```

---

### Group of orthologs #609. Best score 381 bits Score difference with first non-orthologous sequence - AM1\_locus\_tags.txt:381 OB3b\_locus\_tags.txt:381

```
META1_1529          	100.00%		CQW49_RS19195       	100.00%
Bootstrap support for META1_1529 as seed ortholog is 100%.
Bootstrap support for CQW49_RS19195 as seed ortholog is 100%.
```

---

### Group of orthologs #610. Best score 380 bits Score difference with first non-orthologous sequence - AM1\_locus\_tags.txt:380 OB3b\_locus\_tags.txt:380

```
META1_2339          	100.00%		CQW49_RS13150       	100.00%
Bootstrap support for META1_2339 as seed ortholog is 100%.
Bootstrap support for CQW49_RS13150 as seed ortholog is 100%.
```

---

### Group of orthologs #611. Best score 380 bits Score difference with first non-orthologous sequence - AM1\_locus\_tags.txt:313 OB3b\_locus\_tags.txt:380

```
META1_5318          	100.00%		CQW49_RS12760       	100.00%
Bootstrap support for META1_5318 as seed ortholog is 100%.
Bootstrap support for CQW49_RS12760 as seed ortholog is 100%.
```

---

### Group of orthologs #612. Best score 379 bits Score difference with first non-orthologous sequence - AM1\_locus\_tags.txt:379 OB3b\_locus\_tags.txt:379

```
META1_1397          	100.00%		CQW49_RS00880       	100.00%
Bootstrap support for META1_1397 as seed ortholog is 100%.
Bootstrap support for CQW49_RS00880 as seed ortholog is 100%.
```

---

### Group of orthologs #613. Best score 379 bits Score difference with first non-orthologous sequence - AM1\_locus\_tags.txt:379 OB3b\_locus\_tags.txt:379

```
META1_2153          	100.00%		CQW49_RS10520       	100.00%
Bootstrap support for META1_2153 as seed ortholog is 100%.
Bootstrap support for CQW49_RS10520 as seed ortholog is 100%.
```

---

### Group of orthologs #614. Best score 379 bits Score difference with first non-orthologous sequence - AM1\_locus\_tags.txt:240 OB3b\_locus\_tags.txt:273

```
META1_5182          	100.00%		CQW49_RS03385       	100.00%
Bootstrap support for META1_5182 as seed ortholog is 100%.
Bootstrap support for CQW49_RS03385 as seed ortholog is 100%.
```

---

### Group of orthologs #615. Best score 379 bits Score difference with first non-orthologous sequence - AM1\_locus\_tags.txt:379 OB3b\_locus\_tags.txt:379

```
META1_3211          	100.00%		CQW49_RS15500       	100.00%
Bootstrap support for META1_3211 as seed ortholog is 100%.
Bootstrap support for CQW49_RS15500 as seed ortholog is 100%.
```

---

### Group of orthologs #616. Best score 378 bits Score difference with first non-orthologous sequence - AM1\_locus\_tags.txt:108 OB3b\_locus\_tags.txt:378

```
META1_2456          	100.00%		CQW49_RS06900       	100.00%
Bootstrap support for META1_2456 as seed ortholog is 99%.
Bootstrap support for CQW49_RS06900 as seed ortholog is 100%.
```

---

### Group of orthologs #617. Best score 378 bits Score difference with first non-orthologous sequence - AM1\_locus\_tags.txt:378 OB3b\_locus\_tags.txt:378

```
META1_2518          	100.00%		CQW49_RS07625       	100.00%
Bootstrap support for META1_2518 as seed ortholog is 100%.
Bootstrap support for CQW49_RS07625 as seed ortholog is 100%.
```

---

### Group of orthologs #618. Best score 378 bits Score difference with first non-orthologous sequence - AM1\_locus\_tags.txt:378 OB3b\_locus\_tags.txt:378

```
META1_2519          	100.00%		CQW49_RS07640       	100.00%
Bootstrap support for META1_2519 as seed ortholog is 100%.
Bootstrap support for CQW49_RS07640 as seed ortholog is 100%.
```

---

### Group of orthologs #619. Best score 378 bits Score difference with first non-orthologous sequence - AM1\_locus\_tags.txt:249 OB3b\_locus\_tags.txt:378

```
META1_2331          	100.00%		CQW49_RS09065       	100.00%
Bootstrap support for META1_2331 as seed ortholog is 100%.
Bootstrap support for CQW49_RS09065 as seed ortholog is 100%.
```

---

### Group of orthologs #620. Best score 378 bits Score difference with first non-orthologous sequence - AM1\_locus\_tags.txt:378 OB3b\_locus\_tags.txt:378

```
META1_2981          	100.00%		CQW49_RS11760       	100.00%
Bootstrap support for META1_2981 as seed ortholog is 100%.
Bootstrap support for CQW49_RS11760 as seed ortholog is 100%.
```

---

### Group of orthologs #621. Best score 378 bits Score difference with first non-orthologous sequence - AM1\_locus\_tags.txt:225 OB3b\_locus\_tags.txt:100

```
META1_3027          	100.00%		CQW49_RS20795       	100.00%
Bootstrap support for META1_3027 as seed ortholog is 100%.
Bootstrap support for CQW49_RS20795 as seed ortholog is 98%.
```

---

### Group of orthologs #622. Best score 378 bits Score difference with first non-orthologous sequence - AM1\_locus\_tags.txt:378 OB3b\_locus\_tags.txt:378

```
META1_4623          	100.00%		CQW49_RS18000       	100.00%
Bootstrap support for META1_4623 as seed ortholog is 100%.
Bootstrap support for CQW49_RS18000 as seed ortholog is 100%.
```

---

### Group of orthologs #623. Best score 377 bits Score difference with first non-orthologous sequence - AM1\_locus\_tags.txt:377 OB3b\_locus\_tags.txt:377

```
META1_3147          	100.00%		CQW49_RS11260       	100.00%
Bootstrap support for META1_3147 as seed ortholog is 100%.
Bootstrap support for CQW49_RS11260 as seed ortholog is 100%.
```

---

### Group of orthologs #624. Best score 377 bits Score difference with first non-orthologous sequence - AM1\_locus\_tags.txt:275 OB3b\_locus\_tags.txt:377

```
META2_1139          	100.00%		CQW49_RS11785       	100.00%
Bootstrap support for META2_1139 as seed ortholog is 100%.
Bootstrap support for CQW49_RS11785 as seed ortholog is 100%.
```

---

### Group of orthologs #625. Best score 377 bits Score difference with first non-orthologous sequence - AM1\_locus\_tags.txt:377 OB3b\_locus\_tags.txt:377

```
META1_5120          	100.00%		CQW49_RS20250       	100.00%
Bootstrap support for META1_5120 as seed ortholog is 100%.
Bootstrap support for CQW49_RS20250 as seed ortholog is 100%.
```

---

### Group of orthologs #626. Best score 376 bits Score difference with first non-orthologous sequence - AM1\_locus\_tags.txt:311 OB3b\_locus\_tags.txt:376

```
META1_4531          	100.00%		CQW49_RS14500       	100.00%
Bootstrap support for META1_4531 as seed ortholog is 100%.
Bootstrap support for CQW49_RS14500 as seed ortholog is 100%.
```

---

### Group of orthologs #627. Best score 375 bits Score difference with first non-orthologous sequence - AM1\_locus\_tags.txt:375 OB3b\_locus\_tags.txt:375

```
META1_1728          	100.00%		CQW49_RS12245       	100.00%
Bootstrap support for META1_1728 as seed ortholog is 100%.
Bootstrap support for CQW49_RS12245 as seed ortholog is 100%.
```

---

### Group of orthologs #628. Best score 375 bits Score difference with first non-orthologous sequence - AM1\_locus\_tags.txt:375 OB3b\_locus\_tags.txt:189

```
META1_4598          	100.00%		CQW49_RS06115       	100.00%
Bootstrap support for META1_4598 as seed ortholog is 100%.
Bootstrap support for CQW49_RS06115 as seed ortholog is 99%.
```

---

### Group of orthologs #629. Best score 374 bits Score difference with first non-orthologous sequence - AM1\_locus\_tags.txt:374 OB3b\_locus\_tags.txt:374

```
META1_1725          	100.00%		CQW49_RS16080       	100.00%
Bootstrap support for META1_1725 as seed ortholog is 100%.
Bootstrap support for CQW49_RS16080 as seed ortholog is 100%.
```

---

### Group of orthologs #630. Best score 374 bits Score difference with first non-orthologous sequence - AM1\_locus\_tags.txt:248 OB3b\_locus\_tags.txt:259

```
META1_2465          	100.00%		CQW49_RS13320       	100.00%
Bootstrap support for META1_2465 as seed ortholog is 100%.
Bootstrap support for CQW49_RS13320 as seed ortholog is 100%.
```

---

### Group of orthologs #631. Best score 373 bits Score difference with first non-orthologous sequence - AM1\_locus\_tags.txt:373 OB3b\_locus\_tags.txt:373

```
META1_1360          	100.00%		CQW49_RS00300       	100.00%
Bootstrap support for META1_1360 as seed ortholog is 100%.
Bootstrap support for CQW49_RS00300 as seed ortholog is 100%.
```

---

### Group of orthologs #632. Best score 373 bits Score difference with first non-orthologous sequence - AM1\_locus\_tags.txt:373 OB3b\_locus\_tags.txt:373

```
META1_1471          	100.00%		CQW49_RS12805       	100.00%
Bootstrap support for META1_1471 as seed ortholog is 100%.
Bootstrap support for CQW49_RS12805 as seed ortholog is 100%.
```

---

### Group of orthologs #633. Best score 373 bits Score difference with first non-orthologous sequence - AM1\_locus\_tags.txt:130 OB3b\_locus\_tags.txt:373

```
META1_1520          	100.00%		CQW49_RS19870       	100.00%
Bootstrap support for META1_1520 as seed ortholog is 99%.
Bootstrap support for CQW49_RS19870 as seed ortholog is 100%.
```

---

### Group of orthologs #634. Best score 373 bits Score difference with first non-orthologous sequence - AM1\_locus\_tags.txt:236 OB3b\_locus\_tags.txt:294

```
META1_5167          	100.00%		CQW49_RS15980       	100.00%
Bootstrap support for META1_5167 as seed ortholog is 100%.
Bootstrap support for CQW49_RS15980 as seed ortholog is 100%.
```

---

### Group of orthologs #635. Best score 372 bits Score difference with first non-orthologous sequence - AM1\_locus\_tags.txt:1 OB3b\_locus\_tags.txt:372

```
META1_2748          	100.00%		CQW49_RS12280       	100.00%
Bootstrap support for META1_2748 as seed ortholog is 49%.
Alternative seed ortholog is META1_2944 (1 bits away from this cluster)
Bootstrap support for CQW49_RS12280 as seed ortholog is 100%.
```

---

### Group of orthologs #636. Best score 370 bits Score difference with first non-orthologous sequence - AM1\_locus\_tags.txt:241 OB3b\_locus\_tags.txt:242

```
META1_0660          	100.00%		CQW49_RS03145       	100.00%
Bootstrap support for META1_0660 as seed ortholog is 100%.
Bootstrap support for CQW49_RS03145 as seed ortholog is 100%.
```

---

### Group of orthologs #637. Best score 369 bits Score difference with first non-orthologous sequence - AM1\_locus\_tags.txt:64 OB3b\_locus\_tags.txt:168

```
META1_2599          	100.00%		CQW49_RS05010       	100.00%
META2_0201          	75.36%		
Bootstrap support for META1_2599 as seed ortholog is 93%.
Bootstrap support for CQW49_RS05010 as seed ortholog is 99%.
```

---

### Group of orthologs #638. Best score 369 bits Score difference with first non-orthologous sequence - AM1\_locus\_tags.txt:369 OB3b\_locus\_tags.txt:369

```
META1_1389          	100.00%		CQW49_RS02690       	100.00%
Bootstrap support for META1_1389 as seed ortholog is 100%.
Bootstrap support for CQW49_RS02690 as seed ortholog is 100%.
```

---

### Group of orthologs #639. Best score 369 bits Score difference with first non-orthologous sequence - AM1\_locus\_tags.txt:369 OB3b\_locus\_tags.txt:369

```
META1_3472          	100.00%		CQW49_RS10060       	100.00%
Bootstrap support for META1_3472 as seed ortholog is 100%.
Bootstrap support for CQW49_RS10060 as seed ortholog is 100%.
```

---

### Group of orthologs #640. Best score 369 bits Score difference with first non-orthologous sequence - AM1\_locus\_tags.txt:369 OB3b\_locus\_tags.txt:369

```
META1_5104          	100.00%		CQW49_RS03440       	100.00%
Bootstrap support for META1_5104 as seed ortholog is 100%.
Bootstrap support for CQW49_RS03440 as seed ortholog is 100%.
```

---

### Group of orthologs #641. Best score 368 bits Score difference with first non-orthologous sequence - AM1\_locus\_tags.txt:368 OB3b\_locus\_tags.txt:368

```
META1_0321          	100.00%		CQW49_RS06945       	100.00%
Bootstrap support for META1_0321 as seed ortholog is 100%.
Bootstrap support for CQW49_RS06945 as seed ortholog is 100%.
```

---

### Group of orthologs #642. Best score 368 bits Score difference with first non-orthologous sequence - AM1\_locus\_tags.txt:368 OB3b\_locus\_tags.txt:368

```
META1_2289          	100.00%		CQW49_RS19630       	100.00%
Bootstrap support for META1_2289 as seed ortholog is 100%.
Bootstrap support for CQW49_RS19630 as seed ortholog is 100%.
```

---

### Group of orthologs #643. Best score 368 bits Score difference with first non-orthologous sequence - AM1\_locus\_tags.txt:274 OB3b\_locus\_tags.txt:166

```
META1_4228          	100.00%		CQW49_RS11390       	100.00%
Bootstrap support for META1_4228 as seed ortholog is 100%.
Bootstrap support for CQW49_RS11390 as seed ortholog is 99%.
```

---

### Group of orthologs #644. Best score 368 bits Score difference with first non-orthologous sequence - AM1\_locus\_tags.txt:368 OB3b\_locus\_tags.txt:368

```
META1_4890          	100.00%		CQW49_RS15455       	100.00%
Bootstrap support for META1_4890 as seed ortholog is 100%.
Bootstrap support for CQW49_RS15455 as seed ortholog is 100%.
```

---

### Group of orthologs #645. Best score 367 bits Score difference with first non-orthologous sequence - AM1\_locus\_tags.txt:205 OB3b\_locus\_tags.txt:247

```
META1_0525          	100.00%		CQW49_RS06130       	100.00%
Bootstrap support for META1_0525 as seed ortholog is 100%.
Bootstrap support for CQW49_RS06130 as seed ortholog is 100%.
```

---

### Group of orthologs #646. Best score 367 bits Score difference with first non-orthologous sequence - AM1\_locus\_tags.txt:210 OB3b\_locus\_tags.txt:256

```
META1_0377          	100.00%		CQW49_RS17735       	100.00%
Bootstrap support for META1_0377 as seed ortholog is 100%.
Bootstrap support for CQW49_RS17735 as seed ortholog is 100%.
```

---

### Group of orthologs #647. Best score 367 bits Score difference with first non-orthologous sequence - AM1\_locus\_tags.txt:367 OB3b\_locus\_tags.txt:367

```
META1_0218          	100.00%		CQW49_RS20085       	100.00%
Bootstrap support for META1_0218 as seed ortholog is 100%.
Bootstrap support for CQW49_RS20085 as seed ortholog is 100%.
```

---

### Group of orthologs #648. Best score 366 bits Score difference with first non-orthologous sequence - AM1\_locus\_tags.txt:304 OB3b\_locus\_tags.txt:277

```
META1_0404          	100.00%		CQW49_RS18535       	100.00%
Bootstrap support for META1_0404 as seed ortholog is 100%.
Bootstrap support for CQW49_RS18535 as seed ortholog is 100%.
```

---

### Group of orthologs #649. Best score 366 bits Score difference with first non-orthologous sequence - AM1\_locus\_tags.txt:170 OB3b\_locus\_tags.txt:201

```
META1_0406          	100.00%		CQW49_RS18545       	100.00%
Bootstrap support for META1_0406 as seed ortholog is 100%.
Bootstrap support for CQW49_RS18545 as seed ortholog is 100%.
```

---

### Group of orthologs #650. Best score 366 bits Score difference with first non-orthologous sequence - AM1\_locus\_tags.txt:366 OB3b\_locus\_tags.txt:366

```
META1_4640          	100.00%		CQW49_RS04135       	100.00%
Bootstrap support for META1_4640 as seed ortholog is 100%.
Bootstrap support for CQW49_RS04135 as seed ortholog is 100%.
```

---

### Group of orthologs #651. Best score 364 bits Score difference with first non-orthologous sequence - AM1\_locus\_tags.txt:364 OB3b\_locus\_tags.txt:364

```
META1_2397          	100.00%		CQW49_RS06240       	100.00%
META1_4039          	82.30%		
Bootstrap support for META1_2397 as seed ortholog is 100%.
Bootstrap support for CQW49_RS06240 as seed ortholog is 100%.
```

---

### Group of orthologs #652. Best score 364 bits Score difference with first non-orthologous sequence - AM1\_locus\_tags.txt:364 OB3b\_locus\_tags.txt:364

```
META1_2851          	100.00%		CQW49_RS02720       	100.00%
Bootstrap support for META1_2851 as seed ortholog is 100%.
Bootstrap support for CQW49_RS02720 as seed ortholog is 100%.
```

---

### Group of orthologs #653. Best score 364 bits Score difference with first non-orthologous sequence - AM1\_locus\_tags.txt:364 OB3b\_locus\_tags.txt:364

```
META1_4920          	100.00%		CQW49_RS20100       	100.00%
Bootstrap support for META1_4920 as seed ortholog is 100%.
Bootstrap support for CQW49_RS20100 as seed ortholog is 100%.
```

---

### Group of orthologs #654. Best score 363 bits Score difference with first non-orthologous sequence - AM1\_locus\_tags.txt:363 OB3b\_locus\_tags.txt:363

```
META1_0317          	100.00%		CQW49_RS18835       	100.00%
Bootstrap support for META1_0317 as seed ortholog is 100%.
Bootstrap support for CQW49_RS18835 as seed ortholog is 100%.
```

---

### Group of orthologs #655. Best score 363 bits Score difference with first non-orthologous sequence - AM1\_locus\_tags.txt:363 OB3b\_locus\_tags.txt:271

```
META1_1434          	100.00%		CQW49_RS20300       	100.00%
Bootstrap support for META1_1434 as seed ortholog is 100%.
Bootstrap support for CQW49_RS20300 as seed ortholog is 100%.
```

---

### Group of orthologs #656. Best score 362 bits Score difference with first non-orthologous sequence - AM1\_locus\_tags.txt:362 OB3b\_locus\_tags.txt:362

```
META1_0168          	100.00%		CQW49_RS05365       	100.00%
Bootstrap support for META1_0168 as seed ortholog is 100%.
Bootstrap support for CQW49_RS05365 as seed ortholog is 100%.
```

---

### Group of orthologs #657. Best score 362 bits Score difference with first non-orthologous sequence - AM1\_locus\_tags.txt:292 OB3b\_locus\_tags.txt:302

```
META1_4363          	100.00%		CQW49_RS01410       	100.00%
Bootstrap support for META1_4363 as seed ortholog is 100%.
Bootstrap support for CQW49_RS01410 as seed ortholog is 100%.
```

---

### Group of orthologs #658. Best score 362 bits Score difference with first non-orthologous sequence - AM1\_locus\_tags.txt:229 OB3b\_locus\_tags.txt:362

```
META1_0509          	100.00%		CQW49_RS19740       	100.00%
Bootstrap support for META1_0509 as seed ortholog is 99%.
Bootstrap support for CQW49_RS19740 as seed ortholog is 100%.
```

---

### Group of orthologs #659. Best score 362 bits Score difference with first non-orthologous sequence - AM1\_locus\_tags.txt:285 OB3b\_locus\_tags.txt:309

```
META1_4850          	100.00%		CQW49_RS16680       	100.00%
Bootstrap support for META1_4850 as seed ortholog is 100%.
Bootstrap support for CQW49_RS16680 as seed ortholog is 100%.
```

---

### Group of orthologs #660. Best score 361 bits Score difference with first non-orthologous sequence - AM1\_locus\_tags.txt:361 OB3b\_locus\_tags.txt:361

```
META1_3193          	100.00%		CQW49_RS01440       	100.00%
Bootstrap support for META1_3193 as seed ortholog is 100%.
Bootstrap support for CQW49_RS01440 as seed ortholog is 100%.
```

---

### Group of orthologs #661. Best score 361 bits Score difference with first non-orthologous sequence - AM1\_locus\_tags.txt:361 OB3b\_locus\_tags.txt:361

```
META1_3403          	100.00%		CQW49_RS13435       	100.00%
Bootstrap support for META1_3403 as seed ortholog is 100%.
Bootstrap support for CQW49_RS13435 as seed ortholog is 100%.
```

---

### Group of orthologs #662. Best score 361 bits Score difference with first non-orthologous sequence - AM1\_locus\_tags.txt:361 OB3b\_locus\_tags.txt:361

```
META1_3457          	100.00%		CQW49_RS15520       	100.00%
Bootstrap support for META1_3457 as seed ortholog is 100%.
Bootstrap support for CQW49_RS15520 as seed ortholog is 100%.
```

---

### Group of orthologs #663. Best score 360 bits Score difference with first non-orthologous sequence - AM1\_locus\_tags.txt:260 OB3b\_locus\_tags.txt:73

```
META1_1833          	100.00%		CQW49_RS17795       	100.00%
META1_3826          	15.65%		
META1_3741          	10.33%		
Bootstrap support for META1_1833 as seed ortholog is 100%.
Bootstrap support for CQW49_RS17795 as seed ortholog is 98%.
```

---

### Group of orthologs #664. Best score 360 bits Score difference with first non-orthologous sequence - AM1\_locus\_tags.txt:217 OB3b\_locus\_tags.txt:192

```
META1_1298          	100.00%		CQW49_RS03975       	100.00%
Bootstrap support for META1_1298 as seed ortholog is 100%.
Bootstrap support for CQW49_RS03975 as seed ortholog is 100%.
```

---

### Group of orthologs #665. Best score 360 bits Score difference with first non-orthologous sequence - AM1\_locus\_tags.txt:360 OB3b\_locus\_tags.txt:360

```
META1_1763          	100.00%		CQW49_RS13530       	100.00%
Bootstrap support for META1_1763 as seed ortholog is 100%.
Bootstrap support for CQW49_RS13530 as seed ortholog is 100%.
```

---

### Group of orthologs #666. Best score 360 bits Score difference with first non-orthologous sequence - AM1\_locus\_tags.txt:360 OB3b\_locus\_tags.txt:360

```
META1_4386          	100.00%		CQW49_RS11370       	100.00%
Bootstrap support for META1_4386 as seed ortholog is 100%.
Bootstrap support for CQW49_RS11370 as seed ortholog is 100%.
```

---

### Group of orthologs #667. Best score 358 bits Score difference with first non-orthologous sequence - AM1\_locus\_tags.txt:358 OB3b\_locus\_tags.txt:358

```
META1_0556          	100.00%		CQW49_RS11635       	100.00%
Bootstrap support for META1_0556 as seed ortholog is 100%.
Bootstrap support for CQW49_RS11635 as seed ortholog is 100%.
```

---

### Group of orthologs #668. Best score 358 bits Score difference with first non-orthologous sequence - AM1\_locus\_tags.txt:276 OB3b\_locus\_tags.txt:302

```
META1_1175          	100.00%		CQW49_RS09425       	100.00%
Bootstrap support for META1_1175 as seed ortholog is 100%.
Bootstrap support for CQW49_RS09425 as seed ortholog is 100%.
```

---

### Group of orthologs #669. Best score 358 bits Score difference with first non-orthologous sequence - AM1\_locus\_tags.txt:358 OB3b\_locus\_tags.txt:358

```
META1_4359          	100.00%		CQW49_RS01425       	100.00%
Bootstrap support for META1_4359 as seed ortholog is 100%.
Bootstrap support for CQW49_RS01425 as seed ortholog is 100%.
```

---

### Group of orthologs #670. Best score 358 bits Score difference with first non-orthologous sequence - AM1\_locus\_tags.txt:358 OB3b\_locus\_tags.txt:358

```
META1_1987          	100.00%		CQW49_RS13085       	100.00%
Bootstrap support for META1_1987 as seed ortholog is 100%.
Bootstrap support for CQW49_RS13085 as seed ortholog is 100%.
```

---

### Group of orthologs #671. Best score 358 bits Score difference with first non-orthologous sequence - AM1\_locus\_tags.txt:358 OB3b\_locus\_tags.txt:358

```
META1_0484          	100.00%		CQW49_RS20320       	100.00%
Bootstrap support for META1_0484 as seed ortholog is 100%.
Bootstrap support for CQW49_RS20320 as seed ortholog is 100%.
```

---

### Group of orthologs #672. Best score 358 bits Score difference with first non-orthologous sequence - AM1\_locus\_tags.txt:358 OB3b\_locus\_tags.txt:358

```
META1_2058          	100.00%		CQW49_RS15695       	100.00%
Bootstrap support for META1_2058 as seed ortholog is 100%.
Bootstrap support for CQW49_RS15695 as seed ortholog is 100%.
```

---

### Group of orthologs #673. Best score 356 bits Score difference with first non-orthologous sequence - AM1\_locus\_tags.txt:356 OB3b\_locus\_tags.txt:356

```
META1_1988          	100.00%		CQW49_RS16335       	100.00%
                    	       		CQW49_RS16340       	45.38%
Bootstrap support for META1_1988 as seed ortholog is 100%.
Bootstrap support for CQW49_RS16335 as seed ortholog is 100%.
```

---

### Group of orthologs #674. Best score 356 bits Score difference with first non-orthologous sequence - AM1\_locus\_tags.txt:356 OB3b\_locus\_tags.txt:356

```
META1_0659          	100.00%		CQW49_RS03150       	100.00%
Bootstrap support for META1_0659 as seed ortholog is 100%.
Bootstrap support for CQW49_RS03150 as seed ortholog is 100%.
```

---

### Group of orthologs #675. Best score 356 bits Score difference with first non-orthologous sequence - AM1\_locus\_tags.txt:356 OB3b\_locus\_tags.txt:356

```
META1_2949          	100.00%		CQW49_RS12485       	100.00%
Bootstrap support for META1_2949 as seed ortholog is 100%.
Bootstrap support for CQW49_RS12485 as seed ortholog is 100%.
```

---

### Group of orthologs #676. Best score 356 bits Score difference with first non-orthologous sequence - AM1\_locus\_tags.txt:356 OB3b\_locus\_tags.txt:356

```
META1_3662          	100.00%		CQW49_RS09450       	100.00%
Bootstrap support for META1_3662 as seed ortholog is 100%.
Bootstrap support for CQW49_RS09450 as seed ortholog is 100%.
```

---

### Group of orthologs #677. Best score 356 bits Score difference with first non-orthologous sequence - AM1\_locus\_tags.txt:356 OB3b\_locus\_tags.txt:288

```
META1_3381          	100.00%		CQW49_RS19575       	100.00%
Bootstrap support for META1_3381 as seed ortholog is 100%.
Bootstrap support for CQW49_RS19575 as seed ortholog is 100%.
```

---

### Group of orthologs #678. Best score 355 bits Score difference with first non-orthologous sequence - AM1\_locus\_tags.txt:355 OB3b\_locus\_tags.txt:355

```
META1_0706          	100.00%		CQW49_RS00855       	100.00%
Bootstrap support for META1_0706 as seed ortholog is 100%.
Bootstrap support for CQW49_RS00855 as seed ortholog is 100%.
```

---

### Group of orthologs #679. Best score 355 bits Score difference with first non-orthologous sequence - AM1\_locus\_tags.txt:355 OB3b\_locus\_tags.txt:355

```
META1_0622          	100.00%		CQW49_RS13400       	100.00%
Bootstrap support for META1_0622 as seed ortholog is 100%.
Bootstrap support for CQW49_RS13400 as seed ortholog is 100%.
```

---

### Group of orthologs #680. Best score 355 bits Score difference with first non-orthologous sequence - AM1\_locus\_tags.txt:355 OB3b\_locus\_tags.txt:355

```
META1_1783          	100.00%		CQW49_RS19320       	100.00%
Bootstrap support for META1_1783 as seed ortholog is 100%.
Bootstrap support for CQW49_RS19320 as seed ortholog is 100%.
```

---

### Group of orthologs #681. Best score 355 bits Score difference with first non-orthologous sequence - AM1\_locus\_tags.txt:355 OB3b\_locus\_tags.txt:355

```
META1_5090          	100.00%		CQW49_RS11220       	100.00%
Bootstrap support for META1_5090 as seed ortholog is 100%.
Bootstrap support for CQW49_RS11220 as seed ortholog is 100%.
```

---

### Group of orthologs #682. Best score 354 bits Score difference with first non-orthologous sequence - AM1\_locus\_tags.txt:354 OB3b\_locus\_tags.txt:266

```
META1_2989          	100.00%		CQW49_RS01945       	100.00%
Bootstrap support for META1_2989 as seed ortholog is 100%.
Bootstrap support for CQW49_RS01945 as seed ortholog is 100%.
```

---

### Group of orthologs #683. Best score 354 bits Score difference with first non-orthologous sequence - AM1\_locus\_tags.txt:354 OB3b\_locus\_tags.txt:354

```
META1_4925          	100.00%		CQW49_RS11585       	100.00%
Bootstrap support for META1_4925 as seed ortholog is 100%.
Bootstrap support for CQW49_RS11585 as seed ortholog is 100%.
```

---

### Group of orthologs #684. Best score 354 bits Score difference with first non-orthologous sequence - AM1\_locus\_tags.txt:188 OB3b\_locus\_tags.txt:203

```
META1_4280          	100.00%		CQW49_RS18275       	100.00%
Bootstrap support for META1_4280 as seed ortholog is 100%.
Bootstrap support for CQW49_RS18275 as seed ortholog is 100%.
```

---

### Group of orthologs #685. Best score 353 bits Score difference with first non-orthologous sequence - AM1\_locus\_tags.txt:143 OB3b\_locus\_tags.txt:203

```
META1_0223          	100.00%		CQW49_RS19490       	100.00%
Bootstrap support for META1_0223 as seed ortholog is 100%.
Bootstrap support for CQW49_RS19490 as seed ortholog is 100%.
```

---

### Group of orthologs #686. Best score 353 bits Score difference with first non-orthologous sequence - AM1\_locus\_tags.txt:294 OB3b\_locus\_tags.txt:353

```
META1_4809          	100.00%		CQW49_RS15635       	100.00%
Bootstrap support for META1_4809 as seed ortholog is 100%.
Bootstrap support for CQW49_RS15635 as seed ortholog is 100%.
```

---

### Group of orthologs #687. Best score 351 bits Score difference with first non-orthologous sequence - AM1\_locus\_tags.txt:168 OB3b\_locus\_tags.txt:272

```
META1_0925          	100.00%		CQW49_RS07230       	100.00%
Bootstrap support for META1_0925 as seed ortholog is 100%.
Bootstrap support for CQW49_RS07230 as seed ortholog is 100%.
```

---

### Group of orthologs #688. Best score 351 bits Score difference with first non-orthologous sequence - AM1\_locus\_tags.txt:351 OB3b\_locus\_tags.txt:351

```
META1_1010          	100.00%		CQW49_RS10155       	100.00%
Bootstrap support for META1_1010 as seed ortholog is 100%.
Bootstrap support for CQW49_RS10155 as seed ortholog is 100%.
```

---

### Group of orthologs #689. Best score 351 bits Score difference with first non-orthologous sequence - AM1\_locus\_tags.txt:351 OB3b\_locus\_tags.txt:351

```
META1_0783          	100.00%		CQW49_RS15575       	100.00%
Bootstrap support for META1_0783 as seed ortholog is 100%.
Bootstrap support for CQW49_RS15575 as seed ortholog is 100%.
```

---

### Group of orthologs #690. Best score 351 bits Score difference with first non-orthologous sequence - AM1\_locus\_tags.txt:178 OB3b\_locus\_tags.txt:207

```
META1_3844          	100.00%		CQW49_RS05920       	100.00%
Bootstrap support for META1_3844 as seed ortholog is 100%.
Bootstrap support for CQW49_RS05920 as seed ortholog is 100%.
```

---

### Group of orthologs #691. Best score 351 bits Score difference with first non-orthologous sequence - AM1\_locus\_tags.txt:351 OB3b\_locus\_tags.txt:351

```
META1_4495          	100.00%		CQW49_RS05885       	100.00%
Bootstrap support for META1_4495 as seed ortholog is 100%.
Bootstrap support for CQW49_RS05885 as seed ortholog is 100%.
```

---

### Group of orthologs #692. Best score 350 bits Score difference with first non-orthologous sequence - AM1\_locus\_tags.txt:279 OB3b\_locus\_tags.txt:350

```
META1_1814          	100.00%		CQW49_RS00140       	100.00%
Bootstrap support for META1_1814 as seed ortholog is 100%.
Bootstrap support for CQW49_RS00140 as seed ortholog is 100%.
```

---

### Group of orthologs #693. Best score 350 bits Score difference with first non-orthologous sequence - AM1\_locus\_tags.txt:350 OB3b\_locus\_tags.txt:350

```
META1_0760          	100.00%		CQW49_RS18270       	100.00%
Bootstrap support for META1_0760 as seed ortholog is 100%.
Bootstrap support for CQW49_RS18270 as seed ortholog is 100%.
```

---

### Group of orthologs #694. Best score 350 bits Score difference with first non-orthologous sequence - AM1\_locus\_tags.txt:350 OB3b\_locus\_tags.txt:350

```
META1_1492          	100.00%		CQW49_RS19125       	100.00%
Bootstrap support for META1_1492 as seed ortholog is 100%.
Bootstrap support for CQW49_RS19125 as seed ortholog is 100%.
```

---

### Group of orthologs #695. Best score 349 bits Score difference with first non-orthologous sequence - AM1\_locus\_tags.txt:349 OB3b\_locus\_tags.txt:230

```
p1METAp0007         	100.00%		CQW49_RS22805       	100.00%
                    	       		CQW49_RS14005       	43.97%
                    	       		CQW49_RS15255       	42.45%
                    	       		CQW49_RS14555       	28.58%
Bootstrap support for p1METAp0007 as seed ortholog is 100%.
Bootstrap support for CQW49_RS22805 as seed ortholog is 100%.
```

---

### Group of orthologs #696. Best score 349 bits Score difference with first non-orthologous sequence - AM1\_locus\_tags.txt:272 OB3b\_locus\_tags.txt:298

```
META1_0342          	100.00%		CQW49_RS09340       	100.00%
Bootstrap support for META1_0342 as seed ortholog is 100%.
Bootstrap support for CQW49_RS09340 as seed ortholog is 100%.
```

---

### Group of orthologs #697. Best score 349 bits Score difference with first non-orthologous sequence - AM1\_locus\_tags.txt:349 OB3b\_locus\_tags.txt:349

```
META1_5243          	100.00%		CQW49_RS20290       	100.00%
Bootstrap support for META1_5243 as seed ortholog is 100%.
Bootstrap support for CQW49_RS20290 as seed ortholog is 100%.
```

---

### Group of orthologs #698. Best score 348 bits Score difference with first non-orthologous sequence - AM1\_locus\_tags.txt:348 OB3b\_locus\_tags.txt:348

```
META1_3320          	100.00%		CQW49_RS19185       	100.00%
Bootstrap support for META1_3320 as seed ortholog is 100%.
Bootstrap support for CQW49_RS19185 as seed ortholog is 100%.
```

---

### Group of orthologs #699. Best score 348 bits Score difference with first non-orthologous sequence - AM1\_locus\_tags.txt:348 OB3b\_locus\_tags.txt:348

```
META1_4529          	100.00%		CQW49_RS14510       	100.00%
Bootstrap support for META1_4529 as seed ortholog is 100%.
Bootstrap support for CQW49_RS14510 as seed ortholog is 100%.
```

---

### Group of orthologs #700. Best score 347 bits Score difference with first non-orthologous sequence - AM1\_locus\_tags.txt:255 OB3b\_locus\_tags.txt:347

```
META1_0426          	100.00%		CQW49_RS01160       	100.00%
Bootstrap support for META1_0426 as seed ortholog is 100%.
Bootstrap support for CQW49_RS01160 as seed ortholog is 100%.
```

---

### Group of orthologs #701. Best score 347 bits Score difference with first non-orthologous sequence - AM1\_locus\_tags.txt:91 OB3b\_locus\_tags.txt:29

```
META1_2100          	100.00%		CQW49_RS00250       	100.00%
Bootstrap support for META1_2100 as seed ortholog is 99%.
Bootstrap support for CQW49_RS00250 as seed ortholog is 87%.
```

---

### Group of orthologs #702. Best score 347 bits Score difference with first non-orthologous sequence - AM1\_locus\_tags.txt:347 OB3b\_locus\_tags.txt:347

```
META1_3090          	100.00%		CQW49_RS12210       	100.00%
Bootstrap support for META1_3090 as seed ortholog is 100%.
Bootstrap support for CQW49_RS12210 as seed ortholog is 100%.
```

---

### Group of orthologs #703. Best score 346 bits Score difference with first non-orthologous sequence - AM1\_locus\_tags.txt:346 OB3b\_locus\_tags.txt:346

```
META1_1756          	100.00%		CQW49_RS03845       	100.00%
Bootstrap support for META1_1756 as seed ortholog is 100%.
Bootstrap support for CQW49_RS03845 as seed ortholog is 100%.
```

---

### Group of orthologs #704. Best score 346 bits Score difference with first non-orthologous sequence - AM1\_locus\_tags.txt:98 OB3b\_locus\_tags.txt:346

```
META1_3345          	100.00%		CQW49_RS01540       	100.00%
Bootstrap support for META1_3345 as seed ortholog is 99%.
Bootstrap support for CQW49_RS01540 as seed ortholog is 100%.
```

---

### Group of orthologs #705. Best score 346 bits Score difference with first non-orthologous sequence - AM1\_locus\_tags.txt:346 OB3b\_locus\_tags.txt:346

```
META2_0576          	100.00%		CQW49_RS07600       	100.00%
Bootstrap support for META2_0576 as seed ortholog is 100%.
Bootstrap support for CQW49_RS07600 as seed ortholog is 100%.
```

---

### Group of orthologs #706. Best score 346 bits Score difference with first non-orthologous sequence - AM1\_locus\_tags.txt:346 OB3b\_locus\_tags.txt:250

```
META1_4537          	100.00%		CQW49_RS14465       	100.00%
Bootstrap support for META1_4537 as seed ortholog is 100%.
Bootstrap support for CQW49_RS14465 as seed ortholog is 100%.
```

---

### Group of orthologs #707. Best score 344 bits Score difference with first non-orthologous sequence - AM1\_locus\_tags.txt:344 OB3b\_locus\_tags.txt:344

```
META1_0766          	100.00%		CQW49_RS00490       	100.00%
Bootstrap support for META1_0766 as seed ortholog is 100%.
Bootstrap support for CQW49_RS00490 as seed ortholog is 100%.
```

---

### Group of orthologs #708. Best score 344 bits Score difference with first non-orthologous sequence - AM1\_locus\_tags.txt:131 OB3b\_locus\_tags.txt:228

```
META1_2478          	100.00%		CQW49_RS16410       	100.00%
Bootstrap support for META1_2478 as seed ortholog is 100%.
Bootstrap support for CQW49_RS16410 as seed ortholog is 100%.
```

---

### Group of orthologs #709. Best score 344 bits Score difference with first non-orthologous sequence - AM1\_locus\_tags.txt:344 OB3b\_locus\_tags.txt:344

```
META1_4709          	100.00%		CQW49_RS10090       	100.00%
Bootstrap support for META1_4709 as seed ortholog is 100%.
Bootstrap support for CQW49_RS10090 as seed ortholog is 100%.
```

---

### Group of orthologs #710. Best score 344 bits Score difference with first non-orthologous sequence - AM1\_locus\_tags.txt:344 OB3b\_locus\_tags.txt:344

```
META1_5190          	100.00%		CQW49_RS16985       	100.00%
Bootstrap support for META1_5190 as seed ortholog is 100%.
Bootstrap support for CQW49_RS16985 as seed ortholog is 100%.
```

---

### Group of orthologs #711. Best score 343 bits Score difference with first non-orthologous sequence - AM1\_locus\_tags.txt:343 OB3b\_locus\_tags.txt:343

```
META1_0424          	100.00%		CQW49_RS11430       	100.00%
Bootstrap support for META1_0424 as seed ortholog is 100%.
Bootstrap support for CQW49_RS11430 as seed ortholog is 100%.
```

---

### Group of orthologs #712. Best score 343 bits Score difference with first non-orthologous sequence - AM1\_locus\_tags.txt:87 OB3b\_locus\_tags.txt:343

```
META1_0447          	100.00%		CQW49_RS18820       	100.00%
Bootstrap support for META1_0447 as seed ortholog is 99%.
Bootstrap support for CQW49_RS18820 as seed ortholog is 100%.
```

---

### Group of orthologs #713. Best score 343 bits Score difference with first non-orthologous sequence - AM1\_locus\_tags.txt:343 OB3b\_locus\_tags.txt:343

```
META1_2888          	100.00%		CQW49_RS11200       	100.00%
Bootstrap support for META1_2888 as seed ortholog is 100%.
Bootstrap support for CQW49_RS11200 as seed ortholog is 100%.
```

---

### Group of orthologs #714. Best score 342 bits Score difference with first non-orthologous sequence - AM1\_locus\_tags.txt:267 OB3b\_locus\_tags.txt:253

```
META1_2925          	100.00%		CQW49_RS13370       	100.00%
Bootstrap support for META1_2925 as seed ortholog is 100%.
Bootstrap support for CQW49_RS13370 as seed ortholog is 100%.
```

---

### Group of orthologs #715. Best score 342 bits Score difference with first non-orthologous sequence - AM1\_locus\_tags.txt:115 OB3b\_locus\_tags.txt:171

```
META1_4585          	100.00%		CQW49_RS20585       	100.00%
Bootstrap support for META1_4585 as seed ortholog is 99%.
Bootstrap support for CQW49_RS20585 as seed ortholog is 100%.
```

---

### Group of orthologs #716. Best score 341 bits Score difference with first non-orthologous sequence - AM1\_locus\_tags.txt:341 OB3b\_locus\_tags.txt:341

```
META1_3895          	100.00%		CQW49_RS11345       	100.00%
Bootstrap support for META1_3895 as seed ortholog is 100%.
Bootstrap support for CQW49_RS11345 as seed ortholog is 100%.
```

---

### Group of orthologs #717. Best score 341 bits Score difference with first non-orthologous sequence - AM1\_locus\_tags.txt:248 OB3b\_locus\_tags.txt:341

```
META1_4255          	100.00%		CQW49_RS18600       	100.00%
Bootstrap support for META1_4255 as seed ortholog is 100%.
Bootstrap support for CQW49_RS18600 as seed ortholog is 100%.
```

---

### Group of orthologs #718. Best score 341 bits Score difference with first non-orthologous sequence - AM1\_locus\_tags.txt:341 OB3b\_locus\_tags.txt:341

```
META1_4636          	100.00%		CQW49_RS17480       	100.00%
Bootstrap support for META1_4636 as seed ortholog is 100%.
Bootstrap support for CQW49_RS17480 as seed ortholog is 100%.
```

---

### Group of orthologs #719. Best score 340 bits Score difference with first non-orthologous sequence - AM1\_locus\_tags.txt:340 OB3b\_locus\_tags.txt:340

```
META1_1356          	100.00%		CQW49_RS20945       	100.00%
Bootstrap support for META1_1356 as seed ortholog is 100%.
Bootstrap support for CQW49_RS20945 as seed ortholog is 100%.
```

---

### Group of orthologs #720. Best score 340 bits Score difference with first non-orthologous sequence - AM1\_locus\_tags.txt:245 OB3b\_locus\_tags.txt:223

```
META1_2882          	100.00%		CQW49_RS15960       	100.00%
Bootstrap support for META1_2882 as seed ortholog is 100%.
Bootstrap support for CQW49_RS15960 as seed ortholog is 100%.
```

---

### Group of orthologs #721. Best score 340 bits Score difference with first non-orthologous sequence - AM1\_locus\_tags.txt:340 OB3b\_locus\_tags.txt:340

```
META1_4319          	100.00%		CQW49_RS10820       	100.00%
Bootstrap support for META1_4319 as seed ortholog is 100%.
Bootstrap support for CQW49_RS10820 as seed ortholog is 100%.
```

---

### Group of orthologs #722. Best score 339 bits Score difference with first non-orthologous sequence - AM1\_locus\_tags.txt:31 OB3b\_locus\_tags.txt:339

```
META1_0383          	100.00%		CQW49_RS15800       	100.00%
Bootstrap support for META1_0383 as seed ortholog is 81%.
Bootstrap support for CQW49_RS15800 as seed ortholog is 100%.
```

---

### Group of orthologs #723. Best score 338 bits Score difference with first non-orthologous sequence - AM1\_locus\_tags.txt:237 OB3b\_locus\_tags.txt:261

```
META1_3872          	100.00%		CQW49_RS04970       	100.00%
Bootstrap support for META1_3872 as seed ortholog is 100%.
Bootstrap support for CQW49_RS04970 as seed ortholog is 100%.
```

---

### Group of orthologs #724. Best score 338 bits Score difference with first non-orthologous sequence - AM1\_locus\_tags.txt:338 OB3b\_locus\_tags.txt:338

```
META1_4620          	100.00%		CQW49_RS03025       	100.00%
Bootstrap support for META1_4620 as seed ortholog is 100%.
Bootstrap support for CQW49_RS03025 as seed ortholog is 100%.
```

---

### Group of orthologs #725. Best score 337 bits Score difference with first non-orthologous sequence - AM1\_locus\_tags.txt:337 OB3b\_locus\_tags.txt:236

```
META1_0854          	100.00%		CQW49_RS05350       	100.00%
Bootstrap support for META1_0854 as seed ortholog is 100%.
Bootstrap support for CQW49_RS05350 as seed ortholog is 100%.
```

---

### Group of orthologs #726. Best score 337 bits Score difference with first non-orthologous sequence - AM1\_locus\_tags.txt:337 OB3b\_locus\_tags.txt:337

```
META1_3906          	100.00%		CQW49_RS05045       	100.00%
Bootstrap support for META1_3906 as seed ortholog is 100%.
Bootstrap support for CQW49_RS05045 as seed ortholog is 100%.
```

---

### Group of orthologs #727. Best score 336 bits Score difference with first non-orthologous sequence - AM1\_locus\_tags.txt:167 OB3b\_locus\_tags.txt:4

```
META1_2486          	100.00%		CQW49_RS22190       	100.00%
                    	       		CQW49_RS13685       	12.30%
Bootstrap support for META1_2486 as seed ortholog is 99%.
Bootstrap support for CQW49_RS22190 as seed ortholog is 46%.
Alternative seed ortholog is CQW49_RS08375 (4 bits away from this cluster)
```

---

### Group of orthologs #728. Best score 336 bits Score difference with first non-orthologous sequence - AM1\_locus\_tags.txt:336 OB3b\_locus\_tags.txt:336

```
META1_4626          	100.00%		CQW49_RS02195       	100.00%
Bootstrap support for META1_4626 as seed ortholog is 100%.
Bootstrap support for CQW49_RS02195 as seed ortholog is 100%.
```

---

### Group of orthologs #729. Best score 336 bits Score difference with first non-orthologous sequence - AM1\_locus\_tags.txt:336 OB3b\_locus\_tags.txt:336

```
META1_2325          	100.00%		CQW49_RS15395       	100.00%
Bootstrap support for META1_2325 as seed ortholog is 100%.
Bootstrap support for CQW49_RS15395 as seed ortholog is 100%.
```

---

### Group of orthologs #730. Best score 335 bits Score difference with first non-orthologous sequence - AM1\_locus\_tags.txt:335 OB3b\_locus\_tags.txt:335

```
META1_0320          	100.00%		CQW49_RS06950       	100.00%
Bootstrap support for META1_0320 as seed ortholog is 100%.
Bootstrap support for CQW49_RS06950 as seed ortholog is 100%.
```

---

### Group of orthologs #731. Best score 335 bits Score difference with first non-orthologous sequence - AM1\_locus\_tags.txt:335 OB3b\_locus\_tags.txt:335

```
META1_4859          	100.00%		CQW49_RS04465       	100.00%
Bootstrap support for META1_4859 as seed ortholog is 100%.
Bootstrap support for CQW49_RS04465 as seed ortholog is 100%.
```

---

### Group of orthologs #732. Best score 335 bits Score difference with first non-orthologous sequence - AM1\_locus\_tags.txt:186 OB3b\_locus\_tags.txt:176

```
META1_5169          	100.00%		CQW49_RS15990       	100.00%
Bootstrap support for META1_5169 as seed ortholog is 100%.
Bootstrap support for CQW49_RS15990 as seed ortholog is 100%.
```

---

### Group of orthologs #733. Best score 334 bits Score difference with first non-orthologous sequence - AM1\_locus\_tags.txt:264 OB3b\_locus\_tags.txt:270

```
META1_1789          	100.00%		CQW49_RS04695       	100.00%
Bootstrap support for META1_1789 as seed ortholog is 100%.
Bootstrap support for CQW49_RS04695 as seed ortholog is 100%.
```

---

### Group of orthologs #734. Best score 334 bits Score difference with first non-orthologous sequence - AM1\_locus\_tags.txt:192 OB3b\_locus\_tags.txt:221

```
META1_3701          	100.00%		CQW49_RS17800       	100.00%
Bootstrap support for META1_3701 as seed ortholog is 100%.
Bootstrap support for CQW49_RS17800 as seed ortholog is 100%.
```

---

### Group of orthologs #735. Best score 333 bits Score difference with first non-orthologous sequence - AM1\_locus\_tags.txt:333 OB3b\_locus\_tags.txt:333

```
META1_2502          	100.00%		CQW49_RS19470       	100.00%
META1_3692          	86.47%		CQW49_RS00130       	79.95%
Bootstrap support for META1_2502 as seed ortholog is 100%.
Bootstrap support for CQW49_RS19470 as seed ortholog is 100%.
```

---

### Group of orthologs #736. Best score 332 bits Score difference with first non-orthologous sequence - AM1\_locus\_tags.txt:332 OB3b\_locus\_tags.txt:332

```
META1_0804          	100.00%		CQW49_RS09640       	100.00%
Bootstrap support for META1_0804 as seed ortholog is 100%.
Bootstrap support for CQW49_RS09640 as seed ortholog is 100%.
```

---

### Group of orthologs #737. Best score 332 bits Score difference with first non-orthologous sequence - AM1\_locus\_tags.txt:332 OB3b\_locus\_tags.txt:332

```
META1_2450          	100.00%		CQW49_RS17430       	100.00%
Bootstrap support for META1_2450 as seed ortholog is 100%.
Bootstrap support for CQW49_RS17430 as seed ortholog is 100%.
```

---

### Group of orthologs #738. Best score 332 bits Score difference with first non-orthologous sequence - AM1\_locus\_tags.txt:212 OB3b\_locus\_tags.txt:332

```
META1_2417          	100.00%		CQW49_RS20900       	100.00%
Bootstrap support for META1_2417 as seed ortholog is 100%.
Bootstrap support for CQW49_RS20900 as seed ortholog is 100%.
```

---

### Group of orthologs #739. Best score 331 bits Score difference with first non-orthologous sequence - AM1\_locus\_tags.txt:331 OB3b\_locus\_tags.txt:331

```
META1_0369          	100.00%		CQW49_RS09075       	100.00%
Bootstrap support for META1_0369 as seed ortholog is 100%.
Bootstrap support for CQW49_RS09075 as seed ortholog is 100%.
```

---

### Group of orthologs #740. Best score 331 bits Score difference with first non-orthologous sequence - AM1\_locus\_tags.txt:169 OB3b\_locus\_tags.txt:241

```
META1_2530          	100.00%		CQW49_RS06445       	100.00%
Bootstrap support for META1_2530 as seed ortholog is 99%.
Bootstrap support for CQW49_RS06445 as seed ortholog is 99%.
```

---

### Group of orthologs #741. Best score 331 bits Score difference with first non-orthologous sequence - AM1\_locus\_tags.txt:84 OB3b\_locus\_tags.txt:67

```
META1_3249          	100.00%		CQW49_RS06880       	100.00%
Bootstrap support for META1_3249 as seed ortholog is 97%.
Bootstrap support for CQW49_RS06880 as seed ortholog is 97%.
```

---

### Group of orthologs #742. Best score 331 bits Score difference with first non-orthologous sequence - AM1\_locus\_tags.txt:331 OB3b\_locus\_tags.txt:331

```
META1_2948          	100.00%		CQW49_RS10250       	100.00%
Bootstrap support for META1_2948 as seed ortholog is 100%.
Bootstrap support for CQW49_RS10250 as seed ortholog is 100%.
```

---

### Group of orthologs #743. Best score 331 bits Score difference with first non-orthologous sequence - AM1\_locus\_tags.txt:331 OB3b\_locus\_tags.txt:78

```
META1_2845          	100.00%		CQW49_RS16910       	100.00%
Bootstrap support for META1_2845 as seed ortholog is 100%.
Bootstrap support for CQW49_RS16910 as seed ortholog is 98%.
```

---

### Group of orthologs #744. Best score 331 bits Score difference with first non-orthologous sequence - AM1\_locus\_tags.txt:331 OB3b\_locus\_tags.txt:331

```
META1_2537          	100.00%		CQW49_RS20755       	100.00%
Bootstrap support for META1_2537 as seed ortholog is 100%.
Bootstrap support for CQW49_RS20755 as seed ortholog is 100%.
```

---

### Group of orthologs #745. Best score 330 bits Score difference with first non-orthologous sequence - AM1\_locus\_tags.txt:139 OB3b\_locus\_tags.txt:330

```
META1_2070          	100.00%		CQW49_RS00445       	100.00%
Bootstrap support for META1_2070 as seed ortholog is 100%.
Bootstrap support for CQW49_RS00445 as seed ortholog is 100%.
```

---

### Group of orthologs #746. Best score 330 bits Score difference with first non-orthologous sequence - AM1\_locus\_tags.txt:259 OB3b\_locus\_tags.txt:330

```
META1_3106          	100.00%		CQW49_RS02095       	100.00%
Bootstrap support for META1_3106 as seed ortholog is 100%.
Bootstrap support for CQW49_RS02095 as seed ortholog is 100%.
```

---

### Group of orthologs #747. Best score 329 bits Score difference with first non-orthologous sequence - AM1\_locus\_tags.txt:262 OB3b\_locus\_tags.txt:329

```
META1_5048          	100.00%		CQW49_RS03075       	100.00%
Bootstrap support for META1_5048 as seed ortholog is 100%.
Bootstrap support for CQW49_RS03075 as seed ortholog is 100%.
```

---

### Group of orthologs #748. Best score 329 bits Score difference with first non-orthologous sequence - AM1\_locus\_tags.txt:183 OB3b\_locus\_tags.txt:329

```
META1_2496          	100.00%		CQW49_RS16555       	100.00%
Bootstrap support for META1_2496 as seed ortholog is 100%.
Bootstrap support for CQW49_RS16555 as seed ortholog is 100%.
```

---

### Group of orthologs #749. Best score 329 bits Score difference with first non-orthologous sequence - AM1\_locus\_tags.txt:329 OB3b\_locus\_tags.txt:329

```
META1_3646          	100.00%		CQW49_RS12660       	100.00%
Bootstrap support for META1_3646 as seed ortholog is 100%.
Bootstrap support for CQW49_RS12660 as seed ortholog is 100%.
```

---

### Group of orthologs #750. Best score 329 bits Score difference with first non-orthologous sequence - AM1\_locus\_tags.txt:329 OB3b\_locus\_tags.txt:329

```
META1_5165          	100.00%		CQW49_RS15970       	100.00%
Bootstrap support for META1_5165 as seed ortholog is 100%.
Bootstrap support for CQW49_RS15970 as seed ortholog is 100%.
```

---

### Group of orthologs #751. Best score 329 bits Score difference with first non-orthologous sequence - AM1\_locus\_tags.txt:329 OB3b\_locus\_tags.txt:329

```
META1_5121          	100.00%		CQW49_RS20245       	100.00%
Bootstrap support for META1_5121 as seed ortholog is 100%.
Bootstrap support for CQW49_RS20245 as seed ortholog is 100%.
```

---

### Group of orthologs #752. Best score 327 bits Score difference with first non-orthologous sequence - AM1\_locus\_tags.txt:221 OB3b\_locus\_tags.txt:327

```
META1_0053          	100.00%		CQW49_RS04195       	100.00%
Bootstrap support for META1_0053 as seed ortholog is 100%.
Bootstrap support for CQW49_RS04195 as seed ortholog is 100%.
```

---

### Group of orthologs #753. Best score 327 bits Score difference with first non-orthologous sequence - AM1\_locus\_tags.txt:327 OB3b\_locus\_tags.txt:327

```
META1_0954          	100.00%		CQW49_RS22065       	100.00%
Bootstrap support for META1_0954 as seed ortholog is 100%.
Bootstrap support for CQW49_RS22065 as seed ortholog is 100%.
```

---

### Group of orthologs #754. Best score 327 bits Score difference with first non-orthologous sequence - AM1\_locus\_tags.txt:327 OB3b\_locus\_tags.txt:327

```
META1_5132          	100.00%		CQW49_RS09100       	100.00%
Bootstrap support for META1_5132 as seed ortholog is 100%.
Bootstrap support for CQW49_RS09100 as seed ortholog is 100%.
```

---

### Group of orthologs #755. Best score 326 bits Score difference with first non-orthologous sequence - AM1\_locus\_tags.txt:25 OB3b\_locus\_tags.txt:232

```
META1_3199          	100.00%		CQW49_RS06320       	100.00%
                    	       		CQW49_RS19535       	18.18%
Bootstrap support for META1_3199 as seed ortholog is 81%.
Bootstrap support for CQW49_RS06320 as seed ortholog is 100%.
```

---

### Group of orthologs #756. Best score 325 bits Score difference with first non-orthologous sequence - AM1\_locus\_tags.txt:325 OB3b\_locus\_tags.txt:51

```
META1_1491          	100.00%		CQW49_RS03380       	100.00%
Bootstrap support for META1_1491 as seed ortholog is 100%.
Bootstrap support for CQW49_RS03380 as seed ortholog is 92%.
```

---

### Group of orthologs #757. Best score 325 bits Score difference with first non-orthologous sequence - AM1\_locus\_tags.txt:48 OB3b\_locus\_tags.txt:254

```
META1_4558          	100.00%		CQW49_RS21305       	100.00%
Bootstrap support for META1_4558 as seed ortholog is 83%.
Bootstrap support for CQW49_RS21305 as seed ortholog is 100%.
```

---

### Group of orthologs #758. Best score 324 bits Score difference with first non-orthologous sequence - AM1\_locus\_tags.txt:324 OB3b\_locus\_tags.txt:324

```
META1_0199          	100.00%		CQW49_RS02900       	100.00%
Bootstrap support for META1_0199 as seed ortholog is 100%.
Bootstrap support for CQW49_RS02900 as seed ortholog is 100%.
```

---

### Group of orthologs #759. Best score 324 bits Score difference with first non-orthologous sequence - AM1\_locus\_tags.txt:324 OB3b\_locus\_tags.txt:324

```
META1_1003          	100.00%		CQW49_RS13645       	100.00%
Bootstrap support for META1_1003 as seed ortholog is 100%.
Bootstrap support for CQW49_RS13645 as seed ortholog is 100%.
```

---

### Group of orthologs #760. Best score 324 bits Score difference with first non-orthologous sequence - AM1\_locus\_tags.txt:324 OB3b\_locus\_tags.txt:324

```
META1_3080          	100.00%		CQW49_RS12300       	100.00%
Bootstrap support for META1_3080 as seed ortholog is 100%.
Bootstrap support for CQW49_RS12300 as seed ortholog is 100%.
```

---

### Group of orthologs #761. Best score 323 bits Score difference with first non-orthologous sequence - AM1\_locus\_tags.txt:231 OB3b\_locus\_tags.txt:273

```
META1_2047          	100.00%		CQW49_RS02870       	100.00%
Bootstrap support for META1_2047 as seed ortholog is 100%.
Bootstrap support for CQW49_RS02870 as seed ortholog is 100%.
```

---

### Group of orthologs #762. Best score 323 bits Score difference with first non-orthologous sequence - AM1\_locus\_tags.txt:323 OB3b\_locus\_tags.txt:323

```
META1_1385          	100.00%		CQW49_RS12820       	100.00%
Bootstrap support for META1_1385 as seed ortholog is 100%.
Bootstrap support for CQW49_RS12820 as seed ortholog is 100%.
```

---

### Group of orthologs #763. Best score 323 bits Score difference with first non-orthologous sequence - AM1\_locus\_tags.txt:323 OB3b\_locus\_tags.txt:253

```
META1_3373          	100.00%		CQW49_RS19555       	100.00%
Bootstrap support for META1_3373 as seed ortholog is 100%.
Bootstrap support for CQW49_RS19555 as seed ortholog is 100%.
```

---

### Group of orthologs #764. Best score 323 bits Score difference with first non-orthologous sequence - AM1\_locus\_tags.txt:323 OB3b\_locus\_tags.txt:249

```
META1_4584          	100.00%		CQW49_RS20580       	100.00%
Bootstrap support for META1_4584 as seed ortholog is 100%.
Bootstrap support for CQW49_RS20580 as seed ortholog is 100%.
```

---

### Group of orthologs #765. Best score 322 bits Score difference with first non-orthologous sequence - AM1\_locus\_tags.txt:322 OB3b\_locus\_tags.txt:69

```
META1_4959          	100.00%		CQW49_RS00150       	100.00%
Bootstrap support for META1_4959 as seed ortholog is 100%.
Bootstrap support for CQW49_RS00150 as seed ortholog is 94%.
```

---

### Group of orthologs #766. Best score 321 bits Score difference with first non-orthologous sequence - AM1\_locus\_tags.txt:10 OB3b\_locus\_tags.txt:208

```
META1_0557          	100.00%		CQW49_RS06305       	100.00%
META1_4198          	14.21%		
Bootstrap support for META1_0557 as seed ortholog is 57%.
Alternative seed ortholog is META1_2385 (10 bits away from this cluster)
Bootstrap support for CQW49_RS06305 as seed ortholog is 100%.
```

---

### Group of orthologs #767. Best score 321 bits Score difference with first non-orthologous sequence - AM1\_locus\_tags.txt:321 OB3b\_locus\_tags.txt:321

```
META1_3487          	100.00%		CQW49_RS00950       	100.00%
Bootstrap support for META1_3487 as seed ortholog is 100%.
Bootstrap support for CQW49_RS00950 as seed ortholog is 100%.
```

---

### Group of orthologs #768. Best score 321 bits Score difference with first non-orthologous sequence - AM1\_locus\_tags.txt:321 OB3b\_locus\_tags.txt:321

```
META1_4674          	100.00%		CQW49_RS01060       	100.00%
Bootstrap support for META1_4674 as seed ortholog is 100%.
Bootstrap support for CQW49_RS01060 as seed ortholog is 100%.
```

---

### Group of orthologs #769. Best score 320 bits Score difference with first non-orthologous sequence - AM1\_locus\_tags.txt:320 OB3b\_locus\_tags.txt:320

```
META1_2732          	100.00%		CQW49_RS19640       	100.00%
Bootstrap support for META1_2732 as seed ortholog is 100%.
Bootstrap support for CQW49_RS19640 as seed ortholog is 100%.
```

---

### Group of orthologs #770. Best score 319 bits Score difference with first non-orthologous sequence - AM1\_locus\_tags.txt:226 OB3b\_locus\_tags.txt:223

```
META2_0173          	100.00%		CQW49_RS19935       	100.00%
META1_2654          	47.23%		
p1METAp0021         	6.62%		
Bootstrap support for META2_0173 as seed ortholog is 100%.
Bootstrap support for CQW49_RS19935 as seed ortholog is 100%.
```

---

### Group of orthologs #771. Best score 319 bits Score difference with first non-orthologous sequence - AM1\_locus\_tags.txt:319 OB3b\_locus\_tags.txt:319

```
META1_2198          	100.00%		CQW49_RS05385       	100.00%
Bootstrap support for META1_2198 as seed ortholog is 100%.
Bootstrap support for CQW49_RS05385 as seed ortholog is 100%.
```

---

### Group of orthologs #772. Best score 319 bits Score difference with first non-orthologous sequence - AM1\_locus\_tags.txt:319 OB3b\_locus\_tags.txt:319

```
META1_2208          	100.00%		CQW49_RS05880       	100.00%
Bootstrap support for META1_2208 as seed ortholog is 100%.
Bootstrap support for CQW49_RS05880 as seed ortholog is 100%.
```

---

### Group of orthologs #773. Best score 319 bits Score difference with first non-orthologous sequence - AM1\_locus\_tags.txt:319 OB3b\_locus\_tags.txt:319

```
META1_2837          	100.00%		CQW49_RS10330       	100.00%
Bootstrap support for META1_2837 as seed ortholog is 100%.
Bootstrap support for CQW49_RS10330 as seed ortholog is 100%.
```

---

### Group of orthologs #774. Best score 319 bits Score difference with first non-orthologous sequence - AM1\_locus\_tags.txt:59 OB3b\_locus\_tags.txt:319

```
META1_4948          	100.00%		CQW49_RS13010       	100.00%
Bootstrap support for META1_4948 as seed ortholog is 86%.
Bootstrap support for CQW49_RS13010 as seed ortholog is 100%.
```

---

### Group of orthologs #775. Best score 318 bits Score difference with first non-orthologous sequence - AM1\_locus\_tags.txt:318 OB3b\_locus\_tags.txt:318

```
META1_2511          	100.00%		CQW49_RS00060       	100.00%
Bootstrap support for META1_2511 as seed ortholog is 100%.
Bootstrap support for CQW49_RS00060 as seed ortholog is 100%.
```

---

### Group of orthologs #776. Best score 315 bits Score difference with first non-orthologous sequence - AM1\_locus\_tags.txt:182 OB3b\_locus\_tags.txt:226

```
META1_4684          	100.00%		CQW49_RS06540       	100.00%
Bootstrap support for META1_4684 as seed ortholog is 100%.
Bootstrap support for CQW49_RS06540 as seed ortholog is 100%.
```

---

### Group of orthologs #777. Best score 315 bits Score difference with first non-orthologous sequence - AM1\_locus\_tags.txt:213 OB3b\_locus\_tags.txt:225

```
META1_4313          	100.00%		CQW49_RS16175       	100.00%
Bootstrap support for META1_4313 as seed ortholog is 100%.
Bootstrap support for CQW49_RS16175 as seed ortholog is 100%.
```

---

### Group of orthologs #778. Best score 315 bits Score difference with first non-orthologous sequence - AM1\_locus\_tags.txt:315 OB3b\_locus\_tags.txt:315

```
META1_3661          	100.00%		CQW49_RS20175       	100.00%
Bootstrap support for META1_3661 as seed ortholog is 100%.
Bootstrap support for CQW49_RS20175 as seed ortholog is 100%.
```

---

### Group of orthologs #779. Best score 314 bits Score difference with first non-orthologous sequence - AM1\_locus\_tags.txt:314 OB3b\_locus\_tags.txt:314

```
META1_2175          	100.00%		CQW49_RS10435       	100.00%
Bootstrap support for META1_2175 as seed ortholog is 100%.
Bootstrap support for CQW49_RS10435 as seed ortholog is 100%.
```

---

### Group of orthologs #780. Best score 314 bits Score difference with first non-orthologous sequence - AM1\_locus\_tags.txt:234 OB3b\_locus\_tags.txt:314

```
META1_5244          	100.00%		CQW49_RS04360       	100.00%
Bootstrap support for META1_5244 as seed ortholog is 100%.
Bootstrap support for CQW49_RS04360 as seed ortholog is 100%.
```

---

### Group of orthologs #781. Best score 314 bits Score difference with first non-orthologous sequence - AM1\_locus\_tags.txt:172 OB3b\_locus\_tags.txt:243

```
META1_4277          	100.00%		CQW49_RS20515       	100.00%
Bootstrap support for META1_4277 as seed ortholog is 99%.
Bootstrap support for CQW49_RS20515 as seed ortholog is 100%.
```

---

### Group of orthologs #782. Best score 313 bits Score difference with first non-orthologous sequence - AM1\_locus\_tags.txt:135 OB3b\_locus\_tags.txt:313

```
META1_1820          	100.00%		CQW49_RS11845       	100.00%
Bootstrap support for META1_1820 as seed ortholog is 99%.
Bootstrap support for CQW49_RS11845 as seed ortholog is 100%.
```

---

### Group of orthologs #783. Best score 313 bits Score difference with first non-orthologous sequence - AM1\_locus\_tags.txt:313 OB3b\_locus\_tags.txt:84

```
META1_4360          	100.00%		CQW49_RS01420       	100.00%
Bootstrap support for META1_4360 as seed ortholog is 100%.
Bootstrap support for CQW49_RS01420 as seed ortholog is 98%.
```

---

### Group of orthologs #784. Best score 313 bits Score difference with first non-orthologous sequence - AM1\_locus\_tags.txt:66 OB3b\_locus\_tags.txt:313

```
META1_3484          	100.00%		CQW49_RS16650       	100.00%
Bootstrap support for META1_3484 as seed ortholog is 96%.
Bootstrap support for CQW49_RS16650 as seed ortholog is 100%.
```

---

### Group of orthologs #785. Best score 312 bits Score difference with first non-orthologous sequence - AM1\_locus\_tags.txt:312 OB3b\_locus\_tags.txt:312

```
META1_3629          	100.00%		CQW49_RS02205       	100.00%
Bootstrap support for META1_3629 as seed ortholog is 100%.
Bootstrap support for CQW49_RS02205 as seed ortholog is 100%.
```

---

### Group of orthologs #786. Best score 312 bits Score difference with first non-orthologous sequence - AM1\_locus\_tags.txt:312 OB3b\_locus\_tags.txt:312

```
META1_0666          	100.00%		CQW49_RS17635       	100.00%
Bootstrap support for META1_0666 as seed ortholog is 100%.
Bootstrap support for CQW49_RS17635 as seed ortholog is 100%.
```

---

### Group of orthologs #787. Best score 312 bits Score difference with first non-orthologous sequence - AM1\_locus\_tags.txt:52 OB3b\_locus\_tags.txt:312

```
META1_0452          	100.00%		CQW49_RS19820       	100.00%
Bootstrap support for META1_0452 as seed ortholog is 99%.
Bootstrap support for CQW49_RS19820 as seed ortholog is 100%.
```

---

### Group of orthologs #788. Best score 312 bits Score difference with first non-orthologous sequence - AM1\_locus\_tags.txt:312 OB3b\_locus\_tags.txt:312

```
META1_1930          	100.00%		CQW49_RS20600       	100.00%
Bootstrap support for META1_1930 as seed ortholog is 100%.
Bootstrap support for CQW49_RS20600 as seed ortholog is 100%.
```

---

### Group of orthologs #789. Best score 312 bits Score difference with first non-orthologous sequence - AM1\_locus\_tags.txt:312 OB3b\_locus\_tags.txt:312

```
META1_3156          	100.00%		CQW49_RS17850       	100.00%
Bootstrap support for META1_3156 as seed ortholog is 100%.
Bootstrap support for CQW49_RS17850 as seed ortholog is 100%.
```

---

### Group of orthologs #790. Best score 311 bits Score difference with first non-orthologous sequence - AM1\_locus\_tags.txt:151 OB3b\_locus\_tags.txt:171

```
META1_0619          	100.00%		CQW49_RS01850       	100.00%
Bootstrap support for META1_0619 as seed ortholog is 99%.
Bootstrap support for CQW49_RS01850 as seed ortholog is 99%.
```

---

### Group of orthologs #791. Best score 311 bits Score difference with first non-orthologous sequence - AM1\_locus\_tags.txt:145 OB3b\_locus\_tags.txt:160

```
META1_5219          	100.00%		CQW49_RS00720       	100.00%
Bootstrap support for META1_5219 as seed ortholog is 100%.
Bootstrap support for CQW49_RS00720 as seed ortholog is 100%.
```

---

### Group of orthologs #792. Best score 309 bits Score difference with first non-orthologous sequence - AM1\_locus\_tags.txt:309 OB3b\_locus\_tags.txt:309

```
META1_1477          	100.00%		CQW49_RS12785       	100.00%
Bootstrap support for META1_1477 as seed ortholog is 100%.
Bootstrap support for CQW49_RS12785 as seed ortholog is 100%.
```

---

### Group of orthologs #793. Best score 309 bits Score difference with first non-orthologous sequence - AM1\_locus\_tags.txt:89 OB3b\_locus\_tags.txt:309

```
META1_0463          	100.00%		CQW49_RS18800       	100.00%
Bootstrap support for META1_0463 as seed ortholog is 99%.
Bootstrap support for CQW49_RS18800 as seed ortholog is 100%.
```

---

### Group of orthologs #794. Best score 309 bits Score difference with first non-orthologous sequence - AM1\_locus\_tags.txt:309 OB3b\_locus\_tags.txt:309

```
META1_4576          	100.00%		CQW49_RS01610       	100.00%
Bootstrap support for META1_4576 as seed ortholog is 100%.
Bootstrap support for CQW49_RS01610 as seed ortholog is 100%.
```

---

### Group of orthologs #795. Best score 309 bits Score difference with first non-orthologous sequence - AM1\_locus\_tags.txt:309 OB3b\_locus\_tags.txt:309

```
META1_2273          	100.00%		CQW49_RS13475       	100.00%
Bootstrap support for META1_2273 as seed ortholog is 100%.
Bootstrap support for CQW49_RS13475 as seed ortholog is 100%.
```

---

### Group of orthologs #796. Best score 309 bits Score difference with first non-orthologous sequence - AM1\_locus\_tags.txt:309 OB3b\_locus\_tags.txt:309

```
META1_2419          	100.00%		CQW49_RS20885       	100.00%
Bootstrap support for META1_2419 as seed ortholog is 100%.
Bootstrap support for CQW49_RS20885 as seed ortholog is 100%.
```

---

### Group of orthologs #797. Best score 309 bits Score difference with first non-orthologous sequence - AM1\_locus\_tags.txt:90 OB3b\_locus\_tags.txt:257

```
META1_3221          	100.00%		CQW49_RS21380       	100.00%
Bootstrap support for META1_3221 as seed ortholog is 99%.
Bootstrap support for CQW49_RS21380 as seed ortholog is 100%.
```

---

### Group of orthologs #798. Best score 308 bits Score difference with first non-orthologous sequence - AM1\_locus\_tags.txt:308 OB3b\_locus\_tags.txt:118

```
META1_3132          	100.00%		CQW49_RS17895       	100.00%
Bootstrap support for META1_3132 as seed ortholog is 100%.
Bootstrap support for CQW49_RS17895 as seed ortholog is 99%.
```

---

### Group of orthologs #799. Best score 307 bits Score difference with first non-orthologous sequence - AM1\_locus\_tags.txt:224 OB3b\_locus\_tags.txt:307

```
META1_0078          	100.00%		CQW49_RS04735       	100.00%
META1_1241          	100.00%		
Bootstrap support for META1_0078 as seed ortholog is 100%.
Bootstrap support for META1_1241 as seed ortholog is 100%.
Bootstrap support for CQW49_RS04735 as seed ortholog is 100%.
```

---

### Group of orthologs #800. Best score 307 bits Score difference with first non-orthologous sequence - AM1\_locus\_tags.txt:307 OB3b\_locus\_tags.txt:307

```
META1_0814          	100.00%		CQW49_RS12295       	100.00%
Bootstrap support for META1_0814 as seed ortholog is 100%.
Bootstrap support for CQW49_RS12295 as seed ortholog is 100%.
```

---

### Group of orthologs #801. Best score 307 bits Score difference with first non-orthologous sequence - AM1\_locus\_tags.txt:175 OB3b\_locus\_tags.txt:179

```
META1_3067          	100.00%		CQW49_RS01970       	100.00%
Bootstrap support for META1_3067 as seed ortholog is 100%.
Bootstrap support for CQW49_RS01970 as seed ortholog is 100%.
```

---

### Group of orthologs #802. Best score 307 bits Score difference with first non-orthologous sequence - AM1\_locus\_tags.txt:115 OB3b\_locus\_tags.txt:307

```
META1_5008          	100.00%		CQW49_RS00955       	100.00%
Bootstrap support for META1_5008 as seed ortholog is 99%.
Bootstrap support for CQW49_RS00955 as seed ortholog is 100%.
```

---

### Group of orthologs #803. Best score 307 bits Score difference with first non-orthologous sequence - AM1\_locus\_tags.txt:307 OB3b\_locus\_tags.txt:307

```
META1_4311          	100.00%		CQW49_RS18490       	100.00%
Bootstrap support for META1_4311 as seed ortholog is 100%.
Bootstrap support for CQW49_RS18490 as seed ortholog is 100%.
```

---

### Group of orthologs #804. Best score 306 bits Score difference with first non-orthologous sequence - AM1\_locus\_tags.txt:306 OB3b\_locus\_tags.txt:306

```
META1_1497          	100.00%		CQW49_RS04675       	100.00%
Bootstrap support for META1_1497 as seed ortholog is 100%.
Bootstrap support for CQW49_RS04675 as seed ortholog is 100%.
```

---

### Group of orthologs #805. Best score 306 bits Score difference with first non-orthologous sequence - AM1\_locus\_tags.txt:306 OB3b\_locus\_tags.txt:306

```
META1_3019          	100.00%		CQW49_RS01675       	100.00%
Bootstrap support for META1_3019 as seed ortholog is 100%.
Bootstrap support for CQW49_RS01675 as seed ortholog is 100%.
```

---

### Group of orthologs #806. Best score 305 bits Score difference with first non-orthologous sequence - AM1\_locus\_tags.txt:231 OB3b\_locus\_tags.txt:305

```
META2_1007          	100.00%		CQW49_RS10765       	100.00%
META1_1207          	90.31%		
META1_2584          	71.56%		
META1_4209          	37.81%		
Bootstrap support for META2_1007 as seed ortholog is 100%.
Bootstrap support for CQW49_RS10765 as seed ortholog is 100%.
```

---

### Group of orthologs #807. Best score 305 bits Score difference with first non-orthologous sequence - AM1\_locus\_tags.txt:62 OB3b\_locus\_tags.txt:305

```
META1_3430          	100.00%		CQW49_RS00070       	100.00%
Bootstrap support for META1_3430 as seed ortholog is 98%.
Bootstrap support for CQW49_RS00070 as seed ortholog is 100%.
```

---

### Group of orthologs #808. Best score 305 bits Score difference with first non-orthologous sequence - AM1\_locus\_tags.txt:162 OB3b\_locus\_tags.txt:305

```
META1_4821          	100.00%		CQW49_RS22235       	100.00%
Bootstrap support for META1_4821 as seed ortholog is 100%.
Bootstrap support for CQW49_RS22235 as seed ortholog is 100%.
```

---

### Group of orthologs #809. Best score 304 bits Score difference with first non-orthologous sequence - AM1\_locus\_tags.txt:230 OB3b\_locus\_tags.txt:304

```
META1_1815          	100.00%		CQW49_RS11775       	100.00%
Bootstrap support for META1_1815 as seed ortholog is 100%.
Bootstrap support for CQW49_RS11775 as seed ortholog is 100%.
```

---

### Group of orthologs #810. Best score 304 bits Score difference with first non-orthologous sequence - AM1\_locus\_tags.txt:304 OB3b\_locus\_tags.txt:304

```
META1_4420          	100.00%		CQW49_RS01300       	100.00%
Bootstrap support for META1_4420 as seed ortholog is 100%.
Bootstrap support for CQW49_RS01300 as seed ortholog is 100%.
```

---

### Group of orthologs #811. Best score 304 bits Score difference with first non-orthologous sequence - AM1\_locus\_tags.txt:191 OB3b\_locus\_tags.txt:235

```
META1_3234          	100.00%		CQW49_RS09710       	100.00%
Bootstrap support for META1_3234 as seed ortholog is 100%.
Bootstrap support for CQW49_RS09710 as seed ortholog is 100%.
```

---

### Group of orthologs #812. Best score 304 bits Score difference with first non-orthologous sequence - AM1\_locus\_tags.txt:304 OB3b\_locus\_tags.txt:304

```
META1_4722          	100.00%		CQW49_RS17255       	100.00%
Bootstrap support for META1_4722 as seed ortholog is 100%.
Bootstrap support for CQW49_RS17255 as seed ortholog is 100%.
```

---

### Group of orthologs #813. Best score 303 bits Score difference with first non-orthologous sequence - AM1\_locus\_tags.txt:303 OB3b\_locus\_tags.txt:303

```
META1_5166          	100.00%		CQW49_RS15975       	100.00%
Bootstrap support for META1_5166 as seed ortholog is 100%.
Bootstrap support for CQW49_RS15975 as seed ortholog is 100%.
```

---

### Group of orthologs #814. Best score 302 bits Score difference with first non-orthologous sequence - AM1\_locus\_tags.txt:54 OB3b\_locus\_tags.txt:248

```
META1_4072          	100.00%		CQW49_RS19920       	100.00%
META1_4185          	20.38%		
META1_0278          	5.64%		
Bootstrap support for META1_4072 as seed ortholog is 3%.
Alternative seed ortholog is META1_2459 (54 bits away from this cluster)
Bootstrap support for CQW49_RS19920 as seed ortholog is 100%.
```

---

### Group of orthologs #815. Best score 302 bits Score difference with first non-orthologous sequence - AM1\_locus\_tags.txt:101 OB3b\_locus\_tags.txt:58

```
META1_0981          	100.00%		CQW49_RS04325       	100.00%
Bootstrap support for META1_0981 as seed ortholog is 95%.
Bootstrap support for CQW49_RS04325 as seed ortholog is 81%.
```

---

### Group of orthologs #816. Best score 302 bits Score difference with first non-orthologous sequence - AM1\_locus\_tags.txt:302 OB3b\_locus\_tags.txt:178

```
META1_1938          	100.00%		CQW49_RS00205       	100.00%
Bootstrap support for META1_1938 as seed ortholog is 100%.
Bootstrap support for CQW49_RS00205 as seed ortholog is 100%.
```

---

### Group of orthologs #817. Best score 302 bits Score difference with first non-orthologous sequence - AM1\_locus\_tags.txt:302 OB3b\_locus\_tags.txt:302

```
META1_1307          	100.00%		CQW49_RS04015       	100.00%
Bootstrap support for META1_1307 as seed ortholog is 100%.
Bootstrap support for CQW49_RS04015 as seed ortholog is 100%.
```

---

### Group of orthologs #818. Best score 302 bits Score difference with first non-orthologous sequence - AM1\_locus\_tags.txt:302 OB3b\_locus\_tags.txt:302

```
META1_1712          	100.00%		CQW49_RS17450       	100.00%
Bootstrap support for META1_1712 as seed ortholog is 100%.
Bootstrap support for CQW49_RS17450 as seed ortholog is 100%.
```

---

### Group of orthologs #819. Best score 301 bits Score difference with first non-orthologous sequence - AM1\_locus\_tags.txt:165 OB3b\_locus\_tags.txt:92

```
META1_2581          	100.00%		CQW49_RS22350       	100.00%
META2_1015          	60.63%		
META1_1199          	56.69%		
Bootstrap support for META1_2581 as seed ortholog is 100%.
Bootstrap support for CQW49_RS22350 as seed ortholog is 99%.
```

---

### Group of orthologs #820. Best score 301 bits Score difference with first non-orthologous sequence - AM1\_locus\_tags.txt:301 OB3b\_locus\_tags.txt:47

```
META1_2489          	100.00%		CQW49_RS22280       	100.00%
Bootstrap support for META1_2489 as seed ortholog is 100%.
Bootstrap support for CQW49_RS22280 as seed ortholog is 87%.
```

---

### Group of orthologs #821. Best score 301 bits Score difference with first non-orthologous sequence - AM1\_locus\_tags.txt:177 OB3b\_locus\_tags.txt:301

```
META2_0494          	100.00%		CQW49_RS22420       	100.00%
Bootstrap support for META2_0494 as seed ortholog is 99%.
Bootstrap support for CQW49_RS22420 as seed ortholog is 100%.
```

---

### Group of orthologs #822. Best score 300 bits Score difference with first non-orthologous sequence - AM1\_locus\_tags.txt:300 OB3b\_locus\_tags.txt:300

```
META1_1893          	100.00%		CQW49_RS15805       	100.00%
Bootstrap support for META1_1893 as seed ortholog is 100%.
Bootstrap support for CQW49_RS15805 as seed ortholog is 100%.
```

---

### Group of orthologs #823. Best score 300 bits Score difference with first non-orthologous sequence - AM1\_locus\_tags.txt:300 OB3b\_locus\_tags.txt:300

```
META1_5125          	100.00%		CQW49_RS00480       	100.00%
Bootstrap support for META1_5125 as seed ortholog is 100%.
Bootstrap support for CQW49_RS00480 as seed ortholog is 100%.
```

---

### Group of orthologs #824. Best score 300 bits Score difference with first non-orthologous sequence - AM1\_locus\_tags.txt:136 OB3b\_locus\_tags.txt:119

```
META1_0835          	100.00%		CQW49_RS20925       	100.00%
Bootstrap support for META1_0835 as seed ortholog is 100%.
Bootstrap support for CQW49_RS20925 as seed ortholog is 99%.
```

---

### Group of orthologs #825. Best score 300 bits Score difference with first non-orthologous sequence - AM1\_locus\_tags.txt:116 OB3b\_locus\_tags.txt:159

```
META1_3543          	100.00%		CQW49_RS21990       	100.00%
Bootstrap support for META1_3543 as seed ortholog is 99%.
Bootstrap support for CQW49_RS21990 as seed ortholog is 100%.
```

---

### Group of orthologs #826. Best score 299 bits Score difference with first non-orthologous sequence - AM1\_locus\_tags.txt:80 OB3b\_locus\_tags.txt:299

```
META1_0414          	100.00%		CQW49_RS03315       	100.00%
Bootstrap support for META1_0414 as seed ortholog is 96%.
Bootstrap support for CQW49_RS03315 as seed ortholog is 100%.
```

---

### Group of orthologs #827. Best score 299 bits Score difference with first non-orthologous sequence - AM1\_locus\_tags.txt:299 OB3b\_locus\_tags.txt:299

```
META1_4364          	100.00%		CQW49_RS01405       	100.00%
Bootstrap support for META1_4364 as seed ortholog is 100%.
Bootstrap support for CQW49_RS01405 as seed ortholog is 100%.
```

---

### Group of orthologs #828. Best score 299 bits Score difference with first non-orthologous sequence - AM1\_locus\_tags.txt:299 OB3b\_locus\_tags.txt:299

```
META1_3091          	100.00%		CQW49_RS12215       	100.00%
Bootstrap support for META1_3091 as seed ortholog is 100%.
Bootstrap support for CQW49_RS12215 as seed ortholog is 100%.
```

---

### Group of orthologs #829. Best score 298 bits Score difference with first non-orthologous sequence - AM1\_locus\_tags.txt:298 OB3b\_locus\_tags.txt:298

```
META1_3173          	100.00%		CQW49_RS17665       	100.00%
Bootstrap support for META1_3173 as seed ortholog is 100%.
Bootstrap support for CQW49_RS17665 as seed ortholog is 100%.
```

---

### Group of orthologs #830. Best score 298 bits Score difference with first non-orthologous sequence - AM1\_locus\_tags.txt:298 OB3b\_locus\_tags.txt:298

```
META1_2473          	100.00%		CQW49_RS21295       	100.00%
Bootstrap support for META1_2473 as seed ortholog is 100%.
Bootstrap support for CQW49_RS21295 as seed ortholog is 100%.
```

---

### Group of orthologs #831. Best score 298 bits Score difference with first non-orthologous sequence - AM1\_locus\_tags.txt:298 OB3b\_locus\_tags.txt:298

```
META1_4436          	100.00%		CQW49_RS18505       	100.00%
Bootstrap support for META1_4436 as seed ortholog is 100%.
Bootstrap support for CQW49_RS18505 as seed ortholog is 100%.
```

---

### Group of orthologs #832. Best score 297 bits Score difference with first non-orthologous sequence - AM1\_locus\_tags.txt:176 OB3b\_locus\_tags.txt:297

```
META1_0411          	100.00%		CQW49_RS09005       	100.00%
Bootstrap support for META1_0411 as seed ortholog is 100%.
Bootstrap support for CQW49_RS09005 as seed ortholog is 100%.
```

---

### Group of orthologs #833. Best score 297 bits Score difference with first non-orthologous sequence - AM1\_locus\_tags.txt:69 OB3b\_locus\_tags.txt:84

```
META1_0924          	100.00%		CQW49_RS08825       	100.00%
Bootstrap support for META1_0924 as seed ortholog is 95%.
Bootstrap support for CQW49_RS08825 as seed ortholog is 96%.
```

---

### Group of orthologs #834. Best score 297 bits Score difference with first non-orthologous sequence - AM1\_locus\_tags.txt:297 OB3b\_locus\_tags.txt:297

```
META1_4140          	100.00%		CQW49_RS02885       	100.00%
Bootstrap support for META1_4140 as seed ortholog is 100%.
Bootstrap support for CQW49_RS02885 as seed ortholog is 100%.
```

---

### Group of orthologs #835. Best score 297 bits Score difference with first non-orthologous sequence - AM1\_locus\_tags.txt:98 OB3b\_locus\_tags.txt:116

```
META1_4380          	100.00%		CQW49_RS02000       	100.00%
Bootstrap support for META1_4380 as seed ortholog is 99%.
Bootstrap support for CQW49_RS02000 as seed ortholog is 100%.
```

---

### Group of orthologs #836. Best score 297 bits Score difference with first non-orthologous sequence - AM1\_locus\_tags.txt:297 OB3b\_locus\_tags.txt:297

```
META1_2271          	100.00%		CQW49_RS13465       	100.00%
Bootstrap support for META1_2271 as seed ortholog is 100%.
Bootstrap support for CQW49_RS13465 as seed ortholog is 100%.
```

---

### Group of orthologs #837. Best score 296 bits Score difference with first non-orthologous sequence - AM1\_locus\_tags.txt:296 OB3b\_locus\_tags.txt:296

```
META1_1778          	100.00%		CQW49_RS19335       	100.00%
Bootstrap support for META1_1778 as seed ortholog is 100%.
Bootstrap support for CQW49_RS19335 as seed ortholog is 100%.
```

---

### Group of orthologs #838. Best score 296 bits Score difference with first non-orthologous sequence - AM1\_locus\_tags.txt:296 OB3b\_locus\_tags.txt:296

```
META1_2287          	100.00%		CQW49_RS19840       	100.00%
Bootstrap support for META1_2287 as seed ortholog is 100%.
Bootstrap support for CQW49_RS19840 as seed ortholog is 100%.
```

---

### Group of orthologs #839. Best score 296 bits Score difference with first non-orthologous sequence - AM1\_locus\_tags.txt:296 OB3b\_locus\_tags.txt:296

```
META1_2268          	100.00%		CQW49_RS19940       	100.00%
Bootstrap support for META1_2268 as seed ortholog is 100%.
Bootstrap support for CQW49_RS19940 as seed ortholog is 100%.
```

---

### Group of orthologs #840. Best score 295 bits Score difference with first non-orthologous sequence - AM1\_locus\_tags.txt:295 OB3b\_locus\_tags.txt:295

```
META1_1749          	100.00%		CQW49_RS03360       	100.00%
Bootstrap support for META1_1749 as seed ortholog is 100%.
Bootstrap support for CQW49_RS03360 as seed ortholog is 100%.
```

---

### Group of orthologs #841. Best score 295 bits Score difference with first non-orthologous sequence - AM1\_locus\_tags.txt:295 OB3b\_locus\_tags.txt:295

```
META1_1760          	100.00%		CQW49_RS10305       	100.00%
Bootstrap support for META1_1760 as seed ortholog is 100%.
Bootstrap support for CQW49_RS10305 as seed ortholog is 100%.
```

---

### Group of orthologs #842. Best score 295 bits Score difference with first non-orthologous sequence - AM1\_locus\_tags.txt:295 OB3b\_locus\_tags.txt:295

```
META1_2517          	100.00%		CQW49_RS07620       	100.00%
Bootstrap support for META1_2517 as seed ortholog is 100%.
Bootstrap support for CQW49_RS07620 as seed ortholog is 100%.
```

---

### Group of orthologs #843. Best score 295 bits Score difference with first non-orthologous sequence - AM1\_locus\_tags.txt:295 OB3b\_locus\_tags.txt:295

```
META1_1004          	100.00%		CQW49_RS17815       	100.00%
Bootstrap support for META1_1004 as seed ortholog is 100%.
Bootstrap support for CQW49_RS17815 as seed ortholog is 100%.
```

---

### Group of orthologs #844. Best score 295 bits Score difference with first non-orthologous sequence - AM1\_locus\_tags.txt:295 OB3b\_locus\_tags.txt:295

```
META1_3418          	100.00%		CQW49_RS09995       	100.00%
Bootstrap support for META1_3418 as seed ortholog is 100%.
Bootstrap support for CQW49_RS09995 as seed ortholog is 100%.
```

---

### Group of orthologs #845. Best score 295 bits Score difference with first non-orthologous sequence - AM1\_locus\_tags.txt:295 OB3b\_locus\_tags.txt:295

```
META1_1500          	100.00%		CQW49_RS20115       	100.00%
Bootstrap support for META1_1500 as seed ortholog is 100%.
Bootstrap support for CQW49_RS20115 as seed ortholog is 100%.
```

---

### Group of orthologs #846. Best score 295 bits Score difference with first non-orthologous sequence - AM1\_locus\_tags.txt:295 OB3b\_locus\_tags.txt:295

```
META1_5006          	100.00%		CQW49_RS20410       	100.00%
Bootstrap support for META1_5006 as seed ortholog is 100%.
Bootstrap support for CQW49_RS20410 as seed ortholog is 100%.
```

---

### Group of orthologs #847. Best score 294 bits Score difference with first non-orthologous sequence - AM1\_locus\_tags.txt:294 OB3b\_locus\_tags.txt:294

```
META1_0186          	100.00%		CQW49_RS13325       	100.00%
Bootstrap support for META1_0186 as seed ortholog is 100%.
Bootstrap support for CQW49_RS13325 as seed ortholog is 100%.
```

---

### Group of orthologs #848. Best score 294 bits Score difference with first non-orthologous sequence - AM1\_locus\_tags.txt:294 OB3b\_locus\_tags.txt:294

```
META1_3428          	100.00%		CQW49_RS11210       	100.00%
Bootstrap support for META1_3428 as seed ortholog is 100%.
Bootstrap support for CQW49_RS11210 as seed ortholog is 100%.
```

---

### Group of orthologs #849. Best score 294 bits Score difference with first non-orthologous sequence - AM1\_locus\_tags.txt:236 OB3b\_locus\_tags.txt:294

```
META1_2388          	100.00%		CQW49_RS16590       	100.00%
Bootstrap support for META1_2388 as seed ortholog is 100%.
Bootstrap support for CQW49_RS16590 as seed ortholog is 100%.
```

---

### Group of orthologs #850. Best score 294 bits Score difference with first non-orthologous sequence - AM1\_locus\_tags.txt:294 OB3b\_locus\_tags.txt:294

```
META1_3918          	100.00%		CQW49_RS11810       	100.00%
Bootstrap support for META1_3918 as seed ortholog is 100%.
Bootstrap support for CQW49_RS11810 as seed ortholog is 100%.
```

---

### Group of orthologs #851. Best score 293 bits Score difference with first non-orthologous sequence - AM1\_locus\_tags.txt:293 OB3b\_locus\_tags.txt:293

```
META1_2462          	100.00%		CQW49_RS12025       	100.00%
Bootstrap support for META1_2462 as seed ortholog is 100%.
Bootstrap support for CQW49_RS12025 as seed ortholog is 100%.
```

---

### Group of orthologs #852. Best score 293 bits Score difference with first non-orthologous sequence - AM1\_locus\_tags.txt:293 OB3b\_locus\_tags.txt:293

```
META1_5047          	100.00%		CQW49_RS03080       	100.00%
Bootstrap support for META1_5047 as seed ortholog is 100%.
Bootstrap support for CQW49_RS03080 as seed ortholog is 100%.
```

---

### Group of orthologs #853. Best score 292 bits Score difference with first non-orthologous sequence - AM1\_locus\_tags.txt:292 OB3b\_locus\_tags.txt:292

```
META1_3068          	100.00%		CQW49_RS01975       	100.00%
Bootstrap support for META1_3068 as seed ortholog is 100%.
Bootstrap support for CQW49_RS01975 as seed ortholog is 100%.
```

---

### Group of orthologs #854. Best score 292 bits Score difference with first non-orthologous sequence - AM1\_locus\_tags.txt:292 OB3b\_locus\_tags.txt:292

```
META1_1761          	100.00%		CQW49_RS13885       	100.00%
Bootstrap support for META1_1761 as seed ortholog is 100%.
Bootstrap support for CQW49_RS13885 as seed ortholog is 100%.
```

---

### Group of orthologs #855. Best score 292 bits Score difference with first non-orthologous sequence - AM1\_locus\_tags.txt:292 OB3b\_locus\_tags.txt:196

```
META1_3388          	100.00%		CQW49_RS19280       	100.00%
Bootstrap support for META1_3388 as seed ortholog is 100%.
Bootstrap support for CQW49_RS19280 as seed ortholog is 100%.
```

---

### Group of orthologs #856. Best score 292 bits Score difference with first non-orthologous sequence - AM1\_locus\_tags.txt:292 OB3b\_locus\_tags.txt:292

```
META1_5168          	100.00%		CQW49_RS15985       	100.00%
Bootstrap support for META1_5168 as seed ortholog is 100%.
Bootstrap support for CQW49_RS15985 as seed ortholog is 100%.
```

---

### Group of orthologs #857. Best score 291 bits Score difference with first non-orthologous sequence - AM1\_locus\_tags.txt:133 OB3b\_locus\_tags.txt:291

```
META1_1770          	100.00%		CQW49_RS05600       	100.00%
Bootstrap support for META1_1770 as seed ortholog is 99%.
Bootstrap support for CQW49_RS05600 as seed ortholog is 100%.
```

---

### Group of orthologs #858. Best score 291 bits Score difference with first non-orthologous sequence - AM1\_locus\_tags.txt:291 OB3b\_locus\_tags.txt:291

```
META1_4577          	100.00%		CQW49_RS01605       	100.00%
Bootstrap support for META1_4577 as seed ortholog is 100%.
Bootstrap support for CQW49_RS01605 as seed ortholog is 100%.
```

---

### Group of orthologs #859. Best score 290 bits Score difference with first non-orthologous sequence - AM1\_locus\_tags.txt:52 OB3b\_locus\_tags.txt:290

```
META1_1652          	100.00%		CQW49_RS05745       	100.00%
Bootstrap support for META1_1652 as seed ortholog is 95%.
Bootstrap support for CQW49_RS05745 as seed ortholog is 100%.
```

---

### Group of orthologs #860. Best score 290 bits Score difference with first non-orthologous sequence - AM1\_locus\_tags.txt:115 OB3b\_locus\_tags.txt:290

```
META1_3115          	100.00%		CQW49_RS02655       	100.00%
Bootstrap support for META1_3115 as seed ortholog is 99%.
Bootstrap support for CQW49_RS02655 as seed ortholog is 100%.
```

---

### Group of orthologs #861. Best score 289 bits Score difference with first non-orthologous sequence - AM1\_locus\_tags.txt:289 OB3b\_locus\_tags.txt:289

```
META1_0847          	100.00%		CQW49_RS05315       	100.00%
Bootstrap support for META1_0847 as seed ortholog is 100%.
Bootstrap support for CQW49_RS05315 as seed ortholog is 100%.
```

---

### Group of orthologs #862. Best score 289 bits Score difference with first non-orthologous sequence - AM1\_locus\_tags.txt:289 OB3b\_locus\_tags.txt:225

```
META1_2850          	100.00%		CQW49_RS02725       	100.00%
Bootstrap support for META1_2850 as seed ortholog is 100%.
Bootstrap support for CQW49_RS02725 as seed ortholog is 100%.
```

---

### Group of orthologs #863. Best score 289 bits Score difference with first non-orthologous sequence - AM1\_locus\_tags.txt:289 OB3b\_locus\_tags.txt:289

```
META1_3095          	100.00%		CQW49_RS04555       	100.00%
Bootstrap support for META1_3095 as seed ortholog is 100%.
Bootstrap support for CQW49_RS04555 as seed ortholog is 100%.
```

---

### Group of orthologs #864. Best score 289 bits Score difference with first non-orthologous sequence - AM1\_locus\_tags.txt:57 OB3b\_locus\_tags.txt:289

```
META1_4690          	100.00%		CQW49_RS12930       	100.00%
Bootstrap support for META1_4690 as seed ortholog is 11%.
Alternative seed ortholog is META2_0893 (57 bits away from this cluster)
Bootstrap support for CQW49_RS12930 as seed ortholog is 100%.
```

---

### Group of orthologs #865. Best score 289 bits Score difference with first non-orthologous sequence - AM1\_locus\_tags.txt:289 OB3b\_locus\_tags.txt:223

```
META1_4914          	100.00%		CQW49_RS16075       	100.00%
Bootstrap support for META1_4914 as seed ortholog is 100%.
Bootstrap support for CQW49_RS16075 as seed ortholog is 100%.
```

---

### Group of orthologs #866. Best score 288 bits Score difference with first non-orthologous sequence - AM1\_locus\_tags.txt:288 OB3b\_locus\_tags.txt:288

```
META1_2917          	100.00%		CQW49_RS00230       	100.00%
Bootstrap support for META1_2917 as seed ortholog is 100%.
Bootstrap support for CQW49_RS00230 as seed ortholog is 100%.
```

---

### Group of orthologs #867. Best score 288 bits Score difference with first non-orthologous sequence - AM1\_locus\_tags.txt:288 OB3b\_locus\_tags.txt:288

```
META1_0601          	100.00%		CQW49_RS12165       	100.00%
Bootstrap support for META1_0601 as seed ortholog is 100%.
Bootstrap support for CQW49_RS12165 as seed ortholog is 100%.
```

---

### Group of orthologs #868. Best score 288 bits Score difference with first non-orthologous sequence - AM1\_locus\_tags.txt:54 OB3b\_locus\_tags.txt:199

```
META1_2755          	100.00%		CQW49_RS15560       	100.00%
Bootstrap support for META1_2755 as seed ortholog is 98%.
Bootstrap support for CQW49_RS15560 as seed ortholog is 100%.
```

---

### Group of orthologs #869. Best score 287 bits Score difference with first non-orthologous sequence - AM1\_locus\_tags.txt:287 OB3b\_locus\_tags.txt:287

```
META1_1390          	100.00%		CQW49_RS02685       	100.00%
Bootstrap support for META1_1390 as seed ortholog is 100%.
Bootstrap support for CQW49_RS02685 as seed ortholog is 100%.
```

---

### Group of orthologs #870. Best score 287 bits Score difference with first non-orthologous sequence - AM1\_locus\_tags.txt:287 OB3b\_locus\_tags.txt:287

```
META1_0790          	100.00%		CQW49_RS09935       	100.00%
Bootstrap support for META1_0790 as seed ortholog is 100%.
Bootstrap support for CQW49_RS09935 as seed ortholog is 100%.
```

---

### Group of orthologs #871. Best score 287 bits Score difference with first non-orthologous sequence - AM1\_locus\_tags.txt:99 OB3b\_locus\_tags.txt:36

```
META1_4143          	100.00%		CQW49_RS11060       	100.00%
Bootstrap support for META1_4143 as seed ortholog is 99%.
Bootstrap support for CQW49_RS11060 as seed ortholog is 90%.
```

---

### Group of orthologs #872. Best score 286 bits Score difference with first non-orthologous sequence - AM1\_locus\_tags.txt:36 OB3b\_locus\_tags.txt:160

```
META1_1130          	100.00%		CQW49_RS16595       	100.00%
                    	       		CQW49_RS18205       	30.79%
Bootstrap support for META1_1130 as seed ortholog is 82%.
Bootstrap support for CQW49_RS16595 as seed ortholog is 99%.
```

---

### Group of orthologs #873. Best score 286 bits Score difference with first non-orthologous sequence - AM1\_locus\_tags.txt:123 OB3b\_locus\_tags.txt:286

```
META1_0464          	100.00%		CQW49_RS18795       	100.00%
Bootstrap support for META1_0464 as seed ortholog is 99%.
Bootstrap support for CQW49_RS18795 as seed ortholog is 100%.
```

---

### Group of orthologs #874. Best score 286 bits Score difference with first non-orthologous sequence - AM1\_locus\_tags.txt:286 OB3b\_locus\_tags.txt:286

```
META1_1350          	100.00%		CQW49_RS20960       	100.00%
Bootstrap support for META1_1350 as seed ortholog is 100%.
Bootstrap support for CQW49_RS20960 as seed ortholog is 100%.
```

---

### Group of orthologs #875. Best score 285 bits Score difference with first non-orthologous sequence - AM1\_locus\_tags.txt:173 OB3b\_locus\_tags.txt:285

```
META1_1816          	100.00%		CQW49_RS11770       	100.00%
Bootstrap support for META1_1816 as seed ortholog is 100%.
Bootstrap support for CQW49_RS11770 as seed ortholog is 100%.
```

---

### Group of orthologs #876. Best score 285 bits Score difference with first non-orthologous sequence - AM1\_locus\_tags.txt:285 OB3b\_locus\_tags.txt:285

```
META1_3155          	100.00%		CQW49_RS11305       	100.00%
Bootstrap support for META1_3155 as seed ortholog is 100%.
Bootstrap support for CQW49_RS11305 as seed ortholog is 100%.
```

---

### Group of orthologs #877. Best score 285 bits Score difference with first non-orthologous sequence - AM1\_locus\_tags.txt:285 OB3b\_locus\_tags.txt:233

```
META1_4384          	100.00%		CQW49_RS11360       	100.00%
Bootstrap support for META1_4384 as seed ortholog is 100%.
Bootstrap support for CQW49_RS11360 as seed ortholog is 100%.
```

---

### Group of orthologs #878. Best score 284 bits Score difference with first non-orthologous sequence - AM1\_locus\_tags.txt:284 OB3b\_locus\_tags.txt:284

```
META1_1532          	100.00%		CQW49_RS00660       	100.00%
Bootstrap support for META1_1532 as seed ortholog is 100%.
Bootstrap support for CQW49_RS00660 as seed ortholog is 100%.
```

---

### Group of orthologs #879. Best score 284 bits Score difference with first non-orthologous sequence - AM1\_locus\_tags.txt:284 OB3b\_locus\_tags.txt:284

```
META1_2086          	100.00%		CQW49_RS04880       	100.00%
Bootstrap support for META1_2086 as seed ortholog is 100%.
Bootstrap support for CQW49_RS04880 as seed ortholog is 100%.
```

---

### Group of orthologs #880. Best score 284 bits Score difference with first non-orthologous sequence - AM1\_locus\_tags.txt:284 OB3b\_locus\_tags.txt:284

```
META1_2247          	100.00%		CQW49_RS04865       	100.00%
Bootstrap support for META1_2247 as seed ortholog is 100%.
Bootstrap support for CQW49_RS04865 as seed ortholog is 100%.
```

---

### Group of orthologs #881. Best score 284 bits Score difference with first non-orthologous sequence - AM1\_locus\_tags.txt:284 OB3b\_locus\_tags.txt:284

```
META1_5192          	100.00%		CQW49_RS03340       	100.00%
Bootstrap support for META1_5192 as seed ortholog is 100%.
Bootstrap support for CQW49_RS03340 as seed ortholog is 100%.
```

---

### Group of orthologs #882. Best score 283 bits Score difference with first non-orthologous sequence - AM1\_locus\_tags.txt:283 OB3b\_locus\_tags.txt:283

```
META1_0979          	100.00%		CQW49_RS21440       	100.00%
META1_3607          	69.56%		CQW49_RS09285       	9.57%
Bootstrap support for META1_0979 as seed ortholog is 100%.
Bootstrap support for CQW49_RS21440 as seed ortholog is 100%.
```

---

### Group of orthologs #883. Best score 283 bits Score difference with first non-orthologous sequence - AM1\_locus\_tags.txt:283 OB3b\_locus\_tags.txt:283

```
META1_0851          	100.00%		CQW49_RS05335       	100.00%
Bootstrap support for META1_0851 as seed ortholog is 100%.
Bootstrap support for CQW49_RS05335 as seed ortholog is 100%.
```

---

### Group of orthologs #884. Best score 282 bits Score difference with first non-orthologous sequence - AM1\_locus\_tags.txt:60 OB3b\_locus\_tags.txt:86

```
META1_2112          	100.00%		CQW49_RS18175       	100.00%
                    	       		CQW49_RS13695       	43.07%
                    	       		CQW49_RS18235       	42.64%
                    	       		CQW49_RS21785       	39.94%
                    	       		CQW49_RS07180       	36.25%
                    	       		CQW49_RS02430       	28.78%
                    	       		CQW49_RS02600       	24.52%
                    	       		CQW49_RS06265       	21.11%
                    	       		CQW49_RS05625       	10.80%
                    	       		CQW49_RS03265       	9.95%
                    	       		CQW49_RS13595       	8.67%
Bootstrap support for META1_2112 as seed ortholog is 82%.
Bootstrap support for CQW49_RS18175 as seed ortholog is 91%.
```

---

### Group of orthologs #885. Best score 282 bits Score difference with first non-orthologous sequence - AM1\_locus\_tags.txt:282 OB3b\_locus\_tags.txt:282

```
META1_0288          	100.00%		CQW49_RS03935       	100.00%
Bootstrap support for META1_0288 as seed ortholog is 100%.
Bootstrap support for CQW49_RS03935 as seed ortholog is 100%.
```

---

### Group of orthologs #886. Best score 282 bits Score difference with first non-orthologous sequence - AM1\_locus\_tags.txt:282 OB3b\_locus\_tags.txt:282

```
META1_5010          	100.00%		CQW49_RS06435       	100.00%
Bootstrap support for META1_5010 as seed ortholog is 100%.
Bootstrap support for CQW49_RS06435 as seed ortholog is 100%.
```

---

### Group of orthologs #887. Best score 282 bits Score difference with first non-orthologous sequence - AM1\_locus\_tags.txt:282 OB3b\_locus\_tags.txt:282

```
META1_3367          	100.00%		CQW49_RS17930       	100.00%
Bootstrap support for META1_3367 as seed ortholog is 100%.
Bootstrap support for CQW49_RS17930 as seed ortholog is 100%.
```

---

### Group of orthologs #888. Best score 281 bits Score difference with first non-orthologous sequence - AM1\_locus\_tags.txt:281 OB3b\_locus\_tags.txt:281

```
META1_2310          	100.00%		CQW49_RS12600       	100.00%
Bootstrap support for META1_2310 as seed ortholog is 100%.
Bootstrap support for CQW49_RS12600 as seed ortholog is 100%.
```

---

### Group of orthologs #889. Best score 281 bits Score difference with first non-orthologous sequence - AM1\_locus\_tags.txt:180 OB3b\_locus\_tags.txt:281

```
META1_1738          	100.00%		CQW49_RS15925       	100.00%
Bootstrap support for META1_1738 as seed ortholog is 100%.
Bootstrap support for CQW49_RS15925 as seed ortholog is 100%.
```

---

### Group of orthologs #890. Best score 281 bits Score difference with first non-orthologous sequence - AM1\_locus\_tags.txt:281 OB3b\_locus\_tags.txt:226

```
META1_4583          	100.00%		CQW49_RS20575       	100.00%
Bootstrap support for META1_4583 as seed ortholog is 100%.
Bootstrap support for CQW49_RS20575 as seed ortholog is 100%.
```

---

### Group of orthologs #891. Best score 280 bits Score difference with first non-orthologous sequence - AM1\_locus\_tags.txt:280 OB3b\_locus\_tags.txt:280

```
META1_3508          	100.00%		CQW49_RS20030       	100.00%
                    	       		CQW49_RS09585       	17.90%
Bootstrap support for META1_3508 as seed ortholog is 100%.
Bootstrap support for CQW49_RS20030 as seed ortholog is 100%.
```

---

### Group of orthologs #892. Best score 280 bits Score difference with first non-orthologous sequence - AM1\_locus\_tags.txt:216 OB3b\_locus\_tags.txt:200

```
META1_0421          	100.00%		CQW49_RS03045       	100.00%
Bootstrap support for META1_0421 as seed ortholog is 100%.
Bootstrap support for CQW49_RS03045 as seed ortholog is 100%.
```

---

### Group of orthologs #893. Best score 280 bits Score difference with first non-orthologous sequence - AM1\_locus\_tags.txt:280 OB3b\_locus\_tags.txt:280

```
META1_2180          	100.00%		CQW49_RS10460       	100.00%
Bootstrap support for META1_2180 as seed ortholog is 100%.
Bootstrap support for CQW49_RS10460 as seed ortholog is 100%.
```

---

### Group of orthologs #894. Best score 280 bits Score difference with first non-orthologous sequence - AM1\_locus\_tags.txt:280 OB3b\_locus\_tags.txt:280

```
META1_0768          	100.00%		CQW49_RS18445       	100.00%
Bootstrap support for META1_0768 as seed ortholog is 100%.
Bootstrap support for CQW49_RS18445 as seed ortholog is 100%.
```

---

### Group of orthologs #895. Best score 280 bits Score difference with first non-orthologous sequence - AM1\_locus\_tags.txt:280 OB3b\_locus\_tags.txt:280

```
META1_4604          	100.00%		CQW49_RS15540       	100.00%
Bootstrap support for META1_4604 as seed ortholog is 100%.
Bootstrap support for CQW49_RS15540 as seed ortholog is 100%.
```

---

### Group of orthologs #896. Best score 279 bits Score difference with first non-orthologous sequence - AM1\_locus\_tags.txt:279 OB3b\_locus\_tags.txt:82

```
META1_3010          	100.00%		CQW49_RS02335       	100.00%
                    	       		CQW49_RS24070       	55.50%
                    	       		CQW49_RS13815       	51.27%
Bootstrap support for META1_3010 as seed ortholog is 100%.
Bootstrap support for CQW49_RS02335 as seed ortholog is 98%.
```

---

### Group of orthologs #897. Best score 279 bits Score difference with first non-orthologous sequence - AM1\_locus\_tags.txt:279 OB3b\_locus\_tags.txt:279

```
META1_2154          	100.00%		CQW49_RS10515       	100.00%
Bootstrap support for META1_2154 as seed ortholog is 100%.
Bootstrap support for CQW49_RS10515 as seed ortholog is 100%.
```

---

### Group of orthologs #898. Best score 279 bits Score difference with first non-orthologous sequence - AM1\_locus\_tags.txt:173 OB3b\_locus\_tags.txt:279

```
META1_3493          	100.00%		CQW49_RS13190       	100.00%
Bootstrap support for META1_3493 as seed ortholog is 100%.
Bootstrap support for CQW49_RS13190 as seed ortholog is 100%.
```

---

### Group of orthologs #899. Best score 278 bits Score difference with first non-orthologous sequence - AM1\_locus\_tags.txt:278 OB3b\_locus\_tags.txt:278

```
META1_3512          	100.00%		CQW49_RS01285       	100.00%
Bootstrap support for META1_3512 as seed ortholog is 100%.
Bootstrap support for CQW49_RS01285 as seed ortholog is 100%.
```

---

### Group of orthologs #900. Best score 278 bits Score difference with first non-orthologous sequence - AM1\_locus\_tags.txt:278 OB3b\_locus\_tags.txt:278

```
META1_2881          	100.00%		CQW49_RS15955       	100.00%
Bootstrap support for META1_2881 as seed ortholog is 100%.
Bootstrap support for CQW49_RS15955 as seed ortholog is 100%.
```

---

### Group of orthologs #901. Best score 278 bits Score difference with first non-orthologous sequence - AM1\_locus\_tags.txt:278 OB3b\_locus\_tags.txt:278

```
META1_3602          	100.00%		CQW49_RS22150       	100.00%
Bootstrap support for META1_3602 as seed ortholog is 100%.
Bootstrap support for CQW49_RS22150 as seed ortholog is 100%.
```

---

### Group of orthologs #902. Best score 277 bits Score difference with first non-orthologous sequence - AM1\_locus\_tags.txt:277 OB3b\_locus\_tags.txt:277

```
META1_0739          	100.00%		CQW49_RS04280       	100.00%
Bootstrap support for META1_0739 as seed ortholog is 100%.
Bootstrap support for CQW49_RS04280 as seed ortholog is 100%.
```

---

### Group of orthologs #903. Best score 277 bits Score difference with first non-orthologous sequence - AM1\_locus\_tags.txt:29 OB3b\_locus\_tags.txt:277

```
META1_2123          	100.00%		CQW49_RS06545       	100.00%
Bootstrap support for META1_2123 as seed ortholog is 85%.
Bootstrap support for CQW49_RS06545 as seed ortholog is 100%.
```

---

### Group of orthologs #904. Best score 277 bits Score difference with first non-orthologous sequence - AM1\_locus\_tags.txt:277 OB3b\_locus\_tags.txt:200

```
META1_3082          	100.00%		CQW49_RS03705       	100.00%
Bootstrap support for META1_3082 as seed ortholog is 100%.
Bootstrap support for CQW49_RS03705 as seed ortholog is 100%.
```

---

### Group of orthologs #905. Best score 277 bits Score difference with first non-orthologous sequence - AM1\_locus\_tags.txt:27 OB3b\_locus\_tags.txt:102

```
META1_1819          	100.00%		CQW49_RS11840       	100.00%
Bootstrap support for META1_1819 as seed ortholog is 85%.
Bootstrap support for CQW49_RS11840 as seed ortholog is 99%.
```

---

### Group of orthologs #906. Best score 276 bits Score difference with first non-orthologous sequence - AM1\_locus\_tags.txt:276 OB3b\_locus\_tags.txt:276

```
META1_3089          	100.00%		CQW49_RS12205       	100.00%
Bootstrap support for META1_3089 as seed ortholog is 100%.
Bootstrap support for CQW49_RS12205 as seed ortholog is 100%.
```

---

### Group of orthologs #907. Best score 275 bits Score difference with first non-orthologous sequence - AM1\_locus\_tags.txt:275 OB3b\_locus\_tags.txt:275

```
META1_4189          	100.00%		CQW49_RS06230       	100.00%
META1_1079          	11.56%		
META1_1376          	9.28%		
Bootstrap support for META1_4189 as seed ortholog is 100%.
Bootstrap support for CQW49_RS06230 as seed ortholog is 100%.
```

---

### Group of orthologs #908. Best score 275 bits Score difference with first non-orthologous sequence - AM1\_locus\_tags.txt:275 OB3b\_locus\_tags.txt:9

```
META1_4215          	100.00%		CQW49_RS22045       	100.00%
                    	       		CQW49_RS24115       	43.96%
Bootstrap support for META1_4215 as seed ortholog is 100%.
Bootstrap support for CQW49_RS22045 as seed ortholog is 62%.
Alternative seed ortholog is CQW49_RS09685 (9 bits away from this cluster)
```

---

### Group of orthologs #909. Best score 275 bits Score difference with first non-orthologous sequence - AM1\_locus\_tags.txt:275 OB3b\_locus\_tags.txt:229

```
META1_0733          	100.00%		CQW49_RS02240       	100.00%
Bootstrap support for META1_0733 as seed ortholog is 100%.
Bootstrap support for CQW49_RS02240 as seed ortholog is 100%.
```

---

### Group of orthologs #910. Best score 275 bits Score difference with first non-orthologous sequence - AM1\_locus\_tags.txt:223 OB3b\_locus\_tags.txt:275

```
META1_0291          	100.00%		CQW49_RS12435       	100.00%
Bootstrap support for META1_0291 as seed ortholog is 100%.
Bootstrap support for CQW49_RS12435 as seed ortholog is 100%.
```

---

### Group of orthologs #911. Best score 275 bits Score difference with first non-orthologous sequence - AM1\_locus\_tags.txt:275 OB3b\_locus\_tags.txt:275

```
META1_2235          	100.00%		CQW49_RS04625       	100.00%
Bootstrap support for META1_2235 as seed ortholog is 100%.
Bootstrap support for CQW49_RS04625 as seed ortholog is 100%.
```

---

### Group of orthologs #912. Best score 275 bits Score difference with first non-orthologous sequence - AM1\_locus\_tags.txt:275 OB3b\_locus\_tags.txt:275

```
META1_3188          	100.00%		CQW49_RS02925       	100.00%
Bootstrap support for META1_3188 as seed ortholog is 100%.
Bootstrap support for CQW49_RS02925 as seed ortholog is 100%.
```

---

### Group of orthologs #913. Best score 275 bits Score difference with first non-orthologous sequence - AM1\_locus\_tags.txt:275 OB3b\_locus\_tags.txt:275

```
META1_1745          	100.00%		CQW49_RS10295       	100.00%
Bootstrap support for META1_1745 as seed ortholog is 100%.
Bootstrap support for CQW49_RS10295 as seed ortholog is 100%.
```

---

### Group of orthologs #914. Best score 275 bits Score difference with first non-orthologous sequence - AM1\_locus\_tags.txt:275 OB3b\_locus\_tags.txt:275

```
META1_1411          	100.00%		CQW49_RS20620       	100.00%
Bootstrap support for META1_1411 as seed ortholog is 100%.
Bootstrap support for CQW49_RS20620 as seed ortholog is 100%.
```

---

### Group of orthologs #915. Best score 274 bits Score difference with first non-orthologous sequence - AM1\_locus\_tags.txt:274 OB3b\_locus\_tags.txt:274

```
META1_2059          	100.00%		CQW49_RS03825       	100.00%
Bootstrap support for META1_2059 as seed ortholog is 100%.
Bootstrap support for CQW49_RS03825 as seed ortholog is 100%.
```

---

### Group of orthologs #916. Best score 274 bits Score difference with first non-orthologous sequence - AM1\_locus\_tags.txt:157 OB3b\_locus\_tags.txt:274

```
META1_1339          	100.00%		CQW49_RS09300       	100.00%
Bootstrap support for META1_1339 as seed ortholog is 100%.
Bootstrap support for CQW49_RS09300 as seed ortholog is 100%.
```

---

### Group of orthologs #917. Best score 274 bits Score difference with first non-orthologous sequence - AM1\_locus\_tags.txt:175 OB3b\_locus\_tags.txt:274

```
META1_3141          	100.00%		CQW49_RS01175       	100.00%
Bootstrap support for META1_3141 as seed ortholog is 99%.
Bootstrap support for CQW49_RS01175 as seed ortholog is 100%.
```

---

### Group of orthologs #918. Best score 274 bits Score difference with first non-orthologous sequence - AM1\_locus\_tags.txt:167 OB3b\_locus\_tags.txt:214

```
META1_2195          	100.00%		CQW49_RS09325       	100.00%
Bootstrap support for META1_2195 as seed ortholog is 99%.
Bootstrap support for CQW49_RS09325 as seed ortholog is 99%.
```

---

### Group of orthologs #919. Best score 274 bits Score difference with first non-orthologous sequence - AM1\_locus\_tags.txt:274 OB3b\_locus\_tags.txt:274

```
META1_3315          	100.00%		CQW49_RS11560       	100.00%
Bootstrap support for META1_3315 as seed ortholog is 100%.
Bootstrap support for CQW49_RS11560 as seed ortholog is 100%.
```

---

### Group of orthologs #920. Best score 274 bits Score difference with first non-orthologous sequence - AM1\_locus\_tags.txt:274 OB3b\_locus\_tags.txt:274

```
META1_2848          	100.00%		CQW49_RS18640       	100.00%
Bootstrap support for META1_2848 as seed ortholog is 100%.
Bootstrap support for CQW49_RS18640 as seed ortholog is 100%.
```

---

### Group of orthologs #921. Best score 273 bits Score difference with first non-orthologous sequence - AM1\_locus\_tags.txt:89 OB3b\_locus\_tags.txt:108

```
META1_2234          	100.00%		CQW49_RS15755       	100.00%
Bootstrap support for META1_2234 as seed ortholog is 90%.
Bootstrap support for CQW49_RS15755 as seed ortholog is 96%.
```

---

### Group of orthologs #922. Best score 273 bits Score difference with first non-orthologous sequence - AM1\_locus\_tags.txt:137 OB3b\_locus\_tags.txt:6

```
META1_3893          	100.00%		CQW49_RS16835       	100.00%
Bootstrap support for META1_3893 as seed ortholog is 100%.
Bootstrap support for CQW49_RS16835 as seed ortholog is 56%.
Alternative seed ortholog is CQW49_RS22015 (6 bits away from this cluster)
```

---

### Group of orthologs #923. Best score 273 bits Score difference with first non-orthologous sequence - AM1\_locus\_tags.txt:105 OB3b\_locus\_tags.txt:273

```
META1_4945          	100.00%		CQW49_RS19370       	100.00%
Bootstrap support for META1_4945 as seed ortholog is 99%.
Bootstrap support for CQW49_RS19370 as seed ortholog is 100%.
```

---

### Group of orthologs #924. Best score 273 bits Score difference with first non-orthologous sequence - AM1\_locus\_tags.txt:64 OB3b\_locus\_tags.txt:148

```
META1_4822          	100.00%		CQW49_RS22240       	100.00%
Bootstrap support for META1_4822 as seed ortholog is 98%.
Bootstrap support for CQW49_RS22240 as seed ortholog is 100%.
```

---

### Group of orthologs #925. Best score 272 bits Score difference with first non-orthologous sequence - AM1\_locus\_tags.txt:226 OB3b\_locus\_tags.txt:272

```
META1_3176          	100.00%		CQW49_RS00885       	100.00%
Bootstrap support for META1_3176 as seed ortholog is 100%.
Bootstrap support for CQW49_RS00885 as seed ortholog is 100%.
```

---

### Group of orthologs #926. Best score 272 bits Score difference with first non-orthologous sequence - AM1\_locus\_tags.txt:272 OB3b\_locus\_tags.txt:272

```
META1_1977          	100.00%		CQW49_RS17755       	100.00%
Bootstrap support for META1_1977 as seed ortholog is 100%.
Bootstrap support for CQW49_RS17755 as seed ortholog is 100%.
```

---

### Group of orthologs #927. Best score 272 bits Score difference with first non-orthologous sequence - AM1\_locus\_tags.txt:272 OB3b\_locus\_tags.txt:272

```
META1_2264          	100.00%		CQW49_RS20185       	100.00%
Bootstrap support for META1_2264 as seed ortholog is 100%.
Bootstrap support for CQW49_RS20185 as seed ortholog is 100%.
```

---

### Group of orthologs #928. Best score 272 bits Score difference with first non-orthologous sequence - AM1\_locus\_tags.txt:75 OB3b\_locus\_tags.txt:272

```
META1_2472          	100.00%		CQW49_RS21290       	100.00%
Bootstrap support for META1_2472 as seed ortholog is 99%.
Bootstrap support for CQW49_RS21290 as seed ortholog is 100%.
```

---

### Group of orthologs #929. Best score 271 bits Score difference with first non-orthologous sequence - AM1\_locus\_tags.txt:271 OB3b\_locus\_tags.txt:271

```
META1_1380          	100.00%		CQW49_RS06465       	100.00%
Bootstrap support for META1_1380 as seed ortholog is 100%.
Bootstrap support for CQW49_RS06465 as seed ortholog is 100%.
```

---

### Group of orthologs #930. Best score 271 bits Score difference with first non-orthologous sequence - AM1\_locus\_tags.txt:117 OB3b\_locus\_tags.txt:271

```
META1_0508          	100.00%		CQW49_RS19735       	100.00%
Bootstrap support for META1_0508 as seed ortholog is 99%.
Bootstrap support for CQW49_RS19735 as seed ortholog is 100%.
```

---

### Group of orthologs #931. Best score 270 bits Score difference with first non-orthologous sequence - AM1\_locus\_tags.txt:270 OB3b\_locus\_tags.txt:270

```
META1_2060          	100.00%		CQW49_RS04305       	100.00%
Bootstrap support for META1_2060 as seed ortholog is 100%.
Bootstrap support for CQW49_RS04305 as seed ortholog is 100%.
```

---

### Group of orthologs #932. Best score 270 bits Score difference with first non-orthologous sequence - AM1\_locus\_tags.txt:270 OB3b\_locus\_tags.txt:270

```
META1_0335          	100.00%		CQW49_RS12645       	100.00%
Bootstrap support for META1_0335 as seed ortholog is 100%.
Bootstrap support for CQW49_RS12645 as seed ortholog is 100%.
```

---

### Group of orthologs #933. Best score 270 bits Score difference with first non-orthologous sequence - AM1\_locus\_tags.txt:270 OB3b\_locus\_tags.txt:219

```
META1_0980          	100.00%		CQW49_RS12325       	100.00%
Bootstrap support for META1_0980 as seed ortholog is 100%.
Bootstrap support for CQW49_RS12325 as seed ortholog is 100%.
```

---

### Group of orthologs #934. Best score 270 bits Score difference with first non-orthologous sequence - AM1\_locus\_tags.txt:168 OB3b\_locus\_tags.txt:270

```
META1_4917          	100.00%		CQW49_RS10260       	100.00%
Bootstrap support for META1_4917 as seed ortholog is 100%.
Bootstrap support for CQW49_RS10260 as seed ortholog is 100%.
```

---

### Group of orthologs #935. Best score 269 bits Score difference with first non-orthologous sequence - AM1\_locus\_tags.txt:269 OB3b\_locus\_tags.txt:269

```
META1_0470          	100.00%		CQW49_RS04410       	100.00%
Bootstrap support for META1_0470 as seed ortholog is 100%.
Bootstrap support for CQW49_RS04410 as seed ortholog is 100%.
```

---

### Group of orthologs #936. Best score 269 bits Score difference with first non-orthologous sequence - AM1\_locus\_tags.txt:269 OB3b\_locus\_tags.txt:269

```
META1_4291          	100.00%		CQW49_RS01620       	100.00%
Bootstrap support for META1_4291 as seed ortholog is 100%.
Bootstrap support for CQW49_RS01620 as seed ortholog is 100%.
```

---

### Group of orthologs #937. Best score 269 bits Score difference with first non-orthologous sequence - AM1\_locus\_tags.txt:269 OB3b\_locus\_tags.txt:269

```
META1_1528          	100.00%		CQW49_RS19225       	100.00%
Bootstrap support for META1_1528 as seed ortholog is 100%.
Bootstrap support for CQW49_RS19225 as seed ortholog is 100%.
```

---

### Group of orthologs #938. Best score 269 bits Score difference with first non-orthologous sequence - AM1\_locus\_tags.txt:269 OB3b\_locus\_tags.txt:269

```
META1_1518          	100.00%		CQW49_RS19310       	100.00%
Bootstrap support for META1_1518 as seed ortholog is 100%.
Bootstrap support for CQW49_RS19310 as seed ortholog is 100%.
```

---

### Group of orthologs #939. Best score 269 bits Score difference with first non-orthologous sequence - AM1\_locus\_tags.txt:269 OB3b\_locus\_tags.txt:269

```
META1_2449          	100.00%		CQW49_RS17425       	100.00%
Bootstrap support for META1_2449 as seed ortholog is 100%.
Bootstrap support for CQW49_RS17425 as seed ortholog is 100%.
```

---

### Group of orthologs #940. Best score 269 bits Score difference with first non-orthologous sequence - AM1\_locus\_tags.txt:269 OB3b\_locus\_tags.txt:269

```
META2_0738          	100.00%		CQW49_RS15870       	100.00%
Bootstrap support for META2_0738 as seed ortholog is 100%.
Bootstrap support for CQW49_RS15870 as seed ortholog is 100%.
```

---

### Group of orthologs #941. Best score 268 bits Score difference with first non-orthologous sequence - AM1\_locus\_tags.txt:268 OB3b\_locus\_tags.txt:268

```
META1_0669          	100.00%		CQW49_RS17625       	100.00%
Bootstrap support for META1_0669 as seed ortholog is 100%.
Bootstrap support for CQW49_RS17625 as seed ortholog is 100%.
```

---

### Group of orthologs #942. Best score 268 bits Score difference with first non-orthologous sequence - AM1\_locus\_tags.txt:184 OB3b\_locus\_tags.txt:151

```
META1_3890          	100.00%		CQW49_RS16830       	100.00%
Bootstrap support for META1_3890 as seed ortholog is 100%.
Bootstrap support for CQW49_RS16830 as seed ortholog is 100%.
```

---

### Group of orthologs #943. Best score 267 bits Score difference with first non-orthologous sequence - AM1\_locus\_tags.txt:267 OB3b\_locus\_tags.txt:267

```
META1_2475          	100.00%		CQW49_RS21085       	100.00%
Bootstrap support for META1_2475 as seed ortholog is 100%.
Bootstrap support for CQW49_RS21085 as seed ortholog is 100%.
```

---

### Group of orthologs #944. Best score 266 bits Score difference with first non-orthologous sequence - AM1\_locus\_tags.txt:266 OB3b\_locus\_tags.txt:266

```
META1_1469          	100.00%		CQW49_RS10555       	100.00%
Bootstrap support for META1_1469 as seed ortholog is 100%.
Bootstrap support for CQW49_RS10555 as seed ortholog is 100%.
```

---

### Group of orthologs #945. Best score 266 bits Score difference with first non-orthologous sequence - AM1\_locus\_tags.txt:266 OB3b\_locus\_tags.txt:209

```
META1_5176          	100.00%		CQW49_RS02460       	100.00%
Bootstrap support for META1_5176 as seed ortholog is 100%.
Bootstrap support for CQW49_RS02460 as seed ortholog is 100%.
```

---

### Group of orthologs #946. Best score 266 bits Score difference with first non-orthologous sequence - AM1\_locus\_tags.txt:266 OB3b\_locus\_tags.txt:266

```
META1_1978          	100.00%		CQW49_RS17760       	100.00%
Bootstrap support for META1_1978 as seed ortholog is 100%.
Bootstrap support for CQW49_RS17760 as seed ortholog is 100%.
```

---

### Group of orthologs #947. Best score 266 bits Score difference with first non-orthologous sequence - AM1\_locus\_tags.txt:266 OB3b\_locus\_tags.txt:266

```
META1_2847          	100.00%		CQW49_RS18635       	100.00%
Bootstrap support for META1_2847 as seed ortholog is 100%.
Bootstrap support for CQW49_RS18635 as seed ortholog is 100%.
```

---

### Group of orthologs #948. Best score 265 bits Score difference with first non-orthologous sequence - AM1\_locus\_tags.txt:265 OB3b\_locus\_tags.txt:265

```
META1_2344          	100.00%		CQW49_RS08630       	100.00%
Bootstrap support for META1_2344 as seed ortholog is 100%.
Bootstrap support for CQW49_RS08630 as seed ortholog is 100%.
```

---

### Group of orthologs #949. Best score 265 bits Score difference with first non-orthologous sequence - AM1\_locus\_tags.txt:265 OB3b\_locus\_tags.txt:265

```
META1_2149          	100.00%		CQW49_RS10540       	100.00%
Bootstrap support for META1_2149 as seed ortholog is 100%.
Bootstrap support for CQW49_RS10540 as seed ortholog is 100%.
```

---

### Group of orthologs #950. Best score 265 bits Score difference with first non-orthologous sequence - AM1\_locus\_tags.txt:265 OB3b\_locus\_tags.txt:265

```
META1_4321          	100.00%		CQW49_RS02020       	100.00%
Bootstrap support for META1_4321 as seed ortholog is 100%.
Bootstrap support for CQW49_RS02020 as seed ortholog is 100%.
```

---

### Group of orthologs #951. Best score 265 bits Score difference with first non-orthologous sequence - AM1\_locus\_tags.txt:265 OB3b\_locus\_tags.txt:265

```
META1_4581          	100.00%		CQW49_RS01330       	100.00%
Bootstrap support for META1_4581 as seed ortholog is 100%.
Bootstrap support for CQW49_RS01330 as seed ortholog is 100%.
```

---

### Group of orthologs #952. Best score 265 bits Score difference with first non-orthologous sequence - AM1\_locus\_tags.txt:265 OB3b\_locus\_tags.txt:265

```
META1_5115          	100.00%		CQW49_RS01360       	100.00%
Bootstrap support for META1_5115 as seed ortholog is 100%.
Bootstrap support for CQW49_RS01360 as seed ortholog is 100%.
```

---

### Group of orthologs #953. Best score 265 bits Score difference with first non-orthologous sequence - AM1\_locus\_tags.txt:265 OB3b\_locus\_tags.txt:265

```
META1_2250          	100.00%		CQW49_RS20355       	100.00%
Bootstrap support for META1_2250 as seed ortholog is 100%.
Bootstrap support for CQW49_RS20355 as seed ortholog is 100%.
```

---

### Group of orthologs #954. Best score 264 bits Score difference with first non-orthologous sequence - AM1\_locus\_tags.txt:264 OB3b\_locus\_tags.txt:264

```
META1_0425          	100.00%		CQW49_RS00270       	100.00%
Bootstrap support for META1_0425 as seed ortholog is 100%.
Bootstrap support for CQW49_RS00270 as seed ortholog is 100%.
```

---

### Group of orthologs #955. Best score 264 bits Score difference with first non-orthologous sequence - AM1\_locus\_tags.txt:264 OB3b\_locus\_tags.txt:264

```
META1_1427          	100.00%		CQW49_RS06425       	100.00%
Bootstrap support for META1_1427 as seed ortholog is 100%.
Bootstrap support for CQW49_RS06425 as seed ortholog is 100%.
```

---

### Group of orthologs #956. Best score 264 bits Score difference with first non-orthologous sequence - AM1\_locus\_tags.txt:197 OB3b\_locus\_tags.txt:264

```
META1_0628          	100.00%		CQW49_RS12780       	100.00%
Bootstrap support for META1_0628 as seed ortholog is 100%.
Bootstrap support for CQW49_RS12780 as seed ortholog is 100%.
```

---

### Group of orthologs #957. Best score 264 bits Score difference with first non-orthologous sequence - AM1\_locus\_tags.txt:179 OB3b\_locus\_tags.txt:213

```
META1_4242          	100.00%		CQW49_RS00105       	100.00%
Bootstrap support for META1_4242 as seed ortholog is 100%.
Bootstrap support for CQW49_RS00105 as seed ortholog is 100%.
```

---

### Group of orthologs #958. Best score 264 bits Score difference with first non-orthologous sequence - AM1\_locus\_tags.txt:264 OB3b\_locus\_tags.txt:264

```
META1_2817          	100.00%		CQW49_RS10805       	100.00%
Bootstrap support for META1_2817 as seed ortholog is 100%.
Bootstrap support for CQW49_RS10805 as seed ortholog is 100%.
```

---

### Group of orthologs #959. Best score 264 bits Score difference with first non-orthologous sequence - AM1\_locus\_tags.txt:213 OB3b\_locus\_tags.txt:264

```
META1_2207          	100.00%		CQW49_RS16355       	100.00%
Bootstrap support for META1_2207 as seed ortholog is 100%.
Bootstrap support for CQW49_RS16355 as seed ortholog is 100%.
```

---

### Group of orthologs #960. Best score 264 bits Score difference with first non-orthologous sequence - AM1\_locus\_tags.txt:165 OB3b\_locus\_tags.txt:139

```
META1_2820          	100.00%		CQW49_RS16865       	100.00%
Bootstrap support for META1_2820 as seed ortholog is 99%.
Bootstrap support for CQW49_RS16865 as seed ortholog is 99%.
```

---

### Group of orthologs #961. Best score 264 bits Score difference with first non-orthologous sequence - AM1\_locus\_tags.txt:264 OB3b\_locus\_tags.txt:264

```
META1_5307          	100.00%		CQW49_RS06525       	100.00%
Bootstrap support for META1_5307 as seed ortholog is 100%.
Bootstrap support for CQW49_RS06525 as seed ortholog is 100%.
```

---

### Group of orthologs #962. Best score 263 bits Score difference with first non-orthologous sequence - AM1\_locus\_tags.txt:263 OB3b\_locus\_tags.txt:263

```
META1_1768          	100.00%		CQW49_RS13890       	100.00%
Bootstrap support for META1_1768 as seed ortholog is 100%.
Bootstrap support for CQW49_RS13890 as seed ortholog is 100%.
```

---

### Group of orthologs #963. Best score 263 bits Score difference with first non-orthologous sequence - AM1\_locus\_tags.txt:263 OB3b\_locus\_tags.txt:263

```
META1_1688          	100.00%		CQW49_RS17055       	100.00%
Bootstrap support for META1_1688 as seed ortholog is 100%.
Bootstrap support for CQW49_RS17055 as seed ortholog is 100%.
```

---

### Group of orthologs #964. Best score 263 bits Score difference with first non-orthologous sequence - AM1\_locus\_tags.txt:263 OB3b\_locus\_tags.txt:263

```
META1_2299          	100.00%		CQW49_RS14275       	100.00%
Bootstrap support for META1_2299 as seed ortholog is 100%.
Bootstrap support for CQW49_RS14275 as seed ortholog is 100%.
```

---

### Group of orthologs #965. Best score 263 bits Score difference with first non-orthologous sequence - AM1\_locus\_tags.txt:263 OB3b\_locus\_tags.txt:263

```
META1_4708          	100.00%		CQW49_RS10095       	100.00%
Bootstrap support for META1_4708 as seed ortholog is 100%.
Bootstrap support for CQW49_RS10095 as seed ortholog is 100%.
```

---

### Group of orthologs #966. Best score 263 bits Score difference with first non-orthologous sequence - AM1\_locus\_tags.txt:263 OB3b\_locus\_tags.txt:263

```
META1_4724          	100.00%		CQW49_RS17265       	100.00%
Bootstrap support for META1_4724 as seed ortholog is 100%.
Bootstrap support for CQW49_RS17265 as seed ortholog is 100%.
```

---

### Group of orthologs #967. Best score 262 bits Score difference with first non-orthologous sequence - AM1\_locus\_tags.txt:262 OB3b\_locus\_tags.txt:262

```
META1_0625          	100.00%		CQW49_RS06065       	100.00%
Bootstrap support for META1_0625 as seed ortholog is 100%.
Bootstrap support for CQW49_RS06065 as seed ortholog is 100%.
```

---

### Group of orthologs #968. Best score 262 bits Score difference with first non-orthologous sequence - AM1\_locus\_tags.txt:262 OB3b\_locus\_tags.txt:262

```
META1_0821          	100.00%		CQW49_RS17030       	100.00%
Bootstrap support for META1_0821 as seed ortholog is 100%.
Bootstrap support for CQW49_RS17030 as seed ortholog is 100%.
```

---

### Group of orthologs #969. Best score 262 bits Score difference with first non-orthologous sequence - AM1\_locus\_tags.txt:262 OB3b\_locus\_tags.txt:262

```
META1_2439          	100.00%		CQW49_RS14360       	100.00%
Bootstrap support for META1_2439 as seed ortholog is 100%.
Bootstrap support for CQW49_RS14360 as seed ortholog is 100%.
```

---

### Group of orthologs #970. Best score 261 bits Score difference with first non-orthologous sequence - AM1\_locus\_tags.txt:182 OB3b\_locus\_tags.txt:194

```
META1_3609          	100.00%		CQW49_RS21460       	100.00%
Bootstrap support for META1_3609 as seed ortholog is 100%.
Bootstrap support for CQW49_RS21460 as seed ortholog is 100%.
```

---

### Group of orthologs #971. Best score 260 bits Score difference with first non-orthologous sequence - AM1\_locus\_tags.txt:260 OB3b\_locus\_tags.txt:260

```
META1_3557          	100.00%		CQW49_RS22225       	100.00%
META1_4820          	27.65%		
Bootstrap support for META1_3557 as seed ortholog is 100%.
Bootstrap support for CQW49_RS22225 as seed ortholog is 100%.
```

---

### Group of orthologs #972. Best score 260 bits Score difference with first non-orthologous sequence - AM1\_locus\_tags.txt:260 OB3b\_locus\_tags.txt:260

```
META1_1715          	100.00%		CQW49_RS01305       	100.00%
Bootstrap support for META1_1715 as seed ortholog is 100%.
Bootstrap support for CQW49_RS01305 as seed ortholog is 100%.
```

---

### Group of orthologs #973. Best score 260 bits Score difference with first non-orthologous sequence - AM1\_locus\_tags.txt:260 OB3b\_locus\_tags.txt:260

```
META1_0197          	100.00%		CQW49_RS08960       	100.00%
Bootstrap support for META1_0197 as seed ortholog is 100%.
Bootstrap support for CQW49_RS08960 as seed ortholog is 100%.
```

---

### Group of orthologs #974. Best score 260 bits Score difference with first non-orthologous sequence - AM1\_locus\_tags.txt:260 OB3b\_locus\_tags.txt:260

```
META1_0715          	100.00%		CQW49_RS12160       	100.00%
Bootstrap support for META1_0715 as seed ortholog is 100%.
Bootstrap support for CQW49_RS12160 as seed ortholog is 100%.
```

---

### Group of orthologs #975. Best score 260 bits Score difference with first non-orthologous sequence - AM1\_locus\_tags.txt:124 OB3b\_locus\_tags.txt:174

```
META1_1370          	100.00%		CQW49_RS12755       	100.00%
Bootstrap support for META1_1370 as seed ortholog is 99%.
Bootstrap support for CQW49_RS12755 as seed ortholog is 100%.
```

---

### Group of orthologs #976. Best score 260 bits Score difference with first non-orthologous sequence - AM1\_locus\_tags.txt:260 OB3b\_locus\_tags.txt:260

```
META1_3483          	100.00%		CQW49_RS02770       	100.00%
Bootstrap support for META1_3483 as seed ortholog is 100%.
Bootstrap support for CQW49_RS02770 as seed ortholog is 100%.
```

---

### Group of orthologs #977. Best score 260 bits Score difference with first non-orthologous sequence - AM1\_locus\_tags.txt:260 OB3b\_locus\_tags.txt:260

```
META1_0196          	100.00%		CQW49_RS19350       	100.00%
Bootstrap support for META1_0196 as seed ortholog is 100%.
Bootstrap support for CQW49_RS19350 as seed ortholog is 100%.
```

---

### Group of orthologs #978. Best score 260 bits Score difference with first non-orthologous sequence - AM1\_locus\_tags.txt:169 OB3b\_locus\_tags.txt:260

```
META1_0456          	100.00%		CQW49_RS18710       	100.00%
Bootstrap support for META1_0456 as seed ortholog is 100%.
Bootstrap support for CQW49_RS18710 as seed ortholog is 100%.
```

---

### Group of orthologs #979. Best score 260 bits Score difference with first non-orthologous sequence - AM1\_locus\_tags.txt:135 OB3b\_locus\_tags.txt:158

```
META1_1782          	100.00%		CQW49_RS19325       	100.00%
Bootstrap support for META1_1782 as seed ortholog is 100%.
Bootstrap support for CQW49_RS19325 as seed ortholog is 100%.
```

---

### Group of orthologs #980. Best score 260 bits Score difference with first non-orthologous sequence - AM1\_locus\_tags.txt:45 OB3b\_locus\_tags.txt:104

```
META1_3546          	100.00%		CQW49_RS13555       	100.00%
Bootstrap support for META1_3546 as seed ortholog is 94%.
Bootstrap support for CQW49_RS13555 as seed ortholog is 99%.
```

---

### Group of orthologs #981. Best score 260 bits Score difference with first non-orthologous sequence - AM1\_locus\_tags.txt:260 OB3b\_locus\_tags.txt:260

```
META1_3380          	100.00%		CQW49_RS19570       	100.00%
Bootstrap support for META1_3380 as seed ortholog is 100%.
Bootstrap support for CQW49_RS19570 as seed ortholog is 100%.
```

---

### Group of orthologs #982. Best score 259 bits Score difference with first non-orthologous sequence - AM1\_locus\_tags.txt:259 OB3b\_locus\_tags.txt:259

```
META1_2353          	100.00%		CQW49_RS08620       	100.00%
Bootstrap support for META1_2353 as seed ortholog is 100%.
Bootstrap support for CQW49_RS08620 as seed ortholog is 100%.
```

---

### Group of orthologs #983. Best score 259 bits Score difference with first non-orthologous sequence - AM1\_locus\_tags.txt:259 OB3b\_locus\_tags.txt:259

```
META1_2243          	100.00%		CQW49_RS19545       	100.00%
Bootstrap support for META1_2243 as seed ortholog is 100%.
Bootstrap support for CQW49_RS19545 as seed ortholog is 100%.
```

---

### Group of orthologs #984. Best score 259 bits Score difference with first non-orthologous sequence - AM1\_locus\_tags.txt:54 OB3b\_locus\_tags.txt:110

```
META1_3085          	100.00%		CQW49_RS16400       	100.00%
Bootstrap support for META1_3085 as seed ortholog is 97%.
Bootstrap support for CQW49_RS16400 as seed ortholog is 99%.
```

---

### Group of orthologs #985. Best score 258 bits Score difference with first non-orthologous sequence - AM1\_locus\_tags.txt:258 OB3b\_locus\_tags.txt:258

```
META1_0853          	100.00%		CQW49_RS05345       	100.00%
Bootstrap support for META1_0853 as seed ortholog is 100%.
Bootstrap support for CQW49_RS05345 as seed ortholog is 100%.
```

---

### Group of orthologs #986. Best score 258 bits Score difference with first non-orthologous sequence - AM1\_locus\_tags.txt:162 OB3b\_locus\_tags.txt:258

```
META1_2226          	100.00%		CQW49_RS03475       	100.00%
Bootstrap support for META1_2226 as seed ortholog is 100%.
Bootstrap support for CQW49_RS03475 as seed ortholog is 100%.
```

---

### Group of orthologs #987. Best score 258 bits Score difference with first non-orthologous sequence - AM1\_locus\_tags.txt:258 OB3b\_locus\_tags.txt:258

```
META1_0318          	100.00%		CQW49_RS18840       	100.00%
Bootstrap support for META1_0318 as seed ortholog is 100%.
Bootstrap support for CQW49_RS18840 as seed ortholog is 100%.
```

---

### Group of orthologs #988. Best score 258 bits Score difference with first non-orthologous sequence - AM1\_locus\_tags.txt:258 OB3b\_locus\_tags.txt:258

```
META1_5175          	100.00%		CQW49_RS02455       	100.00%
Bootstrap support for META1_5175 as seed ortholog is 100%.
Bootstrap support for CQW49_RS02455 as seed ortholog is 100%.
```

---

### Group of orthologs #989. Best score 258 bits Score difference with first non-orthologous sequence - AM1\_locus\_tags.txt:258 OB3b\_locus\_tags.txt:191

```
META1_3436          	100.00%		CQW49_RS16540       	100.00%
Bootstrap support for META1_3436 as seed ortholog is 100%.
Bootstrap support for CQW49_RS16540 as seed ortholog is 100%.
```

---

### Group of orthologs #990. Best score 257 bits Score difference with first non-orthologous sequence - AM1\_locus\_tags.txt:257 OB3b\_locus\_tags.txt:257

```
META1_2919          	100.00%		CQW49_RS16200       	100.00%
Bootstrap support for META1_2919 as seed ortholog is 100%.
Bootstrap support for CQW49_RS16200 as seed ortholog is 100%.
```

---

### Group of orthologs #991. Best score 257 bits Score difference with first non-orthologous sequence - AM1\_locus\_tags.txt:257 OB3b\_locus\_tags.txt:180

```
META1_4632          	100.00%		CQW49_RS12970       	100.00%
Bootstrap support for META1_4632 as seed ortholog is 100%.
Bootstrap support for CQW49_RS12970 as seed ortholog is 100%.
```

---

### Group of orthologs #992. Best score 257 bits Score difference with first non-orthologous sequence - AM1\_locus\_tags.txt:257 OB3b\_locus\_tags.txt:257

```
META1_4533          	100.00%		CQW49_RS14490       	100.00%
Bootstrap support for META1_4533 as seed ortholog is 100%.
Bootstrap support for CQW49_RS14490 as seed ortholog is 100%.
```

---

### Group of orthologs #993. Best score 256 bits Score difference with first non-orthologous sequence - AM1\_locus\_tags.txt:256 OB3b\_locus\_tags.txt:256

```
META1_2071          	100.00%		CQW49_RS00440       	100.00%
Bootstrap support for META1_2071 as seed ortholog is 100%.
Bootstrap support for CQW49_RS00440 as seed ortholog is 100%.
```

---

### Group of orthologs #994. Best score 256 bits Score difference with first non-orthologous sequence - AM1\_locus\_tags.txt:162 OB3b\_locus\_tags.txt:149

```
META1_0813          	100.00%		CQW49_RS12290       	100.00%
Bootstrap support for META1_0813 as seed ortholog is 100%.
Bootstrap support for CQW49_RS12290 as seed ortholog is 100%.
```

---

### Group of orthologs #995. Best score 256 bits Score difference with first non-orthologous sequence - AM1\_locus\_tags.txt:256 OB3b\_locus\_tags.txt:256

```
META1_3618          	100.00%		CQW49_RS01315       	100.00%
Bootstrap support for META1_3618 as seed ortholog is 100%.
Bootstrap support for CQW49_RS01315 as seed ortholog is 100%.
```

---

### Group of orthologs #996. Best score 256 bits Score difference with first non-orthologous sequence - AM1\_locus\_tags.txt:256 OB3b\_locus\_tags.txt:256

```
META1_2825          	100.00%		CQW49_RS11830       	100.00%
Bootstrap support for META1_2825 as seed ortholog is 100%.
Bootstrap support for CQW49_RS11830 as seed ortholog is 100%.
```

---

### Group of orthologs #997. Best score 256 bits Score difference with first non-orthologous sequence - AM1\_locus\_tags.txt:256 OB3b\_locus\_tags.txt:256

```
META1_3110          	100.00%		CQW49_RS13295       	100.00%
Bootstrap support for META1_3110 as seed ortholog is 100%.
Bootstrap support for CQW49_RS13295 as seed ortholog is 100%.
```

---

### Group of orthologs #998. Best score 255 bits Score difference with first non-orthologous sequence - AM1\_locus\_tags.txt:255 OB3b\_locus\_tags.txt:255

```
META1_2230          	100.00%		CQW49_RS00345       	100.00%
Bootstrap support for META1_2230 as seed ortholog is 100%.
Bootstrap support for CQW49_RS00345 as seed ortholog is 100%.
```

---

### Group of orthologs #999. Best score 255 bits Score difference with first non-orthologous sequence - AM1\_locus\_tags.txt:255 OB3b\_locus\_tags.txt:255

```
META1_1722          	100.00%		CQW49_RS04640       	100.00%
Bootstrap support for META1_1722 as seed ortholog is 100%.
Bootstrap support for CQW49_RS04640 as seed ortholog is 100%.
```

---

### Group of orthologs #1000. Best score 255 bits Score difference with first non-orthologous sequence - AM1\_locus\_tags.txt:255 OB3b\_locus\_tags.txt:255

```
META1_2248          	100.00%		CQW49_RS04870       	100.00%
Bootstrap support for META1_2248 as seed ortholog is 100%.
Bootstrap support for CQW49_RS04870 as seed ortholog is 100%.
```

---

### Group of orthologs #1001. Best score 255 bits Score difference with first non-orthologous sequence - AM1\_locus\_tags.txt:255 OB3b\_locus\_tags.txt:255

```
META1_2137          	100.00%		CQW49_RS12875       	100.00%
Bootstrap support for META1_2137 as seed ortholog is 100%.
Bootstrap support for CQW49_RS12875 as seed ortholog is 100%.
```

---

### Group of orthologs #1002. Best score 255 bits Score difference with first non-orthologous sequence - AM1\_locus\_tags.txt:255 OB3b\_locus\_tags.txt:255

```
META1_1516          	100.00%		CQW49_RS16280       	100.00%
Bootstrap support for META1_1516 as seed ortholog is 100%.
Bootstrap support for CQW49_RS16280 as seed ortholog is 100%.
```

---

### Group of orthologs #1003. Best score 255 bits Score difference with first non-orthologous sequence - AM1\_locus\_tags.txt:192 OB3b\_locus\_tags.txt:255

```
META1_2297          	100.00%		CQW49_RS14255       	100.00%
Bootstrap support for META1_2297 as seed ortholog is 100%.
Bootstrap support for CQW49_RS14255 as seed ortholog is 100%.
```

---

### Group of orthologs #1004. Best score 255 bits Score difference with first non-orthologous sequence - AM1\_locus\_tags.txt:255 OB3b\_locus\_tags.txt:255

```
META1_3865          	100.00%		CQW49_RS17465       	100.00%
Bootstrap support for META1_3865 as seed ortholog is 100%.
Bootstrap support for CQW49_RS17465 as seed ortholog is 100%.
```

---

### Group of orthologs #1005. Best score 254 bits Score difference with first non-orthologous sequence - AM1\_locus\_tags.txt:151 OB3b\_locus\_tags.txt:254

```
META1_3606          	100.00%		CQW49_RS21435       	100.00%
Bootstrap support for META1_3606 as seed ortholog is 100%.
Bootstrap support for CQW49_RS21435 as seed ortholog is 100%.
```

---

### Group of orthologs #1006. Best score 254 bits Score difference with first non-orthologous sequence - AM1\_locus\_tags.txt:254 OB3b\_locus\_tags.txt:254

```
META1_4254          	100.00%		CQW49_RS18605       	100.00%
Bootstrap support for META1_4254 as seed ortholog is 100%.
Bootstrap support for CQW49_RS18605 as seed ortholog is 100%.
```

---

### Group of orthologs #1007. Best score 253 bits Score difference with first non-orthologous sequence - AM1\_locus\_tags.txt:253 OB3b\_locus\_tags.txt:253

```
META1_4207          	100.00%		CQW49_RS10790       	100.00%
META2_1008          	8.33%		
META1_1206          	7.94%		
META1_2585          	6.35%		
Bootstrap support for META1_4207 as seed ortholog is 100%.
Bootstrap support for CQW49_RS10790 as seed ortholog is 100%.
```

---

### Group of orthologs #1008. Best score 253 bits Score difference with first non-orthologous sequence - AM1\_locus\_tags.txt:253 OB3b\_locus\_tags.txt:253

```
META1_0339          	100.00%		CQW49_RS09330       	100.00%
Bootstrap support for META1_0339 as seed ortholog is 100%.
Bootstrap support for CQW49_RS09330 as seed ortholog is 100%.
```

---

### Group of orthologs #1009. Best score 253 bits Score difference with first non-orthologous sequence - AM1\_locus\_tags.txt:253 OB3b\_locus\_tags.txt:253

```
META1_3357          	100.00%		CQW49_RS00195       	100.00%
Bootstrap support for META1_3357 as seed ortholog is 100%.
Bootstrap support for CQW49_RS00195 as seed ortholog is 100%.
```

---

### Group of orthologs #1010. Best score 253 bits Score difference with first non-orthologous sequence - AM1\_locus\_tags.txt:148 OB3b\_locus\_tags.txt:253

```
META1_0385          	100.00%		CQW49_RS23910       	100.00%
Bootstrap support for META1_0385 as seed ortholog is 100%.
Bootstrap support for CQW49_RS23910 as seed ortholog is 100%.
```

---

### Group of orthologs #1011. Best score 252 bits Score difference with first non-orthologous sequence - AM1\_locus\_tags.txt:72 OB3b\_locus\_tags.txt:111

```
META1_1051          	100.00%		CQW49_RS19445       	100.00%
META1_3360          	28.74%		
META1_4457          	28.22%		
META1_2522          	27.71%		
META1_3653          	25.29%		
META1_2286          	23.12%		
META1_0082          	22.48%		
META1_1104          	22.22%		
META1_0534          	21.97%		
META1_2055          	19.80%		
META1_3876          	18.65%		
META1_0493          	18.39%		
META1_0585          	16.73%		
META1_0572          	8.68%		
META1_5140          	6.13%		
META1_5118          	5.49%		
META1_1343          	5.36%		
Bootstrap support for META1_1051 as seed ortholog is 99%.
Bootstrap support for CQW49_RS19445 as seed ortholog is 100%.
```

---

### Group of orthologs #1012. Best score 252 bits Score difference with first non-orthologous sequence - AM1\_locus\_tags.txt:42 OB3b\_locus\_tags.txt:252

```
META1_1643          	100.00%		CQW49_RS06550       	100.00%
Bootstrap support for META1_1643 as seed ortholog is 82%.
Bootstrap support for CQW49_RS06550 as seed ortholog is 100%.
```

---

### Group of orthologs #1013. Best score 252 bits Score difference with first non-orthologous sequence - AM1\_locus\_tags.txt:252 OB3b\_locus\_tags.txt:252

```
META1_3152          	100.00%		CQW49_RS11290       	100.00%
Bootstrap support for META1_3152 as seed ortholog is 100%.
Bootstrap support for CQW49_RS11290 as seed ortholog is 100%.
```

---

### Group of orthologs #1014. Best score 252 bits Score difference with first non-orthologous sequence - AM1\_locus\_tags.txt:252 OB3b\_locus\_tags.txt:252

```
META1_5193          	100.00%		CQW49_RS03335       	100.00%
Bootstrap support for META1_5193 as seed ortholog is 100%.
Bootstrap support for CQW49_RS03335 as seed ortholog is 100%.
```

---

### Group of orthologs #1015. Best score 252 bits Score difference with first non-orthologous sequence - AM1\_locus\_tags.txt:155 OB3b\_locus\_tags.txt:252

```
META1_3706          	100.00%		CQW49_RS12330       	100.00%
Bootstrap support for META1_3706 as seed ortholog is 100%.
Bootstrap support for CQW49_RS12330 as seed ortholog is 100%.
```

---

### Group of orthologs #1016. Best score 252 bits Score difference with first non-orthologous sequence - AM1\_locus\_tags.txt:252 OB3b\_locus\_tags.txt:252

```
META1_3213          	100.00%		CQW49_RS20540       	100.00%
Bootstrap support for META1_3213 as seed ortholog is 100%.
Bootstrap support for CQW49_RS20540 as seed ortholog is 100%.
```

---

### Group of orthologs #1017. Best score 251 bits Score difference with first non-orthologous sequence - AM1\_locus\_tags.txt:251 OB3b\_locus\_tags.txt:251

```
META1_2864          	100.00%		CQW49_RS06795       	100.00%
Bootstrap support for META1_2864 as seed ortholog is 100%.
Bootstrap support for CQW49_RS06795 as seed ortholog is 100%.
```

---

### Group of orthologs #1018. Best score 251 bits Score difference with first non-orthologous sequence - AM1\_locus\_tags.txt:251 OB3b\_locus\_tags.txt:251

```
META1_2274          	100.00%		CQW49_RS13480       	100.00%
Bootstrap support for META1_2274 as seed ortholog is 100%.
Bootstrap support for CQW49_RS13480 as seed ortholog is 100%.
```

---

### Group of orthologs #1019. Best score 251 bits Score difference with first non-orthologous sequence - AM1\_locus\_tags.txt:251 OB3b\_locus\_tags.txt:195

```
META1_4539          	100.00%		CQW49_RS14455       	100.00%
Bootstrap support for META1_4539 as seed ortholog is 100%.
Bootstrap support for CQW49_RS14455 as seed ortholog is 100%.
```

---

### Group of orthologs #1020. Best score 251 bits Score difference with first non-orthologous sequence - AM1\_locus\_tags.txt:103 OB3b\_locus\_tags.txt:189

```
META2_0014          	100.00%		CQW49_RS12400       	100.00%
Bootstrap support for META2_0014 as seed ortholog is 98%.
Bootstrap support for CQW49_RS12400 as seed ortholog is 99%.
```

---

### Group of orthologs #1021. Best score 250 bits Score difference with first non-orthologous sequence - AM1\_locus\_tags.txt:186 OB3b\_locus\_tags.txt:250

```
META1_1766          	100.00%		CQW49_RS13515       	100.00%
                    	       		CQW49_RS13510       	24.39%
Bootstrap support for META1_1766 as seed ortholog is 100%.
Bootstrap support for CQW49_RS13515 as seed ortholog is 100%.
```

---

### Group of orthologs #1022. Best score 250 bits Score difference with first non-orthologous sequence - AM1\_locus\_tags.txt:48 OB3b\_locus\_tags.txt:138

```
META1_1646          	100.00%		CQW49_RS06555       	100.00%
Bootstrap support for META1_1646 as seed ortholog is 96%.
Bootstrap support for CQW49_RS06555 as seed ortholog is 100%.
```

---

### Group of orthologs #1023. Best score 250 bits Score difference with first non-orthologous sequence - AM1\_locus\_tags.txt:250 OB3b\_locus\_tags.txt:250

```
META1_1409          	100.00%		CQW49_RS16560       	100.00%
Bootstrap support for META1_1409 as seed ortholog is 100%.
Bootstrap support for CQW49_RS16560 as seed ortholog is 100%.
```

---

### Group of orthologs #1024. Best score 250 bits Score difference with first non-orthologous sequence - AM1\_locus\_tags.txt:250 OB3b\_locus\_tags.txt:26

```
META1_5040          	100.00%		CQW49_RS03635       	100.00%
Bootstrap support for META1_5040 as seed ortholog is 100%.
Bootstrap support for CQW49_RS03635 as seed ortholog is 73%.
Alternative seed ortholog is CQW49_RS03065 (26 bits away from this cluster)
```

---

### Group of orthologs #1025. Best score 250 bits Score difference with first non-orthologous sequence - AM1\_locus\_tags.txt:250 OB3b\_locus\_tags.txt:250

```
META1_4702          	100.00%		CQW49_RS11935       	100.00%
Bootstrap support for META1_4702 as seed ortholog is 100%.
Bootstrap support for CQW49_RS11935 as seed ortholog is 100%.
```

---

### Group of orthologs #1026. Best score 250 bits Score difference with first non-orthologous sequence - AM1\_locus\_tags.txt:250 OB3b\_locus\_tags.txt:250

```
META1_4915          	100.00%		CQW49_RS13145       	100.00%
Bootstrap support for META1_4915 as seed ortholog is 100%.
Bootstrap support for CQW49_RS13145 as seed ortholog is 100%.
```

---

### Group of orthologs #1027. Best score 250 bits Score difference with first non-orthologous sequence - AM1\_locus\_tags.txt:114 OB3b\_locus\_tags.txt:88

```
META1_4502          	100.00%		CQW49_RS16925       	100.00%
Bootstrap support for META1_4502 as seed ortholog is 100%.
Bootstrap support for CQW49_RS16925 as seed ortholog is 99%.
```

---

### Group of orthologs #1028. Best score 249 bits Score difference with first non-orthologous sequence - AM1\_locus\_tags.txt:193 OB3b\_locus\_tags.txt:176

```
META1_0580          	100.00%		CQW49_RS07670       	100.00%
Bootstrap support for META1_0580 as seed ortholog is 100%.
Bootstrap support for CQW49_RS07670 as seed ortholog is 100%.
```

---

### Group of orthologs #1029. Best score 248 bits Score difference with first non-orthologous sequence - AM1\_locus\_tags.txt:248 OB3b\_locus\_tags.txt:160

```
META1_4922          	100.00%		CQW49_RS10700       	100.00%
Bootstrap support for META1_4922 as seed ortholog is 100%.
Bootstrap support for CQW49_RS10700 as seed ortholog is 100%.
```

---

### Group of orthologs #1030. Best score 247 bits Score difference with first non-orthologous sequence - AM1\_locus\_tags.txt:247 OB3b\_locus\_tags.txt:247

```
META1_3215          	100.00%		CQW49_RS02640       	100.00%
Bootstrap support for META1_3215 as seed ortholog is 100%.
Bootstrap support for CQW49_RS02640 as seed ortholog is 100%.
```

---

### Group of orthologs #1031. Best score 247 bits Score difference with first non-orthologous sequence - AM1\_locus\_tags.txt:55 OB3b\_locus\_tags.txt:247

```
META1_1283          	100.00%		CQW49_RS13275       	100.00%
Bootstrap support for META1_1283 as seed ortholog is 72%.
Alternative seed ortholog is META1_0533 (55 bits away from this cluster)
Bootstrap support for CQW49_RS13275 as seed ortholog is 100%.
```

---

### Group of orthologs #1032. Best score 247 bits Score difference with first non-orthologous sequence - AM1\_locus\_tags.txt:247 OB3b\_locus\_tags.txt:247

```
META1_3522          	100.00%		CQW49_RS04860       	100.00%
Bootstrap support for META1_3522 as seed ortholog is 100%.
Bootstrap support for CQW49_RS04860 as seed ortholog is 100%.
```

---

### Group of orthologs #1033. Best score 247 bits Score difference with first non-orthologous sequence - AM1\_locus\_tags.txt:247 OB3b\_locus\_tags.txt:247

```
META1_4147          	100.00%		CQW49_RS05570       	100.00%
Bootstrap support for META1_4147 as seed ortholog is 100%.
Bootstrap support for CQW49_RS05570 as seed ortholog is 100%.
```

---

### Group of orthologs #1034. Best score 247 bits Score difference with first non-orthologous sequence - AM1\_locus\_tags.txt:14 OB3b\_locus\_tags.txt:102

```
META1_2838          	100.00%		CQW49_RS12185       	100.00%
Bootstrap support for META1_2838 as seed ortholog is 68%.
Alternative seed ortholog is META1_2527 (14 bits away from this cluster)
Bootstrap support for CQW49_RS12185 as seed ortholog is 99%.
```

---

### Group of orthologs #1035. Best score 246 bits Score difference with first non-orthologous sequence - AM1\_locus\_tags.txt:177 OB3b\_locus\_tags.txt:246

```
META1_2044          	100.00%		CQW49_RS01375       	100.00%
Bootstrap support for META1_2044 as seed ortholog is 100%.
Bootstrap support for CQW49_RS01375 as seed ortholog is 100%.
```

---

### Group of orthologs #1036. Best score 246 bits Score difference with first non-orthologous sequence - AM1\_locus\_tags.txt:246 OB3b\_locus\_tags.txt:246

```
META1_2119          	100.00%		CQW49_RS09940       	100.00%
Bootstrap support for META1_2119 as seed ortholog is 100%.
Bootstrap support for CQW49_RS09940 as seed ortholog is 100%.
```

---

### Group of orthologs #1037. Best score 246 bits Score difference with first non-orthologous sequence - AM1\_locus\_tags.txt:246 OB3b\_locus\_tags.txt:246

```
META1_1729          	100.00%		CQW49_RS12250       	100.00%
Bootstrap support for META1_1729 as seed ortholog is 100%.
Bootstrap support for CQW49_RS12250 as seed ortholog is 100%.
```

---

### Group of orthologs #1038. Best score 246 bits Score difference with first non-orthologous sequence - AM1\_locus\_tags.txt:246 OB3b\_locus\_tags.txt:246

```
META1_4624          	100.00%		CQW49_RS02185       	100.00%
Bootstrap support for META1_4624 as seed ortholog is 100%.
Bootstrap support for CQW49_RS02185 as seed ortholog is 100%.
```

---

### Group of orthologs #1039. Best score 246 bits Score difference with first non-orthologous sequence - AM1\_locus\_tags.txt:149 OB3b\_locus\_tags.txt:177

```
META1_3474          	100.00%		CQW49_RS10070       	100.00%
Bootstrap support for META1_3474 as seed ortholog is 100%.
Bootstrap support for CQW49_RS10070 as seed ortholog is 100%.
```

---

### Group of orthologs #1040. Best score 245 bits Score difference with first non-orthologous sequence - AM1\_locus\_tags.txt:245 OB3b\_locus\_tags.txt:245

```
META1_0688          	100.00%		CQW49_RS08555       	100.00%
Bootstrap support for META1_0688 as seed ortholog is 100%.
Bootstrap support for CQW49_RS08555 as seed ortholog is 100%.
```

---

### Group of orthologs #1041. Best score 245 bits Score difference with first non-orthologous sequence - AM1\_locus\_tags.txt:245 OB3b\_locus\_tags.txt:245

```
META1_2097          	100.00%		CQW49_RS03390       	100.00%
Bootstrap support for META1_2097 as seed ortholog is 100%.
Bootstrap support for CQW49_RS03390 as seed ortholog is 100%.
```

---

### Group of orthologs #1042. Best score 245 bits Score difference with first non-orthologous sequence - AM1\_locus\_tags.txt:245 OB3b\_locus\_tags.txt:245

```
META1_2515          	100.00%		CQW49_RS07610       	100.00%
Bootstrap support for META1_2515 as seed ortholog is 100%.
Bootstrap support for CQW49_RS07610 as seed ortholog is 100%.
```

---

### Group of orthologs #1043. Best score 245 bits Score difference with first non-orthologous sequence - AM1\_locus\_tags.txt:245 OB3b\_locus\_tags.txt:245

```
META1_1452          	100.00%		CQW49_RS12855       	100.00%
Bootstrap support for META1_1452 as seed ortholog is 100%.
Bootstrap support for CQW49_RS12855 as seed ortholog is 100%.
```

---

### Group of orthologs #1044. Best score 245 bits Score difference with first non-orthologous sequence - AM1\_locus\_tags.txt:75 OB3b\_locus\_tags.txt:85

```
META1_0735          	100.00%		CQW49_RS21645       	100.00%
Bootstrap support for META1_0735 as seed ortholog is 98%.
Bootstrap support for CQW49_RS21645 as seed ortholog is 99%.
```

---

### Group of orthologs #1045. Best score 245 bits Score difference with first non-orthologous sequence - AM1\_locus\_tags.txt:120 OB3b\_locus\_tags.txt:154

```
META1_3604          	100.00%		CQW49_RS21425       	100.00%
Bootstrap support for META1_3604 as seed ortholog is 99%.
Bootstrap support for CQW49_RS21425 as seed ortholog is 100%.
```

---

### Group of orthologs #1046. Best score 244 bits Score difference with first non-orthologous sequence - AM1\_locus\_tags.txt:124 OB3b\_locus\_tags.txt:244

```
META1_3912          	100.00%		CQW49_RS05035       	100.00%
META1_2638          	10.99%		
Bootstrap support for META1_3912 as seed ortholog is 100%.
Bootstrap support for CQW49_RS05035 as seed ortholog is 100%.
```

---

### Group of orthologs #1047. Best score 244 bits Score difference with first non-orthologous sequence - AM1\_locus\_tags.txt:244 OB3b\_locus\_tags.txt:244

```
META1_4630          	100.00%		CQW49_RS20330       	100.00%
Bootstrap support for META1_4630 as seed ortholog is 100%.
Bootstrap support for CQW49_RS20330 as seed ortholog is 100%.
```

---

### Group of orthologs #1048. Best score 243 bits Score difference with first non-orthologous sequence - AM1\_locus\_tags.txt:243 OB3b\_locus\_tags.txt:243

```
META1_1998          	100.00%		CQW49_RS12475       	100.00%
Bootstrap support for META1_1998 as seed ortholog is 100%.
Bootstrap support for CQW49_RS12475 as seed ortholog is 100%.
```

---

### Group of orthologs #1049. Best score 243 bits Score difference with first non-orthologous sequence - AM1\_locus\_tags.txt:185 OB3b\_locus\_tags.txt:243

```
META1_4586          	100.00%		CQW49_RS04895       	100.00%
Bootstrap support for META1_4586 as seed ortholog is 100%.
Bootstrap support for CQW49_RS04895 as seed ortholog is 100%.
```

---

### Group of orthologs #1050. Best score 243 bits Score difference with first non-orthologous sequence - AM1\_locus\_tags.txt:82 OB3b\_locus\_tags.txt:102

```
META1_4201          	100.00%		CQW49_RS13850       	100.00%
Bootstrap support for META1_4201 as seed ortholog is 96%.
Bootstrap support for CQW49_RS13850 as seed ortholog is 98%.
```

---

### Group of orthologs #1051. Best score 243 bits Score difference with first non-orthologous sequence - AM1\_locus\_tags.txt:57 OB3b\_locus\_tags.txt:128

```
META1_4938          	100.00%		CQW49_RS11555       	100.00%
Bootstrap support for META1_4938 as seed ortholog is 97%.
Bootstrap support for CQW49_RS11555 as seed ortholog is 99%.
```

---

### Group of orthologs #1052. Best score 243 bits Score difference with first non-orthologous sequence - AM1\_locus\_tags.txt:50 OB3b\_locus\_tags.txt:243

```
META1_5119          	100.00%		CQW49_RS20255       	100.00%
Bootstrap support for META1_5119 as seed ortholog is 97%.
Bootstrap support for CQW49_RS20255 as seed ortholog is 100%.
```

---

### Group of orthologs #1053. Best score 242 bits Score difference with first non-orthologous sequence - AM1\_locus\_tags.txt:242 OB3b\_locus\_tags.txt:242

```
META1_1308          	100.00%		CQW49_RS04020       	100.00%
Bootstrap support for META1_1308 as seed ortholog is 100%.
Bootstrap support for CQW49_RS04020 as seed ortholog is 100%.
```

---

### Group of orthologs #1054. Best score 242 bits Score difference with first non-orthologous sequence - AM1\_locus\_tags.txt:242 OB3b\_locus\_tags.txt:242

```
META1_0418          	100.00%		CQW49_RS15815       	100.00%
Bootstrap support for META1_0418 as seed ortholog is 100%.
Bootstrap support for CQW49_RS15815 as seed ortholog is 100%.
```

---

### Group of orthologs #1055. Best score 242 bits Score difference with first non-orthologous sequence - AM1\_locus\_tags.txt:173 OB3b\_locus\_tags.txt:171

```
META1_0501          	100.00%		CQW49_RS19800       	100.00%
Bootstrap support for META1_0501 as seed ortholog is 100%.
Bootstrap support for CQW49_RS19800 as seed ortholog is 100%.
```

---

### Group of orthologs #1056. Best score 242 bits Score difference with first non-orthologous sequence - AM1\_locus\_tags.txt:242 OB3b\_locus\_tags.txt:156

```
META1_4210          	100.00%		CQW49_RS10760       	100.00%
Bootstrap support for META1_4210 as seed ortholog is 100%.
Bootstrap support for CQW49_RS10760 as seed ortholog is 100%.
```

---

### Group of orthologs #1057. Best score 242 bits Score difference with first non-orthologous sequence - AM1\_locus\_tags.txt:54 OB3b\_locus\_tags.txt:242

```
META1_4568          	100.00%		CQW49_RS09115       	100.00%
Bootstrap support for META1_4568 as seed ortholog is 93%.
Bootstrap support for CQW49_RS09115 as seed ortholog is 100%.
```

---

### Group of orthologs #1058. Best score 241 bits Score difference with first non-orthologous sequence - AM1\_locus\_tags.txt:241 OB3b\_locus\_tags.txt:241

```
META1_1295          	100.00%		CQW49_RS11330       	100.00%
Bootstrap support for META1_1295 as seed ortholog is 100%.
Bootstrap support for CQW49_RS11330 as seed ortholog is 100%.
```

---

### Group of orthologs #1059. Best score 241 bits Score difference with first non-orthologous sequence - AM1\_locus\_tags.txt:147 OB3b\_locus\_tags.txt:241

```
META1_2171          	100.00%		CQW49_RS10415       	100.00%
Bootstrap support for META1_2171 as seed ortholog is 100%.
Bootstrap support for CQW49_RS10415 as seed ortholog is 100%.
```

---

### Group of orthologs #1060. Best score 241 bits Score difference with first non-orthologous sequence - AM1\_locus\_tags.txt:241 OB3b\_locus\_tags.txt:241

```
META1_1764          	100.00%		CQW49_RS13525       	100.00%
Bootstrap support for META1_1764 as seed ortholog is 100%.
Bootstrap support for CQW49_RS13525 as seed ortholog is 100%.
```

---

### Group of orthologs #1061. Best score 240 bits Score difference with first non-orthologous sequence - AM1\_locus\_tags.txt:240 OB3b\_locus\_tags.txt:240

```
META1_2716          	100.00%		CQW49_RS17560       	100.00%
Bootstrap support for META1_2716 as seed ortholog is 100%.
Bootstrap support for CQW49_RS17560 as seed ortholog is 100%.
```

---

### Group of orthologs #1062. Best score 240 bits Score difference with first non-orthologous sequence - AM1\_locus\_tags.txt:240 OB3b\_locus\_tags.txt:240

```
META1_2739          	100.00%		CQW49_RS20090       	100.00%
Bootstrap support for META1_2739 as seed ortholog is 100%.
Bootstrap support for CQW49_RS20090 as seed ortholog is 100%.
```

---

### Group of orthologs #1063. Best score 240 bits Score difference with first non-orthologous sequence - AM1\_locus\_tags.txt:15 OB3b\_locus\_tags.txt:240

```
META2_0024          	100.00%		CQW49_RS13450       	100.00%
Bootstrap support for META2_0024 as seed ortholog is 75%.
Bootstrap support for CQW49_RS13450 as seed ortholog is 100%.
```

---

### Group of orthologs #1064. Best score 239 bits Score difference with first non-orthologous sequence - AM1\_locus\_tags.txt:48 OB3b\_locus\_tags.txt:239

```
META1_2558          	100.00%		CQW49_RS09175       	100.00%
Bootstrap support for META1_2558 as seed ortholog is 98%.
Bootstrap support for CQW49_RS09175 as seed ortholog is 100%.
```

---

### Group of orthologs #1065. Best score 239 bits Score difference with first non-orthologous sequence - AM1\_locus\_tags.txt:239 OB3b\_locus\_tags.txt:239

```
META1_2169          	100.00%		CQW49_RS12575       	100.00%
Bootstrap support for META1_2169 as seed ortholog is 100%.
Bootstrap support for CQW49_RS12575 as seed ortholog is 100%.
```

---

### Group of orthologs #1066. Best score 239 bits Score difference with first non-orthologous sequence - AM1\_locus\_tags.txt:239 OB3b\_locus\_tags.txt:239

```
META1_5171          	100.00%		CQW49_RS04190       	100.00%
Bootstrap support for META1_5171 as seed ortholog is 100%.
Bootstrap support for CQW49_RS04190 as seed ortholog is 100%.
```

---

### Group of orthologs #1067. Best score 239 bits Score difference with first non-orthologous sequence - AM1\_locus\_tags.txt:239 OB3b\_locus\_tags.txt:239

```
META1_3317          	100.00%		CQW49_RS18870       	100.00%
Bootstrap support for META1_3317 as seed ortholog is 100%.
Bootstrap support for CQW49_RS18870 as seed ortholog is 100%.
```

---

### Group of orthologs #1068. Best score 239 bits Score difference with first non-orthologous sequence - AM1\_locus\_tags.txt:172 OB3b\_locus\_tags.txt:239

```
META1_3605          	100.00%		CQW49_RS21430       	100.00%
Bootstrap support for META1_3605 as seed ortholog is 100%.
Bootstrap support for CQW49_RS21430 as seed ortholog is 100%.
```

---

### Group of orthologs #1069. Best score 238 bits Score difference with first non-orthologous sequence - AM1\_locus\_tags.txt:238 OB3b\_locus\_tags.txt:238

```
META1_0846          	100.00%		CQW49_RS05310       	100.00%
Bootstrap support for META1_0846 as seed ortholog is 100%.
Bootstrap support for CQW49_RS05310 as seed ortholog is 100%.
```

---

### Group of orthologs #1070. Best score 238 bits Score difference with first non-orthologous sequence - AM1\_locus\_tags.txt:238 OB3b\_locus\_tags.txt:238

```
META1_0681          	100.00%		CQW49_RS08885       	100.00%
Bootstrap support for META1_0681 as seed ortholog is 100%.
Bootstrap support for CQW49_RS08885 as seed ortholog is 100%.
```

---

### Group of orthologs #1071. Best score 238 bits Score difference with first non-orthologous sequence - AM1\_locus\_tags.txt:238 OB3b\_locus\_tags.txt:238

```
META1_0428          	100.00%		CQW49_RS14295       	100.00%
Bootstrap support for META1_0428 as seed ortholog is 100%.
Bootstrap support for CQW49_RS14295 as seed ortholog is 100%.
```

---

### Group of orthologs #1072. Best score 237 bits Score difference with first non-orthologous sequence - AM1\_locus\_tags.txt:237 OB3b\_locus\_tags.txt:237

```
META1_0861          	100.00%		CQW49_RS10610       	100.00%
Bootstrap support for META1_0861 as seed ortholog is 100%.
Bootstrap support for CQW49_RS10610 as seed ortholog is 100%.
```

---

### Group of orthologs #1073. Best score 237 bits Score difference with first non-orthologous sequence - AM1\_locus\_tags.txt:145 OB3b\_locus\_tags.txt:237

```
META1_0663          	100.00%		CQW49_RS17650       	100.00%
Bootstrap support for META1_0663 as seed ortholog is 100%.
Bootstrap support for CQW49_RS17650 as seed ortholog is 100%.
```

---

### Group of orthologs #1074. Best score 237 bits Score difference with first non-orthologous sequence - AM1\_locus\_tags.txt:237 OB3b\_locus\_tags.txt:237

```
META1_1999          	100.00%		CQW49_RS12470       	100.00%
Bootstrap support for META1_1999 as seed ortholog is 100%.
Bootstrap support for CQW49_RS12470 as seed ortholog is 100%.
```

---

### Group of orthologs #1075. Best score 237 bits Score difference with first non-orthologous sequence - AM1\_locus\_tags.txt:132 OB3b\_locus\_tags.txt:237

```
META1_4687          	100.00%		CQW49_RS03305       	100.00%
Bootstrap support for META1_4687 as seed ortholog is 100%.
Bootstrap support for CQW49_RS03305 as seed ortholog is 100%.
```

---

### Group of orthologs #1076. Best score 237 bits Score difference with first non-orthologous sequence - AM1\_locus\_tags.txt:237 OB3b\_locus\_tags.txt:237

```
META1_2476          	100.00%		CQW49_RS21080       	100.00%
Bootstrap support for META1_2476 as seed ortholog is 100%.
Bootstrap support for CQW49_RS21080 as seed ortholog is 100%.
```

---

### Group of orthologs #1077. Best score 237 bits Score difference with first non-orthologous sequence - AM1\_locus\_tags.txt:196 OB3b\_locus\_tags.txt:237

```
META1_4873          	100.00%		CQW49_RS14350       	100.00%
Bootstrap support for META1_4873 as seed ortholog is 100%.
Bootstrap support for CQW49_RS14350 as seed ortholog is 100%.
```

---

### Group of orthologs #1078. Best score 236 bits Score difference with first non-orthologous sequence - AM1\_locus\_tags.txt:169 OB3b\_locus\_tags.txt:113

```
META1_2996          	100.00%		CQW49_RS09465       	100.00%
Bootstrap support for META1_2996 as seed ortholog is 99%.
Bootstrap support for CQW49_RS09465 as seed ortholog is 99%.
```

---

### Group of orthologs #1079. Best score 236 bits Score difference with first non-orthologous sequence - AM1\_locus\_tags.txt:236 OB3b\_locus\_tags.txt:236

```
META1_2983          	100.00%		CQW49_RS16320       	100.00%
Bootstrap support for META1_2983 as seed ortholog is 100%.
Bootstrap support for CQW49_RS16320 as seed ortholog is 100%.
```

---

### Group of orthologs #1080. Best score 235 bits Score difference with first non-orthologous sequence - AM1\_locus\_tags.txt:235 OB3b\_locus\_tags.txt:235

```
META1_0690          	100.00%		CQW49_RS02760       	100.00%
Bootstrap support for META1_0690 as seed ortholog is 100%.
Bootstrap support for CQW49_RS02760 as seed ortholog is 100%.
```

---

### Group of orthologs #1081. Best score 235 bits Score difference with first non-orthologous sequence - AM1\_locus\_tags.txt:235 OB3b\_locus\_tags.txt:235

```
META1_0170          	100.00%		CQW49_RS05360       	100.00%
Bootstrap support for META1_0170 as seed ortholog is 100%.
Bootstrap support for CQW49_RS05360 as seed ortholog is 100%.
```

---

### Group of orthologs #1082. Best score 235 bits Score difference with first non-orthologous sequence - AM1\_locus\_tags.txt:235 OB3b\_locus\_tags.txt:235

```
META1_1433          	100.00%		CQW49_RS06580       	100.00%
Bootstrap support for META1_1433 as seed ortholog is 100%.
Bootstrap support for CQW49_RS06580 as seed ortholog is 100%.
```

---

### Group of orthologs #1083. Best score 235 bits Score difference with first non-orthologous sequence - AM1\_locus\_tags.txt:189 OB3b\_locus\_tags.txt:235

```
META1_0859          	100.00%		CQW49_RS10615       	100.00%
Bootstrap support for META1_0859 as seed ortholog is 100%.
Bootstrap support for CQW49_RS10615 as seed ortholog is 100%.
```

---

### Group of orthologs #1084. Best score 235 bits Score difference with first non-orthologous sequence - AM1\_locus\_tags.txt:111 OB3b\_locus\_tags.txt:94

```
META1_1425          	100.00%		CQW49_RS08590       	100.00%
Bootstrap support for META1_1425 as seed ortholog is 99%.
Bootstrap support for CQW49_RS08590 as seed ortholog is 99%.
```

---

### Group of orthologs #1085. Best score 233 bits Score difference with first non-orthologous sequence - AM1\_locus\_tags.txt:233 OB3b\_locus\_tags.txt:233

```
META1_1290          	100.00%		CQW49_RS01835       	100.00%
Bootstrap support for META1_1290 as seed ortholog is 100%.
Bootstrap support for CQW49_RS01835 as seed ortholog is 100%.
```

---

### Group of orthologs #1086. Best score 233 bits Score difference with first non-orthologous sequence - AM1\_locus\_tags.txt:233 OB3b\_locus\_tags.txt:233

```
META1_1420          	100.00%		CQW49_RS08570       	100.00%
Bootstrap support for META1_1420 as seed ortholog is 100%.
Bootstrap support for CQW49_RS08570 as seed ortholog is 100%.
```

---

### Group of orthologs #1087. Best score 233 bits Score difference with first non-orthologous sequence - AM1\_locus\_tags.txt:233 OB3b\_locus\_tags.txt:233

```
META1_4429          	100.00%		CQW49_RS16185       	100.00%
Bootstrap support for META1_4429 as seed ortholog is 100%.
Bootstrap support for CQW49_RS16185 as seed ortholog is 100%.
```

---

### Group of orthologs #1088. Best score 232 bits Score difference with first non-orthologous sequence - AM1\_locus\_tags.txt:232 OB3b\_locus\_tags.txt:232

```
META1_3246          	100.00%		CQW49_RS01460       	100.00%
Bootstrap support for META1_3246 as seed ortholog is 100%.
Bootstrap support for CQW49_RS01460 as seed ortholog is 100%.
```

---

### Group of orthologs #1089. Best score 232 bits Score difference with first non-orthologous sequence - AM1\_locus\_tags.txt:232 OB3b\_locus\_tags.txt:232

```
META1_4275          	100.00%		CQW49_RS10675       	100.00%
Bootstrap support for META1_4275 as seed ortholog is 100%.
Bootstrap support for CQW49_RS10675 as seed ortholog is 100%.
```

---

### Group of orthologs #1090. Best score 231 bits Score difference with first non-orthologous sequence - AM1\_locus\_tags.txt:109 OB3b\_locus\_tags.txt:231

```
META1_1645          	100.00%		CQW49_RS06560       	100.00%
Bootstrap support for META1_1645 as seed ortholog is 99%.
Bootstrap support for CQW49_RS06560 as seed ortholog is 100%.
```

---

### Group of orthologs #1091. Best score 231 bits Score difference with first non-orthologous sequence - AM1\_locus\_tags.txt:59 OB3b\_locus\_tags.txt:231

```
META1_1378          	100.00%		CQW49_RS12835       	100.00%
Bootstrap support for META1_1378 as seed ortholog is 97%.
Bootstrap support for CQW49_RS12835 as seed ortholog is 100%.
```

---

### Group of orthologs #1092. Best score 230 bits Score difference with first non-orthologous sequence - AM1\_locus\_tags.txt:230 OB3b\_locus\_tags.txt:230

```
META1_2182          	100.00%		CQW49_RS10470       	100.00%
Bootstrap support for META1_2182 as seed ortholog is 100%.
Bootstrap support for CQW49_RS10470 as seed ortholog is 100%.
```

---

### Group of orthologs #1093. Best score 230 bits Score difference with first non-orthologous sequence - AM1\_locus\_tags.txt:230 OB3b\_locus\_tags.txt:230

```
META1_3410          	100.00%		CQW49_RS05915       	100.00%
Bootstrap support for META1_3410 as seed ortholog is 100%.
Bootstrap support for CQW49_RS05915 as seed ortholog is 100%.
```

---

### Group of orthologs #1094. Best score 230 bits Score difference with first non-orthologous sequence - AM1\_locus\_tags.txt:230 OB3b\_locus\_tags.txt:230

```
META1_4458          	100.00%		CQW49_RS04180       	100.00%
Bootstrap support for META1_4458 as seed ortholog is 100%.
Bootstrap support for CQW49_RS04180 as seed ortholog is 100%.
```

---

### Group of orthologs #1095. Best score 230 bits Score difference with first non-orthologous sequence - AM1\_locus\_tags.txt:230 OB3b\_locus\_tags.txt:230

```
META1_3073          	100.00%		CQW49_RS15725       	100.00%
Bootstrap support for META1_3073 as seed ortholog is 100%.
Bootstrap support for CQW49_RS15725 as seed ortholog is 100%.
```

---

### Group of orthologs #1096. Best score 230 bits Score difference with first non-orthologous sequence - AM1\_locus\_tags.txt:230 OB3b\_locus\_tags.txt:134

```
META1_4703          	100.00%		CQW49_RS10270       	100.00%
Bootstrap support for META1_4703 as seed ortholog is 100%.
Bootstrap support for CQW49_RS10270 as seed ortholog is 100%.
```

---

### Group of orthologs #1097. Best score 230 bits Score difference with first non-orthologous sequence - AM1\_locus\_tags.txt:230 OB3b\_locus\_tags.txt:119

```
META1_3437          	100.00%		CQW49_RS16510       	100.00%
Bootstrap support for META1_3437 as seed ortholog is 100%.
Bootstrap support for CQW49_RS16510 as seed ortholog is 100%.
```

---

### Group of orthologs #1098. Best score 230 bits Score difference with first non-orthologous sequence - AM1\_locus\_tags.txt:230 OB3b\_locus\_tags.txt:230

```
META1_4536          	100.00%		CQW49_RS14470       	100.00%
Bootstrap support for META1_4536 as seed ortholog is 100%.
Bootstrap support for CQW49_RS14470 as seed ortholog is 100%.
```

---

### Group of orthologs #1099. Best score 230 bits Score difference with first non-orthologous sequence - AM1\_locus\_tags.txt:83 OB3b\_locus\_tags.txt:133

```
META1_3603          	100.00%		CQW49_RS21420       	100.00%
Bootstrap support for META1_3603 as seed ortholog is 99%.
Bootstrap support for CQW49_RS21420 as seed ortholog is 100%.
```

---

### Group of orthologs #1100. Best score 229 bits Score difference with first non-orthologous sequence - AM1\_locus\_tags.txt:229 OB3b\_locus\_tags.txt:229

```
META1_0157          	100.00%		CQW49_RS02105       	100.00%
Bootstrap support for META1_0157 as seed ortholog is 100%.
Bootstrap support for CQW49_RS02105 as seed ortholog is 100%.
```

---

### Group of orthologs #1101. Best score 229 bits Score difference with first non-orthologous sequence - AM1\_locus\_tags.txt:229 OB3b\_locus\_tags.txt:229

```
META1_1685          	100.00%		CQW49_RS01985       	100.00%
Bootstrap support for META1_1685 as seed ortholog is 100%.
Bootstrap support for CQW49_RS01985 as seed ortholog is 100%.
```

---

### Group of orthologs #1102. Best score 229 bits Score difference with first non-orthologous sequence - AM1\_locus\_tags.txt:229 OB3b\_locus\_tags.txt:229

```
META1_2177          	100.00%		CQW49_RS10445       	100.00%
Bootstrap support for META1_2177 as seed ortholog is 100%.
Bootstrap support for CQW49_RS10445 as seed ortholog is 100%.
```

---

### Group of orthologs #1103. Best score 228 bits Score difference with first non-orthologous sequence - AM1\_locus\_tags.txt:80 OB3b\_locus\_tags.txt:228

```
META1_0041          	100.00%		CQW49_RS00385       	100.00%
Bootstrap support for META1_0041 as seed ortholog is 98%.
Bootstrap support for CQW49_RS00385 as seed ortholog is 100%.
```

---

### Group of orthologs #1104. Best score 228 bits Score difference with first non-orthologous sequence - AM1\_locus\_tags.txt:228 OB3b\_locus\_tags.txt:228

```
META1_0050          	100.00%		CQW49_RS17175       	100.00%
Bootstrap support for META1_0050 as seed ortholog is 100%.
Bootstrap support for CQW49_RS17175 as seed ortholog is 100%.
```

---

### Group of orthologs #1105. Best score 228 bits Score difference with first non-orthologous sequence - AM1\_locus\_tags.txt:48 OB3b\_locus\_tags.txt:164

```
META1_0755          	100.00%		CQW49_RS14890       	100.00%
Bootstrap support for META1_0755 as seed ortholog is 90%.
Bootstrap support for CQW49_RS14890 as seed ortholog is 100%.
```

---

### Group of orthologs #1106. Best score 228 bits Score difference with first non-orthologous sequence - AM1\_locus\_tags.txt:228 OB3b\_locus\_tags.txt:228

```
META1_2319          	100.00%		CQW49_RS12615       	100.00%
Bootstrap support for META1_2319 as seed ortholog is 100%.
Bootstrap support for CQW49_RS12615 as seed ortholog is 100%.
```

---

### Group of orthologs #1107. Best score 228 bits Score difference with first non-orthologous sequence - AM1\_locus\_tags.txt:228 OB3b\_locus\_tags.txt:228

```
META1_1337          	100.00%		CQW49_RS17910       	100.00%
Bootstrap support for META1_1337 as seed ortholog is 100%.
Bootstrap support for CQW49_RS17910 as seed ortholog is 100%.
```

---

### Group of orthologs #1108. Best score 228 bits Score difference with first non-orthologous sequence - AM1\_locus\_tags.txt:131 OB3b\_locus\_tags.txt:80

```
META1_4216          	100.00%		CQW49_RS09680       	100.00%
Bootstrap support for META1_4216 as seed ortholog is 99%.
Bootstrap support for CQW49_RS09680 as seed ortholog is 99%.
```

---

### Group of orthologs #1109. Best score 228 bits Score difference with first non-orthologous sequence - AM1\_locus\_tags.txt:228 OB3b\_locus\_tags.txt:228

```
META1_4385          	100.00%		CQW49_RS11365       	100.00%
Bootstrap support for META1_4385 as seed ortholog is 100%.
Bootstrap support for CQW49_RS11365 as seed ortholog is 100%.
```

---

### Group of orthologs #1110. Best score 227 bits Score difference with first non-orthologous sequence - AM1\_locus\_tags.txt:227 OB3b\_locus\_tags.txt:152

```
META1_1918          	100.00%		CQW49_RS07475       	100.00%
META1_3758          	58.97%		
Bootstrap support for META1_1918 as seed ortholog is 100%.
Bootstrap support for CQW49_RS07475 as seed ortholog is 100%.
```

---

### Group of orthologs #1111. Best score 227 bits Score difference with first non-orthologous sequence - AM1\_locus\_tags.txt:71 OB3b\_locus\_tags.txt:227

```
META1_4582          	100.00%		CQW49_RS01325       	100.00%
Bootstrap support for META1_4582 as seed ortholog is 99%.
Bootstrap support for CQW49_RS01325 as seed ortholog is 100%.
```

---

### Group of orthologs #1112. Best score 227 bits Score difference with first non-orthologous sequence - AM1\_locus\_tags.txt:227 OB3b\_locus\_tags.txt:227

```
META1_1692          	100.00%		CQW49_RS16050       	100.00%
Bootstrap support for META1_1692 as seed ortholog is 100%.
Bootstrap support for CQW49_RS16050 as seed ortholog is 100%.
```

---

### Group of orthologs #1113. Best score 227 bits Score difference with first non-orthologous sequence - AM1\_locus\_tags.txt:227 OB3b\_locus\_tags.txt:227

```
META1_2740          	100.00%		CQW49_RS20095       	100.00%
Bootstrap support for META1_2740 as seed ortholog is 100%.
Bootstrap support for CQW49_RS20095 as seed ortholog is 100%.
```

---

### Group of orthologs #1114. Best score 227 bits Score difference with first non-orthologous sequence - AM1\_locus\_tags.txt:227 OB3b\_locus\_tags.txt:67

```
META1_4712          	100.00%		CQW49_RS11900       	100.00%
Bootstrap support for META1_4712 as seed ortholog is 100%.
Bootstrap support for CQW49_RS11900 as seed ortholog is 97%.
```

---

### Group of orthologs #1115. Best score 226 bits Score difference with first non-orthologous sequence - AM1\_locus\_tags.txt:226 OB3b\_locus\_tags.txt:226

```
META1_1936          	100.00%		CQW49_RS21000       	100.00%
Bootstrap support for META1_1936 as seed ortholog is 100%.
Bootstrap support for CQW49_RS21000 as seed ortholog is 100%.
```

---

### Group of orthologs #1116. Best score 225 bits Score difference with first non-orthologous sequence - AM1\_locus\_tags.txt:225 OB3b\_locus\_tags.txt:225

```
META1_1765          	100.00%		CQW49_RS13520       	100.00%
Bootstrap support for META1_1765 as seed ortholog is 100%.
Bootstrap support for CQW49_RS13520 as seed ortholog is 100%.
```

---

### Group of orthologs #1117. Best score 225 bits Score difference with first non-orthologous sequence - AM1\_locus\_tags.txt:225 OB3b\_locus\_tags.txt:225

```
META1_5021          	100.00%		CQW49_RS01495       	100.00%
Bootstrap support for META1_5021 as seed ortholog is 100%.
Bootstrap support for CQW49_RS01495 as seed ortholog is 100%.
```

---

### Group of orthologs #1118. Best score 225 bits Score difference with first non-orthologous sequence - AM1\_locus\_tags.txt:28 OB3b\_locus\_tags.txt:103

```
META1_4447          	100.00%		CQW49_RS20610       	100.00%
Bootstrap support for META1_4447 as seed ortholog is 20%.
Alternative seed ortholog is META1_2142 (28 bits away from this cluster)
Bootstrap support for CQW49_RS20610 as seed ortholog is 99%.
```

---

### Group of orthologs #1119. Best score 224 bits Score difference with first non-orthologous sequence - AM1\_locus\_tags.txt:224 OB3b\_locus\_tags.txt:224

```
META1_1705          	100.00%		CQW49_RS14435       	100.00%
Bootstrap support for META1_1705 as seed ortholog is 100%.
Bootstrap support for CQW49_RS14435 as seed ortholog is 100%.
```

---

### Group of orthologs #1120. Best score 224 bits Score difference with first non-orthologous sequence - AM1\_locus\_tags.txt:95 OB3b\_locus\_tags.txt:224

```
META1_1530          	100.00%		CQW49_RS19190       	100.00%
Bootstrap support for META1_1530 as seed ortholog is 99%.
Bootstrap support for CQW49_RS19190 as seed ortholog is 100%.
```

---

### Group of orthologs #1121. Best score 224 bits Score difference with first non-orthologous sequence - AM1\_locus\_tags.txt:224 OB3b\_locus\_tags.txt:224

```
META1_4403          	100.00%		CQW49_RS17310       	100.00%
Bootstrap support for META1_4403 as seed ortholog is 100%.
Bootstrap support for CQW49_RS17310 as seed ortholog is 100%.
```

---

### Group of orthologs #1122. Best score 224 bits Score difference with first non-orthologous sequence - AM1\_locus\_tags.txt:44 OB3b\_locus\_tags.txt:45

```
META1_4818          	100.00%		CQW49_RS17690       	100.00%
Bootstrap support for META1_4818 as seed ortholog is 95%.
Bootstrap support for CQW49_RS17690 as seed ortholog is 94%.
```

---

### Group of orthologs #1123. Best score 223 bits Score difference with first non-orthologous sequence - AM1\_locus\_tags.txt:38 OB3b\_locus\_tags.txt:124

```
META1_3042          	100.00%		CQW49_RS09760       	100.00%
                    	       		CQW49_RS19690       	7.32%
Bootstrap support for META1_3042 as seed ortholog is 88%.
Bootstrap support for CQW49_RS09760 as seed ortholog is 99%.
```

---

### Group of orthologs #1124. Best score 223 bits Score difference with first non-orthologous sequence - AM1\_locus\_tags.txt:223 OB3b\_locus\_tags.txt:223

```
META1_1945          	100.00%		CQW49_RS04505       	100.00%
Bootstrap support for META1_1945 as seed ortholog is 100%.
Bootstrap support for CQW49_RS04505 as seed ortholog is 100%.
```

---

### Group of orthologs #1125. Best score 223 bits Score difference with first non-orthologous sequence - AM1\_locus\_tags.txt:223 OB3b\_locus\_tags.txt:223

```
META1_3209          	100.00%		CQW49_RS16275       	100.00%
Bootstrap support for META1_3209 as seed ortholog is 100%.
Bootstrap support for CQW49_RS16275 as seed ortholog is 100%.
```

---

### Group of orthologs #1126. Best score 223 bits Score difference with first non-orthologous sequence - AM1\_locus\_tags.txt:152 OB3b\_locus\_tags.txt:223

```
META1_3611          	100.00%		CQW49_RS16240       	100.00%
Bootstrap support for META1_3611 as seed ortholog is 100%.
Bootstrap support for CQW49_RS16240 as seed ortholog is 100%.
```

---

### Group of orthologs #1127. Best score 223 bits Score difference with first non-orthologous sequence - AM1\_locus\_tags.txt:16 OB3b\_locus\_tags.txt:54

```
META1_3533          	100.00%		CQW49_RS21275       	100.00%
Bootstrap support for META1_3533 as seed ortholog is 72%.
Alternative seed ortholog is META1_0926 (16 bits away from this cluster)
[truncated: 297,636 more chars]
