# Supplementary material for: Genome-Scale Metabolic Model Reconstruction and in Silico Investigations of Methane Metabolism in Methylosinus trichosporium OB3b
Source: Microorganisms. 2020 Mar 20;8(3):437. doi: 10.3390/microorganisms8030437 (PMC7144005; doi:10.3390/microorganisms8030437)
Supplement: Supplementary file 1 [file microorganisms-08-00437-s001.zip › supplementary_materials/gem_reconstruction_supplementary_materials/Inparanoid_outputs/OB3b_refseq_genome_ids_vs_OB3b_genbank_genome_ids/orthologs.OB3b_Refseq_for_inparanoid.fasta-OB3b_Genbank_for_inparanoid.fasta.html]

```
###################################
4095 groups of orthologs
4218 in-paralogs from OB3b_Refseq_for_inparanoid.fasta
4147 in-paralogs from OB3b_Genbank_for_inparanoid.fasta
Grey zone 0 bits
Score cutoff 40 bits
In-paralogs with confidence less than 0.05 not shown
Sequence overlap cutoff 0.5
Group merging cutoff 0.5
Scoring matrix BLOSUM62
###################################
```

---

### Group of orthologs #1. Best score 8639 bits Score difference with first non-orthologous sequence - OB3b\_Refseq\_for\_inparanoid.fasta:8639 OB3b\_Genbank\_for\_inparanoid.fasta:8639

```
CQW49_RS17720       	100.00%		CQW49_17665         	100.00%
Bootstrap support for CQW49_RS17720 as seed ortholog is 100%.
Bootstrap support for CQW49_17665 as seed ortholog is 100%.
```

---

### Group of orthologs #2. Best score 7885 bits Score difference with first non-orthologous sequence - OB3b\_Refseq\_for\_inparanoid.fasta:7885 OB3b\_Genbank\_for\_inparanoid.fasta:5889

```
CQW49_RS02345       	100.00%		CQW49_02340         	100.00%
Bootstrap support for CQW49_RS02345 as seed ortholog is 100%.
Bootstrap support for CQW49_02340 as seed ortholog is 100%.
```

---

### Group of orthologs #3. Best score 7159 bits Score difference with first non-orthologous sequence - OB3b\_Refseq\_for\_inparanoid.fasta:7159 OB3b\_Genbank\_for\_inparanoid.fasta:5955

```
CQW49_RS13625       	100.00%		CQW49_13590         	100.00%
Bootstrap support for CQW49_RS13625 as seed ortholog is 100%.
Bootstrap support for CQW49_13590 as seed ortholog is 100%.
```

---

### Group of orthologs #4. Best score 6064 bits Score difference with first non-orthologous sequence - OB3b\_Refseq\_for\_inparanoid.fasta:6064 OB3b\_Genbank\_for\_inparanoid.fasta:6064

```
CQW49_RS00015       	100.00%		CQW49_00015         	100.00%
Bootstrap support for CQW49_RS00015 as seed ortholog is 100%.
Bootstrap support for CQW49_00015 as seed ortholog is 100%.
```

---

### Group of orthologs #5. Best score 5974 bits Score difference with first non-orthologous sequence - OB3b\_Refseq\_for\_inparanoid.fasta:5974 OB3b\_Genbank\_for\_inparanoid.fasta:4352

```
CQW49_RS09840       	100.00%		CQW49_09820         	100.00%
Bootstrap support for CQW49_RS09840 as seed ortholog is 100%.
Bootstrap support for CQW49_09820 as seed ortholog is 100%.
```

---

### Group of orthologs #6. Best score 4739 bits Score difference with first non-orthologous sequence - OB3b\_Refseq\_for\_inparanoid.fasta:4739 OB3b\_Genbank\_for\_inparanoid.fasta:4739

```
CQW49_RS12625       	100.00%		CQW49_12590         	100.00%
Bootstrap support for CQW49_RS12625 as seed ortholog is 100%.
Bootstrap support for CQW49_12590 as seed ortholog is 100%.
```

---

### Group of orthologs #7. Best score 3877 bits Score difference with first non-orthologous sequence - OB3b\_Refseq\_for\_inparanoid.fasta:3877 OB3b\_Genbank\_for\_inparanoid.fasta:3877

```
CQW49_RS09700       	100.00%		CQW49_09680         	100.00%
Bootstrap support for CQW49_RS09700 as seed ortholog is 100%.
Bootstrap support for CQW49_09680 as seed ortholog is 100%.
```

---

### Group of orthologs #8. Best score 3874 bits Score difference with first non-orthologous sequence - OB3b\_Refseq\_for\_inparanoid.fasta:3874 OB3b\_Genbank\_for\_inparanoid.fasta:3874

```
CQW49_RS12915       	100.00%		CQW49_12880         	100.00%
Bootstrap support for CQW49_RS12915 as seed ortholog is 100%.
Bootstrap support for CQW49_12880 as seed ortholog is 100%.
```

---

### Group of orthologs #9. Best score 3627 bits Score difference with first non-orthologous sequence - OB3b\_Refseq\_for\_inparanoid.fasta:3627 OB3b\_Genbank\_for\_inparanoid.fasta:3627

```
CQW49_RS00895       	100.00%		CQW49_00895         	100.00%
Bootstrap support for CQW49_RS00895 as seed ortholog is 100%.
Bootstrap support for CQW49_00895 as seed ortholog is 100%.
```

---

### Group of orthologs #10. Best score 3287 bits Score difference with first non-orthologous sequence - OB3b\_Refseq\_for\_inparanoid.fasta:3287 OB3b\_Genbank\_for\_inparanoid.fasta:3287

```
CQW49_RS12910       	100.00%		CQW49_12875         	100.00%
Bootstrap support for CQW49_RS12910 as seed ortholog is 100%.
Bootstrap support for CQW49_12875 as seed ortholog is 100%.
```

---

### Group of orthologs #11. Best score 3080 bits Score difference with first non-orthologous sequence - OB3b\_Refseq\_for\_inparanoid.fasta:3080 OB3b\_Genbank\_for\_inparanoid.fasta:3080

```
CQW49_RS02665       	100.00%		CQW49_02655         	100.00%
Bootstrap support for CQW49_RS02665 as seed ortholog is 100%.
Bootstrap support for CQW49_02655 as seed ortholog is 100%.
```

---

### Group of orthologs #12. Best score 2810 bits Score difference with first non-orthologous sequence - OB3b\_Refseq\_for\_inparanoid.fasta:2810 OB3b\_Genbank\_for\_inparanoid.fasta:1099

```
CQW49_RS15250       	100.00%		CQW49_15215         	100.00%
Bootstrap support for CQW49_RS15250 as seed ortholog is 100%.
Bootstrap support for CQW49_15215 as seed ortholog is 100%.
```

---

### Group of orthologs #13. Best score 2809 bits Score difference with first non-orthologous sequence - OB3b\_Refseq\_for\_inparanoid.fasta:2809 OB3b\_Genbank\_for\_inparanoid.fasta:1091

```
CQW49_RS14010       	100.00%		CQW49_13975         	100.00%
Bootstrap support for CQW49_RS14010 as seed ortholog is 100%.
Bootstrap support for CQW49_13975 as seed ortholog is 100%.
```

---

### Group of orthologs #14. Best score 2808 bits Score difference with first non-orthologous sequence - OB3b\_Refseq\_for\_inparanoid.fasta:2808 OB3b\_Genbank\_for\_inparanoid.fasta:2808

```
CQW49_RS17930       	100.00%		CQW49_17875         	100.00%
Bootstrap support for CQW49_RS17930 as seed ortholog is 100%.
Bootstrap support for CQW49_17875 as seed ortholog is 100%.
```

---

### Group of orthologs #15. Best score 2740 bits Score difference with first non-orthologous sequence - OB3b\_Refseq\_for\_inparanoid.fasta:2740 OB3b\_Genbank\_for\_inparanoid.fasta:2740

```
rpoC                	100.00%		CQW49_17280         	100.00%
Bootstrap support for rpoC as seed ortholog is 100%.
Bootstrap support for CQW49_17280 as seed ortholog is 100%.
```

---

### Group of orthologs #16. Best score 2726 bits Score difference with first non-orthologous sequence - OB3b\_Refseq\_for\_inparanoid.fasta:2726 OB3b\_Genbank\_for\_inparanoid.fasta:2726

```
rpoB                	100.00%		CQW49_17275         	100.00%
Bootstrap support for rpoB as seed ortholog is 100%.
Bootstrap support for CQW49_17275 as seed ortholog is 100%.
```

---

### Group of orthologs #17. Best score 2719 bits Score difference with first non-orthologous sequence - OB3b\_Refseq\_for\_inparanoid.fasta:2719 OB3b\_Genbank\_for\_inparanoid.fasta:2719

```
CQW49_RS08505       	100.00%		CQW49_08495         	100.00%
Bootstrap support for CQW49_RS08505 as seed ortholog is 100%.
Bootstrap support for CQW49_08495 as seed ortholog is 100%.
```

---

### Group of orthologs #18. Best score 2716 bits Score difference with first non-orthologous sequence - OB3b\_Refseq\_for\_inparanoid.fasta:2716 OB3b\_Genbank\_for\_inparanoid.fasta:2347

```
CQW49_RS19210       	100.00%		CQW49_19155         	100.00%
Bootstrap support for CQW49_RS19210 as seed ortholog is 100%.
Bootstrap support for CQW49_19155 as seed ortholog is 100%.
```

---

### Group of orthologs #19. Best score 2707 bits Score difference with first non-orthologous sequence - OB3b\_Refseq\_for\_inparanoid.fasta:2707 OB3b\_Genbank\_for\_inparanoid.fasta:2707

```
CQW49_RS21015       	100.00%		CQW49_20940         	100.00%
Bootstrap support for CQW49_RS21015 as seed ortholog is 100%.
Bootstrap support for CQW49_20940 as seed ortholog is 100%.
```

---

### Group of orthologs #20. Best score 2683 bits Score difference with first non-orthologous sequence - OB3b\_Refseq\_for\_inparanoid.fasta:2683 OB3b\_Genbank\_for\_inparanoid.fasta:2683

```
CQW49_RS11690       	100.00%		CQW49_11655         	100.00%
Bootstrap support for CQW49_RS11690 as seed ortholog is 100%.
Bootstrap support for CQW49_11655 as seed ortholog is 100%.
```

---

### Group of orthologs #21. Best score 2666 bits Score difference with first non-orthologous sequence - OB3b\_Refseq\_for\_inparanoid.fasta:2666 OB3b\_Genbank\_for\_inparanoid.fasta:2666

```
CQW49_RS06985       	100.00%		CQW49_06975         	100.00%
Bootstrap support for CQW49_RS06985 as seed ortholog is 100%.
Bootstrap support for CQW49_06975 as seed ortholog is 100%.
```

---

### Group of orthologs #22. Best score 2647 bits Score difference with first non-orthologous sequence - OB3b\_Refseq\_for\_inparanoid.fasta:2647 OB3b\_Genbank\_for\_inparanoid.fasta:2237

```
CQW49_RS01580       	100.00%		CQW49_01575         	100.00%
Bootstrap support for CQW49_RS01580 as seed ortholog is 100%.
Bootstrap support for CQW49_01575 as seed ortholog is 100%.
```

---

### Group of orthologs #23. Best score 2609 bits Score difference with first non-orthologous sequence - OB3b\_Refseq\_for\_inparanoid.fasta:2609 OB3b\_Genbank\_for\_inparanoid.fasta:2609

```
CQW49_RS15520       	100.00%		CQW49_15485         	100.00%
Bootstrap support for CQW49_RS15520 as seed ortholog is 100%.
Bootstrap support for CQW49_15485 as seed ortholog is 100%.
```

---

### Group of orthologs #24. Best score 2557 bits Score difference with first non-orthologous sequence - OB3b\_Refseq\_for\_inparanoid.fasta:2557 OB3b\_Genbank\_for\_inparanoid.fasta:2557

```
CQW49_RS04005       	100.00%		CQW49_03995         	100.00%
Bootstrap support for CQW49_RS04005 as seed ortholog is 100%.
Bootstrap support for CQW49_03995 as seed ortholog is 100%.
```

---

### Group of orthologs #25. Best score 2513 bits Score difference with first non-orthologous sequence - OB3b\_Refseq\_for\_inparanoid.fasta:2513 OB3b\_Genbank\_for\_inparanoid.fasta:2513

```
CQW49_RS05215       	100.00%		CQW49_05210         	100.00%
Bootstrap support for CQW49_RS05215 as seed ortholog is 100%.
Bootstrap support for CQW49_05210 as seed ortholog is 100%.
```

---

### Group of orthologs #26. Best score 2458 bits Score difference with first non-orthologous sequence - OB3b\_Refseq\_for\_inparanoid.fasta:2458 OB3b\_Genbank\_for\_inparanoid.fasta:2458

```
CQW49_RS17905       	100.00%		CQW49_17850         	100.00%
Bootstrap support for CQW49_RS17905 as seed ortholog is 100%.
Bootstrap support for CQW49_17850 as seed ortholog is 100%.
```

---

### Group of orthologs #27. Best score 2440 bits Score difference with first non-orthologous sequence - OB3b\_Refseq\_for\_inparanoid.fasta:2440 OB3b\_Genbank\_for\_inparanoid.fasta:2440

```
CQW49_RS19115       	100.00%		CQW49_19055         	100.00%
Bootstrap support for CQW49_RS19115 as seed ortholog is 100%.
Bootstrap support for CQW49_19055 as seed ortholog is 100%.
```

---

### Group of orthologs #28. Best score 2428 bits Score difference with first non-orthologous sequence - OB3b\_Refseq\_for\_inparanoid.fasta:2428 OB3b\_Genbank\_for\_inparanoid.fasta:2428

```
CQW49_RS05145       	100.00%		CQW49_05140         	100.00%
Bootstrap support for CQW49_RS05145 as seed ortholog is 100%.
Bootstrap support for CQW49_05140 as seed ortholog is 100%.
```

---

### Group of orthologs #29. Best score 2323 bits Score difference with first non-orthologous sequence - OB3b\_Refseq\_for\_inparanoid.fasta:2323 OB3b\_Genbank\_for\_inparanoid.fasta:2323

```
CQW49_RS01825       	100.00%		CQW49_01820         	100.00%
Bootstrap support for CQW49_RS01825 as seed ortholog is 100%.
Bootstrap support for CQW49_01820 as seed ortholog is 100%.
```

---

### Group of orthologs #30. Best score 2300 bits Score difference with first non-orthologous sequence - OB3b\_Refseq\_for\_inparanoid.fasta:2300 OB3b\_Genbank\_for\_inparanoid.fasta:2300

```
CQW49_RS07940       	100.00%		CQW49_07930         	100.00%
Bootstrap support for CQW49_RS07940 as seed ortholog is 100%.
Bootstrap support for CQW49_07930 as seed ortholog is 100%.
```

---

### Group of orthologs #31. Best score 2297 bits Score difference with first non-orthologous sequence - OB3b\_Refseq\_for\_inparanoid.fasta:2297 OB3b\_Genbank\_for\_inparanoid.fasta:1352

```
CQW49_RS05405       	100.00%		CQW49_05400         	100.00%
Bootstrap support for CQW49_RS05405 as seed ortholog is 100%.
Bootstrap support for CQW49_05400 as seed ortholog is 100%.
```

---

### Group of orthologs #32. Best score 2297 bits Score difference with first non-orthologous sequence - OB3b\_Refseq\_for\_inparanoid.fasta:2297 OB3b\_Genbank\_for\_inparanoid.fasta:2297

```
mfd                 	100.00%		CQW49_16665         	100.00%
Bootstrap support for mfd as seed ortholog is 100%.
Bootstrap support for CQW49_16665 as seed ortholog is 100%.
```

---

### Group of orthologs #33. Best score 2287 bits Score difference with first non-orthologous sequence - OB3b\_Refseq\_for\_inparanoid.fasta:2287 OB3b\_Genbank\_for\_inparanoid.fasta:2287

```
CQW49_RS02170       	100.00%		CQW49_02165         	100.00%
Bootstrap support for CQW49_RS02170 as seed ortholog is 100%.
Bootstrap support for CQW49_02165 as seed ortholog is 100%.
```

---

### Group of orthologs #34. Best score 2280 bits Score difference with first non-orthologous sequence - OB3b\_Refseq\_for\_inparanoid.fasta:2280 OB3b\_Genbank\_for\_inparanoid.fasta:2280

```
CQW49_RS08085       	100.00%		CQW49_08075         	100.00%
Bootstrap support for CQW49_RS08085 as seed ortholog is 100%.
Bootstrap support for CQW49_08075 as seed ortholog is 100%.
```

---

### Group of orthologs #35. Best score 2269 bits Score difference with first non-orthologous sequence - OB3b\_Refseq\_for\_inparanoid.fasta:2269 OB3b\_Genbank\_for\_inparanoid.fasta:2269

```
CQW49_RS12670       	100.00%		CQW49_12635         	100.00%
Bootstrap support for CQW49_RS12670 as seed ortholog is 100%.
Bootstrap support for CQW49_12635 as seed ortholog is 100%.
```

---

### Group of orthologs #36. Best score 2265 bits Score difference with first non-orthologous sequence - OB3b\_Refseq\_for\_inparanoid.fasta:2265 OB3b\_Genbank\_for\_inparanoid.fasta:2265

```
CQW49_RS10620       	100.00%		CQW49_10590         	100.00%
Bootstrap support for CQW49_RS10620 as seed ortholog is 100%.
Bootstrap support for CQW49_10590 as seed ortholog is 100%.
```

---

### Group of orthologs #37. Best score 2254 bits Score difference with first non-orthologous sequence - OB3b\_Refseq\_for\_inparanoid.fasta:2254 OB3b\_Genbank\_for\_inparanoid.fasta:2254

```
CQW49_RS20810       	100.00%		CQW49_20735         	100.00%
Bootstrap support for CQW49_RS20810 as seed ortholog is 100%.
Bootstrap support for CQW49_20735 as seed ortholog is 100%.
```

---

### Group of orthologs #38. Best score 2251 bits Score difference with first non-orthologous sequence - OB3b\_Refseq\_for\_inparanoid.fasta:2251 OB3b\_Genbank\_for\_inparanoid.fasta:2251

```
CQW49_RS07105       	100.00%		CQW49_07095         	100.00%
Bootstrap support for CQW49_RS07105 as seed ortholog is 100%.
Bootstrap support for CQW49_07095 as seed ortholog is 100%.
```

---

### Group of orthologs #39. Best score 2250 bits Score difference with first non-orthologous sequence - OB3b\_Refseq\_for\_inparanoid.fasta:2250 OB3b\_Genbank\_for\_inparanoid.fasta:2090

```
CQW49_RS15155       	100.00%		CQW49_15120         	100.00%
Bootstrap support for CQW49_RS15155 as seed ortholog is 100%.
Bootstrap support for CQW49_15120 as seed ortholog is 100%.
```

---

### Group of orthologs #40. Best score 2231 bits Score difference with first non-orthologous sequence - OB3b\_Refseq\_for\_inparanoid.fasta:2231 OB3b\_Genbank\_for\_inparanoid.fasta:1853

```
CQW49_RS01715       	100.00%		CQW49_01710         	100.00%
Bootstrap support for CQW49_RS01715 as seed ortholog is 100%.
Bootstrap support for CQW49_01710 as seed ortholog is 100%.
```

---

### Group of orthologs #41. Best score 2223 bits Score difference with first non-orthologous sequence - OB3b\_Refseq\_for\_inparanoid.fasta:2223 OB3b\_Genbank\_for\_inparanoid.fasta:2223

```
CQW49_RS12450       	100.00%		CQW49_12415         	100.00%
Bootstrap support for CQW49_RS12450 as seed ortholog is 100%.
Bootstrap support for CQW49_12415 as seed ortholog is 100%.
```

---

### Group of orthologs #42. Best score 2215 bits Score difference with first non-orthologous sequence - OB3b\_Refseq\_for\_inparanoid.fasta:2215 OB3b\_Genbank\_for\_inparanoid.fasta:2103

```
addA                	100.00%		CQW49_09355         	100.00%
Bootstrap support for addA as seed ortholog is 100%.
Bootstrap support for CQW49_09355 as seed ortholog is 100%.
```

---

### Group of orthologs #43. Best score 2211 bits Score difference with first non-orthologous sequence - OB3b\_Refseq\_for\_inparanoid.fasta:2211 OB3b\_Genbank\_for\_inparanoid.fasta:2123

```
CQW49_RS13830       	100.00%		CQW49_13795         	100.00%
Bootstrap support for CQW49_RS13830 as seed ortholog is 100%.
Bootstrap support for CQW49_13795 as seed ortholog is 100%.
```

---

### Group of orthologs #44. Best score 2189 bits Score difference with first non-orthologous sequence - OB3b\_Refseq\_for\_inparanoid.fasta:2189 OB3b\_Genbank\_for\_inparanoid.fasta:2189

```
CQW49_RS05945       	100.00%		CQW49_05935         	100.00%
Bootstrap support for CQW49_RS05945 as seed ortholog is 100%.
Bootstrap support for CQW49_05935 as seed ortholog is 100%.
```

---

### Group of orthologs #45. Best score 2179 bits Score difference with first non-orthologous sequence - OB3b\_Refseq\_for\_inparanoid.fasta:2179 OB3b\_Genbank\_for\_inparanoid.fasta:2179

```
CQW49_RS05735       	100.00%		CQW49_05725         	100.00%
Bootstrap support for CQW49_RS05735 as seed ortholog is 100%.
Bootstrap support for CQW49_05725 as seed ortholog is 100%.
```

---

### Group of orthologs #46. Best score 2173 bits Score difference with first non-orthologous sequence - OB3b\_Refseq\_for\_inparanoid.fasta:2173 OB3b\_Genbank\_for\_inparanoid.fasta:2173

```
CQW49_RS18415       	100.00%		CQW49_18355         	100.00%
Bootstrap support for CQW49_RS18415 as seed ortholog is 100%.
Bootstrap support for CQW49_18355 as seed ortholog is 100%.
```

---

### Group of orthologs #47. Best score 2164 bits Score difference with first non-orthologous sequence - OB3b\_Refseq\_for\_inparanoid.fasta:2164 OB3b\_Genbank\_for\_inparanoid.fasta:2164

```
smc                 	100.00%		CQW49_18095         	100.00%
Bootstrap support for smc as seed ortholog is 100%.
Bootstrap support for CQW49_18095 as seed ortholog is 100%.
```

---

### Group of orthologs #48. Best score 2159 bits Score difference with first non-orthologous sequence - OB3b\_Refseq\_for\_inparanoid.fasta:2159 OB3b\_Genbank\_for\_inparanoid.fasta:1260

```
CQW49_RS09805       	100.00%		CQW49_09785         	100.00%
Bootstrap support for CQW49_RS09805 as seed ortholog is 100%.
Bootstrap support for CQW49_09785 as seed ortholog is 100%.
```

---

### Group of orthologs #49. Best score 2148 bits Score difference with first non-orthologous sequence - OB3b\_Refseq\_for\_inparanoid.fasta:2148 OB3b\_Genbank\_for\_inparanoid.fasta:2148

```
cas9                	100.00%		CQW49_07985         	100.00%
Bootstrap support for cas9 as seed ortholog is 100%.
Bootstrap support for CQW49_07985 as seed ortholog is 100%.
```

---

### Group of orthologs #50. Best score 2121 bits Score difference with first non-orthologous sequence - OB3b\_Refseq\_for\_inparanoid.fasta:2121 OB3b\_Genbank\_for\_inparanoid.fasta:2121

```
CQW49_RS08095       	100.00%		CQW49_08085         	100.00%
Bootstrap support for CQW49_RS08095 as seed ortholog is 100%.
Bootstrap support for CQW49_08085 as seed ortholog is 100%.
```

---

### Group of orthologs #51. Best score 2112 bits Score difference with first non-orthologous sequence - OB3b\_Refseq\_for\_inparanoid.fasta:2112 OB3b\_Genbank\_for\_inparanoid.fasta:2112

```
CQW49_RS21180       	100.00%		CQW49_21105         	100.00%
Bootstrap support for CQW49_RS21180 as seed ortholog is 100%.
Bootstrap support for CQW49_21105 as seed ortholog is 100%.
```

---

### Group of orthologs #52. Best score 2110 bits Score difference with first non-orthologous sequence - OB3b\_Refseq\_for\_inparanoid.fasta:2110 OB3b\_Genbank\_for\_inparanoid.fasta:2015

```
CQW49_RS05430       	100.00%		CQW49_05425         	100.00%
Bootstrap support for CQW49_RS05430 as seed ortholog is 100%.
Bootstrap support for CQW49_05425 as seed ortholog is 100%.
```

---

### Group of orthologs #53. Best score 2110 bits Score difference with first non-orthologous sequence - OB3b\_Refseq\_for\_inparanoid.fasta:2110 OB3b\_Genbank\_for\_inparanoid.fasta:2110

```
CQW49_RS20490       	100.00%		CQW49_20420         	100.00%
Bootstrap support for CQW49_RS20490 as seed ortholog is 100%.
Bootstrap support for CQW49_20420 as seed ortholog is 100%.
```

---

### Group of orthologs #54. Best score 2104 bits Score difference with first non-orthologous sequence - OB3b\_Refseq\_for\_inparanoid.fasta:2104 OB3b\_Genbank\_for\_inparanoid.fasta:1078

```
CQW49_RS02085       	100.00%		CQW49_02080         	100.00%
Bootstrap support for CQW49_RS02085 as seed ortholog is 100%.
Bootstrap support for CQW49_02080 as seed ortholog is 100%.
```

---

### Group of orthologs #55. Best score 2096 bits Score difference with first non-orthologous sequence - OB3b\_Refseq\_for\_inparanoid.fasta:2096 OB3b\_Genbank\_for\_inparanoid.fasta:1489

```
CQW49_RS09845       	100.00%		CQW49_09825         	100.00%
Bootstrap support for CQW49_RS09845 as seed ortholog is 100%.
Bootstrap support for CQW49_09825 as seed ortholog is 100%.
```

---

### Group of orthologs #56. Best score 2090 bits Score difference with first non-orthologous sequence - OB3b\_Refseq\_for\_inparanoid.fasta:2090 OB3b\_Genbank\_for\_inparanoid.fasta:2090

```
CQW49_RS13970       	100.00%		CQW49_13935         	100.00%
Bootstrap support for CQW49_RS13970 as seed ortholog is 100%.
Bootstrap support for CQW49_13935 as seed ortholog is 100%.
```

---

### Group of orthologs #57. Best score 2059 bits Score difference with first non-orthologous sequence - OB3b\_Refseq\_for\_inparanoid.fasta:2059 OB3b\_Genbank\_for\_inparanoid.fasta:1023

```
CQW49_RS13675       	100.00%		CQW49_13640         	100.00%
Bootstrap support for CQW49_RS13675 as seed ortholog is 100%.
Bootstrap support for CQW49_13640 as seed ortholog is 100%.
```

---

### Group of orthologs #58. Best score 2052 bits Score difference with first non-orthologous sequence - OB3b\_Refseq\_for\_inparanoid.fasta:2052 OB3b\_Genbank\_for\_inparanoid.fasta:1774

```
CQW49_RS19930       	100.00%		CQW49_19865         	100.00%
Bootstrap support for CQW49_RS19930 as seed ortholog is 100%.
Bootstrap support for CQW49_19865 as seed ortholog is 100%.
```

---

### Group of orthologs #59. Best score 2049 bits Score difference with first non-orthologous sequence - OB3b\_Refseq\_for\_inparanoid.fasta:2049 OB3b\_Genbank\_for\_inparanoid.fasta:996

```
CQW49_RS08385       	100.00%		CQW49_08375         	100.00%
Bootstrap support for CQW49_RS08385 as seed ortholog is 100%.
Bootstrap support for CQW49_08375 as seed ortholog is 100%.
```

---

### Group of orthologs #60. Best score 2042 bits Score difference with first non-orthologous sequence - OB3b\_Refseq\_for\_inparanoid.fasta:2042 OB3b\_Genbank\_for\_inparanoid.fasta:1192

```
CQW49_RS11395       	100.00%		CQW49_11365         	100.00%
Bootstrap support for CQW49_RS11395 as seed ortholog is 100%.
Bootstrap support for CQW49_11365 as seed ortholog is 100%.
```

---

### Group of orthologs #61. Best score 2038 bits Score difference with first non-orthologous sequence - OB3b\_Refseq\_for\_inparanoid.fasta:2038 OB3b\_Genbank\_for\_inparanoid.fasta:2038

```
sucA                	100.00%		CQW49_16390         	100.00%
Bootstrap support for sucA as seed ortholog is 100%.
Bootstrap support for CQW49_16390 as seed ortholog is 100%.
```

---

### Group of orthologs #62. Best score 2015 bits Score difference with first non-orthologous sequence - OB3b\_Refseq\_for\_inparanoid.fasta:2015 OB3b\_Genbank\_for\_inparanoid.fasta:1739

```
CQW49_RS14875       	100.00%		CQW49_14840         	100.00%
Bootstrap support for CQW49_RS14875 as seed ortholog is 100%.
Bootstrap support for CQW49_14840 as seed ortholog is 100%.
```

---

### Group of orthologs #63. Best score 2013 bits Score difference with first non-orthologous sequence - OB3b\_Refseq\_for\_inparanoid.fasta:2013 OB3b\_Genbank\_for\_inparanoid.fasta:2013

```
CQW49_RS16570       	100.00%		CQW49_16530         	100.00%
Bootstrap support for CQW49_RS16570 as seed ortholog is 100%.
Bootstrap support for CQW49_16530 as seed ortholog is 100%.
```

---

### Group of orthologs #64. Best score 1997 bits Score difference with first non-orthologous sequence - OB3b\_Refseq\_for\_inparanoid.fasta:1997 OB3b\_Genbank\_for\_inparanoid.fasta:1997

```
addB                	100.00%		CQW49_09335         	100.00%
Bootstrap support for addB as seed ortholog is 100%.
Bootstrap support for CQW49_09335 as seed ortholog is 100%.
```

---

### Group of orthologs #65. Best score 1994 bits Score difference with first non-orthologous sequence - OB3b\_Refseq\_for\_inparanoid.fasta:1994 OB3b\_Genbank\_for\_inparanoid.fasta:1682

```
CQW49_RS16935       	100.00%		CQW49_16890         	100.00%
Bootstrap support for CQW49_RS16935 as seed ortholog is 100%.
Bootstrap support for CQW49_16890 as seed ortholog is 100%.
```

---

### Group of orthologs #66. Best score 1992 bits Score difference with first non-orthologous sequence - OB3b\_Refseq\_for\_inparanoid.fasta:1992 OB3b\_Genbank\_for\_inparanoid.fasta:1769

```
CQW49_RS04935       	100.00%		CQW49_04930         	100.00%
Bootstrap support for CQW49_RS04935 as seed ortholog is 100%.
Bootstrap support for CQW49_04930 as seed ortholog is 100%.
```

---

### Group of orthologs #67. Best score 1989 bits Score difference with first non-orthologous sequence - OB3b\_Refseq\_for\_inparanoid.fasta:1989 OB3b\_Genbank\_for\_inparanoid.fasta:1989

```
CQW49_RS13955       	100.00%		CQW49_13920         	100.00%
Bootstrap support for CQW49_RS13955 as seed ortholog is 100%.
Bootstrap support for CQW49_13920 as seed ortholog is 100%.
```

---

### Group of orthologs #68. Best score 1985 bits Score difference with first non-orthologous sequence - OB3b\_Refseq\_for\_inparanoid.fasta:1985 OB3b\_Genbank\_for\_inparanoid.fasta:1985

```
CQW49_RS20705       	100.00%		CQW49_20630         	100.00%
Bootstrap support for CQW49_RS20705 as seed ortholog is 100%.
Bootstrap support for CQW49_20630 as seed ortholog is 100%.
```

---

### Group of orthologs #69. Best score 1983 bits Score difference with first non-orthologous sequence - OB3b\_Refseq\_for\_inparanoid.fasta:1983 OB3b\_Genbank\_for\_inparanoid.fasta:1010

```
CQW49_RS01790       	100.00%		CQW49_01785         	100.00%
Bootstrap support for CQW49_RS01790 as seed ortholog is 100%.
Bootstrap support for CQW49_01785 as seed ortholog is 100%.
```

---

### Group of orthologs #70. Best score 1974 bits Score difference with first non-orthologous sequence - OB3b\_Refseq\_for\_inparanoid.fasta:1974 OB3b\_Genbank\_for\_inparanoid.fasta:1974

```
CQW49_RS13965       	100.00%		CQW49_13930         	100.00%
Bootstrap support for CQW49_RS13965 as seed ortholog is 100%.
Bootstrap support for CQW49_13930 as seed ortholog is 100%.
```

---

### Group of orthologs #71. Best score 1971 bits Score difference with first non-orthologous sequence - OB3b\_Refseq\_for\_inparanoid.fasta:1971 OB3b\_Genbank\_for\_inparanoid.fasta:1971

```
CQW49_RS16160       	100.00%		CQW49_16125         	100.00%
Bootstrap support for CQW49_RS16160 as seed ortholog is 100%.
Bootstrap support for CQW49_16125 as seed ortholog is 100%.
```

---

### Group of orthologs #72. Best score 1968 bits Score difference with first non-orthologous sequence - OB3b\_Refseq\_for\_inparanoid.fasta:1968 OB3b\_Genbank\_for\_inparanoid.fasta:1968

```
CQW49_RS16665       	100.00%		CQW49_16625         	100.00%
Bootstrap support for CQW49_RS16665 as seed ortholog is 100%.
Bootstrap support for CQW49_16625 as seed ortholog is 100%.
```

---

### Group of orthologs #73. Best score 1960 bits Score difference with first non-orthologous sequence - OB3b\_Refseq\_for\_inparanoid.fasta:1960 OB3b\_Genbank\_for\_inparanoid.fasta:1685

```
CQW49_RS14895       	100.00%		CQW49_14860         	100.00%
Bootstrap support for CQW49_RS14895 as seed ortholog is 100%.
Bootstrap support for CQW49_14860 as seed ortholog is 100%.
```

---

### Group of orthologs #74. Best score 1957 bits Score difference with first non-orthologous sequence - OB3b\_Refseq\_for\_inparanoid.fasta:1957 OB3b\_Genbank\_for\_inparanoid.fasta:1538

```
CQW49_RS11710       	100.00%		CQW49_11675         	100.00%
Bootstrap support for CQW49_RS11710 as seed ortholog is 100%.
Bootstrap support for CQW49_11675 as seed ortholog is 100%.
```

---

### Group of orthologs #75. Best score 1956 bits Score difference with first non-orthologous sequence - OB3b\_Refseq\_for\_inparanoid.fasta:1956 OB3b\_Genbank\_for\_inparanoid.fasta:1956

```
CQW49_RS17010       	100.00%		CQW49_16965         	100.00%
Bootstrap support for CQW49_RS17010 as seed ortholog is 100%.
Bootstrap support for CQW49_16965 as seed ortholog is 100%.
```

---

### Group of orthologs #76. Best score 1953 bits Score difference with first non-orthologous sequence - OB3b\_Refseq\_for\_inparanoid.fasta:1953 OB3b\_Genbank\_for\_inparanoid.fasta:816

```
CQW49_RS08380       	100.00%		CQW49_08370         	100.00%
Bootstrap support for CQW49_RS08380 as seed ortholog is 100%.
Bootstrap support for CQW49_08370 as seed ortholog is 100%.
```

---

### Group of orthologs #77. Best score 1953 bits Score difference with first non-orthologous sequence - OB3b\_Refseq\_for\_inparanoid.fasta:1953 OB3b\_Genbank\_for\_inparanoid.fasta:847

```
CQW49_RS13680       	100.00%		CQW49_13645         	100.00%
Bootstrap support for CQW49_RS13680 as seed ortholog is 100%.
Bootstrap support for CQW49_13645 as seed ortholog is 100%.
```

---

### Group of orthologs #78. Best score 1946 bits Score difference with first non-orthologous sequence - OB3b\_Refseq\_for\_inparanoid.fasta:1946 OB3b\_Genbank\_for\_inparanoid.fasta:1946

```
CQW49_RS03165       	100.00%		CQW49_03155         	100.00%
Bootstrap support for CQW49_RS03165 as seed ortholog is 100%.
Bootstrap support for CQW49_03155 as seed ortholog is 100%.
```

---

### Group of orthologs #79. Best score 1935 bits Score difference with first non-orthologous sequence - OB3b\_Refseq\_for\_inparanoid.fasta:1935 OB3b\_Genbank\_for\_inparanoid.fasta:1078

```
CQW49_RS01795       	100.00%		CQW49_01790         	100.00%
Bootstrap support for CQW49_RS01795 as seed ortholog is 100%.
Bootstrap support for CQW49_01790 as seed ortholog is 100%.
```

---

### Group of orthologs #80. Best score 1930 bits Score difference with first non-orthologous sequence - OB3b\_Refseq\_for\_inparanoid.fasta:1930 OB3b\_Genbank\_for\_inparanoid.fasta:1930

```
CQW49_RS13000       	100.00%		CQW49_12965         	100.00%
Bootstrap support for CQW49_RS13000 as seed ortholog is 100%.
Bootstrap support for CQW49_12965 as seed ortholog is 100%.
```

---

### Group of orthologs #81. Best score 1925 bits Score difference with first non-orthologous sequence - OB3b\_Refseq\_for\_inparanoid.fasta:1925 OB3b\_Genbank\_for\_inparanoid.fasta:1925

```
CQW49_RS13135       	100.00%		CQW49_13100         	100.00%
Bootstrap support for CQW49_RS13135 as seed ortholog is 100%.
Bootstrap support for CQW49_13100 as seed ortholog is 100%.
```

---

### Group of orthologs #82. Best score 1925 bits Score difference with first non-orthologous sequence - OB3b\_Refseq\_for\_inparanoid.fasta:1925 OB3b\_Genbank\_for\_inparanoid.fasta:1437

```
CQW49_RS17215       	100.00%		CQW49_17170         	100.00%
Bootstrap support for CQW49_RS17215 as seed ortholog is 100%.
Bootstrap support for CQW49_17170 as seed ortholog is 100%.
```

---

### Group of orthologs #83. Best score 1913 bits Score difference with first non-orthologous sequence - OB3b\_Refseq\_for\_inparanoid.fasta:1913 OB3b\_Genbank\_for\_inparanoid.fasta:1913

```
topA                	100.00%		CQW49_02290         	100.00%
Bootstrap support for topA as seed ortholog is 100%.
Bootstrap support for CQW49_02290 as seed ortholog is 100%.
```

---

### Group of orthologs #84. Best score 1911 bits Score difference with first non-orthologous sequence - OB3b\_Refseq\_for\_inparanoid.fasta:1911 OB3b\_Genbank\_for\_inparanoid.fasta:1911

```
traA                	100.00%		CQW49_08920         	100.00%
Bootstrap support for traA as seed ortholog is 100%.
Bootstrap support for CQW49_08920 as seed ortholog is 100%.
```

---

### Group of orthologs #85. Best score 1900 bits Score difference with first non-orthologous sequence - OB3b\_Refseq\_for\_inparanoid.fasta:1900 OB3b\_Genbank\_for\_inparanoid.fasta:1779

```
CQW49_RS13720       	100.00%		CQW49_13685         	100.00%
Bootstrap support for CQW49_RS13720 as seed ortholog is 100%.
Bootstrap support for CQW49_13685 as seed ortholog is 100%.
```

---

### Group of orthologs #86. Best score 1893 bits Score difference with first non-orthologous sequence - OB3b\_Refseq\_for\_inparanoid.fasta:1893 OB3b\_Genbank\_for\_inparanoid.fasta:1534

```
CQW49_RS08345       	100.00%		CQW49_08335         	100.00%
Bootstrap support for CQW49_RS08345 as seed ortholog is 100%.
Bootstrap support for CQW49_08335 as seed ortholog is 100%.
```

---

### Group of orthologs #87. Best score 1885 bits Score difference with first non-orthologous sequence - OB3b\_Refseq\_for\_inparanoid.fasta:1885 OB3b\_Genbank\_for\_inparanoid.fasta:1885

```
CQW49_RS12765       	100.00%		CQW49_12730         	100.00%
Bootstrap support for CQW49_RS12765 as seed ortholog is 100%.
Bootstrap support for CQW49_12730 as seed ortholog is 100%.
```

---

### Group of orthologs #88. Best score 1878 bits Score difference with first non-orthologous sequence - OB3b\_Refseq\_for\_inparanoid.fasta:1878 OB3b\_Genbank\_for\_inparanoid.fasta:1878

```
CQW49_RS07365       	100.00%		CQW49_07355         	100.00%
Bootstrap support for CQW49_RS07365 as seed ortholog is 100%.
Bootstrap support for CQW49_07355 as seed ortholog is 100%.
```

---

### Group of orthologs #89. Best score 1878 bits Score difference with first non-orthologous sequence - OB3b\_Refseq\_for\_inparanoid.fasta:1878 OB3b\_Genbank\_for\_inparanoid.fasta:1878

```
CQW49_RS15900       	100.00%		CQW49_15865         	100.00%
Bootstrap support for CQW49_RS15900 as seed ortholog is 100%.
Bootstrap support for CQW49_15865 as seed ortholog is 100%.
```

---

### Group of orthologs #90. Best score 1872 bits Score difference with first non-orthologous sequence - OB3b\_Refseq\_for\_inparanoid.fasta:1872 OB3b\_Genbank\_for\_inparanoid.fasta:1693

```
CQW49_RS02135       	100.00%		CQW49_02130         	100.00%
Bootstrap support for CQW49_RS02135 as seed ortholog is 100%.
Bootstrap support for CQW49_02130 as seed ortholog is 100%.
```

---

### Group of orthologs #91. Best score 1871 bits Score difference with first non-orthologous sequence - OB3b\_Refseq\_for\_inparanoid.fasta:1871 OB3b\_Genbank\_for\_inparanoid.fasta:1871

```
CQW49_RS01040       	100.00%		CQW49_01040         	100.00%
Bootstrap support for CQW49_RS01040 as seed ortholog is 100%.
Bootstrap support for CQW49_01040 as seed ortholog is 100%.
```

---

### Group of orthologs #92. Best score 1871 bits Score difference with first non-orthologous sequence - OB3b\_Refseq\_for\_inparanoid.fasta:1871 OB3b\_Genbank\_for\_inparanoid.fasta:1871

```
cas3u               	100.00%		CQW49_05800         	100.00%
Bootstrap support for cas3u as seed ortholog is 100%.
Bootstrap support for CQW49_05800 as seed ortholog is 100%.
```

---

### Group of orthologs #93. Best score 1849 bits Score difference with first non-orthologous sequence - OB3b\_Refseq\_for\_inparanoid.fasta:1849 OB3b\_Genbank\_for\_inparanoid.fasta:1849

```
CQW49_RS08020       	100.00%		CQW49_08010         	100.00%
Bootstrap support for CQW49_RS08020 as seed ortholog is 100%.
Bootstrap support for CQW49_08010 as seed ortholog is 100%.
```

---

### Group of orthologs #94. Best score 1848 bits Score difference with first non-orthologous sequence - OB3b\_Refseq\_for\_inparanoid.fasta:1848 OB3b\_Genbank\_for\_inparanoid.fasta:1848

```
CQW49_RS19455       	100.00%		CQW49_19400         	100.00%
Bootstrap support for CQW49_RS19455 as seed ortholog is 100%.
Bootstrap support for CQW49_19400 as seed ortholog is 100%.
```

---

### Group of orthologs #95. Best score 1841 bits Score difference with first non-orthologous sequence - OB3b\_Refseq\_for\_inparanoid.fasta:1841 OB3b\_Genbank\_for\_inparanoid.fasta:1841

```
CQW49_RS16155       	100.00%		CQW49_16120         	100.00%
Bootstrap support for CQW49_RS16155 as seed ortholog is 100%.
Bootstrap support for CQW49_16120 as seed ortholog is 100%.
```

---

### Group of orthologs #96. Best score 1833 bits Score difference with first non-orthologous sequence - OB3b\_Refseq\_for\_inparanoid.fasta:1833 OB3b\_Genbank\_for\_inparanoid.fasta:1833

```
CQW49_RS11735       	100.00%		CQW49_11700         	100.00%
Bootstrap support for CQW49_RS11735 as seed ortholog is 100%.
Bootstrap support for CQW49_11700 as seed ortholog is 100%.
```

---

### Group of orthologs #97. Best score 1824 bits Score difference with first non-orthologous sequence - OB3b\_Refseq\_for\_inparanoid.fasta:1824 OB3b\_Genbank\_for\_inparanoid.fasta:1824

```
CQW49_RS08640       	100.00%		CQW49_08630         	100.00%
Bootstrap support for CQW49_RS08640 as seed ortholog is 100%.
Bootstrap support for CQW49_08630 as seed ortholog is 100%.
```

---

### Group of orthologs #98. Best score 1824 bits Score difference with first non-orthologous sequence - OB3b\_Refseq\_for\_inparanoid.fasta:1824 OB3b\_Genbank\_for\_inparanoid.fasta:1728

```
CQW49_RS12400       	100.00%		CQW49_12365         	100.00%
Bootstrap support for CQW49_RS12400 as seed ortholog is 100%.
Bootstrap support for CQW49_12365 as seed ortholog is 100%.
```

---

### Group of orthologs #99. Best score 1822 bits Score difference with first non-orthologous sequence - OB3b\_Refseq\_for\_inparanoid.fasta:1822 OB3b\_Genbank\_for\_inparanoid.fasta:1822

```
CQW49_RS20965       	100.00%		CQW49_20890         	100.00%
Bootstrap support for CQW49_RS20965 as seed ortholog is 100%.
Bootstrap support for CQW49_20890 as seed ortholog is 100%.
```

---

### Group of orthologs #100. Best score 1815 bits Score difference with first non-orthologous sequence - OB3b\_Refseq\_for\_inparanoid.fasta:1815 OB3b\_Genbank\_for\_inparanoid.fasta:1815

```
acnA                	100.00%		CQW49_13135         	100.00%
Bootstrap support for acnA as seed ortholog is 100%.
Bootstrap support for CQW49_13135 as seed ortholog is 100%.
```

---

### Group of orthologs #101. Best score 1806 bits Score difference with first non-orthologous sequence - OB3b\_Refseq\_for\_inparanoid.fasta:1806 OB3b\_Genbank\_for\_inparanoid.fasta:1806

```
CQW49_RS07355       	100.00%		CQW49_07345         	100.00%
Bootstrap support for CQW49_RS07355 as seed ortholog is 100%.
Bootstrap support for CQW49_07345 as seed ortholog is 100%.
```

---

### Group of orthologs #102. Best score 1806 bits Score difference with first non-orthologous sequence - OB3b\_Refseq\_for\_inparanoid.fasta:1806 OB3b\_Genbank\_for\_inparanoid.fasta:1356

```
CQW49_RS15755       	100.00%		CQW49_15720         	100.00%
Bootstrap support for CQW49_RS15755 as seed ortholog is 100%.
Bootstrap support for CQW49_15720 as seed ortholog is 100%.
```

---

### Group of orthologs #103. Best score 1800 bits Score difference with first non-orthologous sequence - OB3b\_Refseq\_for\_inparanoid.fasta:1800 OB3b\_Genbank\_for\_inparanoid.fasta:1800

```
CQW49_RS10690       	100.00%		CQW49_10660         	100.00%
Bootstrap support for CQW49_RS10690 as seed ortholog is 100%.
Bootstrap support for CQW49_10660 as seed ortholog is 100%.
```

---

### Group of orthologs #104. Best score 1792 bits Score difference with first non-orthologous sequence - OB3b\_Refseq\_for\_inparanoid.fasta:1792 OB3b\_Genbank\_for\_inparanoid.fasta:1792

```
CQW49_RS10130       	100.00%		CQW49_10105         	100.00%
Bootstrap support for CQW49_RS10130 as seed ortholog is 100%.
Bootstrap support for CQW49_10105 as seed ortholog is 100%.
```

---

### Group of orthologs #105. Best score 1789 bits Score difference with first non-orthologous sequence - OB3b\_Refseq\_for\_inparanoid.fasta:1789 OB3b\_Genbank\_for\_inparanoid.fasta:1789

```
CQW49_RS05100       	100.00%		CQW49_05095         	100.00%
Bootstrap support for CQW49_RS05100 as seed ortholog is 100%.
Bootstrap support for CQW49_05095 as seed ortholog is 100%.
```

---

### Group of orthologs #106. Best score 1788 bits Score difference with first non-orthologous sequence - OB3b\_Refseq\_for\_inparanoid.fasta:1788 OB3b\_Genbank\_for\_inparanoid.fasta:1788

```
CQW49_RS17290       	100.00%		CQW49_17245         	100.00%
Bootstrap support for CQW49_RS17290 as seed ortholog is 100%.
Bootstrap support for CQW49_17245 as seed ortholog is 100%.
```

---

### Group of orthologs #107. Best score 1788 bits Score difference with first non-orthologous sequence - OB3b\_Refseq\_for\_inparanoid.fasta:1788 OB3b\_Genbank\_for\_inparanoid.fasta:1788

```
CQW49_RS19355       	100.00%		CQW49_19300         	100.00%
Bootstrap support for CQW49_RS19355 as seed ortholog is 100%.
Bootstrap support for CQW49_19300 as seed ortholog is 100%.
```

---

### Group of orthologs #108. Best score 1780 bits Score difference with first non-orthologous sequence - OB3b\_Refseq\_for\_inparanoid.fasta:1780 OB3b\_Genbank\_for\_inparanoid.fasta:1780

```
CQW49_RS10965       	100.00%		CQW49_10935         	100.00%
Bootstrap support for CQW49_RS10965 as seed ortholog is 100%.
Bootstrap support for CQW49_10935 as seed ortholog is 100%.
```

---

### Group of orthologs #109. Best score 1772 bits Score difference with first non-orthologous sequence - OB3b\_Refseq\_for\_inparanoid.fasta:1772 OB3b\_Genbank\_for\_inparanoid.fasta:1772

```
CQW49_RS12265       	100.00%		CQW49_12230         	100.00%
Bootstrap support for CQW49_RS12265 as seed ortholog is 100%.
Bootstrap support for CQW49_12230 as seed ortholog is 100%.
```

---

### Group of orthologs #110. Best score 1766 bits Score difference with first non-orthologous sequence - OB3b\_Refseq\_for\_inparanoid.fasta:1766 OB3b\_Genbank\_for\_inparanoid.fasta:1766

```
CQW49_RS04965       	100.00%		CQW49_04960         	100.00%
Bootstrap support for CQW49_RS04965 as seed ortholog is 100%.
Bootstrap support for CQW49_04960 as seed ortholog is 100%.
```

---

### Group of orthologs #111. Best score 1762 bits Score difference with first non-orthologous sequence - OB3b\_Refseq\_for\_inparanoid.fasta:1762 OB3b\_Genbank\_for\_inparanoid.fasta:1762

```
CQW49_RS17305       	100.00%		CQW49_17260         	100.00%
Bootstrap support for CQW49_RS17305 as seed ortholog is 100%.
Bootstrap support for CQW49_17260 as seed ortholog is 100%.
```

---

### Group of orthologs #112. Best score 1757 bits Score difference with first non-orthologous sequence - OB3b\_Refseq\_for\_inparanoid.fasta:1757 OB3b\_Genbank\_for\_inparanoid.fasta:1757

```
CQW49_RS02060       	100.00%		CQW49_02055         	100.00%
Bootstrap support for CQW49_RS02060 as seed ortholog is 100%.
Bootstrap support for CQW49_02055 as seed ortholog is 100%.
```

---

### Group of orthologs #113. Best score 1757 bits Score difference with first non-orthologous sequence - OB3b\_Refseq\_for\_inparanoid.fasta:1757 OB3b\_Genbank\_for\_inparanoid.fasta:1757

```
CQW49_RS11135       	100.00%		CQW49_11105         	100.00%
Bootstrap support for CQW49_RS11135 as seed ortholog is 100%.
Bootstrap support for CQW49_11105 as seed ortholog is 100%.
```

---

### Group of orthologs #114. Best score 1741 bits Score difference with first non-orthologous sequence - OB3b\_Refseq\_for\_inparanoid.fasta:1741 OB3b\_Genbank\_for\_inparanoid.fasta:1741

```
CQW49_RS15855       	100.00%		CQW49_15820         	100.00%
Bootstrap support for CQW49_RS15855 as seed ortholog is 100%.
Bootstrap support for CQW49_15820 as seed ortholog is 100%.
```

---

### Group of orthologs #115. Best score 1737 bits Score difference with first non-orthologous sequence - OB3b\_Refseq\_for\_inparanoid.fasta:1737 OB3b\_Genbank\_for\_inparanoid.fasta:1737

```
CQW49_RS04525       	100.00%		CQW49_04520         	100.00%
Bootstrap support for CQW49_RS04525 as seed ortholog is 100%.
Bootstrap support for CQW49_04520 as seed ortholog is 100%.
```

---

### Group of orthologs #116. Best score 1735 bits Score difference with first non-orthologous sequence - OB3b\_Refseq\_for\_inparanoid.fasta:1735 OB3b\_Genbank\_for\_inparanoid.fasta:1633

```
CQW49_RS06445       	100.00%		CQW49_06435         	100.00%
Bootstrap support for CQW49_RS06445 as seed ortholog is 100%.
Bootstrap support for CQW49_06435 as seed ortholog is 100%.
```

---

### Group of orthologs #117. Best score 1734 bits Score difference with first non-orthologous sequence - OB3b\_Refseq\_for\_inparanoid.fasta:1734 OB3b\_Genbank\_for\_inparanoid.fasta:1734

```
CQW49_RS11185       	100.00%		CQW49_11155         	100.00%
Bootstrap support for CQW49_RS11185 as seed ortholog is 100%.
Bootstrap support for CQW49_11155 as seed ortholog is 100%.
```

---

### Group of orthologs #118. Best score 1732 bits Score difference with first non-orthologous sequence - OB3b\_Refseq\_for\_inparanoid.fasta:1732 OB3b\_Genbank\_for\_inparanoid.fasta:1732

```
CQW49_RS19515       	100.00%		CQW49_19460         	100.00%
Bootstrap support for CQW49_RS19515 as seed ortholog is 100%.
Bootstrap support for CQW49_19460 as seed ortholog is 100%.
```

---

### Group of orthologs #119. Best score 1728 bits Score difference with first non-orthologous sequence - OB3b\_Refseq\_for\_inparanoid.fasta:1728 OB3b\_Genbank\_for\_inparanoid.fasta:1728

```
CQW49_RS02080       	100.00%		CQW49_02075         	100.00%
Bootstrap support for CQW49_RS02080 as seed ortholog is 100%.
Bootstrap support for CQW49_02075 as seed ortholog is 100%.
```

---

### Group of orthologs #120. Best score 1722 bits Score difference with first non-orthologous sequence - OB3b\_Refseq\_for\_inparanoid.fasta:1722 OB3b\_Genbank\_for\_inparanoid.fasta:1722

```
CQW49_RS05040       	100.00%		CQW49_05035         	100.00%
Bootstrap support for CQW49_RS05040 as seed ortholog is 100%.
Bootstrap support for CQW49_05035 as seed ortholog is 100%.
```

---

### Group of orthologs #121. Best score 1719 bits Score difference with first non-orthologous sequence - OB3b\_Refseq\_for\_inparanoid.fasta:1719 OB3b\_Genbank\_for\_inparanoid.fasta:1067

```
CQW49_RS02600       	100.00%		CQW49_02590         	100.00%
Bootstrap support for CQW49_RS02600 as seed ortholog is 100%.
Bootstrap support for CQW49_02590 as seed ortholog is 100%.
```

---

### Group of orthologs #122. Best score 1716 bits Score difference with first non-orthologous sequence - OB3b\_Refseq\_for\_inparanoid.fasta:1716 OB3b\_Genbank\_for\_inparanoid.fasta:1716

```
CQW49_RS10150       	100.00%		CQW49_10125         	100.00%
Bootstrap support for CQW49_RS10150 as seed ortholog is 100%.
Bootstrap support for CQW49_10125 as seed ortholog is 100%.
```

---

### Group of orthologs #123. Best score 1704 bits Score difference with first non-orthologous sequence - OB3b\_Refseq\_for\_inparanoid.fasta:1704 OB3b\_Genbank\_for\_inparanoid.fasta:1704

```
CQW49_RS19070       	100.00%		CQW49_19010         	100.00%
Bootstrap support for CQW49_RS19070 as seed ortholog is 100%.
Bootstrap support for CQW49_19010 as seed ortholog is 100%.
```

---

### Group of orthologs #124. Best score 1703 bits Score difference with first non-orthologous sequence - OB3b\_Refseq\_for\_inparanoid.fasta:1703 OB3b\_Genbank\_for\_inparanoid.fasta:1703

```
CQW49_RS01770       	100.00%		CQW49_01765         	100.00%
Bootstrap support for CQW49_RS01770 as seed ortholog is 100%.
Bootstrap support for CQW49_01765 as seed ortholog is 100%.
```

---

### Group of orthologs #125. Best score 1701 bits Score difference with first non-orthologous sequence - OB3b\_Refseq\_for\_inparanoid.fasta:1701 OB3b\_Genbank\_for\_inparanoid.fasta:1701

```
CQW49_RS02995       	100.00%		CQW49_02985         	100.00%
Bootstrap support for CQW49_RS02995 as seed ortholog is 100%.
Bootstrap support for CQW49_02985 as seed ortholog is 100%.
```

---

### Group of orthologs #126. Best score 1694 bits Score difference with first non-orthologous sequence - OB3b\_Refseq\_for\_inparanoid.fasta:1694 OB3b\_Genbank\_for\_inparanoid.fasta:1694

```
CQW49_RS13940       	100.00%		CQW49_13905         	100.00%
Bootstrap support for CQW49_RS13940 as seed ortholog is 100%.
Bootstrap support for CQW49_13905 as seed ortholog is 100%.
```

---

### Group of orthologs #127. Best score 1693 bits Score difference with first non-orthologous sequence - OB3b\_Refseq\_for\_inparanoid.fasta:1693 OB3b\_Genbank\_for\_inparanoid.fasta:1008

```
CQW49_RS02430       	100.00%		CQW49_02420         	100.00%
Bootstrap support for CQW49_RS02430 as seed ortholog is 100%.
Bootstrap support for CQW49_02420 as seed ortholog is 100%.
```

---

### Group of orthologs #128. Best score 1689 bits Score difference with first non-orthologous sequence - OB3b\_Refseq\_for\_inparanoid.fasta:1689 OB3b\_Genbank\_for\_inparanoid.fasta:838

```
CQW49_RS18175       	100.00%		CQW49_18115         	100.00%
Bootstrap support for CQW49_RS18175 as seed ortholog is 100%.
Bootstrap support for CQW49_18115 as seed ortholog is 100%.
```

---

### Group of orthologs #129. Best score 1675 bits Score difference with first non-orthologous sequence - OB3b\_Refseq\_for\_inparanoid.fasta:1675 OB3b\_Genbank\_for\_inparanoid.fasta:1675

```
CQW49_RS13925       	100.00%		CQW49_13890         	100.00%
Bootstrap support for CQW49_RS13925 as seed ortholog is 100%.
Bootstrap support for CQW49_13890 as seed ortholog is 100%.
```

---

### Group of orthologs #130. Best score 1673 bits Score difference with first non-orthologous sequence - OB3b\_Refseq\_for\_inparanoid.fasta:1673 OB3b\_Genbank\_for\_inparanoid.fasta:1673

```
CQW49_RS16775       	100.00%		CQW49_16735         	100.00%
Bootstrap support for CQW49_RS16775 as seed ortholog is 100%.
Bootstrap support for CQW49_16735 as seed ortholog is 100%.
```

---

### Group of orthologs #131. Best score 1671 bits Score difference with first non-orthologous sequence - OB3b\_Refseq\_for\_inparanoid.fasta:1671 OB3b\_Genbank\_for\_inparanoid.fasta:1671

```
CQW49_RS16815       	100.00%		CQW49_16775         	100.00%
Bootstrap support for CQW49_RS16815 as seed ortholog is 100%.
Bootstrap support for CQW49_16775 as seed ortholog is 100%.
```

---

### Group of orthologs #132. Best score 1670 bits Score difference with first non-orthologous sequence - OB3b\_Refseq\_for\_inparanoid.fasta:1670 OB3b\_Genbank\_for\_inparanoid.fasta:1670

```
CQW49_RS03310       	100.00%		CQW49_03300         	100.00%
Bootstrap support for CQW49_RS03310 as seed ortholog is 100%.
Bootstrap support for CQW49_03300 as seed ortholog is 100%.
```

---

### Group of orthologs #133. Best score 1669 bits Score difference with first non-orthologous sequence - OB3b\_Refseq\_for\_inparanoid.fasta:1669 OB3b\_Genbank\_for\_inparanoid.fasta:1669

```
mgtA                	100.00%		CQW49_17395         	100.00%
Bootstrap support for mgtA as seed ortholog is 100%.
Bootstrap support for CQW49_17395 as seed ortholog is 100%.
```

---

### Group of orthologs #134. Best score 1667 bits Score difference with first non-orthologous sequence - OB3b\_Refseq\_for\_inparanoid.fasta:1667 OB3b\_Genbank\_for\_inparanoid.fasta:1667

```
clpB                	100.00%		CQW49_18880         	100.00%
Bootstrap support for clpB as seed ortholog is 100%.
Bootstrap support for CQW49_18880 as seed ortholog is 100%.
```

---

### Group of orthologs #135. Best score 1663 bits Score difference with first non-orthologous sequence - OB3b\_Refseq\_for\_inparanoid.fasta:1663 OB3b\_Genbank\_for\_inparanoid.fasta:1663

```
CQW49_RS15505       	100.00%		CQW49_15470         	100.00%
Bootstrap support for CQW49_RS15505 as seed ortholog is 100%.
Bootstrap support for CQW49_15470 as seed ortholog is 100%.
```

---

### Group of orthologs #136. Best score 1662 bits Score difference with first non-orthologous sequence - OB3b\_Refseq\_for\_inparanoid.fasta:1662 OB3b\_Genbank\_for\_inparanoid.fasta:963

```
CQW49_RS17585       	100.00%		CQW49_17530         	100.00%
Bootstrap support for CQW49_RS17585 as seed ortholog is 100%.
Bootstrap support for CQW49_17530 as seed ortholog is 100%.
```

---

### Group of orthologs #137. Best score 1661 bits Score difference with first non-orthologous sequence - OB3b\_Refseq\_for\_inparanoid.fasta:1661 OB3b\_Genbank\_for\_inparanoid.fasta:1661

```
CQW49_RS10140       	100.00%		CQW49_10115         	100.00%
Bootstrap support for CQW49_RS10140 as seed ortholog is 100%.
Bootstrap support for CQW49_10115 as seed ortholog is 100%.
```

---

### Group of orthologs #138. Best score 1661 bits Score difference with first non-orthologous sequence - OB3b\_Refseq\_for\_inparanoid.fasta:1661 OB3b\_Genbank\_for\_inparanoid.fasta:688

```
CQW49_RS13695       	100.00%		CQW49_13660         	100.00%
Bootstrap support for CQW49_RS13695 as seed ortholog is 100%.
Bootstrap support for CQW49_13660 as seed ortholog is 100%.
```

---

### Group of orthologs #139. Best score 1656 bits Score difference with first non-orthologous sequence - OB3b\_Refseq\_for\_inparanoid.fasta:1656 OB3b\_Genbank\_for\_inparanoid.fasta:1656

```
bamA                	100.00%		CQW49_00465         	100.00%
Bootstrap support for bamA as seed ortholog is 100%.
Bootstrap support for CQW49_00465 as seed ortholog is 100%.
```

---

### Group of orthologs #140. Best score 1656 bits Score difference with first non-orthologous sequence - OB3b\_Refseq\_for\_inparanoid.fasta:1656 OB3b\_Genbank\_for\_inparanoid.fasta:1528

```
CQW49_RS03435       	100.00%		CQW49_03420         	100.00%
Bootstrap support for CQW49_RS03435 as seed ortholog is 100%.
Bootstrap support for CQW49_03420 as seed ortholog is 100%.
```

---

### Group of orthologs #141. Best score 1656 bits Score difference with first non-orthologous sequence - OB3b\_Refseq\_for\_inparanoid.fasta:1656 OB3b\_Genbank\_for\_inparanoid.fasta:1656

```
CQW49_RS04460       	100.00%		CQW49_04455         	100.00%
Bootstrap support for CQW49_RS04460 as seed ortholog is 100%.
Bootstrap support for CQW49_04455 as seed ortholog is 100%.
```

---

### Group of orthologs #142. Best score 1656 bits Score difference with first non-orthologous sequence - OB3b\_Refseq\_for\_inparanoid.fasta:1656 OB3b\_Genbank\_for\_inparanoid.fasta:1656

```
CQW49_RS13750       	100.00%		CQW49_13715         	100.00%
Bootstrap support for CQW49_RS13750 as seed ortholog is 100%.
Bootstrap support for CQW49_13715 as seed ortholog is 100%.
```

---

### Group of orthologs #143. Best score 1654 bits Score difference with first non-orthologous sequence - OB3b\_Refseq\_for\_inparanoid.fasta:1654 OB3b\_Genbank\_for\_inparanoid.fasta:1281

```
ctaD                	100.00%		CQW49_13005         	100.00%
ctaD                	100.00%		
ctaD                	100.00%		
ctaD                	100.00%		
Bootstrap support for ctaD as seed ortholog is 100%.
Bootstrap support for ctaD as seed ortholog is 100%.
Bootstrap support for ctaD as seed ortholog is 100%.
Bootstrap support for ctaD as seed ortholog is 100%.
Bootstrap support for CQW49_13005 as seed ortholog is 100%.
```

---

### Group of orthologs #144. Best score 1654 bits Score difference with first non-orthologous sequence - OB3b\_Refseq\_for\_inparanoid.fasta:1654 OB3b\_Genbank\_for\_inparanoid.fasta:1654

```
CQW49_RS12665       	100.00%		CQW49_12630         	100.00%
Bootstrap support for CQW49_RS12665 as seed ortholog is 100%.
Bootstrap support for CQW49_12630 as seed ortholog is 100%.
```

---

### Group of orthologs #145. Best score 1654 bits Score difference with first non-orthologous sequence - OB3b\_Refseq\_for\_inparanoid.fasta:1654 OB3b\_Genbank\_for\_inparanoid.fasta:468

```
CQW49_RS18235       	100.00%		CQW49_18175         	100.00%
Bootstrap support for CQW49_RS18235 as seed ortholog is 100%.
Bootstrap support for CQW49_18175 as seed ortholog is 100%.
```

---

### Group of orthologs #146. Best score 1653 bits Score difference with first non-orthologous sequence - OB3b\_Refseq\_for\_inparanoid.fasta:1653 OB3b\_Genbank\_for\_inparanoid.fasta:1653

```
CQW49_RS14340       	100.00%		CQW49_14305         	100.00%
Bootstrap support for CQW49_RS14340 as seed ortholog is 100%.
Bootstrap support for CQW49_14305 as seed ortholog is 100%.
```

---

### Group of orthologs #147. Best score 1636 bits Score difference with first non-orthologous sequence - OB3b\_Refseq\_for\_inparanoid.fasta:1636 OB3b\_Genbank\_for\_inparanoid.fasta:1636

```
CQW49_RS03090       	100.00%		CQW49_03080         	100.00%
Bootstrap support for CQW49_RS03090 as seed ortholog is 100%.
Bootstrap support for CQW49_03080 as seed ortholog is 100%.
```

---

### Group of orthologs #148. Best score 1635 bits Score difference with first non-orthologous sequence - OB3b\_Refseq\_for\_inparanoid.fasta:1635 OB3b\_Genbank\_for\_inparanoid.fasta:885

```
CQW49_RS07180       	100.00%		CQW49_07170         	100.00%
Bootstrap support for CQW49_RS07180 as seed ortholog is 100%.
Bootstrap support for CQW49_07170 as seed ortholog is 100%.
```

---

### Group of orthologs #149. Best score 1633 bits Score difference with first non-orthologous sequence - OB3b\_Refseq\_for\_inparanoid.fasta:1633 OB3b\_Genbank\_for\_inparanoid.fasta:1633

```
CQW49_RS04520       	100.00%		CQW49_04515         	100.00%
Bootstrap support for CQW49_RS04520 as seed ortholog is 100%.
Bootstrap support for CQW49_04515 as seed ortholog is 100%.
```

---

### Group of orthologs #150. Best score 1627 bits Score difference with first non-orthologous sequence - OB3b\_Refseq\_for\_inparanoid.fasta:1627 OB3b\_Genbank\_for\_inparanoid.fasta:1627

```
CQW49_RS10240       	100.00%		CQW49_10215         	100.00%
Bootstrap support for CQW49_RS10240 as seed ortholog is 100%.
Bootstrap support for CQW49_10215 as seed ortholog is 100%.
```

---

### Group of orthologs #151. Best score 1625 bits Score difference with first non-orthologous sequence - OB3b\_Refseq\_for\_inparanoid.fasta:1625 OB3b\_Genbank\_for\_inparanoid.fasta:1625

```
CQW49_RS19365       	100.00%		CQW49_19310         	100.00%
Bootstrap support for CQW49_RS19365 as seed ortholog is 100%.
Bootstrap support for CQW49_19310 as seed ortholog is 100%.
```

---

### Group of orthologs #152. Best score 1622 bits Score difference with first non-orthologous sequence - OB3b\_Refseq\_for\_inparanoid.fasta:1622 OB3b\_Genbank\_for\_inparanoid.fasta:1622

```
CQW49_RS01470       	100.00%		CQW49_01465         	100.00%
Bootstrap support for CQW49_RS01470 as seed ortholog is 100%.
Bootstrap support for CQW49_01465 as seed ortholog is 100%.
```

---

### Group of orthologs #153. Best score 1621 bits Score difference with first non-orthologous sequence - OB3b\_Refseq\_for\_inparanoid.fasta:1621 OB3b\_Genbank\_for\_inparanoid.fasta:1459

```
CQW49_RS08575       	100.00%		CQW49_08565         	100.00%
Bootstrap support for CQW49_RS08575 as seed ortholog is 100%.
Bootstrap support for CQW49_08565 as seed ortholog is 100%.
```

---

### Group of orthologs #154. Best score 1619 bits Score difference with first non-orthologous sequence - OB3b\_Refseq\_for\_inparanoid.fasta:1619 OB3b\_Genbank\_for\_inparanoid.fasta:1619

```
CQW49_RS14140       	100.00%		CQW49_14105         	100.00%
Bootstrap support for CQW49_RS14140 as seed ortholog is 100%.
Bootstrap support for CQW49_14105 as seed ortholog is 100%.
```

---

### Group of orthologs #155. Best score 1615 bits Score difference with first non-orthologous sequence - OB3b\_Refseq\_for\_inparanoid.fasta:1615 OB3b\_Genbank\_for\_inparanoid.fasta:1615

```
CQW49_RS18320       	100.00%		CQW49_18260         	100.00%
Bootstrap support for CQW49_RS18320 as seed ortholog is 100%.
Bootstrap support for CQW49_18260 as seed ortholog is 100%.
```

---

### Group of orthologs #156. Best score 1613 bits Score difference with first non-orthologous sequence - OB3b\_Refseq\_for\_inparanoid.fasta:1613 OB3b\_Genbank\_for\_inparanoid.fasta:1613

```
CQW49_RS14995       	100.00%		CQW49_14960         	100.00%
Bootstrap support for CQW49_RS14995 as seed ortholog is 100%.
Bootstrap support for CQW49_14960 as seed ortholog is 100%.
```

---

### Group of orthologs #157. Best score 1611 bits Score difference with first non-orthologous sequence - OB3b\_Refseq\_for\_inparanoid.fasta:1611 OB3b\_Genbank\_for\_inparanoid.fasta:1611

```
CQW49_RS11815       	100.00%		CQW49_11780         	100.00%
Bootstrap support for CQW49_RS11815 as seed ortholog is 100%.
Bootstrap support for CQW49_11780 as seed ortholog is 100%.
```

---

### Group of orthologs #158. Best score 1609 bits Score difference with first non-orthologous sequence - OB3b\_Refseq\_for\_inparanoid.fasta:1609 OB3b\_Genbank\_for\_inparanoid.fasta:1609

```
CQW49_RS12015       	100.00%		CQW49_11980         	100.00%
Bootstrap support for CQW49_RS12015 as seed ortholog is 100%.
Bootstrap support for CQW49_11980 as seed ortholog is 100%.
```

---

### Group of orthologs #159. Best score 1607 bits Score difference with first non-orthologous sequence - OB3b\_Refseq\_for\_inparanoid.fasta:1607 OB3b\_Genbank\_for\_inparanoid.fasta:1607

```
clpA                	100.00%		CQW49_20615         	100.00%
Bootstrap support for clpA as seed ortholog is 100%.
Bootstrap support for CQW49_20615 as seed ortholog is 100%.
```

---

### Group of orthologs #160. Best score 1595 bits Score difference with first non-orthologous sequence - OB3b\_Refseq\_for\_inparanoid.fasta:1595 OB3b\_Genbank\_for\_inparanoid.fasta:1076

```
CQW49_RS01940       	100.00%		CQW49_01935         	100.00%
Bootstrap support for CQW49_RS01940 as seed ortholog is 100%.
Bootstrap support for CQW49_01935 as seed ortholog is 100%.
```

---

### Group of orthologs #161. Best score 1591 bits Score difference with first non-orthologous sequence - OB3b\_Refseq\_for\_inparanoid.fasta:1591 OB3b\_Genbank\_for\_inparanoid.fasta:1591

```
gyrB                	100.00%		CQW49_03775         	100.00%
Bootstrap support for gyrB as seed ortholog is 100%.
Bootstrap support for CQW49_03775 as seed ortholog is 100%.
```

---

### Group of orthologs #162. Best score 1589 bits Score difference with first non-orthologous sequence - OB3b\_Refseq\_for\_inparanoid.fasta:1589 OB3b\_Genbank\_for\_inparanoid.fasta:1589

```
CQW49_RS02005       	100.00%		CQW49_02000         	100.00%
Bootstrap support for CQW49_RS02005 as seed ortholog is 100%.
Bootstrap support for CQW49_02000 as seed ortholog is 100%.
```

---

### Group of orthologs #163. Best score 1589 bits Score difference with first non-orthologous sequence - OB3b\_Refseq\_for\_inparanoid.fasta:1589 OB3b\_Genbank\_for\_inparanoid.fasta:1589

```
CQW49_RS02145       	100.00%		CQW49_02140         	100.00%
Bootstrap support for CQW49_RS02145 as seed ortholog is 100%.
Bootstrap support for CQW49_02140 as seed ortholog is 100%.
```

---

### Group of orthologs #164. Best score 1587 bits Score difference with first non-orthologous sequence - OB3b\_Refseq\_for\_inparanoid.fasta:1587 OB3b\_Genbank\_for\_inparanoid.fasta:1587

```
CQW49_RS12610       	100.00%		CQW49_12575         	100.00%
Bootstrap support for CQW49_RS12610 as seed ortholog is 100%.
Bootstrap support for CQW49_12575 as seed ortholog is 100%.
```

---

### Group of orthologs #165. Best score 1586 bits Score difference with first non-orthologous sequence - OB3b\_Refseq\_for\_inparanoid.fasta:1586 OB3b\_Genbank\_for\_inparanoid.fasta:1331

```
CQW49_RS07220       	100.00%		CQW49_07210         	100.00%
Bootstrap support for CQW49_RS07220 as seed ortholog is 100%.
Bootstrap support for CQW49_07210 as seed ortholog is 100%.
```

---

### Group of orthologs #166. Best score 1585 bits Score difference with first non-orthologous sequence - OB3b\_Refseq\_for\_inparanoid.fasta:1585 OB3b\_Genbank\_for\_inparanoid.fasta:1585

```
hrpB                	100.00%		CQW49_03190         	100.00%
Bootstrap support for hrpB as seed ortholog is 100%.
Bootstrap support for CQW49_03190 as seed ortholog is 100%.
```

---

### Group of orthologs #167. Best score 1582 bits Score difference with first non-orthologous sequence - OB3b\_Refseq\_for\_inparanoid.fasta:1582 OB3b\_Genbank\_for\_inparanoid.fasta:1582

```
CQW49_RS06195       	100.00%		CQW49_06185         	100.00%
Bootstrap support for CQW49_RS06195 as seed ortholog is 100%.
Bootstrap support for CQW49_06185 as seed ortholog is 100%.
```

---

### Group of orthologs #168. Best score 1582 bits Score difference with first non-orthologous sequence - OB3b\_Refseq\_for\_inparanoid.fasta:1582 OB3b\_Genbank\_for\_inparanoid.fasta:1582

```
CQW49_RS15785       	100.00%		CQW49_15750         	100.00%
Bootstrap support for CQW49_RS15785 as seed ortholog is 100%.
Bootstrap support for CQW49_15750 as seed ortholog is 100%.
```

---

### Group of orthologs #169. Best score 1570 bits Score difference with first non-orthologous sequence - OB3b\_Refseq\_for\_inparanoid.fasta:1570 OB3b\_Genbank\_for\_inparanoid.fasta:1323

```
CQW49_RS05120       	100.00%		CQW49_05115         	100.00%
Bootstrap support for CQW49_RS05120 as seed ortholog is 100%.
Bootstrap support for CQW49_05115 as seed ortholog is 100%.
```

---

### Group of orthologs #170. Best score 1570 bits Score difference with first non-orthologous sequence - OB3b\_Refseq\_for\_inparanoid.fasta:1570 OB3b\_Genbank\_for\_inparanoid.fasta:1570

```
CQW49_RS19200       	100.00%		CQW49_19140         	100.00%
Bootstrap support for CQW49_RS19200 as seed ortholog is 100%.
Bootstrap support for CQW49_19140 as seed ortholog is 100%.
```

---

### Group of orthologs #171. Best score 1564 bits Score difference with first non-orthologous sequence - OB3b\_Refseq\_for\_inparanoid.fasta:1564 OB3b\_Genbank\_for\_inparanoid.fasta:1478

```
CQW49_RS04470       	100.00%		CQW49_04465         	100.00%
Bootstrap support for CQW49_RS04470 as seed ortholog is 100%.
Bootstrap support for CQW49_04465 as seed ortholog is 100%.
```

---

### Group of orthologs #172. Best score 1561 bits Score difference with first non-orthologous sequence - OB3b\_Refseq\_for\_inparanoid.fasta:1561 OB3b\_Genbank\_for\_inparanoid.fasta:1561

```
CQW49_RS09720       	100.00%		CQW49_09700         	100.00%
Bootstrap support for CQW49_RS09720 as seed ortholog is 100%.
Bootstrap support for CQW49_09700 as seed ortholog is 100%.
```

---

### Group of orthologs #173. Best score 1557 bits Score difference with first non-orthologous sequence - OB3b\_Refseq\_for\_inparanoid.fasta:1557 OB3b\_Genbank\_for\_inparanoid.fasta:1557

```
CQW49_RS02425       	100.00%		CQW49_02415         	100.00%
Bootstrap support for CQW49_RS02425 as seed ortholog is 100%.
Bootstrap support for CQW49_02415 as seed ortholog is 100%.
```

---

### Group of orthologs #174. Best score 1557 bits Score difference with first non-orthologous sequence - OB3b\_Refseq\_for\_inparanoid.fasta:1557 OB3b\_Genbank\_for\_inparanoid.fasta:1557

```
CQW49_RS16140       	100.00%		CQW49_16105         	100.00%
Bootstrap support for CQW49_RS16140 as seed ortholog is 100%.
Bootstrap support for CQW49_16105 as seed ortholog is 100%.
```

---

### Group of orthologs #175. Best score 1554 bits Score difference with first non-orthologous sequence - OB3b\_Refseq\_for\_inparanoid.fasta:1554 OB3b\_Genbank\_for\_inparanoid.fasta:1554

```
CQW49_RS13805       	100.00%		CQW49_13770         	100.00%
Bootstrap support for CQW49_RS13805 as seed ortholog is 100%.
Bootstrap support for CQW49_13770 as seed ortholog is 100%.
```

---

### Group of orthologs #176. Best score 1548 bits Score difference with first non-orthologous sequence - OB3b\_Refseq\_for\_inparanoid.fasta:1548 OB3b\_Genbank\_for\_inparanoid.fasta:1114

```
CQW49_RS05765       	100.00%		CQW49_05755         	100.00%
Bootstrap support for CQW49_RS05765 as seed ortholog is 100%.
Bootstrap support for CQW49_05755 as seed ortholog is 100%.
```

---

### Group of orthologs #177. Best score 1547 bits Score difference with first non-orthologous sequence - OB3b\_Refseq\_for\_inparanoid.fasta:1547 OB3b\_Genbank\_for\_inparanoid.fasta:1547

```
CQW49_RS08515       	100.00%		CQW49_08505         	100.00%
Bootstrap support for CQW49_RS08515 as seed ortholog is 100%.
Bootstrap support for CQW49_08505 as seed ortholog is 100%.
```

---

### Group of orthologs #178. Best score 1545 bits Score difference with first non-orthologous sequence - OB3b\_Refseq\_for\_inparanoid.fasta:1177 OB3b\_Genbank\_for\_inparanoid.fasta:1545

```
CQW49_RS10975       	100.00%		CQW49_10945         	100.00%
Bootstrap support for CQW49_RS10975 as seed ortholog is 100%.
Bootstrap support for CQW49_10945 as seed ortholog is 100%.
```

---

### Group of orthologs #179. Best score 1543 bits Score difference with first non-orthologous sequence - OB3b\_Refseq\_for\_inparanoid.fasta:1543 OB3b\_Genbank\_for\_inparanoid.fasta:1186

```
CQW49_RS13595       	100.00%		CQW49_13560         	100.00%
Bootstrap support for CQW49_RS13595 as seed ortholog is 100%.
Bootstrap support for CQW49_13560 as seed ortholog is 100%.
```

---

### Group of orthologs #180. Best score 1542 bits Score difference with first non-orthologous sequence - OB3b\_Refseq\_for\_inparanoid.fasta:1542 OB3b\_Genbank\_for\_inparanoid.fasta:1135

```
CQW49_RS17045       	100.00%		CQW49_17000         	100.00%
Bootstrap support for CQW49_RS17045 as seed ortholog is 100%.
Bootstrap support for CQW49_17000 as seed ortholog is 100%.
```

---

### Group of orthologs #181. Best score 1532 bits Score difference with first non-orthologous sequence - OB3b\_Refseq\_for\_inparanoid.fasta:1532 OB3b\_Genbank\_for\_inparanoid.fasta:1532

```
hypF                	100.00%		CQW49_03570         	100.00%
Bootstrap support for hypF as seed ortholog is 100%.
Bootstrap support for CQW49_03570 as seed ortholog is 100%.
```

---

### Group of orthologs #182. Best score 1531 bits Score difference with first non-orthologous sequence - OB3b\_Refseq\_for\_inparanoid.fasta:1531 OB3b\_Genbank\_for\_inparanoid.fasta:1371

```
CQW49_RS14725       	100.00%		CQW49_14690         	100.00%
Bootstrap support for CQW49_RS14725 as seed ortholog is 100%.
Bootstrap support for CQW49_14690 as seed ortholog is 100%.
```

---

### Group of orthologs #183. Best score 1526 bits Score difference with first non-orthologous sequence - OB3b\_Refseq\_for\_inparanoid.fasta:1526 OB3b\_Genbank\_for\_inparanoid.fasta:1526

```
CQW49_RS07385       	100.00%		CQW49_07375         	100.00%
Bootstrap support for CQW49_RS07385 as seed ortholog is 100%.
Bootstrap support for CQW49_07375 as seed ortholog is 100%.
```

---

### Group of orthologs #184. Best score 1521 bits Score difference with first non-orthologous sequence - OB3b\_Refseq\_for\_inparanoid.fasta:1521 OB3b\_Genbank\_for\_inparanoid.fasta:1521

```
katG                	100.00%		CQW49_09835         	100.00%
Bootstrap support for katG as seed ortholog is 100%.
Bootstrap support for CQW49_09835 as seed ortholog is 100%.
```

---

### Group of orthologs #185. Best score 1519 bits Score difference with first non-orthologous sequence - OB3b\_Refseq\_for\_inparanoid.fasta:1519 OB3b\_Genbank\_for\_inparanoid.fasta:1136

```
CQW49_RS03265       	100.00%		CQW49_03255         	100.00%
Bootstrap support for CQW49_RS03265 as seed ortholog is 100%.
Bootstrap support for CQW49_03255 as seed ortholog is 100%.
```

---

### Group of orthologs #186. Best score 1518 bits Score difference with first non-orthologous sequence - OB3b\_Refseq\_for\_inparanoid.fasta:1518 OB3b\_Genbank\_for\_inparanoid.fasta:1518

```
CQW49_RS10895       	100.00%		CQW49_10865         	100.00%
Bootstrap support for CQW49_RS10895 as seed ortholog is 100%.
Bootstrap support for CQW49_10865 as seed ortholog is 100%.
```

---

### Group of orthologs #187. Best score 1515 bits Score difference with first non-orthologous sequence - OB3b\_Refseq\_for\_inparanoid.fasta:1515 OB3b\_Genbank\_for\_inparanoid.fasta:1133

```
CQW49_RS05625       	100.00%		CQW49_05615         	100.00%
Bootstrap support for CQW49_RS05625 as seed ortholog is 100%.
Bootstrap support for CQW49_05615 as seed ortholog is 100%.
```

---

### Group of orthologs #188. Best score 1513 bits Score difference with first non-orthologous sequence - OB3b\_Refseq\_for\_inparanoid.fasta:1513 OB3b\_Genbank\_for\_inparanoid.fasta:1513

```
CQW49_RS19865       	100.00%		CQW49_19810         	100.00%
Bootstrap support for CQW49_RS19865 as seed ortholog is 100%.
Bootstrap support for CQW49_19810 as seed ortholog is 100%.
```

---

### Group of orthologs #189. Best score 1510 bits Score difference with first non-orthologous sequence - OB3b\_Refseq\_for\_inparanoid.fasta:1510 OB3b\_Genbank\_for\_inparanoid.fasta:1510

```
CQW49_RS13920       	100.00%		CQW49_13885         	100.00%
Bootstrap support for CQW49_RS13920 as seed ortholog is 100%.
Bootstrap support for CQW49_13885 as seed ortholog is 100%.
```

---

### Group of orthologs #190. Best score 1505 bits Score difference with first non-orthologous sequence - OB3b\_Refseq\_for\_inparanoid.fasta:1505 OB3b\_Genbank\_for\_inparanoid.fasta:1505

```
CQW49_RS10595       	100.00%		CQW49_10565         	100.00%
Bootstrap support for CQW49_RS10595 as seed ortholog is 100%.
Bootstrap support for CQW49_10565 as seed ortholog is 100%.
```

---

### Group of orthologs #191. Best score 1505 bits Score difference with first non-orthologous sequence - OB3b\_Refseq\_for\_inparanoid.fasta:1505 OB3b\_Genbank\_for\_inparanoid.fasta:1505

```
CQW49_RS20910       	100.00%		CQW49_20835         	100.00%
Bootstrap support for CQW49_RS20910 as seed ortholog is 100%.
Bootstrap support for CQW49_20835 as seed ortholog is 100%.
```

---

### Group of orthologs #192. Best score 1502 bits Score difference with first non-orthologous sequence - OB3b\_Refseq\_for\_inparanoid.fasta:1502 OB3b\_Genbank\_for\_inparanoid.fasta:1502

```
CQW49_RS14620       	100.00%		CQW49_14585         	100.00%
Bootstrap support for CQW49_RS14620 as seed ortholog is 100%.
Bootstrap support for CQW49_14585 as seed ortholog is 100%.
```

---

### Group of orthologs #193. Best score 1502 bits Score difference with first non-orthologous sequence - OB3b\_Refseq\_for\_inparanoid.fasta:1502 OB3b\_Genbank\_for\_inparanoid.fasta:1324

```
CQW49_RS17695       	100.00%		CQW49_17640         	100.00%
Bootstrap support for CQW49_RS17695 as seed ortholog is 100%.
Bootstrap support for CQW49_17640 as seed ortholog is 100%.
```

---

### Group of orthologs #194. Best score 1499 bits Score difference with first non-orthologous sequence - OB3b\_Refseq\_for\_inparanoid.fasta:1499 OB3b\_Genbank\_for\_inparanoid.fasta:947

```
CQW49_RS06265       	100.00%		CQW49_06255         	100.00%
Bootstrap support for CQW49_RS06265 as seed ortholog is 100%.
Bootstrap support for CQW49_06255 as seed ortholog is 100%.
```

---

### Group of orthologs #195. Best score 1498 bits Score difference with first non-orthologous sequence - OB3b\_Refseq\_for\_inparanoid.fasta:1498 OB3b\_Genbank\_for\_inparanoid.fasta:1498

```
gspD                	100.00%		CQW49_00415         	100.00%
Bootstrap support for gspD as seed ortholog is 100%.
Bootstrap support for CQW49_00415 as seed ortholog is 100%.
```

---

### Group of orthologs #196. Best score 1498 bits Score difference with first non-orthologous sequence - OB3b\_Refseq\_for\_inparanoid.fasta:1498 OB3b\_Genbank\_for\_inparanoid.fasta:1169

```
CQW49_RS04325       	100.00%		CQW49_04315         	100.00%
Bootstrap support for CQW49_RS04325 as seed ortholog is 100%.
Bootstrap support for CQW49_04315 as seed ortholog is 100%.
```

---

### Group of orthologs #197. Best score 1497 bits Score difference with first non-orthologous sequence - OB3b\_Refseq\_for\_inparanoid.fasta:1497 OB3b\_Genbank\_for\_inparanoid.fasta:1497

```
CQW49_RS00830       	100.00%		CQW49_00830         	100.00%
Bootstrap support for CQW49_RS00830 as seed ortholog is 100%.
Bootstrap support for CQW49_00830 as seed ortholog is 100%.
```

---

### Group of orthologs #198. Best score 1496 bits Score difference with first non-orthologous sequence - OB3b\_Refseq\_for\_inparanoid.fasta:1496 OB3b\_Genbank\_for\_inparanoid.fasta:1496

```
CQW49_RS11030       	100.00%		CQW49_11000         	100.00%
Bootstrap support for CQW49_RS11030 as seed ortholog is 100%.
Bootstrap support for CQW49_11000 as seed ortholog is 100%.
```

---

### Group of orthologs #199. Best score 1492 bits Score difference with first non-orthologous sequence - OB3b\_Refseq\_for\_inparanoid.fasta:1492 OB3b\_Genbank\_for\_inparanoid.fasta:1492

```
CQW49_RS07060       	100.00%		CQW49_07050         	100.00%
Bootstrap support for CQW49_RS07060 as seed ortholog is 100%.
Bootstrap support for CQW49_07050 as seed ortholog is 100%.
```

---

### Group of orthologs #200. Best score 1492 bits Score difference with first non-orthologous sequence - OB3b\_Refseq\_for\_inparanoid.fasta:1492 OB3b\_Genbank\_for\_inparanoid.fasta:1492

```
CQW49_RS07875       	100.00%		CQW49_07865         	100.00%
Bootstrap support for CQW49_RS07875 as seed ortholog is 100%.
Bootstrap support for CQW49_07865 as seed ortholog is 100%.
```

---

### Group of orthologs #201. Best score 1489 bits Score difference with first non-orthologous sequence - OB3b\_Refseq\_for\_inparanoid.fasta:1489 OB3b\_Genbank\_for\_inparanoid.fasta:1152

```
CQW49_RS09285       	100.00%		CQW49_09265         	100.00%
Bootstrap support for CQW49_RS09285 as seed ortholog is 100%.
Bootstrap support for CQW49_09265 as seed ortholog is 100%.
```

---

### Group of orthologs #202. Best score 1486 bits Score difference with first non-orthologous sequence - OB3b\_Refseq\_for\_inparanoid.fasta:1486 OB3b\_Genbank\_for\_inparanoid.fasta:1486

```
rnr                 	100.00%		CQW49_02300         	100.00%
Bootstrap support for rnr as seed ortholog is 100%.
Bootstrap support for CQW49_02300 as seed ortholog is 100%.
```

---

### Group of orthologs #203. Best score 1485 bits Score difference with first non-orthologous sequence - OB3b\_Refseq\_for\_inparanoid.fasta:1485 OB3b\_Genbank\_for\_inparanoid.fasta:1485

```
CQW49_RS07665       	100.00%		CQW49_07655         	100.00%
Bootstrap support for CQW49_RS07665 as seed ortholog is 100%.
Bootstrap support for CQW49_07655 as seed ortholog is 100%.
```

---

### Group of orthologs #204. Best score 1485 bits Score difference with first non-orthologous sequence - OB3b\_Refseq\_for\_inparanoid.fasta:1485 OB3b\_Genbank\_for\_inparanoid.fasta:1485

```
CQW49_RS18035       	100.00%		CQW49_17975         	100.00%
Bootstrap support for CQW49_RS18035 as seed ortholog is 100%.
Bootstrap support for CQW49_17975 as seed ortholog is 100%.
```

---

### Group of orthologs #205. Best score 1483 bits Score difference with first non-orthologous sequence - OB3b\_Refseq\_for\_inparanoid.fasta:1483 OB3b\_Genbank\_for\_inparanoid.fasta:1483

```
CQW49_RS15510       	100.00%		CQW49_15475         	100.00%
Bootstrap support for CQW49_RS15510 as seed ortholog is 100%.
Bootstrap support for CQW49_15475 as seed ortholog is 100%.
```

---

### Group of orthologs #206. Best score 1482 bits Score difference with first non-orthologous sequence - OB3b\_Refseq\_for\_inparanoid.fasta:1482 OB3b\_Genbank\_for\_inparanoid.fasta:1171

```
CQW49_RS09135       	100.00%		CQW49_09115         	100.00%
Bootstrap support for CQW49_RS09135 as seed ortholog is 100%.
Bootstrap support for CQW49_09115 as seed ortholog is 100%.
```

---

### Group of orthologs #207. Best score 1480 bits Score difference with first non-orthologous sequence - OB3b\_Refseq\_for\_inparanoid.fasta:1480 OB3b\_Genbank\_for\_inparanoid.fasta:1480

```
CQW49_RS17090       	100.00%		CQW49_17045         	100.00%
Bootstrap support for CQW49_RS17090 as seed ortholog is 100%.
Bootstrap support for CQW49_17045 as seed ortholog is 100%.
```

---

### Group of orthologs #208. Best score 1480 bits Score difference with first non-orthologous sequence - OB3b\_Refseq\_for\_inparanoid.fasta:1480 OB3b\_Genbank\_for\_inparanoid.fasta:1480

```
CQW49_RS21200       	100.00%		CQW49_21125         	100.00%
Bootstrap support for CQW49_RS21200 as seed ortholog is 100%.
Bootstrap support for CQW49_21125 as seed ortholog is 100%.
```

---

### Group of orthologs #209. Best score 1478 bits Score difference with first non-orthologous sequence - OB3b\_Refseq\_for\_inparanoid.fasta:1478 OB3b\_Genbank\_for\_inparanoid.fasta:1478

```
CQW49_RS13275       	100.00%		CQW49_13240         	100.00%
Bootstrap support for CQW49_RS13275 as seed ortholog is 100%.
Bootstrap support for CQW49_13240 as seed ortholog is 100%.
```

---

### Group of orthologs #210. Best score 1477 bits Score difference with first non-orthologous sequence - OB3b\_Refseq\_for\_inparanoid.fasta:1477 OB3b\_Genbank\_for\_inparanoid.fasta:1376

```
CQW49_RS18975       	100.00%		CQW49_18915         	100.00%
Bootstrap support for CQW49_RS18975 as seed ortholog is 100%.
Bootstrap support for CQW49_18915 as seed ortholog is 100%.
```

---

### Group of orthologs #211. Best score 1475 bits Score difference with first non-orthologous sequence - OB3b\_Refseq\_for\_inparanoid.fasta:1475 OB3b\_Genbank\_for\_inparanoid.fasta:1279

```
CQW49_RS14935       	100.00%		CQW49_14900         	100.00%
Bootstrap support for CQW49_RS14935 as seed ortholog is 100%.
Bootstrap support for CQW49_14900 as seed ortholog is 100%.
```

---

### Group of orthologs #212. Best score 1474 bits Score difference with first non-orthologous sequence - OB3b\_Refseq\_for\_inparanoid.fasta:1474 OB3b\_Genbank\_for\_inparanoid.fasta:1474

```
CQW49_RS02860       	100.00%		CQW49_02850         	100.00%
Bootstrap support for CQW49_RS02860 as seed ortholog is 100%.
Bootstrap support for CQW49_02850 as seed ortholog is 100%.
```

---

### Group of orthologs #213. Best score 1474 bits Score difference with first non-orthologous sequence - OB3b\_Refseq\_for\_inparanoid.fasta:1474 OB3b\_Genbank\_for\_inparanoid.fasta:1474

```
CQW49_RS16300       	100.00%		CQW49_16265         	100.00%
Bootstrap support for CQW49_RS16300 as seed ortholog is 100%.
Bootstrap support for CQW49_16265 as seed ortholog is 100%.
```

---

### Group of orthologs #214. Best score 1472 bits Score difference with first non-orthologous sequence - OB3b\_Refseq\_for\_inparanoid.fasta:1472 OB3b\_Genbank\_for\_inparanoid.fasta:1472

```
CQW49_RS05135       	100.00%		CQW49_05130         	100.00%
Bootstrap support for CQW49_RS05135 as seed ortholog is 100%.
Bootstrap support for CQW49_05130 as seed ortholog is 100%.
```

---

### Group of orthologs #215. Best score 1471 bits Score difference with first non-orthologous sequence - OB3b\_Refseq\_for\_inparanoid.fasta:1471 OB3b\_Genbank\_for\_inparanoid.fasta:1471

```
CQW49_RS16335       	100.00%		CQW49_16300         	100.00%
Bootstrap support for CQW49_RS16335 as seed ortholog is 100%.
Bootstrap support for CQW49_16300 as seed ortholog is 100%.
```

---

### Group of orthologs #216. Best score 1470 bits Score difference with first non-orthologous sequence - OB3b\_Refseq\_for\_inparanoid.fasta:1470 OB3b\_Genbank\_for\_inparanoid.fasta:1470

```
CQW49_RS10940       	100.00%		CQW49_10910         	100.00%
Bootstrap support for CQW49_RS10940 as seed ortholog is 100%.
Bootstrap support for CQW49_10910 as seed ortholog is 100%.
```

---

### Group of orthologs #217. Best score 1468 bits Score difference with first non-orthologous sequence - OB3b\_Refseq\_for\_inparanoid.fasta:1468 OB3b\_Genbank\_for\_inparanoid.fasta:1041

```
CQW49_RS05900       	100.00%		CQW49_05890         	100.00%
Bootstrap support for CQW49_RS05900 as seed ortholog is 100%.
Bootstrap support for CQW49_05890 as seed ortholog is 100%.
```

---

### Group of orthologs #218. Best score 1468 bits Score difference with first non-orthologous sequence - OB3b\_Refseq\_for\_inparanoid.fasta:1468 OB3b\_Genbank\_for\_inparanoid.fasta:1468

```
parC                	100.00%		CQW49_09500         	100.00%
Bootstrap support for parC as seed ortholog is 100%.
Bootstrap support for CQW49_09500 as seed ortholog is 100%.
```

---

### Group of orthologs #219. Best score 1468 bits Score difference with first non-orthologous sequence - OB3b\_Refseq\_for\_inparanoid.fasta:1468 OB3b\_Genbank\_for\_inparanoid.fasta:1468

```
CQW49_RS16340       	100.00%		CQW49_16305         	100.00%
Bootstrap support for CQW49_RS16340 as seed ortholog is 100%.
Bootstrap support for CQW49_16305 as seed ortholog is 100%.
```

---

### Group of orthologs #220. Best score 1464 bits Score difference with first non-orthologous sequence - OB3b\_Refseq\_for\_inparanoid.fasta:1464 OB3b\_Genbank\_for\_inparanoid.fasta:1464

```
CQW49_RS19585       	100.00%		CQW49_19530         	100.00%
Bootstrap support for CQW49_RS19585 as seed ortholog is 100%.
Bootstrap support for CQW49_19530 as seed ortholog is 100%.
```

---

### Group of orthologs #221. Best score 1463 bits Score difference with first non-orthologous sequence - OB3b\_Refseq\_for\_inparanoid.fasta:1463 OB3b\_Genbank\_for\_inparanoid.fasta:1463

```
CQW49_RS03345       	100.00%		CQW49_03335         	100.00%
Bootstrap support for CQW49_RS03345 as seed ortholog is 100%.
Bootstrap support for CQW49_03335 as seed ortholog is 100%.
```

---

### Group of orthologs #222. Best score 1463 bits Score difference with first non-orthologous sequence - OB3b\_Refseq\_for\_inparanoid.fasta:1463 OB3b\_Genbank\_for\_inparanoid.fasta:1463

```
CQW49_RS18215       	100.00%		CQW49_18155         	100.00%
Bootstrap support for CQW49_RS18215 as seed ortholog is 100%.
Bootstrap support for CQW49_18155 as seed ortholog is 100%.
```

---

### Group of orthologs #223. Best score 1461 bits Score difference with first non-orthologous sequence - OB3b\_Refseq\_for\_inparanoid.fasta:1461 OB3b\_Genbank\_for\_inparanoid.fasta:526

```
CQW49_RS02335       	100.00%		CQW49_02330         	100.00%
Bootstrap support for CQW49_RS02335 as seed ortholog is 100%.
Bootstrap support for CQW49_02330 as seed ortholog is 100%.
```

---

### Group of orthologs #224. Best score 1458 bits Score difference with first non-orthologous sequence - OB3b\_Refseq\_for\_inparanoid.fasta:1458 OB3b\_Genbank\_for\_inparanoid.fasta:1458

```
CQW49_RS05680       	100.00%		CQW49_05670         	100.00%
Bootstrap support for CQW49_RS05680 as seed ortholog is 100%.
Bootstrap support for CQW49_05670 as seed ortholog is 100%.
```

---

### Group of orthologs #225. Best score 1458 bits Score difference with first non-orthologous sequence - OB3b\_Refseq\_for\_inparanoid.fasta:1458 OB3b\_Genbank\_for\_inparanoid.fasta:1458

```
CQW49_RS09385       	100.00%		CQW49_09365         	100.00%
Bootstrap support for CQW49_RS09385 as seed ortholog is 100%.
Bootstrap support for CQW49_09365 as seed ortholog is 100%.
```

---

### Group of orthologs #226. Best score 1458 bits Score difference with first non-orthologous sequence - OB3b\_Refseq\_for\_inparanoid.fasta:1458 OB3b\_Genbank\_for\_inparanoid.fasta:1458

```
CQW49_RS13460       	100.00%		CQW49_13425         	100.00%
Bootstrap support for CQW49_RS13460 as seed ortholog is 100%.
Bootstrap support for CQW49_13425 as seed ortholog is 100%.
```

---

### Group of orthologs #227. Best score 1457 bits Score difference with first non-orthologous sequence - OB3b\_Refseq\_for\_inparanoid.fasta:1457 OB3b\_Genbank\_for\_inparanoid.fasta:1457

```
ptsP                	100.00%		CQW49_01035         	100.00%
Bootstrap support for ptsP as seed ortholog is 100%.
Bootstrap support for CQW49_01035 as seed ortholog is 100%.
```

---

### Group of orthologs #228. Best score 1456 bits Score difference with first non-orthologous sequence - OB3b\_Refseq\_for\_inparanoid.fasta:1456 OB3b\_Genbank\_for\_inparanoid.fasta:1456

```
CQW49_RS06330       	100.00%		CQW49_06320         	100.00%
Bootstrap support for CQW49_RS06330 as seed ortholog is 100%.
Bootstrap support for CQW49_06320 as seed ortholog is 100%.
```

---

### Group of orthologs #229. Best score 1454 bits Score difference with first non-orthologous sequence - OB3b\_Refseq\_for\_inparanoid.fasta:1454 OB3b\_Genbank\_for\_inparanoid.fasta:1454

```
CQW49_RS11955       	100.00%		CQW49_11920         	100.00%
Bootstrap support for CQW49_RS11955 as seed ortholog is 100%.
Bootstrap support for CQW49_11920 as seed ortholog is 100%.
```

---

### Group of orthologs #230. Best score 1451 bits Score difference with first non-orthologous sequence - OB3b\_Refseq\_for\_inparanoid.fasta:1451 OB3b\_Genbank\_for\_inparanoid.fasta:1451

```
CQW49_RS18210       	100.00%		CQW49_18150         	100.00%
Bootstrap support for CQW49_RS18210 as seed ortholog is 100%.
Bootstrap support for CQW49_18150 as seed ortholog is 100%.
```

---

### Group of orthologs #231. Best score 1450 bits Score difference with first non-orthologous sequence - OB3b\_Refseq\_for\_inparanoid.fasta:1450 OB3b\_Genbank\_for\_inparanoid.fasta:1450

```
CQW49_RS04795       	100.00%		CQW49_04785         	100.00%
Bootstrap support for CQW49_RS04795 as seed ortholog is 100%.
Bootstrap support for CQW49_04785 as seed ortholog is 100%.
```

---

### Group of orthologs #232. Best score 1448 bits Score difference with first non-orthologous sequence - OB3b\_Refseq\_for\_inparanoid.fasta:1448 OB3b\_Genbank\_for\_inparanoid.fasta:1317

```
CQW49_RS07560       	100.00%		CQW49_07550         	100.00%
Bootstrap support for CQW49_RS07560 as seed ortholog is 100%.
Bootstrap support for CQW49_07550 as seed ortholog is 100%.
```

---

### Group of orthologs #233. Best score 1445 bits Score difference with first non-orthologous sequence - OB3b\_Refseq\_for\_inparanoid.fasta:1445 OB3b\_Genbank\_for\_inparanoid.fasta:1445

```
CQW49_RS04000       	100.00%		CQW49_03990         	100.00%
Bootstrap support for CQW49_RS04000 as seed ortholog is 100%.
Bootstrap support for CQW49_03990 as seed ortholog is 100%.
```

---

### Group of orthologs #234. Best score 1445 bits Score difference with first non-orthologous sequence - OB3b\_Refseq\_for\_inparanoid.fasta:1445 OB3b\_Genbank\_for\_inparanoid.fasta:1445

```
CQW49_RS04095       	100.00%		CQW49_04085         	100.00%
Bootstrap support for CQW49_RS04095 as seed ortholog is 100%.
Bootstrap support for CQW49_04085 as seed ortholog is 100%.
```

---

### Group of orthologs #235. Best score 1445 bits Score difference with first non-orthologous sequence - OB3b\_Refseq\_for\_inparanoid.fasta:1445 OB3b\_Genbank\_for\_inparanoid.fasta:1445

```
CQW49_RS17935       	100.00%		CQW49_17880         	100.00%
Bootstrap support for CQW49_RS17935 as seed ortholog is 100%.
Bootstrap support for CQW49_17880 as seed ortholog is 100%.
```

---

### Group of orthologs #236. Best score 1443 bits Score difference with first non-orthologous sequence - OB3b\_Refseq\_for\_inparanoid.fasta:1443 OB3b\_Genbank\_for\_inparanoid.fasta:1443

```
CQW49_RS04730       	100.00%		CQW49_04720         	100.00%
Bootstrap support for CQW49_RS04730 as seed ortholog is 100%.
Bootstrap support for CQW49_04720 as seed ortholog is 100%.
```

---

### Group of orthologs #237. Best score 1443 bits Score difference with first non-orthologous sequence - OB3b\_Refseq\_for\_inparanoid.fasta:1443 OB3b\_Genbank\_for\_inparanoid.fasta:1443

```
cas5u6u             	100.00%		CQW49_05815         	100.00%
Bootstrap support for cas5u6u as seed ortholog is 100%.
Bootstrap support for CQW49_05815 as seed ortholog is 100%.
```

---

### Group of orthologs #238. Best score 1442 bits Score difference with first non-orthologous sequence - OB3b\_Refseq\_for\_inparanoid.fasta:1442 OB3b\_Genbank\_for\_inparanoid.fasta:1442

```
CQW49_RS20480       	100.00%		CQW49_20410         	100.00%
Bootstrap support for CQW49_RS20480 as seed ortholog is 100%.
Bootstrap support for CQW49_20410 as seed ortholog is 100%.
```

---

### Group of orthologs #239. Best score 1441 bits Score difference with first non-orthologous sequence - OB3b\_Refseq\_for\_inparanoid.fasta:1441 OB3b\_Genbank\_for\_inparanoid.fasta:1441

```
CQW49_RS03830       	100.00%		CQW49_03820         	100.00%
Bootstrap support for CQW49_RS03830 as seed ortholog is 100%.
Bootstrap support for CQW49_03820 as seed ortholog is 100%.
```

---

### Group of orthologs #240. Best score 1439 bits Score difference with first non-orthologous sequence - OB3b\_Refseq\_for\_inparanoid.fasta:1439 OB3b\_Genbank\_for\_inparanoid.fasta:1439

```
CQW49_RS15535       	100.00%		CQW49_15500         	100.00%
Bootstrap support for CQW49_RS15535 as seed ortholog is 100%.
Bootstrap support for CQW49_15500 as seed ortholog is 100%.
```

---

### Group of orthologs #241. Best score 1437 bits Score difference with first non-orthologous sequence - OB3b\_Refseq\_for\_inparanoid.fasta:1437 OB3b\_Genbank\_for\_inparanoid.fasta:1437

```
CQW49_RS17270       	100.00%		CQW49_17225         	100.00%
Bootstrap support for CQW49_RS17270 as seed ortholog is 100%.
Bootstrap support for CQW49_17225 as seed ortholog is 100%.
```

---

### Group of orthologs #242. Best score 1434 bits Score difference with first non-orthologous sequence - OB3b\_Refseq\_for\_inparanoid.fasta:1434 OB3b\_Genbank\_for\_inparanoid.fasta:1434

```
CQW49_RS19700       	100.00%		CQW49_19645         	100.00%
Bootstrap support for CQW49_RS19700 as seed ortholog is 100%.
Bootstrap support for CQW49_19645 as seed ortholog is 100%.
```

---

### Group of orthologs #243. Best score 1432 bits Score difference with first non-orthologous sequence - OB3b\_Refseq\_for\_inparanoid.fasta:1432 OB3b\_Genbank\_for\_inparanoid.fasta:1432

```
CQW49_RS06720       	100.00%		CQW49_06710         	100.00%
Bootstrap support for CQW49_RS06720 as seed ortholog is 100%.
Bootstrap support for CQW49_06710 as seed ortholog is 100%.
```

---

### Group of orthologs #244. Best score 1432 bits Score difference with first non-orthologous sequence - OB3b\_Refseq\_for\_inparanoid.fasta:1432 OB3b\_Genbank\_for\_inparanoid.fasta:1432

```
CQW49_RS21160       	100.00%		CQW49_21085         	100.00%
Bootstrap support for CQW49_RS21160 as seed ortholog is 100%.
Bootstrap support for CQW49_21085 as seed ortholog is 100%.
```

---

### Group of orthologs #245. Best score 1426 bits Score difference with first non-orthologous sequence - OB3b\_Refseq\_for\_inparanoid.fasta:1426 OB3b\_Genbank\_for\_inparanoid.fasta:1363

```
CQW49_RS01650       	100.00%		CQW49_01645         	100.00%
Bootstrap support for CQW49_RS01650 as seed ortholog is 100%.
Bootstrap support for CQW49_01645 as seed ortholog is 100%.
```

---

### Group of orthologs #246. Best score 1426 bits Score difference with first non-orthologous sequence - OB3b\_Refseq\_for\_inparanoid.fasta:1426 OB3b\_Genbank\_for\_inparanoid.fasta:1426

```
CQW49_RS09220       	100.00%		CQW49_09200         	100.00%
Bootstrap support for CQW49_RS09220 as seed ortholog is 100%.
Bootstrap support for CQW49_09200 as seed ortholog is 100%.
```

---

### Group of orthologs #247. Best score 1424 bits Score difference with first non-orthologous sequence - OB3b\_Refseq\_for\_inparanoid.fasta:1424 OB3b\_Genbank\_for\_inparanoid.fasta:1424

```
CQW49_RS03695       	100.00%		CQW49_03685         	100.00%
Bootstrap support for CQW49_RS03695 as seed ortholog is 100%.
Bootstrap support for CQW49_03685 as seed ortholog is 100%.
```

---

### Group of orthologs #248. Best score 1421 bits Score difference with first non-orthologous sequence - OB3b\_Refseq\_for\_inparanoid.fasta:1421 OB3b\_Genbank\_for\_inparanoid.fasta:1421

```
CQW49_RS14870       	100.00%		CQW49_14835         	100.00%
Bootstrap support for CQW49_RS14870 as seed ortholog is 100%.
Bootstrap support for CQW49_14835 as seed ortholog is 100%.
```

---

### Group of orthologs #249. Best score 1414 bits Score difference with first non-orthologous sequence - OB3b\_Refseq\_for\_inparanoid.fasta:1414 OB3b\_Genbank\_for\_inparanoid.fasta:1414

```
CQW49_RS13430       	100.00%		CQW49_13395         	100.00%
Bootstrap support for CQW49_RS13430 as seed ortholog is 100%.
Bootstrap support for CQW49_13395 as seed ortholog is 100%.
```

---

### Group of orthologs #250. Best score 1411 bits Score difference with first non-orthologous sequence - OB3b\_Refseq\_for\_inparanoid.fasta:1411 OB3b\_Genbank\_for\_inparanoid.fasta:1411

```
CQW49_RS04685       	100.00%		CQW49_04675         	100.00%
Bootstrap support for CQW49_RS04685 as seed ortholog is 100%.
Bootstrap support for CQW49_04675 as seed ortholog is 100%.
```

---

### Group of orthologs #251. Best score 1411 bits Score difference with first non-orthologous sequence - OB3b\_Refseq\_for\_inparanoid.fasta:1411 OB3b\_Genbank\_for\_inparanoid.fasta:1411

```
CQW49_RS07815       	100.00%		CQW49_07805         	100.00%
Bootstrap support for CQW49_RS07815 as seed ortholog is 100%.
Bootstrap support for CQW49_07805 as seed ortholog is 100%.
```

---

### Group of orthologs #252. Best score 1407 bits Score difference with first non-orthologous sequence - OB3b\_Refseq\_for\_inparanoid.fasta:1407 OB3b\_Genbank\_for\_inparanoid.fasta:1407

```
CQW49_RS12585       	100.00%		CQW49_12550         	100.00%
Bootstrap support for CQW49_RS12585 as seed ortholog is 100%.
Bootstrap support for CQW49_12550 as seed ortholog is 100%.
```

---

### Group of orthologs #253. Best score 1406 bits Score difference with first non-orthologous sequence - OB3b\_Refseq\_for\_inparanoid.fasta:1406 OB3b\_Genbank\_for\_inparanoid.fasta:513

```
CQW49_RS13815       	100.00%		CQW49_13780         	100.00%
Bootstrap support for CQW49_RS13815 as seed ortholog is 100%.
Bootstrap support for CQW49_13780 as seed ortholog is 100%.
```

---

### Group of orthologs #254. Best score 1403 bits Score difference with first non-orthologous sequence - OB3b\_Refseq\_for\_inparanoid.fasta:1403 OB3b\_Genbank\_for\_inparanoid.fasta:1403

```
CQW49_RS07885       	100.00%		CQW49_07875         	100.00%
Bootstrap support for CQW49_RS07885 as seed ortholog is 100%.
Bootstrap support for CQW49_07875 as seed ortholog is 100%.
```

---

### Group of orthologs #255. Best score 1400 bits Score difference with first non-orthologous sequence - OB3b\_Refseq\_for\_inparanoid.fasta:1400 OB3b\_Genbank\_for\_inparanoid.fasta:1400

```
hppA                	100.00%		CQW49_19495         	100.00%
Bootstrap support for hppA as seed ortholog is 100%.
Bootstrap support for CQW49_19495 as seed ortholog is 100%.
```

---

### Group of orthologs #256. Best score 1397 bits Score difference with first non-orthologous sequence - OB3b\_Refseq\_for\_inparanoid.fasta:1397 OB3b\_Genbank\_for\_inparanoid.fasta:1397

```
CQW49_RS01055       	100.00%		CQW49_01055         	100.00%
Bootstrap support for CQW49_RS01055 as seed ortholog is 100%.
Bootstrap support for CQW49_01055 as seed ortholog is 100%.
```

---

### Group of orthologs #257. Best score 1397 bits Score difference with first non-orthologous sequence - OB3b\_Refseq\_for\_inparanoid.fasta:1397 OB3b\_Genbank\_for\_inparanoid.fasta:1397

```
CQW49_RS12700       	100.00%		CQW49_12665         	100.00%
Bootstrap support for CQW49_RS12700 as seed ortholog is 100%.
Bootstrap support for CQW49_12665 as seed ortholog is 100%.
```

---

### Group of orthologs #258. Best score 1391 bits Score difference with first non-orthologous sequence - OB3b\_Refseq\_for\_inparanoid.fasta:1391 OB3b\_Genbank\_for\_inparanoid.fasta:741

```
CQW49_RS14555       	100.00%		CQW49_14520         	100.00%
Bootstrap support for CQW49_RS14555 as seed ortholog is 100%.
Bootstrap support for CQW49_14520 as seed ortholog is 100%.
```

---

### Group of orthologs #259. Best score 1391 bits Score difference with first non-orthologous sequence - OB3b\_Refseq\_for\_inparanoid.fasta:1391 OB3b\_Genbank\_for\_inparanoid.fasta:1391

```
CQW49_RS21230       	100.00%		CQW49_21155         	100.00%
Bootstrap support for CQW49_RS21230 as seed ortholog is 100%.
Bootstrap support for CQW49_21155 as seed ortholog is 100%.
```

---

### Group of orthologs #260. Best score 1391 bits Score difference with first non-orthologous sequence - OB3b\_Refseq\_for\_inparanoid.fasta:1391 OB3b\_Genbank\_for\_inparanoid.fasta:1242

```
CQW49_RS21265       	100.00%		CQW49_21190         	100.00%
Bootstrap support for CQW49_RS21265 as seed ortholog is 100%.
Bootstrap support for CQW49_21190 as seed ortholog is 100%.
```

---

### Group of orthologs #261. Best score 1390 bits Score difference with first non-orthologous sequence - OB3b\_Refseq\_for\_inparanoid.fasta:1390 OB3b\_Genbank\_for\_inparanoid.fasta:1390

```
CQW49_RS15870       	100.00%		CQW49_15835         	100.00%
Bootstrap support for CQW49_RS15870 as seed ortholog is 100%.
Bootstrap support for CQW49_15835 as seed ortholog is 100%.
```

---

### Group of orthologs #262. Best score 1386 bits Score difference with first non-orthologous sequence - OB3b\_Refseq\_for\_inparanoid.fasta:1386 OB3b\_Genbank\_for\_inparanoid.fasta:1386

```
CQW49_RS07350       	100.00%		CQW49_07340         	100.00%
Bootstrap support for CQW49_RS07350 as seed ortholog is 100%.
Bootstrap support for CQW49_07340 as seed ortholog is 100%.
```

---

### Group of orthologs #263. Best score 1384 bits Score difference with first non-orthologous sequence - OB3b\_Refseq\_for\_inparanoid.fasta:1384 OB3b\_Genbank\_for\_inparanoid.fasta:1384

```
CQW49_RS03945       	100.00%		CQW49_03935         	100.00%
Bootstrap support for CQW49_RS03945 as seed ortholog is 100%.
Bootstrap support for CQW49_03935 as seed ortholog is 100%.
```

---

### Group of orthologs #264. Best score 1383 bits Score difference with first non-orthologous sequence - OB3b\_Refseq\_for\_inparanoid.fasta:1383 OB3b\_Genbank\_for\_inparanoid.fasta:1383

```
CQW49_RS04985       	100.00%		CQW49_04980         	100.00%
Bootstrap support for CQW49_RS04985 as seed ortholog is 100%.
Bootstrap support for CQW49_04980 as seed ortholog is 100%.
```

---

### Group of orthologs #265. Best score 1383 bits Score difference with first non-orthologous sequence - OB3b\_Refseq\_for\_inparanoid.fasta:1383 OB3b\_Genbank\_for\_inparanoid.fasta:587

```
CQW49_RS15255       	100.00%		CQW49_15220         	100.00%
Bootstrap support for CQW49_RS15255 as seed ortholog is 100%.
Bootstrap support for CQW49_15220 as seed ortholog is 100%.
```

---

### Group of orthologs #266. Best score 1380 bits Score difference with first non-orthologous sequence - OB3b\_Refseq\_for\_inparanoid.fasta:1380 OB3b\_Genbank\_for\_inparanoid.fasta:1380

```
CQW49_RS05300       	100.00%		CQW49_05295         	100.00%
Bootstrap support for CQW49_RS05300 as seed ortholog is 100%.
Bootstrap support for CQW49_05295 as seed ortholog is 100%.
```

---

### Group of orthologs #267. Best score 1380 bits Score difference with first non-orthologous sequence - OB3b\_Refseq\_for\_inparanoid.fasta:1380 OB3b\_Genbank\_for\_inparanoid.fasta:1380

```
pnp                 	100.00%		CQW49_21045         	100.00%
Bootstrap support for pnp as seed ortholog is 100%.
Bootstrap support for CQW49_21045 as seed ortholog is 100%.
```

---

### Group of orthologs #268. Best score 1378 bits Score difference with first non-orthologous sequence - OB3b\_Refseq\_for\_inparanoid.fasta:1378 OB3b\_Genbank\_for\_inparanoid.fasta:991

```
CQW49_RS05700       	100.00%		CQW49_05690         	100.00%
Bootstrap support for CQW49_RS05700 as seed ortholog is 100%.
Bootstrap support for CQW49_05690 as seed ortholog is 100%.
```

---

### Group of orthologs #269. Best score 1375 bits Score difference with first non-orthologous sequence - OB3b\_Refseq\_for\_inparanoid.fasta:1375 OB3b\_Genbank\_for\_inparanoid.fasta:1375

```
fusA                	100.00%		CQW49_10510         	100.00%
Bootstrap support for fusA as seed ortholog is 100%.
Bootstrap support for CQW49_10510 as seed ortholog is 100%.
```

---

### Group of orthologs #270. Best score 1373 bits Score difference with first non-orthologous sequence - OB3b\_Refseq\_for\_inparanoid.fasta:1373 OB3b\_Genbank\_for\_inparanoid.fasta:561

```
CQW49_RS14005       	100.00%		CQW49_13970         	100.00%
Bootstrap support for CQW49_RS14005 as seed ortholog is 100%.
Bootstrap support for CQW49_13970 as seed ortholog is 100%.
```

---

### Group of orthologs #271. Best score 1372 bits Score difference with first non-orthologous sequence - OB3b\_Refseq\_for\_inparanoid.fasta:1372 OB3b\_Genbank\_for\_inparanoid.fasta:1372

```
CQW49_RS04920       	100.00%		CQW49_04915         	100.00%
Bootstrap support for CQW49_RS04920 as seed ortholog is 100%.
Bootstrap support for CQW49_04915 as seed ortholog is 100%.
```

---

### Group of orthologs #272. Best score 1369 bits Score difference with first non-orthologous sequence - OB3b\_Refseq\_for\_inparanoid.fasta:1369 OB3b\_Genbank\_for\_inparanoid.fasta:1369

```
CQW49_RS05325       	100.00%		CQW49_05320         	100.00%
Bootstrap support for CQW49_RS05325 as seed ortholog is 100%.
Bootstrap support for CQW49_05320 as seed ortholog is 100%.
```

---

### Group of orthologs #273. Best score 1368 bits Score difference with first non-orthologous sequence - OB3b\_Refseq\_for\_inparanoid.fasta:1368 OB3b\_Genbank\_for\_inparanoid.fasta:1368

```
CQW49_RS03195       	100.00%		CQW49_03185         	100.00%
Bootstrap support for CQW49_RS03195 as seed ortholog is 100%.
Bootstrap support for CQW49_03185 as seed ortholog is 100%.
```

---

### Group of orthologs #274. Best score 1367 bits Score difference with first non-orthologous sequence - OB3b\_Refseq\_for\_inparanoid.fasta:1367 OB3b\_Genbank\_for\_inparanoid.fasta:1367

```
CQW49_RS03635       	100.00%		CQW49_03625         	100.00%
Bootstrap support for CQW49_RS03635 as seed ortholog is 100%.
Bootstrap support for CQW49_03625 as seed ortholog is 100%.
```

---

### Group of orthologs #275. Best score 1366 bits Score difference with first non-orthologous sequence - OB3b\_Refseq\_for\_inparanoid.fasta:1366 OB3b\_Genbank\_for\_inparanoid.fasta:1190

```
CQW49_RS00010       	100.00%		CQW49_00010         	100.00%
Bootstrap support for CQW49_RS00010 as seed ortholog is 100%.
Bootstrap support for CQW49_00010 as seed ortholog is 100%.
```

---

### Group of orthologs #276. Best score 1363 bits Score difference with first non-orthologous sequence - OB3b\_Refseq\_for\_inparanoid.fasta:1363 OB3b\_Genbank\_for\_inparanoid.fasta:1363

```
CQW49_RS20170       	100.00%		CQW49_20105         	100.00%
Bootstrap support for CQW49_RS20170 as seed ortholog is 100%.
Bootstrap support for CQW49_20105 as seed ortholog is 100%.
```

---

### Group of orthologs #277. Best score 1360 bits Score difference with first non-orthologous sequence - OB3b\_Refseq\_for\_inparanoid.fasta:1360 OB3b\_Genbank\_for\_inparanoid.fasta:1249

```
CQW49_RS16865       	100.00%		CQW49_16820         	100.00%
Bootstrap support for CQW49_RS16865 as seed ortholog is 100%.
Bootstrap support for CQW49_16820 as seed ortholog is 100%.
```

---

### Group of orthologs #278. Best score 1357 bits Score difference with first non-orthologous sequence - OB3b\_Refseq\_for\_inparanoid.fasta:1357 OB3b\_Genbank\_for\_inparanoid.fasta:1357

```
CQW49_RS09775       	100.00%		CQW49_09755         	100.00%
Bootstrap support for CQW49_RS09775 as seed ortholog is 100%.
Bootstrap support for CQW49_09755 as seed ortholog is 100%.
```

---

### Group of orthologs #279. Best score 1355 bits Score difference with first non-orthologous sequence - OB3b\_Refseq\_for\_inparanoid.fasta:1355 OB3b\_Genbank\_for\_inparanoid.fasta:1355

```
CQW49_RS04175       	100.00%		CQW49_04165         	100.00%
Bootstrap support for CQW49_RS04175 as seed ortholog is 100%.
Bootstrap support for CQW49_04165 as seed ortholog is 100%.
```

---

### Group of orthologs #280. Best score 1352 bits Score difference with first non-orthologous sequence - OB3b\_Refseq\_for\_inparanoid.fasta:1352 OB3b\_Genbank\_for\_inparanoid.fasta:1352

```
CQW49_RS01535       	100.00%		CQW49_01530         	100.00%
Bootstrap support for CQW49_RS01535 as seed ortholog is 100%.
Bootstrap support for CQW49_01530 as seed ortholog is 100%.
```

---

### Group of orthologs #281. Best score 1350 bits Score difference with first non-orthologous sequence - OB3b\_Refseq\_for\_inparanoid.fasta:1350 OB3b\_Genbank\_for\_inparanoid.fasta:1350

```
CQW49_RS03950       	100.00%		CQW49_03940         	100.00%
Bootstrap support for CQW49_RS03950 as seed ortholog is 100%.
Bootstrap support for CQW49_03940 as seed ortholog is 100%.
```

---

### Group of orthologs #282. Best score 1349 bits Score difference with first non-orthologous sequence - OB3b\_Refseq\_for\_inparanoid.fasta:1349 OB3b\_Genbank\_for\_inparanoid.fasta:1349

```
CQW49_RS00090       	100.00%		CQW49_00090         	100.00%
Bootstrap support for CQW49_RS00090 as seed ortholog is 100%.
Bootstrap support for CQW49_00090 as seed ortholog is 100%.
```

---

### Group of orthologs #283. Best score 1349 bits Score difference with first non-orthologous sequence - OB3b\_Refseq\_for\_inparanoid.fasta:1349 OB3b\_Genbank\_for\_inparanoid.fasta:1349

```
CQW49_RS19130       	100.00%		CQW49_19070         	100.00%
Bootstrap support for CQW49_RS19130 as seed ortholog is 100%.
Bootstrap support for CQW49_19070 as seed ortholog is 100%.
```

---

### Group of orthologs #284. Best score 1346 bits Score difference with first non-orthologous sequence - OB3b\_Refseq\_for\_inparanoid.fasta:1346 OB3b\_Genbank\_for\_inparanoid.fasta:1346

```
CQW49_RS00020       	100.00%		CQW49_00020         	100.00%
Bootstrap support for CQW49_RS00020 as seed ortholog is 100%.
Bootstrap support for CQW49_00020 as seed ortholog is 100%.
```

---

### Group of orthologs #285. Best score 1345 bits Score difference with first non-orthologous sequence - OB3b\_Refseq\_for\_inparanoid.fasta:1345 OB3b\_Genbank\_for\_inparanoid.fasta:1345

```
shc                 	100.00%		CQW49_17110         	100.00%
Bootstrap support for shc as seed ortholog is 100%.
Bootstrap support for CQW49_17110 as seed ortholog is 100%.
```

---

### Group of orthologs #286. Best score 1342 bits Score difference with first non-orthologous sequence - OB3b\_Refseq\_for\_inparanoid.fasta:1342 OB3b\_Genbank\_for\_inparanoid.fasta:1342

```
CQW49_RS17790       	100.00%		CQW49_17735         	100.00%
Bootstrap support for CQW49_RS17790 as seed ortholog is 100%.
Bootstrap support for CQW49_17735 as seed ortholog is 100%.
```

---

### Group of orthologs #287. Best score 1338 bits Score difference with first non-orthologous sequence - OB3b\_Refseq\_for\_inparanoid.fasta:1338 OB3b\_Genbank\_for\_inparanoid.fasta:1338

```
CQW49_RS14115       	100.00%		CQW49_14080         	100.00%
Bootstrap support for CQW49_RS14115 as seed ortholog is 100%.
Bootstrap support for CQW49_14080 as seed ortholog is 100%.
```

---

### Group of orthologs #288. Best score 1338 bits Score difference with first non-orthologous sequence - OB3b\_Refseq\_for\_inparanoid.fasta:1338 OB3b\_Genbank\_for\_inparanoid.fasta:1338

```
CQW49_RS18420       	100.00%		CQW49_18360         	100.00%
Bootstrap support for CQW49_RS18420 as seed ortholog is 100%.
Bootstrap support for CQW49_18360 as seed ortholog is 100%.
```

---

### Group of orthologs #289. Best score 1336 bits Score difference with first non-orthologous sequence - OB3b\_Refseq\_for\_inparanoid.fasta:1336 OB3b\_Genbank\_for\_inparanoid.fasta:1260

```
CQW49_RS10010       	100.00%		CQW49_09985         	100.00%
Bootstrap support for CQW49_RS10010 as seed ortholog is 100%.
Bootstrap support for CQW49_09985 as seed ortholog is 100%.
```

---

### Group of orthologs #290. Best score 1336 bits Score difference with first non-orthologous sequence - OB3b\_Refseq\_for\_inparanoid.fasta:1336 OB3b\_Genbank\_for\_inparanoid.fasta:1336

```
CQW49_RS21185       	100.00%		CQW49_21110         	100.00%
Bootstrap support for CQW49_RS21185 as seed ortholog is 100%.
Bootstrap support for CQW49_21110 as seed ortholog is 100%.
```

---

### Group of orthologs #291. Best score 1335 bits Score difference with first non-orthologous sequence - OB3b\_Refseq\_for\_inparanoid.fasta:1335 OB3b\_Genbank\_for\_inparanoid.fasta:1335

```
CQW49_RS15045       	100.00%		CQW49_15010         	100.00%
Bootstrap support for CQW49_RS15045 as seed ortholog is 100%.
Bootstrap support for CQW49_15010 as seed ortholog is 100%.
```

---

### Group of orthologs #292. Best score 1334 bits Score difference with first non-orthologous sequence - OB3b\_Refseq\_for\_inparanoid.fasta:1334 OB3b\_Genbank\_for\_inparanoid.fasta:1334

```
parE                	100.00%		CQW49_09465         	100.00%
Bootstrap support for parE as seed ortholog is 100%.
Bootstrap support for CQW49_09465 as seed ortholog is 100%.
```

---

### Group of orthologs #293. Best score 1333 bits Score difference with first non-orthologous sequence - OB3b\_Refseq\_for\_inparanoid.fasta:1333 OB3b\_Genbank\_for\_inparanoid.fasta:1333

```
CQW49_RS10795       	100.00%		CQW49_10765         	100.00%
Bootstrap support for CQW49_RS10795 as seed ortholog is 100%.
Bootstrap support for CQW49_10765 as seed ortholog is 100%.
```

---

### Group of orthologs #294. Best score 1332 bits Score difference with first non-orthologous sequence - OB3b\_Refseq\_for\_inparanoid.fasta:1332 OB3b\_Genbank\_for\_inparanoid.fasta:1332

```
CQW49_RS17795       	100.00%		CQW49_17740         	100.00%
Bootstrap support for CQW49_RS17795 as seed ortholog is 100%.
Bootstrap support for CQW49_17740 as seed ortholog is 100%.
```

---

### Group of orthologs #295. Best score 1327 bits Score difference with first non-orthologous sequence - OB3b\_Refseq\_for\_inparanoid.fasta:1327 OB3b\_Genbank\_for\_inparanoid.fasta:1327

```
CQW49_RS20870       	100.00%		CQW49_20795         	100.00%
Bootstrap support for CQW49_RS20870 as seed ortholog is 100%.
Bootstrap support for CQW49_20795 as seed ortholog is 100%.
```

---

### Group of orthologs #296. Best score 1324 bits Score difference with first non-orthologous sequence - OB3b\_Refseq\_for\_inparanoid.fasta:1324 OB3b\_Genbank\_for\_inparanoid.fasta:1324

```
kdpB                	100.00%		CQW49_10625         	100.00%
Bootstrap support for kdpB as seed ortholog is 100%.
Bootstrap support for CQW49_10625 as seed ortholog is 100%.
```

---

### Group of orthologs #297. Best score 1323 bits Score difference with first non-orthologous sequence - OB3b\_Refseq\_for\_inparanoid.fasta:1323 OB3b\_Genbank\_for\_inparanoid.fasta:1323

```
CQW49_RS19770       	100.00%		CQW49_19715         	100.00%
Bootstrap support for CQW49_RS19770 as seed ortholog is 100%.
Bootstrap support for CQW49_19715 as seed ortholog is 100%.
```

---

### Group of orthologs #298. Best score 1321 bits Score difference with first non-orthologous sequence - OB3b\_Refseq\_for\_inparanoid.fasta:1321 OB3b\_Genbank\_for\_inparanoid.fasta:1321

```
CQW49_RS17990       	100.00%		CQW49_17935         	100.00%
Bootstrap support for CQW49_RS17990 as seed ortholog is 100%.
Bootstrap support for CQW49_17935 as seed ortholog is 100%.
```

---

### Group of orthologs #299. Best score 1321 bits Score difference with first non-orthologous sequence - OB3b\_Refseq\_for\_inparanoid.fasta:1321 OB3b\_Genbank\_for\_inparanoid.fasta:1321

```
CQW49_RS20880       	100.00%		CQW49_20805         	100.00%
Bootstrap support for CQW49_RS20880 as seed ortholog is 100%.
Bootstrap support for CQW49_20805 as seed ortholog is 100%.
```

---

### Group of orthologs #300. Best score 1320 bits Score difference with first non-orthologous sequence - OB3b\_Refseq\_for\_inparanoid.fasta:1320 OB3b\_Genbank\_for\_inparanoid.fasta:1320

```
tkt                 	100.00%		CQW49_18400         	100.00%
Bootstrap support for tkt as seed ortholog is 100%.
Bootstrap support for CQW49_18400 as seed ortholog is 100%.
```

---

### Group of orthologs #301. Best score 1319 bits Score difference with first non-orthologous sequence - OB3b\_Refseq\_for\_inparanoid.fasta:1319 OB3b\_Genbank\_for\_inparanoid.fasta:1319

```
CQW49_RS17335       	100.00%		CQW49_17285         	100.00%
Bootstrap support for CQW49_RS17335 as seed ortholog is 100%.
Bootstrap support for CQW49_17285 as seed ortholog is 100%.
```

---

### Group of orthologs #302. Best score 1318 bits Score difference with first non-orthologous sequence - OB3b\_Refseq\_for\_inparanoid.fasta:1318 OB3b\_Genbank\_for\_inparanoid.fasta:1318

```
CQW49_RS03700       	100.00%		CQW49_03690         	100.00%
Bootstrap support for CQW49_RS03700 as seed ortholog is 100%.
Bootstrap support for CQW49_03690 as seed ortholog is 100%.
```

---

### Group of orthologs #303. Best score 1318 bits Score difference with first non-orthologous sequence - OB3b\_Refseq\_for\_inparanoid.fasta:1318 OB3b\_Genbank\_for\_inparanoid.fasta:1318

```
CQW49_RS17285       	100.00%		CQW49_17240         	100.00%
Bootstrap support for CQW49_RS17285 as seed ortholog is 100%.
Bootstrap support for CQW49_17240 as seed ortholog is 100%.
```

---

### Group of orthologs #304. Best score 1317 bits Score difference with first non-orthologous sequence - OB3b\_Refseq\_for\_inparanoid.fasta:1317 OB3b\_Genbank\_for\_inparanoid.fasta:1317

```
CQW49_RS09915       	100.00%		CQW49_09895         	100.00%
Bootstrap support for CQW49_RS09915 as seed ortholog is 100%.
Bootstrap support for CQW49_09895 as seed ortholog is 100%.
```

---

### Group of orthologs #305. Best score 1308 bits Score difference with first non-orthologous sequence - OB3b\_Refseq\_for\_inparanoid.fasta:1308 OB3b\_Genbank\_for\_inparanoid.fasta:1308

```
CQW49_RS10215       	100.00%		CQW49_10190         	100.00%
Bootstrap support for CQW49_RS10215 as seed ortholog is 100%.
Bootstrap support for CQW49_10190 as seed ortholog is 100%.
```

---

### Group of orthologs #306. Best score 1307 bits Score difference with first non-orthologous sequence - OB3b\_Refseq\_for\_inparanoid.fasta:1307 OB3b\_Genbank\_for\_inparanoid.fasta:1307

```
CQW49_RS14460       	100.00%		CQW49_14425         	100.00%
Bootstrap support for CQW49_RS14460 as seed ortholog is 100%.
Bootstrap support for CQW49_14425 as seed ortholog is 100%.
```

---

### Group of orthologs #307. Best score 1305 bits Score difference with first non-orthologous sequence - OB3b\_Refseq\_for\_inparanoid.fasta:1305 OB3b\_Genbank\_for\_inparanoid.fasta:1305

```
CQW49_RS03315       	100.00%		CQW49_03305         	100.00%
Bootstrap support for CQW49_RS03315 as seed ortholog is 100%.
Bootstrap support for CQW49_03305 as seed ortholog is 100%.
```

---

### Group of orthologs #308. Best score 1304 bits Score difference with first non-orthologous sequence - OB3b\_Refseq\_for\_inparanoid.fasta:1304 OB3b\_Genbank\_for\_inparanoid.fasta:1304

```
CQW49_RS16010       	100.00%		CQW49_15975         	100.00%
Bootstrap support for CQW49_RS16010 as seed ortholog is 100%.
Bootstrap support for CQW49_15975 as seed ortholog is 100%.
```

---

### Group of orthologs #309. Best score 1297 bits Score difference with first non-orthologous sequence - OB3b\_Refseq\_for\_inparanoid.fasta:1297 OB3b\_Genbank\_for\_inparanoid.fasta:1297

```
CQW49_RS19390       	100.00%		CQW49_19335         	100.00%
Bootstrap support for CQW49_RS19390 as seed ortholog is 100%.
Bootstrap support for CQW49_19335 as seed ortholog is 100%.
```

---

### Group of orthologs #310. Best score 1296 bits Score difference with first non-orthologous sequence - OB3b\_Refseq\_for\_inparanoid.fasta:1296 OB3b\_Genbank\_for\_inparanoid.fasta:1296

```
CQW49_RS00685       	100.00%		CQW49_00685         	100.00%
Bootstrap support for CQW49_RS00685 as seed ortholog is 100%.
Bootstrap support for CQW49_00685 as seed ortholog is 100%.
```

---

### Group of orthologs #311. Best score 1293 bits Score difference with first non-orthologous sequence - OB3b\_Refseq\_for\_inparanoid.fasta:1293 OB3b\_Genbank\_for\_inparanoid.fasta:1293

```
CQW49_RS04780       	100.00%		CQW49_04770         	100.00%
Bootstrap support for CQW49_RS04780 as seed ortholog is 100%.
Bootstrap support for CQW49_04770 as seed ortholog is 100%.
```

---

### Group of orthologs #312. Best score 1292 bits Score difference with first non-orthologous sequence - OB3b\_Refseq\_for\_inparanoid.fasta:1292 OB3b\_Genbank\_for\_inparanoid.fasta:1292

```
CQW49_RS20220       	100.00%		CQW49_20150         	100.00%
Bootstrap support for CQW49_RS20220 as seed ortholog is 100%.
Bootstrap support for CQW49_20150 as seed ortholog is 100%.
```

---

### Group of orthologs #313. Best score 1291 bits Score difference with first non-orthologous sequence - OB3b\_Refseq\_for\_inparanoid.fasta:1291 OB3b\_Genbank\_for\_inparanoid.fasta:1291

```
CQW49_RS15650       	100.00%		CQW49_15615         	100.00%
Bootstrap support for CQW49_RS15650 as seed ortholog is 100%.
Bootstrap support for CQW49_15615 as seed ortholog is 100%.
```

---

### Group of orthologs #314. Best score 1290 bits Score difference with first non-orthologous sequence - OB3b\_Refseq\_for\_inparanoid.fasta:1290 OB3b\_Genbank\_for\_inparanoid.fasta:1290

```
CQW49_RS09780       	100.00%		CQW49_09760         	100.00%
Bootstrap support for CQW49_RS09780 as seed ortholog is 100%.
Bootstrap support for CQW49_09760 as seed ortholog is 100%.
```

---

### Group of orthologs #315. Best score 1287 bits Score difference with first non-orthologous sequence - OB3b\_Refseq\_for\_inparanoid.fasta:1287 OB3b\_Genbank\_for\_inparanoid.fasta:1287

```
CQW49_RS11320       	100.00%		CQW49_11290         	100.00%
Bootstrap support for CQW49_RS11320 as seed ortholog is 100%.
Bootstrap support for CQW49_11290 as seed ortholog is 100%.
```

---

### Group of orthologs #316. Best score 1287 bits Score difference with first non-orthologous sequence - OB3b\_Refseq\_for\_inparanoid.fasta:1287 OB3b\_Genbank\_for\_inparanoid.fasta:1287

```
CQW49_RS12980       	100.00%		CQW49_12945         	100.00%
Bootstrap support for CQW49_RS12980 as seed ortholog is 100%.
Bootstrap support for CQW49_12945 as seed ortholog is 100%.
```

---

### Group of orthologs #317. Best score 1285 bits Score difference with first non-orthologous sequence - OB3b\_Refseq\_for\_inparanoid.fasta:1285 OB3b\_Genbank\_for\_inparanoid.fasta:1285

```
CQW49_RS18690       	100.00%		CQW49_18630         	100.00%
Bootstrap support for CQW49_RS18690 as seed ortholog is 100%.
Bootstrap support for CQW49_18630 as seed ortholog is 100%.
```

---

### Group of orthologs #318. Best score 1284 bits Score difference with first non-orthologous sequence - OB3b\_Refseq\_for\_inparanoid.fasta:1284 OB3b\_Genbank\_for\_inparanoid.fasta:1284

```
CQW49_RS01245       	100.00%		CQW49_01240         	100.00%
Bootstrap support for CQW49_RS01245 as seed ortholog is 100%.
Bootstrap support for CQW49_01240 as seed ortholog is 100%.
```

---

### Group of orthologs #319. Best score 1282 bits Score difference with first non-orthologous sequence - OB3b\_Refseq\_for\_inparanoid.fasta:1282 OB3b\_Genbank\_for\_inparanoid.fasta:1282

```
CQW49_RS09705       	100.00%		CQW49_09685         	100.00%
Bootstrap support for CQW49_RS09705 as seed ortholog is 100%.
Bootstrap support for CQW49_09685 as seed ortholog is 100%.
```

---

### Group of orthologs #320. Best score 1280 bits Score difference with first non-orthologous sequence - OB3b\_Refseq\_for\_inparanoid.fasta:1280 OB3b\_Genbank\_for\_inparanoid.fasta:1280

```
CQW49_RS21010       	100.00%		CQW49_20935         	100.00%
Bootstrap support for CQW49_RS21010 as seed ortholog is 100%.
Bootstrap support for CQW49_20935 as seed ortholog is 100%.
```

---

### Group of orthologs #321. Best score 1279 bits Score difference with first non-orthologous sequence - OB3b\_Refseq\_for\_inparanoid.fasta:1279 OB3b\_Genbank\_for\_inparanoid.fasta:1279

```
CQW49_RS17395       	100.00%		CQW49_17345         	100.00%
Bootstrap support for CQW49_RS17395 as seed ortholog is 100%.
Bootstrap support for CQW49_17345 as seed ortholog is 100%.
```

---

### Group of orthologs #322. Best score 1279 bits Score difference with first non-orthologous sequence - OB3b\_Refseq\_for\_inparanoid.fasta:1279 OB3b\_Genbank\_for\_inparanoid.fasta:1279

```
CQW49_RS19150       	100.00%		CQW49_19090         	100.00%
Bootstrap support for CQW49_RS19150 as seed ortholog is 100%.
Bootstrap support for CQW49_19090 as seed ortholog is 100%.
```

---

### Group of orthologs #323. Best score 1278 bits Score difference with first non-orthologous sequence - OB3b\_Refseq\_for\_inparanoid.fasta:1278 OB3b\_Genbank\_for\_inparanoid.fasta:1278

```
CQW49_RS09190       	100.00%		CQW49_09170         	100.00%
Bootstrap support for CQW49_RS09190 as seed ortholog is 100%.
Bootstrap support for CQW49_09170 as seed ortholog is 100%.
```

---

### Group of orthologs #324. Best score 1275 bits Score difference with first non-orthologous sequence - OB3b\_Refseq\_for\_inparanoid.fasta:1275 OB3b\_Genbank\_for\_inparanoid.fasta:1275

```
CQW49_RS18310       	100.00%		CQW49_18250         	100.00%
Bootstrap support for CQW49_RS18310 as seed ortholog is 100%.
Bootstrap support for CQW49_18250 as seed ortholog is 100%.
```

---

### Group of orthologs #325. Best score 1274 bits Score difference with first non-orthologous sequence - OB3b\_Refseq\_for\_inparanoid.fasta:1274 OB3b\_Genbank\_for\_inparanoid.fasta:1274

```
CQW49_RS18920       	100.00%		CQW49_18860         	100.00%
Bootstrap support for CQW49_RS18920 as seed ortholog is 100%.
Bootstrap support for CQW49_18860 as seed ortholog is 100%.
```

---

### Group of orthologs #326. Best score 1272 bits Score difference with first non-orthologous sequence - OB3b\_Refseq\_for\_inparanoid.fasta:1272 OB3b\_Genbank\_for\_inparanoid.fasta:1272

```
CQW49_RS12530       	100.00%		CQW49_12495         	100.00%
Bootstrap support for CQW49_RS12530 as seed ortholog is 100%.
Bootstrap support for CQW49_12495 as seed ortholog is 100%.
```

---

### Group of orthologs #327. Best score 1272 bits Score difference with first non-orthologous sequence - OB3b\_Refseq\_for\_inparanoid.fasta:1272 OB3b\_Genbank\_for\_inparanoid.fasta:1272

```
CQW49_RS15875       	100.00%		CQW49_15840         	100.00%
Bootstrap support for CQW49_RS15875 as seed ortholog is 100%.
Bootstrap support for CQW49_15840 as seed ortholog is 100%.
```

---

### Group of orthologs #328. Best score 1271 bits Score difference with first non-orthologous sequence - OB3b\_Refseq\_for\_inparanoid.fasta:1271 OB3b\_Genbank\_for\_inparanoid.fasta:1271

```
CQW49_RS14430       	100.00%		CQW49_14395         	100.00%
Bootstrap support for CQW49_RS14430 as seed ortholog is 100%.
Bootstrap support for CQW49_14395 as seed ortholog is 100%.
```

---

### Group of orthologs #329. Best score 1268 bits Score difference with first non-orthologous sequence - OB3b\_Refseq\_for\_inparanoid.fasta:1268 OB3b\_Genbank\_for\_inparanoid.fasta:1268

```
CQW49_RS04775       	100.00%		CQW49_04765         	100.00%
Bootstrap support for CQW49_RS04775 as seed ortholog is 100%.
Bootstrap support for CQW49_04765 as seed ortholog is 100%.
```

---

### Group of orthologs #330. Best score 1266 bits Score difference with first non-orthologous sequence - OB3b\_Refseq\_for\_inparanoid.fasta:1266 OB3b\_Genbank\_for\_inparanoid.fasta:1266

```
CQW49_RS09240       	100.00%		CQW49_09220         	100.00%
Bootstrap support for CQW49_RS09240 as seed ortholog is 100%.
Bootstrap support for CQW49_09220 as seed ortholog is 100%.
```

---

### Group of orthologs #331. Best score 1262 bits Score difference with first non-orthologous sequence - OB3b\_Refseq\_for\_inparanoid.fasta:1262 OB3b\_Genbank\_for\_inparanoid.fasta:1262

```
CQW49_RS06110       	100.00%		CQW49_06100         	100.00%
Bootstrap support for CQW49_RS06110 as seed ortholog is 100%.
Bootstrap support for CQW49_06100 as seed ortholog is 100%.
```

---

### Group of orthologs #332. Best score 1262 bits Score difference with first non-orthologous sequence - OB3b\_Refseq\_for\_inparanoid.fasta:1262 OB3b\_Genbank\_for\_inparanoid.fasta:1262

```
trkD                	100.00%		CQW49_11755         	100.00%
Bootstrap support for trkD as seed ortholog is 100%.
Bootstrap support for CQW49_11755 as seed ortholog is 100%.
```

---

### Group of orthologs #333. Best score 1260 bits Score difference with first non-orthologous sequence - OB3b\_Refseq\_for\_inparanoid.fasta:1260 OB3b\_Genbank\_for\_inparanoid.fasta:1260

```
CQW49_RS02340       	100.00%		CQW49_02335         	100.00%
Bootstrap support for CQW49_RS02340 as seed ortholog is 100%.
Bootstrap support for CQW49_02335 as seed ortholog is 100%.
```

---

### Group of orthologs #334. Best score 1260 bits Score difference with first non-orthologous sequence - OB3b\_Refseq\_for\_inparanoid.fasta:1260 OB3b\_Genbank\_for\_inparanoid.fasta:1260

```
acs                 	100.00%		CQW49_13835         	100.00%
Bootstrap support for acs as seed ortholog is 100%.
Bootstrap support for CQW49_13835 as seed ortholog is 100%.
```

---

### Group of orthologs #335. Best score 1260 bits Score difference with first non-orthologous sequence - OB3b\_Refseq\_for\_inparanoid.fasta:1260 OB3b\_Genbank\_for\_inparanoid.fasta:1260

```
CQW49_RS16895       	100.00%		CQW49_16850         	100.00%
Bootstrap support for CQW49_RS16895 as seed ortholog is 100%.
Bootstrap support for CQW49_16850 as seed ortholog is 100%.
```

---

### Group of orthologs #336. Best score 1260 bits Score difference with first non-orthologous sequence - OB3b\_Refseq\_for\_inparanoid.fasta:1260 OB3b\_Genbank\_for\_inparanoid.fasta:1260

```
CQW49_RS18575       	100.00%		CQW49_18515         	100.00%
Bootstrap support for CQW49_RS18575 as seed ortholog is 100%.
Bootstrap support for CQW49_18515 as seed ortholog is 100%.
```

---

### Group of orthologs #337. Best score 1259 bits Score difference with first non-orthologous sequence - OB3b\_Refseq\_for\_inparanoid.fasta:1259 OB3b\_Genbank\_for\_inparanoid.fasta:1259

```
CQW49_RS18565       	100.00%		CQW49_18505         	100.00%
Bootstrap support for CQW49_RS18565 as seed ortholog is 100%.
Bootstrap support for CQW49_18505 as seed ortholog is 100%.
```

---

### Group of orthologs #338. Best score 1257 bits Score difference with first non-orthologous sequence - OB3b\_Refseq\_for\_inparanoid.fasta:1257 OB3b\_Genbank\_for\_inparanoid.fasta:1124

```
CQW49_RS09790       	100.00%		CQW49_09770         	100.00%
Bootstrap support for CQW49_RS09790 as seed ortholog is 100%.
Bootstrap support for CQW49_09770 as seed ortholog is 100%.
```

---

### Group of orthologs #339. Best score 1255 bits Score difference with first non-orthologous sequence - OB3b\_Refseq\_for\_inparanoid.fasta:1255 OB3b\_Genbank\_for\_inparanoid.fasta:1255

```
cobT                	100.00%		CQW49_10210         	100.00%
cobT                	100.00%		
Bootstrap support for cobT as seed ortholog is 100%.
Bootstrap support for cobT as seed ortholog is 100%.
Bootstrap support for CQW49_10210 as seed ortholog is 100%.
```

---

### Group of orthologs #340. Best score 1255 bits Score difference with first non-orthologous sequence - OB3b\_Refseq\_for\_inparanoid.fasta:1255 OB3b\_Genbank\_for\_inparanoid.fasta:1255

```
cysC                	100.00%		CQW49_18065         	100.00%
Bootstrap support for cysC as seed ortholog is 100%.
Bootstrap support for CQW49_18065 as seed ortholog is 100%.
```

---

### Group of orthologs #341. Best score 1251 bits Score difference with first non-orthologous sequence - OB3b\_Refseq\_for\_inparanoid.fasta:1251 OB3b\_Genbank\_for\_inparanoid.fasta:1251

```
CQW49_RS00765       	100.00%		CQW49_00765         	100.00%
Bootstrap support for CQW49_RS00765 as seed ortholog is 100%.
Bootstrap support for CQW49_00765 as seed ortholog is 100%.
```

---

### Group of orthologs #342. Best score 1250 bits Score difference with first non-orthologous sequence - OB3b\_Refseq\_for\_inparanoid.fasta:1250 OB3b\_Genbank\_for\_inparanoid.fasta:1250

```
CQW49_RS02365       	100.00%		CQW49_02360         	100.00%
Bootstrap support for CQW49_RS02365 as seed ortholog is 100%.
Bootstrap support for CQW49_02360 as seed ortholog is 100%.
```

---

### Group of orthologs #343. Best score 1250 bits Score difference with first non-orthologous sequence - OB3b\_Refseq\_for\_inparanoid.fasta:1250 OB3b\_Genbank\_for\_inparanoid.fasta:997

```
CQW49_RS09590       	100.00%		CQW49_09570         	100.00%
Bootstrap support for CQW49_RS09590 as seed ortholog is 100%.
Bootstrap support for CQW49_09570 as seed ortholog is 100%.
```

---

### Group of orthologs #344. Best score 1246 bits Score difference with first non-orthologous sequence - OB3b\_Refseq\_for\_inparanoid.fasta:1246 OB3b\_Genbank\_for\_inparanoid.fasta:1246

```
CQW49_RS13985       	100.00%		CQW49_13950         	100.00%
Bootstrap support for CQW49_RS13985 as seed ortholog is 100%.
Bootstrap support for CQW49_13950 as seed ortholog is 100%.
```

---

### Group of orthologs #345. Best score 1242 bits Score difference with first non-orthologous sequence - OB3b\_Refseq\_for\_inparanoid.fasta:1242 OB3b\_Genbank\_for\_inparanoid.fasta:1242

```
CQW49_RS10840       	100.00%		CQW49_10810         	100.00%
Bootstrap support for CQW49_RS10840 as seed ortholog is 100%.
Bootstrap support for CQW49_10810 as seed ortholog is 100%.
```

---

### Group of orthologs #346. Best score 1239 bits Score difference with first non-orthologous sequence - OB3b\_Refseq\_for\_inparanoid.fasta:1239 OB3b\_Genbank\_for\_inparanoid.fasta:1239

```
CQW49_RS04945       	100.00%		CQW49_04940         	100.00%
Bootstrap support for CQW49_RS04945 as seed ortholog is 100%.
Bootstrap support for CQW49_04940 as seed ortholog is 100%.
```

---

### Group of orthologs #347. Best score 1235 bits Score difference with first non-orthologous sequence - OB3b\_Refseq\_for\_inparanoid.fasta:1235 OB3b\_Genbank\_for\_inparanoid.fasta:1235

```
CQW49_RS03520       	100.00%		CQW49_03510         	100.00%
Bootstrap support for CQW49_RS03520 as seed ortholog is 100%.
Bootstrap support for CQW49_03510 as seed ortholog is 100%.
```

---

### Group of orthologs #348. Best score 1235 bits Score difference with first non-orthologous sequence - OB3b\_Refseq\_for\_inparanoid.fasta:1235 OB3b\_Genbank\_for\_inparanoid.fasta:1235

```
CQW49_RS07785       	100.00%		CQW49_07775         	100.00%
Bootstrap support for CQW49_RS07785 as seed ortholog is 100%.
Bootstrap support for CQW49_07775 as seed ortholog is 100%.
```

---

### Group of orthologs #349. Best score 1233 bits Score difference with first non-orthologous sequence - OB3b\_Refseq\_for\_inparanoid.fasta:1233 OB3b\_Genbank\_for\_inparanoid.fasta:1233

```
CQW49_RS06035       	100.00%		CQW49_06025         	100.00%
Bootstrap support for CQW49_RS06035 as seed ortholog is 100%.
Bootstrap support for CQW49_06025 as seed ortholog is 100%.
```

---

### Group of orthologs #350. Best score 1233 bits Score difference with first non-orthologous sequence - OB3b\_Refseq\_for\_inparanoid.fasta:1233 OB3b\_Genbank\_for\_inparanoid.fasta:1233

```
CQW49_RS12465       	100.00%		CQW49_12430         	100.00%
Bootstrap support for CQW49_RS12465 as seed ortholog is 100%.
Bootstrap support for CQW49_12430 as seed ortholog is 100%.
```

---

### Group of orthologs #351. Best score 1232 bits Score difference with first non-orthologous sequence - OB3b\_Refseq\_for\_inparanoid.fasta:1232 OB3b\_Genbank\_for\_inparanoid.fasta:1232

```
CQW49_RS20505       	100.00%		CQW49_20435         	100.00%
Bootstrap support for CQW49_RS20505 as seed ortholog is 100%.
Bootstrap support for CQW49_20435 as seed ortholog is 100%.
```

---

### Group of orthologs #352. Best score 1231 bits Score difference with first non-orthologous sequence - OB3b\_Refseq\_for\_inparanoid.fasta:1231 OB3b\_Genbank\_for\_inparanoid.fasta:1231

```
CQW49_RS08615       	100.00%		CQW49_08605         	100.00%
Bootstrap support for CQW49_RS08615 as seed ortholog is 100%.
Bootstrap support for CQW49_08605 as seed ortholog is 100%.
```

---

### Group of orthologs #353. Best score 1231 bits Score difference with first non-orthologous sequence - OB3b\_Refseq\_for\_inparanoid.fasta:1231 OB3b\_Genbank\_for\_inparanoid.fasta:1231

```
CQW49_RS18980       	100.00%		CQW49_18920         	100.00%
Bootstrap support for CQW49_RS18980 as seed ortholog is 100%.
Bootstrap support for CQW49_18920 as seed ortholog is 100%.
```

---

### Group of orthologs #354. Best score 1229 bits Score difference with first non-orthologous sequence - OB3b\_Refseq\_for\_inparanoid.fasta:1229 OB3b\_Genbank\_for\_inparanoid.fasta:1229

```
CQW49_RS13010       	100.00%		CQW49_12975         	100.00%
Bootstrap support for CQW49_RS13010 as seed ortholog is 100%.
Bootstrap support for CQW49_12975 as seed ortholog is 100%.
```

---

### Group of orthologs #355. Best score 1228 bits Score difference with first non-orthologous sequence - OB3b\_Refseq\_for\_inparanoid.fasta:1228 OB3b\_Genbank\_for\_inparanoid.fasta:1228

```
CQW49_RS07235       	100.00%		CQW49_07225         	100.00%
Bootstrap support for CQW49_RS07235 as seed ortholog is 100%.
Bootstrap support for CQW49_07225 as seed ortholog is 100%.
```

---

### Group of orthologs #356. Best score 1227 bits Score difference with first non-orthologous sequence - OB3b\_Refseq\_for\_inparanoid.fasta:1227 OB3b\_Genbank\_for\_inparanoid.fasta:1227

```
CQW49_RS05580       	100.00%		CQW49_05570         	100.00%
Bootstrap support for CQW49_RS05580 as seed ortholog is 100%.
Bootstrap support for CQW49_05570 as seed ortholog is 100%.
```

---

### Group of orthologs #357. Best score 1227 bits Score difference with first non-orthologous sequence - OB3b\_Refseq\_for\_inparanoid.fasta:1227 OB3b\_Genbank\_for\_inparanoid.fasta:1227

```
CQW49_RS09585       	100.00%		CQW49_09565         	100.00%
Bootstrap support for CQW49_RS09585 as seed ortholog is 100%.
Bootstrap support for CQW49_09565 as seed ortholog is 100%.
```

---

### Group of orthologs #358. Best score 1225 bits Score difference with first non-orthologous sequence - OB3b\_Refseq\_for\_inparanoid.fasta:1225 OB3b\_Genbank\_for\_inparanoid.fasta:1225

```
CQW49_RS07925       	100.00%		CQW49_07915         	100.00%
Bootstrap support for CQW49_RS07925 as seed ortholog is 100%.
Bootstrap support for CQW49_07915 as seed ortholog is 100%.
```

---

### Group of orthologs #359. Best score 1225 bits Score difference with first non-orthologous sequence - OB3b\_Refseq\_for\_inparanoid.fasta:1225 OB3b\_Genbank\_for\_inparanoid.fasta:1225

```
CQW49_RS12590       	100.00%		CQW49_12555         	100.00%
Bootstrap support for CQW49_RS12590 as seed ortholog is 100%.
Bootstrap support for CQW49_12555 as seed ortholog is 100%.
```

---

### Group of orthologs #360. Best score 1223 bits Score difference with first non-orthologous sequence - OB3b\_Refseq\_for\_inparanoid.fasta:1223 OB3b\_Genbank\_for\_inparanoid.fasta:1223

```
CQW49_RS03645       	100.00%		CQW49_03635         	100.00%
Bootstrap support for CQW49_RS03645 as seed ortholog is 100%.
Bootstrap support for CQW49_03635 as seed ortholog is 100%.
```

---

### Group of orthologs #361. Best score 1223 bits Score difference with first non-orthologous sequence - OB3b\_Refseq\_for\_inparanoid.fasta:1223 OB3b\_Genbank\_for\_inparanoid.fasta:1223

```
CQW49_RS20640       	100.00%		CQW49_20565         	100.00%
Bootstrap support for CQW49_RS20640 as seed ortholog is 100%.
Bootstrap support for CQW49_20565 as seed ortholog is 100%.
```

---

### Group of orthologs #362. Best score 1218 bits Score difference with first non-orthologous sequence - OB3b\_Refseq\_for\_inparanoid.fasta:1218 OB3b\_Genbank\_for\_inparanoid.fasta:1136

```
CQW49_RS16495       	100.00%		CQW49_16460         	100.00%
Bootstrap support for CQW49_RS16495 as seed ortholog is 100%.
Bootstrap support for CQW49_16460 as seed ortholog is 100%.
```

---

### Group of orthologs #363. Best score 1217 bits Score difference with first non-orthologous sequence - OB3b\_Refseq\_for\_inparanoid.fasta:1217 OB3b\_Genbank\_for\_inparanoid.fasta:1217

```
CQW49_RS02385       	100.00%		CQW49_02375         	100.00%
Bootstrap support for CQW49_RS02385 as seed ortholog is 100%.
Bootstrap support for CQW49_02375 as seed ortholog is 100%.
```

---

### Group of orthologs #364. Best score 1215 bits Score difference with first non-orthologous sequence - OB3b\_Refseq\_for\_inparanoid.fasta:1215 OB3b\_Genbank\_for\_inparanoid.fasta:1215

```
CQW49_RS16235       	100.00%		CQW49_16200         	100.00%
Bootstrap support for CQW49_RS16235 as seed ortholog is 100%.
Bootstrap support for CQW49_16200 as seed ortholog is 100%.
```

---

### Group of orthologs #365. Best score 1215 bits Score difference with first non-orthologous sequence - OB3b\_Refseq\_for\_inparanoid.fasta:1215 OB3b\_Genbank\_for\_inparanoid.fasta:1215

```
asnB                	100.00%		CQW49_19895         	100.00%
Bootstrap support for asnB as seed ortholog is 100%.
Bootstrap support for CQW49_19895 as seed ortholog is 100%.
```

---

### Group of orthologs #366. Best score 1215 bits Score difference with first non-orthologous sequence - OB3b\_Refseq\_for\_inparanoid.fasta:1215 OB3b\_Genbank\_for\_inparanoid.fasta:1215

```
CQW49_RS20345       	100.00%		CQW49_20275         	100.00%
Bootstrap support for CQW49_RS20345 as seed ortholog is 100%.
Bootstrap support for CQW49_20275 as seed ortholog is 100%.
```

---

### Group of orthologs #367. Best score 1210 bits Score difference with first non-orthologous sequence - OB3b\_Refseq\_for\_inparanoid.fasta:1210 OB3b\_Genbank\_for\_inparanoid.fasta:1210

```
CQW49_RS12800       	100.00%		CQW49_12765         	100.00%
Bootstrap support for CQW49_RS12800 as seed ortholog is 100%.
Bootstrap support for CQW49_12765 as seed ortholog is 100%.
```

---

### Group of orthologs #368. Best score 1208 bits Score difference with first non-orthologous sequence - OB3b\_Refseq\_for\_inparanoid.fasta:1208 OB3b\_Genbank\_for\_inparanoid.fasta:1208

```
CQW49_RS03915       	100.00%		CQW49_03905         	100.00%
Bootstrap support for CQW49_RS03915 as seed ortholog is 100%.
Bootstrap support for CQW49_03905 as seed ortholog is 100%.
```

---

### Group of orthologs #369. Best score 1208 bits Score difference with first non-orthologous sequence - OB3b\_Refseq\_for\_inparanoid.fasta:1208 OB3b\_Genbank\_for\_inparanoid.fasta:1208

```
CQW49_RS17505       	100.00%		CQW49_17450         	100.00%
Bootstrap support for CQW49_RS17505 as seed ortholog is 100%.
Bootstrap support for CQW49_17450 as seed ortholog is 100%.
```

---

### Group of orthologs #370. Best score 1207 bits Score difference with first non-orthologous sequence - OB3b\_Refseq\_for\_inparanoid.fasta:1207 OB3b\_Genbank\_for\_inparanoid.fasta:1207

```
CQW49_RS07455       	100.00%		CQW49_07445         	100.00%
Bootstrap support for CQW49_RS07455 as seed ortholog is 100%.
Bootstrap support for CQW49_07445 as seed ortholog is 100%.
```

---

### Group of orthologs #371. Best score 1207 bits Score difference with first non-orthologous sequence - OB3b\_Refseq\_for\_inparanoid.fasta:1207 OB3b\_Genbank\_for\_inparanoid.fasta:1207

```
CQW49_RS17750       	100.00%		CQW49_17695         	100.00%
Bootstrap support for CQW49_RS17750 as seed ortholog is 100%.
Bootstrap support for CQW49_17695 as seed ortholog is 100%.
```

---

### Group of orthologs #372. Best score 1205 bits Score difference with first non-orthologous sequence - OB3b\_Refseq\_for\_inparanoid.fasta:1205 OB3b\_Genbank\_for\_inparanoid.fasta:1205

```
CQW49_RS15680       	100.00%		CQW49_15645         	100.00%
Bootstrap support for CQW49_RS15680 as seed ortholog is 100%.
Bootstrap support for CQW49_15645 as seed ortholog is 100%.
```

---

### Group of orthologs #373. Best score 1203 bits Score difference with first non-orthologous sequence - OB3b\_Refseq\_for\_inparanoid.fasta:1203 OB3b\_Genbank\_for\_inparanoid.fasta:1203

```
CQW49_RS06830       	100.00%		CQW49_06820         	100.00%
Bootstrap support for CQW49_RS06830 as seed ortholog is 100%.
Bootstrap support for CQW49_06820 as seed ortholog is 100%.
```

---

### Group of orthologs #374. Best score 1200 bits Score difference with first non-orthologous sequence - OB3b\_Refseq\_for\_inparanoid.fasta:1200 OB3b\_Genbank\_for\_inparanoid.fasta:1200

```
CQW49_RS05005       	100.00%		CQW49_05000         	100.00%
Bootstrap support for CQW49_RS05005 as seed ortholog is 100%.
Bootstrap support for CQW49_05000 as seed ortholog is 100%.
```

---

### Group of orthologs #375. Best score 1200 bits Score difference with first non-orthologous sequence - OB3b\_Refseq\_for\_inparanoid.fasta:1200 OB3b\_Genbank\_for\_inparanoid.fasta:1200

```
CQW49_RS19850       	100.00%		CQW49_19795         	100.00%
Bootstrap support for CQW49_RS19850 as seed ortholog is 100%.
Bootstrap support for CQW49_19795 as seed ortholog is 100%.
```

---

### Group of orthologs #376. Best score 1199 bits Score difference with first non-orthologous sequence - OB3b\_Refseq\_for\_inparanoid.fasta:1199 OB3b\_Genbank\_for\_inparanoid.fasta:1199

```
CQW49_RS09405       	100.00%		CQW49_09385         	100.00%
Bootstrap support for CQW49_RS09405 as seed ortholog is 100%.
Bootstrap support for CQW49_09385 as seed ortholog is 100%.
```

---

### Group of orthologs #377. Best score 1198 bits Score difference with first non-orthologous sequence - OB3b\_Refseq\_for\_inparanoid.fasta:1198 OB3b\_Genbank\_for\_inparanoid.fasta:1198

```
CQW49_RS04925       	100.00%		CQW49_04920         	100.00%
Bootstrap support for CQW49_RS04925 as seed ortholog is 100%.
Bootstrap support for CQW49_04920 as seed ortholog is 100%.
```

---

### Group of orthologs #378. Best score 1197 bits Score difference with first non-orthologous sequence - OB3b\_Refseq\_for\_inparanoid.fasta:1197 OB3b\_Genbank\_for\_inparanoid.fasta:1197

```
CQW49_RS17550       	100.00%		CQW49_17495         	100.00%
Bootstrap support for CQW49_RS17550 as seed ortholog is 100%.
Bootstrap support for CQW49_17495 as seed ortholog is 100%.
```

---

### Group of orthologs #379. Best score 1195 bits Score difference with first non-orthologous sequence - OB3b\_Refseq\_for\_inparanoid.fasta:1195 OB3b\_Genbank\_for\_inparanoid.fasta:1104

```
CQW49_RS00850       	100.00%		CQW49_00850         	100.00%
Bootstrap support for CQW49_RS00850 as seed ortholog is 100%.
Bootstrap support for CQW49_00850 as seed ortholog is 100%.
```

---

### Group of orthologs #380. Best score 1195 bits Score difference with first non-orthologous sequence - OB3b\_Refseq\_for\_inparanoid.fasta:1195 OB3b\_Genbank\_for\_inparanoid.fasta:1090

```
CQW49_RS02695       	100.00%		CQW49_02685         	100.00%
Bootstrap support for CQW49_RS02695 as seed ortholog is 100%.
Bootstrap support for CQW49_02685 as seed ortholog is 100%.
```

---

### Group of orthologs #381. Best score 1195 bits Score difference with first non-orthologous sequence - OB3b\_Refseq\_for\_inparanoid.fasta:1195 OB3b\_Genbank\_for\_inparanoid.fasta:1195

```
CQW49_RS09825       	100.00%		CQW49_09805         	100.00%
Bootstrap support for CQW49_RS09825 as seed ortholog is 100%.
Bootstrap support for CQW49_09805 as seed ortholog is 100%.
```

---

### Group of orthologs #382. Best score 1192 bits Score difference with first non-orthologous sequence - OB3b\_Refseq\_for\_inparanoid.fasta:1192 OB3b\_Genbank\_for\_inparanoid.fasta:1192

```
CQW49_RS15775       	100.00%		CQW49_15740         	100.00%
Bootstrap support for CQW49_RS15775 as seed ortholog is 100%.
Bootstrap support for CQW49_15740 as seed ortholog is 100%.
```

---

### Group of orthologs #383. Best score 1191 bits Score difference with first non-orthologous sequence - OB3b\_Refseq\_for\_inparanoid.fasta:1191 OB3b\_Genbank\_for\_inparanoid.fasta:1191

```
CQW49_RS20985       	100.00%		CQW49_20910         	100.00%
Bootstrap support for CQW49_RS20985 as seed ortholog is 100%.
Bootstrap support for CQW49_20910 as seed ortholog is 100%.
```

---

### Group of orthologs #384. Best score 1190 bits Score difference with first non-orthologous sequence - OB3b\_Refseq\_for\_inparanoid.fasta:1190 OB3b\_Genbank\_for\_inparanoid.fasta:1190

```
CQW49_RS08140       	100.00%		CQW49_08130         	100.00%
Bootstrap support for CQW49_RS08140 as seed ortholog is 100%.
Bootstrap support for CQW49_08130 as seed ortholog is 100%.
```

---

### Group of orthologs #385. Best score 1189 bits Score difference with first non-orthologous sequence - OB3b\_Refseq\_for\_inparanoid.fasta:1189 OB3b\_Genbank\_for\_inparanoid.fasta:1128

```
CQW49_RS18855       	100.00%		CQW49_18795         	100.00%
Bootstrap support for CQW49_RS18855 as seed ortholog is 100%.
Bootstrap support for CQW49_18795 as seed ortholog is 100%.
```

---

### Group of orthologs #386. Best score 1188 bits Score difference with first non-orthologous sequence - OB3b\_Refseq\_for\_inparanoid.fasta:1188 OB3b\_Genbank\_for\_inparanoid.fasta:1188

```
CQW49_RS11600       	100.00%		CQW49_11565         	100.00%
Bootstrap support for CQW49_RS11600 as seed ortholog is 100%.
Bootstrap support for CQW49_11565 as seed ortholog is 100%.
```

---

### Group of orthologs #387. Best score 1186 bits Score difference with first non-orthologous sequence - OB3b\_Refseq\_for\_inparanoid.fasta:1186 OB3b\_Genbank\_for\_inparanoid.fasta:1186

```
typA                	100.00%		CQW49_13015         	100.00%
Bootstrap support for typA as seed ortholog is 100%.
Bootstrap support for CQW49_13015 as seed ortholog is 100%.
```

---

### Group of orthologs #388. Best score 1185 bits Score difference with first non-orthologous sequence - OB3b\_Refseq\_for\_inparanoid.fasta:1185 OB3b\_Genbank\_for\_inparanoid.fasta:1185

```
CQW49_RS06660       	100.00%		CQW49_06650         	100.00%
Bootstrap support for CQW49_RS06660 as seed ortholog is 100%.
Bootstrap support for CQW49_06650 as seed ortholog is 100%.
```

---

### Group of orthologs #389. Best score 1185 bits Score difference with first non-orthologous sequence - OB3b\_Refseq\_for\_inparanoid.fasta:1185 OB3b\_Genbank\_for\_inparanoid.fasta:1185

```
CQW49_RS15525       	100.00%		CQW49_15490         	100.00%
Bootstrap support for CQW49_RS15525 as seed ortholog is 100%.
Bootstrap support for CQW49_15490 as seed ortholog is 100%.
```

---

### Group of orthologs #390. Best score 1185 bits Score difference with first non-orthologous sequence - OB3b\_Refseq\_for\_inparanoid.fasta:1185 OB3b\_Genbank\_for\_inparanoid.fasta:1185

```
CQW49_RS19005       	100.00%		CQW49_18945         	100.00%
Bootstrap support for CQW49_RS19005 as seed ortholog is 100%.
Bootstrap support for CQW49_18945 as seed ortholog is 100%.
```

---

### Group of orthologs #391. Best score 1183 bits Score difference with first non-orthologous sequence - OB3b\_Refseq\_for\_inparanoid.fasta:1183 OB3b\_Genbank\_for\_inparanoid.fasta:1183

```
CQW49_RS04230       	100.00%		CQW49_04220         	100.00%
Bootstrap support for CQW49_RS04230 as seed ortholog is 100%.
Bootstrap support for CQW49_04220 as seed ortholog is 100%.
```

---

### Group of orthologs #392. Best score 1183 bits Score difference with first non-orthologous sequence - OB3b\_Refseq\_for\_inparanoid.fasta:1183 OB3b\_Genbank\_for\_inparanoid.fasta:1183

```
CQW49_RS07100       	100.00%		CQW49_07090         	100.00%
Bootstrap support for CQW49_RS07100 as seed ortholog is 100%.
Bootstrap support for CQW49_07090 as seed ortholog is 100%.
```

---

### Group of orthologs #393. Best score 1183 bits Score difference with first non-orthologous sequence - OB3b\_Refseq\_for\_inparanoid.fasta:1183 OB3b\_Genbank\_for\_inparanoid.fasta:1069

```
CQW49_RS18520       	100.00%		CQW49_18460         	100.00%
Bootstrap support for CQW49_RS18520 as seed ortholog is 100%.
Bootstrap support for CQW49_18460 as seed ortholog is 100%.
```

---

### Group of orthologs #394. Best score 1180 bits Score difference with first non-orthologous sequence - OB3b\_Refseq\_for\_inparanoid.fasta:1180 OB3b\_Genbank\_for\_inparanoid.fasta:1180

```
CQW49_RS02390       	100.00%		CQW49_02380         	100.00%
Bootstrap support for CQW49_RS02390 as seed ortholog is 100%.
Bootstrap support for CQW49_02380 as seed ortholog is 100%.
```

---

### Group of orthologs #395. Best score 1180 bits Score difference with first non-orthologous sequence - OB3b\_Refseq\_for\_inparanoid.fasta:1180 OB3b\_Genbank\_for\_inparanoid.fasta:1180

```
CQW49_RS20045       	100.00%		CQW49_19980         	100.00%
Bootstrap support for CQW49_RS20045 as seed ortholog is 100%.
Bootstrap support for CQW49_19980 as seed ortholog is 100%.
```

---

### Group of orthologs #396. Best score 1178 bits Score difference with first non-orthologous sequence - OB3b\_Refseq\_for\_inparanoid.fasta:1178 OB3b\_Genbank\_for\_inparanoid.fasta:1178

```
recQ                	100.00%		CQW49_21185         	100.00%
Bootstrap support for recQ as seed ortholog is 100%.
Bootstrap support for CQW49_21185 as seed ortholog is 100%.
```

---

### Group of orthologs #397. Best score 1177 bits Score difference with first non-orthologous sequence - OB3b\_Refseq\_for\_inparanoid.fasta:1177 OB3b\_Genbank\_for\_inparanoid.fasta:1177

```
CQW49_RS14840       	100.00%		CQW49_14805         	100.00%
Bootstrap support for CQW49_RS14840 as seed ortholog is 100%.
Bootstrap support for CQW49_14805 as seed ortholog is 100%.
```

---

### Group of orthologs #398. Best score 1176 bits Score difference with first non-orthologous sequence - OB3b\_Refseq\_for\_inparanoid.fasta:1176 OB3b\_Genbank\_for\_inparanoid.fasta:1176

```
CQW49_RS12090       	100.00%		CQW49_12055         	100.00%
Bootstrap support for CQW49_RS12090 as seed ortholog is 100%.
Bootstrap support for CQW49_12055 as seed ortholog is 100%.
```

---

### Group of orthologs #399. Best score 1176 bits Score difference with first non-orthologous sequence - OB3b\_Refseq\_for\_inparanoid.fasta:1176 OB3b\_Genbank\_for\_inparanoid.fasta:1176

```
CQW49_RS18250       	100.00%		CQW49_18190         	100.00%
Bootstrap support for CQW49_RS18250 as seed ortholog is 100%.
Bootstrap support for CQW49_18190 as seed ortholog is 100%.
```

---

### Group of orthologs #400. Best score 1175 bits Score difference with first non-orthologous sequence - OB3b\_Refseq\_for\_inparanoid.fasta:1175 OB3b\_Genbank\_for\_inparanoid.fasta:1175

```
CQW49_RS15050       	100.00%		CQW49_15015         	100.00%
Bootstrap support for CQW49_RS15050 as seed ortholog is 100%.
Bootstrap support for CQW49_15015 as seed ortholog is 100%.
```

---

### Group of orthologs #401. Best score 1174 bits Score difference with first non-orthologous sequence - OB3b\_Refseq\_for\_inparanoid.fasta:1174 OB3b\_Genbank\_for\_inparanoid.fasta:1174

```
recJ                	100.00%		CQW49_01305         	100.00%
Bootstrap support for recJ as seed ortholog is 100%.
Bootstrap support for CQW49_01305 as seed ortholog is 100%.
```

---

### Group of orthologs #402. Best score 1174 bits Score difference with first non-orthologous sequence - OB3b\_Refseq\_for\_inparanoid.fasta:1174 OB3b\_Genbank\_for\_inparanoid.fasta:1174

```
glmS                	100.00%		CQW49_06600         	100.00%
Bootstrap support for glmS as seed ortholog is 100%.
Bootstrap support for CQW49_06600 as seed ortholog is 100%.
```

---

### Group of orthologs #403. Best score 1174 bits Score difference with first non-orthologous sequence - OB3b\_Refseq\_for\_inparanoid.fasta:1174 OB3b\_Genbank\_for\_inparanoid.fasta:1174

```
CQW49_RS12375       	100.00%		CQW49_12340         	100.00%
Bootstrap support for CQW49_RS12375 as seed ortholog is 100%.
Bootstrap support for CQW49_12340 as seed ortholog is 100%.
```

---

### Group of orthologs #404. Best score 1174 bits Score difference with first non-orthologous sequence - OB3b\_Refseq\_for\_inparanoid.fasta:1174 OB3b\_Genbank\_for\_inparanoid.fasta:1174

```
CQW49_RS20030       	100.00%		CQW49_19965         	100.00%
Bootstrap support for CQW49_RS20030 as seed ortholog is 100%.
Bootstrap support for CQW49_19965 as seed ortholog is 100%.
```

---

### Group of orthologs #405. Best score 1173 bits Score difference with first non-orthologous sequence - OB3b\_Refseq\_for\_inparanoid.fasta:1173 OB3b\_Genbank\_for\_inparanoid.fasta:1094

```
CQW49_RS02910       	100.00%		CQW49_02900         	100.00%
Bootstrap support for CQW49_RS02910 as seed ortholog is 100%.
Bootstrap support for CQW49_02900 as seed ortholog is 100%.
```

---

### Group of orthologs #406. Best score 1173 bits Score difference with first non-orthologous sequence - OB3b\_Refseq\_for\_inparanoid.fasta:1173 OB3b\_Genbank\_for\_inparanoid.fasta:1173

```
CQW49_RS14110       	100.00%		CQW49_14075         	100.00%
Bootstrap support for CQW49_RS14110 as seed ortholog is 100%.
Bootstrap support for CQW49_14075 as seed ortholog is 100%.
```

---

### Group of orthologs #407. Best score 1171 bits Score difference with first non-orthologous sequence - OB3b\_Refseq\_for\_inparanoid.fasta:1171 OB3b\_Genbank\_for\_inparanoid.fasta:1171

```
CQW49_RS11095       	100.00%		CQW49_11065         	100.00%
Bootstrap support for CQW49_RS11095 as seed ortholog is 100%.
Bootstrap support for CQW49_11065 as seed ortholog is 100%.
```

---

### Group of orthologs #408. Best score 1170 bits Score difference with first non-orthologous sequence - OB3b\_Refseq\_for\_inparanoid.fasta:1170 OB3b\_Genbank\_for\_inparanoid.fasta:1170

```
CQW49_RS00670       	100.00%		CQW49_00670         	100.00%
Bootstrap support for CQW49_RS00670 as seed ortholog is 100%.
Bootstrap support for CQW49_00670 as seed ortholog is 100%.
```

---

### Group of orthologs #409. Best score 1170 bits Score difference with first non-orthologous sequence - OB3b\_Refseq\_for\_inparanoid.fasta:1170 OB3b\_Genbank\_for\_inparanoid.fasta:1170

```
CQW49_RS06220       	100.00%		CQW49_06210         	100.00%
Bootstrap support for CQW49_RS06220 as seed ortholog is 100%.
Bootstrap support for CQW49_06210 as seed ortholog is 100%.
```

---

### Group of orthologs #410. Best score 1166 bits Score difference with first non-orthologous sequence - OB3b\_Refseq\_for\_inparanoid.fasta:1166 OB3b\_Genbank\_for\_inparanoid.fasta:1166

```
CQW49_RS12365       	100.00%		CQW49_12330         	100.00%
Bootstrap support for CQW49_RS12365 as seed ortholog is 100%.
Bootstrap support for CQW49_12330 as seed ortholog is 100%.
```

---

### Group of orthologs #411. Best score 1164 bits Score difference with first non-orthologous sequence - OB3b\_Refseq\_for\_inparanoid.fasta:1164 OB3b\_Genbank\_for\_inparanoid.fasta:466

```
CQW49_RS19420       	100.00%		CQW49_19365         	100.00%
Bootstrap support for CQW49_RS19420 as seed ortholog is 100%.
Bootstrap support for CQW49_19365 as seed ortholog is 100%.
```

---

### Group of orthologs #412. Best score 1163 bits Score difference with first non-orthologous sequence - OB3b\_Refseq\_for\_inparanoid.fasta:1163 OB3b\_Genbank\_for\_inparanoid.fasta:1163

```
CQW49_RS21070       	100.00%		CQW49_20995         	100.00%
Bootstrap support for CQW49_RS21070 as seed ortholog is 100%.
Bootstrap support for CQW49_20995 as seed ortholog is 100%.
```

---

### Group of orthologs #413. Best score 1159 bits Score difference with first non-orthologous sequence - OB3b\_Refseq\_for\_inparanoid.fasta:1159 OB3b\_Genbank\_for\_inparanoid.fasta:1159

```
CQW49_RS08185       	100.00%		CQW49_08175         	100.00%
Bootstrap support for CQW49_RS08185 as seed ortholog is 100%.
Bootstrap support for CQW49_08175 as seed ortholog is 100%.
```

---

### Group of orthologs #414. Best score 1159 bits Score difference with first non-orthologous sequence - OB3b\_Refseq\_for\_inparanoid.fasta:1159 OB3b\_Genbank\_for\_inparanoid.fasta:1159

```
CQW49_RS16785       	100.00%		CQW49_16745         	100.00%
Bootstrap support for CQW49_RS16785 as seed ortholog is 100%.
Bootstrap support for CQW49_16745 as seed ortholog is 100%.
```

---

### Group of orthologs #415. Best score 1157 bits Score difference with first non-orthologous sequence - OB3b\_Refseq\_for\_inparanoid.fasta:1157 OB3b\_Genbank\_for\_inparanoid.fasta:1157

```
CQW49_RS11230       	100.00%		CQW49_11200         	100.00%
Bootstrap support for CQW49_RS11230 as seed ortholog is 100%.
Bootstrap support for CQW49_11200 as seed ortholog is 100%.
```

---

### Group of orthologs #416. Best score 1156 bits Score difference with first non-orthologous sequence - OB3b\_Refseq\_for\_inparanoid.fasta:1156 OB3b\_Genbank\_for\_inparanoid.fasta:1156

```
CQW49_RS11885       	100.00%		CQW49_11850         	100.00%
Bootstrap support for CQW49_RS11885 as seed ortholog is 100%.
Bootstrap support for CQW49_11850 as seed ortholog is 100%.
```

---

### Group of orthologs #417. Best score 1155 bits Score difference with first non-orthologous sequence - OB3b\_Refseq\_for\_inparanoid.fasta:1155 OB3b\_Genbank\_for\_inparanoid.fasta:1155

```
CQW49_RS06785       	100.00%		CQW49_06775         	100.00%
Bootstrap support for CQW49_RS06785 as seed ortholog is 100%.
Bootstrap support for CQW49_06775 as seed ortholog is 100%.
```

---

### Group of orthologs #418. Best score 1155 bits Score difference with first non-orthologous sequence - OB3b\_Refseq\_for\_inparanoid.fasta:1155 OB3b\_Genbank\_for\_inparanoid.fasta:1155

```
nifA                	100.00%		CQW49_08815         	100.00%
Bootstrap support for nifA as seed ortholog is 100%.
Bootstrap support for CQW49_08815 as seed ortholog is 100%.
```

---

### Group of orthologs #419. Best score 1155 bits Score difference with first non-orthologous sequence - OB3b\_Refseq\_for\_inparanoid.fasta:1155 OB3b\_Genbank\_for\_inparanoid.fasta:1155

```
CQW49_RS18335       	100.00%		CQW49_18275         	100.00%
Bootstrap support for CQW49_RS18335 as seed ortholog is 100%.
Bootstrap support for CQW49_18275 as seed ortholog is 100%.
```

---

### Group of orthologs #420. Best score 1150 bits Score difference with first non-orthologous sequence - OB3b\_Refseq\_for\_inparanoid.fasta:1150 OB3b\_Genbank\_for\_inparanoid.fasta:1150

```
CQW49_RS05425       	100.00%		CQW49_05420         	100.00%
Bootstrap support for CQW49_RS05425 as seed ortholog is 100%.
Bootstrap support for CQW49_05420 as seed ortholog is 100%.
```

---

### Group of orthologs #421. Best score 1150 bits Score difference with first non-orthologous sequence - OB3b\_Refseq\_for\_inparanoid.fasta:1150 OB3b\_Genbank\_for\_inparanoid.fasta:1150

```
CQW49_RS05985       	100.00%		CQW49_05975         	100.00%
Bootstrap support for CQW49_RS05985 as seed ortholog is 100%.
Bootstrap support for CQW49_05975 as seed ortholog is 100%.
```

---

### Group of orthologs #422. Best score 1149 bits Score difference with first non-orthologous sequence - OB3b\_Refseq\_for\_inparanoid.fasta:1149 OB3b\_Genbank\_for\_inparanoid.fasta:1149

```
CQW49_RS05795       	100.00%		CQW49_05785         	100.00%
Bootstrap support for CQW49_RS05795 as seed ortholog is 100%.
Bootstrap support for CQW49_05785 as seed ortholog is 100%.
```

---

### Group of orthologs #423. Best score 1148 bits Score difference with first non-orthologous sequence - OB3b\_Refseq\_for\_inparanoid.fasta:1148 OB3b\_Genbank\_for\_inparanoid.fasta:1148

```
CQW49_RS02300       	100.00%		CQW49_02295         	100.00%
Bootstrap support for CQW49_RS02300 as seed ortholog is 100%.
Bootstrap support for CQW49_02295 as seed ortholog is 100%.
```

---

### Group of orthologs #424. Best score 1148 bits Score difference with first non-orthologous sequence - OB3b\_Refseq\_for\_inparanoid.fasta:1148 OB3b\_Genbank\_for\_inparanoid.fasta:1148

```
CQW49_RS09105       	100.00%		CQW49_09085         	100.00%
Bootstrap support for CQW49_RS09105 as seed ortholog is 100%.
Bootstrap support for CQW49_09085 as seed ortholog is 100%.
```

---

### Group of orthologs #425. Best score 1145 bits Score difference with first non-orthologous sequence - OB3b\_Refseq\_for\_inparanoid.fasta:1145 OB3b\_Genbank\_for\_inparanoid.fasta:1145

```
CQW49_RS00335       	100.00%		CQW49_00335         	100.00%
Bootstrap support for CQW49_RS00335 as seed ortholog is 100%.
Bootstrap support for CQW49_00335 as seed ortholog is 100%.
```

---

### Group of orthologs #426. Best score 1145 bits Score difference with first non-orthologous sequence - OB3b\_Refseq\_for\_inparanoid.fasta:1145 OB3b\_Genbank\_for\_inparanoid.fasta:1058

```
CQW49_RS15935       	100.00%		CQW49_15900         	100.00%
Bootstrap support for CQW49_RS15935 as seed ortholog is 100%.
Bootstrap support for CQW49_15900 as seed ortholog is 100%.
```

---

### Group of orthologs #427. Best score 1143 bits Score difference with first non-orthologous sequence - OB3b\_Refseq\_for\_inparanoid.fasta:1143 OB3b\_Genbank\_for\_inparanoid.fasta:1143

```
CQW49_RS04290       	100.00%		CQW49_04280         	100.00%
Bootstrap support for CQW49_RS04290 as seed ortholog is 100%.
Bootstrap support for CQW49_04280 as seed ortholog is 100%.
```

---

### Group of orthologs #428. Best score 1143 bits Score difference with first non-orthologous sequence - OB3b\_Refseq\_for\_inparanoid.fasta:1143 OB3b\_Genbank\_for\_inparanoid.fasta:1143

```
CQW49_RS09015       	100.00%		CQW49_09000         	100.00%
Bootstrap support for CQW49_RS09015 as seed ortholog is 100%.
Bootstrap support for CQW49_09000 as seed ortholog is 100%.
```

---

### Group of orthologs #429. Best score 1143 bits Score difference with first non-orthologous sequence - OB3b\_Refseq\_for\_inparanoid.fasta:1143 OB3b\_Genbank\_for\_inparanoid.fasta:1143

```
CQW49_RS19620       	100.00%		CQW49_19565         	100.00%
Bootstrap support for CQW49_RS19620 as seed ortholog is 100%.
Bootstrap support for CQW49_19565 as seed ortholog is 100%.
```

---

### Group of orthologs #430. Best score 1142 bits Score difference with first non-orthologous sequence - OB3b\_Refseq\_for\_inparanoid.fasta:1142 OB3b\_Genbank\_for\_inparanoid.fasta:1142

```
CQW49_RS01840       	100.00%		CQW49_01835         	100.00%
Bootstrap support for CQW49_RS01840 as seed ortholog is 100%.
Bootstrap support for CQW49_01835 as seed ortholog is 100%.
```

---

### Group of orthologs #431. Best score 1140 bits Score difference with first non-orthologous sequence - OB3b\_Refseq\_for\_inparanoid.fasta:1140 OB3b\_Genbank\_for\_inparanoid.fasta:1140

```
CQW49_RS06305       	100.00%		CQW49_06295         	100.00%
Bootstrap support for CQW49_RS06305 as seed ortholog is 100%.
Bootstrap support for CQW49_06295 as seed ortholog is 100%.
```

---

### Group of orthologs #432. Best score 1138 bits Score difference with first non-orthologous sequence - OB3b\_Refseq\_for\_inparanoid.fasta:1138 OB3b\_Genbank\_for\_inparanoid.fasta:1138

```
CQW49_RS08180       	100.00%		CQW49_08170         	100.00%
Bootstrap support for CQW49_RS08180 as seed ortholog is 100%.
Bootstrap support for CQW49_08170 as seed ortholog is 100%.
```

---

### Group of orthologs #433. Best score 1136 bits Score difference with first non-orthologous sequence - OB3b\_Refseq\_for\_inparanoid.fasta:1136 OB3b\_Genbank\_for\_inparanoid.fasta:1136

```
CQW49_RS12230       	100.00%		CQW49_12195         	100.00%
Bootstrap support for CQW49_RS12230 as seed ortholog is 100%.
Bootstrap support for CQW49_12195 as seed ortholog is 100%.
```

---

### Group of orthologs #434. Best score 1134 bits Score difference with first non-orthologous sequence - OB3b\_Refseq\_for\_inparanoid.fasta:1134 OB3b\_Genbank\_for\_inparanoid.fasta:1040

```
CQW49_RS01065       	100.00%		CQW49_01065         	100.00%
Bootstrap support for CQW49_RS01065 as seed ortholog is 100%.
Bootstrap support for CQW49_01065 as seed ortholog is 100%.
```

---

### Group of orthologs #435. Best score 1133 bits Score difference with first non-orthologous sequence - OB3b\_Refseq\_for\_inparanoid.fasta:1133 OB3b\_Genbank\_for\_inparanoid.fasta:1133

```
arsA                	100.00%		CQW49_11490         	100.00%
Bootstrap support for arsA as seed ortholog is 100%.
Bootstrap support for CQW49_11490 as seed ortholog is 100%.
```

---

### Group of orthologs #436. Best score 1131 bits Score difference with first non-orthologous sequence - OB3b\_Refseq\_for\_inparanoid.fasta:1131 OB3b\_Genbank\_for\_inparanoid.fasta:1131

```
CQW49_RS12345       	100.00%		CQW49_12310         	100.00%
Bootstrap support for CQW49_RS12345 as seed ortholog is 100%.
Bootstrap support for CQW49_12310 as seed ortholog is 100%.
```

---

### Group of orthologs #437. Best score 1130 bits Score difference with first non-orthologous sequence - OB3b\_Refseq\_for\_inparanoid.fasta:1130 OB3b\_Genbank\_for\_inparanoid.fasta:1130

```
CQW49_RS00390       	100.00%		CQW49_00390         	100.00%
Bootstrap support for CQW49_RS00390 as seed ortholog is 100%.
Bootstrap support for CQW49_00390 as seed ortholog is 100%.
```

---

### Group of orthologs #438. Best score 1129 bits Score difference with first non-orthologous sequence - OB3b\_Refseq\_for\_inparanoid.fasta:1129 OB3b\_Genbank\_for\_inparanoid.fasta:1129

```
CQW49_RS06165       	100.00%		CQW49_06155         	100.00%
Bootstrap support for CQW49_RS06165 as seed ortholog is 100%.
Bootstrap support for CQW49_06155 as seed ortholog is 100%.
```

---

### Group of orthologs #439. Best score 1129 bits Score difference with first non-orthologous sequence - OB3b\_Refseq\_for\_inparanoid.fasta:1129 OB3b\_Genbank\_for\_inparanoid.fasta:1129

```
CQW49_RS06845       	100.00%		CQW49_06835         	100.00%
Bootstrap support for CQW49_RS06845 as seed ortholog is 100%.
Bootstrap support for CQW49_06835 as seed ortholog is 100%.
```

---

### Group of orthologs #440. Best score 1128 bits Score difference with first non-orthologous sequence - OB3b\_Refseq\_for\_inparanoid.fasta:1128 OB3b\_Genbank\_for\_inparanoid.fasta:1128

```
CQW49_RS16360       	100.00%		CQW49_16325         	100.00%
Bootstrap support for CQW49_RS16360 as seed ortholog is 100%.
Bootstrap support for CQW49_16325 as seed ortholog is 100%.
```

---

### Group of orthologs #441. Best score 1127 bits Score difference with first non-orthologous sequence - OB3b\_Refseq\_for\_inparanoid.fasta:1127 OB3b\_Genbank\_for\_inparanoid.fasta:1127

```
CQW49_RS10785       	100.00%		CQW49_10755         	100.00%
Bootstrap support for CQW49_RS10785 as seed ortholog is 100%.
Bootstrap support for CQW49_10755 as seed ortholog is 100%.
```

---

### Group of orthologs #442. Best score 1126 bits Score difference with first non-orthologous sequence - OB3b\_Refseq\_for\_inparanoid.fasta:1126 OB3b\_Genbank\_for\_inparanoid.fasta:1126

```
CQW49_RS13835       	100.00%		CQW49_13800         	100.00%
Bootstrap support for CQW49_RS13835 as seed ortholog is 100%.
Bootstrap support for CQW49_13800 as seed ortholog is 100%.
```

---

### Group of orthologs #443. Best score 1124 bits Score difference with first non-orthologous sequence - OB3b\_Refseq\_for\_inparanoid.fasta:1124 OB3b\_Genbank\_for\_inparanoid.fasta:1040

```
CQW49_RS16600       	100.00%		CQW49_16560         	100.00%
Bootstrap support for CQW49_RS16600 as seed ortholog is 100%.
Bootstrap support for CQW49_16560 as seed ortholog is 100%.
```

---

### Group of orthologs #444. Best score 1122 bits Score difference with first non-orthologous sequence - OB3b\_Refseq\_for\_inparanoid.fasta:1122 OB3b\_Genbank\_for\_inparanoid.fasta:1122

```
CQW49_RS17465       	100.00%		CQW49_17410         	100.00%
Bootstrap support for CQW49_RS17465 as seed ortholog is 100%.
Bootstrap support for CQW49_17410 as seed ortholog is 100%.
```

---

### Group of orthologs #445. Best score 1119 bits Score difference with first non-orthologous sequence - OB3b\_Refseq\_for\_inparanoid.fasta:1119 OB3b\_Genbank\_for\_inparanoid.fasta:1119

```
CQW49_RS11300       	100.00%		CQW49_11270         	100.00%
Bootstrap support for CQW49_RS11300 as seed ortholog is 100%.
Bootstrap support for CQW49_11270 as seed ortholog is 100%.
```

---

### Group of orthologs #446. Best score 1117 bits Score difference with first non-orthologous sequence - OB3b\_Refseq\_for\_inparanoid.fasta:1117 OB3b\_Genbank\_for\_inparanoid.fasta:1117

```
CQW49_RS07825       	100.00%		CQW49_07815         	100.00%
Bootstrap support for CQW49_RS07825 as seed ortholog is 100%.
Bootstrap support for CQW49_07815 as seed ortholog is 100%.
```

---

### Group of orthologs #447. Best score 1117 bits Score difference with first non-orthologous sequence - OB3b\_Refseq\_for\_inparanoid.fasta:1117 OB3b\_Genbank\_for\_inparanoid.fasta:1057

```
CQW49_RS08725       	100.00%		CQW49_08715         	100.00%
Bootstrap support for CQW49_RS08725 as seed ortholog is 100%.
Bootstrap support for CQW49_08715 as seed ortholog is 100%.
```

---

### Group of orthologs #448. Best score 1115 bits Score difference with first non-orthologous sequence - OB3b\_Refseq\_for\_inparanoid.fasta:1115 OB3b\_Genbank\_for\_inparanoid.fasta:1115

```
CQW49_RS09225       	100.00%		CQW49_09205         	100.00%
Bootstrap support for CQW49_RS09225 as seed ortholog is 100%.
Bootstrap support for CQW49_09205 as seed ortholog is 100%.
```

---

### Group of orthologs #449. Best score 1114 bits Score difference with first non-orthologous sequence - OB3b\_Refseq\_for\_inparanoid.fasta:1114 OB3b\_Genbank\_for\_inparanoid.fasta:1114

```
CQW49_RS03160       	100.00%		CQW49_03150         	100.00%
Bootstrap support for CQW49_RS03160 as seed ortholog is 100%.
Bootstrap support for CQW49_03150 as seed ortholog is 100%.
```

---

### Group of orthologs #450. Best score 1114 bits Score difference with first non-orthologous sequence - OB3b\_Refseq\_for\_inparanoid.fasta:1114 OB3b\_Genbank\_for\_inparanoid.fasta:1114

```
CQW49_RS08090       	100.00%		CQW49_08080         	100.00%
Bootstrap support for CQW49_RS08090 as seed ortholog is 100%.
Bootstrap support for CQW49_08080 as seed ortholog is 100%.
```

---

### Group of orthologs #451. Best score 1112 bits Score difference with first non-orthologous sequence - OB3b\_Refseq\_for\_inparanoid.fasta:1112 OB3b\_Genbank\_for\_inparanoid.fasta:1112

```
CQW49_RS00770       	100.00%		CQW49_00770         	100.00%
Bootstrap support for CQW49_RS00770 as seed ortholog is 100%.
Bootstrap support for CQW49_00770 as seed ortholog is 100%.
```

---

### Group of orthologs #452. Best score 1112 bits Score difference with first non-orthologous sequence - OB3b\_Refseq\_for\_inparanoid.fasta:1112 OB3b\_Genbank\_for\_inparanoid.fasta:1009

```
CQW49_RS01685       	100.00%		CQW49_01680         	100.00%
Bootstrap support for CQW49_RS01685 as seed ortholog is 100%.
Bootstrap support for CQW49_01680 as seed ortholog is 100%.
```

---

### Group of orthologs #453. Best score 1112 bits Score difference with first non-orthologous sequence - OB3b\_Refseq\_for\_inparanoid.fasta:1112 OB3b\_Genbank\_for\_inparanoid.fasta:1112

```
CQW49_RS12515       	100.00%		CQW49_12480         	100.00%
Bootstrap support for CQW49_RS12515 as seed ortholog is 100%.
Bootstrap support for CQW49_12480 as seed ortholog is 100%.
```

---

### Group of orthologs #454. Best score 1107 bits Score difference with first non-orthologous sequence - OB3b\_Refseq\_for\_inparanoid.fasta:1107 OB3b\_Genbank\_for\_inparanoid.fasta:1107

```
CQW49_RS20650       	100.00%		CQW49_20575         	100.00%
Bootstrap support for CQW49_RS20650 as seed ortholog is 100%.
Bootstrap support for CQW49_20575 as seed ortholog is 100%.
```

---

### Group of orthologs #455. Best score 1106 bits Score difference with first non-orthologous sequence - OB3b\_Refseq\_for\_inparanoid.fasta:1106 OB3b\_Genbank\_for\_inparanoid.fasta:1106

```
CQW49_RS01755       	100.00%		CQW49_01750         	100.00%
Bootstrap support for CQW49_RS01755 as seed ortholog is 100%.
Bootstrap support for CQW49_01750 as seed ortholog is 100%.
```

---

### Group of orthologs #456. Best score 1106 bits Score difference with first non-orthologous sequence - OB3b\_Refseq\_for\_inparanoid.fasta:1106 OB3b\_Genbank\_for\_inparanoid.fasta:1106

```
CQW49_RS04845       	100.00%		CQW49_04835         	100.00%
Bootstrap support for CQW49_RS04845 as seed ortholog is 100%.
Bootstrap support for CQW49_04835 as seed ortholog is 100%.
```

---

### Group of orthologs #457. Best score 1106 bits Score difference with first non-orthologous sequence - OB3b\_Refseq\_for\_inparanoid.fasta:1106 OB3b\_Genbank\_for\_inparanoid.fasta:1106

```
CQW49_RS09715       	100.00%		CQW49_09695         	100.00%
Bootstrap support for CQW49_RS09715 as seed ortholog is 100%.
Bootstrap support for CQW49_09695 as seed ortholog is 100%.
```

---

### Group of orthologs #458. Best score 1104 bits Score difference with first non-orthologous sequence - OB3b\_Refseq\_for\_inparanoid.fasta:1104 OB3b\_Genbank\_for\_inparanoid.fasta:1104

```
CQW49_RS05280       	100.00%		CQW49_05275         	100.00%
Bootstrap support for CQW49_RS05280 as seed ortholog is 100%.
Bootstrap support for CQW49_05275 as seed ortholog is 100%.
```

---

### Group of orthologs #459. Best score 1104 bits Score difference with first non-orthologous sequence - OB3b\_Refseq\_for\_inparanoid.fasta:1104 OB3b\_Genbank\_for\_inparanoid.fasta:1104

```
CQW49_RS05400       	100.00%		CQW49_05395         	100.00%
Bootstrap support for CQW49_RS05400 as seed ortholog is 100%.
Bootstrap support for CQW49_05395 as seed ortholog is 100%.
```

---

### Group of orthologs #460. Best score 1104 bits Score difference with first non-orthologous sequence - OB3b\_Refseq\_for\_inparanoid.fasta:1104 OB3b\_Genbank\_for\_inparanoid.fasta:1008

```
CQW49_RS06480       	100.00%		CQW49_06470         	100.00%
Bootstrap support for CQW49_RS06480 as seed ortholog is 100%.
Bootstrap support for CQW49_06470 as seed ortholog is 100%.
```

---

### Group of orthologs #461. Best score 1104 bits Score difference with first non-orthologous sequence - OB3b\_Refseq\_for\_inparanoid.fasta:1104 OB3b\_Genbank\_for\_inparanoid.fasta:1104

```
CQW49_RS19345       	100.00%		CQW49_19290         	100.00%
Bootstrap support for CQW49_RS19345 as seed ortholog is 100%.
Bootstrap support for CQW49_19290 as seed ortholog is 100%.
```

---

### Group of orthologs #462. Best score 1101 bits Score difference with first non-orthologous sequence - OB3b\_Refseq\_for\_inparanoid.fasta:1101 OB3b\_Genbank\_for\_inparanoid.fasta:1101

```
CQW49_RS15020       	100.00%		CQW49_14985         	100.00%
Bootstrap support for CQW49_RS15020 as seed ortholog is 100%.
Bootstrap support for CQW49_14985 as seed ortholog is 100%.
```

---

### Group of orthologs #463. Best score 1099 bits Score difference with first non-orthologous sequence - OB3b\_Refseq\_for\_inparanoid.fasta:1099 OB3b\_Genbank\_for\_inparanoid.fasta:1099

```
CQW49_RS06130       	100.00%		CQW49_06120         	100.00%
Bootstrap support for CQW49_RS06130 as seed ortholog is 100%.
Bootstrap support for CQW49_06120 as seed ortholog is 100%.
```

---

### Group of orthologs #464. Best score 1099 bits Score difference with first non-orthologous sequence - OB3b\_Refseq\_for\_inparanoid.fasta:1099 OB3b\_Genbank\_for\_inparanoid.fasta:1099

```
CQW49_RS19385       	100.00%		CQW49_19330         	100.00%
Bootstrap support for CQW49_RS19385 as seed ortholog is 100%.
Bootstrap support for CQW49_19330 as seed ortholog is 100%.
```

---

### Group of orthologs #465. Best score 1098 bits Score difference with first non-orthologous sequence - OB3b\_Refseq\_for\_inparanoid.fasta:1098 OB3b\_Genbank\_for\_inparanoid.fasta:1098

```
CQW49_RS10650       	100.00%		CQW49_10620         	100.00%
Bootstrap support for CQW49_RS10650 as seed ortholog is 100%.
Bootstrap support for CQW49_10620 as seed ortholog is 100%.
```

---

### Group of orthologs #466. Best score 1097 bits Score difference with first non-orthologous sequence - OB3b\_Refseq\_for\_inparanoid.fasta:1097 OB3b\_Genbank\_for\_inparanoid.fasta:1097

```
CQW49_RS04455       	100.00%		CQW49_04450         	100.00%
Bootstrap support for CQW49_RS04455 as seed ortholog is 100%.
Bootstrap support for CQW49_04450 as seed ortholog is 100%.
```

---

### Group of orthologs #467. Best score 1094 bits Score difference with first non-orthologous sequence - OB3b\_Refseq\_for\_inparanoid.fasta:1094 OB3b\_Genbank\_for\_inparanoid.fasta:1094

```
CQW49_RS19000       	100.00%		CQW49_18940         	100.00%
Bootstrap support for CQW49_RS19000 as seed ortholog is 100%.
Bootstrap support for CQW49_18940 as seed ortholog is 100%.
```

---

### Group of orthologs #468. Best score 1092 bits Score difference with first non-orthologous sequence - OB3b\_Refseq\_for\_inparanoid.fasta:1092 OB3b\_Genbank\_for\_inparanoid.fasta:1092

```
CQW49_RS03840       	100.00%		CQW49_03830         	100.00%
Bootstrap support for CQW49_RS03840 as seed ortholog is 100%.
Bootstrap support for CQW49_03830 as seed ortholog is 100%.
```

---

### Group of orthologs #469. Best score 1092 bits Score difference with first non-orthologous sequence - OB3b\_Refseq\_for\_inparanoid.fasta:1092 OB3b\_Genbank\_for\_inparanoid.fasta:1092

```
CQW49_RS05560       	100.00%		CQW49_05550         	100.00%
Bootstrap support for CQW49_RS05560 as seed ortholog is 100%.
Bootstrap support for CQW49_05550 as seed ortholog is 100%.
```

---

### Group of orthologs #470. Best score 1092 bits Score difference with first non-orthologous sequence - OB3b\_Refseq\_for\_inparanoid.fasta:1092 OB3b\_Genbank\_for\_inparanoid.fasta:1092

```
CQW49_RS07685       	100.00%		CQW49_07675         	100.00%
Bootstrap support for CQW49_RS07685 as seed ortholog is 100%.
Bootstrap support for CQW49_07675 as seed ortholog is 100%.
```

---

### Group of orthologs #471. Best score 1092 bits Score difference with first non-orthologous sequence - OB3b\_Refseq\_for\_inparanoid.fasta:1092 OB3b\_Genbank\_for\_inparanoid.fasta:1092

```
CQW49_RS10195       	100.00%		CQW49_10170         	100.00%
Bootstrap support for CQW49_RS10195 as seed ortholog is 100%.
Bootstrap support for CQW49_10170 as seed ortholog is 100%.
```

---

### Group of orthologs #472. Best score 1091 bits Score difference with first non-orthologous sequence - OB3b\_Refseq\_for\_inparanoid.fasta:1091 OB3b\_Genbank\_for\_inparanoid.fasta:1091

```
ggt                 	100.00%		CQW49_02400         	100.00%
Bootstrap support for ggt as seed ortholog is 100%.
Bootstrap support for CQW49_02400 as seed ortholog is 100%.
```

---

### Group of orthologs #473. Best score 1087 bits Score difference with first non-orthologous sequence - OB3b\_Refseq\_for\_inparanoid.fasta:1087 OB3b\_Genbank\_for\_inparanoid.fasta:1087

```
CQW49_RS03065       	100.00%		CQW49_03055         	100.00%
Bootstrap support for CQW49_RS03065 as seed ortholog is 100%.
Bootstrap support for CQW49_03055 as seed ortholog is 100%.
```

---

### Group of orthologs #474. Best score 1086 bits Score difference with first non-orthologous sequence - OB3b\_Refseq\_for\_inparanoid.fasta:1086 OB3b\_Genbank\_for\_inparanoid.fasta:1086

```
CQW49_RS04480       	100.00%		CQW49_04475         	100.00%
Bootstrap support for CQW49_RS04480 as seed ortholog is 100%.
Bootstrap support for CQW49_04475 as seed ortholog is 100%.
```

---

### Group of orthologs #475. Best score 1086 bits Score difference with first non-orthologous sequence - OB3b\_Refseq\_for\_inparanoid.fasta:1086 OB3b\_Genbank\_for\_inparanoid.fasta:1086

```
CQW49_RS09755       	100.00%		CQW49_09735         	100.00%
Bootstrap support for CQW49_RS09755 as seed ortholog is 100%.
Bootstrap support for CQW49_09735 as seed ortholog is 100%.
```

---

### Group of orthologs #476. Best score 1084 bits Score difference with first non-orthologous sequence - OB3b\_Refseq\_for\_inparanoid.fasta:1084 OB3b\_Genbank\_for\_inparanoid.fasta:1084

```
CQW49_RS16385       	100.00%		CQW49_16350         	100.00%
Bootstrap support for CQW49_RS16385 as seed ortholog is 100%.
Bootstrap support for CQW49_16350 as seed ortholog is 100%.
```

---

### Group of orthologs #477. Best score 1082 bits Score difference with first non-orthologous sequence - OB3b\_Refseq\_for\_inparanoid.fasta:1082 OB3b\_Genbank\_for\_inparanoid.fasta:1082

```
CQW49_RS12520       	100.00%		CQW49_12485         	100.00%
Bootstrap support for CQW49_RS12520 as seed ortholog is 100%.
Bootstrap support for CQW49_12485 as seed ortholog is 100%.
```

---

### Group of orthologs #478. Best score 1081 bits Score difference with first non-orthologous sequence - OB3b\_Refseq\_for\_inparanoid.fasta:1081 OB3b\_Genbank\_for\_inparanoid.fasta:1081

```
CQW49_RS01725       	100.00%		CQW49_01720         	100.00%
Bootstrap support for CQW49_RS01725 as seed ortholog is 100%.
Bootstrap support for CQW49_01720 as seed ortholog is 100%.
```

---

### Group of orthologs #479. Best score 1080 bits Score difference with first non-orthologous sequence - OB3b\_Refseq\_for\_inparanoid.fasta:1080 OB3b\_Genbank\_for\_inparanoid.fasta:1080

```
CQW49_RS09195       	100.00%		CQW49_09175         	100.00%
Bootstrap support for CQW49_RS09195 as seed ortholog is 100%.
Bootstrap support for CQW49_09175 as seed ortholog is 100%.
```

---

### Group of orthologs #480. Best score 1079 bits Score difference with first non-orthologous sequence - OB3b\_Refseq\_for\_inparanoid.fasta:1079 OB3b\_Genbank\_for\_inparanoid.fasta:1079

```
CQW49_RS09965       	100.00%		CQW49_09940         	100.00%
Bootstrap support for CQW49_RS09965 as seed ortholog is 100%.
Bootstrap support for CQW49_09940 as seed ortholog is 100%.
```

---

### Group of orthologs #481. Best score 1079 bits Score difference with first non-orthologous sequence - OB3b\_Refseq\_for\_inparanoid.fasta:1079 OB3b\_Genbank\_for\_inparanoid.fasta:1079

```
CQW49_RS10250       	100.00%		CQW49_10225         	100.00%
Bootstrap support for CQW49_RS10250 as seed ortholog is 100%.
Bootstrap support for CQW49_10225 as seed ortholog is 100%.
```

---

### Group of orthologs #482. Best score 1079 bits Score difference with first non-orthologous sequence - OB3b\_Refseq\_for\_inparanoid.fasta:1079 OB3b\_Genbank\_for\_inparanoid.fasta:1079

```
CQW49_RS12350       	100.00%		CQW49_12315         	100.00%
Bootstrap support for CQW49_RS12350 as seed ortholog is 100%.
Bootstrap support for CQW49_12315 as seed ortholog is 100%.
```

---

### Group of orthologs #483. Best score 1077 bits Score difference with first non-orthologous sequence - OB3b\_Refseq\_for\_inparanoid.fasta:1077 OB3b\_Genbank\_for\_inparanoid.fasta:1077

```
CQW49_RS03190       	100.00%		CQW49_03180         	100.00%
Bootstrap support for CQW49_RS03190 as seed ortholog is 100%.
Bootstrap support for CQW49_03180 as seed ortholog is 100%.
```

---

### Group of orthologs #484. Best score 1077 bits Score difference with first non-orthologous sequence - OB3b\_Refseq\_for\_inparanoid.fasta:1077 OB3b\_Genbank\_for\_inparanoid.fasta:759

```
CQW49_RS04475       	100.00%		CQW49_04470         	100.00%
Bootstrap support for CQW49_RS04475 as seed ortholog is 100%.
Bootstrap support for CQW49_04470 as seed ortholog is 100%.
```

---

### Group of orthologs #485. Best score 1077 bits Score difference with first non-orthologous sequence - OB3b\_Refseq\_for\_inparanoid.fasta:1077 OB3b\_Genbank\_for\_inparanoid.fasta:1077

```
nifK                	100.00%		CQW49_08720         	100.00%
Bootstrap support for nifK as seed ortholog is 100%.
Bootstrap support for CQW49_08720 as seed ortholog is 100%.
```

---

### Group of orthologs #486. Best score 1076 bits Score difference with first non-orthologous sequence - OB3b\_Refseq\_for\_inparanoid.fasta:1076 OB3b\_Genbank\_for\_inparanoid.fasta:1076

```
CQW49_RS05520       	100.00%		CQW49_05510         	100.00%
Bootstrap support for CQW49_RS05520 as seed ortholog is 100%.
Bootstrap support for CQW49_05510 as seed ortholog is 100%.
```

---

### Group of orthologs #487. Best score 1071 bits Score difference with first non-orthologous sequence - OB3b\_Refseq\_for\_inparanoid.fasta:1071 OB3b\_Genbank\_for\_inparanoid.fasta:1071

```
CQW49_RS01165       	100.00%		CQW49_01160         	100.00%
Bootstrap support for CQW49_RS01165 as seed ortholog is 100%.
Bootstrap support for CQW49_01160 as seed ortholog is 100%.
```

---

### Group of orthologs #488. Best score 1069 bits Score difference with first non-orthologous sequence - OB3b\_Refseq\_for\_inparanoid.fasta:1069 OB3b\_Genbank\_for\_inparanoid.fasta:789

```
CQW49_RS08050       	100.00%		CQW49_08040         	100.00%
CQW49_RS14635       	100.00%		CQW49_14600         	100.00%
CQW49_RS15350       	100.00%		CQW49_15315         	100.00%
CQW49_RS05860       	100.00%		CQW49_23650         	100.00%
                    	       		CQW49_05850         	100.00%
Bootstrap support for CQW49_RS08050 as seed ortholog is 100%.
Bootstrap support for CQW49_RS14635 as seed ortholog is 100%.
Bootstrap support for CQW49_RS15350 as seed ortholog is 100%.
Bootstrap support for CQW49_RS05860 as seed ortholog is 100%.
Bootstrap support for CQW49_08040 as seed ortholog is 100%.
Bootstrap support for CQW49_14600 as seed ortholog is 100%.
Bootstrap support for CQW49_15315 as seed ortholog is 100%.
Bootstrap support for CQW49_23650 as seed ortholog is 100%.
Bootstrap support for CQW49_05850 as seed ortholog is 100%.
```

---

### Group of orthologs #489. Best score 1069 bits Score difference with first non-orthologous sequence - OB3b\_Refseq\_for\_inparanoid.fasta:1069 OB3b\_Genbank\_for\_inparanoid.fasta:1069

```
CQW49_RS15410       	100.00%		CQW49_15375         	100.00%
Bootstrap support for CQW49_RS15410 as seed ortholog is 100%.
Bootstrap support for CQW49_15375 as seed ortholog is 100%.
```

---

### Group of orthologs #490. Best score 1067 bits Score difference with first non-orthologous sequence - OB3b\_Refseq\_for\_inparanoid.fasta:1067 OB3b\_Genbank\_for\_inparanoid.fasta:312

```
CQW49_RS08075       	100.00%		CQW49_08065         	100.00%
Bootstrap support for CQW49_RS08075 as seed ortholog is 100%.
Bootstrap support for CQW49_08065 as seed ortholog is 100%.
```

---

### Group of orthologs #491. Best score 1066 bits Score difference with first non-orthologous sequence - OB3b\_Refseq\_for\_inparanoid.fasta:1066 OB3b\_Genbank\_for\_inparanoid.fasta:1066

```
CQW49_RS10220       	100.00%		CQW49_10195         	100.00%
Bootstrap support for CQW49_RS10220 as seed ortholog is 100%.
Bootstrap support for CQW49_10195 as seed ortholog is 100%.
```

---

### Group of orthologs #492. Best score 1065 bits Score difference with first non-orthologous sequence - OB3b\_Refseq\_for\_inparanoid.fasta:1065 OB3b\_Genbank\_for\_inparanoid.fasta:1065

```
CQW49_RS11175       	100.00%		CQW49_11145         	100.00%
Bootstrap support for CQW49_RS11175 as seed ortholog is 100%.
Bootstrap support for CQW49_11145 as seed ortholog is 100%.
```

---

### Group of orthologs #493. Best score 1064 bits Score difference with first non-orthologous sequence - OB3b\_Refseq\_for\_inparanoid.fasta:1064 OB3b\_Genbank\_for\_inparanoid.fasta:959

```
CQW49_RS13415       	100.00%		CQW49_13380         	100.00%
Bootstrap support for CQW49_RS13415 as seed ortholog is 100%.
Bootstrap support for CQW49_13380 as seed ortholog is 100%.
```

---

### Group of orthologs #494. Best score 1063 bits Score difference with first non-orthologous sequence - OB3b\_Refseq\_for\_inparanoid.fasta:1063 OB3b\_Genbank\_for\_inparanoid.fasta:969

```
CQW49_RS01925       	100.00%		CQW49_01920         	100.00%
Bootstrap support for CQW49_RS01925 as seed ortholog is 100%.
Bootstrap support for CQW49_01920 as seed ortholog is 100%.
```

---

### Group of orthologs #495. Best score 1063 bits Score difference with first non-orthologous sequence - OB3b\_Refseq\_for\_inparanoid.fasta:1063 OB3b\_Genbank\_for\_inparanoid.fasta:1063

```
CQW49_RS20850       	100.00%		CQW49_20775         	100.00%
Bootstrap support for CQW49_RS20850 as seed ortholog is 100%.
Bootstrap support for CQW49_20775 as seed ortholog is 100%.
```

---

### Group of orthologs #496. Best score 1061 bits Score difference with first non-orthologous sequence - OB3b\_Refseq\_for\_inparanoid.fasta:1061 OB3b\_Genbank\_for\_inparanoid.fasta:1061

```
CQW49_RS17840       	100.00%		CQW49_17785         	100.00%
Bootstrap support for CQW49_RS17840 as seed ortholog is 100%.
Bootstrap support for CQW49_17785 as seed ortholog is 100%.
```

---

### Group of orthologs #497. Best score 1060 bits Score difference with first non-orthologous sequence - OB3b\_Refseq\_for\_inparanoid.fasta:1060 OB3b\_Genbank\_for\_inparanoid.fasta:1060

```
CQW49_RS00355       	100.00%		CQW49_00355         	100.00%
Bootstrap support for CQW49_RS00355 as seed ortholog is 100%.
Bootstrap support for CQW49_00355 as seed ortholog is 100%.
```

---

### Group of orthologs #498. Best score 1060 bits Score difference with first non-orthologous sequence - OB3b\_Refseq\_for\_inparanoid.fasta:1060 OB3b\_Genbank\_for\_inparanoid.fasta:1060

```
CQW49_RS21195       	100.00%		CQW49_21120         	100.00%
Bootstrap support for CQW49_RS21195 as seed ortholog is 100%.
Bootstrap support for CQW49_21120 as seed ortholog is 100%.
```

---

### Group of orthologs #499. Best score 1059 bits Score difference with first non-orthologous sequence - OB3b\_Refseq\_for\_inparanoid.fasta:1059 OB3b\_Genbank\_for\_inparanoid.fasta:1059

```
CQW49_RS12990       	100.00%		CQW49_12955         	100.00%
Bootstrap support for CQW49_RS12990 as seed ortholog is 100%.
Bootstrap support for CQW49_12955 as seed ortholog is 100%.
```

---

### Group of orthologs #500. Best score 1057 bits Score difference with first non-orthologous sequence - OB3b\_Refseq\_for\_inparanoid.fasta:1057 OB3b\_Genbank\_for\_inparanoid.fasta:1057

```
CQW49_RS06710       	100.00%		CQW49_06700         	100.00%
Bootstrap support for CQW49_RS06710 as seed ortholog is 100%.
Bootstrap support for CQW49_06700 as seed ortholog is 100%.
```

---

### Group of orthologs #501. Best score 1057 bits Score difference with first non-orthologous sequence - OB3b\_Refseq\_for\_inparanoid.fasta:1057 OB3b\_Genbank\_for\_inparanoid.fasta:1057

```
CQW49_RS10920       	100.00%		CQW49_10890         	100.00%
Bootstrap support for CQW49_RS10920 as seed ortholog is 100%.
Bootstrap support for CQW49_10890 as seed ortholog is 100%.
```

---

### Group of orthologs #502. Best score 1056 bits Score difference with first non-orthologous sequence - OB3b\_Refseq\_for\_inparanoid.fasta:1056 OB3b\_Genbank\_for\_inparanoid.fasta:1056

```
CQW49_RS08315       	100.00%		CQW49_08305         	100.00%
Bootstrap support for CQW49_RS08315 as seed ortholog is 100%.
Bootstrap support for CQW49_08305 as seed ortholog is 100%.
```

---

### Group of orthologs #503. Best score 1056 bits Score difference with first non-orthologous sequence - OB3b\_Refseq\_for\_inparanoid.fasta:1056 OB3b\_Genbank\_for\_inparanoid.fasta:1056

```
recN                	100.00%		CQW49_17800         	100.00%
Bootstrap support for recN as seed ortholog is 100%.
Bootstrap support for CQW49_17800 as seed ortholog is 100%.
```

---

### Group of orthologs #504. Best score 1055 bits Score difference with first non-orthologous sequence - OB3b\_Refseq\_for\_inparanoid.fasta:1055 OB3b\_Genbank\_for\_inparanoid.fasta:1055

```
nifB                	100.00%		CQW49_08805         	100.00%
Bootstrap support for nifB as seed ortholog is 100%.
Bootstrap support for CQW49_08805 as seed ortholog is 100%.
```

---

### Group of orthologs #505. Best score 1055 bits Score difference with first non-orthologous sequence - OB3b\_Refseq\_for\_inparanoid.fasta:1055 OB3b\_Genbank\_for\_inparanoid.fasta:1055

```
CQW49_RS11055       	100.00%		CQW49_11025         	100.00%
Bootstrap support for CQW49_RS11055 as seed ortholog is 100%.
Bootstrap support for CQW49_11025 as seed ortholog is 100%.
```

---

### Group of orthologs #506. Best score 1054 bits Score difference with first non-orthologous sequence - OB3b\_Refseq\_for\_inparanoid.fasta:1054 OB3b\_Genbank\_for\_inparanoid.fasta:1054

```
fliF                	100.00%		CQW49_18640         	100.00%
Bootstrap support for fliF as seed ortholog is 100%.
Bootstrap support for CQW49_18640 as seed ortholog is 100%.
```

---

### Group of orthologs #507. Best score 1053 bits Score difference with first non-orthologous sequence - OB3b\_Refseq\_for\_inparanoid.fasta:1053 OB3b\_Genbank\_for\_inparanoid.fasta:1053

```
CQW49_RS18450       	100.00%		CQW49_18390         	100.00%
Bootstrap support for CQW49_RS18450 as seed ortholog is 100%.
Bootstrap support for CQW49_18390 as seed ortholog is 100%.
```

---

### Group of orthologs #508. Best score 1052 bits Score difference with first non-orthologous sequence - OB3b\_Refseq\_for\_inparanoid.fasta:1052 OB3b\_Genbank\_for\_inparanoid.fasta:1052

```
CQW49_RS03610       	100.00%		CQW49_03600         	100.00%
Bootstrap support for CQW49_RS03610 as seed ortholog is 100%.
Bootstrap support for CQW49_03600 as seed ortholog is 100%.
```

---

### Group of orthologs #509. Best score 1052 bits Score difference with first non-orthologous sequence - OB3b\_Refseq\_for\_inparanoid.fasta:1052 OB3b\_Genbank\_for\_inparanoid.fasta:1052

```
CQW49_RS06840       	100.00%		CQW49_06830         	100.00%
Bootstrap support for CQW49_RS06840 as seed ortholog is 100%.
Bootstrap support for CQW49_06830 as seed ortholog is 100%.
```

---

### Group of orthologs #510. Best score 1050 bits Score difference with first non-orthologous sequence - OB3b\_Refseq\_for\_inparanoid.fasta:1050 OB3b\_Genbank\_for\_inparanoid.fasta:1050

```
CQW49_RS09735       	100.00%		CQW49_09715         	100.00%
Bootstrap support for CQW49_RS09735 as seed ortholog is 100%.
Bootstrap support for CQW49_09715 as seed ortholog is 100%.
```

---

### Group of orthologs #511. Best score 1048 bits Score difference with first non-orthologous sequence - OB3b\_Refseq\_for\_inparanoid.fasta:1048 OB3b\_Genbank\_for\_inparanoid.fasta:1048

```
CQW49_RS06075       	100.00%		CQW49_06065         	100.00%
Bootstrap support for CQW49_RS06075 as seed ortholog is 100%.
Bootstrap support for CQW49_06065 as seed ortholog is 100%.
```

---

### Group of orthologs #512. Best score 1048 bits Score difference with first non-orthologous sequence - OB3b\_Refseq\_for\_inparanoid.fasta:1048 OB3b\_Genbank\_for\_inparanoid.fasta:990

```
CQW49_RS11065       	100.00%		CQW49_11035         	100.00%
Bootstrap support for CQW49_RS11065 as seed ortholog is 100%.
Bootstrap support for CQW49_11035 as seed ortholog is 100%.
```

---

### Group of orthologs #513. Best score 1047 bits Score difference with first non-orthologous sequence - OB3b\_Refseq\_for\_inparanoid.fasta:1047 OB3b\_Genbank\_for\_inparanoid.fasta:1047

```
CQW49_RS20470       	100.00%		CQW49_20400         	100.00%
Bootstrap support for CQW49_RS20470 as seed ortholog is 100%.
Bootstrap support for CQW49_20400 as seed ortholog is 100%.
```

---

### Group of orthologs #514. Best score 1045 bits Score difference with first non-orthologous sequence - OB3b\_Refseq\_for\_inparanoid.fasta:1045 OB3b\_Genbank\_for\_inparanoid.fasta:362

```
CQW49_RS05770       	100.00%		CQW49_05760         	100.00%
Bootstrap support for CQW49_RS05770 as seed ortholog is 100%.
Bootstrap support for CQW49_05760 as seed ortholog is 100%.
```

---

### Group of orthologs #515. Best score 1045 bits Score difference with first non-orthologous sequence - OB3b\_Refseq\_for\_inparanoid.fasta:1045 OB3b\_Genbank\_for\_inparanoid.fasta:886

```
CQW49_RS07025       	100.00%		CQW49_07015         	100.00%
Bootstrap support for CQW49_RS07025 as seed ortholog is 100%.
Bootstrap support for CQW49_07015 as seed ortholog is 100%.
```

---

### Group of orthologs #516. Best score 1045 bits Score difference with first non-orthologous sequence - OB3b\_Refseq\_for\_inparanoid.fasta:1045 OB3b\_Genbank\_for\_inparanoid.fasta:1045

```
CQW49_RS15640       	100.00%		CQW49_15605         	100.00%
Bootstrap support for CQW49_RS15640 as seed ortholog is 100%.
Bootstrap support for CQW49_15605 as seed ortholog is 100%.
```

---

### Group of orthologs #517. Best score 1045 bits Score difference with first non-orthologous sequence - OB3b\_Refseq\_for\_inparanoid.fasta:1045 OB3b\_Genbank\_for\_inparanoid.fasta:1045

```
CQW49_RS16475       	100.00%		CQW49_16440         	100.00%
Bootstrap support for CQW49_RS16475 as seed ortholog is 100%.
Bootstrap support for CQW49_16440 as seed ortholog is 100%.
```

---

### Group of orthologs #518. Best score 1045 bits Score difference with first non-orthologous sequence - OB3b\_Refseq\_for\_inparanoid.fasta:1045 OB3b\_Genbank\_for\_inparanoid.fasta:1045

```
CQW49_RS16670       	100.00%		CQW49_16630         	100.00%
Bootstrap support for CQW49_RS16670 as seed ortholog is 100%.
Bootstrap support for CQW49_16630 as seed ortholog is 100%.
```

---

### Group of orthologs #519. Best score 1044 bits Score difference with first non-orthologous sequence - OB3b\_Refseq\_for\_inparanoid.fasta:1044 OB3b\_Genbank\_for\_inparanoid.fasta:1044

```
CQW49_RS01595       	100.00%		CQW49_01590         	100.00%
Bootstrap support for CQW49_RS01595 as seed ortholog is 100%.
Bootstrap support for CQW49_01590 as seed ortholog is 100%.
```

---

### Group of orthologs #520. Best score 1044 bits Score difference with first non-orthologous sequence - OB3b\_Refseq\_for\_inparanoid.fasta:1044 OB3b\_Genbank\_for\_inparanoid.fasta:1044

```
CQW49_RS15175       	100.00%		CQW49_15140         	100.00%
Bootstrap support for CQW49_RS15175 as seed ortholog is 100%.
Bootstrap support for CQW49_15140 as seed ortholog is 100%.
```

---

### Group of orthologs #521. Best score 1042 bits Score difference with first non-orthologous sequence - OB3b\_Refseq\_for\_inparanoid.fasta:1042 OB3b\_Genbank\_for\_inparanoid.fasta:761

```
CQW49_RS09815       	100.00%		CQW49_09795         	100.00%
Bootstrap support for CQW49_RS09815 as seed ortholog is 100%.
Bootstrap support for CQW49_09795 as seed ortholog is 100%.
```

---

### Group of orthologs #522. Best score 1042 bits Score difference with first non-orthologous sequence - OB3b\_Refseq\_for\_inparanoid.fasta:1042 OB3b\_Genbank\_for\_inparanoid.fasta:75

```
CQW49_RS15365       	100.00%		CQW49_15330         	100.00%
Bootstrap support for CQW49_RS15365 as seed ortholog is 100%.
Bootstrap support for CQW49_15330 as seed ortholog is 99%.
```

---

### Group of orthologs #523. Best score 1041 bits Score difference with first non-orthologous sequence - OB3b\_Refseq\_for\_inparanoid.fasta:1041 OB3b\_Genbank\_for\_inparanoid.fasta:1041

```
CQW49_RS02185       	100.00%		CQW49_02180         	100.00%
Bootstrap support for CQW49_RS02185 as seed ortholog is 100%.
Bootstrap support for CQW49_02180 as seed ortholog is 100%.
```

---

### Group of orthologs #524. Best score 1040 bits Score difference with first non-orthologous sequence - OB3b\_Refseq\_for\_inparanoid.fasta:1040 OB3b\_Genbank\_for\_inparanoid.fasta:1040

```
CQW49_RS05955       	100.00%		CQW49_05945         	100.00%
Bootstrap support for CQW49_RS05955 as seed ortholog is 100%.
Bootstrap support for CQW49_05945 as seed ortholog is 100%.
```

---

### Group of orthologs #525. Best score 1040 bits Score difference with first non-orthologous sequence - OB3b\_Refseq\_for\_inparanoid.fasta:1040 OB3b\_Genbank\_for\_inparanoid.fasta:1040

```
CQW49_RS11665       	100.00%		CQW49_11630         	100.00%
Bootstrap support for CQW49_RS11665 as seed ortholog is 100%.
Bootstrap support for CQW49_11630 as seed ortholog is 100%.
```

---

### Group of orthologs #526. Best score 1040 bits Score difference with first non-orthologous sequence - OB3b\_Refseq\_for\_inparanoid.fasta:1040 OB3b\_Genbank\_for\_inparanoid.fasta:630

```
CQW49_RS12960       	100.00%		CQW49_12925         	100.00%
Bootstrap support for CQW49_RS12960 as seed ortholog is 100%.
Bootstrap support for CQW49_12925 as seed ortholog is 100%.
```

---

### Group of orthologs #527. Best score 1040 bits Score difference with first non-orthologous sequence - OB3b\_Refseq\_for\_inparanoid.fasta:1040 OB3b\_Genbank\_for\_inparanoid.fasta:1040

```
CQW49_RS18560       	100.00%		CQW49_18500         	100.00%
Bootstrap support for CQW49_RS18560 as seed ortholog is 100%.
Bootstrap support for CQW49_18500 as seed ortholog is 100%.
```

---

### Group of orthologs #528. Best score 1037 bits Score difference with first non-orthologous sequence - OB3b\_Refseq\_for\_inparanoid.fasta:1037 OB3b\_Genbank\_for\_inparanoid.fasta:5

```
CQW49_RS14770       	100.00%		CQW49_14735         	100.00%
Bootstrap support for CQW49_RS14770 as seed ortholog is 100%.
Bootstrap support for CQW49_14735 as seed ortholog is 61%.
Alternative seed ortholog is CQW49_23940 (5 bits away from this cluster)
```

---

### Group of orthologs #529. Best score 1036 bits Score difference with first non-orthologous sequence - OB3b\_Refseq\_for\_inparanoid.fasta:1036 OB3b\_Genbank\_for\_inparanoid.fasta:1036

```
CQW49_RS12125       	100.00%		CQW49_12090         	100.00%
Bootstrap support for CQW49_RS12125 as seed ortholog is 100%.
Bootstrap support for CQW49_12090 as seed ortholog is 100%.
```

---

### Group of orthologs #530. Best score 1036 bits Score difference with first non-orthologous sequence - OB3b\_Refseq\_for\_inparanoid.fasta:1036 OB3b\_Genbank\_for\_inparanoid.fasta:1036

```
CQW49_RS12485       	100.00%		CQW49_12450         	100.00%
Bootstrap support for CQW49_RS12485 as seed ortholog is 100%.
Bootstrap support for CQW49_12450 as seed ortholog is 100%.
```

---

### Group of orthologs #531. Best score 1032 bits Score difference with first non-orthologous sequence - OB3b\_Refseq\_for\_inparanoid.fasta:1032 OB3b\_Genbank\_for\_inparanoid.fasta:1032

```
CQW49_RS04980       	100.00%		CQW49_04975         	100.00%
Bootstrap support for CQW49_RS04980 as seed ortholog is 100%.
Bootstrap support for CQW49_04975 as seed ortholog is 100%.
```

---

### Group of orthologs #532. Best score 1032 bits Score difference with first non-orthologous sequence - OB3b\_Refseq\_for\_inparanoid.fasta:1032 OB3b\_Genbank\_for\_inparanoid.fasta:1032

```
CQW49_RS16685       	100.00%		CQW49_16645         	100.00%
Bootstrap support for CQW49_RS16685 as seed ortholog is 100%.
Bootstrap support for CQW49_16645 as seed ortholog is 100%.
```

---

### Group of orthologs #533. Best score 1032 bits Score difference with first non-orthologous sequence - OB3b\_Refseq\_for\_inparanoid.fasta:1032 OB3b\_Genbank\_for\_inparanoid.fasta:1032

```
groL                	100.00%		CQW49_19005         	100.00%
Bootstrap support for groL as seed ortholog is 100%.
Bootstrap support for CQW49_19005 as seed ortholog is 100%.
```

---

### Group of orthologs #534. Best score 1032 bits Score difference with first non-orthologous sequence - OB3b\_Refseq\_for\_inparanoid.fasta:1032 OB3b\_Genbank\_for\_inparanoid.fasta:1032

```
CQW49_RS21305       	100.00%		CQW49_21230         	100.00%
Bootstrap support for CQW49_RS21305 as seed ortholog is 100%.
Bootstrap support for CQW49_21230 as seed ortholog is 100%.
```

---

### Group of orthologs #535. Best score 1031 bits Score difference with first non-orthologous sequence - OB3b\_Refseq\_for\_inparanoid.fasta:1031 OB3b\_Genbank\_for\_inparanoid.fasta:1031

```
CQW49_RS17005       	100.00%		CQW49_16960         	100.00%
Bootstrap support for CQW49_RS17005 as seed ortholog is 100%.
Bootstrap support for CQW49_16960 as seed ortholog is 100%.
```

---

### Group of orthologs #536. Best score 1030 bits Score difference with first non-orthologous sequence - OB3b\_Refseq\_for\_inparanoid.fasta:1030 OB3b\_Genbank\_for\_inparanoid.fasta:1030

```
CQW49_RS00610       	100.00%		CQW49_00610         	100.00%
Bootstrap support for CQW49_RS00610 as seed ortholog is 100%.
Bootstrap support for CQW49_00610 as seed ortholog is 100%.
```

---

### Group of orthologs #537. Best score 1030 bits Score difference with first non-orthologous sequence - OB3b\_Refseq\_for\_inparanoid.fasta:1030 OB3b\_Genbank\_for\_inparanoid.fasta:887

```
CQW49_RS13850       	100.00%		CQW49_13815         	100.00%
Bootstrap support for CQW49_RS13850 as seed ortholog is 100%.
Bootstrap support for CQW49_13815 as seed ortholog is 100%.
```

---

### Group of orthologs #538. Best score 1030 bits Score difference with first non-orthologous sequence - OB3b\_Refseq\_for\_inparanoid.fasta:1030 OB3b\_Genbank\_for\_inparanoid.fasta:1030

```
CQW49_RS19410       	100.00%		CQW49_19355         	100.00%
Bootstrap support for CQW49_RS19410 as seed ortholog is 100%.
Bootstrap support for CQW49_19355 as seed ortholog is 100%.
```

---

### Group of orthologs #539. Best score 1029 bits Score difference with first non-orthologous sequence - OB3b\_Refseq\_for\_inparanoid.fasta:1029 OB3b\_Genbank\_for\_inparanoid.fasta:1029

```
CQW49_RS19215       	100.00%		CQW49_19160         	100.00%
Bootstrap support for CQW49_RS19215 as seed ortholog is 100%.
Bootstrap support for CQW49_19160 as seed ortholog is 100%.
```

---

### Group of orthologs #540. Best score 1028 bits Score difference with first non-orthologous sequence - OB3b\_Refseq\_for\_inparanoid.fasta:1028 OB3b\_Genbank\_for\_inparanoid.fasta:1028

```
ligD                	100.00%		CQW49_03455         	100.00%
Bootstrap support for ligD as seed ortholog is 100%.
Bootstrap support for CQW49_03455 as seed ortholog is 100%.
```

---

### Group of orthologs #541. Best score 1028 bits Score difference with first non-orthologous sequence - OB3b\_Refseq\_for\_inparanoid.fasta:1028 OB3b\_Genbank\_for\_inparanoid.fasta:1028

```
CQW49_RS20070       	100.00%		CQW49_20005         	100.00%
Bootstrap support for CQW49_RS20070 as seed ortholog is 100%.
Bootstrap support for CQW49_20005 as seed ortholog is 100%.
```

---

### Group of orthologs #542. Best score 1027 bits Score difference with first non-orthologous sequence - OB3b\_Refseq\_for\_inparanoid.fasta:1027 OB3b\_Genbank\_for\_inparanoid.fasta:1027

```
purH                	100.00%		CQW49_01745         	100.00%
Bootstrap support for purH as seed ortholog is 100%.
Bootstrap support for CQW49_01745 as seed ortholog is 100%.
```

---

### Group of orthologs #543. Best score 1027 bits Score difference with first non-orthologous sequence - OB3b\_Refseq\_for\_inparanoid.fasta:1027 OB3b\_Genbank\_for\_inparanoid.fasta:1027

```
CQW49_RS20010       	100.00%		CQW49_19945         	100.00%
Bootstrap support for CQW49_RS20010 as seed ortholog is 100%.
Bootstrap support for CQW49_19945 as seed ortholog is 100%.
```

---

### Group of orthologs #544. Best score 1027 bits Score difference with first non-orthologous sequence - OB3b\_Refseq\_for\_inparanoid.fasta:1027 OB3b\_Genbank\_for\_inparanoid.fasta:1027

```
CQW49_RS20280       	100.00%		CQW49_20210         	100.00%
Bootstrap support for CQW49_RS20280 as seed ortholog is 100%.
Bootstrap support for CQW49_20210 as seed ortholog is 100%.
```

---

### Group of orthologs #545. Best score 1026 bits Score difference with first non-orthologous sequence - OB3b\_Refseq\_for\_inparanoid.fasta:1026 OB3b\_Genbank\_for\_inparanoid.fasta:1026

```
CQW49_RS07125       	100.00%		CQW49_07115         	100.00%
Bootstrap support for CQW49_RS07125 as seed ortholog is 100%.
Bootstrap support for CQW49_07115 as seed ortholog is 100%.
```

---

### Group of orthologs #546. Best score 1026 bits Score difference with first non-orthologous sequence - OB3b\_Refseq\_for\_inparanoid.fasta:1026 OB3b\_Genbank\_for\_inparanoid.fasta:1026

```
CQW49_RS09140       	100.00%		CQW49_09120         	100.00%
Bootstrap support for CQW49_RS09140 as seed ortholog is 100%.
Bootstrap support for CQW49_09120 as seed ortholog is 100%.
```

---

### Group of orthologs #547. Best score 1024 bits Score difference with first non-orthologous sequence - OB3b\_Refseq\_for\_inparanoid.fasta:1024 OB3b\_Genbank\_for\_inparanoid.fasta:1024

```
CQW49_RS09455       	100.00%		CQW49_09435         	100.00%
Bootstrap support for CQW49_RS09455 as seed ortholog is 100%.
Bootstrap support for CQW49_09435 as seed ortholog is 100%.
```

---

### Group of orthologs #548. Best score 1023 bits Score difference with first non-orthologous sequence - OB3b\_Refseq\_for\_inparanoid.fasta:1023 OB3b\_Genbank\_for\_inparanoid.fasta:1023

```
CQW49_RS12140       	100.00%		CQW49_12105         	100.00%
Bootstrap support for CQW49_RS12140 as seed ortholog is 100%.
Bootstrap support for CQW49_12105 as seed ortholog is 100%.
```

---

### Group of orthologs #549. Best score 1022 bits Score difference with first non-orthologous sequence - OB3b\_Refseq\_for\_inparanoid.fasta:1022 OB3b\_Genbank\_for\_inparanoid.fasta:1022

```
CQW49_RS16120       	100.00%		CQW49_16085         	100.00%
Bootstrap support for CQW49_RS16120 as seed ortholog is 100%.
Bootstrap support for CQW49_16085 as seed ortholog is 100%.
```

---

### Group of orthologs #550. Best score 1020 bits Score difference with first non-orthologous sequence - OB3b\_Refseq\_for\_inparanoid.fasta:1020 OB3b\_Genbank\_for\_inparanoid.fasta:1020

```
CQW49_RS13165       	100.00%		CQW49_13130         	100.00%
Bootstrap support for CQW49_RS13165 as seed ortholog is 100%.
Bootstrap support for CQW49_13130 as seed ortholog is 100%.
```

---

### Group of orthologs #551. Best score 1019 bits Score difference with first non-orthologous sequence - OB3b\_Refseq\_for\_inparanoid.fasta:1019 OB3b\_Genbank\_for\_inparanoid.fasta:1019

```
CQW49_RS05540       	100.00%		CQW49_05530         	100.00%
Bootstrap support for CQW49_RS05540 as seed ortholog is 100%.
Bootstrap support for CQW49_05530 as seed ortholog is 100%.
```

---

### Group of orthologs #552. Best score 1019 bits Score difference with first non-orthologous sequence - OB3b\_Refseq\_for\_inparanoid.fasta:1019 OB3b\_Genbank\_for\_inparanoid.fasta:1019

```
CQW49_RS18060       	100.00%		CQW49_18000         	100.00%
Bootstrap support for CQW49_RS18060 as seed ortholog is 100%.
Bootstrap support for CQW49_18000 as seed ortholog is 100%.
```

---

### Group of orthologs #553. Best score 1017 bits Score difference with first non-orthologous sequence - OB3b\_Refseq\_for\_inparanoid.fasta:1017 OB3b\_Genbank\_for\_inparanoid.fasta:1017

```
rpoN                	100.00%		CQW49_13280         	100.00%
Bootstrap support for rpoN as seed ortholog is 100%.
Bootstrap support for CQW49_13280 as seed ortholog is 100%.
```

---

### Group of orthologs #554. Best score 1017 bits Score difference with first non-orthologous sequence - OB3b\_Refseq\_for\_inparanoid.fasta:1017 OB3b\_Genbank\_for\_inparanoid.fasta:1017

```
CQW49_RS14890       	100.00%		CQW49_14855         	100.00%
Bootstrap support for CQW49_RS14890 as seed ortholog is 100%.
Bootstrap support for CQW49_14855 as seed ortholog is 100%.
```

---

### Group of orthologs #555. Best score 1017 bits Score difference with first non-orthologous sequence - OB3b\_Refseq\_for\_inparanoid.fasta:1017 OB3b\_Genbank\_for\_inparanoid.fasta:1017

```
CQW49_RS20020       	100.00%		CQW49_19955         	100.00%
Bootstrap support for CQW49_RS20020 as seed ortholog is 100%.
Bootstrap support for CQW49_19955 as seed ortholog is 100%.
```

---

### Group of orthologs #556. Best score 1015 bits Score difference with first non-orthologous sequence - OB3b\_Refseq\_for\_inparanoid.fasta:1015 OB3b\_Genbank\_for\_inparanoid.fasta:1015

```
CQW49_RS11455       	100.00%		CQW49_11425         	100.00%
Bootstrap support for CQW49_RS11455 as seed ortholog is 100%.
Bootstrap support for CQW49_11425 as seed ortholog is 100%.
```

---

### Group of orthologs #557. Best score 1015 bits Score difference with first non-orthologous sequence - OB3b\_Refseq\_for\_inparanoid.fasta:1015 OB3b\_Genbank\_for\_inparanoid.fasta:716

```
CQW49_RS15950       	100.00%		CQW49_15915         	100.00%
Bootstrap support for CQW49_RS15950 as seed ortholog is 100%.
Bootstrap support for CQW49_15915 as seed ortholog is 100%.
```

---

### Group of orthologs #558. Best score 1015 bits Score difference with first non-orthologous sequence - OB3b\_Refseq\_for\_inparanoid.fasta:1015 OB3b\_Genbank\_for\_inparanoid.fasta:1015

```
CQW49_RS20715       	100.00%		CQW49_20640         	100.00%
Bootstrap support for CQW49_RS20715 as seed ortholog is 100%.
Bootstrap support for CQW49_20640 as seed ortholog is 100%.
```

---

### Group of orthologs #559. Best score 1014 bits Score difference with first non-orthologous sequence - OB3b\_Refseq\_for\_inparanoid.fasta:1014 OB3b\_Genbank\_for\_inparanoid.fasta:1014

```
CQW49_RS04165       	100.00%		CQW49_04155         	100.00%
Bootstrap support for CQW49_RS04165 as seed ortholog is 100%.
Bootstrap support for CQW49_04155 as seed ortholog is 100%.
```

---

### Group of orthologs #560. Best score 1014 bits Score difference with first non-orthologous sequence - OB3b\_Refseq\_for\_inparanoid.fasta:1014 OB3b\_Genbank\_for\_inparanoid.fasta:1014

```
CQW49_RS10875       	100.00%		CQW49_10845         	100.00%
Bootstrap support for CQW49_RS10875 as seed ortholog is 100%.
Bootstrap support for CQW49_10845 as seed ortholog is 100%.
```

---

### Group of orthologs #561. Best score 1014 bits Score difference with first non-orthologous sequence - OB3b\_Refseq\_for\_inparanoid.fasta:1014 OB3b\_Genbank\_for\_inparanoid.fasta:1014

```
CQW49_RS10910       	100.00%		CQW49_10880         	100.00%
Bootstrap support for CQW49_RS10910 as seed ortholog is 100%.
Bootstrap support for CQW49_10880 as seed ortholog is 100%.
```

---

### Group of orthologs #562. Best score 1014 bits Score difference with first non-orthologous sequence - OB3b\_Refseq\_for\_inparanoid.fasta:1014 OB3b\_Genbank\_for\_inparanoid.fasta:1014

```
CQW49_RS17920       	100.00%		CQW49_17865         	100.00%
Bootstrap support for CQW49_RS17920 as seed ortholog is 100%.
Bootstrap support for CQW49_17865 as seed ortholog is 100%.
```

---

### Group of orthologs #563. Best score 1012 bits Score difference with first non-orthologous sequence - OB3b\_Refseq\_for\_inparanoid.fasta:1012 OB3b\_Genbank\_for\_inparanoid.fasta:1012

```
secD                	100.00%		CQW49_03010         	100.00%
Bootstrap support for secD as seed ortholog is 100%.
Bootstrap support for CQW49_03010 as seed ortholog is 100%.
```

---

### Group of orthologs #564. Best score 1012 bits Score difference with first non-orthologous sequence - OB3b\_Refseq\_for\_inparanoid.fasta:1012 OB3b\_Genbank\_for\_inparanoid.fasta:889

```
CQW49_RS10135       	100.00%		CQW49_10110         	100.00%
Bootstrap support for CQW49_RS10135 as seed ortholog is 100%.
Bootstrap support for CQW49_10110 as seed ortholog is 100%.
```

---

### Group of orthologs #565. Best score 1011 bits Score difference with first non-orthologous sequence - OB3b\_Refseq\_for\_inparanoid.fasta:1011 OB3b\_Genbank\_for\_inparanoid.fasta:1011

```
CQW49_RS00805       	100.00%		CQW49_00805         	100.00%
Bootstrap support for CQW49_RS00805 as seed ortholog is 100%.
Bootstrap support for CQW49_00805 as seed ortholog is 100%.
```

---

### Group of orthologs #566. Best score 1011 bits Score difference with first non-orthologous sequence - OB3b\_Refseq\_for\_inparanoid.fasta:1011 OB3b\_Genbank\_for\_inparanoid.fasta:494

```
CQW49_RS08220       	100.00%		CQW49_08210         	100.00%
Bootstrap support for CQW49_RS08220 as seed ortholog is 100%.
Bootstrap support for CQW49_08210 as seed ortholog is 100%.
```

---

### Group of orthologs #567. Best score 1010 bits Score difference with first non-orthologous sequence - OB3b\_Refseq\_for\_inparanoid.fasta:1010 OB3b\_Genbank\_for\_inparanoid.fasta:1010

```
CQW49_RS08280       	100.00%		CQW49_08270         	100.00%
Bootstrap support for CQW49_RS08280 as seed ortholog is 100%.
Bootstrap support for CQW49_08270 as seed ortholog is 100%.
```

---

### Group of orthologs #568. Best score 1010 bits Score difference with first non-orthologous sequence - OB3b\_Refseq\_for\_inparanoid.fasta:1010 OB3b\_Genbank\_for\_inparanoid.fasta:844

```
nifD                	100.00%		CQW49_08725         	100.00%
Bootstrap support for nifD as seed ortholog is 100%.
Bootstrap support for CQW49_08725 as seed ortholog is 100%.
```

---

### Group of orthologs #569. Best score 1010 bits Score difference with first non-orthologous sequence - OB3b\_Refseq\_for\_inparanoid.fasta:1010 OB3b\_Genbank\_for\_inparanoid.fasta:1010

```
CQW49_RS10695       	100.00%		CQW49_10665         	100.00%
Bootstrap support for CQW49_RS10695 as seed ortholog is 100%.
Bootstrap support for CQW49_10665 as seed ortholog is 100%.
```

---

### Group of orthologs #570. Best score 1010 bits Score difference with first non-orthologous sequence - OB3b\_Refseq\_for\_inparanoid.fasta:1010 OB3b\_Genbank\_for\_inparanoid.fasta:1010

```
CQW49_RS19905       	100.00%		CQW49_19840         	100.00%
Bootstrap support for CQW49_RS19905 as seed ortholog is 100%.
Bootstrap support for CQW49_19840 as seed ortholog is 100%.
```

---

### Group of orthologs #571. Best score 1009 bits Score difference with first non-orthologous sequence - OB3b\_Refseq\_for\_inparanoid.fasta:1009 OB3b\_Genbank\_for\_inparanoid.fasta:1009

```
CQW49_RS06285       	100.00%		CQW49_06275         	100.00%
Bootstrap support for CQW49_RS06285 as seed ortholog is 100%.
Bootstrap support for CQW49_06275 as seed ortholog is 100%.
```

---

### Group of orthologs #572. Best score 1007 bits Score difference with first non-orthologous sequence - OB3b\_Refseq\_for\_inparanoid.fasta:1007 OB3b\_Genbank\_for\_inparanoid.fasta:1007

```
CQW49_RS05065       	100.00%		CQW49_05060         	100.00%
Bootstrap support for CQW49_RS05065 as seed ortholog is 100%.
Bootstrap support for CQW49_05060 as seed ortholog is 100%.
```

---

### Group of orthologs #573. Best score 1007 bits Score difference with first non-orthologous sequence - OB3b\_Refseq\_for\_inparanoid.fasta:1007 OB3b\_Genbank\_for\_inparanoid.fasta:1007

```
CQW49_RS11590       	100.00%		CQW49_11555         	100.00%
Bootstrap support for CQW49_RS11590 as seed ortholog is 100%.
Bootstrap support for CQW49_11555 as seed ortholog is 100%.
```

---

### Group of orthologs #574. Best score 1007 bits Score difference with first non-orthologous sequence - OB3b\_Refseq\_for\_inparanoid.fasta:1007 OB3b\_Genbank\_for\_inparanoid.fasta:1007

```
ubiB                	100.00%		CQW49_18425         	100.00%
Bootstrap support for ubiB as seed ortholog is 100%.
Bootstrap support for CQW49_18425 as seed ortholog is 100%.
```

---

### Group of orthologs #575. Best score 1006 bits Score difference with first non-orthologous sequence - OB3b\_Refseq\_for\_inparanoid.fasta:1006 OB3b\_Genbank\_for\_inparanoid.fasta:1006

```
CQW49_RS13385       	100.00%		CQW49_13350         	100.00%
Bootstrap support for CQW49_RS13385 as seed ortholog is 100%.
Bootstrap support for CQW49_13350 as seed ortholog is 100%.
```

---

### Group of orthologs #576. Best score 1005 bits Score difference with first non-orthologous sequence - OB3b\_Refseq\_for\_inparanoid.fasta:1005 OB3b\_Genbank\_for\_inparanoid.fasta:1005

```
CQW49_RS17960       	100.00%		CQW49_17905         	100.00%
Bootstrap support for CQW49_RS17960 as seed ortholog is 100%.
Bootstrap support for CQW49_17905 as seed ortholog is 100%.
```

---

### Group of orthologs #577. Best score 1004 bits Score difference with first non-orthologous sequence - OB3b\_Refseq\_for\_inparanoid.fasta:1004 OB3b\_Genbank\_for\_inparanoid.fasta:1004

```
CQW49_RS04700       	100.00%		CQW49_04690         	100.00%
Bootstrap support for CQW49_RS04700 as seed ortholog is 100%.
Bootstrap support for CQW49_04690 as seed ortholog is 100%.
```

---

### Group of orthologs #578. Best score 1003 bits Score difference with first non-orthologous sequence - OB3b\_Refseq\_for\_inparanoid.fasta:1003 OB3b\_Genbank\_for\_inparanoid.fasta:1003

```
CQW49_RS14835       	100.00%		CQW49_14800         	100.00%
Bootstrap support for CQW49_RS14835 as seed ortholog is 100%.
Bootstrap support for CQW49_14800 as seed ortholog is 100%.
```

---

### Group of orthologs #579. Best score 1003 bits Score difference with first non-orthologous sequence - OB3b\_Refseq\_for\_inparanoid.fasta:1003 OB3b\_Genbank\_for\_inparanoid.fasta:1003

```
CQW49_RS16630       	100.00%		CQW49_16590         	100.00%
Bootstrap support for CQW49_RS16630 as seed ortholog is 100%.
Bootstrap support for CQW49_16590 as seed ortholog is 100%.
```

---

### Group of orthologs #580. Best score 1001 bits Score difference with first non-orthologous sequence - OB3b\_Refseq\_for\_inparanoid.fasta:1001 OB3b\_Genbank\_for\_inparanoid.fasta:1001

```
CQW49_RS14345       	100.00%		CQW49_14310         	100.00%
Bootstrap support for CQW49_RS14345 as seed ortholog is 100%.
Bootstrap support for CQW49_14310 as seed ortholog is 100%.
```

---

### Group of orthologs #581. Best score 1001 bits Score difference with first non-orthologous sequence - OB3b\_Refseq\_for\_inparanoid.fasta:1001 OB3b\_Genbank\_for\_inparanoid.fasta:1001

```
CQW49_RS15830       	100.00%		CQW49_15795         	100.00%
Bootstrap support for CQW49_RS15830 as seed ortholog is 100%.
Bootstrap support for CQW49_15795 as seed ortholog is 100%.
```

---

### Group of orthologs #582. Best score 1000 bits Score difference with first non-orthologous sequence - OB3b\_Refseq\_for\_inparanoid.fasta:1000 OB3b\_Genbank\_for\_inparanoid.fasta:827

```
CQW49_RS01780       	100.00%		CQW49_01775         	100.00%
Bootstrap support for CQW49_RS01780 as seed ortholog is 100%.
Bootstrap support for CQW49_01775 as seed ortholog is 100%.
```

---

### Group of orthologs #583. Best score 999 bits Score difference with first non-orthologous sequence - OB3b\_Refseq\_for\_inparanoid.fasta:999 OB3b\_Genbank\_for\_inparanoid.fasta:999

```
CQW49_RS19495       	100.00%		CQW49_19440         	100.00%
Bootstrap support for CQW49_RS19495 as seed ortholog is 100%.
Bootstrap support for CQW49_19440 as seed ortholog is 100%.
```

---

### Group of orthologs #584. Best score 999 bits Score difference with first non-orthologous sequence - OB3b\_Refseq\_for\_inparanoid.fasta:999 OB3b\_Genbank\_for\_inparanoid.fasta:999

```
trpE                	100.00%		CQW49_20270         	100.00%
Bootstrap support for trpE as seed ortholog is 100%.
Bootstrap support for CQW49_20270 as seed ortholog is 100%.
```

---

### Group of orthologs #585. Best score 998 bits Score difference with first non-orthologous sequence - OB3b\_Refseq\_for\_inparanoid.fasta:998 OB3b\_Genbank\_for\_inparanoid.fasta:953

```
CQW49_RS11865       	100.00%		CQW49_11830         	100.00%
Bootstrap support for CQW49_RS11865 as seed ortholog is 100%.
Bootstrap support for CQW49_11830 as seed ortholog is 100%.
```

---

### Group of orthologs #586. Best score 998 bits Score difference with first non-orthologous sequence - OB3b\_Refseq\_for\_inparanoid.fasta:998 OB3b\_Genbank\_for\_inparanoid.fasta:998

```
CQW49_RS16565       	100.00%		CQW49_16525         	100.00%
Bootstrap support for CQW49_RS16565 as seed ortholog is 100%.
Bootstrap support for CQW49_16525 as seed ortholog is 100%.
```

---

### Group of orthologs #587. Best score 996 bits Score difference with first non-orthologous sequence - OB3b\_Refseq\_for\_inparanoid.fasta:996 OB3b\_Genbank\_for\_inparanoid.fasta:996

```
CQW49_RS04450       	100.00%		CQW49_04445         	100.00%
Bootstrap support for CQW49_RS04450 as seed ortholog is 100%.
Bootstrap support for CQW49_04445 as seed ortholog is 100%.
```

---

### Group of orthologs #588. Best score 995 bits Score difference with first non-orthologous sequence - OB3b\_Refseq\_for\_inparanoid.fasta:995 OB3b\_Genbank\_for\_inparanoid.fasta:995

```
CQW49_RS00815       	100.00%		CQW49_00815         	100.00%
Bootstrap support for CQW49_RS00815 as seed ortholog is 100%.
Bootstrap support for CQW49_00815 as seed ortholog is 100%.
```

---

### Group of orthologs #589. Best score 995 bits Score difference with first non-orthologous sequence - OB3b\_Refseq\_for\_inparanoid.fasta:995 OB3b\_Genbank\_for\_inparanoid.fasta:995

```
CQW49_RS20795       	100.00%		CQW49_20720         	100.00%
Bootstrap support for CQW49_RS20795 as seed ortholog is 100%.
Bootstrap support for CQW49_20720 as seed ortholog is 100%.
```

---

### Group of orthologs #590. Best score 993 bits Score difference with first non-orthologous sequence - OB3b\_Refseq\_for\_inparanoid.fasta:993 OB3b\_Genbank\_for\_inparanoid.fasta:993

```
CQW49_RS03040       	100.00%		CQW49_03030         	100.00%
Bootstrap support for CQW49_RS03040 as seed ortholog is 100%.
Bootstrap support for CQW49_03030 as seed ortholog is 100%.
```

---

### Group of orthologs #591. Best score 993 bits Score difference with first non-orthologous sequence - OB3b\_Refseq\_for\_inparanoid.fasta:993 OB3b\_Genbank\_for\_inparanoid.fasta:993

```
CQW49_RS07770       	100.00%		CQW49_07760         	100.00%
Bootstrap support for CQW49_RS07770 as seed ortholog is 100%.
Bootstrap support for CQW49_07760 as seed ortholog is 100%.
```

---

### Group of orthologs #592. Best score 993 bits Score difference with first non-orthologous sequence - OB3b\_Refseq\_for\_inparanoid.fasta:993 OB3b\_Genbank\_for\_inparanoid.fasta:993

```
CQW49_RS08845       	100.00%		CQW49_08835         	100.00%
Bootstrap support for CQW49_RS08845 as seed ortholog is 100%.
Bootstrap support for CQW49_08835 as seed ortholog is 100%.
```

---

### Group of orthologs #593. Best score 990 bits Score difference with first non-orthologous sequence - OB3b\_Refseq\_for\_inparanoid.fasta:990 OB3b\_Genbank\_for\_inparanoid.fasta:990

```
CQW49_RS04365       	100.00%		CQW49_04360         	100.00%
Bootstrap support for CQW49_RS04365 as seed ortholog is 100%.
Bootstrap support for CQW49_04360 as seed ortholog is 100%.
```

---

### Group of orthologs #594. Best score 989 bits Score difference with first non-orthologous sequence - OB3b\_Refseq\_for\_inparanoid.fasta:989 OB3b\_Genbank\_for\_inparanoid.fasta:989

```
ppx                 	100.00%		CQW49_02845         	100.00%
Bootstrap support for ppx as seed ortholog is 100%.
Bootstrap support for CQW49_02845 as seed ortholog is 100%.
```

---

### Group of orthologs #595. Best score 989 bits Score difference with first non-orthologous sequence - OB3b\_Refseq\_for\_inparanoid.fasta:989 OB3b\_Genbank\_for\_inparanoid.fasta:989

```
CQW49_RS13585       	100.00%		CQW49_13550         	100.00%
Bootstrap support for CQW49_RS13585 as seed ortholog is 100%.
Bootstrap support for CQW49_13550 as seed ortholog is 100%.
```

---

### Group of orthologs #596. Best score 988 bits Score difference with first non-orthologous sequence - OB3b\_Refseq\_for\_inparanoid.fasta:988 OB3b\_Genbank\_for\_inparanoid.fasta:988

```
CQW49_RS00995       	100.00%		CQW49_00995         	100.00%
Bootstrap support for CQW49_RS00995 as seed ortholog is 100%.
Bootstrap support for CQW49_00995 as seed ortholog is 100%.
```

---

### Group of orthologs #597. Best score 988 bits Score difference with first non-orthologous sequence - OB3b\_Refseq\_for\_inparanoid.fasta:988 OB3b\_Genbank\_for\_inparanoid.fasta:988

```
CQW49_RS04890       	100.00%		CQW49_04880         	100.00%
Bootstrap support for CQW49_RS04890 as seed ortholog is 100%.
Bootstrap support for CQW49_04880 as seed ortholog is 100%.
```

---

### Group of orthologs #598. Best score 988 bits Score difference with first non-orthologous sequence - OB3b\_Refseq\_for\_inparanoid.fasta:988 OB3b\_Genbank\_for\_inparanoid.fasta:988

```
CQW49_RS15135       	100.00%		CQW49_15100         	100.00%
Bootstrap support for CQW49_RS15135 as seed ortholog is 100%.
Bootstrap support for CQW49_15100 as seed ortholog is 100%.
```

---

### Group of orthologs #599. Best score 988 bits Score difference with first non-orthologous sequence - OB3b\_Refseq\_for\_inparanoid.fasta:988 OB3b\_Genbank\_for\_inparanoid.fasta:841

```
CQW49_RS17225       	100.00%		CQW49_17180         	100.00%
Bootstrap support for CQW49_RS17225 as seed ortholog is 100%.
Bootstrap support for CQW49_17180 as seed ortholog is 100%.
```

---

### Group of orthologs #600. Best score 988 bits Score difference with first non-orthologous sequence - OB3b\_Refseq\_for\_inparanoid.fasta:988 OB3b\_Genbank\_for\_inparanoid.fasta:988

```
CQW49_RS20425       	100.00%		CQW49_20355         	100.00%
Bootstrap support for CQW49_RS20425 as seed ortholog is 100%.
Bootstrap support for CQW49_20355 as seed ortholog is 100%.
```

---

### Group of orthologs #601. Best score 987 bits Score difference with first non-orthologous sequence - OB3b\_Refseq\_for\_inparanoid.fasta:987 OB3b\_Genbank\_for\_inparanoid.fasta:987

```
CQW49_RS10335       	100.00%		CQW49_10310         	100.00%
Bootstrap support for CQW49_RS10335 as seed ortholog is 100%.
Bootstrap support for CQW49_10310 as seed ortholog is 100%.
```

---

### Group of orthologs #602. Best score 987 bits Score difference with first non-orthologous sequence - OB3b\_Refseq\_for\_inparanoid.fasta:987 OB3b\_Genbank\_for\_inparanoid.fasta:925

```
CQW49_RS14880       	100.00%		CQW49_14845         	100.00%
Bootstrap support for CQW49_RS14880 as seed ortholog is 100%.
Bootstrap support for CQW49_14845 as seed ortholog is 100%.
```

---

### Group of orthologs #603. Best score 986 bits Score difference with first non-orthologous sequence - OB3b\_Refseq\_for\_inparanoid.fasta:986 OB3b\_Genbank\_for\_inparanoid.fasta:986

```
CQW49_RS16455       	100.00%		CQW49_16420         	100.00%
Bootstrap support for CQW49_RS16455 as seed ortholog is 100%.
Bootstrap support for CQW49_16420 as seed ortholog is 100%.
```

---

### Group of orthologs #604. Best score 985 bits Score difference with first non-orthologous sequence - OB3b\_Refseq\_for\_inparanoid.fasta:985 OB3b\_Genbank\_for\_inparanoid.fasta:985

```
CQW49_RS00295       	100.00%		CQW49_00295         	100.00%
Bootstrap support for CQW49_RS00295 as seed ortholog is 100%.
Bootstrap support for CQW49_00295 as seed ortholog is 100%.
```

---

### Group of orthologs #605. Best score 985 bits Score difference with first non-orthologous sequence - OB3b\_Refseq\_for\_inparanoid.fasta:985 OB3b\_Genbank\_for\_inparanoid.fasta:985

```
gatA                	100.00%		CQW49_01100         	100.00%
Bootstrap support for gatA as seed ortholog is 100%.
Bootstrap support for CQW49_01100 as seed ortholog is 100%.
```

---

### Group of orthologs #606. Best score 984 bits Score difference with first non-orthologous sequence - OB3b\_Refseq\_for\_inparanoid.fasta:984 OB3b\_Genbank\_for\_inparanoid.fasta:984

```
CQW49_RS01415       	100.00%		CQW49_01410         	100.00%
Bootstrap support for CQW49_RS01415 as seed ortholog is 100%.
Bootstrap support for CQW49_01410 as seed ortholog is 100%.
```

---

### Group of orthologs #607. Best score 984 bits Score difference with first non-orthologous sequence - OB3b\_Refseq\_for\_inparanoid.fasta:984 OB3b\_Genbank\_for\_inparanoid.fasta:984

```
CQW49_RS05295       	100.00%		CQW49_05290         	100.00%
Bootstrap support for CQW49_RS05295 as seed ortholog is 100%.
Bootstrap support for CQW49_05290 as seed ortholog is 100%.
```

---

### Group of orthologs #608. Best score 984 bits Score difference with first non-orthologous sequence - OB3b\_Refseq\_for\_inparanoid.fasta:984 OB3b\_Genbank\_for\_inparanoid.fasta:984

```
CQW49_RS06010       	100.00%		CQW49_06000         	100.00%
Bootstrap support for CQW49_RS06010 as seed ortholog is 100%.
Bootstrap support for CQW49_06000 as seed ortholog is 100%.
```

---

### Group of orthologs #609. Best score 984 bits Score difference with first non-orthologous sequence - OB3b\_Refseq\_for\_inparanoid.fasta:984 OB3b\_Genbank\_for\_inparanoid.fasta:984

```
CQW49_RS06880       	100.00%		CQW49_06870         	100.00%
Bootstrap support for CQW49_RS06880 as seed ortholog is 100%.
Bootstrap support for CQW49_06870 as seed ortholog is 100%.
```

---

### Group of orthologs #610. Best score 984 bits Score difference with first non-orthologous sequence - OB3b\_Refseq\_for\_inparanoid.fasta:984 OB3b\_Genbank\_for\_inparanoid.fasta:984

```
CQW49_RS08265       	100.00%		CQW49_08255         	100.00%
Bootstrap support for CQW49_RS08265 as seed ortholog is 100%.
Bootstrap support for CQW49_08255 as seed ortholog is 100%.
```

---

### Group of orthologs #611. Best score 984 bits Score difference with first non-orthologous sequence - OB3b\_Refseq\_for\_inparanoid.fasta:984 OB3b\_Genbank\_for\_inparanoid.fasta:984

```
CQW49_RS13800       	100.00%		CQW49_13765         	100.00%
Bootstrap support for CQW49_RS13800 as seed ortholog is 100%.
Bootstrap support for CQW49_13765 as seed ortholog is 100%.
```

---

### Group of orthologs #612. Best score 983 bits Score difference with first non-orthologous sequence - OB3b\_Refseq\_for\_inparanoid.fasta:983 OB3b\_Genbank\_for\_inparanoid.fasta:983

```
CQW49_RS00255       	100.00%		CQW49_00255         	100.00%
Bootstrap support for CQW49_RS00255 as seed ortholog is 100%.
Bootstrap support for CQW49_00255 as seed ortholog is 100%.
```

---

### Group of orthologs #613. Best score 983 bits Score difference with first non-orthologous sequence - OB3b\_Refseq\_for\_inparanoid.fasta:983 OB3b\_Genbank\_for\_inparanoid.fasta:983

```
CQW49_RS02545       	100.00%		CQW49_02535         	100.00%
Bootstrap support for CQW49_RS02545 as seed ortholog is 100%.
Bootstrap support for CQW49_02535 as seed ortholog is 100%.
```

---

### Group of orthologs #614. Best score 983 bits Score difference with first non-orthologous sequence - OB3b\_Refseq\_for\_inparanoid.fasta:983 OB3b\_Genbank\_for\_inparanoid.fasta:983

```
CQW49_RS02730       	100.00%		CQW49_02720         	100.00%
Bootstrap support for CQW49_RS02730 as seed ortholog is 100%.
Bootstrap support for CQW49_02720 as seed ortholog is 100%.
```

---

### Group of orthologs #615. Best score 983 bits Score difference with first non-orthologous sequence - OB3b\_Refseq\_for\_inparanoid.fasta:983 OB3b\_Genbank\_for\_inparanoid.fasta:983

```
CQW49_RS10210       	100.00%		CQW49_10185         	100.00%
Bootstrap support for CQW49_RS10210 as seed ortholog is 100%.
Bootstrap support for CQW49_10185 as seed ortholog is 100%.
```

---

### Group of orthologs #616. Best score 983 bits Score difference with first non-orthologous sequence - OB3b\_Refseq\_for\_inparanoid.fasta:983 OB3b\_Genbank\_for\_inparanoid.fasta:983

```
glpK                	100.00%		CQW49_12325         	100.00%
Bootstrap support for glpK as seed ortholog is 100%.
Bootstrap support for CQW49_12325 as seed ortholog is 100%.
```

---

### Group of orthologs #617. Best score 983 bits Score difference with first non-orthologous sequence - OB3b\_Refseq\_for\_inparanoid.fasta:983 OB3b\_Genbank\_for\_inparanoid.fasta:983

```
CQW49_RS19990       	100.00%		CQW49_19925         	100.00%
Bootstrap support for CQW49_RS19990 as seed ortholog is 100%.
Bootstrap support for CQW49_19925 as seed ortholog is 100%.
```

---

### Group of orthologs #618. Best score 982 bits Score difference with first non-orthologous sequence - OB3b\_Refseq\_for\_inparanoid.fasta:982 OB3b\_Genbank\_for\_inparanoid.fasta:982

```
CQW49_RS03440       	100.00%		CQW49_03425         	100.00%
Bootstrap support for CQW49_RS03440 as seed ortholog is 100%.
Bootstrap support for CQW49_03425 as seed ortholog is 100%.
```

---

### Group of orthologs #619. Best score 981 bits Score difference with first non-orthologous sequence - OB3b\_Refseq\_for\_inparanoid.fasta:981 OB3b\_Genbank\_for\_inparanoid.fasta:981

```
CQW49_RS06355       	100.00%		CQW49_06345         	100.00%
Bootstrap support for CQW49_RS06355 as seed ortholog is 100%.
Bootstrap support for CQW49_06345 as seed ortholog is 100%.
```

---

### Group of orthologs #620. Best score 980 bits Score difference with first non-orthologous sequence - OB3b\_Refseq\_for\_inparanoid.fasta:980 OB3b\_Genbank\_for\_inparanoid.fasta:980

```
CQW49_RS03365       	100.00%		CQW49_03355         	100.00%
Bootstrap support for CQW49_RS03365 as seed ortholog is 100%.
Bootstrap support for CQW49_03355 as seed ortholog is 100%.
```

---

### Group of orthologs #621. Best score 979 bits Score difference with first non-orthologous sequence - OB3b\_Refseq\_for\_inparanoid.fasta:979 OB3b\_Genbank\_for\_inparanoid.fasta:979

```
CQW49_RS01030       	100.00%		CQW49_01030         	100.00%
Bootstrap support for CQW49_RS01030 as seed ortholog is 100%.
Bootstrap support for CQW49_01030 as seed ortholog is 100%.
```

---

### Group of orthologs #622. Best score 979 bits Score difference with first non-orthologous sequence - OB3b\_Refseq\_for\_inparanoid.fasta:979 OB3b\_Genbank\_for\_inparanoid.fasta:979

```
CQW49_RS01215       	100.00%		CQW49_01210         	100.00%
Bootstrap support for CQW49_RS01215 as seed ortholog is 100%.
Bootstrap support for CQW49_01210 as seed ortholog is 100%.
```

---

### Group of orthologs #623. Best score 978 bits Score difference with first non-orthologous sequence - OB3b\_Refseq\_for\_inparanoid.fasta:978 OB3b\_Genbank\_for\_inparanoid.fasta:978

```
CQW49_RS18055       	100.00%		CQW49_17995         	100.00%
Bootstrap support for CQW49_RS18055 as seed ortholog is 100%.
Bootstrap support for CQW49_17995 as seed ortholog is 100%.
```

---

### Group of orthologs #624. Best score 977 bits Score difference with first non-orthologous sequence - OB3b\_Refseq\_for\_inparanoid.fasta:977 OB3b\_Genbank\_for\_inparanoid.fasta:977

```
CQW49_RS02235       	100.00%		CQW49_02230         	100.00%
Bootstrap support for CQW49_RS02235 as seed ortholog is 100%.
Bootstrap support for CQW49_02230 as seed ortholog is 100%.
```

---

### Group of orthologs #625. Best score 977 bits Score difference with first non-orthologous sequence - OB3b\_Refseq\_for\_inparanoid.fasta:977 OB3b\_Genbank\_for\_inparanoid.fasta:852

```
CQW49_RS03600       	100.00%		CQW49_03590         	100.00%
Bootstrap support for CQW49_RS03600 as seed ortholog is 100%.
Bootstrap support for CQW49_03590 as seed ortholog is 100%.
```

---

### Group of orthologs #626. Best score 977 bits Score difference with first non-orthologous sequence - OB3b\_Refseq\_for\_inparanoid.fasta:977 OB3b\_Genbank\_for\_inparanoid.fasta:977

```
CQW49_RS04755       	100.00%		CQW49_04745         	100.00%
Bootstrap support for CQW49_RS04755 as seed ortholog is 100%.
Bootstrap support for CQW49_04745 as seed ortholog is 100%.
```

---

### Group of orthologs #627. Best score 977 bits Score difference with first non-orthologous sequence - OB3b\_Refseq\_for\_inparanoid.fasta:977 OB3b\_Genbank\_for\_inparanoid.fasta:977

```
CQW49_RS13080       	100.00%		CQW49_13045         	100.00%
Bootstrap support for CQW49_RS13080 as seed ortholog is 100%.
Bootstrap support for CQW49_13045 as seed ortholog is 100%.
```

---

### Group of orthologs #628. Best score 976 bits Score difference with first non-orthologous sequence - OB3b\_Refseq\_for\_inparanoid.fasta:976 OB3b\_Genbank\_for\_inparanoid.fasta:976

```
CQW49_RS01000       	100.00%		CQW49_01000         	100.00%
Bootstrap support for CQW49_RS01000 as seed ortholog is 100%.
Bootstrap support for CQW49_01000 as seed ortholog is 100%.
```

---

### Group of orthologs #629. Best score 975 bits Score difference with first non-orthologous sequence - OB3b\_Refseq\_for\_inparanoid.fasta:975 OB3b\_Genbank\_for\_inparanoid.fasta:975

```
CQW49_RS10640       	100.00%		CQW49_10610         	100.00%
Bootstrap support for CQW49_RS10640 as seed ortholog is 100%.
Bootstrap support for CQW49_10610 as seed ortholog is 100%.
```

---

### Group of orthologs #630. Best score 975 bits Score difference with first non-orthologous sequence - OB3b\_Refseq\_for\_inparanoid.fasta:975 OB3b\_Genbank\_for\_inparanoid.fasta:975

```
CQW49_RS10960       	100.00%		CQW49_10930         	100.00%
Bootstrap support for CQW49_RS10960 as seed ortholog is 100%.
Bootstrap support for CQW49_10930 as seed ortholog is 100%.
```

---

### Group of orthologs #631. Best score 974 bits Score difference with first non-orthologous sequence - OB3b\_Refseq\_for\_inparanoid.fasta:974 OB3b\_Genbank\_for\_inparanoid.fasta:974

```
CQW49_RS00550       	100.00%		CQW49_00550         	100.00%
Bootstrap support for CQW49_RS00550 as seed ortholog is 100%.
Bootstrap support for CQW49_00550 as seed ortholog is 100%.
```

---

### Group of orthologs #632. Best score 972 bits Score difference with first non-orthologous sequence - OB3b\_Refseq\_for\_inparanoid.fasta:972 OB3b\_Genbank\_for\_inparanoid.fasta:432

```
CQW49_RS03180       	100.00%		CQW49_03170         	100.00%
Bootstrap support for CQW49_RS03180 as seed ortholog is 100%.
Bootstrap support for CQW49_03170 as seed ortholog is 100%.
```

---

### Group of orthologs #633. Best score 971 bits Score difference with first non-orthologous sequence - OB3b\_Refseq\_for\_inparanoid.fasta:971 OB3b\_Genbank\_for\_inparanoid.fasta:971

```
mviN                	100.00%		CQW49_10285         	100.00%
Bootstrap support for mviN as seed ortholog is 100%.
Bootstrap support for CQW49_10285 as seed ortholog is 100%.
```

---

### Group of orthologs #634. Best score 971 bits Score difference with first non-orthologous sequence - OB3b\_Refseq\_for\_inparanoid.fasta:971 OB3b\_Genbank\_for\_inparanoid.fasta:971

```
CQW49_RS20670       	100.00%		CQW49_20595         	100.00%
Bootstrap support for CQW49_RS20670 as seed ortholog is 100%.
Bootstrap support for CQW49_20595 as seed ortholog is 100%.
```

---

### Group of orthologs #635. Best score 970 bits Score difference with first non-orthologous sequence - OB3b\_Refseq\_for\_inparanoid.fasta:970 OB3b\_Genbank\_for\_inparanoid.fasta:970

```
gatB                	100.00%		CQW49_01110         	100.00%
Bootstrap support for gatB as seed ortholog is 100%.
Bootstrap support for CQW49_01110 as seed ortholog is 100%.
```

---

### Group of orthologs #636. Best score 970 bits Score difference with first non-orthologous sequence - OB3b\_Refseq\_for\_inparanoid.fasta:970 OB3b\_Genbank\_for\_inparanoid.fasta:970

```
CQW49_RS09475       	100.00%		CQW49_09455         	100.00%
Bootstrap support for CQW49_RS09475 as seed ortholog is 100%.
Bootstrap support for CQW49_09455 as seed ortholog is 100%.
```

---

### Group of orthologs #637. Best score 969 bits Score difference with first non-orthologous sequence - OB3b\_Refseq\_for\_inparanoid.fasta:969 OB3b\_Genbank\_for\_inparanoid.fasta:969

```
CQW49_RS02025       	100.00%		CQW49_02020         	100.00%
Bootstrap support for CQW49_RS02025 as seed ortholog is 100%.
Bootstrap support for CQW49_02020 as seed ortholog is 100%.
```

---

### Group of orthologs #638. Best score 969 bits Score difference with first non-orthologous sequence - OB3b\_Refseq\_for\_inparanoid.fasta:969 OB3b\_Genbank\_for\_inparanoid.fasta:969

```
CQW49_RS03770       	100.00%		CQW49_03760         	100.00%
Bootstrap support for CQW49_RS03770 as seed ortholog is 100%.
Bootstrap support for CQW49_03760 as seed ortholog is 100%.
```

---

### Group of orthologs #639. Best score 969 bits Score difference with first non-orthologous sequence - OB3b\_Refseq\_for\_inparanoid.fasta:969 OB3b\_Genbank\_for\_inparanoid.fasta:969

```
CQW49_RS07660       	100.00%		CQW49_07650         	100.00%
Bootstrap support for CQW49_RS07660 as seed ortholog is 100%.
Bootstrap support for CQW49_07650 as seed ortholog is 100%.
```

---

### Group of orthologs #640. Best score 969 bits Score difference with first non-orthologous sequence - OB3b\_Refseq\_for\_inparanoid.fasta:969 OB3b\_Genbank\_for\_inparanoid.fasta:969

```
CQW49_RS08850       	100.00%		CQW49_08840         	100.00%
Bootstrap support for CQW49_RS08850 as seed ortholog is 100%.
Bootstrap support for CQW49_08840 as seed ortholog is 100%.
```

---

### Group of orthologs #641. Best score 968 bits Score difference with first non-orthologous sequence - OB3b\_Refseq\_for\_inparanoid.fasta:968 OB3b\_Genbank\_for\_inparanoid.fasta:968

```
CQW49_RS00005       	100.00%		CQW49_00005         	100.00%
Bootstrap support for CQW49_RS00005 as seed ortholog is 100%.
Bootstrap support for CQW49_00005 as seed ortholog is 100%.
```

---

### Group of orthologs #642. Best score 968 bits Score difference with first non-orthologous sequence - OB3b\_Refseq\_for\_inparanoid.fasta:968 OB3b\_Genbank\_for\_inparanoid.fasta:968

```
CQW49_RS18440       	100.00%		CQW49_18380         	100.00%
Bootstrap support for CQW49_RS18440 as seed ortholog is 100%.
Bootstrap support for CQW49_18380 as seed ortholog is 100%.
```

---

### Group of orthologs #643. Best score 967 bits Score difference with first non-orthologous sequence - OB3b\_Refseq\_for\_inparanoid.fasta:967 OB3b\_Genbank\_for\_inparanoid.fasta:967

```
CQW49_RS08145       	100.00%		CQW49_08135         	100.00%
Bootstrap support for CQW49_RS08145 as seed ortholog is 100%.
Bootstrap support for CQW49_08135 as seed ortholog is 100%.
```

---

### Group of orthologs #644. Best score 967 bits Score difference with first non-orthologous sequence - OB3b\_Refseq\_for\_inparanoid.fasta:967 OB3b\_Genbank\_for\_inparanoid.fasta:967

```
CQW49_RS19655       	100.00%		CQW49_19600         	100.00%
Bootstrap support for CQW49_RS19655 as seed ortholog is 100%.
Bootstrap support for CQW49_19600 as seed ortholog is 100%.
```

---

### Group of orthologs #645. Best score 966 bits Score difference with first non-orthologous sequence - OB3b\_Refseq\_for\_inparanoid.fasta:966 OB3b\_Genbank\_for\_inparanoid.fasta:966

```
CQW49_RS13330       	100.00%		CQW49_13295         	100.00%
Bootstrap support for CQW49_RS13330 as seed ortholog is 100%.
Bootstrap support for CQW49_13295 as seed ortholog is 100%.
```

---

### Group of orthologs #646. Best score 966 bits Score difference with first non-orthologous sequence - OB3b\_Refseq\_for\_inparanoid.fasta:966 OB3b\_Genbank\_for\_inparanoid.fasta:966

```
CQW49_RS20000       	100.00%		CQW49_19935         	100.00%
Bootstrap support for CQW49_RS20000 as seed ortholog is 100%.
Bootstrap support for CQW49_19935 as seed ortholog is 100%.
```

---

### Group of orthologs #647. Best score 965 bits Score difference with first non-orthologous sequence - OB3b\_Refseq\_for\_inparanoid.fasta:965 OB3b\_Genbank\_for\_inparanoid.fasta:965

```
gltD                	100.00%		CQW49_02775         	100.00%
Bootstrap support for gltD as seed ortholog is 100%.
Bootstrap support for CQW49_02775 as seed ortholog is 100%.
```

---

### Group of orthologs #648. Best score 964 bits Score difference with first non-orthologous sequence - OB3b\_Refseq\_for\_inparanoid.fasta:964 OB3b\_Genbank\_for\_inparanoid.fasta:964

```
CQW49_RS11275       	100.00%		CQW49_11245         	100.00%
Bootstrap support for CQW49_RS11275 as seed ortholog is 100%.
Bootstrap support for CQW49_11245 as seed ortholog is 100%.
```

---

### Group of orthologs #649. Best score 963 bits Score difference with first non-orthologous sequence - OB3b\_Refseq\_for\_inparanoid.fasta:963 OB3b\_Genbank\_for\_inparanoid.fasta:963

```
CQW49_RS01015       	100.00%		CQW49_01015         	100.00%
Bootstrap support for CQW49_RS01015 as seed ortholog is 100%.
Bootstrap support for CQW49_01015 as seed ortholog is 100%.
```

---

### Group of orthologs #650. Best score 963 bits Score difference with first non-orthologous sequence - OB3b\_Refseq\_for\_inparanoid.fasta:963 OB3b\_Genbank\_for\_inparanoid.fasta:963

```
CQW49_RS03870       	100.00%		CQW49_03860         	100.00%
Bootstrap support for CQW49_RS03870 as seed ortholog is 100%.
Bootstrap support for CQW49_03860 as seed ortholog is 100%.
```

---

### Group of orthologs #651. Best score 962 bits Score difference with first non-orthologous sequence - OB3b\_Refseq\_for\_inparanoid.fasta:962 OB3b\_Genbank\_for\_inparanoid.fasta:962

```
ntrC                	100.00%		CQW49_01660         	100.00%
Bootstrap support for ntrC as seed ortholog is 100%.
Bootstrap support for CQW49_01660 as seed ortholog is 100%.
```

---

### Group of orthologs #652. Best score 961 bits Score difference with first non-orthologous sequence - OB3b\_Refseq\_for\_inparanoid.fasta:961 OB3b\_Genbank\_for\_inparanoid.fasta:961

```
CQW49_RS13240       	100.00%		CQW49_13205         	100.00%
Bootstrap support for CQW49_RS13240 as seed ortholog is 100%.
Bootstrap support for CQW49_13205 as seed ortholog is 100%.
```

---

### Group of orthologs #653. Best score 961 bits Score difference with first non-orthologous sequence - OB3b\_Refseq\_for\_inparanoid.fasta:961 OB3b\_Genbank\_for\_inparanoid.fasta:961

```
glnA                	100.00%		CQW49_20665         	100.00%
Bootstrap support for glnA as seed ortholog is 100%.
Bootstrap support for CQW49_20665 as seed ortholog is 100%.
```

---

### Group of orthologs #654. Best score 960 bits Score difference with first non-orthologous sequence - OB3b\_Refseq\_for\_inparanoid.fasta:960 OB3b\_Genbank\_for\_inparanoid.fasta:960

```
CQW49_RS01020       	100.00%		CQW49_01020         	100.00%
Bootstrap support for CQW49_RS01020 as seed ortholog is 100%.
Bootstrap support for CQW49_01020 as seed ortholog is 100%.
```

---

### Group of orthologs #655. Best score 960 bits Score difference with first non-orthologous sequence - OB3b\_Refseq\_for\_inparanoid.fasta:960 OB3b\_Genbank\_for\_inparanoid.fasta:960

```
CQW49_RS01170       	100.00%		CQW49_01165         	100.00%
Bootstrap support for CQW49_RS01170 as seed ortholog is 100%.
Bootstrap support for CQW49_01165 as seed ortholog is 100%.
```

---

### Group of orthologs #656. Best score 958 bits Score difference with first non-orthologous sequence - OB3b\_Refseq\_for\_inparanoid.fasta:958 OB3b\_Genbank\_for\_inparanoid.fasta:792

```
CQW49_RS02645       	100.00%		CQW49_02635         	100.00%
Bootstrap support for CQW49_RS02645 as seed ortholog is 100%.
Bootstrap support for CQW49_02635 as seed ortholog is 100%.
```

---

### Group of orthologs #657. Best score 957 bits Score difference with first non-orthologous sequence - OB3b\_Refseq\_for\_inparanoid.fasta:957 OB3b\_Genbank\_for\_inparanoid.fasta:957

```
CQW49_RS07080       	100.00%		CQW49_07070         	100.00%
Bootstrap support for CQW49_RS07080 as seed ortholog is 100%.
Bootstrap support for CQW49_07070 as seed ortholog is 100%.
```

---

### Group of orthologs #658. Best score 957 bits Score difference with first non-orthologous sequence - OB3b\_Refseq\_for\_inparanoid.fasta:957 OB3b\_Genbank\_for\_inparanoid.fasta:957

```
CQW49_RS12220       	100.00%		CQW49_12185         	100.00%
Bootstrap support for CQW49_RS12220 as seed ortholog is 100%.
Bootstrap support for CQW49_12185 as seed ortholog is 100%.
```

---

### Group of orthologs #659. Best score 957 bits Score difference with first non-orthologous sequence - OB3b\_Refseq\_for\_inparanoid.fasta:957 OB3b\_Genbank\_for\_inparanoid.fasta:957

```
CQW49_RS12620       	100.00%		CQW49_12585         	100.00%
Bootstrap support for CQW49_RS12620 as seed ortholog is 100%.
Bootstrap support for CQW49_12585 as seed ortholog is 100%.
```

---

### Group of orthologs #660. Best score 957 bits Score difference with first non-orthologous sequence - OB3b\_Refseq\_for\_inparanoid.fasta:957 OB3b\_Genbank\_for\_inparanoid.fasta:957

```
CQW49_RS16345       	100.00%		CQW49_16310         	100.00%
Bootstrap support for CQW49_RS16345 as seed ortholog is 100%.
Bootstrap support for CQW49_16310 as seed ortholog is 100%.
```

---

### Group of orthologs #661. Best score 956 bits Score difference with first non-orthologous sequence - OB3b\_Refseq\_for\_inparanoid.fasta:956 OB3b\_Genbank\_for\_inparanoid.fasta:956

```
CQW49_RS03510       	100.00%		CQW49_03500         	100.00%
Bootstrap support for CQW49_RS03510 as seed ortholog is 100%.
Bootstrap support for CQW49_03500 as seed ortholog is 100%.
```

---

### Group of orthologs #662. Best score 955 bits Score difference with first non-orthologous sequence - OB3b\_Refseq\_for\_inparanoid.fasta:955 OB3b\_Genbank\_for\_inparanoid.fasta:955

```
CQW49_RS06030       	100.00%		CQW49_06020         	100.00%
Bootstrap support for CQW49_RS06030 as seed ortholog is 100%.
Bootstrap support for CQW49_06020 as seed ortholog is 100%.
```

---

### Group of orthologs #663. Best score 954 bits Score difference with first non-orthologous sequence - OB3b\_Refseq\_for\_inparanoid.fasta:954 OB3b\_Genbank\_for\_inparanoid.fasta:954

```
CQW49_RS12065       	100.00%		CQW49_12030         	100.00%
Bootstrap support for CQW49_RS12065 as seed ortholog is 100%.
Bootstrap support for CQW49_12030 as seed ortholog is 100%.
```

---

### Group of orthologs #664. Best score 953 bits Score difference with first non-orthologous sequence - OB3b\_Refseq\_for\_inparanoid.fasta:953 OB3b\_Genbank\_for\_inparanoid.fasta:953

```
CQW49_RS02740       	100.00%		CQW49_02730         	100.00%
Bootstrap support for CQW49_RS02740 as seed ortholog is 100%.
Bootstrap support for CQW49_02730 as seed ortholog is 100%.
```

---

### Group of orthologs #665. Best score 953 bits Score difference with first non-orthologous sequence - OB3b\_Refseq\_for\_inparanoid.fasta:953 OB3b\_Genbank\_for\_inparanoid.fasta:953

```
CQW49_RS15735       	100.00%		CQW49_15700         	100.00%
Bootstrap support for CQW49_RS15735 as seed ortholog is 100%.
Bootstrap support for CQW49_15700 as seed ortholog is 100%.
```

---

### Group of orthologs #666. Best score 949 bits Score difference with first non-orthologous sequence - OB3b\_Refseq\_for\_inparanoid.fasta:949 OB3b\_Genbank\_for\_inparanoid.fasta:872

```
CQW49_RS08360       	100.00%		CQW49_08350         	100.00%
Bootstrap support for CQW49_RS08360 as seed ortholog is 100%.
Bootstrap support for CQW49_08350 as seed ortholog is 100%.
```

---

### Group of orthologs #667. Best score 948 bits Score difference with first non-orthologous sequence - OB3b\_Refseq\_for\_inparanoid.fasta:948 OB3b\_Genbank\_for\_inparanoid.fasta:948

```
CQW49_RS14235       	100.00%		CQW49_14200         	100.00%
Bootstrap support for CQW49_RS14235 as seed ortholog is 100%.
Bootstrap support for CQW49_14200 as seed ortholog is 100%.
```

---

### Group of orthologs #668. Best score 947 bits Score difference with first non-orthologous sequence - OB3b\_Refseq\_for\_inparanoid.fasta:947 OB3b\_Genbank\_for\_inparanoid.fasta:947

```
CQW49_RS02450       	100.00%		CQW49_02440         	100.00%
Bootstrap support for CQW49_RS02450 as seed ortholog is 100%.
Bootstrap support for CQW49_02440 as seed ortholog is 100%.
```

---

### Group of orthologs #669. Best score 947 bits Score difference with first non-orthologous sequence - OB3b\_Refseq\_for\_inparanoid.fasta:947 OB3b\_Genbank\_for\_inparanoid.fasta:947

```
CQW49_RS11250       	100.00%		CQW49_11220         	100.00%
Bootstrap support for CQW49_RS11250 as seed ortholog is 100%.
Bootstrap support for CQW49_11220 as seed ortholog is 100%.
```

---

### Group of orthologs #670. Best score 947 bits Score difference with first non-orthologous sequence - OB3b\_Refseq\_for\_inparanoid.fasta:947 OB3b\_Genbank\_for\_inparanoid.fasta:947

```
CQW49_RS14325       	100.00%		CQW49_14290         	100.00%
Bootstrap support for CQW49_RS14325 as seed ortholog is 100%.
Bootstrap support for CQW49_14290 as seed ortholog is 100%.
```

---

### Group of orthologs #671. Best score 947 bits Score difference with first non-orthologous sequence - OB3b\_Refseq\_for\_inparanoid.fasta:947 OB3b\_Genbank\_for\_inparanoid.fasta:947

```
CQW49_RS15740       	100.00%		CQW49_15705         	100.00%
Bootstrap support for CQW49_RS15740 as seed ortholog is 100%.
Bootstrap support for CQW49_15705 as seed ortholog is 100%.
```

---

### Group of orthologs #672. Best score 945 bits Score difference with first non-orthologous sequence - OB3b\_Refseq\_for\_inparanoid.fasta:945 OB3b\_Genbank\_for\_inparanoid.fasta:945

```
gabD                	100.00%		CQW49_06105         	100.00%
Bootstrap support for gabD as seed ortholog is 100%.
Bootstrap support for CQW49_06105 as seed ortholog is 100%.
```

---

### Group of orthologs #673. Best score 945 bits Score difference with first non-orthologous sequence - OB3b\_Refseq\_for\_inparanoid.fasta:945 OB3b\_Genbank\_for\_inparanoid.fasta:945

```
CQW49_RS16195       	100.00%		CQW49_16160         	100.00%
Bootstrap support for CQW49_RS16195 as seed ortholog is 100%.
Bootstrap support for CQW49_16160 as seed ortholog is 100%.
```

---

### Group of orthologs #674. Best score 943 bits Score difference with first non-orthologous sequence - OB3b\_Refseq\_for\_inparanoid.fasta:943 OB3b\_Genbank\_for\_inparanoid.fasta:943

```
CQW49_RS00515       	100.00%		CQW49_00515         	100.00%
Bootstrap support for CQW49_RS00515 as seed ortholog is 100%.
Bootstrap support for CQW49_00515 as seed ortholog is 100%.
```

---

### Group of orthologs #675. Best score 943 bits Score difference with first non-orthologous sequence - OB3b\_Refseq\_for\_inparanoid.fasta:943 OB3b\_Genbank\_for\_inparanoid.fasta:943

```
leuC                	100.00%		CQW49_15985         	100.00%
Bootstrap support for leuC as seed ortholog is 100%.
Bootstrap support for CQW49_15985 as seed ortholog is 100%.
```

---

### Group of orthologs #676. Best score 942 bits Score difference with first non-orthologous sequence - OB3b\_Refseq\_for\_inparanoid.fasta:942 OB3b\_Genbank\_for\_inparanoid.fasta:942

```
CQW49_RS12165       	100.00%		CQW49_12130         	100.00%
Bootstrap support for CQW49_RS12165 as seed ortholog is 100%.
Bootstrap support for CQW49_12130 as seed ortholog is 100%.
```

---

### Group of orthologs #677. Best score 942 bits Score difference with first non-orthologous sequence - OB3b\_Refseq\_for\_inparanoid.fasta:942 OB3b\_Genbank\_for\_inparanoid.fasta:942

```
CQW49_RS16595       	100.00%		CQW49_16555         	100.00%
Bootstrap support for CQW49_RS16595 as seed ortholog is 100%.
Bootstrap support for CQW49_16555 as seed ortholog is 100%.
```

---

### Group of orthologs #678. Best score 941 bits Score difference with first non-orthologous sequence - OB3b\_Refseq\_for\_inparanoid.fasta:941 OB3b\_Genbank\_for\_inparanoid.fasta:941

```
CQW49_RS12870       	100.00%		CQW49_12835         	100.00%
Bootstrap support for CQW49_RS12870 as seed ortholog is 100%.
Bootstrap support for CQW49_12835 as seed ortholog is 100%.
```

---

### Group of orthologs #679. Best score 941 bits Score difference with first non-orthologous sequence - OB3b\_Refseq\_for\_inparanoid.fasta:941 OB3b\_Genbank\_for\_inparanoid.fasta:941

```
CQW49_RS13490       	100.00%		CQW49_13455         	100.00%
Bootstrap support for CQW49_RS13490 as seed ortholog is 100%.
Bootstrap support for CQW49_13455 as seed ortholog is 100%.
```

---

### Group of orthologs #680. Best score 940 bits Score difference with first non-orthologous sequence - OB3b\_Refseq\_for\_inparanoid.fasta:940 OB3b\_Genbank\_for\_inparanoid.fasta:940

```
lpdA                	100.00%		CQW49_06860         	100.00%
Bootstrap support for lpdA as seed ortholog is 100%.
Bootstrap support for CQW49_06860 as seed ortholog is 100%.
```

---

### Group of orthologs #681. Best score 940 bits Score difference with first non-orthologous sequence - OB3b\_Refseq\_for\_inparanoid.fasta:940 OB3b\_Genbank\_for\_inparanoid.fasta:940

```
CQW49_RS13015       	100.00%		CQW49_12980         	100.00%
Bootstrap support for CQW49_RS13015 as seed ortholog is 100%.
Bootstrap support for CQW49_12980 as seed ortholog is 100%.
```

---

### Group of orthologs #682. Best score 939 bits Score difference with first non-orthologous sequence - OB3b\_Refseq\_for\_inparanoid.fasta:939 OB3b\_Genbank\_for\_inparanoid.fasta:939

```
CQW49_RS19405       	100.00%		CQW49_19350         	100.00%
Bootstrap support for CQW49_RS19405 as seed ortholog is 100%.
Bootstrap support for CQW49_19350 as seed ortholog is 100%.
```

---

### Group of orthologs #683. Best score 938 bits Score difference with first non-orthologous sequence - OB3b\_Refseq\_for\_inparanoid.fasta:938 OB3b\_Genbank\_for\_inparanoid.fasta:938

```
CQW49_RS02965       	100.00%		CQW49_02955         	100.00%
Bootstrap support for CQW49_RS02965 as seed ortholog is 100%.
Bootstrap support for CQW49_02955 as seed ortholog is 100%.
```

---

### Group of orthologs #684. Best score 938 bits Score difference with first non-orthologous sequence - OB3b\_Refseq\_for\_inparanoid.fasta:938 OB3b\_Genbank\_for\_inparanoid.fasta:938

```
CQW49_RS03400       	100.00%		CQW49_03385         	100.00%
Bootstrap support for CQW49_RS03400 as seed ortholog is 100%.
Bootstrap support for CQW49_03385 as seed ortholog is 100%.
```

---

### Group of orthologs #685. Best score 938 bits Score difference with first non-orthologous sequence - OB3b\_Refseq\_for\_inparanoid.fasta:938 OB3b\_Genbank\_for\_inparanoid.fasta:938

```
CQW49_RS08915       	100.00%		CQW49_08905         	100.00%
Bootstrap support for CQW49_RS08915 as seed ortholog is 100%.
Bootstrap support for CQW49_08905 as seed ortholog is 100%.
```

---

### Group of orthologs #686. Best score 936 bits Score difference with first non-orthologous sequence - OB3b\_Refseq\_for\_inparanoid.fasta:936 OB3b\_Genbank\_for\_inparanoid.fasta:936

```
CQW49_RS08720       	100.00%		CQW49_08710         	100.00%
Bootstrap support for CQW49_RS08720 as seed ortholog is 100%.
Bootstrap support for CQW49_08710 as seed ortholog is 100%.
```

---

### Group of orthologs #687. Best score 935 bits Score difference with first non-orthologous sequence - OB3b\_Refseq\_for\_inparanoid.fasta:935 OB3b\_Genbank\_for\_inparanoid.fasta:935

```
CQW49_RS07115       	100.00%		CQW49_07105         	100.00%
Bootstrap support for CQW49_RS07115 as seed ortholog is 100%.
Bootstrap support for CQW49_07105 as seed ortholog is 100%.
```

---

### Group of orthologs #688. Best score 933 bits Score difference with first non-orthologous sequence - OB3b\_Refseq\_for\_inparanoid.fasta:933 OB3b\_Genbank\_for\_inparanoid.fasta:847

```
CQW49_RS19485       	100.00%		CQW49_19430         	100.00%
Bootstrap support for CQW49_RS19485 as seed ortholog is 100%.
Bootstrap support for CQW49_19430 as seed ortholog is 100%.
```

---

### Group of orthologs #689. Best score 933 bits Score difference with first non-orthologous sequence - OB3b\_Refseq\_for\_inparanoid.fasta:933 OB3b\_Genbank\_for\_inparanoid.fasta:933

```
CQW49_RS19890       	100.00%		CQW49_19830         	100.00%
Bootstrap support for CQW49_RS19890 as seed ortholog is 100%.
Bootstrap support for CQW49_19830 as seed ortholog is 100%.
```

---

### Group of orthologs #690. Best score 933 bits Score difference with first non-orthologous sequence - OB3b\_Refseq\_for\_inparanoid.fasta:933 OB3b\_Genbank\_for\_inparanoid.fasta:933

```
CQW49_RS21175       	100.00%		CQW49_21100         	100.00%
Bootstrap support for CQW49_RS21175 as seed ortholog is 100%.
Bootstrap support for CQW49_21100 as seed ortholog is 100%.
```

---

### Group of orthologs #691. Best score 931 bits Score difference with first non-orthologous sequence - OB3b\_Refseq\_for\_inparanoid.fasta:931 OB3b\_Genbank\_for\_inparanoid.fasta:931

```
CQW49_RS03755       	100.00%		CQW49_03745         	100.00%
Bootstrap support for CQW49_RS03755 as seed ortholog is 100%.
Bootstrap support for CQW49_03745 as seed ortholog is 100%.
```

---

### Group of orthologs #692. Best score 930 bits Score difference with first non-orthologous sequence - OB3b\_Refseq\_for\_inparanoid.fasta:930 OB3b\_Genbank\_for\_inparanoid.fasta:844

```
CQW49_RS13630       	100.00%		CQW49_13595         	100.00%
Bootstrap support for CQW49_RS13630 as seed ortholog is 100%.
Bootstrap support for CQW49_13595 as seed ortholog is 100%.
```

---

### Group of orthologs #693. Best score 930 bits Score difference with first non-orthologous sequence - OB3b\_Refseq\_for\_inparanoid.fasta:930 OB3b\_Genbank\_for\_inparanoid.fasta:930

```
CQW49_RS19740       	100.00%		CQW49_19685         	100.00%
Bootstrap support for CQW49_RS19740 as seed ortholog is 100%.
Bootstrap support for CQW49_19685 as seed ortholog is 100%.
```

---

### Group of orthologs #694. Best score 929 bits Score difference with first non-orthologous sequence - OB3b\_Refseq\_for\_inparanoid.fasta:929 OB3b\_Genbank\_for\_inparanoid.fasta:929

```
CQW49_RS11235       	100.00%		CQW49_11205         	100.00%
Bootstrap support for CQW49_RS11235 as seed ortholog is 100%.
Bootstrap support for CQW49_11205 as seed ortholog is 100%.
```

---

### Group of orthologs #695. Best score 929 bits Score difference with first non-orthologous sequence - OB3b\_Refseq\_for\_inparanoid.fasta:929 OB3b\_Genbank\_for\_inparanoid.fasta:929

```
CQW49_RS17405       	100.00%		CQW49_17355         	100.00%
Bootstrap support for CQW49_RS17405 as seed ortholog is 100%.
Bootstrap support for CQW49_17355 as seed ortholog is 100%.
```

---

### Group of orthologs #696. Best score 929 bits Score difference with first non-orthologous sequence - OB3b\_Refseq\_for\_inparanoid.fasta:929 OB3b\_Genbank\_for\_inparanoid.fasta:787

```
CQW49_RS17420       	100.00%		CQW49_17370         	100.00%
Bootstrap support for CQW49_RS17420 as seed ortholog is 100%.
Bootstrap support for CQW49_17370 as seed ortholog is 100%.
```

---

### Group of orthologs #697. Best score 927 bits Score difference with first non-orthologous sequence - OB3b\_Refseq\_for\_inparanoid.fasta:927 OB3b\_Genbank\_for\_inparanoid.fasta:927

```
atpD                	100.00%		CQW49_00305         	100.00%
Bootstrap support for atpD as seed ortholog is 100%.
Bootstrap support for CQW49_00305 as seed ortholog is 100%.
```

---

### Group of orthologs #698. Best score 926 bits Score difference with first non-orthologous sequence - OB3b\_Refseq\_for\_inparanoid.fasta:926 OB3b\_Genbank\_for\_inparanoid.fasta:421

```
CQW49_RS04915       	100.00%		CQW49_04910         	100.00%
Bootstrap support for CQW49_RS04915 as seed ortholog is 100%.
Bootstrap support for CQW49_04910 as seed ortholog is 100%.
```

---

### Group of orthologs #699. Best score 925 bits Score difference with first non-orthologous sequence - OB3b\_Refseq\_for\_inparanoid.fasta:925 OB3b\_Genbank\_for\_inparanoid.fasta:925

```
CQW49_RS02705       	100.00%		CQW49_02695         	100.00%
Bootstrap support for CQW49_RS02705 as seed ortholog is 100%.
Bootstrap support for CQW49_02695 as seed ortholog is 100%.
```

---

### Group of orthologs #700. Best score 924 bits Score difference with first non-orthologous sequence - OB3b\_Refseq\_for\_inparanoid.fasta:924 OB3b\_Genbank\_for\_inparanoid.fasta:924

```
CQW49_RS06070       	100.00%		CQW49_06060         	100.00%
Bootstrap support for CQW49_RS06070 as seed ortholog is 100%.
Bootstrap support for CQW49_06060 as seed ortholog is 100%.
```

---

### Group of orthologs #701. Best score 924 bits Score difference with first non-orthologous sequence - OB3b\_Refseq\_for\_inparanoid.fasta:924 OB3b\_Genbank\_for\_inparanoid.fasta:924

```
CQW49_RS09110       	100.00%		CQW49_09090         	100.00%
Bootstrap support for CQW49_RS09110 as seed ortholog is 100%.
Bootstrap support for CQW49_09090 as seed ortholog is 100%.
```

---

### Group of orthologs #702. Best score 924 bits Score difference with first non-orthologous sequence - OB3b\_Refseq\_for\_inparanoid.fasta:924 OB3b\_Genbank\_for\_inparanoid.fasta:924

```
CQW49_RS18595       	100.00%		CQW49_18535         	100.00%
Bootstrap support for CQW49_RS18595 as seed ortholog is 100%.
Bootstrap support for CQW49_18535 as seed ortholog is 100%.
```

---

### Group of orthologs #703. Best score 924 bits Score difference with first non-orthologous sequence - OB3b\_Refseq\_for\_inparanoid.fasta:924 OB3b\_Genbank\_for\_inparanoid.fasta:924

```
CQW49_RS18745       	100.00%		CQW49_18685         	100.00%
Bootstrap support for CQW49_RS18745 as seed ortholog is 100%.
Bootstrap support for CQW49_18685 as seed ortholog is 100%.
```

---

### Group of orthologs #704. Best score 923 bits Score difference with first non-orthologous sequence - OB3b\_Refseq\_for\_inparanoid.fasta:923 OB3b\_Genbank\_for\_inparanoid.fasta:923

```
CQW49_RS12150       	100.00%		CQW49_12115         	100.00%
Bootstrap support for CQW49_RS12150 as seed ortholog is 100%.
Bootstrap support for CQW49_12115 as seed ortholog is 100%.
```

---

### Group of orthologs #705. Best score 923 bits Score difference with first non-orthologous sequence - OB3b\_Refseq\_for\_inparanoid.fasta:923 OB3b\_Genbank\_for\_inparanoid.fasta:923

```
CQW49_RS19995       	100.00%		CQW49_19930         	100.00%
Bootstrap support for CQW49_RS19995 as seed ortholog is 100%.
Bootstrap support for CQW49_19930 as seed ortholog is 100%.
```

---

### Group of orthologs #706. Best score 922 bits Score difference with first non-orthologous sequence - OB3b\_Refseq\_for\_inparanoid.fasta:922 OB3b\_Genbank\_for\_inparanoid.fasta:922

```
CQW49_RS01760       	100.00%		CQW49_01755         	100.00%
Bootstrap support for CQW49_RS01760 as seed ortholog is 100%.
Bootstrap support for CQW49_01755 as seed ortholog is 100%.
```

---

### Group of orthologs #707. Best score 922 bits Score difference with first non-orthologous sequence - OB3b\_Refseq\_for\_inparanoid.fasta:922 OB3b\_Genbank\_for\_inparanoid.fasta:922

```
CQW49_RS11265       	100.00%		CQW49_11235         	100.00%
Bootstrap support for CQW49_RS11265 as seed ortholog is 100%.
Bootstrap support for CQW49_11235 as seed ortholog is 100%.
```

---

### Group of orthologs #708. Best score 920 bits Score difference with first non-orthologous sequence - OB3b\_Refseq\_for\_inparanoid.fasta:920 OB3b\_Genbank\_for\_inparanoid.fasta:920

```
CQW49_RS06850       	100.00%		CQW49_06840         	100.00%
Bootstrap support for CQW49_RS06850 as seed ortholog is 100%.
Bootstrap support for CQW49_06840 as seed ortholog is 100%.
```

---

### Group of orthologs #709. Best score 920 bits Score difference with first non-orthologous sequence - OB3b\_Refseq\_for\_inparanoid.fasta:920 OB3b\_Genbank\_for\_inparanoid.fasta:920

```
CQW49_RS09980       	100.00%		CQW49_09955         	100.00%
Bootstrap support for CQW49_RS09980 as seed ortholog is 100%.
Bootstrap support for CQW49_09955 as seed ortholog is 100%.
```

---

### Group of orthologs #710. Best score 920 bits Score difference with first non-orthologous sequence - OB3b\_Refseq\_for\_inparanoid.fasta:920 OB3b\_Genbank\_for\_inparanoid.fasta:920

```
CQW49_RS15845       	100.00%		CQW49_15810         	100.00%
Bootstrap support for CQW49_RS15845 as seed ortholog is 100%.
Bootstrap support for CQW49_15810 as seed ortholog is 100%.
```

---

### Group of orthologs #711. Best score 919 bits Score difference with first non-orthologous sequence - OB3b\_Refseq\_for\_inparanoid.fasta:919 OB3b\_Genbank\_for\_inparanoid.fasta:828

```
CQW49_RS06280       	100.00%		CQW49_06270         	100.00%
Bootstrap support for CQW49_RS06280 as seed ortholog is 100%.
Bootstrap support for CQW49_06270 as seed ortholog is 100%.
```

---

### Group of orthologs #712. Best score 919 bits Score difference with first non-orthologous sequence - OB3b\_Refseq\_for\_inparanoid.fasta:919 OB3b\_Genbank\_for\_inparanoid.fasta:919

```
CQW49_RS09180       	100.00%		CQW49_09160         	100.00%
Bootstrap support for CQW49_RS09180 as seed ortholog is 100%.
Bootstrap support for CQW49_09160 as seed ortholog is 100%.
```

---

### Group of orthologs #713. Best score 918 bits Score difference with first non-orthologous sequence - OB3b\_Refseq\_for\_inparanoid.fasta:918 OB3b\_Genbank\_for\_inparanoid.fasta:918

```
CQW49_RS04225       	100.00%		CQW49_04215         	100.00%
Bootstrap support for CQW49_RS04225 as seed ortholog is 100%.
Bootstrap support for CQW49_04215 as seed ortholog is 100%.
```

---

### Group of orthologs #714. Best score 918 bits Score difference with first non-orthologous sequence - OB3b\_Refseq\_for\_inparanoid.fasta:918 OB3b\_Genbank\_for\_inparanoid.fasta:918

```
CQW49_RS11070       	100.00%		CQW49_11040         	100.00%
Bootstrap support for CQW49_RS11070 as seed ortholog is 100%.
Bootstrap support for CQW49_11040 as seed ortholog is 100%.
```

---

### Group of orthologs #715. Best score 914 bits Score difference with first non-orthologous sequence - OB3b\_Refseq\_for\_inparanoid.fasta:914 OB3b\_Genbank\_for\_inparanoid.fasta:914

```
CQW49_RS15615       	100.00%		CQW49_15580         	100.00%
Bootstrap support for CQW49_RS15615 as seed ortholog is 100%.
Bootstrap support for CQW49_15580 as seed ortholog is 100%.
```

---

### Group of orthologs #716. Best score 913 bits Score difference with first non-orthologous sequence - OB3b\_Refseq\_for\_inparanoid.fasta:913 OB3b\_Genbank\_for\_inparanoid.fasta:913

```
CQW49_RS08080       	100.00%		CQW49_08070         	100.00%
Bootstrap support for CQW49_RS08080 as seed ortholog is 100%.
Bootstrap support for CQW49_08070 as seed ortholog is 100%.
```

---

### Group of orthologs #717. Best score 913 bits Score difference with first non-orthologous sequence - OB3b\_Refseq\_for\_inparanoid.fasta:913 OB3b\_Genbank\_for\_inparanoid.fasta:913

```
CQW49_RS20290       	100.00%		CQW49_20220         	100.00%
Bootstrap support for CQW49_RS20290 as seed ortholog is 100%.
Bootstrap support for CQW49_20220 as seed ortholog is 100%.
```

---

### Group of orthologs #718. Best score 913 bits Score difference with first non-orthologous sequence - OB3b\_Refseq\_for\_inparanoid.fasta:913 OB3b\_Genbank\_for\_inparanoid.fasta:913

```
CQW49_RS20900       	100.00%		CQW49_20825         	100.00%
Bootstrap support for CQW49_RS20900 as seed ortholog is 100%.
Bootstrap support for CQW49_20825 as seed ortholog is 100%.
```

---

### Group of orthologs #719. Best score 912 bits Score difference with first non-orthologous sequence - OB3b\_Refseq\_for\_inparanoid.fasta:912 OB3b\_Genbank\_for\_inparanoid.fasta:912

```
CQW49_RS05725       	100.00%		CQW49_05715         	100.00%
Bootstrap support for CQW49_RS05725 as seed ortholog is 100%.
Bootstrap support for CQW49_05715 as seed ortholog is 100%.
```

---

### Group of orthologs #720. Best score 912 bits Score difference with first non-orthologous sequence - OB3b\_Refseq\_for\_inparanoid.fasta:912 OB3b\_Genbank\_for\_inparanoid.fasta:912

```
CQW49_RS18090       	100.00%		CQW49_18030         	100.00%
Bootstrap support for CQW49_RS18090 as seed ortholog is 100%.
Bootstrap support for CQW49_18030 as seed ortholog is 100%.
```

---

### Group of orthologs #721. Best score 911 bits Score difference with first non-orthologous sequence - OB3b\_Refseq\_for\_inparanoid.fasta:911 OB3b\_Genbank\_for\_inparanoid.fasta:911

```
CQW49_RS11240       	100.00%		CQW49_11210         	100.00%
Bootstrap support for CQW49_RS11240 as seed ortholog is 100%.
Bootstrap support for CQW49_11210 as seed ortholog is 100%.
```

---

### Group of orthologs #722. Best score 911 bits Score difference with first non-orthologous sequence - OB3b\_Refseq\_for\_inparanoid.fasta:911 OB3b\_Genbank\_for\_inparanoid.fasta:911

```
CQW49_RS16445       	100.00%		CQW49_16410         	100.00%
Bootstrap support for CQW49_RS16445 as seed ortholog is 100%.
Bootstrap support for CQW49_16410 as seed ortholog is 100%.
```

---

### Group of orthologs #723. Best score 910 bits Score difference with first non-orthologous sequence - OB3b\_Refseq\_for\_inparanoid.fasta:910 OB3b\_Genbank\_for\_inparanoid.fasta:910

```
CQW49_RS00075       	100.00%		CQW49_00075         	100.00%
Bootstrap support for CQW49_RS00075 as seed ortholog is 100%.
Bootstrap support for CQW49_00075 as seed ortholog is 100%.
```

---

### Group of orthologs #724. Best score 910 bits Score difference with first non-orthologous sequence - OB3b\_Refseq\_for\_inparanoid.fasta:910 OB3b\_Genbank\_for\_inparanoid.fasta:910

```
CQW49_RS06345       	100.00%		CQW49_06335         	100.00%
Bootstrap support for CQW49_RS06345 as seed ortholog is 100%.
Bootstrap support for CQW49_06335 as seed ortholog is 100%.
```

---

### Group of orthologs #725. Best score 910 bits Score difference with first non-orthologous sequence - OB3b\_Refseq\_for\_inparanoid.fasta:910 OB3b\_Genbank\_for\_inparanoid.fasta:910

```
fumC                	100.00%		CQW49_13245         	100.00%
Bootstrap support for fumC as seed ortholog is 100%.
Bootstrap support for CQW49_13245 as seed ortholog is 100%.
```

---

### Group of orthologs #726. Best score 910 bits Score difference with first non-orthologous sequence - OB3b\_Refseq\_for\_inparanoid.fasta:910 OB3b\_Genbank\_for\_inparanoid.fasta:910

```
CQW49_RS14210       	100.00%		CQW49_14175         	100.00%
Bootstrap support for CQW49_RS14210 as seed ortholog is 100%.
Bootstrap support for CQW49_14175 as seed ortholog is 100%.
```

---

### Group of orthologs #727. Best score 909 bits Score difference with first non-orthologous sequence - OB3b\_Refseq\_for\_inparanoid.fasta:909 OB3b\_Genbank\_for\_inparanoid.fasta:909

```
accC                	100.00%		CQW49_05015         	100.00%
Bootstrap support for accC as seed ortholog is 100%.
Bootstrap support for CQW49_05015 as seed ortholog is 100%.
```

---

### Group of orthologs #728. Best score 909 bits Score difference with first non-orthologous sequence - OB3b\_Refseq\_for\_inparanoid.fasta:909 OB3b\_Genbank\_for\_inparanoid.fasta:909

```
CQW49_RS15570       	100.00%		CQW49_15535         	100.00%
Bootstrap support for CQW49_RS15570 as seed ortholog is 100%.
Bootstrap support for CQW49_15535 as seed ortholog is 100%.
```

---

### Group of orthologs #729. Best score 909 bits Score difference with first non-orthologous sequence - OB3b\_Refseq\_for\_inparanoid.fasta:909 OB3b\_Genbank\_for\_inparanoid.fasta:909

```
CQW49_RS17600       	100.00%		CQW49_17545         	100.00%
Bootstrap support for CQW49_RS17600 as seed ortholog is 100%.
Bootstrap support for CQW49_17545 as seed ortholog is 100%.
```

---

### Group of orthologs #730. Best score 909 bits Score difference with first non-orthologous sequence - OB3b\_Refseq\_for\_inparanoid.fasta:909 OB3b\_Genbank\_for\_inparanoid.fasta:909

```
CQW49_RS18070       	100.00%		CQW49_18010         	100.00%
Bootstrap support for CQW49_RS18070 as seed ortholog is 100%.
Bootstrap support for CQW49_18010 as seed ortholog is 100%.
```

---

### Group of orthologs #731. Best score 908 bits Score difference with first non-orthologous sequence - OB3b\_Refseq\_for\_inparanoid.fasta:908 OB3b\_Genbank\_for\_inparanoid.fasta:908

```
pyk                 	100.00%		CQW49_01145         	100.00%
Bootstrap support for pyk as seed ortholog is 100%.
Bootstrap support for CQW49_01145 as seed ortholog is 100%.
```

---

### Group of orthologs #732. Best score 908 bits Score difference with first non-orthologous sequence - OB3b\_Refseq\_for\_inparanoid.fasta:908 OB3b\_Genbank\_for\_inparanoid.fasta:908

```
CQW49_RS01910       	100.00%		CQW49_01905         	100.00%
Bootstrap support for CQW49_RS01910 as seed ortholog is 100%.
Bootstrap support for CQW49_01905 as seed ortholog is 100%.
```

---

### Group of orthologs #733. Best score 908 bits Score difference with first non-orthologous sequence - OB3b\_Refseq\_for\_inparanoid.fasta:908 OB3b\_Genbank\_for\_inparanoid.fasta:908

```
CQW49_RS08995       	100.00%		CQW49_08985         	100.00%
Bootstrap support for CQW49_RS08995 as seed ortholog is 100%.
Bootstrap support for CQW49_08985 as seed ortholog is 100%.
```

---

### Group of orthologs #734. Best score 908 bits Score difference with first non-orthologous sequence - OB3b\_Refseq\_for\_inparanoid.fasta:908 OB3b\_Genbank\_for\_inparanoid.fasta:908

```
CQW49_RS16820       	100.00%		CQW49_16780         	100.00%
Bootstrap support for CQW49_RS16820 as seed ortholog is 100%.
Bootstrap support for CQW49_16780 as seed ortholog is 100%.
```

---

### Group of orthologs #735. Best score 907 bits Score difference with first non-orthologous sequence - OB3b\_Refseq\_for\_inparanoid.fasta:907 OB3b\_Genbank\_for\_inparanoid.fasta:907

```
CQW49_RS06595       	100.00%		CQW49_06580         	100.00%
Bootstrap support for CQW49_RS06595 as seed ortholog is 100%.
Bootstrap support for CQW49_06580 as seed ortholog is 100%.
```

---

### Group of orthologs #736. Best score 907 bits Score difference with first non-orthologous sequence - OB3b\_Refseq\_for\_inparanoid.fasta:907 OB3b\_Genbank\_for\_inparanoid.fasta:907

```
CQW49_RS09370       	100.00%		CQW49_09350         	100.00%
Bootstrap support for CQW49_RS09370 as seed ortholog is 100%.
Bootstrap support for CQW49_09350 as seed ortholog is 100%.
```

---

### Group of orthologs #737. Best score 907 bits Score difference with first non-orthologous sequence - OB3b\_Refseq\_for\_inparanoid.fasta:907 OB3b\_Genbank\_for\_inparanoid.fasta:907

```
CQW49_RS11100       	100.00%		CQW49_11070         	100.00%
Bootstrap support for CQW49_RS11100 as seed ortholog is 100%.
Bootstrap support for CQW49_11070 as seed ortholog is 100%.
```

---

### Group of orthologs #738. Best score 906 bits Score difference with first non-orthologous sequence - OB3b\_Refseq\_for\_inparanoid.fasta:906 OB3b\_Genbank\_for\_inparanoid.fasta:906

```
CQW49_RS01885       	100.00%		CQW49_01880         	100.00%
CQW49_RS08015       	100.00%		CQW49_08005         	100.00%
CQW49_RS08285       	100.00%		CQW49_08275         	100.00%
CQW49_RS13865       	100.00%		CQW49_13830         	100.00%
CQW49_RS14805       	100.00%		CQW49_14770         	100.00%
CQW49_RS19625       	100.00%		CQW49_19570         	100.00%
CQW49_RS01640       	100.00%		CQW49_01635         	100.00%
Bootstrap support for CQW49_RS01885 as seed ortholog is 100%.
Bootstrap support for CQW49_RS08015 as seed ortholog is 100%.
Bootstrap support for CQW49_RS08285 as seed ortholog is 100%.
Bootstrap support for CQW49_RS13865 as seed ortholog is 100%.
Bootstrap support for CQW49_RS14805 as seed ortholog is 100%.
Bootstrap support for CQW49_RS19625 as seed ortholog is 100%.
Bootstrap support for CQW49_RS01640 as seed ortholog is 100%.
Bootstrap support for CQW49_01880 as seed ortholog is 100%.
Bootstrap support for CQW49_08005 as seed ortholog is 100%.
Bootstrap support for CQW49_08275 as seed ortholog is 100%.
Bootstrap support for CQW49_13830 as seed ortholog is 100%.
Bootstrap support for CQW49_14770 as seed ortholog is 100%.
Bootstrap support for CQW49_19570 as seed ortholog is 100%.
Bootstrap support for CQW49_01635 as seed ortholog is 100%.
```

---

### Group of orthologs #739. Best score 906 bits Score difference with first non-orthologous sequence - OB3b\_Refseq\_for\_inparanoid.fasta:906 OB3b\_Genbank\_for\_inparanoid.fasta:906

```
CQW49_RS01950       	100.00%		CQW49_01945         	100.00%
Bootstrap support for CQW49_RS01950 as seed ortholog is 100%.
Bootstrap support for CQW49_01945 as seed ortholog is 100%.
```

---

### Group of orthologs #740. Best score 906 bits Score difference with first non-orthologous sequence - OB3b\_Refseq\_for\_inparanoid.fasta:906 OB3b\_Genbank\_for\_inparanoid.fasta:906

```
mgtE                	100.00%		CQW49_10650         	100.00%
Bootstrap support for mgtE as seed ortholog is 100%.
Bootstrap support for CQW49_10650 as seed ortholog is 100%.
```

---

### Group of orthologs #741. Best score 906 bits Score difference with first non-orthologous sequence - OB3b\_Refseq\_for\_inparanoid.fasta:906 OB3b\_Genbank\_for\_inparanoid.fasta:786

```
CQW49_RS18205       	100.00%		CQW49_18145         	100.00%
Bootstrap support for CQW49_RS18205 as seed ortholog is 100%.
Bootstrap support for CQW49_18145 as seed ortholog is 100%.
```

---

### Group of orthologs #742. Best score 905 bits Score difference with first non-orthologous sequence - OB3b\_Refseq\_for\_inparanoid.fasta:905 OB3b\_Genbank\_for\_inparanoid.fasta:905

```
CQW49_RS09070       	100.00%		CQW49_09055         	100.00%
Bootstrap support for CQW49_RS09070 as seed ortholog is 100%.
Bootstrap support for CQW49_09055 as seed ortholog is 100%.
```

---

### Group of orthologs #743. Best score 905 bits Score difference with first non-orthologous sequence - OB3b\_Refseq\_for\_inparanoid.fasta:905 OB3b\_Genbank\_for\_inparanoid.fasta:905

```
CQW49_RS10025       	100.00%		CQW49_10000         	100.00%
Bootstrap support for CQW49_RS10025 as seed ortholog is 100%.
Bootstrap support for CQW49_10000 as seed ortholog is 100%.
```

---

### Group of orthologs #744. Best score 904 bits Score difference with first non-orthologous sequence - OB3b\_Refseq\_for\_inparanoid.fasta:904 OB3b\_Genbank\_for\_inparanoid.fasta:904

```
CQW49_RS01260       	100.00%		CQW49_01255         	100.00%
Bootstrap support for CQW49_RS01260 as seed ortholog is 100%.
Bootstrap support for CQW49_01255 as seed ortholog is 100%.
```

---

### Group of orthologs #745. Best score 903 bits Score difference with first non-orthologous sequence - OB3b\_Refseq\_for\_inparanoid.fasta:903 OB3b\_Genbank\_for\_inparanoid.fasta:903

```
CQW49_RS05290       	100.00%		CQW49_05285         	100.00%
Bootstrap support for CQW49_RS05290 as seed ortholog is 100%.
Bootstrap support for CQW49_05285 as seed ortholog is 100%.
```

---

### Group of orthologs #746. Best score 903 bits Score difference with first non-orthologous sequence - OB3b\_Refseq\_for\_inparanoid.fasta:903 OB3b\_Genbank\_for\_inparanoid.fasta:903

```
CQW49_RS09465       	100.00%		CQW49_09445         	100.00%
Bootstrap support for CQW49_RS09465 as seed ortholog is 100%.
Bootstrap support for CQW49_09445 as seed ortholog is 100%.
```

---

### Group of orthologs #747. Best score 903 bits Score difference with first non-orthologous sequence - OB3b\_Refseq\_for\_inparanoid.fasta:903 OB3b\_Genbank\_for\_inparanoid.fasta:813

```
CQW49_RS20200       	100.00%		CQW49_20135         	100.00%
Bootstrap support for CQW49_RS20200 as seed ortholog is 100%.
Bootstrap support for CQW49_20135 as seed ortholog is 100%.
```

---

### Group of orthologs #748. Best score 902 bits Score difference with first non-orthologous sequence - OB3b\_Refseq\_for\_inparanoid.fasta:902 OB3b\_Genbank\_for\_inparanoid.fasta:902

```
CQW49_RS08260       	100.00%		CQW49_08250         	100.00%
Bootstrap support for CQW49_RS08260 as seed ortholog is 100%.
Bootstrap support for CQW49_08250 as seed ortholog is 100%.
```

---

### Group of orthologs #749. Best score 902 bits Score difference with first non-orthologous sequence - OB3b\_Refseq\_for\_inparanoid.fasta:902 OB3b\_Genbank\_for\_inparanoid.fasta:902

```
CQW49_RS10385       	100.00%		CQW49_10360         	100.00%
Bootstrap support for CQW49_RS10385 as seed ortholog is 100%.
Bootstrap support for CQW49_10360 as seed ortholog is 100%.
```

---

### Group of orthologs #750. Best score 902 bits Score difference with first non-orthologous sequence - OB3b\_Refseq\_for\_inparanoid.fasta:902 OB3b\_Genbank\_for\_inparanoid.fasta:902

```
CQW49_RS12865       	100.00%		CQW49_12830         	100.00%
Bootstrap support for CQW49_RS12865 as seed ortholog is 100%.
Bootstrap support for CQW49_12830 as seed ortholog is 100%.
```

---

### Group of orthologs #751. Best score 901 bits Score difference with first non-orthologous sequence - OB3b\_Refseq\_for\_inparanoid.fasta:901 OB3b\_Genbank\_for\_inparanoid.fasta:901

```
CQW49_RS15555       	100.00%		CQW49_15520         	100.00%
Bootstrap support for CQW49_RS15555 as seed ortholog is 100%.
Bootstrap support for CQW49_15520 as seed ortholog is 100%.
```

---

### Group of orthologs #752. Best score 901 bits Score difference with first non-orthologous sequence - OB3b\_Refseq\_for\_inparanoid.fasta:901 OB3b\_Genbank\_for\_inparanoid.fasta:901

```
CQW49_RS15745       	100.00%		CQW49_15710         	100.00%
Bootstrap support for CQW49_RS15745 as seed ortholog is 100%.
Bootstrap support for CQW49_15710 as seed ortholog is 100%.
```

---

### Group of orthologs #753. Best score 900 bits Score difference with first non-orthologous sequence - OB3b\_Refseq\_for\_inparanoid.fasta:900 OB3b\_Genbank\_for\_inparanoid.fasta:900

```
CQW49_RS01025       	100.00%		CQW49_01025         	100.00%
Bootstrap support for CQW49_RS01025 as seed ortholog is 100%.
Bootstrap support for CQW49_01025 as seed ortholog is 100%.
```

---

### Group of orthologs #754. Best score 899 bits Score difference with first non-orthologous sequence - OB3b\_Refseq\_for\_inparanoid.fasta:899 OB3b\_Genbank\_for\_inparanoid.fasta:899

```
CQW49_RS02925       	100.00%		CQW49_02915         	100.00%
Bootstrap support for CQW49_RS02925 as seed ortholog is 100%.
Bootstrap support for CQW49_02915 as seed ortholog is 100%.
```

---

### Group of orthologs #755. Best score 899 bits Score difference with first non-orthologous sequence - OB3b\_Refseq\_for\_inparanoid.fasta:899 OB3b\_Genbank\_for\_inparanoid.fasta:899

```
CQW49_RS19660       	100.00%		CQW49_19605         	100.00%
Bootstrap support for CQW49_RS19660 as seed ortholog is 100%.
Bootstrap support for CQW49_19605 as seed ortholog is 100%.
```

---

### Group of orthologs #756. Best score 898 bits Score difference with first non-orthologous sequence - OB3b\_Refseq\_for\_inparanoid.fasta:898 OB3b\_Genbank\_for\_inparanoid.fasta:898

```
CQW49_RS15495       	100.00%		CQW49_15460         	100.00%
Bootstrap support for CQW49_RS15495 as seed ortholog is 100%.
Bootstrap support for CQW49_15460 as seed ortholog is 100%.
```

---

### Group of orthologs #757. Best score 898 bits Score difference with first non-orthologous sequence - OB3b\_Refseq\_for\_inparanoid.fasta:898 OB3b\_Genbank\_for\_inparanoid.fasta:898

```
CQW49_RS20780       	100.00%		CQW49_20705         	100.00%
Bootstrap support for CQW49_RS20780 as seed ortholog is 100%.
Bootstrap support for CQW49_20705 as seed ortholog is 100%.
```

---

### Group of orthologs #758. Best score 897 bits Score difference with first non-orthologous sequence - OB3b\_Refseq\_for\_inparanoid.fasta:897 OB3b\_Genbank\_for\_inparanoid.fasta:897

```
CQW49_RS10865       	100.00%		CQW49_10835         	100.00%
Bootstrap support for CQW49_RS10865 as seed ortholog is 100%.
Bootstrap support for CQW49_10835 as seed ortholog is 100%.
```

---

### Group of orthologs #759. Best score 896 bits Score difference with first non-orthologous sequence - OB3b\_Refseq\_for\_inparanoid.fasta:896 OB3b\_Genbank\_for\_inparanoid.fasta:896

```
CQW49_RS05650       	100.00%		CQW49_05640         	100.00%
Bootstrap support for CQW49_RS05650 as seed ortholog is 100%.
Bootstrap support for CQW49_05640 as seed ortholog is 100%.
```

---

### Group of orthologs #760. Best score 896 bits Score difference with first non-orthologous sequence - OB3b\_Refseq\_for\_inparanoid.fasta:896 OB3b\_Genbank\_for\_inparanoid.fasta:896

```
cobA                	100.00%		CQW49_21195         	100.00%
Bootstrap support for cobA as seed ortholog is 100%.
Bootstrap support for CQW49_21195 as seed ortholog is 100%.
```

---

### Group of orthologs #761. Best score 895 bits Score difference with first non-orthologous sequence - OB3b\_Refseq\_for\_inparanoid.fasta:895 OB3b\_Genbank\_for\_inparanoid.fasta:895

```
hflX                	100.00%		CQW49_01625         	100.00%
Bootstrap support for hflX as seed ortholog is 100%.
Bootstrap support for CQW49_01625 as seed ortholog is 100%.
```

---

### Group of orthologs #762. Best score 895 bits Score difference with first non-orthologous sequence - OB3b\_Refseq\_for\_inparanoid.fasta:895 OB3b\_Genbank\_for\_inparanoid.fasta:734

```
CQW49_RS18555       	100.00%		CQW49_18495         	100.00%
Bootstrap support for CQW49_RS18555 as seed ortholog is 100%.
Bootstrap support for CQW49_18495 as seed ortholog is 100%.
```

---

### Group of orthologs #763. Best score 894 bits Score difference with first non-orthologous sequence - OB3b\_Refseq\_for\_inparanoid.fasta:894 OB3b\_Genbank\_for\_inparanoid.fasta:894

```
CQW49_RS05330       	100.00%		CQW49_05325         	100.00%
Bootstrap support for CQW49_RS05330 as seed ortholog is 100%.
Bootstrap support for CQW49_05325 as seed ortholog is 100%.
```

---

### Group of orthologs #764. Best score 894 bits Score difference with first non-orthologous sequence - OB3b\_Refseq\_for\_inparanoid.fasta:894 OB3b\_Genbank\_for\_inparanoid.fasta:894

```
argH                	100.00%		CQW49_11950         	100.00%
Bootstrap support for argH as seed ortholog is 100%.
Bootstrap support for CQW49_11950 as seed ortholog is 100%.
```

---

### Group of orthologs #765. Best score 894 bits Score difference with first non-orthologous sequence - OB3b\_Refseq\_for\_inparanoid.fasta:894 OB3b\_Genbank\_for\_inparanoid.fasta:894

```
CQW49_RS13105       	100.00%		CQW49_13070         	100.00%
Bootstrap support for CQW49_RS13105 as seed ortholog is 100%.
Bootstrap support for CQW49_13070 as seed ortholog is 100%.
```

---

### Group of orthologs #766. Best score 894 bits Score difference with first non-orthologous sequence - OB3b\_Refseq\_for\_inparanoid.fasta:894 OB3b\_Genbank\_for\_inparanoid.fasta:894

```
CQW49_RS19925       	100.00%		CQW49_19860         	100.00%
Bootstrap support for CQW49_RS19925 as seed ortholog is 100%.
Bootstrap support for CQW49_19860 as seed ortholog is 100%.
```

---

### Group of orthologs #767. Best score 893 bits Score difference with first non-orthologous sequence - OB3b\_Refseq\_for\_inparanoid.fasta:893 OB3b\_Genbank\_for\_inparanoid.fasta:893

```
CQW49_RS00740       	100.00%		CQW49_00740         	100.00%
Bootstrap support for CQW49_RS00740 as seed ortholog is 100%.
Bootstrap support for CQW49_00740 as seed ortholog is 100%.
```

---

### Group of orthologs #768. Best score 893 bits Score difference with first non-orthologous sequence - OB3b\_Refseq\_for\_inparanoid.fasta:893 OB3b\_Genbank\_for\_inparanoid.fasta:803

```
CQW49_RS03280       	100.00%		CQW49_03270         	100.00%
Bootstrap support for CQW49_RS03280 as seed ortholog is 100%.
Bootstrap support for CQW49_03270 as seed ortholog is 100%.
```

---

### Group of orthologs #769. Best score 893 bits Score difference with first non-orthologous sequence - OB3b\_Refseq\_for\_inparanoid.fasta:893 OB3b\_Genbank\_for\_inparanoid.fasta:893

```
CQW49_RS20570       	100.00%		CQW49_20500         	100.00%
Bootstrap support for CQW49_RS20570 as seed ortholog is 100%.
Bootstrap support for CQW49_20500 as seed ortholog is 100%.
```

---

### Group of orthologs #770. Best score 892 bits Score difference with first non-orthologous sequence - OB3b\_Refseq\_for\_inparanoid.fasta:892 OB3b\_Genbank\_for\_inparanoid.fasta:892

```
CQW49_RS09030       	100.00%		CQW49_09015         	100.00%
Bootstrap support for CQW49_RS09030 as seed ortholog is 100%.
Bootstrap support for CQW49_09015 as seed ortholog is 100%.
```

---

### Group of orthologs #771. Best score 892 bits Score difference with first non-orthologous sequence - OB3b\_Refseq\_for\_inparanoid.fasta:892 OB3b\_Genbank\_for\_inparanoid.fasta:892

```
CQW49_RS17965       	100.00%		CQW49_17910         	100.00%
Bootstrap support for CQW49_RS17965 as seed ortholog is 100%.
Bootstrap support for CQW49_17910 as seed ortholog is 100%.
```

---

### Group of orthologs #772. Best score 891 bits Score difference with first non-orthologous sequence - OB3b\_Refseq\_for\_inparanoid.fasta:891 OB3b\_Genbank\_for\_inparanoid.fasta:807

```
CQW49_RS04335       	100.00%		CQW49_04330         	100.00%
Bootstrap support for CQW49_RS04335 as seed ortholog is 100%.
Bootstrap support for CQW49_04330 as seed ortholog is 100%.
```

---

### Group of orthologs #773. Best score 890 bits Score difference with first non-orthologous sequence - OB3b\_Refseq\_for\_inparanoid.fasta:890 OB3b\_Genbank\_for\_inparanoid.fasta:890

```
CQW49_RS02805       	100.00%		CQW49_02795         	100.00%
Bootstrap support for CQW49_RS02805 as seed ortholog is 100%.
Bootstrap support for CQW49_02795 as seed ortholog is 100%.
```

---

### Group of orthologs #774. Best score 889 bits Score difference with first non-orthologous sequence - OB3b\_Refseq\_for\_inparanoid.fasta:889 OB3b\_Genbank\_for\_inparanoid.fasta:889

```
CQW49_RS09130       	100.00%		CQW49_09110         	100.00%
Bootstrap support for CQW49_RS09130 as seed ortholog is 100%.
Bootstrap support for CQW49_09110 as seed ortholog is 100%.
```

---

### Group of orthologs #775. Best score 889 bits Score difference with first non-orthologous sequence - OB3b\_Refseq\_for\_inparanoid.fasta:889 OB3b\_Genbank\_for\_inparanoid.fasta:889

```
CQW49_RS13365       	100.00%		CQW49_13330         	100.00%
Bootstrap support for CQW49_RS13365 as seed ortholog is 100%.
Bootstrap support for CQW49_13330 as seed ortholog is 100%.
```

---

### Group of orthologs #776. Best score 889 bits Score difference with first non-orthologous sequence - OB3b\_Refseq\_for\_inparanoid.fasta:889 OB3b\_Genbank\_for\_inparanoid.fasta:800

```
CQW49_RS13610       	100.00%		CQW49_13575         	100.00%
Bootstrap support for CQW49_RS13610 as seed ortholog is 100%.
Bootstrap support for CQW49_13575 as seed ortholog is 100%.
```

---

### Group of orthologs #777. Best score 889 bits Score difference with first non-orthologous sequence - OB3b\_Refseq\_for\_inparanoid.fasta:889 OB3b\_Genbank\_for\_inparanoid.fasta:832

```
CQW49_RS16755       	100.00%		CQW49_16715         	100.00%
Bootstrap support for CQW49_RS16755 as seed ortholog is 100%.
Bootstrap support for CQW49_16715 as seed ortholog is 100%.
```

---

### Group of orthologs #778. Best score 889 bits Score difference with first non-orthologous sequence - OB3b\_Refseq\_for\_inparanoid.fasta:889 OB3b\_Genbank\_for\_inparanoid.fasta:889

```
CQW49_RS17275       	100.00%		CQW49_17230         	100.00%
Bootstrap support for CQW49_RS17275 as seed ortholog is 100%.
Bootstrap support for CQW49_17230 as seed ortholog is 100%.
```

---

### Group of orthologs #779. Best score 887 bits Score difference with first non-orthologous sequence - OB3b\_Refseq\_for\_inparanoid.fasta:887 OB3b\_Genbank\_for\_inparanoid.fasta:887

```
CQW49_RS02540       	100.00%		CQW49_02530         	100.00%
Bootstrap support for CQW49_RS02540 as seed ortholog is 100%.
Bootstrap support for CQW49_02530 as seed ortholog is 100%.
```

---

### Group of orthologs #780. Best score 887 bits Score difference with first non-orthologous sequence - OB3b\_Refseq\_for\_inparanoid.fasta:887 OB3b\_Genbank\_for\_inparanoid.fasta:887

```
CQW49_RS02770       	100.00%		CQW49_02760         	100.00%
Bootstrap support for CQW49_RS02770 as seed ortholog is 100%.
Bootstrap support for CQW49_02760 as seed ortholog is 100%.
```

---

### Group of orthologs #781. Best score 887 bits Score difference with first non-orthologous sequence - OB3b\_Refseq\_for\_inparanoid.fasta:887 OB3b\_Genbank\_for\_inparanoid.fasta:887

```
CQW49_RS10085       	100.00%		CQW49_10060         	100.00%
Bootstrap support for CQW49_RS10085 as seed ortholog is 100%.
Bootstrap support for CQW49_10060 as seed ortholog is 100%.
```

---

### Group of orthologs #782. Best score 887 bits Score difference with first non-orthologous sequence - OB3b\_Refseq\_for\_inparanoid.fasta:887 OB3b\_Genbank\_for\_inparanoid.fasta:887

```
CQW49_RS14530       	100.00%		CQW49_14495         	100.00%
Bootstrap support for CQW49_RS14530 as seed ortholog is 100%.
Bootstrap support for CQW49_14495 as seed ortholog is 100%.
```

---

### Group of orthologs #783. Best score 886 bits Score difference with first non-orthologous sequence - OB3b\_Refseq\_for\_inparanoid.fasta:886 OB3b\_Genbank\_for\_inparanoid.fasta:886

```
CQW49_RS02405       	100.00%		CQW49_02395         	100.00%
Bootstrap support for CQW49_RS02405 as seed ortholog is 100%.
Bootstrap support for CQW49_02395 as seed ortholog is 100%.
```

---

### Group of orthologs #784. Best score 886 bits Score difference with first non-orthologous sequence - OB3b\_Refseq\_for\_inparanoid.fasta:886 OB3b\_Genbank\_for\_inparanoid.fasta:831

```
CQW49_RS03970       	100.00%		CQW49_03960         	100.00%
Bootstrap support for CQW49_RS03970 as seed ortholog is 100%.
Bootstrap support for CQW49_03960 as seed ortholog is 100%.
```

---

### Group of orthologs #785. Best score 886 bits Score difference with first non-orthologous sequence - OB3b\_Refseq\_for\_inparanoid.fasta:886 OB3b\_Genbank\_for\_inparanoid.fasta:886

```
CQW49_RS15140       	100.00%		CQW49_15105         	100.00%
Bootstrap support for CQW49_RS15140 as seed ortholog is 100%.
Bootstrap support for CQW49_15105 as seed ortholog is 100%.
```

---

### Group of orthologs #786. Best score 886 bits Score difference with first non-orthologous sequence - OB3b\_Refseq\_for\_inparanoid.fasta:886 OB3b\_Genbank\_for\_inparanoid.fasta:789

```
CQW49_RS16845       	100.00%		CQW49_16805         	100.00%
Bootstrap support for CQW49_RS16845 as seed ortholog is 100%.
Bootstrap support for CQW49_16805 as seed ortholog is 100%.
```

---

### Group of orthologs #787. Best score 886 bits Score difference with first non-orthologous sequence - OB3b\_Refseq\_for\_inparanoid.fasta:886 OB3b\_Genbank\_for\_inparanoid.fasta:886

```
CQW49_RS18115       	100.00%		CQW49_18055         	100.00%
Bootstrap support for CQW49_RS18115 as seed ortholog is 100%.
Bootstrap support for CQW49_18055 as seed ortholog is 100%.
```

---

### Group of orthologs #788. Best score 886 bits Score difference with first non-orthologous sequence - OB3b\_Refseq\_for\_inparanoid.fasta:886 OB3b\_Genbank\_for\_inparanoid.fasta:886

```
CQW49_RS20890       	100.00%		CQW49_20815         	100.00%
Bootstrap support for CQW49_RS20890 as seed ortholog is 100%.
Bootstrap support for CQW49_20815 as seed ortholog is 100%.
```

---

### Group of orthologs #789. Best score 885 bits Score difference with first non-orthologous sequence - OB3b\_Refseq\_for\_inparanoid.fasta:885 OB3b\_Genbank\_for\_inparanoid.fasta:885

```
gor                 	100.00%		CQW49_14250         	100.00%
Bootstrap support for gor as seed ortholog is 100%.
Bootstrap support for CQW49_14250 as seed ortholog is 100%.
```

---

### Group of orthologs #790. Best score 885 bits Score difference with first non-orthologous sequence - OB3b\_Refseq\_for\_inparanoid.fasta:885 OB3b\_Genbank\_for\_inparanoid.fasta:885

```
CQW49_RS17495       	100.00%		CQW49_17440         	100.00%
Bootstrap support for CQW49_RS17495 as seed ortholog is 100%.
Bootstrap support for CQW49_17440 as seed ortholog is 100%.
```

---

### Group of orthologs #791. Best score 885 bits Score difference with first non-orthologous sequence - OB3b\_Refseq\_for\_inparanoid.fasta:885 OB3b\_Genbank\_for\_inparanoid.fasta:885

```
fliI                	100.00%		CQW49_18710         	100.00%
Bootstrap support for fliI as seed ortholog is 100%.
Bootstrap support for CQW49_18710 as seed ortholog is 100%.
```

---

### Group of orthologs #792. Best score 884 bits Score difference with first non-orthologous sequence - OB3b\_Refseq\_for\_inparanoid.fasta:884 OB3b\_Genbank\_for\_inparanoid.fasta:884

```
hemN                	100.00%		CQW49_06290         	100.00%
Bootstrap support for hemN as seed ortholog is 100%.
Bootstrap support for CQW49_06290 as seed ortholog is 100%.
```

---

### Group of orthologs #793. Best score 884 bits Score difference with first non-orthologous sequence - OB3b\_Refseq\_for\_inparanoid.fasta:884 OB3b\_Genbank\_for\_inparanoid.fasta:884

```
CQW49_RS11380       	100.00%		CQW49_11350         	100.00%
Bootstrap support for CQW49_RS11380 as seed ortholog is 100%.
Bootstrap support for CQW49_11350 as seed ortholog is 100%.
```

---

### Group of orthologs #794. Best score 883 bits Score difference with first non-orthologous sequence - OB3b\_Refseq\_for\_inparanoid.fasta:883 OB3b\_Genbank\_for\_inparanoid.fasta:883

```
CQW49_RS20770       	100.00%		CQW49_20695         	100.00%
CQW49_RS00210       	100.00%		CQW49_21465         	100.00%
                    	       		CQW49_00210         	100.00%
Bootstrap support for CQW49_RS20770 as seed ortholog is 100%.
Bootstrap support for CQW49_RS00210 as seed ortholog is 100%.
Bootstrap support for CQW49_20695 as seed ortholog is 100%.
Bootstrap support for CQW49_21465 as seed ortholog is 100%.
Bootstrap support for CQW49_00210 as seed ortholog is 100%.
```

---

### Group of orthologs #795. Best score 883 bits Score difference with first non-orthologous sequence - OB3b\_Refseq\_for\_inparanoid.fasta:883 OB3b\_Genbank\_for\_inparanoid.fasta:883

```
CQW49_RS06730       	100.00%		CQW49_06720         	100.00%
Bootstrap support for CQW49_RS06730 as seed ortholog is 100%.
Bootstrap support for CQW49_06720 as seed ortholog is 100%.
```

---

### Group of orthologs #796. Best score 883 bits Score difference with first non-orthologous sequence - OB3b\_Refseq\_for\_inparanoid.fasta:883 OB3b\_Genbank\_for\_inparanoid.fasta:883

```
CQW49_RS08395       	100.00%		CQW49_08385         	100.00%
Bootstrap support for CQW49_RS08395 as seed ortholog is 100%.
Bootstrap support for CQW49_08385 as seed ortholog is 100%.
```

---

### Group of orthologs #797. Best score 883 bits Score difference with first non-orthologous sequence - OB3b\_Refseq\_for\_inparanoid.fasta:883 OB3b\_Genbank\_for\_inparanoid.fasta:883

```
CQW49_RS12070       	100.00%		CQW49_12035         	100.00%
Bootstrap support for CQW49_RS12070 as seed ortholog is 100%.
Bootstrap support for CQW49_12035 as seed ortholog is 100%.
```

---

### Group of orthologs #798. Best score 883 bits Score difference with first non-orthologous sequence - OB3b\_Refseq\_for\_inparanoid.fasta:883 OB3b\_Genbank\_for\_inparanoid.fasta:883

```
CQW49_RS13645       	100.00%		CQW49_13610         	100.00%
Bootstrap support for CQW49_RS13645 as seed ortholog is 100%.
Bootstrap support for CQW49_13610 as seed ortholog is 100%.
```

---

### Group of orthologs #799. Best score 883 bits Score difference with first non-orthologous sequence - OB3b\_Refseq\_for\_inparanoid.fasta:883 OB3b\_Genbank\_for\_inparanoid.fasta:883

```
CQW49_RS15865       	100.00%		CQW49_15830         	100.00%
Bootstrap support for CQW49_RS15865 as seed ortholog is 100%.
Bootstrap support for CQW49_15830 as seed ortholog is 100%.
```

---

### Group of orthologs #800. Best score 880 bits Score difference with first non-orthologous sequence - OB3b\_Refseq\_for\_inparanoid.fasta:880 OB3b\_Genbank\_for\_inparanoid.fasta:880

```
CQW49_RS14905       	100.00%		CQW49_14870         	100.00%
Bootstrap support for CQW49_RS14905 as seed ortholog is 100%.
Bootstrap support for CQW49_14870 as seed ortholog is 100%.
```

---

### Group of orthologs #801. Best score 879 bits Score difference with first non-orthologous sequence - OB3b\_Refseq\_for\_inparanoid.fasta:879 OB3b\_Genbank\_for\_inparanoid.fasta:830

```
CQW49_RS19720       	100.00%		CQW49_19665         	100.00%
Bootstrap support for CQW49_RS19720 as seed ortholog is 100%.
Bootstrap support for CQW49_19665 as seed ortholog is 100%.
```

---

### Group of orthologs #802. Best score 878 bits Score difference with first non-orthologous sequence - OB3b\_Refseq\_for\_inparanoid.fasta:878 OB3b\_Genbank\_for\_inparanoid.fasta:797

```
CQW49_RS03605       	100.00%		CQW49_03595         	100.00%
Bootstrap support for CQW49_RS03605 as seed ortholog is 100%.
Bootstrap support for CQW49_03595 as seed ortholog is 100%.
```

---

### Group of orthologs #803. Best score 878 bits Score difference with first non-orthologous sequence - OB3b\_Refseq\_for\_inparanoid.fasta:878 OB3b\_Genbank\_for\_inparanoid.fasta:878

```
CQW49_RS06215       	100.00%		CQW49_06205         	100.00%
Bootstrap support for CQW49_RS06215 as seed ortholog is 100%.
Bootstrap support for CQW49_06205 as seed ortholog is 100%.
```

---

### Group of orthologs #804. Best score 878 bits Score difference with first non-orthologous sequence - OB3b\_Refseq\_for\_inparanoid.fasta:878 OB3b\_Genbank\_for\_inparanoid.fasta:878

```
CQW49_RS10410       	100.00%		CQW49_10385         	100.00%
Bootstrap support for CQW49_RS10410 as seed ortholog is 100%.
Bootstrap support for CQW49_10385 as seed ortholog is 100%.
```

---

### Group of orthologs #805. Best score 878 bits Score difference with first non-orthologous sequence - OB3b\_Refseq\_for\_inparanoid.fasta:878 OB3b\_Genbank\_for\_inparanoid.fasta:878

```
CQW49_RS16305       	100.00%		CQW49_16270         	100.00%
Bootstrap support for CQW49_RS16305 as seed ortholog is 100%.
Bootstrap support for CQW49_16270 as seed ortholog is 100%.
```

---

### Group of orthologs #806. Best score 878 bits Score difference with first non-orthologous sequence - OB3b\_Refseq\_for\_inparanoid.fasta:878 OB3b\_Genbank\_for\_inparanoid.fasta:457

```
CQW49_RS18950       	100.00%		CQW49_18890         	100.00%
Bootstrap support for CQW49_RS18950 as seed ortholog is 100%.
Bootstrap support for CQW49_18890 as seed ortholog is 100%.
```

---

### Group of orthologs #807. Best score 877 bits Score difference with first non-orthologous sequence - OB3b\_Refseq\_for\_inparanoid.fasta:877 OB3b\_Genbank\_for\_inparanoid.fasta:787

```
ccrA                	100.00%		CQW49_03790         	100.00%
Bootstrap support for ccrA as seed ortholog is 100%.
Bootstrap support for CQW49_03790 as seed ortholog is 100%.
```

---

### Group of orthologs #808. Best score 877 bits Score difference with first non-orthologous sequence - OB3b\_Refseq\_for\_inparanoid.fasta:877 OB3b\_Genbank\_for\_inparanoid.fasta:877

```
CQW49_RS07295       	100.00%		CQW49_07285         	100.00%
Bootstrap support for CQW49_RS07295 as seed ortholog is 100%.
Bootstrap support for CQW49_07285 as seed ortholog is 100%.
```

---

### Group of orthologs #809. Best score 877 bits Score difference with first non-orthologous sequence - OB3b\_Refseq\_for\_inparanoid.fasta:877 OB3b\_Genbank\_for\_inparanoid.fasta:877

```
CQW49_RS15300       	100.00%		CQW49_15265         	100.00%
Bootstrap support for CQW49_RS15300 as seed ortholog is 100%.
Bootstrap support for CQW49_15265 as seed ortholog is 100%.
```

---

### Group of orthologs #810. Best score 876 bits Score difference with first non-orthologous sequence - OB3b\_Refseq\_for\_inparanoid.fasta:876 OB3b\_Genbank\_for\_inparanoid.fasta:876

```
CQW49_RS02200       	100.00%		CQW49_02195         	100.00%
Bootstrap support for CQW49_RS02200 as seed ortholog is 100%.
Bootstrap support for CQW49_02195 as seed ortholog is 100%.
```

---

### Group of orthologs #811. Best score 876 bits Score difference with first non-orthologous sequence - OB3b\_Refseq\_for\_inparanoid.fasta:876 OB3b\_Genbank\_for\_inparanoid.fasta:876

```
CQW49_RS16310       	100.00%		CQW49_16275         	100.00%
Bootstrap support for CQW49_RS16310 as seed ortholog is 100%.
Bootstrap support for CQW49_16275 as seed ortholog is 100%.
```

---

### Group of orthologs #812. Best score 876 bits Score difference with first non-orthologous sequence - OB3b\_Refseq\_for\_inparanoid.fasta:876 OB3b\_Genbank\_for\_inparanoid.fasta:775

```
CQW49_RS17705       	100.00%		CQW49_17650         	100.00%
Bootstrap support for CQW49_RS17705 as seed ortholog is 100%.
Bootstrap support for CQW49_17650 as seed ortholog is 100%.
```

---

### Group of orthologs #813. Best score 876 bits Score difference with first non-orthologous sequence - OB3b\_Refseq\_for\_inparanoid.fasta:876 OB3b\_Genbank\_for\_inparanoid.fasta:876

```
CQW49_RS18370       	100.00%		CQW49_18310         	100.00%
Bootstrap support for CQW49_RS18370 as seed ortholog is 100%.
Bootstrap support for CQW49_18310 as seed ortholog is 100%.
```

---

### Group of orthologs #814. Best score 875 bits Score difference with first non-orthologous sequence - OB3b\_Refseq\_for\_inparanoid.fasta:875 OB3b\_Genbank\_for\_inparanoid.fasta:875

```
CQW49_RS20465       	100.00%		CQW49_20395         	100.00%
Bootstrap support for CQW49_RS20465 as seed ortholog is 100%.
Bootstrap support for CQW49_20395 as seed ortholog is 100%.
```

---

### Group of orthologs #815. Best score 874 bits Score difference with first non-orthologous sequence - OB3b\_Refseq\_for\_inparanoid.fasta:874 OB3b\_Genbank\_for\_inparanoid.fasta:874

```
CQW49_RS02475       	100.00%		CQW49_02465         	100.00%
Bootstrap support for CQW49_RS02475 as seed ortholog is 100%.
Bootstrap support for CQW49_02465 as seed ortholog is 100%.
```

---

### Group of orthologs #816. Best score 873 bits Score difference with first non-orthologous sequence - OB3b\_Refseq\_for\_inparanoid.fasta:873 OB3b\_Genbank\_for\_inparanoid.fasta:770

```
CQW49_RS01645       	100.00%		CQW49_01640         	100.00%
Bootstrap support for CQW49_RS01645 as seed ortholog is 100%.
Bootstrap support for CQW49_01640 as seed ortholog is 100%.
```

---

### Group of orthologs #817. Best score 873 bits Score difference with first non-orthologous sequence - OB3b\_Refseq\_for\_inparanoid.fasta:873 OB3b\_Genbank\_for\_inparanoid.fasta:873

```
glmU                	100.00%		CQW49_09125         	100.00%
Bootstrap support for glmU as seed ortholog is 100%.
Bootstrap support for CQW49_09125 as seed ortholog is 100%.
```

---

### Group of orthologs #818. Best score 873 bits Score difference with first non-orthologous sequence - OB3b\_Refseq\_for\_inparanoid.fasta:873 OB3b\_Genbank\_for\_inparanoid.fasta:873

```
CQW49_RS10030       	100.00%		CQW49_10005         	100.00%
Bootstrap support for CQW49_RS10030 as seed ortholog is 100%.
Bootstrap support for CQW49_10005 as seed ortholog is 100%.
```

---

### Group of orthologs #819. Best score 873 bits Score difference with first non-orthologous sequence - OB3b\_Refseq\_for\_inparanoid.fasta:873 OB3b\_Genbank\_for\_inparanoid.fasta:873

```
CQW49_RS13070       	100.00%		CQW49_13035         	100.00%
Bootstrap support for CQW49_RS13070 as seed ortholog is 100%.
Bootstrap support for CQW49_13035 as seed ortholog is 100%.
```

---

### Group of orthologs #820. Best score 873 bits Score difference with first non-orthologous sequence - OB3b\_Refseq\_for\_inparanoid.fasta:873 OB3b\_Genbank\_for\_inparanoid.fasta:873

```
CQW49_RS20515       	100.00%		CQW49_20445         	100.00%
Bootstrap support for CQW49_RS20515 as seed ortholog is 100%.
Bootstrap support for CQW49_20445 as seed ortholog is 100%.
```

---

### Group of orthologs #821. Best score 872 bits Score difference with first non-orthologous sequence - OB3b\_Refseq\_for\_inparanoid.fasta:872 OB3b\_Genbank\_for\_inparanoid.fasta:872

```
tolB                	100.00%		CQW49_00705         	100.00%
Bootstrap support for tolB as seed ortholog is 100%.
Bootstrap support for CQW49_00705 as seed ortholog is 100%.
```

---

### Group of orthologs #822. Best score 872 bits Score difference with first non-orthologous sequence - OB3b\_Refseq\_for\_inparanoid.fasta:872 OB3b\_Genbank\_for\_inparanoid.fasta:872

```
aroA                	100.00%		CQW49_09925         	100.00%
Bootstrap support for aroA as seed ortholog is 100%.
Bootstrap support for CQW49_09925 as seed ortholog is 100%.
```

---

### Group of orthologs #823. Best score 871 bits Score difference with first non-orthologous sequence - OB3b\_Refseq\_for\_inparanoid.fasta:871 OB3b\_Genbank\_for\_inparanoid.fasta:871

```
CQW49_RS02715       	100.00%		CQW49_02705         	100.00%
Bootstrap support for CQW49_RS02715 as seed ortholog is 100%.
Bootstrap support for CQW49_02705 as seed ortholog is 100%.
```

---

### Group of orthologs #824. Best score 871 bits Score difference with first non-orthologous sequence - OB3b\_Refseq\_for\_inparanoid.fasta:871 OB3b\_Genbank\_for\_inparanoid.fasta:871

```
CQW49_RS08100       	100.00%		CQW49_08090         	100.00%
Bootstrap support for CQW49_RS08100 as seed ortholog is 100%.
Bootstrap support for CQW49_08090 as seed ortholog is 100%.
```

---

### Group of orthologs #825. Best score 870 bits Score difference with first non-orthologous sequence - OB3b\_Refseq\_for\_inparanoid.fasta:870 OB3b\_Genbank\_for\_inparanoid.fasta:870

```
CQW49_RS05690       	100.00%		CQW49_05680         	100.00%
Bootstrap support for CQW49_RS05690 as seed ortholog is 100%.
Bootstrap support for CQW49_05680 as seed ortholog is 100%.
```

---

### Group of orthologs #826. Best score 870 bits Score difference with first non-orthologous sequence - OB3b\_Refseq\_for\_inparanoid.fasta:870 OB3b\_Genbank\_for\_inparanoid.fasta:870

```
CQW49_RS16650       	100.00%		CQW49_16610         	100.00%
Bootstrap support for CQW49_RS16650 as seed ortholog is 100%.
Bootstrap support for CQW49_16610 as seed ortholog is 100%.
```

---

### Group of orthologs #827. Best score 870 bits Score difference with first non-orthologous sequence - OB3b\_Refseq\_for\_inparanoid.fasta:870 OB3b\_Genbank\_for\_inparanoid.fasta:783

```
CQW49_RS16920       	100.00%		CQW49_16875         	100.00%
Bootstrap support for CQW49_RS16920 as seed ortholog is 100%.
Bootstrap support for CQW49_16875 as seed ortholog is 100%.
```

---

### Group of orthologs #828. Best score 869 bits Score difference with first non-orthologous sequence - OB3b\_Refseq\_for\_inparanoid.fasta:869 OB3b\_Genbank\_for\_inparanoid.fasta:869

```
CQW49_RS04715       	100.00%		CQW49_04705         	100.00%
Bootstrap support for CQW49_RS04715 as seed ortholog is 100%.
Bootstrap support for CQW49_04705 as seed ortholog is 100%.
```

---

### Group of orthologs #829. Best score 869 bits Score difference with first non-orthologous sequence - OB3b\_Refseq\_for\_inparanoid.fasta:869 OB3b\_Genbank\_for\_inparanoid.fasta:869

```
gltA                	100.00%		CQW49_04750         	100.00%
Bootstrap support for gltA as seed ortholog is 100%.
Bootstrap support for CQW49_04750 as seed ortholog is 100%.
```

---

### Group of orthologs #830. Best score 869 bits Score difference with first non-orthologous sequence - OB3b\_Refseq\_for\_inparanoid.fasta:869 OB3b\_Genbank\_for\_inparanoid.fasta:869

```
CQW49_RS08650       	100.00%		CQW49_08640         	100.00%
Bootstrap support for CQW49_RS08650 as seed ortholog is 100%.
Bootstrap support for CQW49_08640 as seed ortholog is 100%.
```

---

### Group of orthologs #831. Best score 869 bits Score difference with first non-orthologous sequence - OB3b\_Refseq\_for\_inparanoid.fasta:869 OB3b\_Genbank\_for\_inparanoid.fasta:869

```
CQW49_RS10585       	100.00%		CQW49_10555         	100.00%
Bootstrap support for CQW49_RS10585 as seed ortholog is 100%.
Bootstrap support for CQW49_10555 as seed ortholog is 100%.
```

---

### Group of orthologs #832. Best score 869 bits Score difference with first non-orthologous sequence - OB3b\_Refseq\_for\_inparanoid.fasta:869 OB3b\_Genbank\_for\_inparanoid.fasta:869

```
CQW49_RS14405       	100.00%		CQW49_14370         	100.00%
Bootstrap support for CQW49_RS14405 as seed ortholog is 100%.
Bootstrap support for CQW49_14370 as seed ortholog is 100%.
```

---

### Group of orthologs #833. Best score 869 bits Score difference with first non-orthologous sequence - OB3b\_Refseq\_for\_inparanoid.fasta:869 OB3b\_Genbank\_for\_inparanoid.fasta:869

```
CQW49_RS20005       	100.00%		CQW49_19940         	100.00%
Bootstrap support for CQW49_RS20005 as seed ortholog is 100%.
Bootstrap support for CQW49_19940 as seed ortholog is 100%.
```

---

### Group of orthologs #834. Best score 868 bits Score difference with first non-orthologous sequence - OB3b\_Refseq\_for\_inparanoid.fasta:868 OB3b\_Genbank\_for\_inparanoid.fasta:868

```
CQW49_RS02220       	100.00%		CQW49_02215         	100.00%
Bootstrap support for CQW49_RS02220 as seed ortholog is 100%.
Bootstrap support for CQW49_02215 as seed ortholog is 100%.
```

---

### Group of orthologs #835. Best score 868 bits Score difference with first non-orthologous sequence - OB3b\_Refseq\_for\_inparanoid.fasta:868 OB3b\_Genbank\_for\_inparanoid.fasta:868

```
CQW49_RS06895       	100.00%		CQW49_06885         	100.00%
Bootstrap support for CQW49_RS06895 as seed ortholog is 100%.
Bootstrap support for CQW49_06885 as seed ortholog is 100%.
```

---

### Group of orthologs #836. Best score 868 bits Score difference with first non-orthologous sequence - OB3b\_Refseq\_for\_inparanoid.fasta:868 OB3b\_Genbank\_for\_inparanoid.fasta:868

```
CQW49_RS08245       	100.00%		CQW49_08235         	100.00%
Bootstrap support for CQW49_RS08245 as seed ortholog is 100%.
Bootstrap support for CQW49_08235 as seed ortholog is 100%.
```

---

### Group of orthologs #837. Best score 867 bits Score difference with first non-orthologous sequence - OB3b\_Refseq\_for\_inparanoid.fasta:867 OB3b\_Genbank\_for\_inparanoid.fasta:867

```
CQW49_RS04385       	100.00%		CQW49_04380         	100.00%
Bootstrap support for CQW49_RS04385 as seed ortholog is 100%.
Bootstrap support for CQW49_04380 as seed ortholog is 100%.
```

---

### Group of orthologs #838. Best score 867 bits Score difference with first non-orthologous sequence - OB3b\_Refseq\_for\_inparanoid.fasta:867 OB3b\_Genbank\_for\_inparanoid.fasta:867

```
CQW49_RS06505       	100.00%		CQW49_06495         	100.00%
Bootstrap support for CQW49_RS06505 as seed ortholog is 100%.
Bootstrap support for CQW49_06495 as seed ortholog is 100%.
```

---

### Group of orthologs #839. Best score 867 bits Score difference with first non-orthologous sequence - OB3b\_Refseq\_for\_inparanoid.fasta:867 OB3b\_Genbank\_for\_inparanoid.fasta:867

```
CQW49_RS07160       	100.00%		CQW49_07150         	100.00%
Bootstrap support for CQW49_RS07160 as seed ortholog is 100%.
Bootstrap support for CQW49_07150 as seed ortholog is 100%.
```

---

### Group of orthologs #840. Best score 867 bits Score difference with first non-orthologous sequence - OB3b\_Refseq\_for\_inparanoid.fasta:867 OB3b\_Genbank\_for\_inparanoid.fasta:867

```
CQW49_RS09380       	100.00%		CQW49_09360         	100.00%
Bootstrap support for CQW49_RS09380 as seed ortholog is 100%.
Bootstrap support for CQW49_09360 as seed ortholog is 100%.
```

---

### Group of orthologs #841. Best score 867 bits Score difference with first non-orthologous sequence - OB3b\_Refseq\_for\_inparanoid.fasta:867 OB3b\_Genbank\_for\_inparanoid.fasta:867

```
CQW49_RS10145       	100.00%		CQW49_10120         	100.00%
Bootstrap support for CQW49_RS10145 as seed ortholog is 100%.
Bootstrap support for CQW49_10120 as seed ortholog is 100%.
```

---

### Group of orthologs #842. Best score 867 bits Score difference with first non-orthologous sequence - OB3b\_Refseq\_for\_inparanoid.fasta:867 OB3b\_Genbank\_for\_inparanoid.fasta:867

```
CQW49_RS21045       	100.00%		CQW49_20970         	100.00%
Bootstrap support for CQW49_RS21045 as seed ortholog is 100%.
Bootstrap support for CQW49_20970 as seed ortholog is 100%.
```

---

### Group of orthologs #843. Best score 866 bits Score difference with first non-orthologous sequence - OB3b\_Refseq\_for\_inparanoid.fasta:866 OB3b\_Genbank\_for\_inparanoid.fasta:866

```
CQW49_RS10420       	100.00%		CQW49_10395         	100.00%
Bootstrap support for CQW49_RS10420 as seed ortholog is 100%.
Bootstrap support for CQW49_10395 as seed ortholog is 100%.
```

---

### Group of orthologs #844. Best score 866 bits Score difference with first non-orthologous sequence - OB3b\_Refseq\_for\_inparanoid.fasta:866 OB3b\_Genbank\_for\_inparanoid.fasta:866

```
CQW49_RS13390       	100.00%		CQW49_13355         	100.00%
Bootstrap support for CQW49_RS13390 as seed ortholog is 100%.
Bootstrap support for CQW49_13355 as seed ortholog is 100%.
```

---

### Group of orthologs #845. Best score 866 bits Score difference with first non-orthologous sequence - OB3b\_Refseq\_for\_inparanoid.fasta:866 OB3b\_Genbank\_for\_inparanoid.fasta:866

```
CQW49_RS16725       	100.00%		CQW49_16685         	100.00%
Bootstrap support for CQW49_RS16725 as seed ortholog is 100%.
Bootstrap support for CQW49_16685 as seed ortholog is 100%.
```

---

### Group of orthologs #846. Best score 866 bits Score difference with first non-orthologous sequence - OB3b\_Refseq\_for\_inparanoid.fasta:866 OB3b\_Genbank\_for\_inparanoid.fasta:866

```
CQW49_RS19635       	100.00%		CQW49_19580         	100.00%
Bootstrap support for CQW49_RS19635 as seed ortholog is 100%.
Bootstrap support for CQW49_19580 as seed ortholog is 100%.
```

---

### Group of orthologs #847. Best score 864 bits Score difference with first non-orthologous sequence - OB3b\_Refseq\_for\_inparanoid.fasta:864 OB3b\_Genbank\_for\_inparanoid.fasta:864

```
CQW49_RS00150       	100.00%		CQW49_00150         	100.00%
Bootstrap support for CQW49_RS00150 as seed ortholog is 100%.
Bootstrap support for CQW49_00150 as seed ortholog is 100%.
```

---

### Group of orthologs #848. Best score 864 bits Score difference with first non-orthologous sequence - OB3b\_Refseq\_for\_inparanoid.fasta:864 OB3b\_Genbank\_for\_inparanoid.fasta:526

```
CQW49_RS08335       	100.00%		CQW49_08325         	100.00%
Bootstrap support for CQW49_RS08335 as seed ortholog is 100%.
Bootstrap support for CQW49_08325 as seed ortholog is 100%.
```

---

### Group of orthologs #849. Best score 864 bits Score difference with first non-orthologous sequence - OB3b\_Refseq\_for\_inparanoid.fasta:864 OB3b\_Genbank\_for\_inparanoid.fasta:864

```
CQW49_RS14575       	100.00%		CQW49_14540         	100.00%
Bootstrap support for CQW49_RS14575 as seed ortholog is 100%.
Bootstrap support for CQW49_14540 as seed ortholog is 100%.
```

---

### Group of orthologs #850. Best score 864 bits Score difference with first non-orthologous sequence - OB3b\_Refseq\_for\_inparanoid.fasta:864 OB3b\_Genbank\_for\_inparanoid.fasta:864

```
CQW49_RS15645       	100.00%		CQW49_15610         	100.00%
Bootstrap support for CQW49_RS15645 as seed ortholog is 100%.
Bootstrap support for CQW49_15610 as seed ortholog is 100%.
```

---

### Group of orthologs #851. Best score 864 bits Score difference with first non-orthologous sequence - OB3b\_Refseq\_for\_inparanoid.fasta:864 OB3b\_Genbank\_for\_inparanoid.fasta:864

```
CQW49_RS17540       	100.00%		CQW49_17485         	100.00%
Bootstrap support for CQW49_RS17540 as seed ortholog is 100%.
Bootstrap support for CQW49_17485 as seed ortholog is 100%.
```

---

### Group of orthologs #852. Best score 863 bits Score difference with first non-orthologous sequence - OB3b\_Refseq\_for\_inparanoid.fasta:863 OB3b\_Genbank\_for\_inparanoid.fasta:863

```
CQW49_RS04355       	100.00%		CQW49_04350         	100.00%
Bootstrap support for CQW49_RS04355 as seed ortholog is 100%.
Bootstrap support for CQW49_04350 as seed ortholog is 100%.
```

---

### Group of orthologs #853. Best score 863 bits Score difference with first non-orthologous sequence - OB3b\_Refseq\_for\_inparanoid.fasta:863 OB3b\_Genbank\_for\_inparanoid.fasta:863

```
CQW49_RS20400       	100.00%		CQW49_20330         	100.00%
Bootstrap support for CQW49_RS20400 as seed ortholog is 100%.
Bootstrap support for CQW49_20330 as seed ortholog is 100%.
```

---

### Group of orthologs #854. Best score 861 bits Score difference with first non-orthologous sequence - OB3b\_Refseq\_for\_inparanoid.fasta:861 OB3b\_Genbank\_for\_inparanoid.fasta:799

```
CQW49_RS00785       	100.00%		CQW49_00785         	100.00%
Bootstrap support for CQW49_RS00785 as seed ortholog is 100%.
Bootstrap support for CQW49_00785 as seed ortholog is 100%.
```

---

### Group of orthologs #855. Best score 861 bits Score difference with first non-orthologous sequence - OB3b\_Refseq\_for\_inparanoid.fasta:861 OB3b\_Genbank\_for\_inparanoid.fasta:861

```
CQW49_RS09645       	100.00%		CQW49_09625         	100.00%
Bootstrap support for CQW49_RS09645 as seed ortholog is 100%.
Bootstrap support for CQW49_09625 as seed ortholog is 100%.
```

---

### Group of orthologs #856. Best score 861 bits Score difference with first non-orthologous sequence - OB3b\_Refseq\_for\_inparanoid.fasta:861 OB3b\_Genbank\_for\_inparanoid.fasta:861

```
CQW49_RS15310       	100.00%		CQW49_15275         	100.00%
Bootstrap support for CQW49_RS15310 as seed ortholog is 100%.
Bootstrap support for CQW49_15275 as seed ortholog is 100%.
```

---

### Group of orthologs #857. Best score 861 bits Score difference with first non-orthologous sequence - OB3b\_Refseq\_for\_inparanoid.fasta:861 OB3b\_Genbank\_for\_inparanoid.fasta:861

```
CQW49_RS18905       	100.00%		CQW49_18845         	100.00%
Bootstrap support for CQW49_RS18905 as seed ortholog is 100%.
Bootstrap support for CQW49_18845 as seed ortholog is 100%.
```

---

### Group of orthologs #858. Best score 860 bits Score difference with first non-orthologous sequence - OB3b\_Refseq\_for\_inparanoid.fasta:860 OB3b\_Genbank\_for\_inparanoid.fasta:860

```
CQW49_RS14535       	100.00%		CQW49_14500         	100.00%
Bootstrap support for CQW49_RS14535 as seed ortholog is 100%.
Bootstrap support for CQW49_14500 as seed ortholog is 100%.
```

---

### Group of orthologs #859. Best score 859 bits Score difference with first non-orthologous sequence - OB3b\_Refseq\_for\_inparanoid.fasta:859 OB3b\_Genbank\_for\_inparanoid.fasta:802

```
CQW49_RS08540       	100.00%		CQW49_08530         	100.00%
Bootstrap support for CQW49_RS08540 as seed ortholog is 100%.
Bootstrap support for CQW49_08530 as seed ortholog is 100%.
```

---

### Group of orthologs #860. Best score 859 bits Score difference with first non-orthologous sequence - OB3b\_Refseq\_for\_inparanoid.fasta:859 OB3b\_Genbank\_for\_inparanoid.fasta:809

```
CQW49_RS09160       	100.00%		CQW49_09140         	100.00%
Bootstrap support for CQW49_RS09160 as seed ortholog is 100%.
Bootstrap support for CQW49_09140 as seed ortholog is 100%.
```

---

### Group of orthologs #861. Best score 858 bits Score difference with first non-orthologous sequence - OB3b\_Refseq\_for\_inparanoid.fasta:858 OB3b\_Genbank\_for\_inparanoid.fasta:858

```
CQW49_RS06980       	100.00%		CQW49_06970         	100.00%
Bootstrap support for CQW49_RS06980 as seed ortholog is 100%.
Bootstrap support for CQW49_06970 as seed ortholog is 100%.
```

---

### Group of orthologs #862. Best score 858 bits Score difference with first non-orthologous sequence - OB3b\_Refseq\_for\_inparanoid.fasta:858 OB3b\_Genbank\_for\_inparanoid.fasta:858

```
CQW49_RS20040       	100.00%		CQW49_19975         	100.00%
Bootstrap support for CQW49_RS20040 as seed ortholog is 100%.
Bootstrap support for CQW49_19975 as seed ortholog is 100%.
```

---

### Group of orthologs #863. Best score 857 bits Score difference with first non-orthologous sequence - OB3b\_Refseq\_for\_inparanoid.fasta:857 OB3b\_Genbank\_for\_inparanoid.fasta:857

```
CQW49_RS16190       	100.00%		CQW49_16155         	100.00%
Bootstrap support for CQW49_RS16190 as seed ortholog is 100%.
Bootstrap support for CQW49_16155 as seed ortholog is 100%.
```

---

### Group of orthologs #864. Best score 857 bits Score difference with first non-orthologous sequence - OB3b\_Refseq\_for\_inparanoid.fasta:857 OB3b\_Genbank\_for\_inparanoid.fasta:857

```
hemA                	100.00%		CQW49_19185         	100.00%
Bootstrap support for hemA as seed ortholog is 100%.
Bootstrap support for CQW49_19185 as seed ortholog is 100%.
```

---

### Group of orthologs #865. Best score 856 bits Score difference with first non-orthologous sequence - OB3b\_Refseq\_for\_inparanoid.fasta:856 OB3b\_Genbank\_for\_inparanoid.fasta:856

```
CQW49_RS00055       	100.00%		CQW49_00055         	100.00%
Bootstrap support for CQW49_RS00055 as seed ortholog is 100%.
Bootstrap support for CQW49_00055 as seed ortholog is 100%.
```

---

### Group of orthologs #866. Best score 856 bits Score difference with first non-orthologous sequence - OB3b\_Refseq\_for\_inparanoid.fasta:856 OB3b\_Genbank\_for\_inparanoid.fasta:856

```
CQW49_RS03815       	100.00%		CQW49_03805         	100.00%
Bootstrap support for CQW49_RS03815 as seed ortholog is 100%.
Bootstrap support for CQW49_03805 as seed ortholog is 100%.
```

---

### Group of orthologs #867. Best score 856 bits Score difference with first non-orthologous sequence - OB3b\_Refseq\_for\_inparanoid.fasta:856 OB3b\_Genbank\_for\_inparanoid.fasta:856

```
CQW49_RS13930       	100.00%		CQW49_13895         	100.00%
Bootstrap support for CQW49_RS13930 as seed ortholog is 100%.
Bootstrap support for CQW49_13895 as seed ortholog is 100%.
```

---

### Group of orthologs #868. Best score 855 bits Score difference with first non-orthologous sequence - OB3b\_Refseq\_for\_inparanoid.fasta:855 OB3b\_Genbank\_for\_inparanoid.fasta:855

```
CQW49_RS10275       	100.00%		CQW49_10250         	100.00%
CQW49_RS01230       	100.00%		CQW49_01225         	100.00%
Bootstrap support for CQW49_RS10275 as seed ortholog is 100%.
Bootstrap support for CQW49_RS01230 as seed ortholog is 100%.
Bootstrap support for CQW49_10250 as seed ortholog is 100%.
Bootstrap support for CQW49_01225 as seed ortholog is 100%.
```

---

### Group of orthologs #869. Best score 855 bits Score difference with first non-orthologous sequence - OB3b\_Refseq\_for\_inparanoid.fasta:855 OB3b\_Genbank\_for\_inparanoid.fasta:855

```
CQW49_RS15465       	100.00%		CQW49_15430         	100.00%
Bootstrap support for CQW49_RS15465 as seed ortholog is 100%.
Bootstrap support for CQW49_15430 as seed ortholog is 100%.
```

---

### Group of orthologs #870. Best score 855 bits Score difference with first non-orthologous sequence - OB3b\_Refseq\_for\_inparanoid.fasta:855 OB3b\_Genbank\_for\_inparanoid.fasta:855

```
CQW49_RS16715       	100.00%		CQW49_16675         	100.00%
Bootstrap support for CQW49_RS16715 as seed ortholog is 100%.
Bootstrap support for CQW49_16675 as seed ortholog is 100%.
```

---

### Group of orthologs #871. Best score 855 bits Score difference with first non-orthologous sequence - OB3b\_Refseq\_for\_inparanoid.fasta:855 OB3b\_Genbank\_for\_inparanoid.fasta:855

```
CQW49_RS16840       	100.00%		CQW49_16800         	100.00%
Bootstrap support for CQW49_RS16840 as seed ortholog is 100%.
Bootstrap support for CQW49_16800 as seed ortholog is 100%.
```

---

### Group of orthologs #872. Best score 854 bits Score difference with first non-orthologous sequence - OB3b\_Refseq\_for\_inparanoid.fasta:854 OB3b\_Genbank\_for\_inparanoid.fasta:703

```
CQW49_RS14910       	100.00%		CQW49_14875         	100.00%
Bootstrap support for CQW49_RS14910 as seed ortholog is 100%.
Bootstrap support for CQW49_14875 as seed ortholog is 100%.
```

---

### Group of orthologs #873. Best score 854 bits Score difference with first non-orthologous sequence - OB3b\_Refseq\_for\_inparanoid.fasta:854 OB3b\_Genbank\_for\_inparanoid.fasta:854

```
CQW49_RS19045       	100.00%		CQW49_18985         	100.00%
Bootstrap support for CQW49_RS19045 as seed ortholog is 100%.
Bootstrap support for CQW49_18985 as seed ortholog is 100%.
```

---

### Group of orthologs #874. Best score 852 bits Score difference with first non-orthologous sequence - OB3b\_Refseq\_for\_inparanoid.fasta:852 OB3b\_Genbank\_for\_inparanoid.fasta:29

```
trbL                	100.00%		CQW49_14945         	100.00%
trbL                	100.00%		CQW49_14625         	100.00%
trbL                	100.00%		
trbL                	100.00%		
trbL                	100.00%		
trbL                	100.00%		
trbL                	100.00%		
trbL                	100.00%		
trbL                	100.00%		
Bootstrap support for trbL as seed ortholog is 100%.
Bootstrap support for trbL as seed ortholog is 100%.
Bootstrap support for trbL as seed ortholog is 100%.
Bootstrap support for trbL as seed ortholog is 100%.
Bootstrap support for trbL as seed ortholog is 100%.
Bootstrap support for trbL as seed ortholog is 100%.
Bootstrap support for trbL as seed ortholog is 100%.
Bootstrap support for trbL as seed ortholog is 100%.
Bootstrap support for trbL as seed ortholog is 100%.
Bootstrap support for CQW49_14945 as seed ortholog is 97%.
Bootstrap support for CQW49_14625 as seed ortholog is 97%.
```

---

### Group of orthologs #875. Best score 852 bits Score difference with first non-orthologous sequence - OB3b\_Refseq\_for\_inparanoid.fasta:852 OB3b\_Genbank\_for\_inparanoid.fasta:852

```
CQW49_RS07830       	100.00%		CQW49_07820         	100.00%
Bootstrap support for CQW49_RS07830 as seed ortholog is 100%.
Bootstrap support for CQW49_07820 as seed ortholog is 100%.
```

---

### Group of orthologs #876. Best score 852 bits Score difference with first non-orthologous sequence - OB3b\_Refseq\_for\_inparanoid.fasta:852 OB3b\_Genbank\_for\_inparanoid.fasta:595

```
CQW49_RS08375       	100.00%		CQW49_08365         	100.00%
Bootstrap support for CQW49_RS08375 as seed ortholog is 100%.
Bootstrap support for CQW49_08365 as seed ortholog is 100%.
```

---

### Group of orthologs #877. Best score 852 bits Score difference with first non-orthologous sequence - OB3b\_Refseq\_for\_inparanoid.fasta:852 OB3b\_Genbank\_for\_inparanoid.fasta:852

```
CQW49_RS20845       	100.00%		CQW49_20770         	100.00%
Bootstrap support for CQW49_RS20845 as seed ortholog is 100%.
Bootstrap support for CQW49_20770 as seed ortholog is 100%.
```

---

### Group of orthologs #878. Best score 851 bits Score difference with first non-orthologous sequence - OB3b\_Refseq\_for\_inparanoid.fasta:851 OB3b\_Genbank\_for\_inparanoid.fasta:851

```
CQW49_RS12760       	100.00%		CQW49_12725         	100.00%
Bootstrap support for CQW49_RS12760 as seed ortholog is 100%.
Bootstrap support for CQW49_12725 as seed ortholog is 100%.
```

---

### Group of orthologs #879. Best score 850 bits Score difference with first non-orthologous sequence - OB3b\_Refseq\_for\_inparanoid.fasta:850 OB3b\_Genbank\_for\_inparanoid.fasta:783

```
CQW49_RS06260       	100.00%		CQW49_06250         	100.00%
Bootstrap support for CQW49_RS06260 as seed ortholog is 100%.
Bootstrap support for CQW49_06250 as seed ortholog is 100%.
```

---

### Group of orthologs #880. Best score 849 bits Score difference with first non-orthologous sequence - OB3b\_Refseq\_for\_inparanoid.fasta:849 OB3b\_Genbank\_for\_inparanoid.fasta:849

```
ftsA                	100.00%		CQW49_11265         	100.00%
Bootstrap support for ftsA as seed ortholog is 100%.
Bootstrap support for CQW49_11265 as seed ortholog is 100%.
```

---

### Group of orthologs #881. Best score 849 bits Score difference with first non-orthologous sequence - OB3b\_Refseq\_for\_inparanoid.fasta:849 OB3b\_Genbank\_for\_inparanoid.fasta:849

```
CQW49_RS11755       	100.00%		CQW49_11720         	100.00%
Bootstrap support for CQW49_RS11755 as seed ortholog is 100%.
Bootstrap support for CQW49_11720 as seed ortholog is 100%.
```

---

### Group of orthologs #882. Best score 848 bits Score difference with first non-orthologous sequence - OB3b\_Refseq\_for\_inparanoid.fasta:848 OB3b\_Genbank\_for\_inparanoid.fasta:848

```
CQW49_RS06780       	100.00%		CQW49_06770         	100.00%
Bootstrap support for CQW49_RS06780 as seed ortholog is 100%.
Bootstrap support for CQW49_06770 as seed ortholog is 100%.
```

---

### Group of orthologs #883. Best score 848 bits Score difference with first non-orthologous sequence - OB3b\_Refseq\_for\_inparanoid.fasta:848 OB3b\_Genbank\_for\_inparanoid.fasta:848

```
CQW49_RS11010       	100.00%		CQW49_10980         	100.00%
Bootstrap support for CQW49_RS11010 as seed ortholog is 100%.
Bootstrap support for CQW49_10980 as seed ortholog is 100%.
```

---

### Group of orthologs #884. Best score 848 bits Score difference with first non-orthologous sequence - OB3b\_Refseq\_for\_inparanoid.fasta:848 OB3b\_Genbank\_for\_inparanoid.fasta:848

```
CQW49_RS19630       	100.00%		CQW49_19575         	100.00%
Bootstrap support for CQW49_RS19630 as seed ortholog is 100%.
Bootstrap support for CQW49_19575 as seed ortholog is 100%.
```

---

### Group of orthologs #885. Best score 847 bits Score difference with first non-orthologous sequence - OB3b\_Refseq\_for\_inparanoid.fasta:847 OB3b\_Genbank\_for\_inparanoid.fasta:847

```
CQW49_RS03320       	100.00%		CQW49_03310         	100.00%
Bootstrap support for CQW49_RS03320 as seed ortholog is 100%.
Bootstrap support for CQW49_03310 as seed ortholog is 100%.
```

---

### Group of orthologs #886. Best score 847 bits Score difference with first non-orthologous sequence - OB3b\_Refseq\_for\_inparanoid.fasta:847 OB3b\_Genbank\_for\_inparanoid.fasta:847

```
CQW49_RS20085       	100.00%		CQW49_20020         	100.00%
Bootstrap support for CQW49_RS20085 as seed ortholog is 100%.
Bootstrap support for CQW49_20020 as seed ortholog is 100%.
```

---

### Group of orthologs #887. Best score 846 bits Score difference with first non-orthologous sequence - OB3b\_Refseq\_for\_inparanoid.fasta:846 OB3b\_Genbank\_for\_inparanoid.fasta:846

```
CQW49_RS05165       	100.00%		CQW49_05160         	100.00%
Bootstrap support for CQW49_RS05165 as seed ortholog is 100%.
Bootstrap support for CQW49_05160 as seed ortholog is 100%.
```

---

### Group of orthologs #888. Best score 846 bits Score difference with first non-orthologous sequence - OB3b\_Refseq\_for\_inparanoid.fasta:846 OB3b\_Genbank\_for\_inparanoid.fasta:846

```
CQW49_RS11810       	100.00%		CQW49_11775         	100.00%
Bootstrap support for CQW49_RS11810 as seed ortholog is 100%.
Bootstrap support for CQW49_11775 as seed ortholog is 100%.
```

---

### Group of orthologs #889. Best score 845 bits Score difference with first non-orthologous sequence - OB3b\_Refseq\_for\_inparanoid.fasta:845 OB3b\_Genbank\_for\_inparanoid.fasta:845

```
CQW49_RS04545       	100.00%		CQW49_04540         	100.00%
Bootstrap support for CQW49_RS04545 as seed ortholog is 100%.
Bootstrap support for CQW49_04540 as seed ortholog is 100%.
```

---

### Group of orthologs #890. Best score 844 bits Score difference with first non-orthologous sequence - OB3b\_Refseq\_for\_inparanoid.fasta:844 OB3b\_Genbank\_for\_inparanoid.fasta:844

```
glyA                	100.00%		CQW49_06365         	100.00%
Bootstrap support for glyA as seed ortholog is 100%.
Bootstrap support for CQW49_06365 as seed ortholog is 100%.
```

---

### Group of orthologs #891. Best score 843 bits Score difference with first non-orthologous sequence - OB3b\_Refseq\_for\_inparanoid.fasta:843 OB3b\_Genbank\_for\_inparanoid.fasta:843

```
CQW49_RS11085       	100.00%		CQW49_11055         	100.00%
Bootstrap support for CQW49_RS11085 as seed ortholog is 100%.
Bootstrap support for CQW49_11055 as seed ortholog is 100%.
```

---

### Group of orthologs #892. Best score 843 bits Score difference with first non-orthologous sequence - OB3b\_Refseq\_for\_inparanoid.fasta:843 OB3b\_Genbank\_for\_inparanoid.fasta:843

```
CQW49_RS11845       	100.00%		CQW49_11810         	100.00%
Bootstrap support for CQW49_RS11845 as seed ortholog is 100%.
Bootstrap support for CQW49_11810 as seed ortholog is 100%.
```

---

### Group of orthologs #893. Best score 843 bits Score difference with first non-orthologous sequence - OB3b\_Refseq\_for\_inparanoid.fasta:843 OB3b\_Genbank\_for\_inparanoid.fasta:843

```
CQW49_RS15665       	100.00%		CQW49_15630         	100.00%
Bootstrap support for CQW49_RS15665 as seed ortholog is 100%.
Bootstrap support for CQW49_15630 as seed ortholog is 100%.
```

---

### Group of orthologs #894. Best score 843 bits Score difference with first non-orthologous sequence - OB3b\_Refseq\_for\_inparanoid.fasta:843 OB3b\_Genbank\_for\_inparanoid.fasta:843

```
CQW49_RS19250       	100.00%		CQW49_19195         	100.00%
Bootstrap support for CQW49_RS19250 as seed ortholog is 100%.
Bootstrap support for CQW49_19195 as seed ortholog is 100%.
```

---

### Group of orthologs #895. Best score 842 bits Score difference with first non-orthologous sequence - OB3b\_Refseq\_for\_inparanoid.fasta:842 OB3b\_Genbank\_for\_inparanoid.fasta:842

```
CQW49_RS09245       	100.00%		CQW49_09225         	100.00%
Bootstrap support for CQW49_RS09245 as seed ortholog is 100%.
Bootstrap support for CQW49_09225 as seed ortholog is 100%.
```

---

### Group of orthologs #896. Best score 842 bits Score difference with first non-orthologous sequence - OB3b\_Refseq\_for\_inparanoid.fasta:842 OB3b\_Genbank\_for\_inparanoid.fasta:842

```
CQW49_RS13205       	100.00%		CQW49_13170         	100.00%
Bootstrap support for CQW49_RS13205 as seed ortholog is 100%.
Bootstrap support for CQW49_13170 as seed ortholog is 100%.
```

---

### Group of orthologs #897. Best score 841 bits Score difference with first non-orthologous sequence - OB3b\_Refseq\_for\_inparanoid.fasta:841 OB3b\_Genbank\_for\_inparanoid.fasta:841

```
CQW49_RS11785       	100.00%		CQW49_11750         	100.00%
Bootstrap support for CQW49_RS11785 as seed ortholog is 100%.
Bootstrap support for CQW49_11750 as seed ortholog is 100%.
```

---

### Group of orthologs #898. Best score 841 bits Score difference with first non-orthologous sequence - OB3b\_Refseq\_for\_inparanoid.fasta:841 OB3b\_Genbank\_for\_inparanoid.fasta:841

```
trpB                	100.00%		CQW49_17140         	100.00%
Bootstrap support for trpB as seed ortholog is 100%.
Bootstrap support for CQW49_17140 as seed ortholog is 100%.
```

---

### Group of orthologs #899. Best score 841 bits Score difference with first non-orthologous sequence - OB3b\_Refseq\_for\_inparanoid.fasta:841 OB3b\_Genbank\_for\_inparanoid.fasta:841

```
CQW49_RS18085       	100.00%		CQW49_18025         	100.00%
Bootstrap support for CQW49_RS18085 as seed ortholog is 100%.
Bootstrap support for CQW49_18025 as seed ortholog is 100%.
```

---

### Group of orthologs #900. Best score 840 bits Score difference with first non-orthologous sequence - OB3b\_Refseq\_for\_inparanoid.fasta:840 OB3b\_Genbank\_for\_inparanoid.fasta:840

```
CQW49_RS00745       	100.00%		CQW49_00745         	100.00%
Bootstrap support for CQW49_RS00745 as seed ortholog is 100%.
Bootstrap support for CQW49_00745 as seed ortholog is 100%.
```

---

### Group of orthologs #901. Best score 840 bits Score difference with first non-orthologous sequence - OB3b\_Refseq\_for\_inparanoid.fasta:840 OB3b\_Genbank\_for\_inparanoid.fasta:739

```
CQW49_RS01850       	100.00%		CQW49_01845         	100.00%
Bootstrap support for CQW49_RS01850 as seed ortholog is 100%.
Bootstrap support for CQW49_01845 as seed ortholog is 100%.
```

---

### Group of orthologs #902. Best score 839 bits Score difference with first non-orthologous sequence - OB3b\_Refseq\_for\_inparanoid.fasta:839 OB3b\_Genbank\_for\_inparanoid.fasta:839

```
CQW49_RS14780       	100.00%		CQW49_14745         	100.00%
Bootstrap support for CQW49_RS14780 as seed ortholog is 100%.
Bootstrap support for CQW49_14745 as seed ortholog is 100%.
```

---

### Group of orthologs #903. Best score 838 bits Score difference with first non-orthologous sequence - OB3b\_Refseq\_for\_inparanoid.fasta:838 OB3b\_Genbank\_for\_inparanoid.fasta:838

```
CQW49_RS06230       	100.00%		CQW49_06220         	100.00%
Bootstrap support for CQW49_RS06230 as seed ortholog is 100%.
Bootstrap support for CQW49_06220 as seed ortholog is 100%.
```

---

### Group of orthologs #904. Best score 838 bits Score difference with first non-orthologous sequence - OB3b\_Refseq\_for\_inparanoid.fasta:838 OB3b\_Genbank\_for\_inparanoid.fasta:838

```
CQW49_RS13060       	100.00%		CQW49_13025         	100.00%
Bootstrap support for CQW49_RS13060 as seed ortholog is 100%.
Bootstrap support for CQW49_13025 as seed ortholog is 100%.
```

---

### Group of orthologs #905. Best score 838 bits Score difference with first non-orthologous sequence - OB3b\_Refseq\_for\_inparanoid.fasta:838 OB3b\_Genbank\_for\_inparanoid.fasta:838

```
CQW49_RS20530       	100.00%		CQW49_20460         	100.00%
Bootstrap support for CQW49_RS20530 as seed ortholog is 100%.
Bootstrap support for CQW49_20460 as seed ortholog is 100%.
```

---

### Group of orthologs #906. Best score 837 bits Score difference with first non-orthologous sequence - OB3b\_Refseq\_for\_inparanoid.fasta:837 OB3b\_Genbank\_for\_inparanoid.fasta:837

```
ispG                	100.00%		CQW49_12305         	100.00%
Bootstrap support for ispG as seed ortholog is 100%.
Bootstrap support for CQW49_12305 as seed ortholog is 100%.
```

---

### Group of orthologs #907. Best score 837 bits Score difference with first non-orthologous sequence - OB3b\_Refseq\_for\_inparanoid.fasta:837 OB3b\_Genbank\_for\_inparanoid.fasta:837

```
CQW49_RS13225       	100.00%		CQW49_13190         	100.00%
Bootstrap support for CQW49_RS13225 as seed ortholog is 100%.
Bootstrap support for CQW49_13190 as seed ortholog is 100%.
```

---

### Group of orthologs #908. Best score 837 bits Score difference with first non-orthologous sequence - OB3b\_Refseq\_for\_inparanoid.fasta:837 OB3b\_Genbank\_for\_inparanoid.fasta:716

```
fabF                	100.00%		CQW49_17690         	100.00%
Bootstrap support for fabF as seed ortholog is 100%.
Bootstrap support for CQW49_17690 as seed ortholog is 100%.
```

---

### Group of orthologs #909. Best score 836 bits Score difference with first non-orthologous sequence - OB3b\_Refseq\_for\_inparanoid.fasta:836 OB3b\_Genbank\_for\_inparanoid.fasta:555

```
CQW49_RS04905       	100.00%		CQW49_04895         	100.00%
Bootstrap support for CQW49_RS04905 as seed ortholog is 100%.
Bootstrap support for CQW49_04895 as seed ortholog is 100%.
```

---

### Group of orthologs #910. Best score 836 bits Score difference with first non-orthologous sequence - OB3b\_Refseq\_for\_inparanoid.fasta:836 OB3b\_Genbank\_for\_inparanoid.fasta:836

```
CQW49_RS07070       	100.00%		CQW49_07060         	100.00%
Bootstrap support for CQW49_RS07070 as seed ortholog is 100%.
Bootstrap support for CQW49_07060 as seed ortholog is 100%.
```

---

### Group of orthologs #911. Best score 835 bits Score difference with first non-orthologous sequence - OB3b\_Refseq\_for\_inparanoid.fasta:835 OB3b\_Genbank\_for\_inparanoid.fasta:835

```
CQW49_RS02150       	100.00%		CQW49_02145         	100.00%
Bootstrap support for CQW49_RS02150 as seed ortholog is 100%.
Bootstrap support for CQW49_02145 as seed ortholog is 100%.
```

---

### Group of orthologs #912. Best score 835 bits Score difference with first non-orthologous sequence - OB3b\_Refseq\_for\_inparanoid.fasta:835 OB3b\_Genbank\_for\_inparanoid.fasta:835

```
CQW49_RS02830       	100.00%		CQW49_02820         	100.00%
Bootstrap support for CQW49_RS02830 as seed ortholog is 100%.
Bootstrap support for CQW49_02820 as seed ortholog is 100%.
```

---

### Group of orthologs #913. Best score 835 bits Score difference with first non-orthologous sequence - OB3b\_Refseq\_for\_inparanoid.fasta:835 OB3b\_Genbank\_for\_inparanoid.fasta:835

```
CQW49_RS11075       	100.00%		CQW49_11045         	100.00%
Bootstrap support for CQW49_RS11075 as seed ortholog is 100%.
Bootstrap support for CQW49_11045 as seed ortholog is 100%.
```

---

### Group of orthologs #914. Best score 835 bits Score difference with first non-orthologous sequence - OB3b\_Refseq\_for\_inparanoid.fasta:835 OB3b\_Genbank\_for\_inparanoid.fasta:835

```
CQW49_RS12805       	100.00%		CQW49_12770         	100.00%
Bootstrap support for CQW49_RS12805 as seed ortholog is 100%.
Bootstrap support for CQW49_12770 as seed ortholog is 100%.
```

---

### Group of orthologs #915. Best score 834 bits Score difference with first non-orthologous sequence - OB3b\_Refseq\_for\_inparanoid.fasta:834 OB3b\_Genbank\_for\_inparanoid.fasta:834

```
CQW49_RS05460       	100.00%		CQW49_05450         	100.00%
Bootstrap support for CQW49_RS05460 as seed ortholog is 100%.
Bootstrap support for CQW49_05450 as seed ortholog is 100%.
```

---

### Group of orthologs #916. Best score 834 bits Score difference with first non-orthologous sequence - OB3b\_Refseq\_for\_inparanoid.fasta:834 OB3b\_Genbank\_for\_inparanoid.fasta:834

```
lysA                	100.00%		CQW49_10715         	100.00%
Bootstrap support for lysA as seed ortholog is 100%.
Bootstrap support for CQW49_10715 as seed ortholog is 100%.
```

---

### Group of orthologs #917. Best score 834 bits Score difference with first non-orthologous sequence - OB3b\_Refseq\_for\_inparanoid.fasta:834 OB3b\_Genbank\_for\_inparanoid.fasta:834

```
CQW49_RS15710       	100.00%		CQW49_15675         	100.00%
Bootstrap support for CQW49_RS15710 as seed ortholog is 100%.
Bootstrap support for CQW49_15675 as seed ortholog is 100%.
```

---

### Group of orthologs #918. Best score 834 bits Score difference with first non-orthologous sequence - OB3b\_Refseq\_for\_inparanoid.fasta:834 OB3b\_Genbank\_for\_inparanoid.fasta:834

```
CQW49_RS17660       	100.00%		CQW49_17605         	100.00%
Bootstrap support for CQW49_RS17660 as seed ortholog is 100%.
Bootstrap support for CQW49_17605 as seed ortholog is 100%.
```

---

### Group of orthologs #919. Best score 833 bits Score difference with first non-orthologous sequence - OB3b\_Refseq\_for\_inparanoid.fasta:833 OB3b\_Genbank\_for\_inparanoid.fasta:414

```
CQW49_RS01400       	100.00%		CQW49_01395         	100.00%
Bootstrap support for CQW49_RS01400 as seed ortholog is 100%.
Bootstrap support for CQW49_01395 as seed ortholog is 100%.
```

---

### Group of orthologs #920. Best score 833 bits Score difference with first non-orthologous sequence - OB3b\_Refseq\_for\_inparanoid.fasta:833 OB3b\_Genbank\_for\_inparanoid.fasta:752

```
CQW49_RS04315       	100.00%		CQW49_04305         	100.00%
Bootstrap support for CQW49_RS04315 as seed ortholog is 100%.
Bootstrap support for CQW49_04305 as seed ortholog is 100%.
```

---

### Group of orthologs #921. Best score 833 bits Score difference with first non-orthologous sequence - OB3b\_Refseq\_for\_inparanoid.fasta:833 OB3b\_Genbank\_for\_inparanoid.fasta:833

```
CQW49_RS04415       	100.00%		CQW49_04410         	100.00%
Bootstrap support for CQW49_RS04415 as seed ortholog is 100%.
Bootstrap support for CQW49_04410 as seed ortholog is 100%.
```

---

### Group of orthologs #922. Best score 833 bits Score difference with first non-orthologous sequence - OB3b\_Refseq\_for\_inparanoid.fasta:833 OB3b\_Genbank\_for\_inparanoid.fasta:833

```
CQW49_RS09065       	100.00%		CQW49_09050         	100.00%
Bootstrap support for CQW49_RS09065 as seed ortholog is 100%.
Bootstrap support for CQW49_09050 as seed ortholog is 100%.
```

---

### Group of orthologs #923. Best score 833 bits Score difference with first non-orthologous sequence - OB3b\_Refseq\_for\_inparanoid.fasta:833 OB3b\_Genbank\_for\_inparanoid.fasta:715

```
CQW49_RS17515       	100.00%		CQW49_17460         	100.00%
Bootstrap support for CQW49_RS17515 as seed ortholog is 100%.
Bootstrap support for CQW49_17460 as seed ortholog is 100%.
```

---

### Group of orthologs #924. Best score 832 bits Score difference with first non-orthologous sequence - OB3b\_Refseq\_for\_inparanoid.fasta:832 OB3b\_Genbank\_for\_inparanoid.fasta:832

```
CQW49_RS10890       	100.00%		CQW49_10860         	100.00%
Bootstrap support for CQW49_RS10890 as seed ortholog is 100%.
Bootstrap support for CQW49_10860 as seed ortholog is 100%.
```

---

### Group of orthologs #925. Best score 832 bits Score difference with first non-orthologous sequence - OB3b\_Refseq\_for\_inparanoid.fasta:832 OB3b\_Genbank\_for\_inparanoid.fasta:832

```
CQW49_RS17925       	100.00%		CQW49_17870         	100.00%
Bootstrap support for CQW49_RS17925 as seed ortholog is 100%.
Bootstrap support for CQW49_17870 as seed ortholog is 100%.
```

---

### Group of orthologs #926. Best score 832 bits Score difference with first non-orthologous sequence - OB3b\_Refseq\_for\_inparanoid.fasta:832 OB3b\_Genbank\_for\_inparanoid.fasta:832

```
CQW49_RS19170       	100.00%		CQW49_19110         	100.00%
Bootstrap support for CQW49_RS19170 as seed ortholog is 100%.
Bootstrap support for CQW49_19110 as seed ortholog is 100%.
```

---

### Group of orthologs #927. Best score 832 bits Score difference with first non-orthologous sequence - OB3b\_Refseq\_for\_inparanoid.fasta:832 OB3b\_Genbank\_for\_inparanoid.fasta:832

```
CQW49_RS20930       	100.00%		CQW49_20855         	100.00%
Bootstrap support for CQW49_RS20930 as seed ortholog is 100%.
Bootstrap support for CQW49_20855 as seed ortholog is 100%.
```

---

### Group of orthologs #928. Best score 832 bits Score difference with first non-orthologous sequence - OB3b\_Refseq\_for\_inparanoid.fasta:832 OB3b\_Genbank\_for\_inparanoid.fasta:832

```
CQW49_RS21105       	100.00%		CQW49_21030         	100.00%
Bootstrap support for CQW49_RS21105 as seed ortholog is 100%.
Bootstrap support for CQW49_21030 as seed ortholog is 100%.
```

---

### Group of orthologs #929. Best score 831 bits Score difference with first non-orthologous sequence - OB3b\_Refseq\_for\_inparanoid.fasta:831 OB3b\_Genbank\_for\_inparanoid.fasta:831

```
CQW49_RS07695       	100.00%		CQW49_07685         	100.00%
Bootstrap support for CQW49_RS07695 as seed ortholog is 100%.
Bootstrap support for CQW49_07685 as seed ortholog is 100%.
```

---

### Group of orthologs #930. Best score 831 bits Score difference with first non-orthologous sequence - OB3b\_Refseq\_for\_inparanoid.fasta:831 OB3b\_Genbank\_for\_inparanoid.fasta:831

```
CQW49_RS11765       	100.00%		CQW49_11730         	100.00%
Bootstrap support for CQW49_RS11765 as seed ortholog is 100%.
Bootstrap support for CQW49_11730 as seed ortholog is 100%.
```

---

### Group of orthologs #931. Best score 830 bits Score difference with first non-orthologous sequence - OB3b\_Refseq\_for\_inparanoid.fasta:830 OB3b\_Genbank\_for\_inparanoid.fasta:830

```
hisD                	100.00%		CQW49_00600         	100.00%
Bootstrap support for hisD as seed ortholog is 100%.
Bootstrap support for CQW49_00600 as seed ortholog is 100%.
```

---

### Group of orthologs #932. Best score 830 bits Score difference with first non-orthologous sequence - OB3b\_Refseq\_for\_inparanoid.fasta:830 OB3b\_Genbank\_for\_inparanoid.fasta:677

```
CQW49_RS14945       	100.00%		CQW49_14910         	100.00%
Bootstrap support for CQW49_RS14945 as seed ortholog is 100%.
Bootstrap support for CQW49_14910 as seed ortholog is 100%.
```

---

### Group of orthologs #933. Best score 830 bits Score difference with first non-orthologous sequence - OB3b\_Refseq\_for\_inparanoid.fasta:830 OB3b\_Genbank\_for\_inparanoid.fasta:830

```
CQW49_RS16940       	100.00%		CQW49_16895         	100.00%
Bootstrap support for CQW49_RS16940 as seed ortholog is 100%.
Bootstrap support for CQW49_16895 as seed ortholog is 100%.
```

---

### Group of orthologs #934. Best score 829 bits Score difference with first non-orthologous sequence - OB3b\_Refseq\_for\_inparanoid.fasta:829 OB3b\_Genbank\_for\_inparanoid.fasta:829

```
CQW49_RS00710       	100.00%		CQW49_00710         	100.00%
Bootstrap support for CQW49_RS00710 as seed ortholog is 100%.
Bootstrap support for CQW49_00710 as seed ortholog is 100%.
```

---

### Group of orthologs #935. Best score 829 bits Score difference with first non-orthologous sequence - OB3b\_Refseq\_for\_inparanoid.fasta:829 OB3b\_Genbank\_for\_inparanoid.fasta:829

```
CQW49_RS02260       	100.00%		CQW49_02255         	100.00%
Bootstrap support for CQW49_RS02260 as seed ortholog is 100%.
Bootstrap support for CQW49_02255 as seed ortholog is 100%.
```

---

### Group of orthologs #936. Best score 829 bits Score difference with first non-orthologous sequence - OB3b\_Refseq\_for\_inparanoid.fasta:829 OB3b\_Genbank\_for\_inparanoid.fasta:829

```
CQW49_RS09605       	100.00%		CQW49_09585         	100.00%
Bootstrap support for CQW49_RS09605 as seed ortholog is 100%.
Bootstrap support for CQW49_09585 as seed ortholog is 100%.
```

---

### Group of orthologs #937. Best score 829 bits Score difference with first non-orthologous sequence - OB3b\_Refseq\_for\_inparanoid.fasta:829 OB3b\_Genbank\_for\_inparanoid.fasta:829

```
CQW49_RS17080       	100.00%		CQW49_17035         	100.00%
Bootstrap support for CQW49_RS17080 as seed ortholog is 100%.
Bootstrap support for CQW49_17035 as seed ortholog is 100%.
```

---

### Group of orthologs #938. Best score 828 bits Score difference with first non-orthologous sequence - OB3b\_Refseq\_for\_inparanoid.fasta:828 OB3b\_Genbank\_for\_inparanoid.fasta:828

```
sufD                	100.00%		CQW49_01400         	100.00%
Bootstrap support for sufD as seed ortholog is 100%.
Bootstrap support for CQW49_01400 as seed ortholog is 100%.
```

---

### Group of orthologs #939. Best score 827 bits Score difference with first non-orthologous sequence - OB3b\_Refseq\_for\_inparanoid.fasta:827 OB3b\_Genbank\_for\_inparanoid.fasta:827

```
CQW49_RS07440       	100.00%		CQW49_07430         	100.00%
Bootstrap support for CQW49_RS07440 as seed ortholog is 100%.
Bootstrap support for CQW49_07430 as seed ortholog is 100%.
```

---

### Group of orthologs #940. Best score 826 bits Score difference with first non-orthologous sequence - OB3b\_Refseq\_for\_inparanoid.fasta:826 OB3b\_Genbank\_for\_inparanoid.fasta:826

```
CQW49_RS00680       	100.00%		CQW49_00680         	100.00%
Bootstrap support for CQW49_RS00680 as seed ortholog is 100%.
Bootstrap support for CQW49_00680 as seed ortholog is 100%.
```

---

### Group of orthologs #941. Best score 826 bits Score difference with first non-orthologous sequence - OB3b\_Refseq\_for\_inparanoid.fasta:826 OB3b\_Genbank\_for\_inparanoid.fasta:826

```
CQW49_RS00775       	100.00%		CQW49_00775         	100.00%
Bootstrap support for CQW49_RS00775 as seed ortholog is 100%.
Bootstrap support for CQW49_00775 as seed ortholog is 100%.
```

---

### Group of orthologs #942. Best score 826 bits Score difference with first non-orthologous sequence - OB3b\_Refseq\_for\_inparanoid.fasta:826 OB3b\_Genbank\_for\_inparanoid.fasta:616

```
CQW49_RS09595       	100.00%		CQW49_09575         	100.00%
Bootstrap support for CQW49_RS09595 as seed ortholog is 100%.
Bootstrap support for CQW49_09575 as seed ortholog is 100%.
```

---

### Group of orthologs #943. Best score 825 bits Score difference with first non-orthologous sequence - OB3b\_Refseq\_for\_inparanoid.fasta:825 OB3b\_Genbank\_for\_inparanoid.fasta:825

```
CQW49_RS01515       	100.00%		CQW49_01510         	100.00%
Bootstrap support for CQW49_RS01515 as seed ortholog is 100%.
Bootstrap support for CQW49_01510 as seed ortholog is 100%.
```

---

### Group of orthologs #944. Best score 825 bits Score difference with first non-orthologous sequence - OB3b\_Refseq\_for\_inparanoid.fasta:825 OB3b\_Genbank\_for\_inparanoid.fasta:825

```
CQW49_RS08635       	100.00%		CQW49_08625         	100.00%
Bootstrap support for CQW49_RS08635 as seed ortholog is 100%.
Bootstrap support for CQW49_08625 as seed ortholog is 100%.
```

---

### Group of orthologs #945. Best score 825 bits Score difference with first non-orthologous sequence - OB3b\_Refseq\_for\_inparanoid.fasta:825 OB3b\_Genbank\_for\_inparanoid.fasta:825

```
CQW49_RS15490       	100.00%		CQW49_15455         	100.00%
Bootstrap support for CQW49_RS15490 as seed ortholog is 100%.
Bootstrap support for CQW49_15455 as seed ortholog is 100%.
```

---

### Group of orthologs #946. Best score 825 bits Score difference with first non-orthologous sequence - OB3b\_Refseq\_for\_inparanoid.fasta:825 OB3b\_Genbank\_for\_inparanoid.fasta:825

```
CQW49_RS20265       	100.00%		CQW49_20195         	100.00%
Bootstrap support for CQW49_RS20265 as seed ortholog is 100%.
Bootstrap support for CQW49_20195 as seed ortholog is 100%.
```

---

### Group of orthologs #947. Best score 824 bits Score difference with first non-orthologous sequence - OB3b\_Refseq\_for\_inparanoid.fasta:824 OB3b\_Genbank\_for\_inparanoid.fasta:824

```
CQW49_RS05575       	100.00%		CQW49_05565         	100.00%
Bootstrap support for CQW49_RS05575 as seed ortholog is 100%.
Bootstrap support for CQW49_05565 as seed ortholog is 100%.
```

---

### Group of orthologs #948. Best score 824 bits Score difference with first non-orthologous sequence - OB3b\_Refseq\_for\_inparanoid.fasta:824 OB3b\_Genbank\_for\_inparanoid.fasta:824

```
CQW49_RS12510       	100.00%		CQW49_12475         	100.00%
Bootstrap support for CQW49_RS12510 as seed ortholog is 100%.
Bootstrap support for CQW49_12475 as seed ortholog is 100%.
```

---

### Group of orthologs #949. Best score 824 bits Score difference with first non-orthologous sequence - OB3b\_Refseq\_for\_inparanoid.fasta:824 OB3b\_Genbank\_for\_inparanoid.fasta:824

```
CQW49_RS16145       	100.00%		CQW49_16110         	100.00%
Bootstrap support for CQW49_RS16145 as seed ortholog is 100%.
Bootstrap support for CQW49_16110 as seed ortholog is 100%.
```

---

### Group of orthologs #950. Best score 824 bits Score difference with first non-orthologous sequence - OB3b\_Refseq\_for\_inparanoid.fasta:824 OB3b\_Genbank\_for\_inparanoid.fasta:824

```
CQW49_RS19980       	100.00%		CQW49_19915         	100.00%
Bootstrap support for CQW49_RS19980 as seed ortholog is 100%.
Bootstrap support for CQW49_19915 as seed ortholog is 100%.
```

---

### Group of orthologs #951. Best score 824 bits Score difference with first non-orthologous sequence - OB3b\_Refseq\_for\_inparanoid.fasta:824 OB3b\_Genbank\_for\_inparanoid.fasta:824

```
CQW49_RS20830       	100.00%		CQW49_20755         	100.00%
Bootstrap support for CQW49_RS20830 as seed ortholog is 100%.
Bootstrap support for CQW49_20755 as seed ortholog is 100%.
```

---

### Group of orthologs #952. Best score 822 bits Score difference with first non-orthologous sequence - OB3b\_Refseq\_for\_inparanoid.fasta:822 OB3b\_Genbank\_for\_inparanoid.fasta:822

```
CQW49_RS04505       	100.00%		CQW49_04500         	100.00%
Bootstrap support for CQW49_RS04505 as seed ortholog is 100%.
Bootstrap support for CQW49_04500 as seed ortholog is 100%.
```

---

### Group of orthologs #953. Best score 822 bits Score difference with first non-orthologous sequence - OB3b\_Refseq\_for\_inparanoid.fasta:822 OB3b\_Genbank\_for\_inparanoid.fasta:822

```
CQW49_RS12280       	100.00%		CQW49_12245         	100.00%
Bootstrap support for CQW49_RS12280 as seed ortholog is 100%.
Bootstrap support for CQW49_12245 as seed ortholog is 100%.
```

---

### Group of orthologs #954. Best score 822 bits Score difference with first non-orthologous sequence - OB3b\_Refseq\_for\_inparanoid.fasta:822 OB3b\_Genbank\_for\_inparanoid.fasta:822

```
CQW49_RS19350       	100.00%		CQW49_19295         	100.00%
Bootstrap support for CQW49_RS19350 as seed ortholog is 100%.
Bootstrap support for CQW49_19295 as seed ortholog is 100%.
```

---

### Group of orthologs #955. Best score 821 bits Score difference with first non-orthologous sequence - OB3b\_Refseq\_for\_inparanoid.fasta:821 OB3b\_Genbank\_for\_inparanoid.fasta:821

```
CQW49_RS06920       	100.00%		CQW49_06910         	100.00%
Bootstrap support for CQW49_RS06920 as seed ortholog is 100%.
Bootstrap support for CQW49_06910 as seed ortholog is 100%.
```

---

### Group of orthologs #956. Best score 821 bits Score difference with first non-orthologous sequence - OB3b\_Refseq\_for\_inparanoid.fasta:821 OB3b\_Genbank\_for\_inparanoid.fasta:821

```
CQW49_RS15660       	100.00%		CQW49_15625         	100.00%
Bootstrap support for CQW49_RS15660 as seed ortholog is 100%.
Bootstrap support for CQW49_15625 as seed ortholog is 100%.
```

---

### Group of orthologs #957. Best score 820 bits Score difference with first non-orthologous sequence - OB3b\_Refseq\_for\_inparanoid.fasta:820 OB3b\_Genbank\_for\_inparanoid.fasta:820

```
CQW49_RS07305       	100.00%		CQW49_07295         	100.00%
Bootstrap support for CQW49_RS07305 as seed ortholog is 100%.
Bootstrap support for CQW49_07295 as seed ortholog is 100%.
```

---

### Group of orthologs #958. Best score 819 bits Score difference with first non-orthologous sequence - OB3b\_Refseq\_for\_inparanoid.fasta:819 OB3b\_Genbank\_for\_inparanoid.fasta:819

```
CQW49_RS01455       	100.00%		CQW49_01450         	100.00%
Bootstrap support for CQW49_RS01455 as seed ortholog is 100%.
Bootstrap support for CQW49_01450 as seed ortholog is 100%.
```

---

### Group of orthologs #959. Best score 819 bits Score difference with first non-orthologous sequence - OB3b\_Refseq\_for\_inparanoid.fasta:819 OB3b\_Genbank\_for\_inparanoid.fasta:563

```
CQW49_RS01785       	100.00%		CQW49_01780         	100.00%
Bootstrap support for CQW49_RS01785 as seed ortholog is 100%.
Bootstrap support for CQW49_01780 as seed ortholog is 100%.
```

---

### Group of orthologs #960. Best score 819 bits Score difference with first non-orthologous sequence - OB3b\_Refseq\_for\_inparanoid.fasta:819 OB3b\_Genbank\_for\_inparanoid.fasta:819

```
rho                 	100.00%		CQW49_12775         	100.00%
Bootstrap support for rho as seed ortholog is 100%.
Bootstrap support for CQW49_12775 as seed ortholog is 100%.
```

---

### Group of orthologs #961. Best score 819 bits Score difference with first non-orthologous sequence - OB3b\_Refseq\_for\_inparanoid.fasta:819 OB3b\_Genbank\_for\_inparanoid.fasta:819

```
CQW49_RS16150       	100.00%		CQW49_16115         	100.00%
Bootstrap support for CQW49_RS16150 as seed ortholog is 100%.
Bootstrap support for CQW49_16115 as seed ortholog is 100%.
```

---

### Group of orthologs #962. Best score 818 bits Score difference with first non-orthologous sequence - OB3b\_Refseq\_for\_inparanoid.fasta:818 OB3b\_Genbank\_for\_inparanoid.fasta:818

```
CQW49_RS03895       	100.00%		CQW49_03885         	100.00%
Bootstrap support for CQW49_RS03895 as seed ortholog is 100%.
Bootstrap support for CQW49_03885 as seed ortholog is 100%.
```

---

### Group of orthologs #963. Best score 817 bits Score difference with first non-orthologous sequence - OB3b\_Refseq\_for\_inparanoid.fasta:817 OB3b\_Genbank\_for\_inparanoid.fasta:817

```
CQW49_RS05340       	100.00%		CQW49_05335         	100.00%
Bootstrap support for CQW49_RS05340 as seed ortholog is 100%.
Bootstrap support for CQW49_05335 as seed ortholog is 100%.
```

---

### Group of orthologs #964. Best score 817 bits Score difference with first non-orthologous sequence - OB3b\_Refseq\_for\_inparanoid.fasta:817 OB3b\_Genbank\_for\_inparanoid.fasta:760

```
CQW49_RS05630       	100.00%		CQW49_05620         	100.00%
Bootstrap support for CQW49_RS05630 as seed ortholog is 100%.
Bootstrap support for CQW49_05620 as seed ortholog is 100%.
```

---

### Group of orthologs #965. Best score 817 bits Score difference with first non-orthologous sequence - OB3b\_Refseq\_for\_inparanoid.fasta:817 OB3b\_Genbank\_for\_inparanoid.fasta:817

```
CQW49_RS06645       	100.00%		CQW49_06635         	100.00%
Bootstrap support for CQW49_RS06645 as seed ortholog is 100%.
Bootstrap support for CQW49_06635 as seed ortholog is 100%.
```

---

### Group of orthologs #966. Best score 817 bits Score difference with first non-orthologous sequence - OB3b\_Refseq\_for\_inparanoid.fasta:817 OB3b\_Genbank\_for\_inparanoid.fasta:817

```
CQW49_RS18410       	100.00%		CQW49_18350         	100.00%
Bootstrap support for CQW49_RS18410 as seed ortholog is 100%.
Bootstrap support for CQW49_18350 as seed ortholog is 100%.
```

---

### Group of orthologs #967. Best score 816 bits Score difference with first non-orthologous sequence - OB3b\_Refseq\_for\_inparanoid.fasta:816 OB3b\_Genbank\_for\_inparanoid.fasta:816

```
CQW49_RS13550       	100.00%		CQW49_13515         	100.00%
Bootstrap support for CQW49_RS13550 as seed ortholog is 100%.
Bootstrap support for CQW49_13515 as seed ortholog is 100%.
```

---

### Group of orthologs #968. Best score 815 bits Score difference with first non-orthologous sequence - OB3b\_Refseq\_for\_inparanoid.fasta:815 OB3b\_Genbank\_for\_inparanoid.fasta:815

```
CQW49_RS16430       	100.00%		CQW49_16395         	100.00%
Bootstrap support for CQW49_RS16430 as seed ortholog is 100%.
Bootstrap support for CQW49_16395 as seed ortholog is 100%.
```

---

### Group of orthologs #969. Best score 814 bits Score difference with first non-orthologous sequence - OB3b\_Refseq\_for\_inparanoid.fasta:814 OB3b\_Genbank\_for\_inparanoid.fasta:814

```
CQW49_RS00130       	100.00%		CQW49_00130         	100.00%
Bootstrap support for CQW49_RS00130 as seed ortholog is 100%.
Bootstrap support for CQW49_00130 as seed ortholog is 100%.
```

---

### Group of orthologs #970. Best score 814 bits Score difference with first non-orthologous sequence - OB3b\_Refseq\_for\_inparanoid.fasta:814 OB3b\_Genbank\_for\_inparanoid.fasta:814

```
CQW49_RS03495       	100.00%		CQW49_03485         	100.00%
Bootstrap support for CQW49_RS03495 as seed ortholog is 100%.
Bootstrap support for CQW49_03485 as seed ortholog is 100%.
```

---

### Group of orthologs #971. Best score 814 bits Score difference with first non-orthologous sequence - OB3b\_Refseq\_for\_inparanoid.fasta:814 OB3b\_Genbank\_for\_inparanoid.fasta:639

```
CQW49_RS05595       	100.00%		CQW49_05585         	100.00%
Bootstrap support for CQW49_RS05595 as seed ortholog is 100%.
Bootstrap support for CQW49_05585 as seed ortholog is 100%.
```

---

### Group of orthologs #972. Best score 814 bits Score difference with first non-orthologous sequence - OB3b\_Refseq\_for\_inparanoid.fasta:814 OB3b\_Genbank\_for\_inparanoid.fasta:814

```
CQW49_RS08190       	100.00%		CQW49_08180         	100.00%
Bootstrap support for CQW49_RS08190 as seed ortholog is 100%.
Bootstrap support for CQW49_08180 as seed ortholog is 100%.
```

---

### Group of orthologs #973. Best score 814 bits Score difference with first non-orthologous sequence - OB3b\_Refseq\_for\_inparanoid.fasta:814 OB3b\_Genbank\_for\_inparanoid.fasta:814

```
CQW49_RS13875       	100.00%		CQW49_13840         	100.00%
Bootstrap support for CQW49_RS13875 as seed ortholog is 100%.
Bootstrap support for CQW49_13840 as seed ortholog is 100%.
```

---

### Group of orthologs #974. Best score 813 bits Score difference with first non-orthologous sequence - OB3b\_Refseq\_for\_inparanoid.fasta:813 OB3b\_Genbank\_for\_inparanoid.fasta:813

```
CQW49_RS06875       	100.00%		CQW49_06865         	100.00%
Bootstrap support for CQW49_RS06875 as seed ortholog is 100%.
Bootstrap support for CQW49_06865 as seed ortholog is 100%.
```

---

### Group of orthologs #975. Best score 813 bits Score difference with first non-orthologous sequence - OB3b\_Refseq\_for\_inparanoid.fasta:813 OB3b\_Genbank\_for\_inparanoid.fasta:813

```
CQW49_RS08425       	100.00%		CQW49_08415         	100.00%
Bootstrap support for CQW49_RS08425 as seed ortholog is 100%.
Bootstrap support for CQW49_08415 as seed ortholog is 100%.
```

---

### Group of orthologs #976. Best score 813 bits Score difference with first non-orthologous sequence - OB3b\_Refseq\_for\_inparanoid.fasta:813 OB3b\_Genbank\_for\_inparanoid.fasta:641

```
rocD                	100.00%		CQW49_17895         	100.00%
Bootstrap support for rocD as seed ortholog is 100%.
Bootstrap support for CQW49_17895 as seed ortholog is 100%.
```

---

### Group of orthologs #977. Best score 812 bits Score difference with first non-orthologous sequence - OB3b\_Refseq\_for\_inparanoid.fasta:812 OB3b\_Genbank\_for\_inparanoid.fasta:812

```
CQW49_RS07285       	100.00%		CQW49_07275         	100.00%
Bootstrap support for CQW49_RS07285 as seed ortholog is 100%.
Bootstrap support for CQW49_07275 as seed ortholog is 100%.
```

---

### Group of orthologs #978. Best score 812 bits Score difference with first non-orthologous sequence - OB3b\_Refseq\_for\_inparanoid.fasta:812 OB3b\_Genbank\_for\_inparanoid.fasta:812

```
CQW49_RS16625       	100.00%		CQW49_16585         	100.00%
Bootstrap support for CQW49_RS16625 as seed ortholog is 100%.
Bootstrap support for CQW49_16585 as seed ortholog is 100%.
```

---

### Group of orthologs #979. Best score 811 bits Score difference with first non-orthologous sequence - OB3b\_Refseq\_for\_inparanoid.fasta:811 OB3b\_Genbank\_for\_inparanoid.fasta:811

```
CQW49_RS10090       	100.00%		CQW49_10065         	100.00%
Bootstrap support for CQW49_RS10090 as seed ortholog is 100%.
Bootstrap support for CQW49_10065 as seed ortholog is 100%.
```

---

### Group of orthologs #980. Best score 811 bits Score difference with first non-orthologous sequence - OB3b\_Refseq\_for\_inparanoid.fasta:811 OB3b\_Genbank\_for\_inparanoid.fasta:811

```
CQW49_RS18045       	100.00%		CQW49_17985         	100.00%
Bootstrap support for CQW49_RS18045 as seed ortholog is 100%.
Bootstrap support for CQW49_17985 as seed ortholog is 100%.
```

---

### Group of orthologs #981. Best score 810 bits Score difference with first non-orthologous sequence - OB3b\_Refseq\_for\_inparanoid.fasta:810 OB3b\_Genbank\_for\_inparanoid.fasta:486

```
CQW49_RS09255       	100.00%		CQW49_09235         	100.00%
Bootstrap support for CQW49_RS09255 as seed ortholog is 100%.
Bootstrap support for CQW49_09235 as seed ortholog is 100%.
```

---

### Group of orthologs #982. Best score 810 bits Score difference with first non-orthologous sequence - OB3b\_Refseq\_for\_inparanoid.fasta:810 OB3b\_Genbank\_for\_inparanoid.fasta:810

```
CQW49_RS10985       	100.00%		CQW49_10955         	100.00%
Bootstrap support for CQW49_RS10985 as seed ortholog is 100%.
Bootstrap support for CQW49_10955 as seed ortholog is 100%.
```

---

### Group of orthologs #983. Best score 810 bits Score difference with first non-orthologous sequence - OB3b\_Refseq\_for\_inparanoid.fasta:810 OB3b\_Genbank\_for\_inparanoid.fasta:810

```
CQW49_RS11990       	100.00%		CQW49_11955         	100.00%
Bootstrap support for CQW49_RS11990 as seed ortholog is 100%.
Bootstrap support for CQW49_11955 as seed ortholog is 100%.
```

---

### Group of orthologs #984. Best score 810 bits Score difference with first non-orthologous sequence - OB3b\_Refseq\_for\_inparanoid.fasta:810 OB3b\_Genbank\_for\_inparanoid.fasta:414

```
CQW49_RS13685       	100.00%		CQW49_13650         	100.00%
Bootstrap support for CQW49_RS13685 as seed ortholog is 100%.
Bootstrap support for CQW49_13650 as seed ortholog is 100%.
```

---

### Group of orthologs #985. Best score 809 bits Score difference with first non-orthologous sequence - OB3b\_Refseq\_for\_inparanoid.fasta:809 OB3b\_Genbank\_for\_inparanoid.fasta:691

```
CQW49_RS09335       	100.00%		CQW49_09315         	100.00%
Bootstrap support for CQW49_RS09335 as seed ortholog is 100%.
Bootstrap support for CQW49_09315 as seed ortholog is 100%.
```

---

### Group of orthologs #986. Best score 809 bits Score difference with first non-orthologous sequence - OB3b\_Refseq\_for\_inparanoid.fasta:809 OB3b\_Genbank\_for\_inparanoid.fasta:809

```
CQW49_RS18755       	100.00%		CQW49_18695         	100.00%
Bootstrap support for CQW49_RS18755 as seed ortholog is 100%.
Bootstrap support for CQW49_18695 as seed ortholog is 100%.
```

---

### Group of orthologs #987. Best score 808 bits Score difference with first non-orthologous sequence - OB3b\_Refseq\_for\_inparanoid.fasta:808 OB3b\_Genbank\_for\_inparanoid.fasta:808

```
CQW49_RS07040       	100.00%		CQW49_07030         	100.00%
Bootstrap support for CQW49_RS07040 as seed ortholog is 100%.
Bootstrap support for CQW49_07030 as seed ortholog is 100%.
```

---

### Group of orthologs #988. Best score 808 bits Score difference with first non-orthologous sequence - OB3b\_Refseq\_for\_inparanoid.fasta:808 OB3b\_Genbank\_for\_inparanoid.fasta:808

```
CQW49_RS07870       	100.00%		CQW49_07860         	100.00%
Bootstrap support for CQW49_RS07870 as seed ortholog is 100%.
Bootstrap support for CQW49_07860 as seed ortholog is 100%.
```

---

### Group of orthologs #989. Best score 808 bits Score difference with first non-orthologous sequence - OB3b\_Refseq\_for\_inparanoid.fasta:808 OB3b\_Genbank\_for\_inparanoid.fasta:808

```
CQW49_RS12750       	100.00%		CQW49_12715         	100.00%
Bootstrap support for CQW49_RS12750 as seed ortholog is 100%.
Bootstrap support for CQW49_12715 as seed ortholog is 100%.
```

---

### Group of orthologs #990. Best score 808 bits Score difference with first non-orthologous sequence - OB3b\_Refseq\_for\_inparanoid.fasta:808 OB3b\_Genbank\_for\_inparanoid.fasta:808

```
CQW49_RS13340       	100.00%		CQW49_13305         	100.00%
Bootstrap support for CQW49_RS13340 as seed ortholog is 100%.
Bootstrap support for CQW49_13305 as seed ortholog is 100%.
```

---

### Group of orthologs #991. Best score 807 bits Score difference with first non-orthologous sequence - OB3b\_Refseq\_for\_inparanoid.fasta:807 OB3b\_Genbank\_for\_inparanoid.fasta:761

```
CQW49_RS01670       	100.00%		CQW49_01665         	100.00%
Bootstrap support for CQW49_RS01670 as seed ortholog is 100%.
Bootstrap support for CQW49_01665 as seed ortholog is 100%.
```

---

### Group of orthologs #992. Best score 807 bits Score difference with first non-orthologous sequence - OB3b\_Refseq\_for\_inparanoid.fasta:807 OB3b\_Genbank\_for\_inparanoid.fasta:807

```
CQW49_RS03640       	100.00%		CQW49_03630         	100.00%
Bootstrap support for CQW49_RS03640 as seed ortholog is 100%.
Bootstrap support for CQW49_03630 as seed ortholog is 100%.
```

---

### Group of orthologs #993. Best score 807 bits Score difference with first non-orthologous sequence - OB3b\_Refseq\_for\_inparanoid.fasta:807 OB3b\_Genbank\_for\_inparanoid.fasta:807

```
CQW49_RS07450       	100.00%		CQW49_07440         	100.00%
Bootstrap support for CQW49_RS07450 as seed ortholog is 100%.
Bootstrap support for CQW49_07440 as seed ortholog is 100%.
```

---

### Group of orthologs #994. Best score 807 bits Score difference with first non-orthologous sequence - OB3b\_Refseq\_for\_inparanoid.fasta:807 OB3b\_Genbank\_for\_inparanoid.fasta:600

```
CQW49_RS11390       	100.00%		CQW49_11360         	100.00%
Bootstrap support for CQW49_RS11390 as seed ortholog is 100%.
Bootstrap support for CQW49_11360 as seed ortholog is 100%.
```

---

### Group of orthologs #995. Best score 807 bits Score difference with first non-orthologous sequence - OB3b\_Refseq\_for\_inparanoid.fasta:807 OB3b\_Genbank\_for\_inparanoid.fasta:807

```
CQW49_RS12000       	100.00%		CQW49_11965         	100.00%
Bootstrap support for CQW49_RS12000 as seed ortholog is 100%.
Bootstrap support for CQW49_11965 as seed ortholog is 100%.
```

---

### Group of orthologs #996. Best score 806 bits Score difference with first non-orthologous sequence - OB3b\_Refseq\_for\_inparanoid.fasta:806 OB3b\_Genbank\_for\_inparanoid.fasta:806

```
CQW49_RS17450       	100.00%		CQW49_17400         	100.00%
Bootstrap support for CQW49_RS17450 as seed ortholog is 100%.
Bootstrap support for CQW49_17400 as seed ortholog is 100%.
```

---

### Group of orthologs #997. Best score 805 bits Score difference with first non-orthologous sequence - OB3b\_Refseq\_for\_inparanoid.fasta:805 OB3b\_Genbank\_for\_inparanoid.fasta:805

```
CQW49_RS00455       	100.00%		CQW49_00455         	100.00%
Bootstrap support for CQW49_RS00455 as seed ortholog is 100%.
Bootstrap support for CQW49_00455 as seed ortholog is 100%.
```

---

### Group of orthologs #998. Best score 805 bits Score difference with first non-orthologous sequence - OB3b\_Refseq\_for\_inparanoid.fasta:805 OB3b\_Genbank\_for\_inparanoid.fasta:805

```
CQW49_RS03460       	100.00%		CQW49_03445         	100.00%
Bootstrap support for CQW49_RS03460 as seed ortholog is 100%.
Bootstrap support for CQW49_03445 as seed ortholog is 100%.
```

---

### Group of orthologs #999. Best score 805 bits Score difference with first non-orthologous sequence - OB3b\_Refseq\_for\_inparanoid.fasta:805 OB3b\_Genbank\_for\_inparanoid.fasta:805

```
CQW49_RS05535       	100.00%		CQW49_05525         	100.00%
Bootstrap support for CQW49_RS05535 as seed ortholog is 100%.
Bootstrap support for CQW49_05525 as seed ortholog is 100%.
```

---

### Group of orthologs #1000. Best score 805 bits Score difference with first non-orthologous sequence - OB3b\_Refseq\_for\_inparanoid.fasta:805 OB3b\_Genbank\_for\_inparanoid.fasta:805

```
CQW49_RS07675       	100.00%		CQW49_07665         	100.00%
Bootstrap support for CQW49_RS07675 as seed ortholog is 100%.
Bootstrap support for CQW49_07665 as seed ortholog is 100%.
```

---

### Group of orthologs #1001. Best score 805 bits Score difference with first non-orthologous sequence - OB3b\_Refseq\_for\_inparanoid.fasta:805 OB3b\_Genbank\_for\_inparanoid.fasta:805

```
CQW49_RS17415       	100.00%		CQW49_17365         	100.00%
Bootstrap support for CQW49_RS17415 as seed ortholog is 100%.
Bootstrap support for CQW49_17365 as seed ortholog is 100%.
```

---

### Group of orthologs #1002. Best score 804 bits Score difference with first non-orthologous sequence - OB3b\_Refseq\_for\_inparanoid.fasta:804 OB3b\_Genbank\_for\_inparanoid.fasta:804

```
CQW49_RS04855       	100.00%		CQW49_04845         	100.00%
Bootstrap support for CQW49_RS04855 as seed ortholog is 100%.
Bootstrap support for CQW49_04845 as seed ortholog is 100%.
```

---

### Group of orthologs #1003. Best score 804 bits Score difference with first non-orthologous sequence - OB3b\_Refseq\_for\_inparanoid.fasta:804 OB3b\_Genbank\_for\_inparanoid.fasta:804

```
CQW49_RS07595       	100.00%		CQW49_07585         	100.00%
Bootstrap support for CQW49_RS07595 as seed ortholog is 100%.
Bootstrap support for CQW49_07585 as seed ortholog is 100%.
```

---

### Group of orthologs #1004. Best score 804 bits Score difference with first non-orthologous sequence - OB3b\_Refseq\_for\_inparanoid.fasta:804 OB3b\_Genbank\_for\_inparanoid.fasta:674

```
CQW49_RS11580       	100.00%		CQW49_11545         	100.00%
Bootstrap support for CQW49_RS11580 as seed ortholog is 100%.
Bootstrap support for CQW49_11545 as seed ortholog is 100%.
```

---

### Group of orthologs #1005. Best score 802 bits Score difference with first non-orthologous sequence - OB3b\_Refseq\_for\_inparanoid.fasta:802 OB3b\_Genbank\_for\_inparanoid.fasta:802

```
CQW49_RS01265       	100.00%		CQW49_01260         	100.00%
Bootstrap support for CQW49_RS01265 as seed ortholog is 100%.
Bootstrap support for CQW49_01260 as seed ortholog is 100%.
```

---

### Group of orthologs #1006. Best score 802 bits Score difference with first non-orthologous sequence - OB3b\_Refseq\_for\_inparanoid.fasta:802 OB3b\_Genbank\_for\_inparanoid.fasta:802

```
CQW49_RS09315       	100.00%		CQW49_09295         	100.00%
Bootstrap support for CQW49_RS09315 as seed ortholog is 100%.
Bootstrap support for CQW49_09295 as seed ortholog is 100%.
```

---

### Group of orthologs #1007. Best score 802 bits Score difference with first non-orthologous sequence - OB3b\_Refseq\_for\_inparanoid.fasta:802 OB3b\_Genbank\_for\_inparanoid.fasta:802

```
CQW49_RS13380       	100.00%		CQW49_13345         	100.00%
Bootstrap support for CQW49_RS13380 as seed ortholog is 100%.
Bootstrap support for CQW49_13345 as seed ortholog is 100%.
```

---

### Group of orthologs #1008. Best score 802 bits Score difference with first non-orthologous sequence - OB3b\_Refseq\_for\_inparanoid.fasta:802 OB3b\_Genbank\_for\_inparanoid.fasta:802

```
CQW49_RS19445       	100.00%		CQW49_19390         	100.00%
Bootstrap support for CQW49_RS19445 as seed ortholog is 100%.
Bootstrap support for CQW49_19390 as seed ortholog is 100%.
```

---

### Group of orthologs #1009. Best score 801 bits Score difference with first non-orthologous sequence - OB3b\_Refseq\_for\_inparanoid.fasta:801 OB3b\_Genbank\_for\_inparanoid.fasta:2

```
CQW49_RS07210       	100.00%		CQW49_07200         	100.00%
CQW49_RS12130       	100.00%		CQW49_12095         	100.00%
CQW49_RS14570       	100.00%		CQW49_14535         	100.00%
CQW49_RS21095       	100.00%		CQW49_21020         	100.00%
CQW49_RS00615       	100.00%		CQW49_22350         	100.00%
                    	       		CQW49_00615         	100.00%
Bootstrap support for CQW49_RS07210 as seed ortholog is 100%.
Bootstrap support for CQW49_RS12130 as seed ortholog is 100%.
Bootstrap support for CQW49_RS14570 as seed ortholog is 100%.
Bootstrap support for CQW49_RS21095 as seed ortholog is 100%.
Bootstrap support for CQW49_RS00615 as seed ortholog is 100%.
Bootstrap support for CQW49_07200 as seed ortholog is 53%.
Alternative seed ortholog is CQW49_23920 (2 bits away from this cluster)
Bootstrap support for CQW49_12095 as seed ortholog is 54%.
Alternative seed ortholog is CQW49_23920 (2 bits away from this cluster)
Bootstrap support for CQW49_14535 as seed ortholog is 54%.
Alternative seed ortholog is CQW49_23920 (2 bits away from this cluster)
Bootstrap support for CQW49_21020 as seed ortholog is 54%.
Alternative seed ortholog is CQW49_23920 (2 bits away from this cluster)
Bootstrap support for CQW49_22350 as seed ortholog is 55%.
Alternative seed ortholog is CQW49_23920 (2 bits away from this cluster)
Bootstrap support for CQW49_00615 as seed ortholog is 53%.
Alternative seed ortholog is CQW49_23920 (2 bits away from this cluster)
```

---

### Group of orthologs #1010. Best score 801 bits Score difference with first non-orthologous sequence - OB3b\_Refseq\_for\_inparanoid.fasta:801 OB3b\_Genbank\_for\_inparanoid.fasta:801

```
CQW49_RS04075       	100.00%		CQW49_04065         	100.00%
Bootstrap support for CQW49_RS04075 as seed ortholog is 100%.
Bootstrap support for CQW49_04065 as seed ortholog is 100%.
```

---

### Group of orthologs #1011. Best score 801 bits Score difference with first non-orthologous sequence - OB3b\_Refseq\_for\_inparanoid.fasta:801 OB3b\_Genbank\_for\_inparanoid.fasta:801

```
CQW49_RS16070       	100.00%		CQW49_16035         	100.00%
Bootstrap support for CQW49_RS16070 as seed ortholog is 100%.
Bootstrap support for CQW49_16035 as seed ortholog is 100%.
```

---

### Group of orthologs #1012. Best score 800 bits Score difference with first non-orthologous sequence - OB3b\_Refseq\_for\_inparanoid.fasta:800 OB3b\_Genbank\_for\_inparanoid.fasta:800

```
CQW49_RS02310       	100.00%		CQW49_02305         	100.00%
Bootstrap support for CQW49_RS02310 as seed ortholog is 100%.
Bootstrap support for CQW49_02305 as seed ortholog is 100%.
```

---

### Group of orthologs #1013. Best score 800 bits Score difference with first non-orthologous sequence - OB3b\_Refseq\_for\_inparanoid.fasta:800 OB3b\_Genbank\_for\_inparanoid.fasta:800

```
CQW49_RS13915       	100.00%		CQW49_13880         	100.00%
Bootstrap support for CQW49_RS13915 as seed ortholog is 100%.
Bootstrap support for CQW49_13880 as seed ortholog is 100%.
```

---

### Group of orthologs #1014. Best score 800 bits Score difference with first non-orthologous sequence - OB3b\_Refseq\_for\_inparanoid.fasta:800 OB3b\_Genbank\_for\_inparanoid.fasta:134

```
CQW49_RS14000       	100.00%		CQW49_13965         	100.00%
Bootstrap support for CQW49_RS14000 as seed ortholog is 100%.
Bootstrap support for CQW49_13965 as seed ortholog is 100%.
```

---

### Group of orthologs #1015. Best score 800 bits Score difference with first non-orthologous sequence - OB3b\_Refseq\_for\_inparanoid.fasta:800 OB3b\_Genbank\_for\_inparanoid.fasta:800

```
CQW49_RS20710       	100.00%		CQW49_20635         	100.00%
Bootstrap support for CQW49_RS20710 as seed ortholog is 100%.
Bootstrap support for CQW49_20635 as seed ortholog is 100%.
```

---

### Group of orthologs #1016. Best score 799 bits Score difference with first non-orthologous sequence - OB3b\_Refseq\_for\_inparanoid.fasta:799 OB3b\_Genbank\_for\_inparanoid.fasta:799

```
CQW49_RS12325       	100.00%		CQW49_12290         	100.00%
Bootstrap support for CQW49_RS12325 as seed ortholog is 100%.
Bootstrap support for CQW49_12290 as seed ortholog is 100%.
```

---

### Group of orthologs #1017. Best score 799 bits Score difference with first non-orthologous sequence - OB3b\_Refseq\_for\_inparanoid.fasta:799 OB3b\_Genbank\_for\_inparanoid.fasta:799

```
CQW49_RS14245       	100.00%		CQW49_14210         	100.00%
Bootstrap support for CQW49_RS14245 as seed ortholog is 100%.
Bootstrap support for CQW49_14210 as seed ortholog is 100%.
```

---

### Group of orthologs #1018. Best score 798 bits Score difference with first non-orthologous sequence - OB3b\_Refseq\_for\_inparanoid.fasta:798 OB3b\_Genbank\_for\_inparanoid.fasta:798

```
CQW49_RS11850       	100.00%		CQW49_11815         	100.00%
Bootstrap support for CQW49_RS11850 as seed ortholog is 100%.
Bootstrap support for CQW49_11815 as seed ortholog is 100%.
```

---

### Group of orthologs #1019. Best score 797 bits Score difference with first non-orthologous sequence - OB3b\_Refseq\_for\_inparanoid.fasta:797 OB3b\_Genbank\_for\_inparanoid.fasta:797

```
CQW49_RS04695       	100.00%		CQW49_04685         	100.00%
Bootstrap support for CQW49_RS04695 as seed ortholog is 100%.
Bootstrap support for CQW49_04685 as seed ortholog is 100%.
```

---

### Group of orthologs #1020. Best score 797 bits Score difference with first non-orthologous sequence - OB3b\_Refseq\_for\_inparanoid.fasta:797 OB3b\_Genbank\_for\_inparanoid.fasta:797

```
CQW49_RS06360       	100.00%		CQW49_06350         	100.00%
Bootstrap support for CQW49_RS06360 as seed ortholog is 100%.
Bootstrap support for CQW49_06350 as seed ortholog is 100%.
```

---

### Group of orthologs #1021. Best score 797 bits Score difference with first non-orthologous sequence - OB3b\_Refseq\_for\_inparanoid.fasta:797 OB3b\_Genbank\_for\_inparanoid.fasta:797

```
CQW49_RS16695       	100.00%		CQW49_16655         	100.00%
Bootstrap support for CQW49_RS16695 as seed ortholog is 100%.
Bootstrap support for CQW49_16655 as seed ortholog is 100%.
```

---

### Group of orthologs #1022. Best score 796 bits Score difference with first non-orthologous sequence - OB3b\_Refseq\_for\_inparanoid.fasta:796 OB3b\_Genbank\_for\_inparanoid.fasta:796

```
CQW49_RS17190       	100.00%		CQW49_17145         	100.00%
Bootstrap support for CQW49_RS17190 as seed ortholog is 100%.
Bootstrap support for CQW49_17145 as seed ortholog is 100%.
```

---

### Group of orthologs #1023. Best score 796 bits Score difference with first non-orthologous sequence - OB3b\_Refseq\_for\_inparanoid.fasta:796 OB3b\_Genbank\_for\_inparanoid.fasta:796

```
CQW49_RS20235       	100.00%		CQW49_20165         	100.00%
Bootstrap support for CQW49_RS20235 as seed ortholog is 100%.
Bootstrap support for CQW49_20165 as seed ortholog is 100%.
```

---

### Group of orthologs #1024. Best score 795 bits Score difference with first non-orthologous sequence - OB3b\_Refseq\_for\_inparanoid.fasta:795 OB3b\_Genbank\_for\_inparanoid.fasta:795

```
dprA                	100.00%		CQW49_01075         	100.00%
Bootstrap support for dprA as seed ortholog is 100%.
Bootstrap support for CQW49_01075 as seed ortholog is 100%.
```

---

### Group of orthologs #1025. Best score 795 bits Score difference with first non-orthologous sequence - OB3b\_Refseq\_for\_inparanoid.fasta:795 OB3b\_Genbank\_for\_inparanoid.fasta:795

```
CQW49_RS06975       	100.00%		CQW49_06965         	100.00%
Bootstrap support for CQW49_RS06975 as seed ortholog is 100%.
Bootstrap support for CQW49_06965 as seed ortholog is 100%.
```

---

### Group of orthologs #1026. Best score 795 bits Score difference with first non-orthologous sequence - OB3b\_Refseq\_for\_inparanoid.fasta:795 OB3b\_Genbank\_for\_inparanoid.fasta:795

```
CQW49_RS21115       	100.00%		CQW49_21040         	100.00%
Bootstrap support for CQW49_RS21115 as seed ortholog is 100%.
Bootstrap support for CQW49_21040 as seed ortholog is 100%.
```

---

### Group of orthologs #1027. Best score 794 bits Score difference with first non-orthologous sequence - OB3b\_Refseq\_for\_inparanoid.fasta:794 OB3b\_Genbank\_for\_inparanoid.fasta:794

```
CQW49_RS00265       	100.00%		CQW49_00265         	100.00%
Bootstrap support for CQW49_RS00265 as seed ortholog is 100%.
Bootstrap support for CQW49_00265 as seed ortholog is 100%.
```

---

### Group of orthologs #1028. Best score 794 bits Score difference with first non-orthologous sequence - OB3b\_Refseq\_for\_inparanoid.fasta:794 OB3b\_Genbank\_for\_inparanoid.fasta:794

```
CQW49_RS04060       	100.00%		CQW49_04050         	100.00%
Bootstrap support for CQW49_RS04060 as seed ortholog is 100%.
Bootstrap support for CQW49_04050 as seed ortholog is 100%.
```

---

### Group of orthologs #1029. Best score 794 bits Score difference with first non-orthologous sequence - OB3b\_Refseq\_for\_inparanoid.fasta:794 OB3b\_Genbank\_for\_inparanoid.fasta:794

```
CQW49_RS09570       	100.00%		CQW49_09550         	100.00%
Bootstrap support for CQW49_RS09570 as seed ortholog is 100%.
Bootstrap support for CQW49_09550 as seed ortholog is 100%.
```

---

### Group of orthologs #1030. Best score 794 bits Score difference with first non-orthologous sequence - OB3b\_Refseq\_for\_inparanoid.fasta:794 OB3b\_Genbank\_for\_inparanoid.fasta:794

```
hpnH                	100.00%		CQW49_17120         	100.00%
Bootstrap support for hpnH as seed ortholog is 100%.
Bootstrap support for CQW49_17120 as seed ortholog is 100%.
```

---

### Group of orthologs #1031. Best score 793 bits Score difference with first non-orthologous sequence - OB3b\_Refseq\_for\_inparanoid.fasta:793 OB3b\_Genbank\_for\_inparanoid.fasta:793

```
CQW49_RS00400       	100.00%		CQW49_00400         	100.00%
Bootstrap support for CQW49_RS00400 as seed ortholog is 100%.
Bootstrap support for CQW49_00400 as seed ortholog is 100%.
```

---

### Group of orthologs #1032. Best score 793 bits Score difference with first non-orthologous sequence - OB3b\_Refseq\_for\_inparanoid.fasta:793 OB3b\_Genbank\_for\_inparanoid.fasta:793

```
CQW49_RS00840       	100.00%		CQW49_00840         	100.00%
Bootstrap support for CQW49_RS00840 as seed ortholog is 100%.
Bootstrap support for CQW49_00840 as seed ortholog is 100%.
```

---

### Group of orthologs #1033. Best score 793 bits Score difference with first non-orthologous sequence - OB3b\_Refseq\_for\_inparanoid.fasta:793 OB3b\_Genbank\_for\_inparanoid.fasta:793

```
CQW49_RS06625       	100.00%		CQW49_06610         	100.00%
Bootstrap support for CQW49_RS06625 as seed ortholog is 100%.
Bootstrap support for CQW49_06610 as seed ortholog is 100%.
```

---

### Group of orthologs #1034. Best score 793 bits Score difference with first non-orthologous sequence - OB3b\_Refseq\_for\_inparanoid.fasta:793 OB3b\_Genbank\_for\_inparanoid.fasta:793

```
CQW49_RS12235       	100.00%		CQW49_12200         	100.00%
Bootstrap support for CQW49_RS12235 as seed ortholog is 100%.
Bootstrap support for CQW49_12200 as seed ortholog is 100%.
```

---

### Group of orthologs #1035. Best score 793 bits Score difference with first non-orthologous sequence - OB3b\_Refseq\_for\_inparanoid.fasta:793 OB3b\_Genbank\_for\_inparanoid.fasta:793

```
[truncated: 1,178,327 more chars]
