# Supplementary material for: Genome-Scale Metabolic Model Reconstruction and in Silico Investigations of Methane Metabolism in Methylosinus trichosporium OB3b
Source: Microorganisms. 2020 Mar 20;8(3):437. doi: 10.3390/microorganisms8030437 (PMC7144005; doi:10.3390/microorganisms8030437)
Supplement: Supplementary file 1 [file microorganisms-08-00437-s001.zip › supplementary_materials/Supplementary materials explanations.docx]

**memote_m_trichosporium: A genome-scale metabolic model for *Methylosinus trichosporium* OB3b**

**Model description:**

This repository contains the genome-scale metabolic model for *Methylosinus trichosporium* OB3b. The model contains 1043 reactions, 1020 metabolites, 683 genes (as of 18/02/2020). The model file is available in json and sbml formats in model_files folder above.

**MemoteReportApp.html**

- Memote report with the extended model statistics. The report was generated with the help of memote web tool <https://memote.io/>

**Escher metabolic map**

- Escher metabolic map of selected biochemical pathways of iMsOB3b in .json and .png formats. The map was drawn using Escher web tool which is available at <https://escher.github.io/>

**GEM reconstruction supplementary materials**

- InParanoid outputs of ortholog analysis between *Methylorubrum extorquens* AM1 vs *Methylosinus trichosporium* OB3b translated coding sequences; and between *Methylosinus trichosporium* OB3b Refseq vs *Methylosinus trichosporium* OB3b Genbank-annotated translated coding sequences.
- Lists of candidate reactions from BiGG-compliant metabolic models which were either added or not added to the model.
- pkgs.txt file which contains the environment configuration with which supplementary scripts described above were written.

**Simulation files**

- model confirmation folder contains 2 files:

1. parameter_fit_3_modes.xls --> In silico uptake rates, growth predictions and molar ratios to confirm growth rate and mode of electron transfer to pMMO

2. redox_arm_different_nitrogen_sources.xls --> In silico uptake rates, growth predictions and molar ratios for iMsOB3b in different environmental conditions with the redox arm as the mode of electron transfer.

- flux distribution folder contains 6 files:

1. falda_knocked_out.csv --> flux distributions when spontaneous formaldehyde condensation reaction (model reaction ID: FALDA) is knocked out

2. acetyl_coA_flux_33_from_pyruvate.csv --> flux distributions when 1/3 of acetyl-CoA comes from pyruvate

3. reverse_TCA_AKGDH_reversible_OOR3r_blocked.csv --> flux distributions when 2-oxoglutarate (model reaction ID: AKGDH) is allowed to be produced from succinyl-CoA via reversible 2-oxoglutarate dehydrogenase reaction

4. reverse_TCA_AKGDH_blocked_OOR3r_active.csv --> flux distributions when 2-oxoglutarate is allowed to be produced from succinyl-CoA via forward 2-oxoacid ferredoxin synthase reaction (model reaction ID: OOR3r) only

5. reverse_TCA_AKGDH_active_OOR3r_active.csv --> flux distributions when both irreversible 2-oxoglutarate dehydrogenase reaction (model reaction ID: AKGDH) and 2-oxoacid ferredoxin synthase reaction (model reaction ID: OOR3r) are allowed to carry flux.

6. reduced_costs_redox_arm_falda_knockout.csv --> reduced costs analysis when spontaneous formaldehyde condensation reaction (model ID: FALDA) is knocked out

**Supplementary scripts**

1. Check GEM for sanity.ipynb --> Add and explore modes of electron transfer to pMMO, fix directions of ATP-producing reactions, remove proton leakage outside the GEM, fix ammonia-assimilation.

2. Adapt nitrogen metabolism.ipynb --> An attempt to include subsystems information to the model, addition of nitrogen metabolism, quinone fix since there were 2 separate ubiquinone metabolites in the model.

3. Refine central carbon metabolism for AM1-based OB3b GEM.ipynb --> Changes model's Refseq-based gene reaction rules into Genbank-based gene reaction rules. This change was required because Methylosinus trichosporium's OB3b's organism-specific KEGG database uses Genbank-based genes to predict functional enzymes. The script also curates central carbon metabolism based on genome annotations, literature review and KEGG-database information.

4. Add reactions not present in OB3b GEM.ipynb --> Creates a list of Methylosinus trichosporium OB3b locus tags which are not included in the model. Adds new reactions based on the found locus tags with the help of either Methylosinus trichosporium OB3b's organism-specific KEGG-pathway database or models of organisms related to OB3b.

5. Curate electron transport chain and nitrogen metabolism.ipynb --> Curates electron transport chain and nitrogen metabolism based on organism-specific KEGG database and literature information.

6. Validate the model.ipynb --> Compares predicted oxygen:methane molar consumption ratios and specific growth rates to the available experimental data to validate the model. The environmental condition used for the comparison is primarily nitrate-supplemented copper-added medium. In addition to that, the script checks model's in silico growth rate predictions and other parameters in other enviromental conditions (nitrogen, or ammonia as nitrogen source and copper-free medium).

7. Small fixes.ipynb --> Applies cosmetic changes to the model such as the removal of genes, reactions and metabolites unused in the model. Fixes model compartments too.

8. Apply omics information to the model.ipynb --> Forces 1/3 of acetyl-CoA to be produced from 2 separate sources. Checks the plausability of reverse TCA in the model. Checks CO2 supplementation effect on the model.
