# Supplementary figures and images for: Genome-Scale Metabolic Model Reconstruction and in Silico Investigations of Methane Metabolism in Methylosinus trichosporium OB3b
Source: Microorganisms. 2020 Mar 20;8(3):437. doi: 10.3390/microorganisms8030437 (PMC7144005; doi:10.3390/microorganisms8030437)

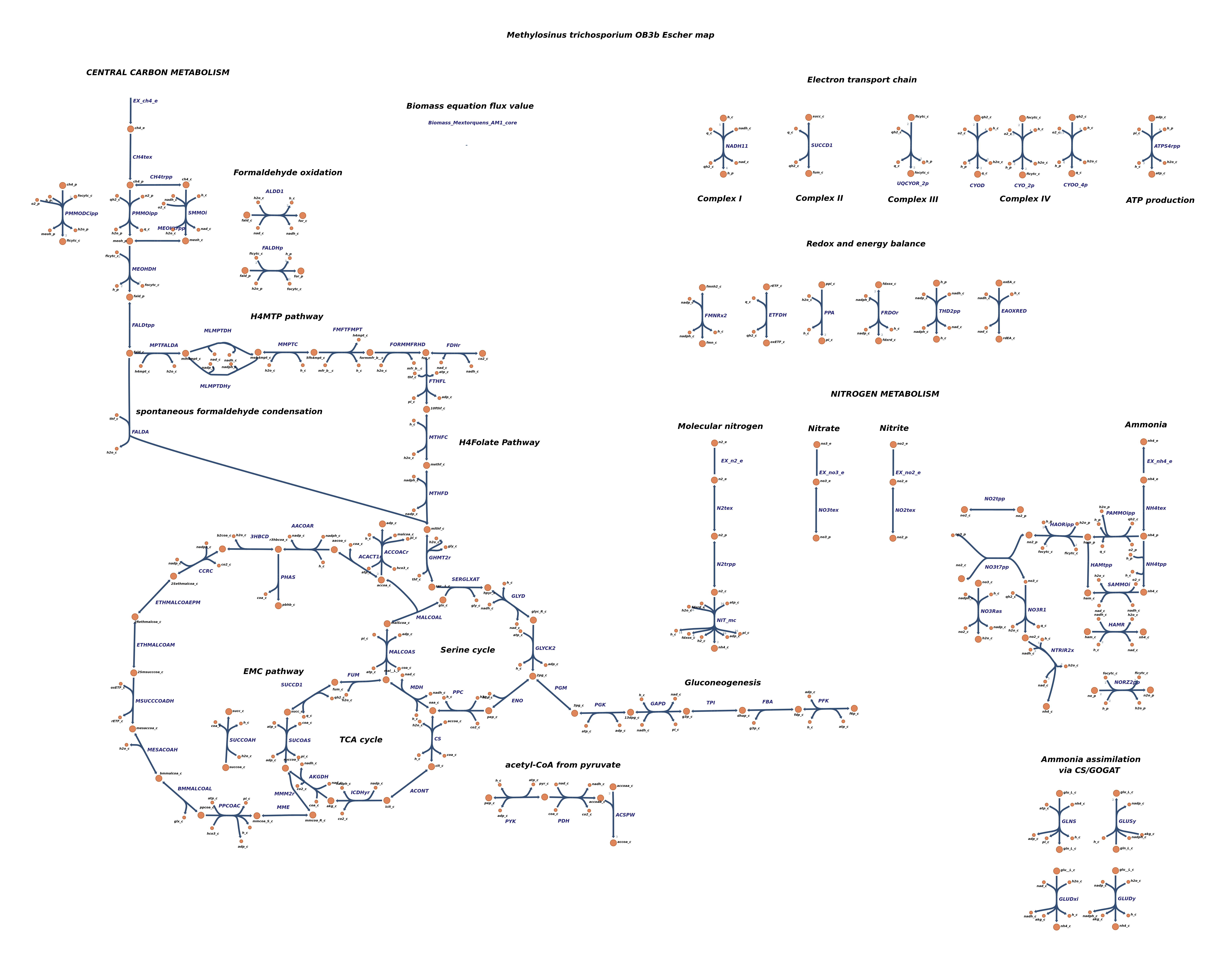

Supplement: Supplementary file 1 [file microorganisms-08-00437-s001.zip › supplementary_materials/escher_metabolic_map/Methylosinus_trichosporium_OB3b_map.png]
